# Dynamic proteomics reveals that endosperm weakening plays a critical role during seed germination in *Polygonatum cyrtonema* Hua

**DOI:** 10.3389/fpls.2025.1662175

**Published:** 2025-11-18

**Authors:** Huilong Xu, Yongsheng Wang, Zhiming Chen, Rongyu Huang, Xinyu Liu, Mengting Pan, Nan Yang, Lianghua Chen, Wen Xu, Fangyu Chen

**Affiliations:** 1 College of Pharmacy, Fujian University of Traditional Chinese Medicine, Fuzhou, Fujian, China; 2 Anhui Engineering Research Center for Intelligent Computing and Information Innovation, Fuyang Normal University, Fuyang, Anhui, China; 3 School of Life Sciences, Xiamen University, Xiamen, Fujian, China; 4 Key Laboratory of Fujian Province for Physiology and Biochemistry of Subtropical Plant, Fujian Institute of Subtropical Botany, Xiamen, Fujian, China; 5 Key Laboratory of Ministry of Education for Genetics, Breeding and Multiple Utilization of Crops, Key Laboratory of Ministry of Agriculture and Rural Affair for Biological Breeding for Fujian and Taiwan Crops, Fujian Provincial Key Laboratory of Crop Breeding by Design, Fujian Agriculture and Forestry University, Fuzhou, China

**Keywords:** *Polygonatum cyrtonema* Hua, endosperm weakening, seed germination, proteomics, transcriptomics, plant hormones

## Abstract

**Introduction:**

*Polygonatum cyrtonema* Hua, a valued Chinese medicinal herb, faces challenges in cultivation and seedling quality due to seed dormancy, a combinational type resulting from multiple factors whose molecular mechanisms remain unclear.

**Methods:**

This study employed iTRAQ-based proteomics, transcriptomics, and hormone profiling to analyze three key germination stages (S1, pulp removal and initial imbibition. S2, radicle emergence through the seed coat. S3, transition phase between primary root elongation and cotyledon emergence).

**Results:**

Morphological observations indicated gradual endosperm weakening and embryo development during germination. Transcriptomics highlighted 30 enriched pathways, notably plant hormone signaling and starch and sucrose metabolism. Proteomics revealed consistent enrichment of FOG: RRM domain, aspartyl protease, and β-fructofuranosidase across comparisons. Hormone assays showed decreases in ABA, bioactive GAs, IAA, JA, and SA from S1 to S3, with a marked increase in the bioactive GAs/ABA ratio. Integrated omics emphasized metabolism and hormone signal transduction. Key enzymes in starch and sucrose metabolism (e.g., β-fructofuranosidase, α-xylosidase, β-D-xylosidase, and β-glucosidase) increased, supporting energy conversion and endosperm weakening. Conversely, ABA-related proteins (PYL4, PP2C) decreased. Sucrose synthase, involved in ABA-GA antagonism, also declined.

**Discussion:**

These results underscore synergistic endosperm weakening and hormonal regulation during *P. cyrtonema* seed germination, offering new insights for improving germination in species constrained by endosperm strength.

## 1 Introduction

*Polygonatum cyrtonema* Hua (*P. cyrtonema*) is a member of the Liliaceae family and is widely distributed in southern China (Xia et al., 2022; Xie et al., 2023). The dried rhizomes of *P. cyrtonema*, known as Huangjing (HJ), have been a renowned traditional medicinal resource in China for over two thousand years, with benefits to the lung, spleen, kidney, and other organs (Zhao et al., 2018; Ye et al., 2025). Recently, with the rapid development of the health industry in China, HJ has garnered significant attention for its potential to treat fatigue, diabetes, and premature graying of hair. The increasing demand for HJ raw materials has led to the overexploitation of natural resources, rendering *P. cyrtonema* an endangered species. Currently, asexual propagation via rhizome is the predominant method for cultivating *P. cyrtonema*. However, this method often results in germplasm degradation, severe pest and disease issues, and quality deterioration (Pang et al., 2022; Liu et al., 2017; Li et al., 2021a; Su et al., 2018). Therefore, seed propagation may offer an ideal solution to these challenges.

The seed dormancy in *Polygonatum, such as P. cyrtonema*, *Polygonatum sibiricum* Red. (*P. sibiricum*), *Polygonatum kingianum* Coll. et Hemsl (*P. kingianum*) and *Polygonatum macranthum* (Maxim.) Koidz., results from the synergistic interactions of multiple factors, including the seed coat, embryo, endosperm, and endogenous inhibitors (Bao, 2018; Baskin and Baskin, 2004; Cheng et al., 2018; Liu et al., 2025; Liao et al., 2021; Wang et al., 2022; Lekamge et al., 2020). This type of dormancy is a composite form that combines physiological, morphological, and physical dormancy, and is classified as combinational dormancy. It represents a natural adaptive mechanism that has evolved to withstand unfavorable environmental conditions (Hashimshony et al., 2024; Finch-Savage and Leubner-Metzge, 2006; Xu et al., 2025). Under natural conditions, it takes at least one and a half years for *Polygonatum* seeds to germinate after being released from mature capsules (Bao, 2018; Baskin and Baskin, 2004; Cheng et al., 2018; Liu et al., 2025; Liao et al., 2021; Wang et al., 2022; Lekamge et al., 2020). This prolonged germination period severely limits the expansion of its planting scale. The waxy and hard seed coats, composed of lignified cells, prevent water absorption and inhibit germination (Zhu et al., 2020; Liu et al., 2021a). The embryo of fresh seeds is structurally simple and underdeveloped, and it undergoes a morphological after-ripening stage (Hashimshony et al., 2024; Finch-Savage and Leubner-Metzge, 2006; Xu et al., 2025; Liu et al., 2025; Liao et al., 2021). The immature embryo is encapsulated by a thick, rigid endosperm, a process closely linked to changes in endogenous hormone levels (Bao, 2018; Zhu et al., 2020; Chen et al., 2020; Duan et al., 2023; Liu et al., 2025). The endosperm of *Polygonatum* is composed of multiple cell layers, serving as a critical mechanical barrier during seed development and germination (Liu et al., 2025; Liu et al., 2021a). It not only provides nutritional support to the embryo but also facilitates extensive bidirectional molecular communication, exemplified by the transport of plant hormones between the endosperm and embryo, thereby modulating embryonic growth and seed germination processes (Zhang et al., 2025). Successful seed germination requires the growth of the embryo and the weakening of the endosperm. Endosperm weakening is considered a prerequisite for radicle emergence in some species (Zhang et al., 2025). During germination, *Polygonatum* seeds undergo a significant process of endosperm weakening (Liao et al., 2021; Liu et al., 2021a). However, the molecular mechanisms underlying this process remain unclear. Elucidating these mechanisms may help to break dormancy and shorten the breeding cycle.

So far, studies on the germination of *P. cyrtonema, P. sibiricum and P. kingianum*, seeds using transcriptomics technology have been reported, which have partially elucidated their physiological and molecular mechanisms (Liu et al., 2021a; Duan et al., 2023; Liao et al., 2021; Wang et al., 2022). However, proteins are the fundamental molecular entities that drive vital biological processes. Changes at the transcriptional level do not always translate into corresponding protein expression, and there is often a poor correlation between mRNA and protein levels. Therefore, an integrated proteomic and transcriptomic analysis is essential for identifying the regulatory networks that govern the expression of seed germination-related proteins and genes (Zhang et al., 2025). In this study, we employed iTRAQ-based proteomics, combined with morphological observations, transcriptomics, and hormone profiling, to analyze three key stages of *P. cyrtonema* seed germination (S1: pulp removal and initial imbibition; S2: radicle emergence through the seed coat; S3: transition phase between primary root elongation and cotyledon emergence), aiming to unravel the internal dynamic functional networks.

## 2 Materials and methods

### 2.1 Plant materials and treatments

The seeds were collected in October from Minhou County, Fuzhou City, Fujian Province (26°4′39.48″N, 119°10′54.27″E). Following pulp removal, the freshly harvested seeds were rinsed and immediately underwent an after-ripening process through cold stratification at 4 °C in moist sand for approximately 120 days. This moist-chilling treatment was applied to break physiological dormancy and promote metabolic preparation for germination. After stratification, the seeds were transferred to a growth chamber set at 25 °C with a 14/10 h light/dark cycle to induce germination. Germination progression and seedling development were monitored every 5 days. To elucidate the germination dynamics, three critical stages were defined. At each stage, biological replicates (n=10 seeds per sample) were randomly harvested, quenched in liquid nitrogen and stored at −80 °C for subsequent experiment and analysis. Three independent biological replicates were performed in this study.

### 2.2 Transcriptomic analysis

Total RNA was extracted from seeds using the Trizol method. The concentration and integrity of the total RNA were assessed. Total RNA was used for library preparation following the manufacturer’s protocol (New England Biolabs, Ipswich, MA, USA). All nine libraries, representing three different stages with triplicate biological replicates designated as S1-1, S1-2, S1-3; S2-1, S2-2, S2-3; S3-1, S3-2, S3-3, were sequenced on the Illumina HiSeq X Ten platform (Illumina, San Diego, CA, USA), with services exclusively provided by Biomics Biotech Co., Ltd. (Beijing, China). The raw sequencing data have been submitted to the NCBI’s Sequence Read Archive (SRA), with the accession number PRJNA1013739. To acquire clean reads, adaptors, poly-N sequences, and low-quality bases were eliminated. Subsequently, the HISAT2 tools software was employed to map the clean reads to the transcriptome sequence. Gene function annotation was carried out using multiple databases, namely Non-redundant (NR), euKaryotic Ortholog Groups (KOG), Clusters of Orthologous Groups (COG), Gene Ontology (GO), Swiss-Prot, Kyoto Encyclopedia of Genes and Genomes (KEGG), and Protein family (Pfam). The Fragments per Kilobase of transcript per Million fragments mapped (FPKM) values were used to represent gene expression levels.

The raw sequencing data have been deposited in the NCBI’s Sequence Read Archive (SRA; accession number: PRJNA1013739). Adaptors, poly-N sequences, and low-quality bases were removed to obtain clean reads. Gene function was annotated using the following databases: NR, KOG, COG, GO, Swiss-Prot, KEGG, and Pfam. Gene expression levels were represented by FPKM.

### 2.3 Proteomic analysis

The seed samples used for iTRAQ analysis consisted of the same three biological replicates as those utilized in the RNA-seq experiment. Protein extraction, concentration, tryptic digestion, iTRAQ labeling, and LC-MS/MS analysis were performed according to the method described by Chen et al (Chen et al., 2022). Briefly, digested peptides were desalted using a Sep-Pak C18 column (Waters, Milford, MA, USA). The column was washed with 300 µL of Milli-Q water, and peptides were eluted with 50 µL of methanol. The eluate was dried by vacuum centrifugation and stored at –80 °C until further use. Peptide labeling was performed using an iTRAQ 4-plex kit (Applied Biosystems, USA) following the manufacturer’s protocol. Labeled peptides were also dried by vacuum centrifugation and stored at –80 °C prior to LC-MS/MS analysis.

LC-MS/MS analysis was carried out on a nanoElute system (plug-in V1.1.0.27; Bruker, Bremen, Germany) coupled to a timsTOF Pro mass spectrometer (Bruker, Bremen, Germany) equipped with a CaptiveSpray ion source. Raw data were processed using Peaks Studio X software (Bioinformatics Solutions Inc., Waterloo, ON, Canada). The mass spectrometry proteomics data have been deposited to the ProteomeXchange Consortium with the dataset identifier PXD045963. To facilitate accurate protein identification, a species-specific protein database was constructed. This was achieved by first performing a *de novo* transcriptome assembly on RNA-seq data derived from the same seed batch, followed by predicting the coding sequences using TransDecoder (v5.5.0) with default parameters. All acquired MS/MS spectra were then queried against this custom database. A false discovery rate (FDR) threshold of ≤1% was applied, and the significance score [-10×log(p)] was calculated, where p denotes the probability that a peptide match occurred by chance.

### 2.4 Bioinformatics analysis

The identification of significant differentially accumulated proteins (DAP) was filtered using thresholds of Q-value < 0.05 and |fold change (FC)| > 1.2, while DESeq2 software (based on a negative binomial distribution) was employed to analyze raw counts and identify differentially expressed genes (DEGs) from shared genes between all stages with the widely accepted criteria of |log_2_ FC| ≥ 1 and adjusted *P*-value < 0.05. GO enrichment analysis was performed using the BiNGO plugin in Cytoscape, with an FDR cutoff of 0.05 for both DEGs and DAP. KEGG enrichment analysis was conducted using the KOBAS software for DEGs and DAP.

### 2.5 Quantification of endogenous phytohormones

The quantification of endogenous phytohormones was conducted by MetWare (Wuhan, China). To measure the concentrations of phytohormones, including ABA, indole-3-acetic acid (IAA), jasmonic acid (JA), methyle jasmonate (MeJA), salicylic acid (SA), methyle salicylic acid (MeSA), gibberellin 1 (GA_1_), gibberellin 3 (GA_3_), gibberellin 4 (GA_4_), gibberellin 6 (GA_6_), gibberellin 7 (GA_7_), gibberellin 8 (GA_8_), gibberellin 9 (GA_9_), gibberellin 12-ald (GA_12_-ald), gibberellin 15 (GA_15_), gibberellin 19 (GA_19_), gibberellin 20 (GA_20_), gibberellin 24 (GA_24_), gibberellin 29 (GA_29_), gibberellin 34 (GA_34_), gibberellin 44 (GA_44_), gibberellin 51 (GA_51_), gibberellin 53 (GA_53_), cis-zeatin-riboside (CZR), trans-zeatin-riboside (TZR), 6-(gamma,gamma-dimethylallylamino) purine (iP), 6-(gamma,gamma-dimethylallylamino) purine riboside (iPR), and 3-indolebutyric acid (IBA) were purchased from Sigma Aldrich (USA), approximately 1 g of seeds was ground in liquid nitrogen and extracted with acetonitrile.

The chromatographic separation was carried out on an ACQUITY UPLC CSH C18 column (2.1 × 100 mm, 1.7 µm; Agilent Technologies, Inc.). The mobile phase was composed of 0.05% (v/v) formic acid in a water–acetonitrile mixture (5:95, v/v). An injection volume of 10.0 µL was used, and the flow rate of the mobile phase was maintained at 0.35 mL/min. The column temperature was kept constant at 40 °C throughout the analysis. Phytohormones were detected and quantified in each sample using a UPLC-ESI-MS/MS system (UPLC, ExionLC™ AD, https://sciex.com.cn/, MS, QTRAP^®^ 6500+, https://sciex.com.cn/) after the addition of internal standards prior to grinding.

## 3 Results

### 3.1 Morphological observation of the seed germination process of *P. cyrtonema*

**Figure 1:**
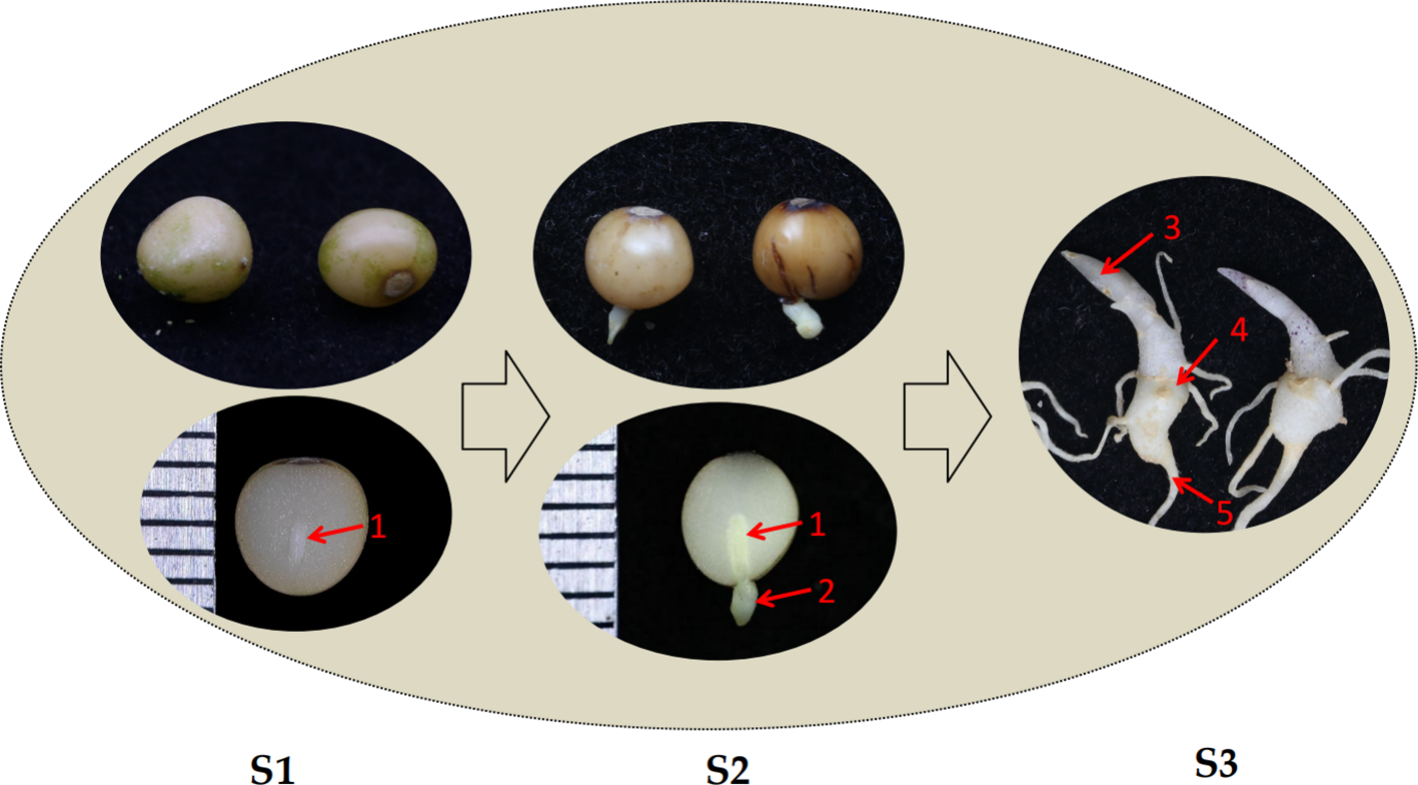
Three different stages of seed germination in *P. cyrtonema*. S1, pulp removal and initial imbibition. S2, radicle emergence through the seed coat. S3, transition phase between primary root elongation and cotyledon emergence. The arrow indicates key structures: 1, Embryo. 2, Radicle. 3, Cotyledon. 4, Primary rhizome. 5, Root.

*P. cyrtonema* flowered from May to June and subsequently fruited from August to October. Fresh seeds extracted from mature capsules at the S1 stage had not yet achieved physiological maturity. At this stage, the endosperm occupied the majority of the seed volume, while the embryo showed no obvious differentiation, appearing rod-shaped and encapsulated by both the endosperm and the seed coat (**Figure 1**-left). After approximately 4 months of low-temperature storage, the endosperm near the seed pore began to degrade in the S2 stage (**Figure 1**-middle). During this stage, the embryo protruded from the seed pore, broke through the seed coat, and the seed commenced germination. As germination progressed, the embryo differentiated to form the primary rhizome and cotyledons during the S3 period, and the endosperm was fully absorbed (**Figure 1**-right). These three stages were critical in the process of embryo development and endosperm weakening. Investigating the differential genes, proteins, and metabolites during these periods might elucidate the molecular mechanisms underlying dormancy release and germination in *P. cyrtonema* seeds.

### 3.2 Identification and analysis of DEGs

To investigate transcriptional dynamics during seed germination, nine samples representing three germination stages were subjected to RNA sequencing using the Illumina HiSeq platform. The RNA-Seq data are summarized in **Table 1**, **Supplementary Table S1**, and **Supplementary Text S1**, revealing the assembly of 197,609 unigenes with an N50 value of 270 bp. Functional annotation demonstrated that 775,597 (88.45%) unigenes showed homology to known sequences in the NR database, followed by 53,754 (61.27%) in KOG, 47,394 (54.02%) in COG, 43,817 (49.95%) in GO, 40,423 (46.08%) in Swiss-Prot, 40,112 (45.72%) in KEGG, and 30,132 (3.35%) in Pfam. Comparative genomic analysis revealed distinct sequence homology patterns: 19.35% of unigenes showed similarity to *Asparagus officinalis*, 6.40% to Fusarium sp., 5.68% to *Rhizoctonia solani*, with decreasing proportions for *Elaeis guineensis* (2.21%), *Neonectria ditissima* (2.18%), *Phoenix dactylifera* (1.63%), *Ensete ventricosum* (1.24%), *Nothophytophthora* sp. (1.16%), *Fusarium oxysporum* (0.78%), and *Fusarium ambrosium* (0.75%). Notably, 58.63% of sequences exhibited homology to other uncharacterized genomes (**Supplementary Figure S1**).

**Table 1:** Statistics and functional annotations of unigenes in 9 RNA sequencing libraries.

Category	Number of unigenes	Percentage (%)
>200 bp	197,609	100
>300 bp	25,966	13.14
≥1000 bp	12,652	6.40
N50	270	–
Max length	15,892	–
Min length	196	–
Average length	374	–
NR	775,597	88.45
Swiss-Prot	53,754	61.27
Pfam	47,394	54.02
KOG	43,817	49.95
GO	40,423	46.08
KEGG	40,112	45.72
COG	30,132	3.35

**Figure 2:**
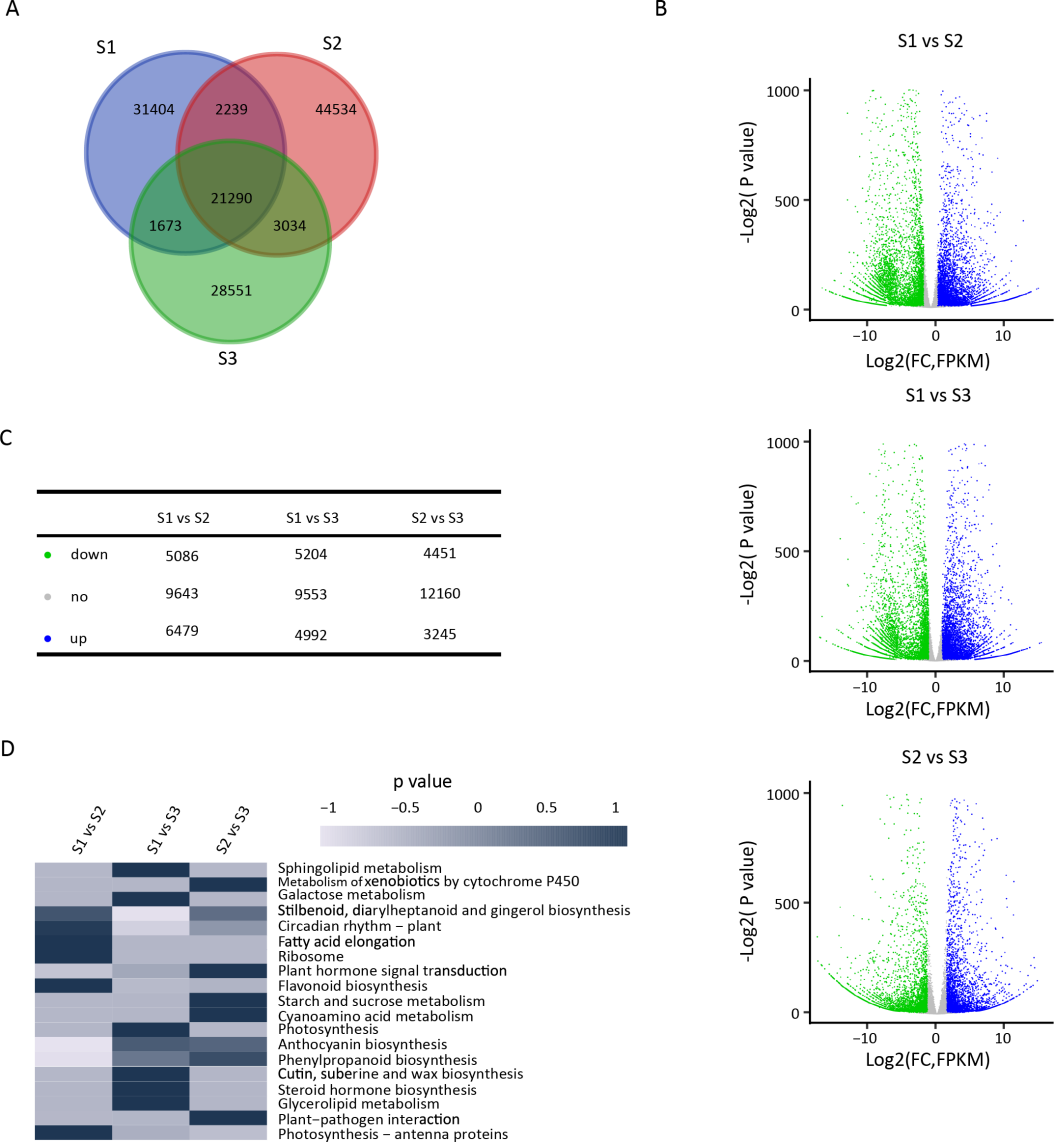
Identification of differentially abundant assembled unigenes during different stages of seed germination. **(A)** Number of identified unigenes in three different stages of seed germination. **(B)** Volcano Plot indicating the differentially expressed assembled unigenes during different stages of seed germination. **(C)** Table showing the number of differentially expressed unigenes (upregulated, downregulated, and no-regulated). Blue, green and gray points represent upregulated, downregulated, and no-regulated unigenes, respectively. **(D)** KEGG enrichment analysis performed using all differentially expressed assembled unigenes.

As shown in **Supplementary Figure S2**, the correlation coefficients of sample replicates were very high, indicating that the sequencing data were highly reliable. The number of unigenes identified at S1, S2, and S3 was 56,606, 71,097, and 54,818, respectively, with only 21,290 unigenes common to all three groups (**Figure 2A**). The number of DEGs between stages was as follows: 11,565 DEGs between S1 and S2, 10,196 between S1 and S3, and 7,696 between S2 and S3 (**Supplementary Table S2**). As illustrated in **Figures 2B, C**, the number of upregulated and downregulated DEGs varied across the comparisons. According to KEGG enrichment analysis, these DEGs were associated with 339, 340, and 321 pathways, respectively, of which 30 pathways were significantly enriched (**Figure 2D**). Among these pathways, those related to seed germination, phenylpropanoid biosynthesis, stilbenoid, diarylheptanoid, and gingerol biosynthesis, flavonoid biosynthesis, plant hormone signal transduction, and starch and sucrose metabolism exhibited the highest levels of enrichment. Therefore, the results suggest that plant hormone signal transduction pathways, as well as starch and sucrose metabolism pathways, may play significant roles in seed germination.

### 3.3 Identification and analysis of DAP

**Figure 3:**
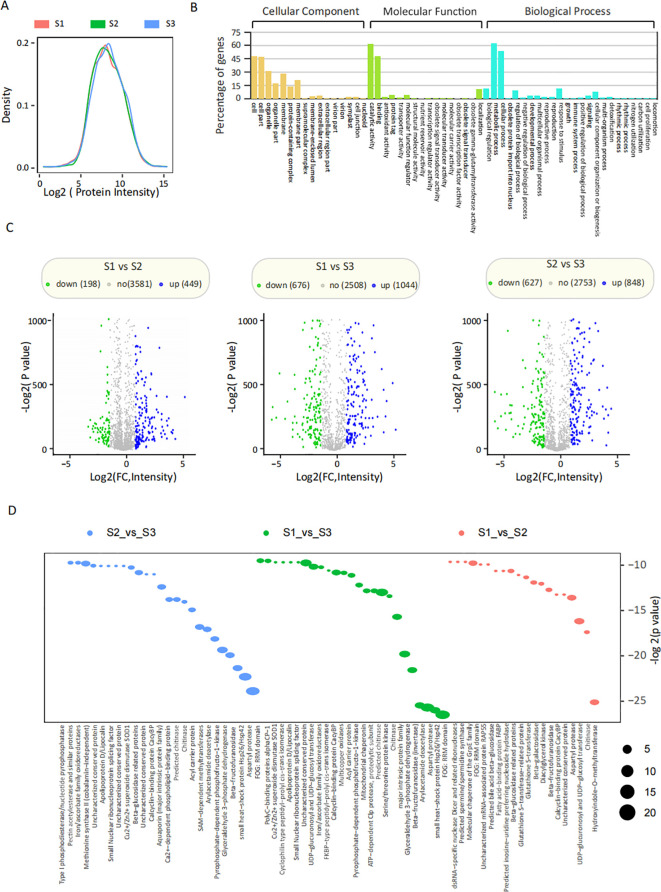
Differentially abundant proteins during different stages of seed germination. **(A)** Abundance of detected proteins in three different stages of seed germination. **(B)** GO enrichment analysis of all detected proteins was performed using the biological process, cellular component, and molecular function terms. **(C)** DAP in different stages of seed germination. The number of up-regulation, down-regulation and non-regulation unigenes were annotated above. The Volcano Plot (below) showed the DAP, with blue, green, and gray points representing increased, decreased, and no-changed unigenes, respectively. **(D)** KEGG enrichment analysis was performed using DAP after pairwise comparisons (e.g., S1 vs S2, S1 vs S3, S2 vs S3).

Seed proteome profiles were analyzed, and a total of 4,228 proteins were detected. The distribution of protein abundance across the three different stages of seed germination exhibited a nearly identical general pattern (**Figure 3A**; **Supplementary Table S3**). These identified proteins were further analyzed for GO enrichment. In the GO analysis (**Figure 3B**), “cell” was the most enriched subclass among cellular components, followed by “cell part,” “organelle,” and “membrane.” Within the category of molecular function, “catalytic activity” ranked highest, followed by “binding” and “localization.” In the biological process category, “metabolic process” was the most enriched, followed by “cellular process” and “biological regulation.” The results revealed that 647 proteins (449 increased and 198 decreased) were differentially expressed between S1 and S2, 1,720 proteins (1,044 increased and 676 decreased) between S1 and S3, and 1,475 proteins (848 increased and 627 decreased) between S2 and S3 (**Figure 3C**). KEGG annotation-based enrichment analysis revealed a set of significantly enriched protein functional terms across different pairwise comparisons. In the S1 vs. S2 comparison, “UDP-glucuronosyl and UDP-glucosyl transferase” was the most prominently enriched term, followed by “FOG: RRM domain”, “hydroxyindole-O-methyltransferase”, and “aspartyl protease”. For S1 vs. S3, “FOG: RRM domain” exhibited the highest enrichment, trailed by “aspartyl protease”, “serine/threonine protein kinase”, and “UDP-glucuronosyl and UDP-glucosyl transferase”. In the S2 vs. S3 comparison, “FOG: RRM domain” was again the most significantly enriched term, followed by “aspartyl protease”, “glyceraldehyde 3-phosphate dehydrogenase”, and “iron/ascorbate family oxidoreductases”. As illustrated in **Figure 3D**, several proteins—notably the FOG: RRM domain, aspartyl protease, and β-fructofuranosidase—were consistently and significantly enriched across all comparisons. This conserved pattern suggests that these proteins may play critical regulatory roles in seed germination.

### 3.4 Integrated proteomic and transcriptomic analysis

**Figure 4:**
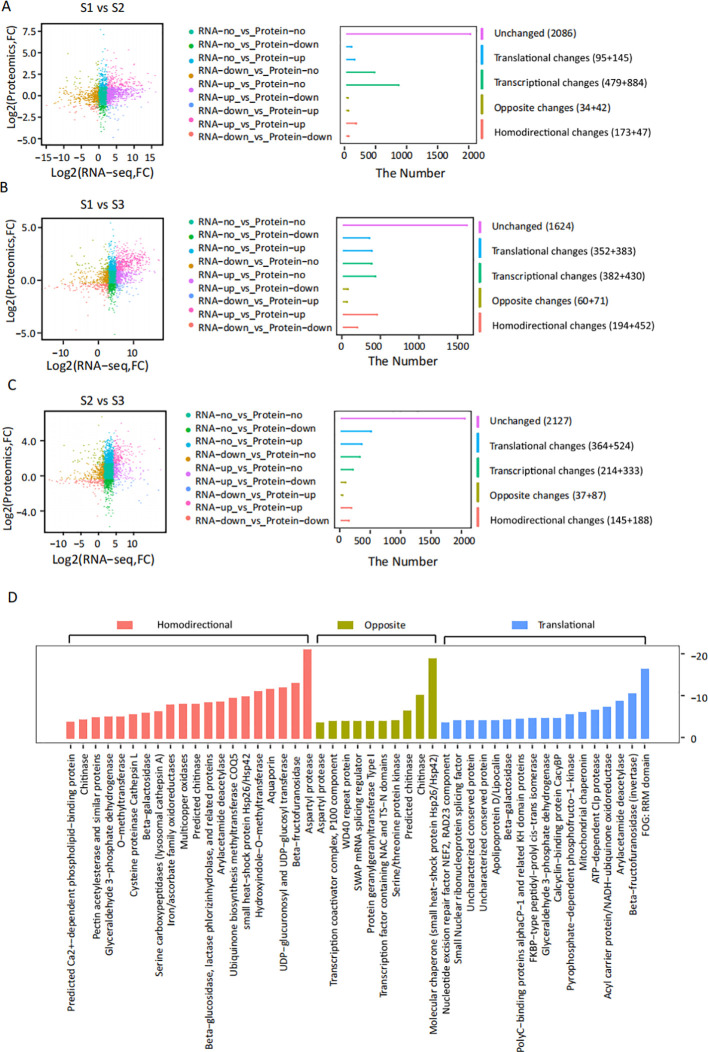
Integrated proteomic and transcriptiomic profiling of seed germination dynamics. **(A–C)** Comparative analysis of transcriptional and translational regulation across developmental stages: **(A)** S1 vs S2, **(B)** S1 vs S3, **(C)** S2 vs S3. Left panels display differential expression profiles (log2 fold-change) with nine molecular clusters color-coded by regulation patterns. Right histograms quantify five functional modules: unchanged (|fold change| <2), translational regulation (alterations in protein accumulation that are not reflected at the RNA level), transcriptional regulation (alterations in RNA accumulation that are not reflected at the protein level), discordant expression (opposite directions, RNA and protein levels are differentially expressed but in contrasting directions), and concordant expression (homodirectional changes, both RNA and protein exhibit coordinated expression changes in the same direction). Significant responses were defined as |fold change| ≥2 with FDR-adjusted q-value ≤0.01. **(D)** Pathway enrichment patterns of three core regulatory modules: concordant expression (red), discordant expression (yellow), and translationally regulated (blue) clusters.

To elucidate the regulatory relationship between transcriptional and translational processes, we conducted a systematic correlation analysis of proteomic and transcriptomic data. Comparative expression profiles of genes and their corresponding proteins across different experimental groups (S1, S2, S3) were presented in **Figures 4A–C** and **Supplementary Table S4**. The analysis revealed distinct patterns of expression coordination. A comparative analysis revealed distinct patterns of expression coordination among the three sample groups. The most significant translational changes were observed in the S2 vs. S3, accounting for 66.0% of all changes. Opposite changes were predominant in S1 vs. S2, making up 42.7% of the changes, whereas homodirectional changes were the main feature in S1 vs. S3 (14.2%). Notably, S2 vs. S3 exhibited the highest translational ratio and the lowest opposite/homodirectional ratio, indicating that translational changes played a predominant role in the late stage of seed germination.

KEGG pathway enrichment analysis of these differentially expressed modules identified key functional categories (**Figure 4D**). The concordant expression group showed predominant enrichment of aspartyl proteases (EC 3.4.23), β-fructofuranosidases (EC 3.2.1.26), and UDP-glycosyltransferases (EC 2.4.1.-). Conversely, the discordant expression category was dominated by molecular chaperones (particularly small heat-shock proteins Hsp26/Hsp42), followed by chitinase-related enzymes (EC 3.2.1.14). The translational regulation group exhibited significant enrichment of RNA-binding proteins containing FOG: RRM domains, along with β-fructofuranosidase (invertase) and arylacetamide deacetylase (EC 3.1.1.-) activities. Comprehensive pathway mapping identified three principal functional clusters associated with *P. cyrtonema* seed germination: (1) Genetic Information Processing (Ribosome, protein processing in the endoplasmic reticulum, spliceosome, RNA transport); (2) Metabolism (Starch and sucrose metabolism, cyanoamino acid metabolism, anthocyanin biosynthesis, phenylpropanoid biosynthesis, stilbenoid, diarylheptanoid, and gingerol biosynthesis, amino sugar and nucleotide sugar metabolism, ubiquinone and other terpenoid-quinone biosynthesis); (3) Signal transduction (Plant hormone signal transduction). These pathways were likely to play significant roles in seed germination.

### 3.5 The phytohormones associated genes/proteins and phytohormone levels

**Figure 5:**
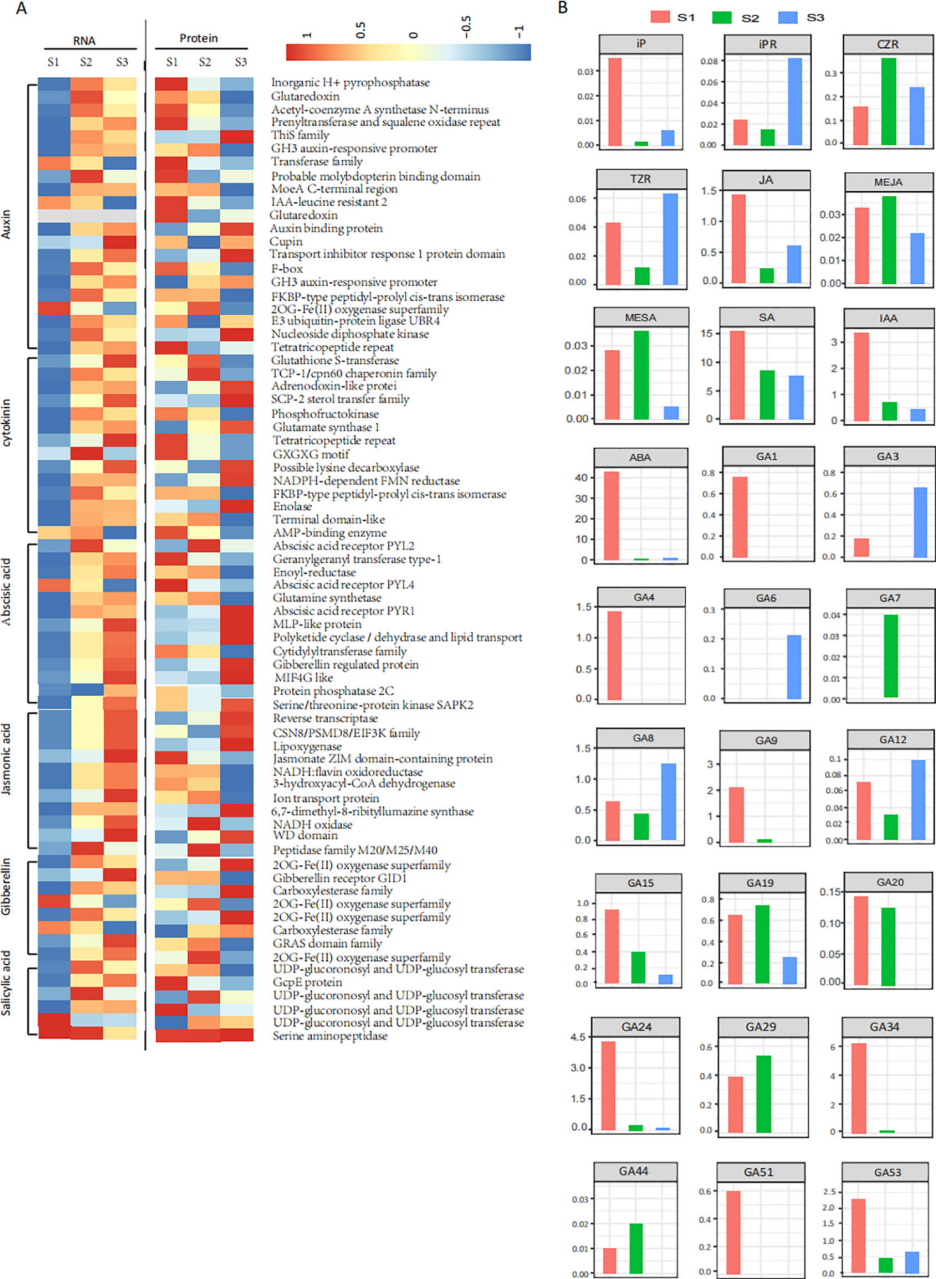
Dynamic changes of hormone-related unigenes at transcriptional and translational levels and hormone distribution during seed germination. **(A)** Heatmap showing the transcriptional and translational levels of seven hormone-related unigenes across three different stages of seed germination. **(B)** Hormone distribution in three different stages of seed germination.

During seed germination, 483 unigenes and 72 proteins were implicated in plant hormone signal transduction (**Figure 5A**; **Supplementary Table S5**). However, only 41 DAP were identified among them. Several phytohormone signal transduction-related genes exhibited consistent trends in mRNA and protein expression changes. For example, genes encoding transport inhibitor response 1 protein (TIR1) and GH3 auxin-responsive promoter (GH3) related to auxin, abscisic acid receptor PYL2 (PYL2), abscisic acid receptor PYR1 (PYR1) and gibberellin-regulated protein (GRP) related to ABA, and NADH oxidase (Nox) related to JA, all showed increased transcripts and protein abundance between S1 and S2 (**Supplementary Table S5**). In contrast, other genes/proteins between S2 and S3, such as those encoding nucleoside diphosphate kinase (NDK), PYR1, PYL2, 2-oxoglutarate-dependent dioxygenase (2OGD) and gibberellin receptor GID1 (GID1), exhibited opposite transcription levels (**Supplementary Table S5**). Overall, the upregulation and downregulation of these genes and proteins could lead to an imbalance of phytohormones during seed germination.

Dynamic profiles of ABA, IAA, JA, MeJA, SA, MeSA, GA_1_, GA_3_, GA_4_, GA_6_, GA_7_, GA_8_, GA_9_, GA_12_-ald, GA_15_, GA_19_, GA_20_, GA_24_, GA_29_, GA_34_, GA_44_, GA_51_, GA_53_, CZR, TZR, iP, iPR, and IBA were analyzed across three developmental stages (S1, S2, S3) of *P. cyrtonema* seeds to track phytohormone dynamics (**Figure 5B**; **Table 2**). ABA, JA, and total GAs exhibited synchronized trends: levels decreased significantly from S1 to S2 but rebounded at S3. The contents of several bioactive GAs (He et al., 2019; Yamaguchi, 2008) changed significantly across stages. For instance, GA_1_, GA_4_, GA_9_, and total bioactive GAs levels decreased, whereas GA_3_, GA_6_, and GA_7_ levels increased from S1 to S2 or S3. Significantly, the bioactive GAs/ABA ratio increased from S1 to S3. Concurrently, most GA precursors, intermediates, and catabolites (e.g., GA_15_, GA_24_, GA_53_, GA_34_, etc.) also exhibited a decreasing trend from S1 to S2 or S3 (He et al., 2019; Yamaguchi, 2008). In contrast, IAA and SA contents declined progressively throughout all stages, with marked reductions from S1 to S3. CKs showed minimal fluctuations and remained at low concentrations, while IBA was undetectable in all samples.

**Table 2:** Levels of phytohormones in three different stages of seed germination.

Hormone types	Hormone	S1	S2	S3
ABA	ABA	42.93±1.19^a^	0.66±0.08^b^	0.95±0.05^b^
GAs	GA_1_	0.73±0.01^a^	0^b^	0^b^
	GA_3_	0.17±0.03^b^	0^a^	0.67±0.10^a^
	GA_4_	1.43±0.007^a^	0^b^	0^b^
	GA_6_	0^b^	0^b^	0.21±0.039^a^
	GA_7_	0^b^	0.04±0.008^a^	0^b^
	GA_8_	0.65±0.017^b^	0.49±0.092^b^	1.21±0.13^a^
	GA_9_	2.01±0.072^a^	0.04±0.006^b^	0^b^
	GA_12_-ald	0.07±0.031^ab^	0.03±0.006^b^	0.10±0.019^a^
	GA_15_	0.94±0.025^a^	0.04±0.002^b^	0.01±0.001^c^
	GA_19_	0.62±0.012^a^	0.72±0.16^a^	0.27±0.047^b^
	GA_20_	0.12±0.011^a^	0.14±0.011^a^	0^b^
	GA_24_	4.37±0.82^a^	0.15±0.035^b^	0.10±0.001^b^
	GA_29_	0.36±0.047^b^	0.53±0.025^a^	0^c^
	GA_34_	6.30±0.52^a^	0.05±0.004^b^	0^b^
	GA_44_	0.01±0.0002^a^	0.02±0.005^a^	0^a^
	GA_51_	0.60±0.041^a^	0^b^	0^b^
	GA_53_	2.21±0.43^a^	0.44±0.029^b^	0.69±0.034^b^
IAA	IAA	3.37±0.18^a^	0.69±0.11^b^	0.44±0.06^c^
IBA	IBA	0^a^	0^a^	0^a^
JAs	JA	1.43±0.07^a^	0.23±0.07^c^	0.61±0.10^b^
	MEJA	0.03±0.007^a^	0.04±0.01^a^	0.02±0.005^b^
SAs	SA	15.41±1.40^a^	8.52±1.19^b^	7.60±0.65^b^
	MESA	0.03±0.009^a^	0.04±0.009^a^	0^b^
CKs	CZR	0.16±0.01^b^	0.36±0.065^a^	0.24±0.05^b^
	TZR	0.04±0.006^b^	0.01±0.002^c^	0.063±0.007^a^
	iP	0.04±0.003^a^	0^b^	0^b^
	iPR	0.02±0.005^b^	0.02±0.005^b^	0.08±0.007^a^
GAs/ABA	bioactive GAs/ABA	0.09±0.003^b^	0.12±0.01^ab^	0.94±0.11^b^

^1^Significant differences between stages are indicated by different letters above the values (P ≤ 0.05).

### 3.6 Multiple molecular networks mediated by differentially abundant proteins

**Figure 6:**
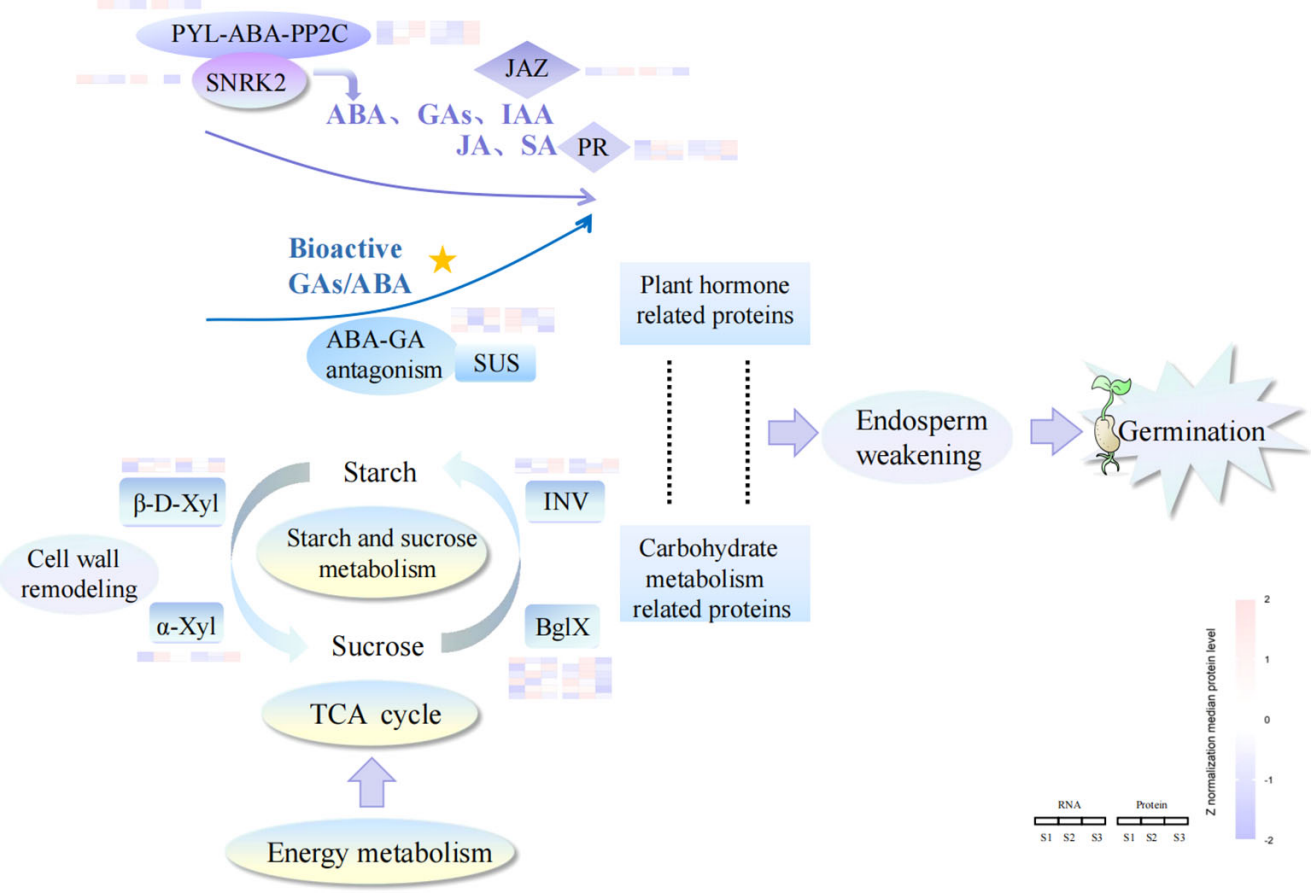
A proposed model of seed germination of *P. cyrtonema* driven by endosperm weakening.

Seed germination involved the dynamic rewiring of molecular networks, orchestrated by differentially abundant biomolecules. **Figure 6** presented a proposed model of seed germination driven by endosperm weakening, which elucidated the germination mechanism of *P. cyrtonema* seeds (**Supplementary Table S6**). It showed that endosperm weakening was closely related to energy metabolism, carbohydrate metabolism and plant hormone signal transduction. Several key proteins in these pathways, such as beta-fructofuranosidase (INV), alpha-xylosidase (α-Xyl), beta-D-xylosidase (β-D-Xyl), and beta-glucosidase (bglX), promoted the conversion of starch into sucrose, and sucrose synthase (SUS) protein could promote the degradation of storage substances by the interaction of gibberellin and abscisic acid.

## 4 Discussion

The dormancy of *P. cyrtonema* seeds is a typical combinational dormancy caused by the combined effects of multiple factors, and its mechanism is relatively complex. Previous studies have reported that sequential physiological after-ripening is required to break the dormancy of *P. cyrtonema* seeds (Bao, 2018; Baskin and Baskin, 2004; Cheng et al., 2018; Liu et al., 2021a; Duan et al., 2023). Naturally, it takes at least two years for the seeds to germinate, and the survival rate of seedlings is low (Bao, 2018; Baskin and Baskin, 2004; Zhang et al., 2023; Cheng et al., 2018). Morphological observations in this study indicated that during the dormancy release process of *P. cyrtonema* seeds, there was a pronounced gradual weakening of endosperm, concurrent with after-ripening growth and development of the embryo until germination (**Figure 1**). *P. cyrtonema* seed germination was comprehensively studied through proteomic and transcriptomic analyses, revealing global changes in protein and mRNA levels (**Figures 2**; **3**; **4**). Furthermore, according to previous studies, there is often little correlation between transcriptomic and proteomic data, with less than 20% of proteins being correlated at the transcriptional level (Ge et al., 2024; Liu et al., 2021b; Guo et al., 2023). This study also obtained consistent results. Therefore, integrated analysis of proteomics and transcriptomics enables the acquisition of more comprehensive molecular information. KEGG annotations showed that stilbenoid, diarylheptanoid, and gingerol biosynthesis, phenylpropanoid biosynthesis, flavonoid biosynthesis, and plant hormone signal transduction were significantly enriched for DEGs (**Figure 2D**), while aspartyl protease, FOG: RRM, and β-fructofuranosidase were significantly enriched for DAP (**Figure 3D**). In fact, many DAP implicated in metabolic and signal pathways have been identified through proteome studies, providing a molecular explanation for seed germination, as these proteins are directly involved in biological activity.

During seed germination, numerous metabolic and structural changes occur, including the mobilization of active reserves, the repair of DNA templates, *de novo* synthesis of proteins, rapid cell division, and so on (Han and Yang, 2015). It is well established that the germination process is regulated by a complex metabolic and signaling network that is highly sensitive to changes in phytohormone levels (Han and Yang, 2015). In studies of *P. cyrtonema* seed germination, pathways such as signal transduction, carbohydrate metabolism, lipid metabolism, amino acid metabolism, and secondary metabolite synthesis have been identified as being involved in germination (Liu et al., 2021a; Duan et al., 2023). In the present study, a total of 115 key proteins were identified across the seed germination process of *P. cyrtonema*. Among these proteins, 57 were related to metabolism, 40 to signal transduction, 21 to organismal systems, 19 to genetic information processing, and 9 to cellular processes.

Seed germination typically initiates through coordinated embryo growth and progressive endosperm weakening. The endosperm acts as a mechanical constraint on embryo growth, and its rupture is a critical step in seed germination (Muller et al., 2006). During seed germination, a variety of metabolic processes are activated in endosperm cells, providing essential nutrients and energy to the developing seedling (Han and Yang, 2015). For example, the substantial amount of starch stored in the endosperm is gradually degraded, serving as a primary source of ATP and a precursor for anabolic reactions in plant embryos (Zhu et al., 2023). In this study, several key proteins related to starch and sucrose metabolism, such as INV, α-Xyl, β-D-Xyl and bglX, which are associated with seed germination (**Figure 6**), exhibited increased abundance between stages S1 and S3. The seeds of *P. cyrtonema* contain a significant amount of starch in the endosperm, which is hydrolyzed into glucose to provide the primary source of energy for seedling emergence (Liu et al., 2021a; Duan et al., 2023). It has been reported that INV, α-Xyl, β-D-Xyl, and bglX are the major enzymes involved in the hydrolysis of starch into glucose (Liu et al., 2020; Hou et al., 2021). In particular, α-Xyl and β-D-Xyl weaken the endosperm cell wall by modulating the structure of xyloglucan during seed germination, leading to cell wall relaxation and endosperm weakening, thereby facilitating radicle emergence (Shigeyama et al., 2016; Arsovski et al., 2009). The decomposition of starch can also promote seed sprouting (Li et al., 2022). Therefore, these metabolism-related proteins may play a crucial role in the gradual weakening of the endosperm, ultimately leading to seed germination.

Signal transduction pathways, particularly plant hormone signaling, control environmental responses during seed dormancy and germination. Plant hormones, including ABA, IAA, and GA, are key regulators of seed germination (Graeber et al., 2012; Nonogaki, 2014; Zeng et al., 2024). For instance, ABA promotes seed dormancy and inhibits germination by suppressing cell wall loosening and expansion (Gimeno-Gilles et al., 2009). In this study, ABA content was higher in S1 (**Figure 5B** and **Table 2**), likely due to the thick and hard endosperm surrounding the seed embryo, which blocks its transport. ABA levels decreased significantly during physiological after-ripening and remained low. Several key proteins involved in ABA signaling, such as abscisic acid receptor PYL4 (PYL4), protein phosphatase 2C (PP2C), and serine/threonine-protein kinase SAPK2-like (SNRK2), exhibited decreased abundance between S1 and S3. ABA binding to PYL4 causes PP2C to lose phosphatase activity, and the inhibition of PP2Cs enables SNRK2 activation (**Figure 6**), which phosphorylates ABA-responsive element-binding factors to induce ABA-related gene expression (Park et al., 2009; Rubio et al., 2009; Zhao et al., 2020; Xue et al., 2021). This suggests that seed germination might be inhibited in S1. In contrast, in S3, PP2C phosphatases inhibit SNRK2, turning off the ABA signal transduction pathway and allowing seeds to start germinating (Park et al., 2009; Wang et al., 2020). Thus, ABA appears to play a major role in regulating *P. cyrtonema* seed germination. IAA and JA have been shown to work synergistically with ABA to regulate seed dormancy and germination (Liu et al., 2013; Shuai et al., 2017; Pan et al., 2020). In this study, IAA and JA contents decreased from S1 to S2 (**Figure 5B**; **Table 2**), aligning with the decreasing trend of ABA. Additionally, 11 proteins related to IAA and JA signaling, including auxin-binding protein (ABP), TIR1, GH3, 2OGD, NDK, Reverse transcriptase (RT), jasmonate ZIM domain-containing protein (JAZ), 3-hydroxyacyl-CoA dehydrogenase (HAD), PYL2, Nox, and 12-oxophytodienoate reductase (OPR), were identified during seed germination (**Figure 5A**). For example, GH3, which was increased between S1 and S3, can conjugate plant hormones such as IAA and JA to amino acids for degradation or storage, thereby downregulating active hormone levels (Khan and Stone, 2007). JAZ, a critical regulator of JA-responsive gene expression (Srivastava et al., 2018), was decreased between S1 and S3, potentially leading to decreased JA levels. It has also been reported that the auxin signaling pathway controls JAZ gene expression through ABA signaling, enabling molecular interplay between IAA, JA, and ABA signaling (Liu et al., 2013; Grunewald et al., 2009). Therefore, IAA and JA actions depend on the ABA signaling pathway, suggesting interdependent roles of IAA, JA, and ABA in seed dormancy and germination. SA has suppressive or promotive effects on seed germination, depending on its concentration and the conditions used (Lee et al., 2010). In our study, SA levels decreased significantly across the three stages (**Figure 5B** and **Table 2**), suggesting that germination of *P. cyrtonema* seeds may be promoted with reduced SA. However, pathogenesis-related protein (PR), which is suggested to play a role in SA-mediated defense signaling (Molinari et al., 2014), increased significantly from S1 to S2/S3 (**Figure 5A**). Additionally, PR-3 has been reported to mediate ABA-dependent salt stress signals affecting seed germination in *Arabidopsis* (Seo et al., 2008). This result may suggest an unusual SA-mediated PR-ABA regulation in *P. cyrtonema* seed germination.

Conversely, GAs promote seed germination by antagonizing the inhibitory effects of ABA, thereby inducing enzymes that accelerate cell division, cell enlargement, endosperm degradation, and so on (Yamaguchi et al., 2001; Werner and Schmülling, 2009). In this study, the total content of bioactive GAs decreased significantly, while the bioactive GAs/ABA ratio increased significantly from S1 to S3. Concurrently, other GAs were quantified to assess whether fluctuations in precursor levels (e.g., GA_19_, GA_20_, GA_9_) or inactivated metabolites (e.g., GA_8_, GA_34_) correlated with changes in bioactive GA concentrations (He et al., 2019; Yamaguchi, 2008), thereby offering deeper mechanistic insight into the regulation of GA metabolism during dormancy release (**Figure 5B**; **Supplementary Figure S3**; **Table 2**). The ratio of ABA to GA regulates seed germination in seeds with endosperm through communication between the embryo and endosperm. ABA produced by the endosperm inhibits embryo growth, while GA synthesized by the embryo promotes germination (Gubler et al., 2005; Finkelstein et al., 2008). Both embryo growth and endosperm weakening during seed germination are regulated by GA and ABA (Belin et al., 2009; Lee et al., 2012). Notably, three core GA-regulatory components were identified throughout the seed germination process of *P. cyrtonema*: GID1, 2OGD, and GRAS domain family (GRAS). GID1 is the canonical GA receptor mediating GA perception. 2OGD is the key biosynthetic enzyme converting ent-kaurene to bioactive GAs. GRAS is the signal integrator coordinating GA-responsive gene networks. These components exhibited stage-specific activation patterns, suggesting their synergistic regulation of GA accumulation (2OGD), signal transduction (GID1), and downstream transcriptional reprogramming (GRAS) during germination progression (Voegele et al., 2011). Moreover, SUS, which is considered to be associated with ABA-GA antagonism (Li et al., 2021b), exhibited significantly decreased abundance from S1 to S2 and S3 (**Figure 6**). SUS is differentially regulated by ABA and GA (Li et al., 2021b) and can promote storage substance degradation during seed germination (Dong et al., 2015). These results indicate that GAs may promote embryo growth and endosperm weakening in *P. cyrtonema* seeds through ABA-GA antagonism regulation.

## 5 Conclusions

This study utilized iTRAQ-based dynamic proteomics, in conjunction with transcriptomics and hormone profiling, to perform a comprehensive analysis of the seed germination process in *P. cyrtonema*. The integrated proteomic and transcriptomic analyses unveiled alterations in several key KEGG categories, such as signal transduction, energy metabolism, carbohydrate metabolism, and more. Notably, several pivotal proteins, including PYL4, PP2C, SNRK2, and SUS, exhibited decreased abundance between S1 and S3. PYL4 mediated the activity of PP2C and SNRK2, thereby regulating the state of the ABA signaling pathway, while SUS participated in the antagonism between ABA and GA. Conversely, proteins involved in starch and sucrose metabolism, such as INV, α-Xyl, β-D-Xyl, and bglX, showed increased abundance. These proteins facilitated endosperm weakening and the hydrolysis of starch into glucose. Additionally, the levels of ABA, bioactive GAs, IAA, JA, and SA significantly decreased, while the bioactive GAs/ABA ratio increased significantly from S1 to S3. The integrative multilevel omics analysis highlighted that the synergistic interplay between endosperm weakening and hormonal regulation plays crucial roles in the seed germination of *P. cyrtonema*. A model of seed germination driven by endosperm weakening was proposed to elucidate the germination mechanism of *P. cyrtonema* seeds.

## Data Availability

The mass spectrometric and original sequencing data have been submitted to ProteomeXchange (identifier PXD045963) and NCBI’s SRA (accession PRJNA1013739).

## Supplementary material

Supplementary Figure 1 Species distribution of assembled unigenes annotated by NR.

Supplementary Figure 2 Heatmap plot of unigenes during different stages of seed germination.

Supplementary Figure 3 A simplified schematic of the GA metabolic pathway. GGPP, geranylgeranyl diphosphate. CPS, *ent*-copalyl diphosphate synthase. KS, *ent*-kaurenoic acid synthase. KO, *ent*-kaurene oxidase. KAO, *ent*-kaurenoic acid oxidase. GA13ox, GA 13-oxidase. GA20ox, GA 20-oxidase. GA2ox, GA 2-oxidase. GA 3ox, GA 3-oxidase.

## Glossary

DAP: differentially expressed proteins
DEGs: differentially expressed genes
ABA: abscisic acid
GAs: gibberellins
IAA: indole-3-acetic acid
JA: jasmonic acid
CK: cytokinin
NR: Non-redundant
KOG: Eukaryotic Ortholog Groups
COG: Clusters of Orthologous Groups
GO: Gene Ontology
KEGG: Kyoto Encyclopedia of Genes and Genomes
Pfam: Protein family
FPKM: Fragments per Kilobase of transcript per Million fragments mapped
MeJA: methyl jasmonate
SA: salicylic acid
MeSA: methyl salicylate
GA_1_: gibberellin 1
GA_3_: gibberellin 3
GA_4_: gibberellin 4
GA_6_: gibberellin 6
GA_7_: gibberellin 7
GA_8_: gibberellin 8
GA_9_: gibberellin 9
GA_12_-ald: gibberellin 12-ald
GA_15_: gibberellin 15
GA_19_: gibberellin 19
GA_20_: gibberellin 20
GA_24_: gibberellin 24
GA_29_: gibberellin 29
GA_34_: gibberellin 34
GA_44_: gibberellin 44
GA_51_: gibberellin 51
GA_53_: gibberellin 53
CZR: cis-zeatin-riboside
TZR: trans-zeatin-riboside
iP: 6-(γ,γ-dimethylallylamino) purine
iPR: 6-(γ,γ-dimethylallylamino) purine riboside
IBA: 3-indolebutyric acid
TIR1: transport inhibitor response 1 protein
GH3: GH3 auxin-responsive promoter
PYL2: abscisic acid receptor PYL2
SNRK2: serine/threonine-protein kinase SAPK2-like
PYR1: abscisic acid receptor PYR1
PYL4: abscisic acid receptor PYL4
PP2C: protein phosphatase 2C
GRP: gibberellin-regulated protein
Nox: NADH oxidase
NDK: nucleoside diphosphate kinase
2OGD: 2-oxoglutarate-dependent dioxygenase
GID1: gibberellin receptor GID1
ABP: auxin-binding protein
PR: pathogenesis-related protein
RT: Reverse transcriptase
JAZ: jasmonate ZIM domain-containing protein
HAD: 3-hydroxyacyl-CoA dehydrogenase
OPR: 12-oxophytodienoate reductase
GRAS: GRAS domain family
INV: beta-fructofuranosidase
α-Xyl: alpha-xylosidase
β-D-Xyl: beta-D-xylosidase
bglX: beta-glucosidase
SUS: sucrose synthase;
