# Supplementary figures and images for: Dynamic proteomics reveals that endosperm weakening plays a critical role during seed germination in Polygonatum cyrtonema Hua
Source: Front Plant Sci. 2025 Nov 18;16:1662175. doi: 10.3389/fpls.2025.1662175 (PMC12670176; doi:10.3389/fpls.2025.1662175)

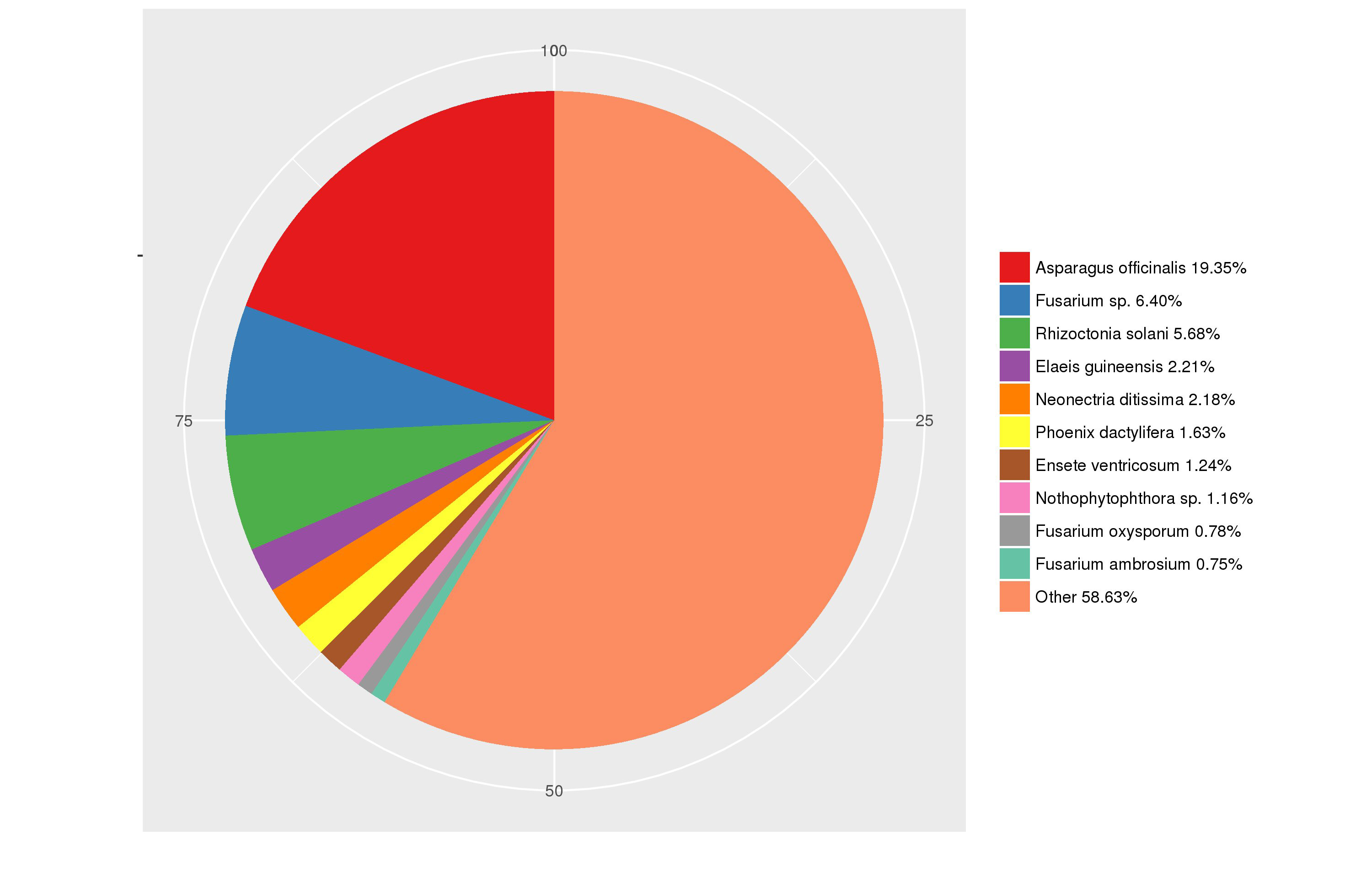

Supplement: Supplementary Figure 1 — Species distribution of assembled unigenes annotated by NR. [file Image1.jpeg]

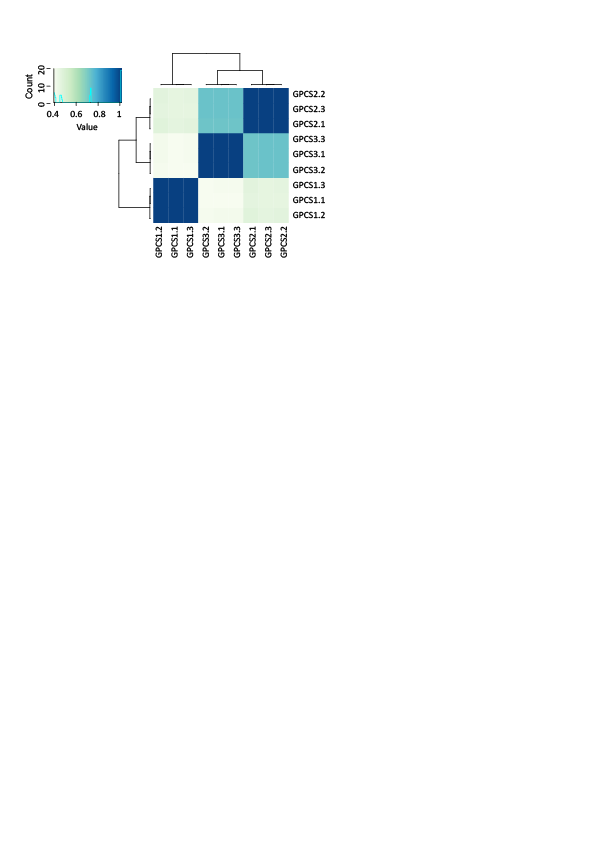

Supplement: Supplementary Figure 2 — Heatmap plot of unigenes during different stages of seed germination. [file Image2.tif]

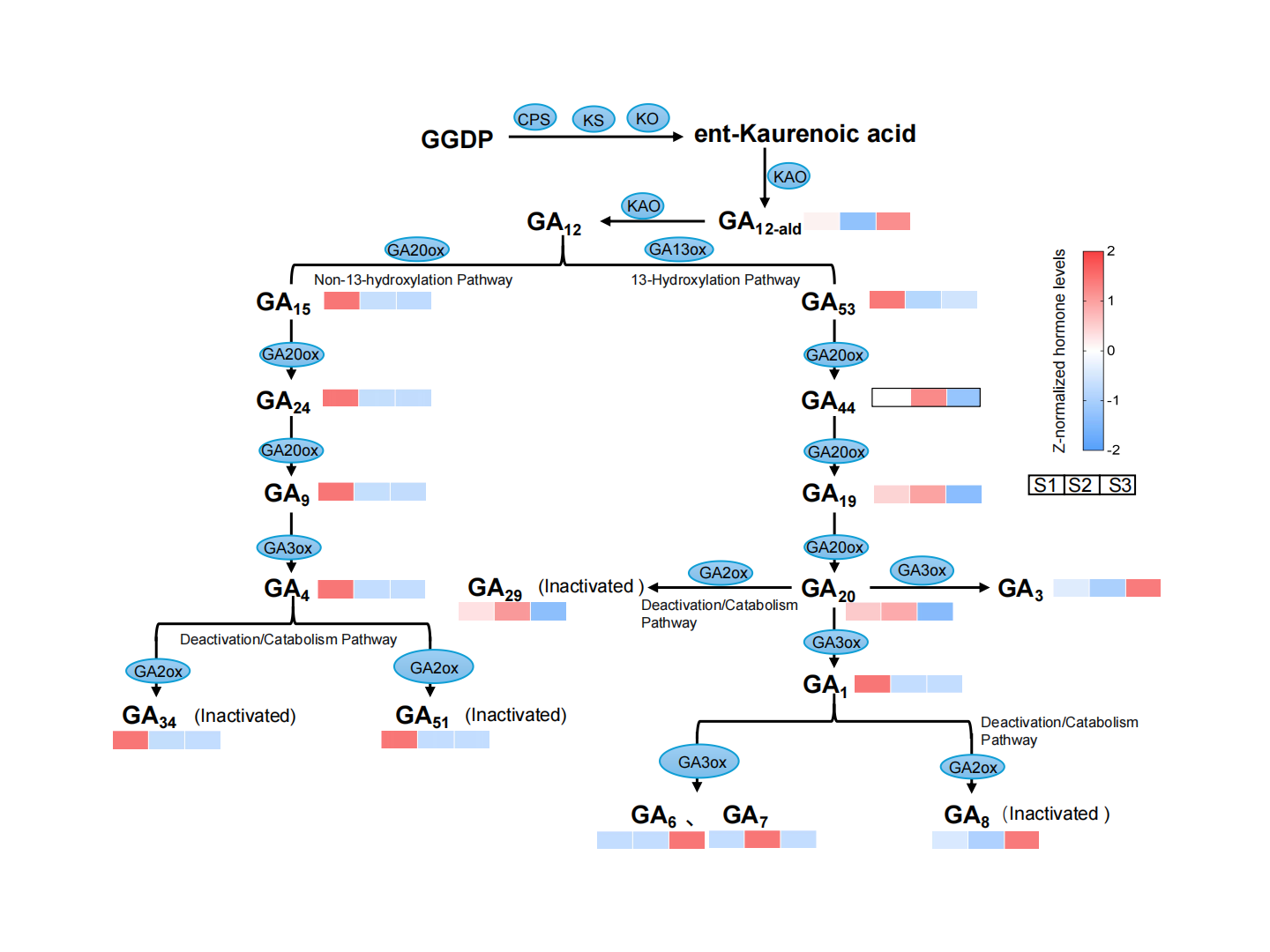

Supplement: Supplementary Figure 3 — A simplified schematic of the GA metabolic pathway. GGPP, geranylgeranyl diphosphate. CPS, ent-copalyl diphosphate synthase. KS, ent-kaurenoic acid synthase. KO, ent-kaurene oxidase. KAO, ent-kaurenoic acid oxidase. GA13ox, GA 13-oxidase. GA20ox, GA 20-oxidase. GA2ox, GA 2-oxidase. GA 3ox, GA 3-oxidase. [file Image3.tif]
