# Supplementary material for: Dynamic proteomics reveals that endosperm weakening plays a critical role during seed germination in Polygonatum cyrtonema Hua
Source: Front Plant Sci. 2025 Nov 18;16:1662175. doi: 10.3389/fpls.2025.1662175 (PMC12670176; doi:10.3389/fpls.2025.1662175)
Supplement: Supplementary file 10 [file DataSheet1.docx]

**Txt S1. The predicting CDS sequences of unigenes**

>TRINITY_DN0_c0_g1|m.3 TRINITY_DN0_c0_g1|g.3 ORF TRINITY_DN0_c0_g1|g.3 TRINITY_DN0_c0_g1|m.3 type:complete len:127 (+) TRINITY_DN0_c0_g1:2591-2971(+)

ATGGAAATAATTACAAAAGTCCAATGCAGTCAGCCTCACATACTGCCTTGTGGGTACAAA

CACCAAAGCAGATTCCCTGTTTTTGGCATTCTGGACTATAGCAGTGTAAGTCGGTTTCGT

CATTGCCCGCATCCTTGCTTCAAAGTTCGATATATCTATGCTCTGGATGTGTATGTCCAA

AGGCACAGGACGAGTTTCTGGAGGAAAATTGAAGAGGCCATGAGAAGTCACCCCAATCCA

TTCCCCAATGTCCTTAGCATTAGCAAGGGAAGCCGAGAGTGCTACAATTCGGGTATTACT

GCCAATTTGACTTGTGATTCTCCTCGCCCTAGAGATTATAATCTCCAAGATTGGACCCAT

TGGTCCACCAATCAGGTGTAG

>TRINITY_DN0_c0_g1|m.1 TRINITY_DN0_c0_g1|g.1 ORF TRINITY_DN0_c0_g1|g.1 TRINITY_DN0_c0_g1|m.1 type:complete len:1144 (-) TRINITY_DN0_c0_g1:908-4339(-)

ATGACAAAAAGACTGGCTGCTTTCAGTCCACTGACCTTGGTAGAATTGCAAGTTATTACT

ATTGAAGAATTCAAGTATATCTCAGTGAGGCAAGATGAGAAGGTGGAACTGGCAAAGCTC

CTGGAGCGGGTGCCTGTTCCTGTCAAGGAAAGCTTGAATGAGCCAAGTGCAAAGATAAAT

GTTCTTCTACAGACATATATATCAAATCTTAAGCTTGAAGGGCTTTCACTTGCATCTGAT

ATGGTTTTCATCAGACAGAGTGCTGGGCGTCTTCTACGAGCACTGTTTGAGATTGTTTTA

AGGAAGGGTTGGGCGCAATTGGCCAAGAAGCTTTTGAATCTTTGCAAGTCAGTGGATAAG

CAAATGTGGGGTATCCAAACTCCGCTGCATCAATTCCATGACATTCCGAATGCAATCTTG

GGGAAACTTGAGAAGAAGGTTTTGGCCTGGGAAAGGTTATATGACCTCTCCCCACAAGAG

ATTGGAGAGCTGATCCACTACCCAAAGATGGGCAGGCTACTGTACAAGCGAATACATCAG

GTGCCTAAAGTTAATCTCACAGCTCATGTTCAGCCTATCTCTCGCACTACCTTGAGCATT

GAGCTGAAGATAATACCTGACTTTCAATGGAATGATAAAGTCCATAAGTGTGCTGAGCCA

TTTTGGATCATTGTTGAGGATAATGATAGCGAATGCATCCTTCACTATGAGTACTTCATA

CTCAAAAAACAGTTTATTGAAGAAGACCACACTTTAAACTTCATAGTTTCCATTTATGAA

CCGCTGCCTCCCCAGTACTTCATTTGGGTGGTGTCTGATCGATGGCTTGGTTCTCAGACA

GTGTTACCTGTTTGTTTTAGACATCTAATCTTGCCAGAAAAGTACCCACCAGCAACTGAG

CTGCTTGATTTGCAGCCTTTACCAATGACTGCACTAAGAAACCCAGAATATGAAGCTCTG

TATGCTACATTCAAGCAGTTCAATCCCATCCAAACACAAGTATTTACTGCTCTATATAAT

ACAGACTACAATGTGTTGGTTGCAGCTCCAACAGGGAGCGGAAAGACCATATGTGCTGAG

TTTGCCTTGTTGCGGAACCACCAAAAGAAGCCTTCAGGCTCCATGAGGGCAATATATATT

GCTCCTATTGATGCACTTGCTGCGGAAAGGTACAGAGAGTGGAAGGAGAAGTTTGAAAAG

AACTTAGGCATTCATGTTGTGAAGTTAACTGGGAATATCACAAGAGATCTGAAGCTTCTT

GAGGAAGGGAAGATCATTATCAGCACACCTGAGAAGTGGGATGTTCTTTCTCGTCGGTGG

AAGCAGTGCAAGCCCATCCAGAAGATCAGTCTCTTCATTGTTGACGAGCTACACCTGATT

GGTGGACCAATGGGTCCAATCTTGGAGATTATAATCTCTAGGGCGAGGAGAATCACAAGT

CAAATTGGCAGTAATACCCGAATTGTAGCACTCTCGGCTTCCCTTGCTAATGCTAAGGAC

ATTGGGGAATGGATTGGGGTGACTTCTCATGGCCTCTTCAATTTTCCTCCAGAAACTCGT

CCTGTGCCTTTGGACATACACATCCAGAGCATAGATATATCGAACTTTGAAGCAAGGATG

CGGGCAATGACGAAACCGACTTACACTGCTATAGTCCAGAATGCCAAAAACAGGGAATCT

GCTTTGGTGTTTGTACCCACAAGGCAGTATGTGAGGCTGACTGCATTGGACTTTTGTAAT

TATTTCCATGCTGAGATGGGGAAAAAGCCTCTATTCCTACTTGGATCTGACACAGAAGTG

GAGGCTGTGCTTCCAGGCATCAAGGAGGAGACTTTAAAGAGGACCCTCCCTCTTGGAGTT

GGCTATTTACATGAAGGGCTTGACGAACATGATCAGAAAATTGTGTTGCAACTGTTTGTC

GATGGAAAGATTCAAGTCTGTGTCGCAAGCAGCTCAATGTGCTGGGGAAAACCAATTCCA

GCTCATCTGGTTGTAGTGATGGGAACCCAGTACTATGATGGGCTCAATAATGCTCATACT

GATTATCCAATAACTAATCTATTACAGATGATGGGCCATGCAAATAGGCCTCTTATTAAC

AGCTCAAACAAATGTGTCGTCCTTTGCCACACTTCTCAAAAGAGTTACTACAAGAAGTTC

CTGTATGAGGCTATTGTTGTCGAGAGCCATCTGCATCACTTCCTCCATGACCACTTGAAT

GCAGAAGTAGTTGCTTGTGTTCTGGGGAGCAAACAGGATGCCGTGGATTATTTCACTTGG

ACTTTCATGTACCGGAGGCTCACCAAGAACCCAAACTACTACAATCTTCATGGTATTAGC

CATCGACATCTCTCTGACTATCTCTCAGAATTAGTGGAGAATACATTGACCGATTTGGAA

GCAAGCAAGTGTGTCTGCATATCTGAGGATATGTATGTCGAGGCTAGTAAGCTTGGTTTG

ATTGCTTCATATCACTACATTAGTTACACCACTATTGAACGTTTCAATTCATCTCTAACT

CAGAAAGCCAAGAAAACGGACCTTCTCGATATTTTGGCATCGTCTTCGGAGTATGCTGAG

CTCCCCATTCGACCCGGAGAGCAGGAGCTGCTTAAAACATTGATAAATCATCAGAGATTC

TCCTTCAAGAACCCAAAATACACAGACCCACACGTGAAAGCCAAAGCTCTGTTACAAGCA

CGTATCTCGAGGCAGAAATTGGTCGGGAACCTAGCACATGACCAGCAACAGGTGCTATGG

TCTGCCTACAGATTGATTCACGGTATGGTGGATGTGATCTCTAGCAATGGCTGGCTCAGC

CTTGCCCTTTCTGCAATGGAGCTGAGCCAGATGTTGACACAAGGCATGTGGATTACTGAT

TCAGTGGCTTTACAACTTCCACACTTCACCAATGAGATGGTCAATAGATGTGAAGTGAAC

CTAGGAAGAAGTATGGCCACAGTCTTTGATCTATTGGACATTGAGGAAAATGACAGGACC

ACATTGCTACAGATGCCCGGTCCCCAATTGCTAGAAATTGCTGAGTTCATCAGCAGATTG

AATATTGATATGAGTTATGAAGTGATCAATGGCGATATTGTTAGGCCTGGGGAGAATGTT

ACTCTGCAGGTTATGTTAGAACGCGATCTTGTAATGCTGGAGGAGATGGGACAGGCTAAT

GCTAAAGATGGCTGGTGGCTTGTGGTGGGTGATACTGGGATCGATGAGTTGTTTGCTATC

AAGAGGGTTTCGTTTCAGAATACACGCAAAGTGAAGCTCGACTTCCCTGCCCCTTCAGAA

GTTGGGAAGAAGGCTTACAATGTTTACTTCATGTCTGATTCATATGTGGGCTGCGATCAG

GAGTATGTGTTCACCCTGGATGTCAGGGATAACAGGGAAGATGATGGAGGTATGGTCGAG

GGGATGTGTTAA

>TRINITY_DN0_c0_g1|m.2 TRINITY_DN0_c0_g1|g.2 ORF TRINITY_DN0_c0_g1|g.2 TRINITY_DN0_c0_g1|m.2 type:complete len:1019 (-) TRINITY_DN0_c0_g1:4275-7331(-)

ATGTCTTATCTGGAAGGAGGTGCAGAAACCCATGCACGGTCCAAGCAGTACGAGTACCAT

GCAAACTCAAGCCTTGTATTGGCTGAAGATTTGGACCGTCAAGGTAACCATGAGCCCACT

GGCAAGCCTCAGTCCCTGCAGGGAAAGATTTACCTTAAACACTTTGGTGATAGGGCACGG

CATGAAAGCTCCATACTTGACGAGAAGCTCCAAACATTCAAGAAGAAGAAAGAGCGAGAG

CTTGTGGAACCTGTTCCAGACCAAACATTACACCGAAAGCGGAGACGATTCCAGGAGGAG

AGCGTCCTCTTTCTTGCTGATGATGGTGGCTACCGGCCCAAGACCATGGTGACATGTGCC

GCGTATGAGACATTGCTTAACATGGTACAACAGCAGATTGGCGGACAGCCTCAGGATATT

CTGCATGGAGCTGCAAGCAAGGTGTTATCAATTTTAAAGAATGAGAAGAGCGAGAATACT

GACATGAAGAATGAAATAGAGAAGTTCTTGAATCCGATATCAAATCAGCTCTTTGATCAA

CTTGTATCAATTGGAAGGCTTATCAAAGATTTTCAAGCTGAAGGCGAGATTATGGTCTCT

GCTAATGCTAATACCGAGGCACTTGATGATGAATTTGGTGTTGCTGTTGAGTTTAAAGAG

GACGAGGAGGAAGAAGAGAGCATTTATGATCAGATCCTTGAAGAATTAGATGATGATGAA

GATGGTGATCAGGAATTCAATGGTATTCGGGCTATTCAAATGGATGGGACAGAAGAAGAT

GAATTGGAAGAAGCTATTGAAGAGCTGACGTTAAATGTTCAAGACATTGATGCCTACTGG

CTCCAGAGGAAAATTTCTCAATCATATAAAGAGATTGACCCGAAGGACAGCCATAAGCTT

GCAGAGGATGTTCTTAAGATATTAGCAGAAGGGAATGATATTGATTTGGAGAATCGTCTC

GTCATGCTTATGAACTATGATAAATTTGTTCTCATCAAATTGCTTCTAAGAAACCGGCTA

AAGATAACATGGTGTACAAGGTTGGCAAGAGCAGAAGACCCAGAGCAGCGGAAAAGGATT

GAGGAAGAGATGAGAATGGCGGGTCCAAGCCTAGTTACAATTCTGGAAGAGTTGCAAGTT

ACAAGGGTGTCAGCAAAAGAGCACCAAAAGTACATGGGGAAGAGCTTACAAGAGGCTAGG

AGATTAAATAATGAAGGTGGTGGCAAGGATGGGAAGAGAGTTTCAGATCAGATGATTTTC

AGTAGAGAGGTGAAGAATGAGCAATTGAAGGGGCCACATCAGCTACTTAATCTTGATAGC

ATTGCATTCCATCAAGGTGACCTTTTAATTTCAAACATAAGATGCGATCTTCCTCAAGGA

TGTTATAGAACTCTGCACAAGGGTTATGATGAAGTTTATGTGCCAGCTCCAAAGCCAAAA

GAATTCACTCCTGAAGAGGAGCCTATAAAGATATCTAATATGCCAGATTGGGCTCAAAAG

GCTTTTGAAGGGTTAGAACATCTAAATAGAGTGCAGAGTAAAGTTTATAAGGCTGCCCTT

TTTACATCCGAGAACATACTTTTATGTGCTCCTACAGGTGCAGGAAAAACAAATGTTGCT

GTGCTGACTATACTTCATCAGATTGGTTTGCATAGAAAGGATGGAGTATTAGACAACACG

AAGTATAAAATTGTTTATGTGGCACCAATGAAGGCATTGGTGGCTGAGGTGGTCAGAAAC

TTGTCTAACCGCCTGGAGTCTTATGGGATTGTTGTGAGGGAACTTAGTGGCGATAATTCG

CTTACTGGCCTACAGTTTGAAGAGAGCCAGATAATTGTTACAACACCAGAGAAGTGGGAC

ATTGTCACAAGAAAATCTGGGGATAGAGTATATACTAATATGGTTAAGCTTATTATCATT

GATGAGATCCATATGCTTCATGATAATAGGGGACCTGTTCTTGAAAGCATCATTACGAGG

ACATTTAGACAAATTGAGAACAGCAAGGAGCTTGTTCGGTTTGTTGGGTTGTCAGCTACT

CTCCCAAACTACAAGGATGTCGCACAATTTTTACGTGTTAATCCATCTGAAGGTCTCTTT

CATTTTGACAATAGTTACAGGCCCTGCCCACTGGCACAACGTTATATTGGAATCACAGCT

AAGCAACCTCTGCAGAGATTTCAGTTGATAAATGAAATCTGTTATGAAAAGGTTGTGGCT

GCTGCAGGAAAGCATCAAGTACTCATATTTGTACATTCTAGGAAAGAAACTACCAAAACA

GCCCGTGCTATTAGAGATGTTGCACTGGCTAATAACAAACTAAGTAGGTTTCTGAAGGAT

GATTGTGCCAGCCATGAGATTCTTCAGAGTCTAACAGGATCAGTCCAGAGCAATGATCTC

AAGGACCTCTTACCATATGGCTTTGCAATCCATCATGCAGGGATGTCAAGATTGGACCGA

GATCTCGTTGAGGAGTATTTTACCAATAAGCATATACAGGTGTTGGTCTCAACAGCCACT

CTTGCTTGGGGTGTCAATTTGCCAGCGCACACTGTGATTATTAAGGGGACCCAGATGTAT

AATCCTGAGAGAGGAGCGTGGACTGAGCTGAATTCTTTGGATGTAATGCAGATGATTGGT

AGGGCAGGTAGGCCTCAGCATGACTCTTACGGAGAAGGAATAATCTTAACGGGGCACAAT

GAGTTGATGTACTATCTATCCCTCATGAATCAGCAACTGCCTATTGAGAGCCAATTTGTG

TCAAAATTAGCAGATCATCTGAATGCTGAGATTGTGTTAGGAACTGTTCAGGATGTTCGG

GAAGCTTGTATCTGGATTGGGTACACTTATTTATATATCAGAATGCGAAGGAACCCAAGT

CTTTATGGTCTACCAGCTGATATTCTGGACAGAGACGAGACATTGGATGGAAGGAGAGCT

GATCTGATCCACTCTGCTGCTACTGTTCTGGAAAAGAATAATTTGATCAAGTATGACAAA

AAGACTGGCTGCTTTCAGTCCACTGACCTTGGTAGAATTGCAAGTTATTACTATTGA

>TRINITY_DN0_c1_g1|m.6 TRINITY_DN0_c1_g1|g.6 ORF TRINITY_DN0_c1_g1|g.6 TRINITY_DN0_c1_g1|m.6 type:complete len:718 (+) TRINITY_DN0_c1_g1:122-2275(+)

ATGGCTCCCAGCTCCGAACCCCAATTCGTTGTCTACTCCCCCGTCAAAATGCCCAACAAA

TCCCCGCCCCCTAACCCCTTCTCCCATCTTCTCCCCTTTGCCCACCAAAACCTCTCCTTC

CACCTCCAACGAGCTCACGAGTCCATCAAAACCCTAGCCAGCTCGCTGCAATCGGGTCCA

GTCCCGAAACCCGCAAACCCTAGCAAAACCCTAGAAGCCCCTCGCAATCCCGACGCCCAA

TTCGTCCAATCCTCCATCAAAATCCCCAGACCCCAATTCCCTCGCCCATTCCCCTCGTCC

CTCCCCTTTGCGCACCAAAACCTTTCCTTTCACCTGCAGAGGGCCAGAGATTCCGTCGTC

AACCTCTTCAGGCGCGGGCCCCACCCAATCCTGTGCTCGTCGACGCTGGCCGTGGCGAGG

AAGAGAGAGGACGACCAGGAGAGGGTTCTGATTAGTGAGGTGTTGATAAGGAACAAGGAT

GGGGAGGTGATGGAGAGGAGGGACCTCGAGGAGGTTGCGGCCTCGGCGATCAAGGCTTGC

CGGCCCAATTCGGCGCTGACGGTGAGGGAGGTCCAGGAGGATGTGCACCGCATCGTGGAG

AGCGGCCTGTTCTCGTCGTGCATGCCGGTGGCGGTCGACACGAGGGATGGGATCAGATTA

GTCTTTCAGGTAGAACCCAACCAAGAGTTCCAAGGGTTGATCTGTGAGGGAGCAAGTGTT

CTTCCTGCTAAGTTTTTGGAAGACGCATTTCGTGATGGATATGGAAAAATAGTCAATATA

AGGCATTTGGATGAGGTGATCAAATCTATCAATGGGTGGTATACAGACCGAGGGCTATTT

GGTCTGGTTTCAGGCATTGAGATCCTTTCTGGAGGAATCCTTAGGCTGCAAGTCTCTGAA

GCTGAGGTTAATAACATAACAATACGTTTTCTTGATAGAAGAACTGGGGAACCTACTGTG

GGAAAGACAAAACCGGAGACTATACTTAGACAACTCACAACAAAGAAGGGACAGGTTTAC

AGTTTGCTACAAGGTAAAAGAGATGTGGAAACAATTTTAACAATGGGTATCATGGAAGAT

GTGACGATCATTCCCCAACCTGCTGGAGATACTGGTAAGGTGGATCTTGTGATGAATCTT

GTTGAACGCCCTAGTGGTGGTTTCTCTGCTGGTGGTGGTATTTCCAGCGGGATAACAAAT

GGCCCTCTCTCTGGACTCATTGGAAGCTTTGCATATTCTCATAGGAATGTTTTTGGGAGA

AATCAAAAACTTAATCTTTCGCTAGAAAGGGGCCAGATTGATTCCATATTTCGAATAAAT

TATACAGATCCATGGATAGAAGGAGACAATAAAAGGACATCTAGATCAATTATGATTCAG

AATTCAAGGACTCCAGGAACACTTGTCCATGGAAATCAACGTGGTGGTGTAACAATTGGG

CGAGTTTCAGGTGGAATTGAATTCAGTCGACCATTAAGGCCCAAGTGGAGTGGTACAGCT

GGGCTCATATTTCAGCATGCTGGTGCCCGTGATGATAGTGGAGCTCCCATCAATAGAGAT

GTCTACAACTGCCCATTAACTGCAAGTGGCAATACTTATGATGACATGCTAATTGGCAAA

CTTGAAAGCGTATATACAGGTTCTGGCGATCATGGGTCCTCAATGTTTGCCTTCAACATG

GAGCAAGGACTGCCTGTTCTTCCTGAGTGGCTCCGTTTCAACAGAGCAACTGCTCGTGCC

CGACAGGGTGTAGAAATAGGTCCTGCTCGAGTACTTTTAAGCTTTTCTGGTGGTCATGTA

GCCGGAAGTTTTCCTCCTCATGAAGCATTTGCAATTGGTGGGACCAACAGCGTTAGAGGT

TATGAGGAAGGTGCTGTGGGTTCTGGACGTTCATATGCTGTTGGCAGCGGAGAACTTTCA

TTTCCCCTGTACGGGCTACTAGAAGGCGTGCTATTTGCTGATTATGGAAGTGATCTTGGC

TCAGGCCCATCCGTACCTGGTGATCCTGCTGGAGCTCGGGGCAAGCCAGGCACTGGTTAT

GGATATGGGTTAGGAATACGCATAGACTCACCATTGGGACCTCTCCGTCTAGAATATGCT

TTGAATGACAGACATGCCAGAAGGTTTCACTTTGGAGTTGGCCTACGCAACTAA

>TRINITY_DN10001_c0_g1|m.7 TRINITY_DN10001_c0_g1|g.7 ORF TRINITY_DN10001_c0_g1|g.7 TRINITY_DN10001_c0_g1|m.7 type:internal len:145 (-) TRINITY_DN10001_c0_g1:2-433(-)

GCGTTCCTCTCCCAGTACGGGATCGCCAAGGAGACCGATACCGTCAAGAAGTACAATACC

AACGCCGCCTCCGTGTACCGTGACAGGATCCAGGCCTTGGCCGAAGGCCGGCCGTGGAGA

GACCCGCCGGTAGTTAAGGAGAGCCTCGGCGGTGGGGCCCAGTCGAGGAAGCCGCCGACT

GGACAGAATGGTGCGAGTTCATTGAAGAGTAATGGCGGGTGGGATGATTGGGATGATGAT

AACCGATCATCCTCGGATATGAGGAGGAATCAGTCGGCCGGAGATTTCAGGTCGGGGAAT

GGTGGTGGGATGAGAGGAGGACATCCCTCTAGGTCGCGATCGCAGGAGGATATTTATACG

AGATCGCAGCTGGAGGCCTCGGCGGCTAATAAGGAGAGTTTCTTTGCGAGAAAGATGCAG

GAGAATGAGTCG

>TRINITY_DN100069_c0_g1|m.10 TRINITY_DN100069_c0_g1|g.10 ORF TRINITY_DN100069_c0_g1|g.10 TRINITY_DN100069_c0_g1|m.10 type:internal len:103 (+) TRINITY_DN100069_c0_g1:2-307(+)

GGCAGCTTGTTGCGGATCGCCACATGCAGCGGCAGCATGCCCGACAGGTTCTCGATGCGG

CAGGCGCCCGGGAAGGCGTCCAGCAGCTGCTTGACGACCGCGAGCGGCACCGAGGGCTCG

GTGCACGCCCAGTGGAGCGGGAGCATGCCGAAGTCGTCCTGCGCCTTGGCGAGCGCGCGC

TGGTCCGCGAGCAGCGGCAGCGCCTCCATCCAGTCGCCGGCCTCGGCCAGCGCCAGCACA

CGCAGCCTGTCGGCGTCCATGGCGCGGTCTCCGGCCGATCGAGAGACAGAGAGACGAGAG

GTATAC

>TRINITY_DN100069_c0_g1|m.11 TRINITY_DN100069_c0_g1|g.11 ORF TRINITY_DN100069_c0_g1|g.11 TRINITY_DN100069_c0_g1|m.11 type:internal len:103 (-) TRINITY_DN100069_c0_g1:1-306(-)

TATACCTCTCGTCTCTCTGTCTCTCGATCGGCCGGAGACCGCGCCATGGACGCCGACAGG

CTGCGTGTGCTGGCGCTGGCCGAGGCCGGCGACTGGATGGAGGCGCTGCCGCTGCTCGCG

GACCAGCGCGCGCTCGCCAAGGCGCAGGACGACTTCGGCATGCTCCCGCTCCACTGGGCG

TGCACCGAGCCCTCGGTGCCGCTCGCGGTCGTCAAGCAGCTGCTGGACGCCTTCCCGGGC

GCCTGCCGCATCGAGAACCTGTCGGGCATGCTGCCGCTGCATGTGGCGATCCGCAACAAG

CTGCCG

>TRINITY_DN10007_c1_g1|m.13 TRINITY_DN10007_c1_g1|g.13 ORF TRINITY_DN10007_c1_g1|g.13 TRINITY_DN10007_c1_g1|m.13 type:5prime_partial len:137 (-) TRINITY_DN10007_c1_g1:230-640(-)

AACAACTTCCCTTTTCAAGAACTAAAACACACACTTCACATGCAGAACGCGAAGGACCTT

GCTCTTTTCGATGCCCTTGGCGGAGAAGCCGCCATCGACGCCGCTGTTGACATCTTCTAC

GGAAAGGTGCTCGCCGACGACCGCGTCAAGCACTTCTTCGACAAGACCGATATGGAGAAG

CAGAGAAACCACCAAAAGAGATTCTTGACTGTGGCCCTCGGAGGACCCAACAGATACACT

GGAAGAACCATGAGAGGCGCTCACAAGGGTATGGGCCTCACCGACGAGCACTTCAACGCT

ATTGGCGAAAACTTGGCTGCCACCCTGAAGGAACTTAAGGTCGCCGATGAAATGATCGAG

AAGGTTTTGGCTGTTGTCGAGACGACCAGAACTGACGTTCTTGACAGATAA

>TRINITY_DN10008_c0_g1|m.14 TRINITY_DN10008_c0_g1|g.14 ORF TRINITY_DN10008_c0_g1|g.14 TRINITY_DN10008_c0_g1|m.14 type:3prime_partial len:129 (+) TRINITY_DN10008_c0_g1:130-513(+)

ATGCCGCGGTTCCACGTCGAGGGCAAGGTGGTCGAGGGCGTCGACCTCTTCAGGCGGAGG

CATTGGGCGTGGCGAGTCGACGTCTGGCCCTTCGCTCTCCTCTACTCGGTCTGGCTCTTC

GCCGTGCTTCCCAGCCTCGATTTCACCGACGCCTGCATCGTTCTCGGGGGAATCTCGGTG

TTTCATATTCTCGTGTTGCTGTTCACCGCTTGGTCCGTCGATTTCCGGTGTTTTGTTCAG

TTTAGCAAGGCTAAAGACATCAATTCTGCGAATGCTTGCAAAATTATGCCGGCTAAGTTT

GCGGGTTCGAAAGAAATCGTGCCACTTCATTTTAGGAGCCATGTTACAGGAACACCAGCA

TCTTCATTAAGGAATGTTGATGAT

>TRINITY_DN10008_c0_g1|m.15 TRINITY_DN10008_c0_g1|g.15 ORF TRINITY_DN10008_c0_g1|g.15 TRINITY_DN10008_c0_g1|m.15 type:complete len:127 (-) TRINITY_DN10008_c0_g1:91-471(-)

ATGGCTCCTAAAATGAAGTGGCACGATTTCTTTCGAACCCGCAAACTTAGCCGGCATAAT

TTTGCAAGCATTCGCAGAATTGATGTCTTTAGCCTTGCTAAACTGAACAAAACACCGGAA

ATCGACGGACCAAGCGGTGAACAGCAACACGAGAATATGAAACACCGAGATTCCCCCGAG

AACGATGCAGGCGTCGGTGAAATCGAGGCTGGGAAGCACGGCGAAGAGCCAGACCGAGTA

GAGGAGAGCGAAGGGCCAGACGTCGACTCGCCACGCCCAATGCCTCCGCCTGAAGAGGTC

GACGCCCTCGACCACCTTGCCCTCGACGTGGAACCGCGGCATGATTCAATGGTCAAAACC

CTAGGGGTGGTTGTGATCTAG

>TRINITY_DN100092_c0_g1|m.17 TRINITY_DN100092_c0_g1|g.17 ORF TRINITY_DN100092_c0_g1|g.17 TRINITY_DN100092_c0_g1|m.17 type:complete len:318 (+) TRINITY_DN100092_c0_g1:77-1030(+)

ATGGCTGCCATCCGTACCGACCTTCCCGGCCCCATTGGGGACAAGAAGCTGGAGAAGAAG

CCCATCAAGTTCTCCAACTTGCTGCTGGGCGCCGGCCTCAACATGTTCGAGGTGACCACC

CTGGGTCAGCCTCTCGAGGTCGTCAAGACTACCATGGCTGCCAACCGAGGTGACGGCATG

GCCAAGGCTTTGGGACGCGTCTGGTCCCGCGGTGGTCCTCTGGGCTTCTACCAAGGTCTC

ATCCCCTGGGCCTGGATCGAAGCTTCCACCAAGGGCGCCGTCCTTCTCTTCGTCGCCTCC

GAGGCCGAGTACTACGCCCGCGTCGCTGGCGCCTCCGAGTTTGGCGGTGGCATCCTCGGT

GGCATCACCGGTGGTGTCGCTCAGGCCTATGCCACCATGGGCTTCTGCACCTGCATGAAG

ACGGTCGAGATTACCAAGCACAAGATCGCCGCCACCGGTGTCAAGCCCCCCTCCACCTGG

CAGACCTTTGGCGACATCTACCGCAAGGAGGGTATCCGTGGCATCAACAAGGGTGTCAAC

GCCGTCGCCATCCGCCAGATGACCAACTGGGGTAGCCGCTTCGGTCTGAGCCGTCTCGCC

GAGGGTTGGATTCGCTCCGCCACTGGCAAGAAGGAGGGTGAGAAGCTCTCTGCTGGCGAA

AAGGTCATTGCCAGCGCCGTCGGTGGTGGTCTCAGCGCCTGGAACCAGCCCATCGAGGTC

ATCCGCGTTGAGATGCAGAGCAAGAAGGAGGACCCCAACCGTCCCAAGAAGATGACGGTT

GGCAACACTTTCAAGTACATCTACGAGACCAACGGTGTCCGCGGTCTCTACCGTGGTATT

ACCCCCCGTATCTCTCTGGGTATCTGGCAGACGGTCTGCATGGTCGCGTTTGGTGACATG

GCCAAGGCTTATGTCGAGAAGCTGACCGGCGATGCGGTTACTGCTAAGCATTAG

>TRINITY_DN100092_c0_g1|m.16 TRINITY_DN100092_c0_g1|g.16 ORF TRINITY_DN100092_c0_g1|g.16 TRINITY_DN100092_c0_g1|m.16 type:3prime_partial len:343 (-) TRINITY_DN100092_c0_g1:2-1027(-)

ATGCTTAGCAGTAACCGCATCGCCGGTCAGCTTCTCGACATAAGCCTTGGCCATGTCACC

AAACGCGACCATGCAGACCGTCTGCCAGATACCCAGAGAGATACGGGGGGTAATACCACG

GTAGAGACCGCGGACACCGTTGGTCTCGTAGATGTACTTGAAAGTGTTGCCAACCGTCAT

CTTCTTGGGACGGTTGGGGTCCTCCTTCTTGCTCTGCATCTCAACGCGGATGACCTCGAT

GGGCTGGTTCCAGGCGCTGAGACCACCACCGACGGCGCTGGCAATGACCTTTTCGCCAGC

AGAGAGCTTCTCACCCTCCTTCTTGCCAGTGGCGGAGCGAATCCAACCCTCGGCGAGACG

GCTCAGACCGAAGCGGCTACCCCAGTTGGTCATCTGGCGGATGGCGACGGCGTTGACACC

CTTGTTGATGCCACGGATACCCTCCTTGCGGTAGATGTCGCCAAAGGTCTGCCAGGTGGA

GGGGGGCTTGACACCGGTGGCGGCGATCTTGTGCTTGGTAATCTCGACCGTCTTCATGCA

GGTGCAGAAGCCCATGGTGGCATAGGCCTGAGCGACACCACCGGTGATGCCACCGAGGAT

GCCACCGCCAAACTCGGAGGCGCCAGCGACGCGGGCGTAGTACTCGGCCTCGGAGGCGAC

GAAGAGAAGGACGGCGCCCTTGGTGGAAGCTTCGATCCAGGCCCAGGGGATGAGACCTTG

GTAGAAGCCCAGAGGACCACCGCGGGACCAGACGCGTCCCAAAGCCTTGGCCATGCCGTC

ACCTCGGTTGGCAGCCATGGTAGTCTTGACGACCTCGAGAGGCTGACCCAGGGTGGTCAC

CTCGAACATGTTGAGGCCGGCGCCCAGCAGCAAGTTGGAGAACTTGATGGGCTTCTTCTC

CAGCTTCTTGTCCCCAATGGGGCCGGGAAGGTCGGTACGGATGGCAGCCATGACTTTGGG

ATGTGTCGCAGAATGAGGGCCCTGACCAAGGGAAAAGGGAGCTTCTTCGCAAAAAAAAAG

GTGTTA

>TRINITY_DN1000_c0_g1|m.19 TRINITY_DN1000_c0_g1|g.19 ORF TRINITY_DN1000_c0_g1|g.19 TRINITY_DN1000_c0_g1|m.19 type:complete len:589 (+) TRINITY_DN1000_c0_g1:290-2056(+)

ATGCCGGGCCTCGTGACTCCGGCCGAAGCTCCGCCGCTCGAAATCACTGTCCCTGACTCG

AATCCCCAGATCCGGCCCCAATCCCAACAGTCCACCCCCCAAAACCCGAGGAATCGTTCG

TCCCCGGTCCCAAGGAAGACTCCTTCCCCTTCTTCGTCCTCCACATTCAAGAAGCGGAGC

TCGCTGTCGACGCCGGCGTTCGATGAATCGGCGCTCGACAACCCCGATCTCGGCCCGTTC

CTCCTCAAGCTCGCCCGCGACACGATCGCCTCCGGCGAGAGCCCCGTCAAGGCCTTGGAG

TATGCGATACGAGCTTCCAAGTCGTTCGAGAGGTTCGAGGAGATGGGCTTGGATCTGGCG

ATGAGCCTGCACGTGACGGCGGCTATCTACTGCAGTTTGGGGAGGTACGAGGAGGCGGTG

CCGGTTCTTGAACGGGCGGTGGCGGTGCCGGAGGCGGAGAGAGGGCCGGAGCATGCGCTG

GCCAAGTTTTCGGGGTATATGCAGCTTGGAGATACGTATTCGATGCTCGGGAGAGTGGAT

CAGAGTATTGAGTGCTATACCAAAGGGCTTCAGATCCAGATGGAGGCTCTAGGGGAGATG

GATCCCAGAGTTGCCGAGACTTGCAGGTACTTGGCGGAAGCTCATGTCCAGGCAATGCAG

TTTGATGAGGCTGAAAAGCTCTGCAAGAAAACCCTTGATATTCATCGAGAGCACAGTGCT

CCTGCTTCTATCGAGGAAGCAGCAGATCGCCGATTAATGGCCCTCATTGCAGAGGCCAAA

GGAGACTTTGAATCTGCCCTTGAACACCTTGTGCTTGCGTCTATGGCAATGATTGCCAAT

GGCCAAGAAACTGAGGTCGCCTCTATTGATGTCAGCATTGGAAACACCTACCTCTCACTT

GGTCGTTTTGATGAAGCCATCTTCTCTTACCAGAAAGCCCTCACAGTTTTCAAATCTACC

AAAGGTGATAATCACCCATCAGTTGCTTTAGTCTTTGTTCGACTAGCTGAACTTTATTAT

AAGACAGGCAAGCTCCGGGAATCAAGATCCTATTGTGAGAATGCCCTTAGAATCTATGCA

AAGCCTGTTCCAGGGACAACACCAGAGGATATTGCTGGTGGTTTGATGGAGATTTCTGGT

ATTTATGAAGCCATGGAGGAGCCTGATGAGGCGTTGAAGCTATTGCAGAAAGCTTTGAAG

CTGTTGGAGGATGTGCCGGGGCAGTGGAGTACGGTTGCTGGGATTGAAGCTCAAATGGGA

GTGATCTATTACATGGTAGGGAAGTATGGAGAGTCTAGGAATTCTTTTGAAAGTGCTGTG

GCTAAGCTGAGAGCAAGTGGCGAGAGAAAGTCGGCATTCTTTGGAGTGGTGTTGAACCAG

ATGGGGTTGGCATGTGTGCAGTTGTTTAAAATTGATGAAGCAGCTGAGCTGTTTGAAGAA

GCAAGGGGGATCCTGGAACAGGAATATGGGGCTTGTCATCAGGACACGCTTGGTGTATAT

AGCAATCTAGCTGCAACTTATGACGCCATGGGAAGGGTAGAAGATGCAATTGAAATCTTG

GAATATGTTCTCAAGATCAGAGAGGAAAAGCTCGGGACAGCAAACCCTGATGTAGACGAG

GAGAAGAGATGGCTAGCTGAACTCTTAAAAGAAGCTGGCCGGGCCAGGAAAAAGAACGCA

AAATCCCTGGAAAACCTTATTGTCAACACATCGCAGAGGATGAAGGAGGTTACAAGGAGG

TGGTCAGGCCTGGGGTTCAAAACTTAA

>TRINITY_DN10010_c0_g1|m.22 TRINITY_DN10010_c0_g1|g.22 ORF TRINITY_DN10010_c0_g1|g.22 TRINITY_DN10010_c0_g1|m.22 type:5prime_partial len:734 (-) TRINITY_DN10010_c0_g1:462-2663(-)

AAATTCTCAGTCTCACCACTAAAAAAAAACCCCAATCATCCTCTTCCCCCCTTTGTCTTG

AATCAAAATCCCCAAATTTTTTCTAGGGTTTCATCGATCTGCGCCAGCGTCGAGGCCATG

GCGTCCTCAATCACGGGCCAAACCCCCGCGTTGAAGAGACGGGAGTCGACGGCGACGAGA

GAAGGGGATCAACTCACCATCACTCCGCTGGGGGCGGGAAACGAAGTGGGTCGATCGTGC

GTCTTCATGTCCTATAGAGGAAAGACCGTCATGTTTGATTGTGGGATTCATCCGGCGTAT

TCGGGGATGGCAGCATTGCCGTATTTTGACGAGATCGATCCATCCACCATTGATGTCCTT

CTGATTACTCATTTTCATCTTGATCATGCTGCTTCACTACCTTACTTCTTAGAAAAGACA

AATTTCAAAGGTCGTGTTTTCATGACCCATGCAACAAAGGCGATTTATCGGCTGCTTTTA

TCTGATTATGTTAAAGTTAGTAAAGTTTCTGTTGAGGATATGTTGTATGATGAGAAAGAC

ATAGTTCGCTCCATGGATAAAATTGAGGTCATTGACTTCCACCAGACACTAGAAGTGAAT

GGAATACGCTTCTGGTGCTATACTGCTGGTCATGTCTTGGGTGCTGCAATGTTCATGGTT

GATATTGCTGGTGTCCGGGTTCTGTATACCGGTGATTACTCAAGGGAGGAGGATCGCCAC

CTTCGTGCTGCTGAAACTCCGCAGTTTTCCCCTGACATCTGTATCATTGAGTCCACATAT

GGTGTCCAGACCCACCAGCCCCGACACATTCGTGAGAAGCGGTTCACTGACGTCATCCAC

TCGACAGTGAGCCAAGGTGGGCGAGTGCTCATCCCAGCCTTTGCCCTTGGCCGAGCACAG

GAGCTCCTTCTTATCCTTGATGAGTATTGGTCCAACCACCCTGAACTTCATAACATTCCT

ATATACTATGCTTCGCCTCTGGCTAAGAGATGCATGGCAGTATATCAAACCTACATTAAT

GCCATGAATGAGAGGATCCGAAACCAGTTTGCTAATTCGAATCCTTTCGATTTCAAACAT

ATTTCCCCACTGAAAAGCATTGAGAATTTTCAAGATGTGGGTCCATCAGTGGTAATGGCA

AGCCCTGGTGGACTCCAGAGTGGACTTTCAAGGCAGCTGTTTGATAAGTGGTGCGCAGAT

AAGAAGAATGCCTGTGTGATTCCAGGGTATGTTGTGGAAGGAACACTGGCTAAGACTATA

ATTAATGAGCCGAAAGAGGTAACTCTCATGAACGGTTTAACCGCTGCCCTTAACATGCAG

GTTCACTACATCTCCTTCTCTGCTCACGCAGATTCTGATCAGACTAGTGCATTTTTAGAG

GAGCTCATGCCTCCCAATATAATCCTTGTCCACGGAGAAGCCAATGAAATGGGCCGGCTG

AAGCAGAAGCTTACTACTCAGTTTGCAGATCGCAACACAAAGATCCTTTCTCCCAAGAAT

TGTCAATCGGTCGAGATGTACTTCAGTTCCGAGAAAATGGCCAAAACAATAGGAAGGCTT

GCTGAGAGAACACCGGAAGTTGGAGAAACTGTCAGTGGGCTACTGGTCAAAAAAGGCTTC

ACATATCAGATTATGGCTCCTGATGACCTCCATGTATTCTCGCAGCTATCAACTGCAAAC

ATCACCCAGCGCATCTCTATCCCTTATACTGGTTCTTTTGGAGTTATCAGGCACAGGTTG

AAGCAGATATATGAGAGTGTAGAGTCTCCACAGGAGGAGACAGAAGTTCCGACTATTATT

GTTCATGAGAAGGTTACCATAAGGCATGAGTCAGAGAAGTATGTGACATTGCAGTGGTCG

TCAGATCCTATCAGTGATATGGTTTCTGATTCTGTTGTGGCTATGATCTTGAATATTAGC

CGGGAAGGTCCAAAAGTGATGCCAATTGAAGAGGCCACCAAGACTGCGGAGGAGACAGAA

AAGATGGTCCAAAAGGTAATCCATTCACTGTTTGCCTCCCTGTTTGGAGATGTTAAAGTC

GTGGATGGTAAGTTGGTGATAACCGTGGATGGGGTTGTGGCGCGTGTGGATGGGAAGAAT

GGTGAGGTTGAATGTGATAACGAGGCTTTGAAGGAGAGGGTGGAGTCTGCTTTCAGGAGG

ATACAAAGTGCTGCTAGGCCAATCCCCCTGTCATCATCTTGA

>TRINITY_DN10010_c0_g1|m.24 TRINITY_DN10010_c0_g1|g.24 ORF TRINITY_DN10010_c0_g1|g.24 TRINITY_DN10010_c0_g1|m.24 type:5prime_partial len:120 (+) TRINITY_DN10010_c0_g1:3-362(+)

CTACTTGAAAACGCGATAAAAGATACACTCGATGAAAAGTTTATGAAGTTGGCCATTCCC

AGCTCTGTTTCTTACAAAGAAAAGTACTATAGCACTCAAAGAAACAAATTAGTGTCTTCT

CTAATGATCAGAAAAACATATAATTTCACACTAAAGAACCATATCTGTCTGCAAATAGTA

CAAAATGTGAGGCCAATCACTCTGAACCCTCATGGTTTTCTTCAGTTTCTAGTTAGCAAG

GCAGTGGCTCGAAAAGGCAGGAGCCTGCTGCTTGATTACACAAGTCATACATTCGCAGTG

ATGAATATCAAGAAGAGAGAATTCAGAGTGATGATACATGAAAAAGCCACTCTTGGCTAG

>TRINITY_DN10011_c1_g1|m.25 TRINITY_DN10011_c1_g1|g.25 ORF TRINITY_DN10011_c1_g1|g.25 TRINITY_DN10011_c1_g1|m.25 type:5prime_partial len:219 (-) TRINITY_DN10011_c1_g1:236-892(-)

CTCAATCTCTCCACTCCCACAACTCGATCGCTCCCAAGGAATTCGCAGCTCGCAATGGGG

TTAACCACCGCCACCACCGATCAAGAGAAATCGCCGTCAGTCGGCGACGGGTCTTCGCCT

ACGAGCCTGGTTCGGACGGCAGAGACGGTGCTGCGGGTGGCGCCGATGGGGCTCTGCCTG

GCGGCGCTTGTGGTGATGCTCAAGAACTCCGAGGACAACGACTACGGATCCGTCTCCTAC

TCCAACCTCACGCCGTTTATGTATCTCGTCTACGCAAACGCCATCTGCGCCGTCTACTCT

CTCATCTCCGCGTTCTTCACTGCCGCGGCGGCCCCGCGGCCCGCCCTGTCCCGGGCCTGG

GCCCTGTTCCTGCTCGATCAGGTGCTGACCTACGTGATCCTAGCAGCCGGGACAGTCTCG

GCAGAGATCGTGTACTTAGCATACAACGGAGACCAGCAGGTGACATGGAGCAAGGAATGC

GGCGTCTTCAACGGGTTCTGCAAGAAGGCTACAACTTCAGTCGGTATCACCTTCGGAGCA

GTGGTTTGCTACGCGATGCTCTCCCTCATCTCCTCCTACCGACTTTTCAGCGCCTATGAA

GCCCCCATCCCATTCCTCAGCAGCAAAGGCGTGGAGATTGCGGCCTTCCCTCGTTAA

>TRINITY_DN10011_c2_g1|m.27 TRINITY_DN10011_c2_g1|g.27 ORF TRINITY_DN10011_c2_g1|g.27 TRINITY_DN10011_c2_g1|m.27 type:complete len:236 (-) TRINITY_DN10011_c2_g1:640-1347(-)

ATGTTGGCTATCTTTGGTGGTTCGGTTGTGAAGGCCCCTGAGGAGCTGATTCAGGCAGGC

AACCGGAGCCCGACGCCGAAGGAGAGCGCGGCGAAGCTCGTCGACGCCTTCGTCCGGCGG

TTTCCGTCGTCGGTGAACGTTAATATTGGATCCTTGGCGCAGATTGTGTACTCTCACGAC

ACACAAAACTCCTTGCAACCAAGGTCATTTGCGTCGAAGGATGAAATCTTCTGCTTGTTT

GGGGGCACCCTAGAGAACCTAGGGAGCATGAAACAATACTACGGCCTCGCCAAAAACGCA

ACGGAGGTCATTCTGGTGATGGAAGCATACAAGGCTCTGCGCGATCGCGCCCCCTACCCG

ACGAACCACATGCTCGGCCACCTCGACGGGGGGTTCGCCTTCGTCGTCTTCGACAGGGCC

ACCTCCAGGATTCTGGTTGCCTCTGACCAAGAGGGTAAGGTTCCCTTGTATATGGGGATC

ACGGCCGATGGATGCCTGGCTTTCTCTGATGATGCGGAGATACTTAGAGCTGCTTGTGGG

AAGTCTCTTGCAACTTTTCCTGCAGGGTGCTTCTTCTCAACTTCCACTGGGCTGACAAGC

TATGAACATCCAAAGAACAAGGTGACTGCAATCCCAGCAAAGGAGGAAGAGATGTGGGGT

GCAACCTTCAAGATTGAGAGACCTCATCTGCAACCAACTGCTGAATAG

>TRINITY_DN10013_c0_g1|m.28 TRINITY_DN10013_c0_g1|g.28 ORF TRINITY_DN10013_c0_g1|g.28 TRINITY_DN10013_c0_g1|m.28 type:internal len:167 (+) TRINITY_DN10013_c0_g1:1-498(+)

GAGGCCGGACCTGCAGCTGAACAAGAACCTAAAATGGAAACTAAATCAGATGATGATAAT

GATGATGTTGAGGATCCAGAAGAAAACATGTCAGATGATGATAATGAAAATGAGAATGGT

GATGAGGAAGAGGAAGATCCTGAAGAAATAATCGAAGACGACCAGGAAATGAACGATGTT

GCGCTTTCCAAAGATGGAGTCCAGGAGGATGAGAATGCAGCTAAAACAGATGATTTAATG

GCTGAGACTGAAAATGTAACAGTTGAAGAAGACAATGGTAATAAGACCGCGAGCAACAAT

AATATTGAACCAATGGATGAGAAATCAGATAAGGAGAAGGAAGATGACAAACGGTCCAGG

ACTGCAAACTCTGATTCTGTAAAAGATGAAGTCGTCGATAAAGAAGTATTGCAGGCGTTC

AGATATTTCGATCATAACCGTGTGGGCTATATCAAGGTTGAAGATTTGCGATGCCTACTT

CATAATTTGGGCAACTTT

>TRINITY_DN10014_c0_g1|m.29 TRINITY_DN10014_c0_g1|g.29 ORF TRINITY_DN10014_c0_g1|g.29 TRINITY_DN10014_c0_g1|m.29 type:5prime_partial len:334 (-) TRINITY_DN10014_c0_g1:218-1219(-)

TCATCTCAAATCATCGCTCTCGCATCTCAATCCTCAGAATCCAACCCGATGGACGAACAA

CCCGCGGCCGGCGACGGCGGAGGCACCGGGACCCACCGCTACCCGCCGGAACCGACCCGC

GACTGGTCTGATCTGACCCCGGTCCTCCTCGTTAATGTGTTCCGCAGGCTCAGCGTACAG

GACCGATGGACGGGGCCGATGCTAGCGTGCCGATCTTGGCTCGAGGCGGCCAGAGATCCG

TCGCTCCTCTCCTCGTTCGATCTCGAGCCCTACTTCGTTACCGCCGATTCGGCCCGGTGG

TGGACCGCCGGGTTCGAGCGGCGGATCGACTCAATGGTTCGGTCGGTGGCTGATTGGGGT

GCCGGTTCGGTGAAGGAGGTCCGGATCCGGCACTGCTCGGATCGGTCGCTGAGTTTGGTG

GCTGAAAGGTCACCGAATCTTGAAATCCTTTCAATTGGAAGCAGTCAGAATGTAACTGAT

ACATCTATCTCTAGGATCACACTAAGTTGTCCGATGCTTAGGGAATTAGACATCAGCAAC

TGCTACGAGATATCCTACAAGTCACTCGAAATGCTTGGAAAGAACTGCCCGAACCTCATA

ATTCTCAAAAGGAATTTCATGAACTGGCTTGACCCTTCACAGCATGCAGGGATAGTCCCA

GATGAGTATCTAAGAGCTTGCCCCCAAGATGGTGATAGGGAGGCCTCTGCGATCTCACGG

TCCATGCCAAAGCTGAAGCACCTTGAACTCCGCTTCTCCAAGCTTTCCGCCACCGGTCTG

ATCAATGTGTCGTTGGGATGTCACGAGCTGGAAGTACTGGATCTTTTCGGGTGTGCAAAC

CTGACGAGCAGGGGCATCGAACAAGCTTCCAGTAATCTGAAGAACATGAATAAGCTAACA

AAGCCAAACTTCTACATCCCGAGATCTGTCTTTCATATAGAGAGGTATGGGCACTGGAGG

CTTTACGATGAGAGGTTCCAAACAAATGTATTCCAGATCTGA

>TRINITY_DN10015_c0_g1|m.30 TRINITY_DN10015_c0_g1|g.30 ORF TRINITY_DN10015_c0_g1|g.30 TRINITY_DN10015_c0_g1|m.30 type:complete len:186 (+) TRINITY_DN10015_c0_g1:110-667(+)

ATGCGTGAGTATGAAGTGGTCGTCCTTGGCGCTGGAGGCGTTGGCAAGTCTGCCCTCGCT

GTGAGATTCGTTCAAAACGAGTTTTTGGAACATTACAATCCAACAATAGAGGAGGAATAC

CGGAGAGAGATCGTCGTAGACGGTGAGACCATTGAGCTAGATATTATGGACACAGCAGGA

ATCGAGCAATTCACTGCCCTCAATGAGGTTTATGTCAATACCGCGCATGGATTTTTGCTC

GTTTTTAGTTTGACTCATGAGGGCAGTCTTCAGGAGTTAGAACTCATACGCCAGCAGATA

TATCACATTAGGAGCGGACAGCTGAACCTACCTATGGTAATTGTTGGTACCAAGATGGAT

CTGTACAATGAACGTGAGATTACACGCCAGGACGTTCAAGAACTTGCTTCAAAGTGGGAC

ATTCCTTTCTATGAAACATCTTCTAAGAAGAATTGGCATGTCAACGATGTCTTTTTGGAT

CTCACGAAGCAGATGCGAGCACGCTTCCCAAATGAGCCTCCAAGGAAGCGCCAGAAGAAG

GATTGCATCATTATGTGA

>TRINITY_DN10016_c0_g1|m.31 TRINITY_DN10016_c0_g1|g.31 ORF TRINITY_DN10016_c0_g1|g.31 TRINITY_DN10016_c0_g1|m.31 type:complete len:394 (-) TRINITY_DN10016_c0_g1:217-1398(-)

ATGGCTAAGAAGGCTGTTCACTTTGGCGCTGGCAACATCGGCCGTGGCTTTGTCGCCTGC

TTCCTCCACAACTCTGGCTACGACGTCGTCTTTGCCGATGTCAACGACACCATTGTCAAC

CTCATCAACGAGACCCCCTCATACCGTGTCATCGAGGTCGGCTCTGAGGGTACCACCGAG

AACACCATCACAAACTACCGAGCCATCAACTCGCGAACCCACGAGGAGGATCTGATCGAG

GAGATCCGCACAGCAGAGGTCGTCACCTGCTCCGTCGGCCCCAACATCCTCAAGTTCATC

GCTCCCGTCATTGCCAAGGGTATCGACCGCCGATCAACCGACGACGCTCCCCTGCACGTT

ATTGCTTGCGAGAACGCCATTGGTGCCACTGATACCCTTGCCGAGCACATCAAGGACCCC

CGCAACACCTCCCCCGAGCGTCTTGAGGACCACCACCTCCGCGCCCGCTACGCCAACTCT

GCTATTGACAGAATTGTCCCCGCTCAGGACGCCGATGCCGGTCTTGATGTTACCCTCGAG

AAGTTCTTTGAGTGGGTTGTTGACCGCACTCCCTTCGAGGATGTTGGCATTCCCGACATC

AAGGGTATCAACTGGGTTGACAACCTCGGCCCCTTCATTGAGCGCAAGCTCTTCACTGTC

AACACCAGCCACGCCACCGCCGCCTACCACGGTTACAACCGAAGAAAGCGCACCGTCTAC

GATGCTCTTCAGGACAAGGAGATCATGGCCGAGGTCCGAGGTGCCCTCATGGAGACCAAG

TCTCTGATCGTCTCCAAGCACGCCATCGATGAGGAGGCCCAGGCTGCCTACGTCAACAAG

ATTGTCAAGCGAATTGGCAACCCTCATCTTGAGGATGCCGTCGAGCGCGTTGGCCGTGCC

CCTCTCCGAAAGCTCTCCCGCAAGGAGCGCTTCGTCGGCCCCGCCGCTGAGCTTGCCGAA

AACGGCCAGTCCATCAAGTACCTCCTCGACGCCATCGAGATGGCCTTCCGTTTCCAGGAA

GTCGAGGATGACGAGGAGTCTAAGCAGCTTGCCAAGATCATGTCCGAGAACGGACCCGAG

GATGTTGTCAAGCAGGTCTGTGGCATTCAGGACAGCGAGAAGATCTTCCCCCAGCTTGTG

CAAGTTGTGCAGCGCGTCCAGGCCGACAGCGCCGAGGACTAA

>TRINITY_DN10018_c0_g1|m.34 TRINITY_DN10018_c0_g1|g.34 ORF TRINITY_DN10018_c0_g1|g.34 TRINITY_DN10018_c0_g1|m.34 type:internal len:113 (-) TRINITY_DN10018_c0_g1:1-336(-)

GGGCACAAGCATAGGATGTTGCATTGCTTCTCTTCTTTCTTTCTTTTTCTTTTTTTTTCA

ATCTTGACAACAAAAATGACCACTAGCATCATATCTACATTACAAGCTTTCCCCTCCATT

GATCAGCAGATGCTTAGAATTTGCCCATTAAGCTCATTTTCAACATTCTATTTTCATTGC

CTTAATTTCTGGCATGTACACAATAATACAAACCTTACCCAATTTTTAAAGAGAAATGAA

GGGGTAGTTTTTTTTTCTTCTTCTTTTCTCGGAACTTTTACACGCAGTCACTATCTCGTC

GGTGTTGCCGGAGGCCTCACCGGCAGTCACTATCTC

>TRINITY_DN10019_c0_g1|m.35 TRINITY_DN10019_c0_g1|g.35 ORF TRINITY_DN10019_c0_g1|g.35 TRINITY_DN10019_c0_g1|m.35 type:complete len:253 (+) TRINITY_DN10019_c0_g1:249-1007(+)

ATGCTGGACATGCGACGTCCACCAACTATTATTTATAGGCCAATGCGGCCATCTGATCTT

GTAGCTCTTGGGCAGATCCATGCTGCCTTATTTCCAGTAAGGTATGAGAGAGAATTTTTT

TTAAATGTTGTCAATGGCAATGGTATCATCTCTTGGGCTGCTGTTGCTATTGACCAGCCT

GATGGCCAAAGGGATGAACTTATTGGATTTGTCACCACGCGAACCATTGCAGCAAAAGAT

AGTGAGATAGCCGACTTGCTAAGTTATGATATTTCGAGGAAAGATGCAACTCTAGTATAT

ATTTTGACACTTGGTGTGGTTGAACGCTATCGAAATCTTGGAATAGCTACTTCACTTGTT

CAAGAGGTTACAAAATATGCGTCAAGTATTACAAATTGCAGAGCTGTTTATCTGCATGTT

ATTTCTTACAACCTTCCAGCCATCCATTTTTACAAGAAGATGCTTTTTAAGCTTGTAAGG

AGCTTACCGAAGTTTTACTACATAAATGGGCAACATTACGATTCATACTTATTTGTTTAC

TATGTAAACGGTGGTCGATCTCCCTGCTCCCCACTAAACATTTTGGCAGCAGTTGCATCA

TACATTGGGGGCGTATTAAAGTCTCTGTCTGCAAAACTCTGGAAGAGCAATGAGAAGAAG

ATCTCAAGATGGTCTAGATGTAAAGAGACCAGCACCATACTAGTCACACACAACTCGAGA

ATCTTGAGCTCGGAGACTGCTCTTTGTGAAGCTGTTTAA

>TRINITY_DN1001_c0_g1|m.36 TRINITY_DN1001_c0_g1|g.36 ORF TRINITY_DN1001_c0_g1|g.36 TRINITY_DN1001_c0_g1|m.36 type:complete len:1630 (+) TRINITY_DN1001_c0_g1:604-5493(+)

ATGGGTATAGTGGGTTACCCATTTCTTGACCTGATACAAAGAGTTAGATCTTGGTTTGGT

GGTGATTCTGTTGGTTTGGGGATGTCTGGTTATTGCCACGAGTGCCGGGGTTTGTTTCCG

GCGCCCGGCTACGGCCACCGGTGCCGGAGCTGCCGGCGGTTGTTCTGTAGGAAGTGCATG

CAGGTCGGCTCTGACCAGTCCAAGTTCTGTAAATTCTGCTTCCATGCAATTAGCAGCAGC

AGCCGTGCCGAGGTCTCTGCCGGGCTGCCGCAGGCGATACCGGATTCGCCCTTGTCGAGA

ATCCGCAATTTCAGGCTCGCGCATTTGGCTGAGTTCTCTAACTTCTCTTCTCCGCGCTCG

TCCAGGAGTGATGATGATGAAGATGGTGATGATAGCGGAAAGAATTTTTTTAGTTCGCCG

AGTGAGTTGTCTCAAGATGTTTCTGATGTAGATTCGGTTAGTATTAGTACCGGTCTCGAG

TTACATAGTTTTAAATCTGTGAATTCTAGTCTTTTGGATAGTCCTAGTAGAGGTGGTGAG

ACCACAACGGATAACAATCCTTTGGATCGAGAGGCTCCGAGTTACTTTAGAAAAACTGGA

GCTGAGGCCGAGGATCTGTTGGAGAGTTCCCATGACTCCGGGCATAGGCTTTCGGTGTAT

AGAGATCATGAGACCCAGAAGGTTCAACGGCCATTGGATTTTGAAAACAACAGGCTTATT

TGGGACCCTCCGCCGCCCGAAGATGAGAACGACGATATGGAGACCGGATTCTTTGGGTAT

GATGACGAGGATGATGATGCTGGTGACTCGAGTGATGTCTTCTCGTCTGGTAGCTTCAGT

AGTCATTCCTTGAGTACAAGGGAGAAGCCTAGTGATGCACATAAGGAGCAGTTGAGGAAC

TCAGTTCATGGGCATTTTAGAGCGCTTGTTTGCCAGTTATTGAGAGGGGAAGGGCTTCAG

GTTAGAAGTGATGATGGCGGGAAGGGATGGTTGGACGTAATTTCCTCGTTGGCATGGCGA

GCTGCGACTTTCATCAAGCCGGATACCAGCAAAGGTAGCAGCATGGATCCTGGTAATTAT

GTGAAGGTCAAGTGTATAGTTTCTGGAAATCTGAGTGATAGCACCCTCATAAAAGGAGTG

GTTTGTACAAAAAATATCAAGCATAAACGGATGGTTTCTCAGCATAAAAGTCCAAGATTA

TTTCTTCTGGGAGGAGCACTGGAGTACCAAAGAGTTACGAATAGGCTGGCATCTATAAAT

ACTGTCCTGGAACAGGAAATCGAACACCTAAAGATGTTCCTTTCAAAAATAGTGGCCCAT

CGGCCTAATGTTCTGCTTGTAGAAAAGAGTGTGTCCTCATATGCTCAAGAATATCTTCTG

ACAAAGGATATATCTCTGGTGTTGAATGTTAAGCGGAAACTTTTGGAGAGAATAGCCCAG

TGTACTGGTGCACAGATTGCTCCATCCATTGATAGTATTTCTTCAGCTAGATTGGGACAT

TGTGACATGTTTCGGGTAGAGAAGTTTTTTGAAGAGTGTTCAACTGGAAGCCATCCAAAA

AGGAAATCCATGAAGACACTGATGTTTTTTGACAACTGTCCACGGCGTTTAGGTTGCACG

GTTCTTCTACGTGGCACAAATCCTGAAGAACTGAAGAAGGTTAAACATGTAGTCCAATAT

GCAAGCTTTGCAGCCTATCATCTGTCACGTGAGACCTCCTTCCTTGCAGATGAAGGTGCC

ACACTTCCCAACGTTCCTGTGAATCCTCCCATGGCGGCACCAGCGAAGATAATGGATGCT

GAGATTGGAATTTTGAAGACTTCAACTGTCTCTGTTACACAACCAAGAGTTTGTGGCGGA

CATAAAAGACTTGGTTCTTGCCAAAATAACACCATCAATTGCTACTTTCCAAGCTCAAAT

GTTCTAGCTCTGCATGGGGATATATGTTCTGGTGACAGTTCTCAATTAACTGGGCATCTT

TCTACTAGATCCAAAGGACCTTGTGAACCTTCAGATTTTCCCACTGATATAACAACACCG

GAAAAAGTGACAGGGAATACACTTGAAGAAGGACAAAACTTGGAATATGATAAGGAACTT

GTAGCAGTGCATGAGGGTAAGCCAACTGGCACAGATGGTGAAAATGACGCTACAGATTAC

TTCTCGACTGCTGACAGTCAGAGCATCTTGGTTTCCTTATCAACGACATGTGCCCTTAAA

GGCACTGTTTGTGAACCCTCTCAACTCTTTCGAATTAAATTTTATGGAAGTTTTGATAAG

CCCCTGGGGAGGTATCTTCGTGATGACTTATTTGATCAGACATCCTGCTGTCAATCTTGT

AAAGAGCCAGCAGAAGCCCATATTCGGTGCTATACTCACCAGCATGGAAACCTGACCATA

AGTGTCCGTCGACATCCTTCCATGAAGCTACCTGGAGAACGAGATGGAAGGATATGGATG

TGGCACCGATGTCTCAAGTGTGAACAGAATAATGGAGTCCCACCTGCTGCTCGTAGAGTT

GTCATGTCTGATGCTGCGTGGGGCCTTTCTTTTGGAAAATTTTTGGAGCTTAGTTTTTCA

AATCATGCTACTGCTAATCGTGTTGCAAGTTGTGGCCATTCTCTTCAGAAGGACTGCCTT

CGCTTTTATGGGCTTGGAAGCATGGTTGCCTTCTTTCGATATTCCCCTGTTGATATTCTT

TCTGTTCGATTGCCACCTTTGGTATTGGATTTCGCTTGTGAAAGTCAGCAAGAGTGGGCA

AAAGAAGAGGCAGCTGCTATCGCAAGGAAAATTGAATTCCTGCATACAGAGGTTAATGAT

GTGCTCCAAGCATTTGGGAAAAGGATTACAACCTCTGAGTGTGAACCTGTGAAGACAAGC

ATCTATAAATACATTACAGAGCTAAAAGATCTACTGAATAGGGAAAGAAATGAGTACGAT

GTCTTGCTGCAACCATCTGCCACGGACAATAGTTCACTATTTCAGGAAACAATAGATATA

CTTGTGCTAAATCGTTTAAGGAGATTCCTGTTAATTGATTCTCACATGTGGGACCGTCGG

CTCTGTCTCTTGGATTCATTTTCTAAGTCGAAAACTTCTGCCTCGAAACTCAAGCCACAA

ATCTCTGTGATTGCAAATCACACCTGTCTGAAGGAATGGAAGGTTGAGCCAACTGATAAT

GATAGGAGTTTTAGAAAATCTCCCGACGAAGCTATCTCAATGCTTGCAAATTTGTCGAAG

CATCATGAGGAGTTGAATGAGTGTGTTTTTGAGTGTAATTCTAACAATTTAGTCGAAACG

GACTGGTTGATTGAGTCCATTGAGGGTTATGCTAGTTCTGCAGCTCTCAACTTGGTTTCT

GGTCAGTATTACCAAGGGGATGACGAAACACAGGTAGCTTCTGAAACTTGCATTGGAAGT

TCATCCTTGGAGAGATTACCATCAGCTGCCTCCACTCTGTCTGATAAGATTGATTGCTTA

TGGAGTGGTAATCCAGAAGCTGATGGAGTTGGATCTATCAGTCTGATGGATAATCCACTA

TATAGAAAGGTGATTTCTCCTCTCAGAGTTAATTCTTTTGATTCTGCCTTGAGATCCCGA

GCCAGATTGCGTGGACTATCTCCTGCTTCATTGCATTTGACGTCAGTTAGATCATTTGAT

GCAACAGGAGATTTCACTAATATGGATATTTCACATAGATCTCCAGGATCAAAGAGATTA

AACTTTTTTCTGAGTGATAACCCTGTACTTATATCCTCAGCTTATCATATGGTTGATGAA

GGAGCGCGGTTAATGCTCCCTCTAACTGATTGTGATGATTTAGTCATTGTGCTTTATGAC

AATGAGCCCACCACTATTATATCATATGCCATTAGTTCTCAAGATCATGCTGACTTCATA

ACTTCAGATCATCAATCGAGTGAGGATGGCATTGGCAGGCCGTCAATATCTCAGTTTCAG

TCAGATGTGGAGCCTCAGCGGTTACGAGGAAATTCTTCTGTCGATCCCAAGGCCACTCAT

TTCAGGATTGCTTTTGATGACGAGTCTTCAATACCTAAAGACAAAATGAAGTTCTCTGTG

ATCTGTTATTTTGCTAAGCAGTTTGCTGCACTTAGGAAAAAGTGTTGCCCCGAGGAAGCT

GATTTCTTGCGTTCTCTGAGCCGCTGCAAGAGATGGAATGCTCAAGGTGGTAAAAGCAAT

GTGTACTTCGCTAAGTCACTGGATGAGAGATTTATTATAAAGCAAGTGACAAAGACAGAG

CTGGATTCTTTTGCGATCTTTGCCCCTGAATACTTTAAGTATATGACTGAGTCAATAAAT

ACTGGAAGCCCCACTTGCCTTGCTAAAGTCCTTGGAATTTACCAGGTGACCGTCAGGCAC

TCAAAAGGTGGGCGGGAGATGAAGATGGATTTGATGGTGATGGAGAATCTCTTCTTTGGT

AGGACCTTATCAAAAGTCTATGATCTCAAAGGCTCGTTGCGTGCTCGGTACAATCCTGAT

ACTTCTGGAAGTGACAGTGTTCTTTTAGATCTGAATCTATTAGAGTCAGTGAGAACAAAG

CCGATATTTCTCGGAAGCAAGGCAAAGAGAAGCTTGGAACGAGCTGTCTGGAATGACACG

TCATTTCTTGCGTCTGTTGATGTCATGGACTATTCGTTACTGGTTGGCATTGACGATGAA

AGGAAGGAGCTTGTTATGGGAATCATAGATTTCATAAGGCAGTACACATGGGACAAGCAT

CTGGAAACATGGGTGAAAGCTTCAGGCATCCTCGGGGGCCCCAAAAACGAATCCCCAACG

GTGGTGTCTCCTTTGCAGTACAAGAAGAGGTTCAGGAAAGCAATGTCAAAGTATTTCCTC

ACCGTTCCTGACCAGTGGTCATCTCCTTGA

>TRINITY_DN1001_c0_g1|m.38 TRINITY_DN1001_c0_g1|g.38 ORF TRINITY_DN1001_c0_g1|g.38 TRINITY_DN1001_c0_g1|m.38 type:complete len:134 (-) TRINITY_DN1001_c0_g1:1084-1485(-)

ATGTGCATCACTAGGCTTCTCCCTTGTACTCAAGGAATGACTACTGAAGCTACCAGACGA

GAAGACATCACTCGAGTCACCAGCATCATCATCCTCGTCATCATACCCAAAGAATCCGGT

CTCCATATCGTCGTTCTCATCTTCGGGCGGCGGAGGGTCCCAAATAAGCCTGTTGTTTTC

AAAATCCAATGGCCGTTGAACCTTCTGGGTCTCATGATCTCTATACACCGAAAGCCTATG

CCCGGAGTCATGGGAACTCTCCAACAGATCCTCGGCCTCAGCTCCAGTTTTTCTAAAGTA

ACTCGGAGCCTCTCGATCCAAAGGATTGTTATCCGTTGTGGTCTCACCACCTCTACTAGG

ACTATCCAAAAGACTAGAATTCACAGATTTAAAACTATGTAA

>TRINITY_DN10021_c0_g1|m.40 TRINITY_DN10021_c0_g1|g.40 ORF TRINITY_DN10021_c0_g1|g.40 TRINITY_DN10021_c0_g1|m.40 type:complete len:441 (+) TRINITY_DN10021_c0_g1:128-1450(+)

ATGACTTCTCCAAAGATCTCCGCTCCCTCAATCAAAGACCTCAGTGCAAAGCTTAAAGGA

GTGTCAATCCAAGCTCCGACCAATGGAGCAGTGGATTCTTTGGAAGGGGGAGAGAGATTT

TCGATGGTAAGGAGAAAAAAAGATTTCAAGTGTATGGAGAGGGTTAAGGGGAGGCTGGTG

AATGTATTGGAAGGACTAGAGCTTCATACCAGTGTCTTTAGTGCCGCCGAACAGAAGAGG

ATTGTCGATTATGTGTATGACCTTCAAGATAAGGGGCGGAAACGACAGCTTAGAGAACGA

ACATACTCAGAGCCAAAGAAGTGGATGCGAGGCAAAGGACGAGTCACCTTGCAATTTGGT

TGTTGTTATAACTATGCCACAGACAAGAATGGAAATCCACCAGGTATTATACGTGATGAG

GATGTTGACCCAATTCCTCCACTATTGAAGTCGATGATTAAGAGGATGGTCACATGGCAT

GTTTTGCCTCCTACGTGTATTCCCAATAGTTGCATCATCAATATCTATGATAAAGATGAT

TGTATTCCTCCACATATCGATCATCATGATTTTGTTCGGCCTTTTTGCACAGTCTCTTTC

ATAACAGAATGCAGTATATTATTTGGCGCCGAGCTCAAGGTACTTGGTCCAGGAGAGTTC

TCAGGTTCTACTGCTATTTCTCTACCCGTGGGCTCTGTGCTCATCTTAAAAGGGAACGGT

GCTGATGTCGCTAAACACTGTGTTCCTGCTGTCCCTGCCAAAAGGATATCTATAACTTTC

AGGAAGATGGACGATAGTAAGCTTCCTTTTAGGTTCACTCCCGATCCTGAATTGCAGAAT

CTTCGGCCTCTTCCGCTCCCTGCGAATGGATCTTCAATTCAGCCGATAGATAATCGGGCT

TCAGTTTCCCCTGTACAACAGCAGTTCCAACATCGTACTCCACCATCACAACAAGCCCAA

GAAACTAGTGGAGAGAAACTTGTTAGACAGTTCAATCTCAGTCAAGATGATTTTCCTTCG

TTGGGTTCTAGCTCAACACCTGTCTCGGCTGTGCAACAACAATCCCAACATTGGACTCCA

CCTTCACTAGCCCAACAGACTAGGTCTCATCGTAGGAGTTGGAAATCGGAAGATCAGAAC

ATAAGCAAACATAGTTCTGGTTTTCGGAGTGGGAACTCAGAAGAACTGAGCACGAGGGGA

AGGAGTCAATCGGTAGCCAGCAATGAGAAACCAGTTAGAGCATTCGACCTCAGTCAAGAT

GGTTTTCCTTCGTTAGGTTCTAGCTCCTCCATGAGAAAGCCTGGGAGATCCTTGCGACAA

TAA

>TRINITY_DN10022_c0_g1|m.41 TRINITY_DN10022_c0_g1|g.41 ORF TRINITY_DN10022_c0_g1|g.41 TRINITY_DN10022_c0_g1|m.41 type:complete len:596 (-) TRINITY_DN10022_c0_g1:734-2521(-)

ATGAAGGGCGCCTCCTCCTTCTCTCAGCCCACCACCCCAAAATCGGGCTTCCACTTGAGA

AAAGTTTCGGTAGGAAACTTCAAGGATTTGGAGATGGGCGGCTACAGCTTCCCGTCGCCG

ACGCCGTCTCCGACCGCCAGCTCCGCCCCGGCGCTGGTCCTGTCTAACTCCGGCAAGCGG

ATGGACCAGACCGGCAGCAACAGCCGGAAGAAATACGTGAAGCAGGTGACGGGGCGGCAC

AACGACACGGAGCTGCATCTGGCGGCGCAGAGGGGGGACCTGGTGGCGGTCAGGATGATA

TTGGGGGAGATTGATGCGCAGATGACGGGGACCGGGGTGGAGTTCGATGCCGAGGTGGCG

GAGATCCGGTCGGCGATGGTTAACGAGCTGAACGAGATGGGGGAGACAGCGCTGTTCATC

GCGGCGGAGAAGGGACATTTGGATATGGTGGTGGAGCTGTTGAGGTACACGGACCGGGAG

AGCTTGGTGAGGAGGAATGGATCGGGGTTCGACGCGCTGCATGTGGCTGCTAGAGAGGGG

CACCAAGCCATTGTGCAGTTACTCTTGAACCATGACCCGACACTTAGCAAGACGTTTGGC

CTATCAAATGCAACTCCACTTATTTCTGCTGCAACAAGGGGTCACCTTAATGTTGTAAAC

GAGCTGCTAGCTCATGATTCCAGTTTGGTTGAACTGGCCAAGTCTAATGGAAAAAATGCT

TTGCATTTTGCTTCCCGGCAAGGACATGCTGATGTTGTGAAAGCACTGTTGCAGAAAGAT

CCTCAGCTTGCTAGAAGAACTGATAAGAAAGGGCAGACTGCTTTGCACATGGCAGTGAAA

GGAACAAGCAGTGATGTAGTTAAAGCTCTAGTTGAGGCTGACCCAGCGATTGTCATGCTA

CCGGACAAGTTTGGTAATACTGCATTGCATGTAGCGACTAGGAAGAAGCGAGCAGAGATT

GTGAATGTTCTATTACTCCTCCGAGACACCCATGTGAATGCATTGACAAGAGATCACAAG

ACTGCTCTTGACATTGCTGAAGGCTTACCTCTTTCGGAAGAATCCCAAGAGATTAAGGAA

TGTTTATCCCATAATGGGGCTGTCAGGGCCAATGATCTAAATCAGCCTCGAGATGAGCTG

CGGAAGACAGTGACAGAGATCAAGAAGGATGTACATACTCAATTGGAACAGACAAGAAAA

ACTAACAAAAACGTATATGGGATTGCTAAGGAGCTCAGGAAACTACACAGAGAGGGCATC

AACAATGCCACAAACTCTGTAACTGTGGTGGCTGTTCTCTTTGCTACTGTGGCATTTGCT

GCAATCTTCACGGTGCCTGGTGGTAACGAGGATAATGGAGTTGCAGTAGCTGTTGAGAGT

CCTTCTTTCAAGATATTCTTCATATTCAATGCCATAGCTCTTTTCACATCACTGGCCGTG

GTGGTGGTTCAGATAACACTTGTCCGGGGGGAAACAAAATCAGAGAGGAAGGTTGTAGAG

GTGATCAACAAGCTTATGTGGTTGGCGTCGGTCTGTACCACAGTTGCTTTCATCGCATCC

TCGTATATTGTGGTTGGCCGCCACTTCCAGTGGGCAGCGGTCCTGGTTACATTGATAGGT

GGAGTTATTATGGCAGGGGTTCTAGGAACCATGACATACTATGTGGTAAAGTCGAAGCGC

ATCCGTAAGATAAGAAAGAGAGTGCGGTCAAAGAAGAGTGGGTCGAGCTCATGGCATTAT

AACTCTGAGTTATCCGATTCTGACATAAATCCTATTTATGCTATTTAA

>TRINITY_DN100230_c0_g1|m.44 TRINITY_DN100230_c0_g1|g.44 ORF TRINITY_DN100230_c0_g1|g.44 TRINITY_DN100230_c0_g1|m.44 type:complete len:480 (-) TRINITY_DN100230_c0_g1:324-1763(-)

ATGAGCGCTCTTGCGACGAAGCAGCAGTCGCTGAAGATCTTTGAGAAGTTGAAGACGAAG

CCTACGAACAAGATCTGCTTCGACTGCGGCCAGAAGAACCCTACCTGGACATCAGTCCCC

TTCGGCATCTACCTCTGCCTGGACTGCTCCGCCAACCACCGAAACCTGGGTGTGCATATC

TCATTCGTGCGATCAACCAACCTCGACCAGTGGCAATGGGACCAGCTGCGCTTGATGAAG

GTCGGTGGCAACGAGTCTGCCACCAAGTTCTTCCAGCAGAATGGCGGCACCGCTGCCCTG

AACAGCAAGGACCCCAAGACCAAGTACCAATCTAACGCTGCCACCAAGTACAAGGACGAG

CTTAAGCGACGCGCTGCGCGGGACGCCCAAGATTTCCCCGACGAGGTTGTCATCACCGAT

GCTGTCGATGACGGCTCTGCTACCCCCGCCGGCGAGCCTGACGACGACTTCTTCTCTTCC

TGGGACAAGCCTGCCATCAAGAAGCCTACACCCCCCGTTTCTCGCACTGCGACCCCGCCT

GTGATGGGCCGAACGCCCTCTCCTTTCCTCAACGCCGGTAACGGCAAGGACATTGCCCGC

GCTTCTTCGCCTCTTGCCCGAAACGCCTCGAGCGAGTCCAAGCCCGCAAGCAGAATCACG

ACATCGGCTGCCCTCCGCAAGACTGGTGGCATTGGTGGTCCCCGCAAGGCCAATGTCCTC

GGTGCCAAGAAGACTACCAAGCTGGGCGCAAAGAAGGTTACTAGCGACGCCATCGACTTT

GACGAGGCCGAGAGGAAGGCCAAGGAGGAGGCCGACAGGATCGCGAAGCTTGGCTACGAC

CCCGACGCGGAAGAGGACCCTGCGACCAAGGCTGCTACTGGATCGGCTGCTGCCATCATT

TCTCCCACCCCCGTCAGCCCCAACAAGAACAGTTATTCATCGCATACACGCCAGAAGTCG

GATGCCGAGGTTGAGCGATTGGGCATGGGCATTGGACGACTGGGCTTCGGCCAAATTGGC

GGTCCCAAGGCTGCCGCCGCCGCCCCCAAGAAGAATGCTGGTGGATTTGGTTCCGTTGGA

CCTGTCAAGGCTGCCGCTGTTGATGACTCGGAGCGATATGCCCGCGACAAGTTCGGTGCC

CAGAAGGGCATCTCATCAGACGAATTCTTCGGCAAGGGTGCCTTTGACCCCAACGCCCAG

GCTGAGGCCAAGACCCGACTCCAAGGCTTCGAGGGCGCGAGTGCCATCTCGTCCAATGCT

TACTTTGGACGACCTGAGGATGAGCCTGAGGAGGAGTACGGCGATCTCGAGTCGGCGGCC

AAGGACTTTGTGCGCAAGTTTGGCATCACGGCCGGTGATGATCTCGAGAACCTGACACAA

ATGGCGGGCGAGGTGTCCTCCCGGCTCCAGGGAGCCATCCGATCTTACCTTGGCAACTAA

>TRINITY_DN100230_c0_g1|m.48 TRINITY_DN100230_c0_g1|g.48 ORF TRINITY_DN100230_c0_g1|g.48 TRINITY_DN100230_c0_g1|m.48 type:complete len:105 (+) TRINITY_DN100230_c0_g1:517-831(+)

ATGGCACTCGCGCCCTCGAAGCCTTGGAGTCGGGTCTTGGCCTCAGCCTGGGCGTTGGGG

TCAAAGGCACCCTTGCCGAAGAATTCGTCTGATGAGATGCCCTTCTGGGCACCGAACTTG

TCGCGGGCATATCGCTCCGAGTCATCAACAGCGGCAGCCTTGACAGGTCCAACGGAACCA

AATCCACCAGCATTCTTCTTGGGGGCGGCGGCGGCAGCCTTGGGACCGCCAATTTGGCCG

AAGCCCAGTCGTCCAATGCCCATGCCCAATCGCTCAACCTCGGCATCCGACTTCTGGCGT

GTATGCGATGAATAA

>TRINITY_DN100230_c0_g1|m.45 TRINITY_DN100230_c0_g1|g.45 ORF TRINITY_DN100230_c0_g1|g.45 TRINITY_DN100230_c0_g1|m.45 type:complete len:221 (-) TRINITY_DN100230_c0_g1:389-1051(-)

ATGTCCTCGGTGCCAAGAAGACTACCAAGCTGGGCGCAAAGAAGGTTACTAGCGACGCCA

TCGACTTTGACGAGGCCGAGAGGAAGGCCAAGGAGGAGGCCGACAGGATCGCGAAGCTTG

GCTACGACCCCGACGCGGAAGAGGACCCTGCGACCAAGGCTGCTACTGGATCGGCTGCTG

CCATCATTTCTCCCACCCCCGTCAGCCCCAACAAGAACAGTTATTCATCGCATACACGCC

AGAAGTCGGATGCCGAGGTTGAGCGATTGGGCATGGGCATTGGACGACTGGGCTTCGGCC

AAATTGGCGGTCCCAAGGCTGCCGCCGCCGCCCCCAAGAAGAATGCTGGTGGATTTGGTT

CCGTTGGACCTGTCAAGGCTGCCGCTGTTGATGACTCGGAGCGATATGCCCGCGACAAGT

TCGGTGCCCAGAAGGGCATCTCATCAGACGAATTCTTCGGCAAGGGTGCCTTTGACCCCA

ACGCCCAGGCTGAGGCCAAGACCCGACTCCAAGGCTTCGAGGGCGCGAGTGCCATCTCGT

CCAATGCTTACTTTGGACGACCTGAGGATGAGCCTGAGGAGGAGTACGGCGATCTCGAGT

CGGCGGCCAAGGACTTTGTGCGCAAGTTTGGCATCACGGCCGGTGATGATCTCGAGAACC

TGA

>TRINITY_DN10025_c0_g1|m.49 TRINITY_DN10025_c0_g1|g.49 ORF TRINITY_DN10025_c0_g1|g.49 TRINITY_DN10025_c0_g1|m.49 type:complete len:199 (-) TRINITY_DN10025_c0_g1:591-1187(-)

ATGAGCGCCTCGAGGTTCATAAAGTGCGTGACGGTGGGGGACGGCGCCGTCGGCAAGACC

TGCATGCTCATTTCCTACACCAGCAACACTTTCCCCACCGACTATGTGCCAACCGTGTTT

GACAATTTCAGTGCAAATGTGGTGGTGGATGGTAACACCGTTAACCTAGGTTTGTGGGAT

ACTGCAGGTCAAGAGGATTACAATAGATTAAGACCCTTGAGCTATCGTGGGGCTGATGTT

TTTCTCCTGGCCTTCTCTCTTATCAGTAAGGCCAGCTATGAGAATGTTTCAAAGAAGTGG

ATTCCGGAACTAAGGCACTATGCTCCAGGTGTTCCCATAGTTCTCGTTGGAACAAAGCTT

GATCTTCGGGATGACAAGCAGTTCTTTGTAGATCACCCAGGCGCTGTCCCCATTACAACA

GCACAGGGGGAAGAATTGATGAAGCAAATAGGCGCTCCTGCCTACATCGAGTGCAGCTCA

AAAACACAGCAAAACGTGAAGGGAGTGTTTGATGCTGCTATCAAGGTAGTTCTCCAGCCA

CCCAAGGCAAAGAAGAAGAAAAAGATCAAGGCACAGAAGAGCTGCTCCATTTTGTGA

>TRINITY_DN10026_c0_g1|m.51 TRINITY_DN10026_c0_g1|g.51 ORF TRINITY_DN10026_c0_g1|g.51 TRINITY_DN10026_c0_g1|m.51 type:complete len:1329 (+) TRINITY_DN10026_c0_g1:49-4035(+)

ATGGCGCCTCCCTTTATTTTCCCGTCGGAGCTCCAATACCTGGAAGAGTTCCCCGACGAC

CACCGCCTCTACGCCGCCGCCCCCGTCGACGCCGCCACCCTCCGCCCCTCCGAACTCGAG

GAGCTCGTCAAAGGAGTCGTATTCGATCTCTCCGACAAGGAGCTCTTCTGCGTTGAGGAG

CAGGACGTGTTCGATCGCGTGTACTCTCTCGTCAAGGGCTTTTCTTTACTGAACTCTTCG

TCCAAGTTCAATCTCGTTGAAACCCTAAGGTCCAATCTCAGCGTGCTGCTCCCCAGCGTC

GACTCACTCTCGCGGGCCCCGCAGGCGGCGGCCGTGGCGTCGCCGGGGGAGTTTAGTGGG

GCCCACATCGCGGCGAGGATCGCCTCGCATCGGAATGCGCTGAAGATCTACACATATTTT

CTTCTAACTGTGGTGCTTGCTGTCGAAGCGGCAGGTCAGGAGGCTACTGCTAGCTCAAAG

GCGGCTACACAAGCTCGAAAGAAGAATTCTACAAATTCATGGAACTGGGAGACGCATAGG

AGCCGGATAATCAATCTGATTGCAAATTCTCTCGAGATCAACCTTTCGTTGCTCTTCGGT

TCAAATGATGTTGACCAGAAGTATCTGTCCTTCATCTCCAAGTGTACTTTCACTTTGTAT

GAGAACCCAGCCTTATTGAGGGATTCCAATACAAGAGATAGTCTTGGTCGAATCATAGGC

ACCATAGCAACCAAGTACCATCAGACAGACCAAACTTGTGCGTCGATCCTTCACTTGATT

CACAAGTTTGACTTCACAGTCCCCCACATTGCTGAAGCAGTCTGTGAAGCTGAGAAGAAA

TATGGTGATGGAAGCCTGGCAATTGCCCTTATTCGTGATATTGGTAGGACTGATCCAAAG

GATTATGTGAGGGATGGAGTTGGTGCAGAGAATGTGGGGAGATTCCTTGTAGACCTTGCT

GACCGTTCCCCTAAGCTTGTCTCCATAAATGTTGGGGTCTTAGTTCCGCATTTTGGTGGA

GAATCATATAAGATTCGAAATGCCCTCATTGGGGTCTTGGGGAAGTTGGTTGCCAAGGCT

TTTAAGGATGTGGAAGGAAATGTGAGCTCTAAGTCTCTGAGGTTGCGGGGAAAGCAGGCT

ATGCTTGAGATCTTGATTGAGCGGTGCAGGGATGTGTCTGCATATACAAGGAGTAGAGTG

CTTCAGGTGTGGGCAGAGTTGTGCGAAGAACATGCTCTTTCCATTGGGCTGTGGAATGAA

GTGGCGGTGGTAGCTTCAGGGAGACTGGAGGATAAGAGTGCAATGGTGAGGAAATCAGCA

TTGAATCTTCTCATCACAATGTTGCAACACAACCCATTTGGACCACAGCTCCGTGTGGTT

GCTTTTGAGGCAACATTGGAGAAATACAAAGATAAATTGAGAGGGATGGAGCCTACTGGG

ACATCTGCAGAGGACGATCATGATGACGAAGTGGTACATGAACACGCCGAGGAAGTGCAG

CAAGAAAGTGTGAGCGACAGTTGTTTGCCTTCAAGCCAAGAGCAGGATTCTACTGTTCCA

GTTATTGGGAATTTGGAACAGGTTAGGGCTTTGGTAGCATCTCTGGAGTCTGGGTTGCAG

TTTTCAAAATGTATAACTTCTACAATGACAATTTTGGTTCAACTTTTGGCTTCCTCTTCG

GCTACTGATGTGGAGAGCACGATTCTTCTGCTGATGAGGTGCAGACAATTTCAGATTGAT

GGTTCAGAAGAATGTCTTCGCAAAATGTTGCCCCTGGTATTTTCCCAGGACAAGTCTATT

TATGAAGCTGTGGAGAATGCATTCATAAATATCTACATCAGGAAAAATCCTGCTGAAACT

GCTCGAAATCTGTTGAGCCTAGTCATTGACTCTAGCATTGGCGACCTTGCAGCTTTGGAA

TCTTTAATTGGCAATTTGGTGTCCAAAGGGGAAATTTCTACAAACACGATATCAGCTTTA

TGGGATTTCTTTAGCTTTAACATCAGTGGGGTTGTCGCTGTCCAAAGTCGTGGAGCTCTG

TCAGTTCTCTGTATGGCAGCAAAATCGTCTCCTGGCATTTTAAGTTCTCACTTGCAAGAT

ATTATTGATATTGGTTTTGGGCGTTGGGCTAAGGAGGAACCGTTGCTCGCAAGAACAGCA

TGTGTTGCTCTAGAAAGATTGTCAGAGGAAGATAAGGAGAAACTCAGATGCAGTGGAAGC

AGGGTTTTTGGTGCTCTTCATAGTCTAATAACTGGCTTTGGGCTTCCAGAAAACATTTGG

TATGCTGCCGCTGACAAAGCGATTAGCACCATATACTCTATCCATCCAACCCCTGAAATC

GTTGCAGCCGATATAGTTAAGAAGTCCTTAAGTTCTGTATTTAGTTGTACTGAAAATGAG

AAAGTTCCCACTGGACTAGGTGAGGGAGTGAACTACGTTTCCACAGTGTCTGCAGCAAAG

CTTGCTAGTTTTCTCTTCATTATAAGCCATATTGCTCTGAATCAGTTGGTTTACATTGAG

TCTTGCATTAGAAAAATTCGGAAACAGAAATCAAAGAAAGAAAGATCGAGGCATGAATCT

CATGCTAGTAGTGATGGTACAAATGACGCAGCTGAGGTGCAAGGTATAAATGCTGAACTG

GGCATTGCAATATCTGAAGATGTGAAAATTGATTTGCTTTCTGAAAGAGCTGAGAAAGAG

ATTGTATCTGGCGGTTCGGTGGAAAAGAACATCATTGGTCATTGTGCACTGTTTCTCTCA

AAACTTTGTAGAAATTTTAACTTGATGCAAAAGTTTCCACAGCTGCAGGCTTCTGCAATG

CTTTCCCTTTGTAAACTAATGATTATCGATACAGACTTTTGTGAAGCAAATCTTCAACTT

CTATTCACTGTTGTGGAGAATGCACCGTCAGAAACTGTTCGGTCCAACTGCACAGTGGCT

CTTGGGGACTTGGCTGCTCGTTTTCCAAACCTCTTAGAACCCTGGACTGAAAATATGTAT

GCTCGCTTGCGAGATCCTTCTGTATCTGTTAGGAAAAATGCTGTTTTGGTGCTTTCTCAT

CTCATATTAAATGACATGATGAAGGTGAAAGGATACATAAATGAAATGGCCATTTGTATA

GAAGATAAGGATGAAAGGATCTCAAGTCTTGCCAAACTTTTCTTTCATGAGTTGTCTAAG

AAAGGTAGCAATCCGATTTATAATCTACTTCCAGATATCTTGGGCAGATTGTCAAGCCAA

AATCTTAAAGAGGAAACTTTTTGCAATATTATGCAGTTTCTAATAAATTCTATCAAGAAG

GACAAACAAATGGAAGCTCTTGTTGAAAAGCTTTGTAATCGGTTCAGTGGAGTCGCTGAT

ATTAGACAGTGGGAATATATTGCATATTGCCTTTCTCAGCTAACCTTCAGTGAGAAGGGC

CTGAAAAAACTTATCGACTCTTTCAAAACTTATGAACATGCTCTGTGTGAAGATTCTGTG

ATGGATTACTTCCGAGTCATTATCAGCAAGTGTAAAAAGTTTGCAAAACCAGAGCTTAAA

TCAATCATTGAGGAATTTGAGGAGAAGCTTAACAAGATCCACATGGAGAAAAAGGAGCAA

GAAGTGACTGCTAGAAATGCCCAGGTTCACCAACAGAAACTTGGTACTCTACAAGGCCTC

ACAACGACAAAGAATAACACAGAAGAACAAGAAACAAGTGTAAATGGTGCTGAAGATGAA

AATGGTGTGGTAATCGATGATGAAGAGGAAAAAAATGTCACGTCTCGGGAATGTCTTCCC

CAAGATATCAATACAGTATCAGAAGAAAGCGGTATTTCGAGTATTGTAGATAAAGGCGAG

GAAGATGGTTTTGAGGTTCAATCACCAAAAACTTCTCGCAGAGGTGTTCCCAAGTCCAAA

GTGAAGGGAAGCAGAAGGTTAACGCAGCAAGATGAAACGGCTCCTAGAACTGTAAGACGG

ACAGCCCGCTCAACCAGAAGAAGTTGA

>TRINITY_DN10029_c0_g1|m.53 TRINITY_DN10029_c0_g1|g.53 ORF TRINITY_DN10029_c0_g1|g.53 TRINITY_DN10029_c0_g1|m.53 type:complete len:112 (-) TRINITY_DN10029_c0_g1:46-381(-)

ATGTCAACTAATTTACCAAATGTAATCCGTCGAGTCAGTATCTTCTGGATATATCCACCA

CATGTTCTCCCATGGAATAATAAAAATAGCGATTTGTTTCAGATGGGTGAGAAAATATGG

GGGCCTTCCAGAGTCAAAAATGGATACTTGATCCAGTTCCATCATGAGTCAGCAATCTCC

TCTCGAATTAGCATGCAGGCTTTCAGTTCTGTAGAAGTCCCAATGCATATGAAATTATCT

GTGCCCCTAAGAGTTATGATACCACCTTGTACTTCAACTGCTCAGGTTCCACTGCATCTT

GCTAAATGGTTAACCGAAGAAGCCTCGACACTTTGA

>TRINITY_DN1002_c0_g1|m.56 TRINITY_DN1002_c0_g1|g.56 ORF TRINITY_DN1002_c0_g1|g.56 TRINITY_DN1002_c0_g1|m.56 type:3prime_partial len:129 (+) TRINITY_DN1002_c0_g1:2469-2852(+)

ATGTGCCGAGTAGCATCGTCACCCTCGACGAGCAGCACCCGCAGCGATCTCCTCGGCAGG

AAGCTCTCCCACCGCATCACGTGCCGCTCAGGCACCGCCGCCCCGCTCGGCCCTACGCCT

CCACCGCCCGCCGATTCGCCGCCGTCGTCCGCCTCCATCGATCGTCGGTCGAAGAGAGAA

AGAAAGAAGAGAGAGAAAAAGAAAAAAAAAGAAAGAAGAGAAGAAAAAGAGGAGAGAGAT

AGGACGAATCCGTGGCCACGCCGACCCGAGCGGGGCGAAAACGAAATATCTTCCCGTGGG

CGCCACGTCAGCAGGAGGCCTACATGGCCTTCTCGCGATTCACTAGTCTCCACTTGCCCT

CTCGCAGTGCCCCAGATTTTTTTT

>TRINITY_DN1002_c0_g1|m.54 TRINITY_DN1002_c0_g1|g.54 ORF TRINITY_DN1002_c0_g1|g.54 TRINITY_DN1002_c0_g1|m.54 type:5prime_partial len:786 (-) TRINITY_DN1002_c0_g1:497-2854(-)

AAAAAAAAAATCTGGGGCACTGCGAGAGGGCAAGTGGAGACTAGTGAATCGCGAGAAGGC

CATGTAGGCCTCCTGCTGACGTGGCGCCCACGGGAAGATATTTCGTTTTCGCCCCGCTCG

GGTCGGCGTGGCCACGGATTCGTCCTATCTCTCTCCTCTTTTTCTTCTCTTCTTTCTTTT

TTTTTCTTTTTCTCTCTCTTCTTTCTTTCTCTCTTCGACCGACGATCGATGGAGGCGGAC

GACGGCGGCGAATCGGCGGGCGGTGGAGGCGTAGGGCCGAGCGGGGCGGCGGTGCCTGAG

CGGCACGTGATGCGGTGGGAGAGCTTCCTGCCGAGGAGATCGCTGCGGGTGCTGCTCGTC

GAGGGTGACGATGCTACTCGGCACATCGTCACCGCTCTGCTGAGGAAATGCAATTACCGC

GTTGCGGGAGTGGCGGATGGGATGAAGGCGTGGGAGATGATAAAGGAGAAGAGGTACAGC

TTCGATCTGGTGCTCACCGAAGTGGCCATGCCGTCGCTGTCGGGTATCGGGCTGTTGACG

AAGATCATGGGCGCTGAAGAGTGCAAGAACATCCCTGTCATCATGATGTCTACTCATGAT

TCTGTGAACGTAGTACTTAAATGCATGCTCAAGGGTGCCGTTGATTTTCTTGTGAAGCCA

GTTAGGAAAAATGAGCTGCGGAATCTGTGGCAGCATGTTTGGAGAAGACAATGTTCAAAT

AGATATTTGAATGCATTGGACAACAATACTGCTAGCAATCAAATTAGTGCGAATGTCAGT

GGCAGATCCAAGACCGGGGAAAACAGTGATGAAGGAAGCGATGCTCAGACTACAGGCAGT

AAGGGAGATACAGAAATTGAAAGCATACAGAAGCATGAAGAACCTCTTCATGTCGAAGAT

GGGTGTACCAGTGAGGGGGAAGCCAACCTTGACTCTCAGAAAGATACAGTAATTAGGGCA

AGCATATCAAAGATGGACAACGTTCGGAATACAAATAAGACTGTGGGCGTTAGGGTCGAT

GTAGGTTTACCTATTCAAGAAATTTCCGGAGCTAATGTAACTCAAGAACAAGACCGACCT

TACAATATTTCCTCGTATAGGGAGGAAAAGTTGGAGCTTGTGAGATATAGTGAGGGCAAA

ACAAATGACCCTAAACCATGTCAGAACAATGATCCAAATGAGTCTTTGCAGAACATGATT

GTATTCGTTGAGCCAAAATCTAACAAACAATGTAGTTACACTGTTTTGAAAAAGGATGCT

CTCTTGGAAGATGGATTACGTGAAATTGCAAAATCTTCTTTTGCAAATGATGCATCGAAT

GTTGGCGCTTCACCTTTATGCGAGCTTTCCTTGAGAAGACCACAGCCTAATGGTTGCGTG

CAGGCTGAGTTAAAAGAGAAGCATGTGTTGAAACATTCTGTGGCCTCGGCCTTTTCGAGG

TATGGTGAGAAAAAGATTCATTCTTCATTTCAGAAGTCAGTTTCTTCTGATTTATGTACA

AGAACACGGGAAGGTGTGGAGAAATGCCACTCCCTTGTTAGCGGTCATGGCATTGATAAT

GAAAATGACTCACCCTTCTCTCGTACGGAGAAGACACTGCCATCCCCTCAGGGCAATGAA

GGCAAAACTACAGTGTATTTTCAGGTCTCGTCCACTAACAAGGAGAAAGTTGGCTGTTCT

TCTCAACCATTAGAGAAGGGCCGTCCTGTTCACTCTTCCACTGTGGAGGATACTGCTCTC

CATCGCCCCCAATATGGTTTCATACCAGTGCCAATTCCTGTCGGGGTAATACCTTACCAG

AGCATATGTGCAGGATATGGAGGTATGCTACAACCAGTATTTTATCCAGAGATTTACTTT

GCACCACAGGTTTCAGCTACAGTTGAGAAACCTGCAGCCCTGATTCCTTCTGAGCATCCT

GTTCATCACAACCATCATCTGATTGATCGTTCCTCCTCTTTGGACTTCCACCACCATGAA

GCAAGTGATAATCCTCATCACTGGAGACGAACAATTGACATATCAGAACCAGGTGAGTCA

AGAGAGTTGTGCAACCACCTGGACCAAGCCTATCAAAATGGTAGTTGTATTCATGATTGT

CTAAAAGGCAGTGGAAGCAATGGTAGTGGTGAAACTGCTGATGAAGCTGGAAATACTGGA

AATGCTCTGGATTGTGATCGTTCTCGACGTGAAGCTGCCTTGATTAAATTCCGCTTGAAG

CGAAAAGACAGATGTTTTGAGAAGAAGGTTAGATACCATAGTAGGAAAAAGCTTGCGGAG

CAGCGTCCAAGGATAAAAGGCCAATTTGTGAGGCAAAAAATTCCAGAGTCCATAACAACA

GGAGAAGGGGACAACTGA

>TRINITY_DN10031_c0_g1|m.57 TRINITY_DN10031_c0_g1|g.57 ORF TRINITY_DN10031_c0_g1|g.57 TRINITY_DN10031_c0_g1|m.57 type:complete len:299 (+) TRINITY_DN10031_c0_g1:140-1036(+)

ATGTCCGAAACCTTGACTACCGCTACTACTACGAAAAAGACCCTCGCCAACCCCGAAGTT

CTCGAGCATATCTTCCAAAGATTGGAAGCCGAGAGTGCAGAGCGCGCTCGTCGTGAGGAA

GAACTCGAGCGTTCCCAGAGAAAACAAGAGCTTTCATTACGTCAAGAAGCATCTGACGTC

GTTCCGGAGACTGTCCCCGCATTTTCCAGCCAGAACACCTTAGACTCTGCTACAACAAAA

ATGGTCAAGGAGCGCCGGAGAGGCAGTATCTCCATTTCGCGCTTTGGCTACCCACCAGAG

TGCGCCAGTATCCCCACTACTCCTCTTACTGAGTCACAGGCGCAGTCGAATCGCCCATCG

CGTTCGCCATCCGTTATCCTCACGAAATCGACATTCTACCAACTGGACGCTGTCCGTTAC

CACGGCCGTCAACGGCCATTAGCCAGCGCTAGCCAGGATTCATTTGCCTCGCTCAACGAT

GATCCAAACGCTCATTTCGAGGACGAAATGGTGACCGTGCAGAGTATCACTATTGCTCCA

AAACAGTCGCTCTCGAAAGCCATCTCGCGGCGTCTTTCTCGCGCGCGTGACATCGCTCTG

CCCCCAGCAGTTACTGGCCAGCTGGTGATCGGGGTCGCAGTGGAAGAGGCGACGATCGAG

CATCATGCGGAGGACCCAGAAGTCCTGCACAGCCCGGTTCTCACCAGCACTGTCATCACG

GCTGGCAACGGTACTGGCACCCTCAAGACCAAGAAGTCTTTCTCGAGACTTATCTCGGGT

AGTGACGACGACTCCAGCTCGACAAAGAGCACAACCGCCTGGATTGAAAAGGCGAGGGAT

ATCACCACCAAGTTCAAGCGGCGAAGTATCGCTGCGTTGAACTCCGCTATGAATTAG

>TRINITY_DN100328_c0_g1|m.59 TRINITY_DN100328_c0_g1|g.59 ORF TRINITY_DN100328_c0_g1|g.59 TRINITY_DN100328_c0_g1|m.59 type:internal len:105 (+) TRINITY_DN100328_c0_g1:1-312(+)

CAACCATCAACCATGTCCTACACTGAAGCCCAGTTCAACAAGGCCGTTGAGATTGTGCAG

GGTATGCCCAAAGATGGCCCTGTCAAGCCCACTCAAGATCAGCAGCTCGAGTTCTACAAG

TACTACAAACAAGCCACGATCGGTGATGTCAACACCGCTCGGCCAGGAATGCTCGACTTT

GTAGGAAAGGCCAAATGGGATGCTTGGAACGGCGCCAAGGGGACCTCGAAGGAGGAGTGC

TGGACAGCATATGTTAAAAAGCTGCTTGAGATCCTCGAGCCTTCGGAAGACGAGGATGCG

AAGAAGTGGGTT

>TRINITY_DN10032_c0_g1|m.61 TRINITY_DN10032_c0_g1|g.61 ORF TRINITY_DN10032_c0_g1|g.61 TRINITY_DN10032_c0_g1|m.61 type:5prime_partial len:122 (+) TRINITY_DN10032_c0_g1:3-368(+)

ACGCTTCACTTGCTGGCACTCAGAATGTCTGCTGCTCACGACGACCCCCGCGACGCTTCC

GCCGAAACAAAGCTCACATTTCCAGAGTCATGGCAGGCTGACAGCCCTGATGGCCTCAAT

GCGTCTGTCTTGTTCATTTCTGGGCTGATCATGGTCACCCGGAATCGTTACCTCTCGTGG

CCAAACCTTATGCTCACGCTTAATAGTATGTTCAACCAGCACCCACTGCGAACAAAGGAG

GGAGGAAACAGTGGCTTCAGTGCATTGACGATTGCTGTCTCTGCCCTAATTGCGTCATAC

CTACCCATGATCATGATTAACCCTCAGAGGACGCCCACCCAGGCACCTTTGCCTGTCCCG

AACTAG

>TRINITY_DN10033_c0_g1|m.62 TRINITY_DN10033_c0_g1|g.62 ORF TRINITY_DN10033_c0_g1|g.62 TRINITY_DN10033_c0_g1|m.62 type:internal len:298 (+) TRINITY_DN10033_c0_g1:1-891(+)

TCATTAGTCCAGATAGAAAGAACCTCCGTTGCTGTCACCAGTGATCTCACTCATCGATCC

GCCATTGCAGATTGCAGTCATCTGCTGGAACTGTCACACGACCGTCTTCTAGACTCGACC

ACCGCCCTCACAGCAGGGGCCCACACCGACGCCCGAACGTGGCTCAGTGCCGTGCTCACC

AACTACGCCACATGCCTCGATGGGCTTACAGGTTCATCTGATCCCCTGCCAATGATCAAG

TCCCATTTAAATTCCTTGTCGGCGTTGGCAAGTACTTCGCTTGCTGTGCTCAACGCAGTT

TCACCTTATAATAATGGTGATCACGCAATTGAAACCATCCTGAAGCTTCCATCATGGGTT

ACGTTCAAGGACAGGAAGCTTCTGCAAGCTTCTTTGAGAGATGGAATCGAAGCAAATGTT

ACTGTGGCAACTGATGGGAGTGGAGACTTCAGCACAGTGCAAGATGCAGTGAATTCTGCA

CCGGACAAGGGCAGTACTAGATATGTGATATACGTGAGGAACGGAACATATGCAGAAAAT

GTGGTCGTGGGAAAGAAAAAGACTAATGTTATGATAGTTGGTGATGGCTTGAATTCTACT

GTGATAACGGGTAGCCTCAACGTCATTGATGGATCTACCACGTTTAACTCAGCTACACTA

GCTGCTGTGGGGGATGGATTTATACTGCAGGACATGTGCGTTGAGAACACGGCAGGACCC

GAGAAGCACCAAGCGGTTGCGCTTCGTGTGGGAGCTGATAGATCGGTCATAAACCGATGC

CAGCTGAATGGGTACCAGGACACCCTCTACGCTCACTCTCTCAGACAATTTTATCGCGAC

AGCTTGGTGTCAGGCACGGTGGACTTCATCTTTGGCAATGCAGCAGTGGTG

>TRINITY_DN10033_c0_g2|m.64 TRINITY_DN10033_c0_g2|g.64 ORF TRINITY_DN10033_c0_g2|g.64 TRINITY_DN10033_c0_g2|m.64 type:3prime_partial len:169 (+) TRINITY_DN10033_c0_g2:387-890(+)

ATGGCATCAACTCAAGAACCTCTCATTCCTTCTCCTATCAACAACACCACCATTTTCCCC

TGTAAAGCCTTCATAATCACTCTATCCGTTGCTTCTCTCATTTGCTTCGCCACTATCATC

ACTCTTCAACTGCCATCCATCCACCACTCCAATTCACTCCAACCCTCCCATCTGTGCACC

CAATCCCCAAATCCTCTCTCGTGCCACGCCATCGTCACCAGCGCCATCGCCCAATCTCAA

ATCTCTGATCCACTTCCGATCCAAATCCTGCAAAACCTCATCGACCAGTCATTAGTCCAG

ATAGAAAGAACCTCCGTTGCTGTCACCAGTGATCTCACTCATCGATCCGCCATTGCAGAT

TGCAGTCATCTGCTGGAACTGTCACACGACCGTCTTCTAGACTCGACCACCGCCCTCACA

GCAGGGGCCCACACCGACGCCCGAACGTGGCTCAGTGCCGTGCTCACCAACTACGCCACA

TGCCTCGATGGGCTTACAGGTTCA

>TRINITY_DN10034_c0_g1|m.65 TRINITY_DN10034_c0_g1|g.65 ORF TRINITY_DN10034_c0_g1|g.65 TRINITY_DN10034_c0_g1|m.65 type:complete len:981 (+) TRINITY_DN10034_c0_g1:115-3057(+)

ATGGCGACTCCGGCGAAGCCATGGAGGGCCGAGTACGCGAAGTCGGGGCGATCGTCGTGC

AAGGCCTGCAAGTCTCCCATCGGCAAGGACCAGTTCCGCCTCGGCAAGATCGTCCAAGCC

TCCAATTTCGATGGTCTCATGCCGATGTGGAATCATGCTGGTTGTATCCTGAAGAAGGCA

AAGCAGATCAAATCCTTGGATGACGTTGAAGGCATAGATTTGCTACGTTGGGAGGACCAA

GAAAAGATACAGAAATATGTTGATGGTGGCTCTGTGAATACTACTGCTGCTGCTAGTGTT

ACTGGTGACAAGGATTGTGTCGTTGAAGTTTCTCAAACTTCCCGTGCTACTTGTAAGCGC

TGCAGTGAGAAGATTATGAAAGGAACGGTTCGTGTTTCTACCAAGCCAGAGGGACAGGGT

GCTAGGGGCGTAGCTTGGCACCACATCCACTGTTTTATACAGATGTCCCCATCCATTAAT

ATAGAGAAAGTGTCAGGTTGGGACAACCTTCCATCTGAAGAAAAAGCAGCTGTTTCTTCT

CTAGCTAAAAGGGACACATCTACTACTAAAAAAGGGGGTGCAGTAACGGAAGATAACCTT

GCTGCTTCTGAGCAGACATCTAAAGGCATTAAACGTAAAAATGATGGGAATAATGAGCAA

AAATCTAAAATCCCAAAATCAGATGGAAATGTATCCGCTGGCGCTGTTGATTTGGAAAGC

AAGCTGAAGGAGCAAACTAATGCATTGTGGGAGATAAAGGATGAGCTTAAGAATCACGTA

ACCGCTACAGAGCTCCGTGGAATGCTTGAAGCTAATGGTCAAGATTCAGCAGGATCAGAA

AATGATCTACGTGAACGTTGTGCTGATGGCATGCTGTTTGGAGCACTGGGGAAATGCCCT

ATTTGTTCTGGTTCTCTTCACTACTCTGAAGGCCAATATCGTTGCCATGGTTATGCATCT

GCTTGGAGCAAGTGTTCGTACTCAACTACTGAACCTGTACGAATCAAGCAGAAGTGGAAA

ATTCCAGAGGGAGTAAACAGTGAATATATTCTACATTGGTCAAAGTCTCAAAAGACGAAA

AAGCCTGATCGAATCCTTCCTCCAGTGTCTACTAAATCTTTCGTTAGTCAAACTTCCCCT

ACCAAGTCTCAGGCATCTTATAGTGAAAGATTGGAGGACCTGAAAATTGCTATTGCTGGC

GAACTTAAAGTAAACTTGGACGGCTGGAAGAAGAAGCTTGTGGAGGCTGGGGGAAAGGTC

CATAACAAGATCACTAAAGATACTAGCTGTCTGGTTTTGGGCGGTGCGGTAGCCGATTCG

GATGCAGACATCAGAAAAGCCAGGAGGATGAAAATTCCTGTTGTGAGGGGTGAATACCTG

GGTGAATGTATTAAAATGAAGAAGAAGCTTCCATTCGATCTATATAAAGTCGAAGCAGCC

GGTGAGACTTCAAAATATGGTACGGTAACTGTGAAAGTTAAAGGTCGTAGTGCAGTGCAT

GAGGCCTCTGGTTTACAAGACACTGGCCACATTCTTGAGGATGGAAAAAGTATCTACAAT

ACAACTTTAAATATGTCTGATCTTGTGACTGGTGTCAACAGTTATTATATTCTTCAGATC

ATCCAAGAGGATAAAGGGTCAGCTTGTTATGTTTTCCGTAAGTGGGGACGTGTCGGAAAT

GATAAAATTGGAAGTTCTAAATTGGAAGAAATGTCGAAATATGATGCAATTGAAGAATTT

AAACGTTTATTTCTTGAAAAGACTGGTAATCCTTGGGAAGCCTGGGAGCAGAAGCAGAAC

TTCCAGAAGCATCCAGGCCGATTTTTTCCACTGGATATTGACTATGGTGTTAAACAAGCT

CCAAAAAAGAAAGATCCTACAAAGACAAAGAGCCTACTTGCTCCTCAGCTAACAGAGCTA

ATGCAGATGCTATTTAATGTTGAGACTTACAGGGCTGCTATGCTGGAATTTGAAATTAAT

TTGTCCGAGATGCCACTTGGGAAGTTAAGCAAGGCGAACATCCAGAAGGGATTTGAAGTA

TTAACGGAGATACAAAATGTATTAAATAATACTAGTAAGAATGATCCTGCTGTTAGAGAG

AGCTTGATTGTCGATGCCAGCAATCGCTTTTTCACTCTTATCCCTTCAATTCATCCTCAT

GTCATAAGGGATGAAGATGATTTCAAAGCAAAGGTGAAAATGCTAGATGCTCTTCAAGAT

ATTGAAATTGCTTCTAGACTGGTTGGTTTTGATAGCGAAGATGATGAATCTCTTGATGAC

AAGTATAAGAAGCTCCACTGTGATATTGCTCCACTACCTCATGATAGTGAAGATTATAGA

CTGATTGAGAAGTATCTTCATAGTACTCATGCTCCGACCCATAAGGATTGGAGCCTCGAA

CTGGAAGAAGTTTTTGCTCTTGACCGAGAAGGAGAATTTGACGAGTATGCACCATACCGA

GATAAACTCAAGAATAAGATGCTGTTATGGCATGGTTCTCGGTTGACTAACTTTGTTGGT

ATTCTTAGCCAAGGTCTAAGAATAGCACCCCCTGAAGCTCCAGCTACTGGTTACATGTTT

GGCAAGGGAATTTACTTTGCTGACCTTGTTAGCAAGAGTGCACAGTACTGCTATGTTGAC

AGAAAAGATCCAGTAGGTCTCATGCTCCTTAGTGAGGTTGCTTTGGGGGAGATTCATGAG

CTTAAAAAGGCCACTTATATGGATAAGCCGCCTAGGGGTAAGCATTCAACCAAGGGACTC

GGAAAGACTGTACCTCTGAAGTCCGAGTTTGAGACGTGGAGAGATGTAGTTGTGCCTTGT

GGGAAACCTGTGTCCTCATCTGTTAGAGCATCCGAGCTCCTATACAACGAGTACATTGTA

TATGATACAGCTCAGGTTAAGATGCAGTTCCTGTTGAAGGTGAGGTTTAACCACAAGAGG

TGA

>TRINITY_DN10035_c0_g1|m.68 TRINITY_DN10035_c0_g1|g.68 ORF TRINITY_DN10035_c0_g1|g.68 TRINITY_DN10035_c0_g1|m.68 type:internal len:102 (-) TRINITY_DN10035_c0_g1:1-303(-)

GAGGGGGTGGGAGTGAACTGGGGGACAATGGCGACCCACCAGCTGCCGCCGGGGACAGTT

GTGCAGATGCTGCAGGACAATGGGATCATGAAGGTGAAGCTGTTTGATGCTGATCCTAAC

ACGATGAGCGCGCTCGCGGGGACGAATATTGAAGTTATGGTTGCGATTCCGAACGATCAG

CTCGCGACCATGAACGATTATGACGAGGCCAAGAAGTGGGTAGATCGAAATGTCACCAGA

TATAACTTCAAAGGAGGTGTTAACATCAAATATGTAGCAGTCGGAAATGAGCCCTTCCTA

TCA

>TRINITY_DN1003_c0_g1|m.69 TRINITY_DN1003_c0_g1|g.69 ORF TRINITY_DN1003_c0_g1|g.69 TRINITY_DN1003_c0_g1|m.69 type:5prime_partial len:231 (+) TRINITY_DN1003_c0_g1:3-695(+)

AAAAAGTTCGAATTTCTCACAAAACGAAACGAGAGTGGCGGAGAGATCTCTCTATCTCTC

TCGTTGCACGCCAAATTCGTCCTCTCCTCCTCCCCCATGGCCGCTCTCCTCACCTCCGCC

GTCCCCGAGGTTGGGTTGCGGTTGCTGCTTTATCCTCTTGGTGCTAACATAGTGACTAGA

ACAGCTTGTTGCACAGTGGGGATTGTTTTTCCTGTATACTCTACTTTCAAGGCAATCGAG

AAGAAGGACCGAAGTGAACAAGAAAAATGGCTTTTATATTGGGCAGCATATGGATCTTTT

AGCCTTGTGGAAATGTTCTCTGACAAAATCCTTTCCTGGTTCCCACTATACTACCATATG

AAATTTGCTTTTCTTGTCTGGCTCCAACTTCCATCTGGTAATGGTGCTAGGCATTTATAT

GCAAGGCATTTGCGCCCCTTTCTGTTGAGACATCAAGACAGACTTGATCAACTACACGGT

TTTGTCACAAGAGAAATTACAAAGTTTATCATCACTCATCAAGGAGAAATTGAATTCCTA

AAAGCCGTGCTTAAAAGGTGCGCAAATACAGCAAATCAGATGCTGAAGGAAGTCAATCAA

CCAGTACAACCTGGAGGTCAGAATATGATCGAAGGCCCAAATGGATCTACACGACCACGC

ATTCAGGATTCTGGTTCTGATTCTGATAATTGA

>TRINITY_DN100407_c0_g1|m.70 TRINITY_DN100407_c0_g1|g.70 ORF TRINITY_DN100407_c0_g1|g.70 TRINITY_DN100407_c0_g1|m.70 type:complete len:994 (-) TRINITY_DN100407_c0_g1:497-3478(-)

ATGGCGCCACCACCGATCTCCACGCGAGCAAGGAATCGGGCGCAGAAGCGCAGGGCGGGC

CGGCGGTTCTTCTGCGGCGCCATCTCCTTCTTCGTCTTAGCCATCATCGTCATCTTCGTC

TACACGCTCAACAAGATCCCCAACGTCTCCCGGGACAAGGACTGGCCGGAGCCCTCGACG

GTGAAGTGGACTCGGCCGCCGGGGATGGTGCTGAGGTTCCGGCCGGTGGATCTGATGCGT

AGGCTGGAGGCGCAGGGGAGGAGGCTGGATCGGTTAAGATCCGAGAGGAGAGTCGGGATT

CGGCCGCCAAGGCTCGCACTGGTTGTGGGGAACATGAATAAAGATTCGCAATCCTTGATG

CTCTTAACAGTAGTGAAAGGTCTTAGAGAGCTGGGATACGTATTCACGGTTTATACATTA

GAGGATGGAGAAACTGACTCTTCATGGAAGCATGTTGGTTGTCAAGTATCAGTTTTGAGT

TCTGACAGTTCTAGTACCGTTGATTGGTCAAACTATGAAGGCATTATTCTAAGCTCTCTT

GAAGCCAAAAAAGTCATTACAAGTTTCGTGGAGCAACCTTTTGATTCTATACCAGTTGTA

TGGCTTATTCAAGAAGATATCCTTGGGAGGCACCTTCCTTCATATGAATCCTCGGGTTGG

AATGATCTTGTTACCATGTGGAGAAGCGATTTTAGTAGAGCTAATGTTGTGGTGTTTCCA

GATTTATCCCTTCCAATGATTTATAATTCACTTGATATTGGAAACTTCTATGTGATCCCT

GGATCTCCAGTCAATATCTGGGAAGCTGAGAGTTACTCTACCTCCCATTCTCGAAGCCAA

TTAAGGAAAGATGATGAATTCGATGATGATGATTTTATAATGTTACTGATCGGGAGCTCC

TTTTTCTATGATGAACTTCCAAATGAATATAAAGCTATTATGAACGCCTTAATTCCTGAG

ATTAAGAAAAGTACAAGAATTGAAGGGCTGGGCGGAACATTTAAGTTTATTTTTTTACGT

GGGGATACCACACTTGTCCATGAGCCTTCTTTTCAGGCACTTATTTCACATATGGGGCTC

CCATCTGGTTCTGTAAGGTACTATTCAGCAAGTAGTGATGTGAACAGGGTGCTTTTGATG

GCTGATTTAGTTCTCTACGGCTCCTTTATGGAGGAACAGACTTTTCCGCCACTATTGATG

CGAGCAATGTCATTTGAAATTCCCATCATCGTTCCCAGTTTGGATATCATAACAAAATAT

GTTGAGAATCAAGTCCATGGGATGATTTTTCATCCACATGATCTGGGCACATTGGCGAAA

GCTTTCTCACTTCTGATAAAAGATAAGAAGCTGTCTAAATTAGCTTATTCAGTTGCTTCT

TCTGGGAAATTACTCTCCAAGAACATGTTAGCATCGGAGTGCATTAGGGAGTATGCGAAG

CTTCTTGAAAACTTACTCCAGTTTCCTTCTGAATCAATGCTCCCTCTGCCCATTTCTCAT

ATTAAGCAAAATACCTGGGCGTGGGACTCGTTTCAAAAGGAAAATAAACAAACCAGTGTT

TCTGGCCAACAAGAGAGTTTCCAAAACAATAGCACACCGCGAAGAAGCAGCATTGTTGTC

TTTCTTGAGGATCAGGCAACTGGTAAATTTCAGGTTCAGAATACCAGCCTGATCGTTAAC

GAGTCTTCTGCAGAAGATTTTCCTACCCAGTTAGATTGGGATATTCTTGCCGAAATGGAA

ATTCTGGAAGATTCTGATAGACAAGAAAGGGAAGAGATTGCAGAGAGGATGCCAAGAGAT

TTGGGACATTGGGAAACTGTATACAATAAAGCACGGAAAGCTGACAAAAATACAAAATTT

GAAGAACACGAGCGTGATGAAGCTGAATTGGAAAAGATTGGACTTCAGTTGTGTATCTAT

CAGGTTTACAGCGGACAAGGAGCATGGCCATTTTTACAGCATGGTTCTCTATACCGTGGG

ATAAGTCTCTCCAAGAGAGCTCAAAGACCAAGATCAGATGATGTTGATGCAGTTGGTCGC

CTTCCACTCTTGAATAATGCATATGATAGAGATCTTCTTTGCGAGTTCGGTGCCATGTTT

TCAATTGCAAATAAGGTGGACAGCATTCACAATATGCCTTGGATTGGTTTCCAGTCATGG

CGTGCTGCTGGAAGGAAGGTCTCGCTGTCAATTAGTGCTGAAGAAATTTTGGAACAAATT

GCAATTGAAAAATCTGGAGGAGATGTAGTTTACTACTGGGCACCAATGAAGATGGACTTG

AAGGATGAGGGAAAAGATGAGAAGGTTGACTTTTGGTCGATGTGTGACATCTTAAATGCT

GGACAGTGCAGGACTGTGTTTGAGGATGCATTCAGATTAATGTATGGTTTGCCTATGGAG

ATGGAAGCTTTACCACCAATGCCTGATGATGGTGACCGATGGTCTACTCTCCACAGTTGG

GTGATGCCTACCCCCTCCTTCCTAGAATTCATGATGTTCTCAAGAATGTTTGTGGACTCG

ATGGATAGCCTAAACCATAAAAACAGCACCCCTGCTTCATGTGTTCTTGGATCCTCAGAA

CTGGAGAAAAAAAACTGCTACTGCCGAGTGCTGGAAGTCCTGGTCAATGTGTGGGCTTAT

CACAGTGCCAGAAAGCTGGTTTACATTGATCCAAGCACTGGGAAGATGACTGAGCAGCAT

CCTATTGACCAACGCACTGGGAAAATGTGGGTGAAGTACTTTGACATCACATTGTTGAAG

AGTATGGATGAGGATTTAGCTGAGGAAGCAGATGACGGCATTCATCCAACAGATAGATGG

CTGTGGCCTTTTACAGGAGAAGTACATTGGTCAGGGATATTCGATAAGGAGAGGGAGGAC

AAATATAGGGGTAAAATGGATAAGAAGAGAAAGAATAAGGAGAAGTTATTAGATAGGCAG

AAGCATGGATACAAACAGAAATCTTTAGGAGGAGCGCGTTAG

>TRINITY_DN10041_c0_g1|m.73 TRINITY_DN10041_c0_g1|g.73 ORF TRINITY_DN10041_c0_g1|g.73 TRINITY_DN10041_c0_g1|m.73 type:5prime_partial len:104 (-) TRINITY_DN10041_c0_g1:340-651(-)

GACCTGCAATCTGCTAGATCTTATTATTCCCGTGCTTTGGATGTTCGTCGCAATGCAGTC

AAAGAGCTTGCGAATGCCTCTTCACAGGTAGTTGATCTGGCAGTTTCTCTAGCCAAAGTT

GCTGATTTGGATAGGAATCTCGGTATTGAGGGTGAAGCAATTAGTGGATTTCGGGAAGCT

ATAGAACACCTAGAATCCTTGAAGTTGGATTCCAGTGAAACCAGTCTAGAACAGCGGCGC

CATTCTGTGCTGGAGTTCCTGCACGACCGACTTGGAGAAAAACCAGAGCATGCTCCTGTT

TCTACTGTCTGA

>TRINITY_DN10044_c0_g1|m.74 TRINITY_DN10044_c0_g1|g.74 ORF TRINITY_DN10044_c0_g1|g.74 TRINITY_DN10044_c0_g1|m.74 type:complete len:412 (+) TRINITY_DN10044_c0_g1:112-1347(+)

ATGTCGTCGCAGGTCGACCCCGAGAAGTATGCTGCGCTAGTCGTAGCCAAGTTTCTTGAG

ATGAAGTACGCCAATTCATGCGCGGTGGCAGCAACAATCCTTCTATACTACGACTTCCTC

ATCACCTTCTCCGACGAGGTCCGCTGCATCTGGAAACGCAAGTTTACCGGTGCCTCGCTC

ATCTTCTTCATCAACCGATATGTCACGCTCGTCTATAAGACGTTCATGATCATCCAGATG

TTGCCCTGGGACCATGTCAATCCTGATCGTGCTGACCACATATGCGTAGGAATCTTGAGA

ACTTGTGAGATATTCACACTGACCTTACAGCTGGTTACTGCCGCATTCACCTCACTCCGG

ATGTACGCCATCTGGTCCAAAGACATCAAAATATTTAGCCTCGTTCTTTTCTTGGGGCTG

ATTCCTATTGGAGTGAACATCTATTATTACACGAGATTAACCATCATAGCTACCCCTCCT

CCTTTCACAGGGTGTGGCGAGCTTGTCGACCTGGGTTCGCCTACGGGCCCGCTTACACTG

AGCATCGTAAACAGAGTCTTCGCCATCGTCTTTGACGCTCTCATCCTCATCCTCACCTGG

ATCCGAACTGCGGACATCAGGAAGCTATTCATGCAGCTGAACATCAAGACGTCTTTAGCG

ATGATGCTTCTGAGGGATGGCACTATGTATTTTGTGGTGCTGTTGATTTTGAACGTGATC

AACCTCATTGCTATCAAGTACAAGGCATTCGGCTCTCTTCCTTCAATCACAGAAACTATA

ACCTCCATCTTCGTCTCCCGTTTCATCATCAATCTCCGCTCCATCTATCTCTCCGACTCC

CGCATCTCCACCATAGGCCCTGCAAACGGTTTACACGCCTCCACCTTCCACGCCTCCAAA

ATCTCCGACATCCGCTTCGCAGGCGCCTCCAATCTCGTCGGGAACCTCGGTGCTCCTCTG

GACATCGGCTCATTCGACTCCCCACCTGAACTTAACCGATCTTCTACTTTGTACTCGCCG

AGGAGCGAGAGTAGGAGAAGTGGTGGGTCCGTTTATGGTGAGTTGGAGGTGGACGCGGAC

GCGGATAGGCCCGGGTTGAGCGAGGATCCGTTGTTAGCGGGTATGATGGACGCGGTGGAG

AGAGAGGAGGAGAGGAGGGAGAGGGAGGAGGTGGGGAGGGTGTATGAGGATAGTGTATAT

GCAGGGAGTGAGCGGTCGTCAACGTACTCTGTTTAG

>TRINITY_DN10045_c0_g1|m.77 TRINITY_DN10045_c0_g1|g.77 ORF TRINITY_DN10045_c0_g1|g.77 TRINITY_DN10045_c0_g1|m.77 type:complete len:137 (-) TRINITY_DN10045_c0_g1:953-1363(-)

ATGATGAAAGTCTCCCTGCGGAGCAAATGTTATGTTCACAGCAAGAATGCAGCGGTTGCT

GACAGAATACCAGCAAACGCCAAGGAGCTCAACGATAATGATGGAGCGGAGCTAGGGCTT

GATCCACTCCCTGTTGGCCCTGGTGACACAGTGGGAACCTGGCCGATGCCAGGGTTGCTC

GAGGACGGCCCTGATGTTGAACTGCTTGGTGTTGTTGGAGCTGCAGGAACGGCAGGTGTG

GAGCCAGGAGGGGAGGCAGCAGGGGCCATACCTCTGCTGCTACTGGGCGCTACGGGAGCC

GCAGGACCACCAGCTGCGGGGGCAGGTGCAGGGGCAGAAGAGGACGAAGCGGGCACACTG

ATGGCGAGCTTCTGCCCGATGGAACAGTGGCCAGTGACGGCACAGATGTAG

>TRINITY_DN10045_c0_g1|m.76 TRINITY_DN10045_c0_g1|g.76 ORF TRINITY_DN10045_c0_g1|g.76 TRINITY_DN10045_c0_g1|m.76 type:complete len:380 (+) TRINITY_DN10045_c0_g1:189-1328(+)

ATGCAAAGAGAGTTCAAGAGGGGTGACCTGAAGAACACAACCAGGACAATCGGAATGACG

ATGAGGATGACGGCCGGAAAATGGTTGATAATCGCTTTGGCCGTAGTTGCGGCGACGACG

GAGGCGGCCACGAGCCATACTGTCGGAGGAAGCGGCACGGGATGGACCATCCCCACGAAC

ACTTCCCTTTACTCCCAGTGGGCGTCATCTCAGAAGTTTGCCGTTGGCGACACCCTGGTG

TTCAATTTCGCGACGGGGCAACATAACGTGGTTGAGGTGCCGAAGGCGAGCTATGATGCG

TGCTCGACAAATAACCGGATAGGATCCACCTTTACCACTGGTCCGGCCAGCGTCACCATA

ACCACCCCGGGCACCCACTACTACATCTGTGGCGTCACCGGTCACTGTTCCATCGGCCAG

AAGCTCGCCATCAGCGTGCCTGCTGCTGACTCCTCGTCTCCTGCCCCTGCGCCTGCCCCG

AATGCTGTCAGTCCTGTGAAACCGAAAAAAGCAGTTGTGAACTATGTTGTCGGAGGCAGC

ACCGGATGGACCATCCCCACGAGCAATTCCTTCTACTCCCAGTGGGCTTCCTCTCAGAAG

TTTGCCGTCGGCGACACCCTATTGTTCAATTTCACCACCGGGCAACATGATGTGGTTGAG

GTGCCAAACGCAAGCTATGATTCGTGCTCGACGAAGAACCAGATAGGTTCCACCATGACC

ACTGGCCCGGCCACCGTCACCATAACCACCCCAGGCAACCACTACTACATCTGTGCCGTC

ACTGGCCACTGTTCCATCGGGCAGAAGCTCGCCATCAGTGTGCCCGCTTCGTCCTCTTCT

GCCCCTGCACCTGCCCCCGCAGCTGGTGGTCCTGCGGCTCCCGTAGCGCCCAGTAGCAGC

AGAGGTATGGCCCCTGCTGCCTCCCCTCCTGGCTCCACACCTGCCGTTCCTGCAGCTCCA

ACAACACCAAGCAGTTCAACATCAGGGCCGTCCTCGAGCAACCCTGGCATCGGCCAGGTT

CCCACTGTGTCACCAGGGCCAACAGGGAGTGGATCAAGCCCTAGCTCCGCTCCATCATTA

TCGTTGAGCTCCTTGGCGTTTGCTGGTATTCTGTCAGCAACCGCTGCATTCTTGCTGTGA

>TRINITY_DN10047_c0_g1|m.79 TRINITY_DN10047_c0_g1|g.79 ORF TRINITY_DN10047_c0_g1|g.79 TRINITY_DN10047_c0_g1|m.79 type:complete len:575 (+) TRINITY_DN10047_c0_g1:98-1822(+)

ATGAGGCTCGGTGTGCGGCTTTCCAGGCTGCCTTACTCGCTGTTGGTCACCTCTCTGGCC

ATTGCTGCAGTCGGTGTACCAGTTGGCTCTGTGGAACTTCAAGTTCTCACACCAGAAAAC

TTTGAGAATACTATATCTAATGGAGTGTGGTTCATTGAGCACTTCTCTCCCTACTGTGGA

CATTGCCGTCGTTTTGCACCAACATGGGAGCAACTCGTTGAGGAGAACCAGAAGCAAGAA

AATCCTGGAATTCATCTTGCTCAAGTGAATTGCGCGGTCCATGGTGACCTATGTCAGAAA

AACAAGGTGGAGGGGTATCCTCAAATGAATCTGTATAAGGATGGAAAATATGTCGACACG

TTCCGAAAGTCTCGCGAGATAGAGATCCTACGGGAATATCTCGCCGAGCACGCAGAGCCT

AAACCAGTTCCCTCCCAGCAACAAACACATTCTGTAGAAGAAGTCCCAGCAAAAACCTAC

AATCCCACTGGAGCAGTGCTACCCCTTGATGAGAAAACTTTTGACGATGCCATAAAAGCA

GGTAACGTATTTGTGAAGTTCTTCGCGCCATGGTGCGGTCATTGCAAGAAGCTGGCGCCG

ACATGGGTCGAACTAGCGAACATCATGCGAGGGAAAATGACCATCGCTGAAGTGGACTGT

GAAGCGCATGGTTCCATCTGCAGAAGTCAGGGTATCAGTGGATATCCGATGCTCTTCTAC

TTTGGGGAGCAAGGCGGTCACAAGACTGAGTACACTGGCGGCCGAAAGCTCGACCAGCTC

AAGGCTTTTTCAAACAAGGTATCTGGGCCAGCTGTTCAAGAACTGGAATATGAGGAGTTG

GAGAGGGAGGTTACCGCTCACCCTGTCGTATATATGCTCCTCCACCCATCCTCCGACACC

AAATCGCTTCACCAAGTCACAGAAGCGTCTCACATCCTGTTCGGTTCCCCGCCCGTCTAT

GTCTCGTCATCTCAATCGTTCTACACTCGTTTTTCACTCGATCAATCAACTGCACACGTC

ATCGTATTTAAAGATCGCAATTATGCTATACCTGTTGACGTTTATTCTCTCTCAAGCGCC

ACATCTTTACACTCGGATGGCAAAAAAGAATCGCTTCGGGGCTGGATTGAGCGCAATAGG

CTCCCCACAAGTTTACAGCTCGACTCGGACAACTTCCAAGATGTGATGAATGCAGCTCAT

AAGCCCCTCATCATCCTTGTCTCTACCCCCAGCGAAGGGCGATCCAAGACCATCGAACAA

GTCCAGGACATCGCTCAACGCTGGAAGGACGGTAAATACAAGGGCGATGTTGTCTTTGCG

TGGATGGATGCGGAGAAGTGGGGAAGCTGGTTGAAAAGCATGTATGGGATTCCGAACGAT

GGTGTTCCACATGTTGTTATCGCAAACCACTCGCGTCTAGTCTACTATGATACAACACCT

ACTGGGGCGAAGCTCAGTCTTAGTGTTGGCAGTATTTTCCCCACTGTTGCTAGTGCCTTG

GCAGGGTCGGGTTCCTACAAATACTCCGAGAACATTGTTGAACGGTTCGCCAAGTATATG

AACAACAAGCTAATATCTGTAGAGCGGGTGGTATCAGATCATCCTTGGGGTGTTGCGTTC

TTTGTCGTCAGCGTGGTTGTGGCTGTTGTTCTCTTGTTGAGGAGACTCATCGCGCAAGAG

TCAGCGATGGCCGATGACTTGGCCCGGAAAGCACGTCTCGACTAG

>TRINITY_DN10047_c0_g1|m.78 TRINITY_DN10047_c0_g1|g.78 ORF TRINITY_DN10047_c0_g1|g.78 TRINITY_DN10047_c0_g1|m.78 type:complete len:1356 (-) TRINITY_DN10047_c0_g1:2060-6127(-)

ATGCTACCCAAGGTAGCAAACCACATCATCCATCACACCGGTCGTGCCGTAGCCGCCGTC

CAAAACCAGACCAGTCACACTATCCGTAATGTTCTCCAGCTGCAGACCTCGACTGGCCCT

TCCTCTGCAGGCAACCTTGGGCCATGGAGTGGTCCTGGGTCTTCTAGCTCAGGCTGGGGT

GGAAACGGTCCTGGGACTGGGGGCGCAAAGCAGCACTCTAGTAGTCGCTTTCACACAGGT

TACACAAGCCTAACCCGGACAGCTACTCAAGCAGACCTTTCTTCGACCAGCCTTGTACAA

GAACGTTCAGACGATGTTGAGGACGTCCATTCTCCTTCCCACAATTCTACCAAGCGTTCG

CGTGCCCGCCCTCGTCCAAGAAGCAGCAGCTTATCTTTCACCCTTCAAGACCGGGATGAG

CTGAAGGGTGGTGTACTGAAGGCTGTTCAGCTTCACATCCGATCCCGACACGCTTTTGCT

GCACCCTCCTCCCATACCAATGCCGATGAACACCCTGTGGAGGTTCATGCTCCCAAGGTT

GCCTCAGACCCAGAGTCGATTCCCATTGAGGATACACTGGCTTCAGATGGGTGGCTCTCT

GCCCTCAAGTCAGCACGAGAGAGTCGCGACGCTTCTCGTGTCCTCCAAGAGGTTGAGAAA

CTCAAGTCCCATCCTGCACCAACCACAGCCCTCTACAATGCTGCTCTCGCCGCTCTATCT

TCTGTACGCAAGGCTGGTGAGCCTCTGCACGCACTGTTGTCCACCTATAACGACATGATT

GCGCTTTCGATTGCTCCAACGGTTGCAACCTACGGTATCCTCATTGTTGCACTCACAGAA

CGCGATCACGAAGTGGCAGGAGCGGTTCAAGCACTTGAACGTCGCATTCATCGTCGGTCT

ATCACAGGTCGTTCGGGTACTAACGCACTGGACGAAAAGCGCATAGCTCGTCTCCATGCT

GAGAATAACTTTGGCTCCGCACTGGCTCTGTTCCAAGCGGCTTGCCTTATCCCTCGAGCC

CGGCTCGGTGAAGTTTTATACACCAATTTGCTTCGCTCTTGCTCTCTCCATGGTAACATC

AGCGCGGCCATCCGCATATTTGCCCACCTCGAACGCCAGCCCGAATATGAACTCTCACCG

CTGGTCTTCAAGCATCTCGTTGCTACTTATAGTAAAGCGGGAGATTTGGATGGGGCAAAG

GAAGTGTTTGAAGAGTACAAGAAGAAATTAGAGTCGGGGTCCCTTGCACACAACTCGGAG

ATTGACGAAGTCAGCGGATTACCCTATTGGCGCACCGTGCAGATGGGTATTTGGAACAAG

ATGATTGAGGTGCATTTCCGGTGTGGTCATCCGGCTGGTGCACTTGATCTATTGGGACAA

ATGATGGATTCCGATTCTTCTGGCAGCACATCTGAAGTCACATCAGCACCCCCTCCTTCC

TCCATCACCTTTTACAACGTCATCGATGGTTTCTGTCAATCCGGCGACGTCAAGAGTGCC

TTGTCGTGGTTTGAACGCCTTCTCAATCAACAGGACAGTCCTACTTCGTCTCGCACTCCA

ACAGTTGTTCCTACGCGCCCTAACCGGTTTGCATGGTCGGTCATGCTTGAGGGCCTGGCT

CAATACGGTATGATCAAGGACCTCAATCGCCTGTTCGAAAAATTCGTCGAGACTGCCCCT

CGTGACGGTCTTCAGATCAACCACGCAGACTGCGGGCTCGTCGCCATGGCCAACCTCCAC

TACCTTGACATCCACCCTGAACTCGACAGTGCTGAAGCTGTCGGTATGTTGGATTTCGTG

GCCAACTTTGTGTGCGAGAGCACGAATCACGGAGACCGTATGATCCAGACGTTTGCATCT

CGGGATGTTTACCTATTACTTGTGTCGTTATACCTCCGTCATGGTGGACTTGAGCGTGCC

ATCACCCTCGCCACGCGTTACCTTGACTCGATGAGGCAAACGGTATATGCCCAGGAGTCT

CAGGTCGATGCAGACACGGTGCTCTCGACGGTTAAGCAATTCCGCTTGGTAACCCATGAC

TTCATACAGCGATTCATCCCCTTGGCCTCTATGTGGTCTCTGAACCTTGCCATCTGTTTG

GACGAGGCACGTGCTCGCTCACACCTGGTTGCAACACCGGCTTTTTCCGCCCAGGTCTTA

TCTGCTTTCGCCAGGGCCTCTGAGTTGGAGAAGGATTGCCTCACGCCATATCAGTGGGGT

TTGCTTCTCAACGCGTCCTCGGGCATACGACGTGCAACGATCAAGGAGCCCTCCCAGGCC

GTAGAAGCACCAGTCTCCATCCTCGATGTTCTCACTGAGATGTCGAAACGCCATGTACCT

CTCGATTCATTGTCTCATGATATTCCCCCCATCGTTGGCGGTGTTCTCGCACTGGAGGGA

CAGCGGGACAGTATTCTGGAAACTCTACGGTCGATCGGTTATAGCGACATCCTCGATAAG

TACATCATCTCAAGGGAGACAGGTGTTCAGGTTCCACAGACATCTACCGTCACCGAGGTA

TCAGATACACCCGAACCGGTAGCTGACAAGGGCATTCGTATCGACTTTTATCACAGTAAG

TTCGTTGACGAGTACTTCCCTTCGAACCCCGATATTACCCCCGCTATGGCTTATGAACGC

TTCGTCTCTGGTGCACGAGTCGGGGTGTACCCTGCTCCTGAAGTCCTAGGTCGCTTGATC

AACTCCCTGGGTAGATTGGGAGAAGTCGAAAAGGTCGGACAACTGTACAGCGCCGCCCAG

GACGTTCTCGCTGTATTGAGCCATGACAAGCAACGCCAGTCGATAGGTTGGTTCCAGATT

GAGGATCAGATGATCGTTGCGTTGGCGCACGCTGGTGATACAGATGCAGCCCATGTTCAT

CGCGGTCGTATCCTGGAGCATGGTGGCACACCCTCCGCGGACGCATATGGTGCACTAATT

CAGTGTGTCAAGGATACCACGGACGACACCTCGAACGCCATGGCTTTGTTCCAGGAAGCT

ATTGCTCACGGCGTCACCCCTAACATCTACCTATACAACACTGCAATTTCGAAGCTGGCA

AAGGCTCGCAAAGCCGACTATGCCTTGGAATTGTTCCAGGAGATGAAGGCTCGTGGCATT

CGTCCAACCTCGGTTACTTACGGTGCTGTCATTGCAGCGTGCTGCCGTGTTGGCGATGCT

CAGTCCGCGGAACTTCTCTTCCAAGAAATGTCGTCCCAGCCTAACTTCAAGCCCCGCGTC

CCTCCATACAACACTATGATGCAGCTCTATACTCACACCAAGCCTGATCGCGAGCGTGTT

CTGTACTATTACAATGCTCTATTAGGGGCTCGTGTGCGTCCTACTGCTCACACATACAAG

CTGCTCATTGATGCATATGGTACCATTGAACCTGCCAATGTGGAGGCCATGGAAGATGTC

TTCAAGCGGTTGGTCGCTGACCCTCAGGTTCCAGTTCAAGGAACCCATTGGGCCGCAGTC

ATTAACGCTTATGGTTGTGTGGGGAAGGACTTGGATAAGGCTATCTCAATCTTCGAGTCC

ATCGCACAGCACCCCGCGTCGCGACAATCAAAAACCTCCCTTCCTGATGCTGTTGTTTTC

GAGGCCCTCATGAACGTGCTTGTTACTCTTCGCCGCTCAGACCTTATCCCTGTCTACACT

GCCAAACTCTCTACATACGGTATCCACATGACCGCATACATTGCCAACCTTTTAATCAAG

GGCTACGCTGCAGCTGGTGACATCCATCAGGCTCGGGCGATTTTCGAAGATCTCAATGAT

CCTCCAGAGGGTGTTGCCGCTCCTAACAACCATGCCCCTCATGATAATGAGCAATCTTCT

CTAGTCCCTGCATTTGCGCCAGTCTACCGCGAGCCTTCAACATGGGAAGCTATGGTGCGT

GCAGAACTAGGTAATGGTAACCGGGATGAGGCTGTAGCTCTTCTCGAACGAGTAAAGGCT

AGGCGATTCCCTCCGGCTGTATACAACCGTATCAGCGGTATTATGCTCGATGACTCTGTC

TCTCCATGGGCACGGTCACCGGAGACATCTTCGCCATCTAGCCTTTAA

>TRINITY_DN10048_c0_g1|m.82 TRINITY_DN10048_c0_g1|g.82 ORF TRINITY_DN10048_c0_g1|g.82 TRINITY_DN10048_c0_g1|m.82 type:complete len:408 (+) TRINITY_DN10048_c0_g1:102-1325(+)

ATGAACATGATGATAGTGAGGCTGCTGCAACTAGTAATGGTGTTAGCTTGCATCTGTGCT

TGCAGTGGCTCAAGTCTAGTGACTGATCATCAGGACAACAGGAGGATTCTCCTCTCCAAT

GGCCTTGGCAAGACTCCTCCCATGGGCTGGAACAGCTGGAATCACTTCAATTGCTTCATC

AATGAAACAACTATCAAAGAAACAGCTGATGCATTGGTGTCCACTGGGCTTGCTGAGCTT

GGGTATCGCTATGTCAATATTGATGATTGCTGGGCTGAGTACAATCGCGATGCAAAGGGC

TATTTAGTTCCCCAAAAGTCAACGTTTCCATCAGGAATGAAAGCCCTTGCTGATTATGTT

CACAGCAAGGGGCTTAAGCTTGGAATTTATTCTGATGCAGGGTATAGAACATGTAGCAAA

AAGATGCCAGGGTCAATTGGTCATGAGGAGCAAGACGCTAAAACCTTTGCCTCATGGGGA

ATTGATTATCTGAAGTATGACAATTGTAACAATGGAAATGTGAAGCCAACTATTCGGTAT

CCGCTAATGACCCGGGCACTAATGAAGACTGGTCGGCCAATCTTTTTCTCTCTTTGTGAA

TGGGGGGATATGCACCCAGCTCTGTGGGGATCACAGTTAGGAAACAGTTGGAGAACAACC

AACGACATAAATGATTCATGGGAGAGCATGGTGTCAAGGGCAGACCAAAATGAAGTGTAT

GCTGAGTATGCAAGGCCCGGAGGATGGAATGATCCAGACATGCTTGAAGTTGGAAATGGA

GGGATGACAAAAGATGAATATATTGTACACTTCAGTATATGGGCAATCTCCAAAGCTCCC

CTTCTTATCGGATGCGACGTAAGAAACATGACTAAGGATACAATGAAAATACTTGGAAAC

AAAGAGGTCATTGCTGTTAACCAAGATCCTCTTGGCGTTCAGGCAAAGAAAGTTCGCATG

GAAGGAGACCTTGAGGTCTGGGCTGGACCTCTTTCTGGATATCGGACAGCTGTTGTCTTG

TTGAATCGGGCCACTAATAGTGAACCCACTCCCGCTATAGCAAATTGGGATGATATAGGC

ATTCCTTCGAACACACTCGTTGAAGCAAGAGATCTCTGGGAGCATCAAACACTGAAGGAG

CCAATTACCGACAAACTGACGGTCATACTTGGGCCCCACTCATGCAAGATGTTCATCTTA

ACACCTATTTTAACAGTTTCTTGA

>TRINITY_DN10052_c0_g1|m.85 TRINITY_DN10052_c0_g1|g.85 ORF TRINITY_DN10052_c0_g1|g.85 TRINITY_DN10052_c0_g1|m.85 type:complete len:221 (+) TRINITY_DN10052_c0_g1:217-879(+)

ATGATGACGATGGAGTCGGTCCTGGATGGGGAGCTCGATCTCGATTATTATGCTCGGATC

GTGGAGGGCGCCGAATCCATCGATTTCTACTTGAACGATTCCCCAAGTGCTGGAGCGGAA

TTCGATGCCTCTTTTGCTAATGTTATTACCCAAGAAAATGGTACTAAGAAAAGAGATCGT

GATGAGCAGTCTCTTAGGCAGGGTTCCAAAGCTTGCCGCGAGAAAATGCGGAGGGACAAG

ATGAATGAGAGGTTTTCAGAGCTAAGCAAACTTTTGGACCCCAGAAGGCCTGTCAAAACT

GACAAATCTGCAGTACTGGATGATGTCGTCCGTGTGTTGAATCAACTGAAAGCTGAAGCT

AAGGAGCTTAAAGAGGCAAATACAAAGCTTGAAGAAGAAATTAAAAGCTTAAAGGAGGAG

AAGCATGAGCTCCGGGAAGAGAAATCCTTAATGAAAGCGGAGAAGGAGAAGATAGAAGAG

CGTGTTAAAGCTATGTCAATGGTACCTGGTGGATATGTACCCCCACATCCAGCAGCGTAC

CATCCTGGTGTCAACAAGATGATTGCTTTTCCTAGTTATGGAACATTTCCTATGTGGCAG

TGGATGCCTCCTGCTGCTCGTGACATCTCAAAAGATCATGAGTTGAGGCCGCCGATGGCT

TAG

>TRINITY_DN10053_c0_g1|m.86 TRINITY_DN10053_c0_g1|g.86 ORF TRINITY_DN10053_c0_g1|g.86 TRINITY_DN10053_c0_g1|m.86 type:complete len:982 (-) TRINITY_DN10053_c0_g1:802-3747(-)

ATGCATCTTGCAAGGCATAAGAGGGTGAAGTATCATAATGAAGATATAAATTACACTTTC

TCATCGAGGAACGCAATACTTGCTGACCATAAAGGAGAAGGGACTTCCATAAATGTCAAA

GATTATTCAGATGCCTATGCTTTATCCAATCTCTTTCAAGATGATGGAATGTATGGGAGT

GTCACAAAGGACTTTGAATCTTTGCATCCTCAAAGAATGCAGATGATAAATTATCTGTCA

GCACTTCAGACGCCAATTTCTAGTCCTTGTTCAGGTTCACAAATGCTTTCCCAAGCTTTC

AGAGTTACTACATCTAATCCATCTTCTATGTATCAACAAATCGGGGATGTAATTGACTTG

GAAGAACGAAAAGAACCAGAAAGCAAAACAGGAAATGATGTCACGTCAGCCATAGTAGTT

GATTCAGATGATGAGGATGGAAATCGTCATGTGCAGAACATGCAGCCTTCCAGTTCAGAC

CGGCTTCATGATTTTAGAGCGTGGTTGAGCTCTCAGATAGAGGAACGACTGAAAAGAAAT

AGGTTGTCTGCCGGGGCAGCTAATTCTGCTCTTCAAGTTTCTGCTGAACAAGCAATAGCT

ATAGAAGGGAAAAGGCTTCCTTCTATTCAGTATGAAAAGGTTGTTCTGAGGAATGCAACG

GAGAAACAACTTGCTCCGAATCTTGAGCTTGTGCAGAATCAAAGTTTAGTTGTAGGAAGA

GAAAGGGAAGGTGAAGCTCTAGCATCTGTAGTGAATGTGAAAAAGAATCAAAGGGCTAAA

GTTGCAAGAGGTGAAAGAAAAGGAAATGATTTAAATGTTAAAGGCAATGCTTTAAATGTT

GAAGTGAACATGGAGAAAGAAACTGCTATTAGTTCTTCCCCAGTTTCTTATGAGGACGAC

CATTACTATACACCAAGCATGAAAACTCATCAAGACGATACAGAAACACCAAAAAGTGAT

GGTCTAGAAGATCTTTGGAAAGATATGTCTGTAGCAATGGAGTGCTCAAAGTCTACAGCA

CCAACAGATGAAGCAAGTATTGTTCAAGAAGATGAAGAAGAAGAAGAATGTGCTCATTCC

TTCATGCTTGAGGATGATCTCGGGCTTGTTTGTCGTGTTTGCGGAGTCATTCAGAAAAGA

ATTGAGACAATTTTTGATTACCAGTGGATTAAGGGTACCCGGACAACAAGGACTTACTTG

CCTGGATCAAGGAATTCCAATTTGGGTGACATAGCTGAAACTTCTGGAACTAATATCGCA

GAGCACTTTCTTATTGCTGCTGATATTTCTATCCATCCAAGACACATGAAGCAGATGAAG

CCCCACCAAATAGAAGGTTTTAACTTCTTAGTTAAAAATTTACTGGGTGATCCGCCAGGG

GGCTGCATTCTGGCTCATGCACCAGGTTCTGGAAAGACATTTATGCTCATAAGCTTTATC

CAGAGCTTTATGGCTAAAGATCCCCTTGCAAGGCCATTAGTTGTTCTGCCAAAGGGAATC

TTGGCCACATGGAGGAAAGAATTTCAACTCTGGCAAGTGGAAGACATCCCACTGTATGAT

TTCTACTCCTCCAAAGCCGATAACAGGTTTCAACAATTAGAAGTTTTAAACAAGTGGCAG

GAGAATAGAAGCATTCTACTTCTGGGCTACAAGCAGTTTGCGAATATTATCTGTGAAGGT

GGTGATAACAAGGTAACAGCAGCATGCCAGGATAAGCTCCTGAAGGTCCCAACTCTTCTT

ATTCTTGATGAAGGCCATACTCCACGGAATGATAACACGGATATGGTGGATTCACTTGCA

AAAGTTAAAACGCCATGTAAAGTAGTCCTGTCAGGGACTTTATTCCAGAATCATGTGGGA

GAAGTATTCAACATTCTGAAGCTTGTACGCCCAAATTTCCTGAAGACTGAGTCATCACGT

GCTATTGTAAAGCGTATCTTGAGCCGGGTACATATATCAGGTGGTAGGAAGCTAACCAAG

GGCAATCAGGAGTCGTATTTCTGTGATTTGGTTGAGGATACACTGCGGAATGATGAAAAT

TATAAGAGGAAAATTGCAGTAATTCAAGAGCTAAGGGACATGACAAGAAATGTTCTTCAT

TATTATAAAGGAGATTTCTTGGATGAACTCCCTGGAATGGTTGACTTCACTGTTTTTTTG

AATCTTGGCCCCAAACAAAACGAAGTAATCAGGAAGTTAGGTAAGCTTGATAAGTTCAAG

AAAAGCTCTTTGGAGAAAGCTATATATATTCACCCGCAGCTAATGGAACTTAGAGAAAAT

GCCTCAGGGGACAAAGATACCAGTATTAACATACACAGGGTTGATGCCATTATAGAATCT

ATAAATGTCAGAGAAGGGGCAAAAGCAAAGTTCTTTCTTAATCTCCTGGCAATGGCTGAG

CATTCTGGGGAGAAGCTACTTGTATTCAGTCAATACCTTGTTCCATTGAAATTTTTGGAA

AGGTTGATAATTCGGCTAAAAGGATGGCATCCTAGGAAAGAAATCTTTGTCATCTCTGGC

GATTCTAGTCCTGAGGACCGGGAATGGTCAATGGATCAATTCAACAACTCCCCAGATGCT

AAGGTTTTCTTTGGATCCATTAAGGCCTGTGGAGAGGGTATTTCTCTTGTGGGTGCATCA

AGAGTTGTCATCTTGGACGTCCACCTTAACCCTTCAGTTACTCGGCAAGCAATTGGACGT

GCATTTCGTCCGGGGCAACAGAAAAAAGTTTACATATATAGACTGGTTGCAGCTGATTCC

CAGGAGGAGGATGACCACAATACCTCATTTCGGAAGGAAACAATTTCAAAGTTGTGGTTC

GAATGGAGTGAGCATCATGGTCATCACGACTTCGAGTTGGATGCCATTAACATCGGAGAG

AGTCAGGACGTGTTCTTTGAAAACCCTGCTCTTGGTGAAGATATCAAAGCGCTCTACATA

AGGTGA

>TRINITY_DN100554_c0_g2|m.88 TRINITY_DN100554_c0_g2|g.88 ORF TRINITY_DN100554_c0_g2|g.88 TRINITY_DN100554_c0_g2|m.88 type:internal len:100 (+) TRINITY_DN100554_c0_g2:2-298(+)

TCCACCATGCACTACACAACTTTGCTTGTCGATACTATTTCTCCTTTCTCTTCCCTTGCA

CTCCAAGTTCCTAGCTCTACTCCGCTCGTTTCTATAGAGCCCATTCTCACCGCCCGCTAT

CCCGACTTGCCATACCATGCTCTTTCCCTCGCCTCACCTAGTGGCTTTGACCTGTCCTCG

GATACACCACTTTCGACTCTTGCGACCGGTGGAGACCTGGTCACGCTGAGACTTATGCCG

AGCCTGCTTGGTGGAAAGGGAGGTTTCGGGTCCCAGCTTCGTGCTGCTGGTGGGCGT

>TRINITY_DN1005_c0_g1|m.89 TRINITY_DN1005_c0_g1|g.89 ORF TRINITY_DN1005_c0_g1|g.89 TRINITY_DN1005_c0_g1|m.89 type:complete len:575 (+) TRINITY_DN1005_c0_g1:376-2100(+)

ATGGCGATGGGTCGGATTGTTTGTTTAGTGGTGGTGGTGGTGTTGTTCTGTTGCTGGAAC

ATTGGCGGTGTCGAGGCGTCTCATGCCGTGTATGAGGATCTCATGTCGGTCCCGGCGGTC

GTCGTCGACAGCCAGTACAGGACTGCCTTCCATTTCCAGCCACCAAGGAATTGGATCAAT

GATCCAAATGGACCAATGTACTACAACGGCGTATACCATCTCTTCTACCAATACAACCCA

TACGGAGCCGTATGGGGCAACATCGTGTGGGCCCACTCCGTATCAACGGACCTGATCAAC

TGGAAGGCGCTCGAACCCGCAATCTACCCGTCGAAACCTTTCGACATTAACGGGTGCTGG

TCCGGGTCCGCAACCATCCTCCCGGGCAACAAACCCGTCATCCTCTACACGGGTGTGGAC

CCGCAGAACAGGCAGGTCCAAAACATTGCATTCCCAAAGAACCTATCGGACCCATACCTC

CGCGAATGGGTCAAACCCGATTACAACCCGATAATCGCCCCGATCAACGGGATCAACGCC

AGCGCTTTCCGTGACCCGACGACGGCCTGGTACGGGCCCGACGGGCACTGGAGGCTGGTT

ATTGGCAGCAAGAGGAAGCACAGGGGGATGGCCATCATGTACCGTAGCAAGGACTTCGTC

CATTGGATCCGGGCGAAGCACCCGTTGCACTCCGCTAACGGCACGGGTATGTGGGAGTGC

CCTGATTTCTACCCGGTATCCACTAAGGGTAAGTTGGGGGTGGATACTTCGGAGTACGGA

GCTGGGCTGAAGTATGTGCTCAAGAGCAGCTTGGACATTACCCGGTACGAGTATTATACG

CTCGGCTCGTATCTACGTGACGTAGACAGATACGTACCGGATGGTACGTCGCCCGATAAC

GGGTCGGGTTTGAGGTATGATTACGGTAACTTCTACGCGTCGAAGACGTTCTACGATGCG

GGCAAGAAGAGGAGGATCCTGTTGGGGTGGTCCAATGAGTCCGATAGTAGGCAGGATGAT

GTGAACAAGGGTTGGGCAGGGATTCAGACAATTCCTCGGGCAGTATGGCTTGATAGCAAT

GAGAGGCAACTGATTCAGTGGCCAGTTGAGGAGATTGAGGCTATTAGAGGGAAGCAAGTT

TCATTGAAGAACATTAAAGTCAAGAGTGGGTATTTTGTTGAAGTCTCTGGGATTATGGCT

GCACAGGCAGACGTGGAGGCGAGATTTGAATTGTCAAGCCTAGCAAAGGCTGAGCCATTT

GATCCGTCTTGGACTGACCCACAGAAGCTGTGTGAGCTCAAGGGTGCAGGTGTCAAGGGT

GGGGTTGGACCCTTTGGGCTCCTCACCTTGGCCTCTGCCAAGAGAGAGGAGCAAACTGCT

GTTTTCTTCACCATCTTCAAGGCTACCAATGGCTATGTTGCCCTCATGTGCCATGATCCC

TCAAGGTCGTCAGTAAGGCCAGGATTGTACAAACCAACCTATGGAGGGTGGCTTGATGTT

GACATCCAAAAGAATGGGATGATATCTCTAAGGAGTCTGATCGACCACTCTGCGGTGGAG

AGCTTCGGAGGTGAAGGCAGGACATGCATCACTTCTCGGGTTTACCCGAGCCTGGCAGTA

GGCAATGCTGCTCACCTGTTCGTCTTCAACAACGGAGCAGAAGATGTGAAGATCTCCGAG

CTCAGGGCGTGGGAGATGAGGAAGCCTTTGATGAACGGAGCATAA

>TRINITY_DN1005_c1_g1|m.91 TRINITY_DN1005_c1_g1|g.91 ORF TRINITY_DN1005_c1_g1|g.91 TRINITY_DN1005_c1_g1|m.91 type:5prime_partial len:502 (-) TRINITY_DN1005_c1_g1:553-2058(-)

GATCCAAATGGACCAATGTACTATAATGGCATCTACCACTTATTCTACCAGTACAACCCA

TACGCCGCAGTATGGGGCAACATCTCATGGGGTCACTCAGTCTCAACCGACCTCATTCAC

TGGGCCGGGCTCGAACTTGCACTCTCTCCTACTGATCCATTCGACATCAATGGGTGCTGG

ACTGGGTCAGCCACAATCCTACCGGGCAATAAACCTGTTATCATCTATACAGGCAGTGAC

CCAAAGAAGAGGCAGGTTCAAAACATTGCATACCCGAAAAACTTATCCGACCCATTCCTT

CGTGAATGGATCAAACCTGATTATAACCCGCTTATAGAACCAGTTGACGGACTCAATTCA

AGCCAATTTCGTGACCCGACCACAGGTTGGCTTGGACGGGATGGGCTCTGGAGAATAGCG

GTTGGAGCTGAGATTGGATTAAACGGGCGGGCCTTATTATATAGAAGTAAAGATTTTGTA

CATTGGGTTCGGGCTAAAAACCCATTGCATGCTACAAATGGATCAGGTATGTGGGAATGC

CCAGACTTCTATTCTCTTGAGGGAAAGGAGAGGAAATATGTGCTGAAAATGAGTTTGGGT

GAAACTCAGTCTGATCACTACATGTTGGGAAGATATGATGAGGAAAAAGATGTCTTTGTG

CGTGATGACCCATCAGATGATTACCGGATGTGGCGAAGGTACGATTACGGGACGTTTTAT

GCATCAAAGACGTTCTTTGACGAGAAGAAACAGCGAAGGATCTTGTGGGGTTGGGTGAAG

GAATCTGATATCGTTGCAGACGATGTGGCAAAAGGGTGGTCTGGTATTCAGGCAGTCCCC

AGGGTTGTTAGTCTAGATACCAATGAAAGACAGCTGGTGCAGTGGCCCGTTAAGGAGCTT

GAATCACTTCGAAAGAAACAGACACGTTTACATGACATTGAGCTCGAGGCAGGAGGCCTA

GTTGAAATCAAAGGCCTAACAGTTTCCGAGGCAGATGTTGAGGTAGAATTCGAGTTGCCA

AGCTTGAAGTCAGCTGAGCCTTTTAATGCTAATTGGGTTTTAGACCCTCCAATGCTTTGT

CGTGAAAAGGGTGCTTACGTTAATGGTGGGGTTGGACCTATCGGGTTGTTAGTGTTGGCT

TCACGTAACTTAGAGGAACATACTGCCATTTTCTTCAGGGTTTTCAAATCTCATGATAAT

TACAAGGTTCTCATGTGCGCTGATGAGAGAAGGTCCTCCTTGCGAGCAGAAGTATACAAG

CCAGCATATGGTGCCTTTGTTGACATAGACATAAAGAAAGATGGGAAAATTTCTTTAAGG

ACTCTGATTGATCATTCTGTCATAGAGAGCTTTGGTGCTGGGGGAAGGACATGCATCACA

TCTAGAGTTTATCCTACATTGCTCGTGGGCGGTGACCCCCATCTCTATGCTTTTAACAAT

GGGACCGAGACCATAAAGATTTCGCACCTGAAGGCATGGAACATGGCTAAATCTCGGATA

AGCTAA

>TRINITY_DN1005_c1_g2|m.93 TRINITY_DN1005_c1_g2|g.93 ORF TRINITY_DN1005_c1_g2|g.93 TRINITY_DN1005_c1_g2|m.93 type:5prime_partial len:502 (-) TRINITY_DN1005_c1_g2:236-1741(-)

GATCCAAATGGACCAATGTACTATAATGGCATCTACCACTTATTCTACCAGTACAACCCA

TACGCCGCAGTATGGGGCAACATCTCATGGGGTCACTCAGTCTCAACCGACCTCATTCAA

TGGACTGGCCTTGAAGTTGCACTCTCCCCTATCAATCCATATGACAAAAACGGTTGTTTT

TCGGGATCTGCAACGATTCTACCGGGCAATAAACCAGTCATCATCTATACAGGTGTTGAC

GAAAAGAACAGGCAAGTTCAAAACATTGCATATCCGAAAAACTTATCTGACCCGATGCTT

CGTGAATGGATCAAATATGATCATAACCCGGTTATGGAACCAGTTGACGGGCTCAACGCA

GGCCAATTTCGTGACCCAACCACAGGTTGGCTTGGACGGGACGGGCTTTGGAGAGTAGTG

GTTGGGGCAGAGATTGGACTGAAGGGGCAGGCCATGTTATATAGGAGCAAAGATTTTGTA

CAATGGGCGCGGGCTGAGAATCCGTTGCATGCTACAAATGGATCACGTATGTGGGAATGC

CCGGACTTCTATTCTTTGAAGGGAAACGAGAAGAAATATGTGCTGAAGATGAGTTTGGGG

GAGACAGTGTCTGACCATTACATGTTGGGACAATATGATGAGGAGAAAGATGTGTTTGTG

CGTGATGAACCATCAGATGATTACCGGATGTGGCGAAGGTATGATTATGGCAAATTTTAT

GCATCAAAGTCTTTCTTTGATGAGAAGCAGCGGCGAAGGATCTTATGGGGGTGGGTGAAT

GAATCTGATAGCGTAACGGATGATAACACAAAAGGGTGGTCTGGTATTCAGACTTTTCCA

AGGGTTGTTAGTCTAGATGACAATGGGAGGCAACTGTTGCAGTGGCCCATCAAGGAGCTT

GAATCATTTAGGAGGAAACAAGGAAATTTACATGACATTGAGCTTGAGACAGGAGGTTTA

GTTGAAGTTGAAGGGCTGAAAGTTTCCGAGGCAGATGTTGAAGTAGAATTCGAGTTGTTA

AGCTTGAAGACAGCCGAACCTTTCAATGATGATTGGGTTTTAGACCCCCCTATGCTTTGT

CGCGAAAAGGGTGCTTCTGTTAACGGTGGGGTTGGCCCTTTCGGGTTGCTAGTGTTGGCT

TCACGTAACTTGGAGGAACATACTGCTATTTTCTTCAGGGTTTTTAAATCTGGCGAAGTT

TATAAGGTTCTCATGTGCTCCGACCTGAGAAGGTCCTCCTTGAGAGCAGAAGTCGACAAG

CCAGCATACGGAGCCTTTGTGGACATAGAGATAAAGAATGGTGGAAAGATATCTTTAAAA

GCTCTGATTGATCATTCAATCGTAGAGTGCTTCGGCGCTGGGGGGAAAACATGCATCACG

TCTAGAGTTTATCCTACATTGCTTGTGAGTGGTGGTGCCCATCTCTATGCATTCAACAAT

GGGACAGAGAGCGTAAAGATTTCGGACCTGAAAGCATGGAGCATGGATAGACCTAAGATA

AGTTAA

>TRINITY_DN1005_c1_g2|m.94 TRINITY_DN1005_c1_g2|g.94 ORF TRINITY_DN1005_c1_g2|g.94 TRINITY_DN1005_c1_g2|m.94 type:5prime_partial len:139 (-) TRINITY_DN1005_c1_g2:1324-1740(-)

ATCCAAATGGACCAATGTACTATAATGGCATCTACCACTTATTCTACCAGTACAACCCAT

ACGCCGCAGTATGGGGCAACATCTCATGGGGTCACTCAGTCTCAACCGACCTCATTCAAT

GGACTGGCCTTGAAGTTGCACTCTCCCCTATCAATCCATATGACAAAAACGGTTGTTTTT

CGGGATCTGCAACGATTCTACCGGGCAATAAACCAGTCATCATCTATACAGGTGTTGACG

AAAAGAACAGGCAAGTTCAAAACATTGCATATCCGAAAAACTTATCTGACCCGATGCTTC

GTGAATGGATCAAATATGATCATAACCCGGTTATGGAACCAGTTGACGGGCTCAACGCAG

GCCAATTTCGTGACCCAACCACAGGTTGGCTTGGACGGGACGGGCTTTGGAGAGTAG

>TRINITY_DN100600_c0_g1|m.96 TRINITY_DN100600_c0_g1|g.96 ORF TRINITY_DN100600_c0_g1|g.96 TRINITY_DN100600_c0_g1|m.96 type:5prime_partial len:136 (-) TRINITY_DN100600_c0_g1:148-555(-)

GACAGTCCGAAATTTAGTTTTCTCTCTGCCCTCTATCCAGAAATGTCGCTCGTCACCAAG

CCCACCAAGACCGTCGAGGCGGAGGATGTCCGCATCCACCGCATCCGCATCACGCTGTCC

AGCCGCAACGTGAAGAACCTCGAGAAGGTGTGCGCGGACCTCAAGCGTGGCGCCGTCGAC

AAGAACCTCAAGGTCTCGGGCCCCGTGCGCCTCCCCACCAAGATCCTGCGCCTCACGACG

CGCAAGTCGCCGTGTGGTGAGGGTACCAACACGTGGGACCGCTTCGAGATGCGCATCCAC

AAGCGCATCATCGACCTGCACGCGCCCTCGGACATCGTCAAGCAGATCACCTCCATCTCG

ATCGAGCCGGGTGTCGAGGTCGAGGTCACGATCGCCGACTCGGCCTAA

>TRINITY_DN10061_c0_g1|m.99 TRINITY_DN10061_c0_g1|g.99 ORF TRINITY_DN10061_c0_g1|g.99 TRINITY_DN10061_c0_g1|m.99 type:complete len:567 (+) TRINITY_DN10061_c0_g1:314-2014(+)

ATGAAGCAAGAGGTTCCCACACTAGAGAGCAGGGCCTCAACCTTCTTGCATTCTCCGGTA

CCTGCCACCCGTGCTAGGGTTTCGAGGACTAAGGATAGGCCGAGAGTAGACTCTCCCAAT

GGGGTCTTACTCTCGCCAGGATCAAAAGCAAGCCCGAAACCATCTCCACCAGAGGGAAAC

AACATTTCCCGAGTTAGAAGGCCTATTCTCCCTACCAAGCCAAAGGAAAGGGAGGTGGAG

GATCCAAAGATTGTTGATCAACCGCAAACCCGTACAGTCGAACAGTATGCACGGGTGAGG

CGCCATGTGGATACTAATCGCAGGGGATCGGAGTTTCAGAGTGATGGGAAAGTGAAGGAT

GATTTGCAGAGAAGGCTTCATGTGAGTGAGAGCTTGGTTAAGGAGTTGCAATCTGAGGTT

GAAGCTTTGAAGGCACTGGCAGAGAAGCTGCAGAGCCGTAATGTTCATCTGGAGTTGCAG

AACAAACAGCTTGGCAAAGATCTATTTTCAGCTGAAGAAAAGATTAAAACTCTCGAGAAG

CTTGATCAGATAGAACATGTCAGCAAAGAAATTCATCCTTCTGAATTTAAGAATGTACGA

GAACTCATTGCAAATAAGTTAGACCTTTTTCCAGTGAAAAGAGAAGTTACTAAAGGAGAT

AAAGATGCAAAAATGCCGTCTTTACCACTGGGGGCAGGTGTTAAATCTGCAGAAATTCAT

CCAAAAGTTCAGTATACAGTGCCTCCATCTCGTGCTGCTTTCGTTACGGGGCCACCACCA

CCTCCTCCATCTCAGGCTGCTCCAGTTCCAGGGCCGCCTCCACCTCCTCCCCTCCCTCCC

CCTCGTACTGTTTCTTCAAGAACAAATACAATGCAAAAACCTACGGCATTAGTAGAGTTC

TATCACTCAATAACCAAGAGAGATGGAAAGAGGGGGCACTTGGGAAGTGAGAATTGTGCT

AGTCCACTCGCTAATAATGTTCACAACAGCATTGTTGACGAACTTCAAAACCGGTCAGCT

CATTTGTTAGCGATTAAAGCAGATGTAGAGAAGAAAGGAGACTTCATCAATCATCTCATA

CAGAAGGTCCAATCTGCAGCTTTTACTGATATTGAAGAGGTTCCGACATTTGTTGATTGG

CTTGACGGGCAACTCTCTACATTGGCTGATGAGAGAGCCGTACTGAAGCATTTCAGCTGG

CCTGAAAGGAAAGCAGATGCATTGAGAGAAGCTGCTTTTGAGTACCGTGATCTTAATCGG

TTATTGGTTGAGATTTCTTCTTTTCAGGATAATCTCTCTCTTTCATGTGAGGCCACCTTG

AAGAGGATCGCAGCCTTACTAGATAAGTCAGAGAGAAGTATTCAGAGGTTAATTAAGTTG

AGAGACACAACCATGGTTTCTTTTAGGGACTGCAAAATTTCCGTTGACTGGATGCTTGAT

TCAGGAATGATTTGCAAGATAAAGCAGGCTTCTGTGAGACTTGCAAAAGTTTATATCAAG

AGAGTGTCAGCTGAACTCAAAATGATAAGACATACTGAGAGAGAATCAATACAGGAAGCG

CTTTTACTTCAGGGTGTGCGTTTTGCATACCGGGCATACCAGTTTGCGGGAGGACTAGAC

TCTGAAACAATGCGTGCCTTTGAAGAGCTAAGGGAGCGAGTTCAGTTACATAGGAGAGGA

TCGGGAGAGTTCATGGCTTAA

>TRINITY_DN10062_c0_g1|m.101 TRINITY_DN10062_c0_g1|g.101 ORF TRINITY_DN10062_c0_g1|g.101 TRINITY_DN10062_c0_g1|m.101 type:complete len:176 (-) TRINITY_DN10062_c0_g1:295-822(-)

ATGGCTGCTTTGGGGAGAAATGTAGCAGCACCGCTCCTGTTCCTGAACCTCGTCATGTAT

CTCATTGTCATCGGGTTCGCGAGCTGGTGCTTGAACCACTTCATCAATGGCCAGACCAAC

CACCCAGGCCTGGCGGGTAACGGGGCAACGTTCTACTTCCTGGTGTTTGCCATCCTCGCC

GGCGTTGTCGGCGTGACGACCAAGCTGTCCGGCGCCTACCACATCCGGTCGTGGCGCAAC

GACACGCTGGCGGCCGCGGCGTCATCGGCCGCCGTTGCGTGGGCCATAACGGCCTTGGCC

TTCGGGGTGGCATGCAAGGAGATTCACATGGGCGGATACAGAGGGTGGAGGCTGAGGGTG

CTGGAGGCATTCATCATAATTCTTGCCTTCACGCAGCTGCTGTACGTTCTGCTCCTGCAC

GCGGGGATGTTCAGCAGCAAGTACGGGCCCGGGTACAGAGACCCCGACTACGGCGTCGCC

GCCGGAGCGGCCAGCGAGGGGATGCCGAAGGGCACGGCGAGAGTGTAG

>TRINITY_DN10067_c0_g2|m.102 TRINITY_DN10067_c0_g2|g.102 ORF TRINITY_DN10067_c0_g2|g.102 TRINITY_DN10067_c0_g2|m.102 type:5prime_partial len:467 (+) TRINITY_DN10067_c0_g2:1-1401(+)

CGACCACGACGACCTCAACAGCATAGACCCCCTAGGACCTTGTCTTTCTTTCGTATAGCA

CATCCTTTTAATACCTACATCCATCCTCGACGCTCGATGCTCAGGGCCTTGACCCCTTCA

CGATGTCTCTTCATCCTTGCAACCGTATCAGTTCTCCTCTTATTCACTCTTGTCCCGCGA

TATGAACTCCTCGAATCAGAAAGTACGCAACTCGTCGCACGCCAGGTAGAGGTAGGGGGT

GGAAGTAGTGCTGAACCGGGAGCGAGTGCTTCTCCATCCCAGTCGCATAGTGCTGATCCT

TCCAGTGCCTCATCTCAACCCGTGACAAGCCAACAGCCTTCGACCACAAGTCAGCAGTCC

CAGACCAGCCAGCAACAGCAGCAAAGTAGCGCATCACAATCTTCTGCCAGTTCCAACACA

CCGCCCTCTGCTTCGTCCGTTCCGCCGAGCACTACGAGTGAGGCACCGCCCCAGACATCC

AACAACAATCAACCCGGTCCTGCTCAGTCATCCCCTACTACTGCGCCACCGACGCCCACT

CCCACGGTATCACAAGTCACATCGGTCAATTCAGATGGTGAAACTGTCGTAATTTCCGTT

ACGGTCACCCCTTCCATCACGACATCCCCAAGCGCAAGTTCAAGCGCAGGTTCGGGGAGT

GGTAATAACGACGGAGGAAGTGGTACCAGTACCGCTACTATCGTTGGGCTGAGTGTTGCA

GGTGGTGTCGCATTGATTGGCATCGCAGCCTTCGTGGTGTGGAAGTTTACTAGGAAGCGG

TTCTCGGACAGTGACTTTGATGACAACGAAGCTATCAAGTGGCCTGAGCTCAATACGCAT

GGTGAATCGCCTCACGCACTTCCTACGAACAAAACAGCGAACTTTGGCATGGAAACAAAC

TCGGAAGTTAACCTTACTCGTCCGGACTCCCGTGCAGGCTCATTTGCTCCTAGCGCTTCC

GCGTCCGCAGTCGACCTCTATGCCGCACAAGACCCTTACGCTGTACCACCACTCCCACAC

TTGAACCCCAATCAGCCCTATAGGGATGACCCACATGGTGGCGCATTTTACGACCCATAC

AGCGGACCTGTACCCCAAACGTTTGCAGACGCGGATACGGCAAGCATGCAGGGCGGTCCT

GAGGCAATACCGATGACGCAAATCAATCGGTCCCGGAGCCCAGGGCCGGCCATGGCTTAT

GACATGGCCGGCAGGGCCAGCCCAGGACCCCAGCAAGCTTACTTTGAGCAAGGGCGAGCG

AGTCCTGGTCCCAGGGCAATGAGCCCGGGGCCTCAGATGGCATTAGGTGGAAGGGCATCA

CCAGGGCCGCAAGTAGCTTATGGTGCTTACGGAGGGAGGCAATCACCTGGGCCTCAAATG

GCTTATGGAGGTCCACAATAA

>TRINITY_DN1006_c0_g1|m.103 TRINITY_DN1006_c0_g1|g.103 ORF TRINITY_DN1006_c0_g1|g.103 TRINITY_DN1006_c0_g1|m.103 type:complete len:579 (-) TRINITY_DN1006_c0_g1:438-2174(-)

ATGGCTGGTATATTACCAGGGGATCCTTCACATCACGGAATGGTGGAAAGTAGTCCCTAC

AGAATTTCTCATGACCAGATGGAGGAAGGTGATCACACAGGTTCTAGTTGGTATTTTAGC

CGGAAGGAAATAGAAGAAAATTCTCCATCTCGAAGAGACGGTATTGATCTTAAGAAAGAG

GCATATTATCGTAAGTCATATTGTACATTTCTGCAGGACTTAGGCATGAGACTTAAAGTA

CCTCAGGTCACAATCGCCACTGCTATTGTTTTCTGCCATCGTTTTTTCCTTCGTCAGTCT

CATTTGAAGAATGATCGAAGGACTATAGCTACTGTTTGCATGTTTTTGGCGGGAAAGGTT

GAAGAAACTCCTCGGCCTCTAAAGGATGTCATTCTTGTTTCGTATGAAATTATCAACAAA

AAGGATCCTAGTGCTGTGCAGAAAATTAAACAGAGGGAGGTGTACGAAGAACAGAAAGAA

CTTATCGTACTGGGGGAACGGGTAGTACTAACAACCCTTGGTTTTGATCTCAATGTGCAC

CATCCCTATAAACCCCTTGTGGAATCAATAAAGAAATTCAAAGTTGCGCAGAATGCCCTT

GCACAAGTTGCTTGGAACTTTGTTAATGATGGGCTACGTACTTCACTTTGCCTGCAATTT

AAGCCCCACCATATAGCTGCTGGTGCAATCTTTCTCGCCGCTAAGTTTCTGAAGGTGAAG

CTTCCATCTGATGGTGACAAGGTCTGGTGGCAGGAATTCGACGTCACACCAAGGCAATTG

GAAGAGGTTAGCAATCAAATGTTGGAATTGTACGAACAGAACCGGAATCAGCCACCATTC

CATGGAAATGAAACAGAAGGGAGCTCCGCCAGTGCAGCCAATCATCGGGCTCCTCCAAGA

GCATCAATTGCTACTGAGGAGGCACCTTCACAAAATGGACCACATCAAGCTGCTGGAACC

TCTTCTTTGCATCATGGCATTCAAGCTAGCTCAAGTCGACCTGCACCAGATCAAGTGCAC

ACTGAGAAGCAAAACGGACCCCAGAGATTTGCCCAGAGCGAAAGAAATGACCATGACAAC

AAGGACTTGAGATATGGGAAAACAGGAAGCGGGTCAAAAGATCGTCTGCAACACGAGCCA

GGAAATGTTACTGAAGGATTAACAAATAAACCTGCTCATGGTTCCGAACACCCAACTGAA

GAGCCAGGTGAAATTAGTCAAACAAGAGACAGGAGTTCCGGGCACAGTGAAGGCCCAAAA

AGCTACTCTCCATTAGATGCTATAAGAAAGATTGACAAGGACAAGGTGAAGGCCGCACTG

GAGAAACGAAGGAAGGAGCGAAGTGAGGTCTCAAAAAAGCTGGATGTTATGGATGATGAT

GACCTTATTGAGAGGGAGCTGGAAAGCGGTATTGAATTGGCAGCTGAGGATGAGAAGGTA

AAGCAGGAGAGGCGGCATGGCTGGTCCAAACCTTTGTGCAGGCAAGAGCCTCAGAACCAA

GACCATGGTATTGTGAACGGGGAGCTTGGAACTGAGAAGGAAATGGAGAATGCCGAAGAA

GGGGAATTCCACACACCAGAGCCTGCTAGTCGCAAGAGAAAGGATGGGTATGACCACCCG

TATCAGCACCATCCCCCACCCTTAAAACACCAAGATGCTGATGATTCACGTGCAATGGGC

CGGTTAGAACGGGCTGAGAGGGATCATAAAAGGATCAGGCAGGAAAATCATGTGTGA

>TRINITY_DN1006_c0_g1|m.104 TRINITY_DN1006_c0_g1|g.104 ORF TRINITY_DN1006_c0_g1|g.104 TRINITY_DN1006_c0_g1|m.104 type:complete len:120 (-) TRINITY_DN1006_c0_g1:1085-1444(-)

ATGGTGACAAGGTCTGGTGGCAGGAATTCGACGTCACACCAAGGCAATTGGAAGAGGTTA

GCAATCAAATGTTGGAATTGTACGAACAGAACCGGAATCAGCCACCATTCCATGGAAATG

AAACAGAAGGGAGCTCCGCCAGTGCAGCCAATCATCGGGCTCCTCCAAGAGCATCAATTG

CTACTGAGGAGGCACCTTCACAAAATGGACCACATCAAGCTGCTGGAACCTCTTCTTTGC

ATCATGGCATTCAAGCTAGCTCAAGTCGACCTGCACCAGATCAAGTGCACACTGAGAAGC

AAAACGGACCCCAGAGATTTGCCCAGAGCGAAAGAAATGACCATGACAACAAGGACTTGA

>TRINITY_DN1006_c1_g1|m.105 TRINITY_DN1006_c1_g1|g.105 ORF TRINITY_DN1006_c1_g1|g.105 TRINITY_DN1006_c1_g1|m.105 type:internal len:111 (-) TRINITY_DN1006_c1_g1:3-332(-)

AAGCCAAAGCCCGACGGCCCCGTAGCCGAAAAATTGACCCCCCAGCCGCTCAAGTCAGCC

CGGGCAGCCCCAACTTCCTGTAGGAGGTATATCTCATTCTCCTTGTTCAAATTTTCTTCT

ACAATGCCTAGGAAGATCACTAGGGATCAAATTTCTGTTTTACCGGAGGAAAACTTTGAT

GAGGTGTTTGATTTATTTGGTGTTTACTTTCTAGATGAAACTAGGAGAAGAGGATGGGAC

TATTTGTTGCATGATAAAAAAGATGATTTCTTTATTGAATATCTTGTGCAAAAAGTTTAT

AATGGGTTTGAGAAAAAGAATATTGATAAA

>TRINITY_DN100709_c0_g1|m.106 TRINITY_DN100709_c0_g1|g.106 ORF TRINITY_DN100709_c0_g1|g.106 TRINITY_DN100709_c0_g1|m.106 type:internal len:108 (-) TRINITY_DN100709_c0_g1:1-321(-)

GCGTTCGACGGAGTTCTGGGCACTTTAGCCCACTCTTTTTCCCCGACGGATGGGAGGTTC

CACTTGGATGCTGCGGAGAGCTGGGTGGCGGAGGGGGACGTGGCCGAGGCGGCCTCGGAC

AATGCGGTGGACTTGGAGTCGGTGGCCGTGCACGAGATCGGGCACCTGCTCGGGCTCGGG

CACTCGTCGGTGAAGGAGGCGATCATGTACCCGACGATAAGGACGAGGACGAGGAAGGTG

GAGCTGGCCAAGGATGATGTGGAGGGAGTGCAGAAGCTATACGGGAGCAACCCGAATTAC

CGCGGCGGGACGGCGGCGTCG

>TRINITY_DN10072_c0_g1|m.108 TRINITY_DN10072_c0_g1|g.108 ORF TRINITY_DN10072_c0_g1|g.108 TRINITY_DN10072_c0_g1|m.108 type:complete len:143 (-) TRINITY_DN10072_c0_g1:310-738(-)

ATGGGGAAGACCAGGGGAATGGGAGCTGGGCGCAAGCTCAAGACTCACAGGAGAAATCAG

AGATGGGCTGATAAGGCATACAAGAAAAGTCATCTTGGCAACGAATGGAAGAAGCCATTT

GCTGGTTCTTCCCATGCCAAGGGCATTGTCCTTGAGAAGATAGGCATTGAAGCCAAGCAG

CCTAACTCTGCTATCCGAAAGTGTGCCAGGGTTCAGCTCATTAAGAACGGCAAGAAAATT

GCAGCTTTTGTCCCGAATGACGGTTGCTTGAACTACATCGAAGAAAATGATGAAGTGTTG

ATTGCTGGATTTGGAAGGAAGGGTCACGCCGTGGGCGATATTCCTGGAGTTAGGTTCAAG

GTTGTCAAGGTCTCTGGGGTTTCGCTGCTGGCGCTCTTCAAGGAGAAGAAAGAGAAACCC

CGCTCTTAA

>TRINITY_DN10074_c0_g1|m.109 TRINITY_DN10074_c0_g1|g.109 ORF TRINITY_DN10074_c0_g1|g.109 TRINITY_DN10074_c0_g1|m.109 type:5prime_partial len:127 (+) TRINITY_DN10074_c0_g1:3-383(+)

AAAGCAAGTGCTAGCTATTACAGTTCAGCAGCAGTATTTACAAAAGGTAAGCAAATATAT

TTTAACAAGAAGCTCTCTCTCTCTCTCTCTCTCTCTCTCTCTCTCTCTAAGTTTACTTTC

ACCCCCATGAATCTTACAGGTCTCTGCCGACAGTTTCTAAAGACAGTTCATCATCATCCA

AAAGGAACAAAAAAGTTCAGTAGAAGCAAAAATAAAGTTGCAGACATAACATGTGAAGAT

AATGGAGCAATAAATTTCCCTTTTTTCAACTTTAGAGTGAAATATCCATACAAAAAATAT

TTTGTGAACATGTACATTGGAGATATTGAAAGTTATTTGTTCACAGCGGGTTCACTCTTC

ACTCTTGATTCATACAACTAA

>TRINITY_DN10076_c0_g1|m.110 TRINITY_DN10076_c0_g1|g.110 ORF TRINITY_DN10076_c0_g1|g.110 TRINITY_DN10076_c0_g1|m.110 type:5prime_partial len:374 (-) TRINITY_DN10076_c0_g1:1042-2163(-)

CAACAACCTAATGGAAGTTCTGGTACTTCCAGTCCCAAGTGGAAGAATGGGATGAAGCAG

CCAAATCATGCATCTACTTCCACAGCATCAGGTTCAAGTGCTCGTATGAACAATAATAGA

CCTAATACACCTCAAATACCCCCCGAGTCTAGTAAGCCTAATACACCAAGTGGGCCATCA

TCAGGCTATGTTCAGCCTCGCCCTGATATACCCGCAAACAATGGGAATATCTTTCCTAAC

CATATTCACACCCAATATTCTCAACCACCAGGGCCTAATGGTTTCCAGCCACAAACGAAT

CTTCAACCTTGGAATTTGTCTGAATCAAATTCCATGTACAACTCTTATCCTCCTTCACCA

GGGATCTATGGTCCATCCTTTTCTACAGCACCTACAACTCATGGCACGCCCTTTACTTCT

CTACCTGATATGAGTAGGCTTAGTATTTCAGAATATCCTAATGGTGGATATCAAAATATG

CCGCCATTTTATCACAACAACATGCATAGATCTACTCCTTCCATGGATAGCAACACATCC

AGCAATGGTTCATGGGGTACTCCTGGATGCCCCTCACCATCAAATTTTGTCTGGGAACAG

ATAAGATTTATTTTAGGAGCCTTGCACATTTTAAAGACTGACAAGATGGCCCCCTCCGAA

GCTAACATAGCAGATTGTATCCGCTATGGAGAGATGAACATCCAGAACTTTAATGTTAGG

AAGGCCCTGGAGTATGCTATCGACCATCAGGTTGTTTTGGTACACAAAATAGGAAACAGC

TTGCCTCTTTATGTAGGGAAGGCTGATACACTTTGGACATGCTTGAATATCATGGATAAT

AATGCTAAGCATGGTAAAGCAAAATTTGGTGCAGTTCAAAAATTTCTATCTTCTGCCGAT

GGACGTGCTGCAATATTAGCCTCACAATGCAGGTACCAAGCTGCTGTTATTGTCAAAAGA

TCATGTTTGAAAGATCTTGAGCTAGGCAAGATACTACAGATTTTGCATCTAATAATCGAG

GTCAAAAAGTGGATTGTACCGCATGCTTCTGGATGGCAGCCGTTGGATCTATCCATTTCA

CCAGATTCAAATGCAAATGCTGGCGCAAGTACTATCTCTTGA

>TRINITY_DN10076_c0_g2|m.111 TRINITY_DN10076_c0_g2|g.111 ORF TRINITY_DN10076_c0_g2|g.111 TRINITY_DN10076_c0_g2|m.111 type:5prime_partial len:274 (-) TRINITY_DN10076_c0_g2:761-1582(-)

AATGGGAGGTCTCATAATCGCAACAAAAGCAAGCAAAGTCGGAATACACAAAGCCAGACG

AATACCAACATACCCAGGACCACATCCAGTGGTAGTCAAGAAGGTCAGCAACCCATTGGA

AGCACTGGTGCTTCCAGTCCCAAGGGGAAGAAGGGGATGAAGCAGCCACATCATGCATCA

ACTTCCACAACATCAGGTTCCAGAGCATCAGAGGGTGCTCATATGAACAATAGTAGACCT

AATACAGCTCAAATACCAACACTATCTAATGTTGTCCAGGGACAGATAGGATCTATCTTA

CAAGGATTTATTTTGCAAGCCTTGCAGACTTTAAAGTCCGAGAAGATGGCTCCCACCGAA

GCTAACATAGCAGATTGTATCCGCTATGGAGAGATGAACATCCAGAACTTTGATGTTAGA

AAGGCCCTGGACCACGCTATCGAGCATCAGGTTGTTTCGGTGCACAAAATAGGGAACAAC

TCGCCTCTCTATTTGGGGAAGACCGATACACTTTGGAAATGCGTGAATATTGTGGATAAT

AATGCTAAGCATGCTAAAGCAGTATTTGATGCAGTCCTAAAATTCTTATCTTCTGCCGAT

GGGCGTGATGCAATATTAGCCTCACAATCCAGGTACCAGGCTGCAGTTACACTCAAGAGA

TCATGTTTGAAAGATCTTGTGCTTGGCAAGATTCTACGGATTTTGCATCTAACTGTCCAG

ATCAAGAAATGGATTGTGCCGCATGCTTCTGGATGGCAGCCGTTGGTTCTAGCCATTCCA

CCCGATTCAGATGCAAATGTTGGTGCAAGTACTAGCTCTTGA

>TRINITY_DN10077_c0_g1|m.113 TRINITY_DN10077_c0_g1|g.113 ORF TRINITY_DN10077_c0_g1|g.113 TRINITY_DN10077_c0_g1|m.113 type:internal len:155 (+) TRINITY_DN10077_c0_g1:2-463(+)

ACCGTGGCCGCCGTGGCCCTCCTCTGCGGCGTGTCCTACCTCCTTGGCGTCTACAACAAC

CACCAGGGCGGCAGCGACACCGTCTCCGCCGTCTCCATCTCCGCCGCCACCGTTCCCTGC

CACCTCACCAACATCCCCTCCTCCTCCTCCTTCTCCTCCAACCTCACCCTAGACTTCGGG

GCCCACCACAACTTCGACGACTCCGCTGTCAACGCCCCAACCGAGTTCCCCCCGTGCGGC

TCAAAATACTCCGAGTACACCCCGTGCGAGGACCGGGACCGGTCGCTGAAGTTCGACCGG

GACAGGCTTATTTACCGCGAGCGCCACTGCCCGGCGAAGGGCGAGCTGCTGAAGTGCCTC

GTCCCGGCCCCGCACGGGTACAAGAACCCGTTCCCGTGGCCGGCCAGCCGCGGCGTCGCC

TGGTTCGACAACGTCCCCCACAGGGAACTCACCGTCGAGAAG

>TRINITY_DN10078_c0_g1|m.114 TRINITY_DN10078_c0_g1|g.114 ORF TRINITY_DN10078_c0_g1|g.114 TRINITY_DN10078_c0_g1|m.114 type:5prime_partial len:462 (+) TRINITY_DN10078_c0_g1:2-1387(+)

TCTCTCGGGGTCCCAATCGAAAAAAAAAAAAAAATGTACCTCAGGTCGGGACCGGGACCT

CGACTTGGCCTCCACTGTCAACTCCGCCATGGGATGTCGTCTCCGCTTGACCTCGGAAGC

TCGACTTCGTCTCCGCCTCCTCGTTCTCCGCCTTGCCCGCCTCAGAGTCGGGACCTCGGA

AGCTCGACTCAGCCTCCACAAGCTCGACTCCGCCTCAGTTGCCGCCTCAGCAAGGGACCT

CCGACTTCGCCTCCGCAAGGTCGTTGTCCACCAGTCCAGTATTTAATGCACCAAGATTGT

GACACCATAGTTAAATACAGCCCTGGGAGAATTCAATGGCGTCTCATAAACTGTCGACAT

ACTAATGCAAAATCTACATACTATGGGAGATCTTATGAGTTGAGCCATGTAAAAGCCACA

TCAGGACATCCTTTAGAATCTGAACCTGAAGAATACTCTTCCACAAGCACATGGAGAGCG

ATTCTGGCTTCACTAAGTGTACTTTACCGGTTCTCAAGGCCTCACACAGTTATAGGAACA

GCTATGGGCATAATCTCGGTTTCCCTGCTGGCTGTTGAGAATTTGTCTGATATTTCTCCA

CTGTTTTTTGCTGGAATGTTGGAGGCAGTGGTAGCTGCACTTTTCATGAATGTTTACATT

GTTGGACTAAATCAGTTGTTTGATATAGAAATAGACAAGGTTAATAAGCCAGATCTTCCT

CTTGCATCCGGAGAGTATTCTCTGGGAACTGGCATTGCAACTATTACTGCCTCTGCTATC

ATGAGCTTTGGCATTGGATGGTTAGTTGGTTCGTGGCCATTGTTCTGGGCTCTTTTCATC

AGTTTTATCCTTGGCACTGCATATTCAATCGATCTACCGTTTTTAAGATGGAAGAGATTT

GCTATTGTTGCTGCAATCTGTATCCTCTCTGTGCGTGCAGTTATTGTTCAGCTAGCTTTT

TTCTTGCACATGCAGACCTTTGTTTTCAGAAGACAAGCAAACTTCTCCAGGCCATTGATA

TTTGCAACAGCCTTTATGAGCTTCTTCTCAGTTGTTATTGCATTATTTAAGGACATACCT

GATATTGATGGAGATCGCATATTTGGCATTCATTCCTTCAGCGTACGACTCGGTCAACAG

CGGGTGTTTTGGATTTGTATATATCTACTTGAGATGGCTTACACTGTTGCCATGGTTTTT

GGAGCCACTTCTTCCTGCCTCTGGAGCAAATGCATAACGGTCTCGGGTCATGCAATCCTT

GCTTCAATACTCTGGAACCGTGCTAGATCTCTTGACTCGATGACAAAAGCTACAATAACA

TCCTTCTACATGTTTGTTTGGAAGCTCTTTTATGCCGAGTACTTGCTTATTCCATTTGTG

AGATGA

>TRINITY_DN10079_c0_g1|m.115 TRINITY_DN10079_c0_g1|g.115 ORF TRINITY_DN10079_c0_g1|g.115 TRINITY_DN10079_c0_g1|m.115 type:complete len:292 (-) TRINITY_DN10079_c0_g1:413-1288(-)

ATGACGGTGGTGAGCGAAATCGTTGATGATATCGTGGTCTCGGAGTCGAAGATGGGATTG

GGGGATTACGTGAGCTTGCCTCAGTCCGACGATCCGGAGCTGGGGAGATTGGAGCAAGCT

CAATGTTCTTCGAGGTGGTGGGGCTCTTGGATCTGGTGGTGGTTAAAGGTGGCTCTTTTA

TGCCTCTTCTTGGCGACGCTTGCTGCGCTCTTCATCATCTTTGTAGGCCCTTTGCTACTC

GATAAGGTGGTTATACCTATACTGGACTGGGAAATGTCAACATTCAGCACCCCAGTCCTT

GGCCTGTTAATGTTTGCTGGTATTGCAATATTACCTGTCCTGCTTTTGCCTTGTGGACCA

TGTTACTGGATAGCTGGATTGACATTTGGCTATGGTTACGGTTTTCTGTTAATTATAGGA

GCAATAAGCATTGCCATGTCATTGTCATTTTTTATTGGTTCTCTCTTTCGTCATAGAATC

AATAGATGGTTGGAGAAGTGGCCCAAGAAAGCAGCTATTGTCAGATTAGCTGGTGAAGGA

AATTGGTTTCATCAATTCCGAGCTGTTACATTGCTTAGAATTTCTCCCTTCCCATTTGTC

ATTTTCAATTATGCAGCTGTAGCTACAAATGTTAAATACTTCCCTTACATATGTGGATCT

CTTGTGGGGACAGTGCCTGAGACTTTTATGACGATCTACAGCGGGAGACTGCTCCGGACA

TTGGCAGACATAACTACCGAGGGTGGCTTCATGCCGGTGGAACAGATCATCTATAATGCA

CTTTGTTTTGGTGCTGGTGTAGGTGTGACCGCGGCCATCACTATCTATGCCAAGAGGACT

CTTCAAAGTCTCCAAGCCAAGGAAGAGCTAAGCTAG

>TRINITY_DN1007_c0_g1|m.116 TRINITY_DN1007_c0_g1|g.116 ORF TRINITY_DN1007_c0_g1|g.116 TRINITY_DN1007_c0_g1|m.116 type:5prime_partial len:102 (-) TRINITY_DN1007_c0_g1:124-429(-)

CCAAAGACACCCCCAATACTGGGACACCACCACTCAAATCACACATCCAATCTATTCACT

GACAACATGGCCTCCGGGACTCAATCTCCTCCCCTCGAAGCGACGGGCGAACATCAGCCC

ATCGGTGCTGCCAACAAGGCAGGCGAGCCTCTGAAGGAGGGCGTCAAGGACTCCGTTCAG

CGGAAGGAATACTTTGGCGGTGACAAGTCTGCCAAAGACACCCCCAATACTGGGTCTGGT

ATTTCTAGCGAAGCCATCGCTGAAGCCGAAAATTCTGCACGCAACGTTGAAAACAAAGGA

CACTAG

>TRINITY_DN100824_c0_g1|m.118 TRINITY_DN100824_c0_g1|g.118 ORF TRINITY_DN100824_c0_g1|g.118 TRINITY_DN100824_c0_g1|m.118 type:complete len:174 (-) TRINITY_DN100824_c0_g1:645-1166(-)

ATGGCGTCCGAGAAGACCAGCAACCCCATGCGGGAGCTCAAGATTCAGAAGCTCGTTCTG

AACATCTCCGTCGGCGAGTCTGGTGACAGACTTACCCGTGCCGCCAAGGTGCTCGAGCAG

CTTTCTGGTCAGACCCCCGTCTACAGCAAGGCCCGCTACACTGTCCGTACCTTCGGTATC

CGACGTAACGAAAAGATCTCTGTCCACGTCACCGTCCGAGGCCCCAAGGCCGAGGAGATT

CTCGAGCGTGGCCTCAAGGTCAAGGAGTACGAGCTCCGCAAGCGCAACTTCTCCGAGACC

GGCAACTTCGGCTTCGGTATCAGCGAGCACATCGATCTTGGTATCAAGTACGACCCCTCG

ATCGGTATCTACGGCATGGACTTCTACTGCTGCATGACCCGACCTGGTGAGCGCGTCACC

CGCCGACGACGCACCAAGAGCCGCATCGGTTCCGGCCACCGCATCAAGCGCGAGGAGACC

GTCAAGTGGTTCAAGTCTCGCTTCGACGGTATTGTCCGATAA

>TRINITY_DN10082_c0_g1|m.119 TRINITY_DN10082_c0_g1|g.119 ORF TRINITY_DN10082_c0_g1|g.119 TRINITY_DN10082_c0_g1|m.119 type:5prime_partial len:250 (-) TRINITY_DN10082_c0_g1:375-1124(-)

AATGGAGTGCGAGCCCTGATGTTAGACACCTACGACTTTGATGGTGACGTGTGGCTTTGC

CATTCAAGTGGAGGGAAATGCCACGATGTAACTGCATTTGAACCGGCTATTGATACATTG

AAGGAAATAGAAGCCTTTCTTTCAGCAAATCCATCTGAAATTGTTACATTGATACTAGAA

GACTACGTTGAGGCTCCCAATGGTTTGACAAATGTCTTTAATGCGTCAGGGTTGTTGAAA

TACTGGTTCCCTCTCTCGAGGATGCCCCAGAACGGCCAAGATTGGCCACCGGTAAGTGAT

ATGGTTGCGAACAACCAACGCCTCATTGTATTCACATCAATCAGATCAAAACAAGATACC

GAAGGGATTGCCTATCAATGGAACTTCATGGTTGAAAACCAATATGGTGATGGTGGAATG

AAAGACGGAGAATGCTTTAACCGTGCTGAATCTGCCCCTCTGAATGACACGAGCAAACCC

CTAGTTCTGGTGAACTATTTCCCTTCAATACCGGTGAAACCCTTGACATGCGAGCACAAC

TCAGAAGCGCTCGTAAACATGCTTCATACTTGTTATGGCGCTGCCAGCAATCGTTGGGCT

AATTTTGTGGCCGTAGATTTTTACAAGAGAAGTGAGGGAGGAGGGGCGTTTCAAGCAACA

GACATGCTTAATGGGAGGCTGTTGTGTGGGTGCGATGATGTTCATGTGTGCGCGGCTGGT

TCTACTTCTGGAGCATGCACTCCACCATAG

>TRINITY_DN10082_c0_g2|m.120 TRINITY_DN10082_c0_g2|g.120 ORF TRINITY_DN10082_c0_g2|g.120 TRINITY_DN10082_c0_g2|m.120 type:3prime_partial len:190 (-) TRINITY_DN10082_c0_g2:3-569(-)

ATGGGTGTTCTCAGAGTCAGCTCTTTGATGATAATCTCAATGGCCCTTCTTGGCTTTGCA

GCTGCCTGTTCCAATGGAACCTGCAAGCTTCTTGATGGTTGCTCTTCTGATGGAGATTGC

GAGGCTGGATTGTTCTGTTCTTCATGCCTAGAAGGGTTTTCTGGCTCAAGGTGCATTCGG

TCTACTATCACTGATCAATTCAAGCTTGTGAATAACTCATTGCCTTTCAACAAGTATGCA

TATCTTACTACACATAATGCTTACGCAATTGAAGGGGAACCATCACATACTGGAGTTCCT

CGTGTTACCTTCAACAACCAAGAGGATACTGTTACTCAGCAATTAATCAATGGAGTGCGA

GCCCTGATGTTAGACACCTACGACTTTGATGGTGACGTGTGGCTTTGCCATTCAAGTGGA

GGGAAATGCCACGATGTAACTGCATTTGAACCGGCTATTGATACATTGAAGGAAATAGAA

GCCTTTCTTTCAGCAAATCCATCTGAAATTGTTACATTGATACTAGAAGACTACGTTGAG

GCTCCCAATGGTTTGACAAATGTCTTT

>TRINITY_DN10084_c0_g1|m.122 TRINITY_DN10084_c0_g1|g.122 ORF TRINITY_DN10084_c0_g1|g.122 TRINITY_DN10084_c0_g1|m.122 type:complete len:104 (-) TRINITY_DN10084_c0_g1:184-495(-)

ATGCCAATGTTGTTGCTTTCGTTCTTGACGTTGTGTCGCTGCGTAGTGAGCTCGTATGTA

TTGTTGCTGTTGTTGTTGTTGTTGCTGTTGGGTTGTCGTTGTTGTGTTGTGCAGAGTGAT

TATCGCCGTGTGGGGTGTTGTTGTGGCAGTCGGTTGTGTGGTTGCTGGGAGGCGTTTGTT

GTGTTGTTGGTTGTGGAGTTGGTGGTTCCCGTTGGTTGGTTTGCGGCGTGGTGGTGTGTT

GTTGTTGTTGTTTTTATCGGAGCGTTGAGGGGTGTGTGTCGTGTACTCGTATGGGCTAGT

TGCTACCGTTGA

>TRINITY_DN10084_c2_g1|m.123 TRINITY_DN10084_c2_g1|g.123 ORF TRINITY_DN10084_c2_g1|g.123 TRINITY_DN10084_c2_g1|m.123 type:3prime_partial len:187 (+) TRINITY_DN10084_c2_g1:264-821(+)

ATGGCATCTTCAGCTGGGAGTTTAGAGACTTCAGCGAATTCACTCCCCAATTCATTTTAC

TTCTCGAGCTCATTTCCGACGTCCTTCTCCGAGCTGCTCGCCGGAGGAGGAGGCGGCGGA

GGCGGAGATGGAGATGAGGAGGAGAACGAGAGGATGAGTGGCAGCAACAGAGGAGGAGGA

GTTGGGGTGCCAAAGTTTAAGTCTCTGCATCCACCCTCGCTGCCGATATCGCCGTCTTCG

TACTTTTCCATCCCGGCCGGGCTCAGCCCTGCTGAGCTCCTCGACTCCCCTGTTCTTCTC

TCGTCTTATTATAACATCTTGCCGTCTCCAACAACAGGAACAATCCCTGCACAATCTTAC

AACTCCAAGCAGAACTCGACAAGCTACGTCAAAGAGGAACAAGGAGCCTACTCCGATTTC

TCTTTCCAAACCAGCGCCGCAAGGCCCGAGAGCTTCAATCCCTCTTCGCAACAGAAGTGG

AGCAGCAGCAGCAACAACAACAACAGCAACAATACGTACGAGCTCACTACGCAGGGACAC

AACGTCAAGAACGAAAGC

>TRINITY_DN100865_c0_g1|m.125 TRINITY_DN100865_c0_g1|g.125 ORF TRINITY_DN100865_c0_g1|g.125 TRINITY_DN100865_c0_g1|m.125 type:5prime_partial len:500 (+) TRINITY_DN100865_c0_g1:1-1500(+)

TTTTCGTCTTCTCCCACAACTCACTCGCATTCTCCACCTCTCCACCCCTCTGCTTCGGCA

TCCATCTTACCTGTACTCCCTCTCGATCCTCTCTACGACGTTTACGTCCCTAAACCAAGC

TCTCTCCACCTCTGCAAGCCTGTCGCACGTCACCGACGACAGCGTCTCCAACCCTCGCGC

AAACAGTCTCTGACTCGATCCTCCACCCTCGACCTCACACCACCCTCACCCGCGATGGCT

GCCCAGGCTGCTCAGCCCGCTTCCAAGTCGGCCAAGAAGAAGGCCGCCAAGGCCATCGAG

CGAACTGAATCCCCCGCCCCCAGCGTCACCTCCGCCGCCGCCGATAAGACCGGCGACGAC

ACCTTCGAGTCCCCCTACATCAAGGAGCTCCAAAAGAACATTCGCAATGTCAACAAGAAG

ATCACCAACGCTTCCAAGACCGACGCCCTTCTAAGCCAACACGCTGACAAGTCGCTCGAT

GAGCTGGTTGCCACCAAGATCATCAACGCCGACCAGAAGGCCCAGATCCTCAAGAAGCCC

GCTCTGCAGGCCCAGCTCGCTCAGTTCGAGGAGCAGCTGACGCAATACCAGAAGGTCGAC

GAGCAGTACCGGACACGCGCCGCCGCCGACAAGGCCGAGTGGGAGAAGGGCCTCGAGAAG

GCCAAGGCCGATGCTGTTGCTGAGGCCAAGGAGGAGGCCAGCAAGTCTCTGAACGACAAC

CTGCTGGTCCTGTCGCAGTTCCTGCGACTTGCTGCCTACCGCCGTGAGGAGGCTCAGGAC

CCCGAGTCTGACGAAAGCCAGGCTATCGAGGGTGTCCTCCTCGCCATCTACTCTGGCGAC

CAGAATGCCGTTCAGTCCATGCTGAAGCTTGTCAACGGCACTGAGGACCGGATCTTCAGC

GTTCCCGGAGAGCAGCTTCAGACCACTTTCTCCAAGGTCAAGTCTTTGGCTCAAGAGTAT

AAGACTCCCTATGACGAAGCCCCGGCTACCGAGGGTGAGGCTGCGCCCGCCGAGGTTGCC

TCGGACCCCACTGTCGCACACGCCTCCGCCACCGAGATCGAGGCTGGCGATGTTTCCGCT

CCCGTCGAGGCTGCTGTCCAGCCTTCTTCCAACGGCCTGGCCAATGCCAGCGTGGCCGAC

GACGCCGCTAACGCCGTCGCCGAGAGCCATTGGGACACCTCTAACCAGGAGCTTTCCGCT

TCTCAGGAATGGGTTGACGTGAAGGCTACTGAGGCTACTGAAGCCGAGGCTGCTACCCCG

GCCCCTGCTGCCAACACTCAGTCTTGGGCTGATGACCACCCTGAACACGCTGCTGAGACC

GCCGCTCCCGCCGACCCCAACGACGGATTCCACCAGGTCCAGAGAAACCGACCTGGCAGC

CACCGCGGACGTGGTCGTGGTGACTGGCGCGGCCGGGGCGGACACCGTGGTGATGGCCGT

GGACGAGGTCGTGGCCACCGCGGTGCCCCCCGAGGCGGCCGCCGCAACGAGGAGTCGTAA

>TRINITY_DN100865_c0_g1|m.126 TRINITY_DN100865_c0_g1|g.126 ORF TRINITY_DN100865_c0_g1|g.126 TRINITY_DN100865_c0_g1|m.126 type:complete len:358 (-) TRINITY_DN100865_c0_g1:451-1524(-)

ATGCCATGTCATGTTAGGGAGCGTTTACGACTCCTCGTTGCGGCGGCCGCCTCGGGGGGC

ACCGCGGTGGCCACGACCTCGTCCACGGCCATCACCACGGTGTCCGCCCCGGCCGCGCCA

GTCACCACGACCACGTCCGCGGTGGCTGCCAGGTCGGTTTCTCTGGACCTGGTGGAATCC

GTCGTTGGGGTCGGCGGGAGCGGCGGTCTCAGCAGCGTGTTCAGGGTGGTCATCAGCCCA

AGACTGAGTGTTGGCAGCAGGGGCCGGGGTAGCAGCCTCGGCTTCAGTAGCCTCAGTAGC

CTTCACGTCAACCCATTCCTGAGAAGCGGAAAGCTCCTGGTTAGAGGTGTCCCAATGGCT

CTCGGCGACGGCGTTAGCGGCGTCGTCGGCCACGCTGGCATTGGCCAGGCCGTTGGAAGA

AGGCTGGACAGCAGCCTCGACGGGAGCGGAAACATCGCCAGCCTCGATCTCGGTGGCGGA

GGCGTGTGCGACAGTGGGGTCCGAGGCAACCTCGGCGGGCGCAGCCTCACCCTCGGTAGC

CGGGGCTTCGTCATAGGGAGTCTTATACTCTTGAGCCAAAGACTTGACCTTGGAGAAAGT

GGTCTGAAGCTGCTCTCCGGGAACGCTGAAGATCCGGTCCTCAGTGCCGTTGACAAGCTT

CAGCATGGACTGAACGGCATTCTGGTCGCCAGAGTAGATGGCGAGGAGGACACCCTCGAT

AGCCTGGCTTTCGTCAGACTCGGGGTCCTGAGCCTCCTCACGGCGGTAGGCAGCAAGTCG

CAGGAACTGCGACAGGACCAGCAGGTTGTCGTTCAGAGACTTGCTGGCCTCCTCCTTGGC

CTCAGCAACAGCATCGGCCTTGGCCTTCTCGAGGCCCTTCTCCCACTCGGCCTTGTCGGC

GGCGGCGCGTGTCCGGTACTGCTCGTCGACCTTCTGGTATTGCGTCAGCTGCTCCTCGAA

CTGAGCGAGCTGGGCCTGCAGAGCGGGCTTCTTGAGGATCTGGGCCTTCTGGTCGGCGTT

GATGATCTTGGTGGCAACCAGCTCATCGAGCGACTTGTCAGCGTGTTGGCTTAG

>TRINITY_DN10086_c0_g1|m.127 TRINITY_DN10086_c0_g1|g.127 ORF TRINITY_DN10086_c0_g1|g.127 TRINITY_DN10086_c0_g1|m.127 type:3prime_partial len:152 (-) TRINITY_DN10086_c0_g1:2-454(-)

ATGGCCGATCTCAGGATTCTCAGCAGCCCCGTGACTGGGATCCCCTCATCGGATGCGGAT

CCGGTACCTGACCCGATCGACGATCCGAAGGTTTTATGTGGTGGAATCACAAGTAGAGAA

GTCAAAGATCGGATCCCAGATGTCTCGGAGAAGTTGCGTCAGGATGTCGAGGTAGTTAAT

ACTCCTGGTTTAACCAGTGCCAGTAAAGATGATCATCCAATAACCATTCATGATGATGGT

GCATTGGTAGATTCTGATCCCAAGCATAAGCACTCTGAGGCTGATGCCTCAAGCCAAGAT

AAGGTGCTCAAGAAGCCAGATAAAATTTTGCCCTGTCCTCGCTGCAATAGCTTAAACACC

AAGTTCTGCTATTACAACAACCACAATGTTAACCAACCAAGGCACTTCTGTAGGAATTGT

CAGAGATATTGGACTGCAGGTGGGACAATGAGA

>TRINITY_DN100871_c0_g1|m.128 TRINITY_DN100871_c0_g1|g.128 ORF TRINITY_DN100871_c0_g1|g.128 TRINITY_DN100871_c0_g1|m.128 type:complete len:955 (-) TRINITY_DN100871_c0_g1:222-3086(-)

ATGGGTTCACCTGGCCAACTTCTCTTCTTGTTGCTGAGCTTTGCAGCTGTTCAATTCAGT

TCTTGCATCACATATGGTCGAGATGCTGCTGCTCTCCTGTCCTTGATGGATCAGATGAAG

AACACGCCGCCGAATTGGGGGCAGTCCAACGATCCTTGTGGCGCACCGTGGGATGGGGTT

ACATGCAACAATGATTCGAGGGTGACGGCGCTGAAATTGTATAGCATGGGTGTCGAAGGT

ACCCTGAGCAGTGACATTGGAAGCCTGACCGAACTTATAACCTTGGACTTATCGTACAAT

AACAAGCTTGGTGGTCCACTAACACCTGCTATTGGAGAGTTGAAGAAGCTTACTGATCTG

TTCTTGATCGGTTGCAGCTTCAGTGGTACAATCCCAAATGAACTAGGCAATTTAGCACAA

CTCGTTTATTTGGCTTTAAACTCAAACCAATTCACAGGACAGATACCTTCTTCTCTCGGT

AACCTCTTTAATCTCTTCTGGCTAGACCTGGCAGACAATCAGTTGACTGGATCTCTCCCC

GTCTCAACAAACACAACCCGTGGTTTAGACCAGCTTGTCAAAGCACTACACTTCCATTTA

AATCAGAATCAGTTGACTGGTCCAATCCCTGAAAATCTTTTCAGCTCTAAAATGGCACTG

GTTCATATTCTTCTGGATAGCAATCGACTTACTGGGAGTATTCCGGCATCTGTTGGACTT

GTGCAAAATCTTACCATTATTCGACTTGATAATAATGCACTCAATGGCTCAGTTCCGTCA

AGTATTAATAATCTTACACGCCTTAATGTACTTAATTTAGCGAACAACATGCTAATTGGC

CCACTTCCAAATTTGACTGGAATGAACGTGCTCAATTCTGTGGACTTGAGTAACAATTCA

TTTGATTCTTCAGAAGCTCCTGCTTGGTTTTCTGAATTAGGATCCCTTACCTCTTTGGTG

ATAGAATCAGGACAACTTCATGGTGAAGTGCCACAAAAGCTCTTCAGCTTTCCACTGCTA

CAGCAAGTGATACTAAAGAACAATCGATTTAACGGAAGTCTTGACTTGGGCAGCAACATC

AGCACTCAACTAAAGACTGTGAATTTCCAGAATAATTCTCTTACCTCCGTTGAACTTAGC

TCCATCTATAATAATATGATAATGCTCACTGGAAACCCAGTATGTAGCAATGTTCTTCTT

TCCCAAACAAGCTATTGCATCCCTCAGCAAGAAACATCTCCACCTTATTCCACCGACACT

ACCAAATGCGGTTCCCAGTCATGCTCTCAGGAATATAACTGTTCCAATCCGTACGAGGGG

GTTATGGTTTTCAGAGCACCTTTCTTTCAAGACGTGAAGAATGATTCGGTGTTTGAATCA

CTAAAAGAGAGTGTATGGAAACAATTTGGATCTAAACTGGGATCAGTATCTCTTGAAAAT

CCCTTCTTTGATAGTAACTCTTATCTTCTAGTGAAGTTGAAACTCTGTCCATCAACAGGC

AGTTACTTCAACCGGTCACAGATTCTTATTGAGCTTGATCTAAGCAGCCAAAACTATATA

CCTCCGATATTTTTTGGTCCATACTATTTCAATGCATCTTCATATCCTTTCCCAGGTGGT

TCCGGTATAAAGAAAGGTCTGATTATTGGAGTTGTAGTTGGCTGTACATTTCTTGTCCTT

GGACTTGTAGCTATTGGAACATATGCCTTCCGTCAAAAGAAAAGGGCTGAAAGAGCCATT

GAAATAAGCAACCCTTTTGCGTCATGGGAAGCAAATGAAGATACTGGTGGCGCACCAAAG

TTGAAAGGAGCAAAATGTTTTTCTTTCGATGAACTGAAGAAGTACACCAATAATTTTCCA

GAAATCAATGAAATTGGATCAGGAGGCTACGGCAAGGTCTACAGAGGGATGCTTCCAGAT

GGGCAGGTAGTTGCGATCAAGAGGGCGCAAGTAGGGTCTAATCAGGGTGGCCTCGAGTTC

AAGACTGAAATTGAATTGCTCTCAAGGGTCCATCACAAAAACTTAGTAGGTCTTGTAGGC

TTTTGCTTTCAGCAGGGAGAGAGAATACTCGTCTACGAGTACATCTCCAGTGGAACACTA

AGGGATTGCTTGTTTGGCAAGGCCAATATTCAACTTGACTGGAAAAGACGGCTCTGGATT

GCTCTTGGATCCGCCAGAGGACTAGCTTACCTTCATGAACATGCATATCCACCAATTATT

CACAGAGATATTAAGACCACCAATATACTTCTGGATGAGAATTTAAATGCAAAGGTTGCA

GACTTTGGTCTCTCAAAGTTGGTATCAGACTGTGAGATCGGATATGTTTCTACTCAAGTT

AAGGGAACGTTGGGCTATCTGGATCCTGAATACTACATGACTCAACAATTGACCGAGAAG

AGCGATGTTTATAGTTTTGGGGTAGTAATGCTTGAATTGATATGTGCTAGGCCACCATTA

TACAAGGGGCAGTACATTGTTCGCGAAGTGAAGGAGGCAATCAATCAGAATGATGAAGAG

TATTTTGGTCTGAAAGACATAATGGATGTTCTCATCCGTGATGCCAAATATCTCATTGGT

TTCAGAAGGTTTGTAGAGTTGGCTTTACAATGCATTCAAGACTCTTCTGTAGATCGTCCA

ACAATGAGTTACATTGTGAAAGAAATCGAGAGAATTTTGCAAGATGATGGAATAAATACA

AGCTCAGCATCTTCATCTGCCAGAGATTTTGGAGTCAAAAGGGCGGCTCCTCAGTATGAT

TTTAGTGACTCGACACAGGGAACAGATACACAGGGAACAGATACGTCCAGTAATTCGTTC

AAGACTTTCTTATTCTCACCAGCCATAACTCCAAATAGCAGATAA

>TRINITY_DN10087_c0_g1|m.129 TRINITY_DN10087_c0_g1|g.129 ORF TRINITY_DN10087_c0_g1|g.129 TRINITY_DN10087_c0_g1|m.129 type:complete len:414 (-) TRINITY_DN10087_c0_g1:704-1945(-)

ATGGAATCGGCAACGGCGCTTCGATCCGCTACGGCTTCTCTCAAGTTTACAGATCTCGGT

TCGAGATCTTGCCTTGTAGCCGCTCGTCGCCCTAATTTTGTCTCCAAATCCAATCTCTCC

AATAGATTTCTTAAGGGCCAATGCTCGTTCTCTCTTTCGAATCGAGGGCAGAGGAGATAT

GCTATTATGACATTTAGAAGAAATTCTGGAATTAGAGCTTCCTCTAATTCATCCTCGAAT

TATTCGGAGTCTTCCTCTCCTATTGCTCCGCTTCAATTGGAATCACCAATTGGTCAGTTC

CTATCTGAGATTCTCCACAGCCATCCTCATCTTATTCCTGCTGCCGTTGATCAACAGCTC

GAGCAGCTTCAGATTGACCGAGAATCCCAGAAGAAAGATAATTCGCCTTCTTCAGGCACT

GATCTCGTCTTATACAAGAGGATTGCTGAGGTGAAGGCCAATGAGAGGAAAAGAGCTCTA

GAGGAGATATTATATGCTTTGGTAGTTCAAAAGTTTGTGGAAGCTGGTGTATCATTGGTT

CCCTCTTTATCTCTGTCTGATCCATCGGGAAGGGTAGATCAACGGCCATCCGAGGAAGAC

AGGCTTAAGAGGCTTCATTCTTCAGAGGCTTACGAGATGATCAAGAACCACCTTTCCCTT

ATTCTGGGTCAGCGCTTGGATGATGGAAGCTCCATTGCTCCAATTAGCAAGCTCAGGGTT

GGGCAGGTGTATGCAGCCTCCATCATGTACGGCTACTTCCTCAAGAGGGTGGACCAGAGA

TTCCAGCTCGAGAAGTCCATGAAAACCCTCCCTTGGGGGGTGGAAGAGGAGAACACAACC

ATCAAACAATCGATGCCTGACGAGTCTCGGCCCTCTGTGGGAAATCCCCGTCATGAAGTC

TCTTCTTGGTCTTATCCTAGCCCCGGTGGGTTTGCTGATGGGATCAACCCATACCGGTTG

CGCACTTATGTCATGTCATTCGATCCAGAAACACTTCAGAGATACGCTGCCATAAGGTCG

AAGGAAGCCTTTGGCATCATCGAGAAGCATACAGAGGCATTGTTCGGGAGGCCAGAGATT

GTCATCACTCCTCAGGGGACTATTGATTCTTCCAAAGATGAGCTTATCAAGATTAGTTTT

GGTGGATTAAAGAGACTCATATTAGAGGCCGTAACTTTTGGTTCTTTCCTATGGGATGTT

GAAAGCTATGTGGATTCCCGTTACCATTTTGTCGCTAATTAG

>TRINITY_DN1008_c0_g1|m.130 TRINITY_DN1008_c0_g1|g.130 ORF TRINITY_DN1008_c0_g1|g.130 TRINITY_DN1008_c0_g1|m.130 type:complete len:366 (+) TRINITY_DN1008_c0_g1:227-1324(+)

ATGGCGGCGATTAGATCGATTTGCTGCTTTGAGACGGTTCAGATGCTGCTTCTGCTGCTG

ATTTTTATTTTGGCTGCGGACATCCAACTGTTGCAGGTGGTTGCTGTGAAACAAATCCCT

CAAGATAGGACAGATGCCAGTATCCTTCAGGACTCCATCGTACAAAAGGTTAATGATAAC

CCTAATGCTGGATGGAAAGCTTCAATGAACTCTCGCTTCTCCAATTATACATTCGGACAA

TTCAAATATATTCTCGGAGTGAAGCAGATGCCTCAAAATGCCTTGGAAGATATTCCTGTA

AAATCTTATCGCAAATCTCTAGATCTGCCTAAACAGTTTGATGCTAGGACAGCTTGGCCT

CAATGTAGCACTATTGGAAGGATACTTGACCAGGGACACTGTGGTTCTTGTTGGGCTTTT

GGTGCAGTGGAATCACTGTCGGATCGTTTTTGTATCCATTTTGACATGAACATATCTCTA

TCTGTTAATGATCTCTTATCCTGTTGTGGTTTTATGTGTGGAGATGGTTGTGATGGAGGG

TATCCTATACATGCATGGCAATACTTTGTGCAGAATGGTGTTGTTACTGAAGAGTGTGAC

CCGTATTTCGATGACATTGGATGTTCTCATCCAGGCTGTGAACCTCTATATCCGACACCA

CGATGCGAAAAAGAGTGCAGAGTCAAGAATCTTCTTTGGGTGAAAACGAAGCATTTCAGT

GTGGATGCTTACTATGTAGGCTCCGATCCACATGATATCATGGCAGAGGTCTATAAGAAT

GGTCCTGTGGAAGTTGCCTTCACAGTTTATGAGGACTTTGCTCACTATAAATCAGGGATC

TACAAACACATTACTGGCGATTACATGGGTGGCCATGCTGTGAAATTGATTGGTTGGGGG

ACTAGTGAGGATGGTGTAGACTATTGGCTCCTTGCAAATCAGTGGAATAGAGGATGGGGT

GATGATGGTTACTTCAAGATTGTGAGGGGAACAAATGAATGCGGGATTGAAGAGGAAGTT

GTTGCCGGGCTGCCTTCTTCGAAGAATCTAATCAAGAACTTTGCCAGCACAGATATGCCT

GGTTATTCCAGTGTTTGA

>TRINITY_DN1008_c0_g2|m.131 TRINITY_DN1008_c0_g2|g.131 ORF TRINITY_DN1008_c0_g2|g.131 TRINITY_DN1008_c0_g2|m.131 type:3prime_partial len:145 (+) TRINITY_DN1008_c0_g2:392-823(+)

ATGGCGGCGGTTAGATCGATTTGCTGCTTTGAGACGGTTCAGATGCTGCTTCTGCTGCTG

ATTTTTTCCGCGGAGATCCAAGTGTTGCAGGTGGTTGCAGTGAAACAAATCCCTCGAGCC

AGGACGGATGCCAATATTCTTCAGGATTCTATTGTGCAAAAGGTTAATGCTAACCCTAAT

GCGGGATGGAAAGCTTCAATGAACTCTCGTTTCTCCAATTATACGGTCGGGCAATTCAAA

TATATTCTTGGAGTGAAGCAGATACCTCAATATGCCTTGGAAGATATTCCTGTAAAATCT

TATCATAAATCTCTATATCTGCCTAAACAGTTCGATGCTAGAACAGCATGGCCTCAATGC

AGCACAATTGGAAGGATACTTGATCAGGGACACTGTGGTTCTTGTTGGGCTTTTGGTGCA

GTGGAATCACTG

>TRINITY_DN10090_c0_g1|m.132 TRINITY_DN10090_c0_g1|g.132 ORF TRINITY_DN10090_c0_g1|g.132 TRINITY_DN10090_c0_g1|m.132 type:complete len:692 (-) TRINITY_DN10090_c0_g1:211-2286(-)

ATGTCTTCACCTCCGAACGTGCCCAGATGGACCCCGAGCCCGAGCCCGAGCAAGCCGATA

TTGGGCCGGGGCGCCGGAGACGAGAGCCCATTCCCGTTCAGCAGCTCCCCGTCGCCCCCG

CCAGCGACGATCCATCACCGTATCAACGCGAACTACGTCGAAGCGGAGGAACCGCCGGAG

CTGGAAATGGGGAGGGTACCCCGAAACGGAGGGGTATCCCTGACATGGGAAGACCTGTGG

GTTACCGTTGGAGATGGCAGAGATGGGCGGGTGCCGATTCTTAGTGGGCTCACCGGGTTT

GCTCGGCCCGGTGAGGTCCTGGCCATCATGGGACCATCTGGCTGTGGCAAATCTACTCTC

CTCAATACATTGGCAGGAAGATTAGGGTCCAACATTAGACAAACGGGAGACATTCTGATC

AATGGCCGGAAACAGTCCCTCACCTTCGGCACATCCGCCTACGTCACCCAAGACGACGTC

CTCATGACGACTCTCACCGTCCAAGAGGCCGTCTGCTACTCGGCCGAGCTCCAGCTGCCG

TCGACCCTGTCAAAATCCCAGAAGATCGCCCGAGCAGAGGAAACGATTCGAGAAATGGGG

TTGGTCGCCGCGAAGAACACCAGAGTATCAGGCATAAGTGGAGGCCAGAGAAGACGAGTG

AGTATATGCTCGGAGATATTGACTCGGCCAAAACTCTTGTTTCTCGATGAGCCGACCAGC

GGTTTAGATAGCGCAGCATCGTACCATGTCGTCAGTCGGATCGTCAAGCTAGCGCGCCGA

GAAGGGATGACGGTGATCGCTTCTATTCACCAGCCGAGTGGAGAGGTGTTTAATTTGTTC

AGCAGTCTCTGTTTGCTTGCATATGGCAAGACTGTCTATTTCGGGCCAGCATCTTCGACA

AGCGAGTTCTTTTCGTTGAATGGTTTCCCATGTCCATCGCTGACAAACCCTTCGGATCAC

TTCCTGAGGACAATTAACAAGGATTTCGACATGGATATTGAAGAAGGCCCAGATGCCGAA

CTGACAACCACTGCACAAGCAATCGAAACCCTTGTGAAGTCCTACAGATCTTCGACTAAT

TCACAAACAGTGAAGCTACAAATTGCTGAAATACATGAAATGGGAGGAGGTCAGGTCATG

AAAGGGAATCAAGCTAGCCCCTTGACACAATCCGTTGTGCTCACCAGGAGATCGTTTGTT

AACATGTACAGAGATCTTGGTTACTATTGGTTGCGCTTAGCAATTTACATAGCACTCTGC

CTCTGCGTCGGAACAATTTACTATGACATTGGTCATAGCTATGGCTCGATTCAGGCCAGA

GGCTCTATGCTCATGTTTGTAGCTGCATTTCTCACTTTTATGGCGATTGGCGGGTTTCCA

TCTTTTGTCGAAGACATGAAGATTTTTGGACGAGAAAGGCTAAATGGGCACTACGGCGTA

GCCACTTTCGTGATCAGCAGCATCCTATCCTCCACTCCATACCTGGTCCTCATCTCGGTA

ATCCCGGGAGCCATAGCTTACTATCTAGTCGGCCTCCAGCGCCAATTCGACCACTTCCTC

TACTTCATCCTCGTCCTGTTCATGTGCATGATGCTAGTTGAAAGCCTGATGATGATTGTC

GCCAGCATTGTTCCCGACTTCCTCATGGGCATCATAACCGGCGCAGGCATCCAGGGGGTG

ATGATGCTCAACGGAGGGTTCTTCCGGCTGCCCAACGACCTCCCGAAGCCGGTGTGGCGA

TACCCAATGTACTACATTGCCTTCCATAAGTATGCCAACCAAGGGTTCTACAAGAATGAG

TTCATTGGGCTGACATTTCCTAACAACCAAGCTGGGGGGCCTGCTACGATCACTGGTGAG

GAGATTCTGAGAAATTTATGGCAAGTGGAGATGGGGTATTCTAAGTGGGTTGATCTGGCT

ATTTTGTTTGGGATGGTGGTTATTTATCGGTTGATGTTCTTCATGATCGTGAAGATTGTT

GAGAAGGTGAAGCCAATGCTGAAAGGGCTGATGGCCAAGGGAAAGACACAGTCTGGTCAT

GGCATAGTGCAGCTATCTTCAGCATCATCACCATGA

>TRINITY_DN100931_c0_g2|m.135 TRINITY_DN100931_c0_g2|g.135 ORF TRINITY_DN100931_c0_g2|g.135 TRINITY_DN100931_c0_g2|m.135 type:5prime_partial len:206 (-) TRINITY_DN100931_c0_g2:66-683(-)

TCGATCCCGAACCTCCCAACAGCAGCAGCAGCCATGGCCCCCGCCGAGCACGACCACGTC

CACGACCACGCGCACGCGCACGAGCACTCGCACGAGCACGCGCACGACCACTCGGACGCG

GACTCGGACGACGAGATCCCGGCCCTGGAGGAGGCCGGCGACGCCAACTCGGCCGAGGCC

GGCAAGCACAACCGCTCGGAGAAGAAGTCGCGCAAGGCCATGCAGAAGCTCGGGCTCAAG

CCCGTGGGCGGCATCATCCGCGTGACCATCAAGAAGAACAAGAACGTGCTCTTCGTCATC

TCCAAGCCCGACGTGTTCAAGAGCGCCGTCAGCGACACGTACGTCATCTTCGGAGAGGCC

AAGATCGAGGACCTCAACGCCCAGGCCCAGCAGCTCGCCGCCCAGCAGTTCAAGGCCCCC

GAGCCCGCTGCTGCTGACGCCGCCGCTGACGCCGCCGAGGACGAGGAGGACGACGCCGAG

GTCGACGAGTCGGGCATCGACGCCAAGGACATCCAGCTCGTCATGAGCCAGGCCGGCGTC

AGCAAGGCCAAGGCCGTCGCCGCCCTGCGCTCCAACGACAACGACATTGTCAACGCCATC

ATGGAGCTCACCATGTAA

>TRINITY_DN100931_c0_g2|m.134 TRINITY_DN100931_c0_g2|g.134 ORF TRINITY_DN100931_c0_g2|g.134 TRINITY_DN100931_c0_g2|m.134 type:internal len:229 (+) TRINITY_DN100931_c0_g2:1-684(+)

CACGAGCGGATCAAGCCTTCGATTCCATTATGGCGGCCGCCGATAAACTACCCTAGACTG

CAGACTTACATGGTGAGCTCCATGATGGCGTTGACAATGTCGTTGTCGTTGGAGCGCAGG

GCGGCGACGGCCTTGGCCTTGCTGACGCCGGCCTGGCTCATGACGAGCTGGATGTCCTTG

GCGTCGATGCCCGACTCGTCGACCTCGGCGTCGTCCTCCTCGTCCTCGGCGGCGTCAGCG

GCGGCGTCAGCAGCAGCGGGCTCGGGGGCCTTGAACTGCTGGGCGGCGAGCTGCTGGGCC

TGGGCGTTGAGGTCCTCGATCTTGGCCTCTCCGAAGATGACGTACGTGTCGCTGACGGCG

CTCTTGAACACGTCGGGCTTGGAGATGACGAAGAGCACGTTCTTGTTCTTCTTGATGGTC

ACGCGGATGATGCCGCCCACGGGCTTGAGCCCGAGCTTCTGCATGGCCTTGCGCGACTTC

TTCTCCGAGCGGTTGTGCTTGCCGGCCTCGGCCGAGTTGGCGTCGCCGGCCTCCTCCAGG

GCCGGGATCTCGTCGTCCGAGTCCGCGTCCGAGTGGTCGTGCGCGTGCTCGTGCGAGTGC

TCGTGCGCGTGCGCGTGGTCGTGGACGTGGTCGTGCTCGGCGGGGGCCATGGCTGCTGCT

GCTGTTGGGAGGTTCGGGATCGAC

>TRINITY_DN10095_c0_g1|m.137 TRINITY_DN10095_c0_g1|g.137 ORF TRINITY_DN10095_c0_g1|g.137 TRINITY_DN10095_c0_g1|m.137 type:complete len:432 (-) TRINITY_DN10095_c0_g1:325-1620(-)

ATGGGAAGGCTGAGAGCCAAGCAAGACTACGAGAGCCTCCGAAATGCTCGAATCACTCGA

AACAAGGCTAGACTGGAGGAGGCTCTTGGGCTCAACCGCAGTGGCGGAGAACTCGCTTCC

ATCGTTTCTACGGCCAAATCGATTAAAAGGGTTAGAGGATCCATCATGGGTTCAGCAAGT

ACTCAAGTTCGTCGCTCAGTTCGATTGAACGCCGTATCTGGTGACCATGATCCATCATCT

CCCCAAGATTCTGGCAAAAGCGAAGTAGAGAAAAGAATCGATGATCATGTTTCAGAAATT

GAGGGAAATGAAAGCCTTCGAAATGCAGGAATATCTGAGAACAAGAAGGCTAGAATGCCA

TCTCTCGGGCTCGACAAAAATGTTAGAGAGATCCAAATTACTAAGAAATTGAAGCCAGAT

TCTGACTTTGATTCCTTATCTCCTAAAGACCCTTCGAAAGAAATCGGGGAACAGGAGGAG

GAAAGATCCATGGATTCCTGCAAAAGTAGAGATAATGCGAGAACTGATGATCAGGTCTCA

GGGTTCTCCGAGTATGAAAGCCTTCGAAATGCCCGAATCTCAGAGAACATGGCTAGATTG

GCTGAACTTGGGCTTGATCGGAAACTCAGAGAGCTTCATGAACTCTTTTTTTCTGCCAAA

TCCAGTGCCAAAACCAACAAAACGGCCAAGAAAAAGAAGCTGGATTTAGCGACCCCACGC

CGTCAATCAGACCGTTTGAAGGGCAAGTCTGGCGGTTCTGATGCCTCAACTCCCCAAGCT

GCATCTGTTGAAAGACAAGTACTCTCGGGGCGGTCAGTATGTTCTGAAAGGGTGAAGAGG

AGACTGTTTTCTCATGAGGAGCGATGTGGGAGTAAAGGGAGAGGAAGCATCTATGATTCT

ATTATAGGAATTTGTTGTCACTTTTGCAGGCAGAAGAAGTTGTGCGGGGAAGAAGACTGC

AAACGCTGTGGTGACAGTGATGCGGATCAGCCATGCATAGGAAAGACAGAATGTTCAGCC

TGCCACTCTTCAAGAGGTGTTTTGTGCCGTGCTTGCCTCATGGTTAGATATGGTGAAGAT

ATAGAAGAAGTCAGAAAGAAGAAGGATTGGATGTGCCCACATTGTATTGAAGAGAATGGA

ATCAACCCATATTGGATCTGCAACAGTTCTCTGTGCTTGAAAAAGAGAAAGATGGTTCCC

ACTGGAATTGCGATCTACCATGCGCGTCAGGAGGGGTATGAATCTGTGGCTCACCTGATA

ATGGACAAGCTGAAGCGCACGGCACGCTGCCAGTGA

>TRINITY_DN10097_c0_g1|m.138 TRINITY_DN10097_c0_g1|g.138 ORF TRINITY_DN10097_c0_g1|g.138 TRINITY_DN10097_c0_g1|m.138 type:5prime_partial len:1311 (-) TRINITY_DN10097_c0_g1:532-4464(-)

GGTTCTTCTCCAATGATGAGTCCTGTACCACATGAAGGAAAACAACCCTTGAATATCACA

GATGCTCCATTGTCTTCTAATCCAGGACTTTCTGGATCTTCTCCAGCGGCAAGTCCTGTG

CCACATAGCAAACATCCCCCCTCAAGTGCACATGCTCCTCAAGCTTCACATCTCCAACCT

TCAGGATCACCTCCAGTGATAGACCCTATACCACATGAAGGGAACAAGACCTCATATATT

ACGCATTCACCTGCACCTTCAAGTCTCCATCCTTCAGAATCCCCTCCAGGGATAAGCCCT

ACTCCACATGAAGTGAAGGAGCCATCAAACATCACACACTTGCCTCCTCCTTCACATCAA

CTTCCTTCGGGATCTTCTCCGGTGACGAGTCCTGCACCACCTCATGTGAAGGAATCATCA

AACATCACTGATTCTCCTCTTCCTTCACATCCACTTCCTTCAGAATCTCCTCCAATGACA

AGCCCTACACCCCCTCAAGTGAAGGAGCCCTCAAATGCTGCACATTCTCCTCTTCCTTCA

GAATCTTCTCCAATGCCAAGCCCTGCACCCCCTCAAGTGAAGGTGCCCCCAAATGCTACA

CATTCTCCTCTTCCCTCAGAATCTTCTCCAGTGACAAGCCCTACACCCCTCCAAGTGAAA

GAGCCCGCAAATGCTACACATTCTCCCCTTCCTTCACACCCCCTTCCTCCAGAATCATCA

CCAATGACCAGCCCTACGCCACCTAAAGTAAAGCAGCACCCAAACACTACACATTCTCCT

CTTCCTTTGCATCCGCTTCCTTCAGAATTTTCTCCGGCAATAAGCCCTGTCCCACATGAA

GTAATAGATCCTTCAAATAATACACATGCACCTTTTGCTTCGAATCCTAAACCCTCAGGA

TCATCTCCTTCGACAAGTCCTATGCCACATGGAGTGGAGGAGCCTTCAAACATTACACAT

TCTCCTTTACCTTCAGTTCCCTTTCCCTTTCCCTCGGTATCTTCACCAGTGATAAGCCCT

GCACCATCTGAGGTTAAGGATCCCTCAAGTATTACATATCCACCTTTTGCTTCTGATCCC

AAACATTCAGCAATGCCTCCAATGATAAGCCCTACCGCGCATGAAGTGAAGAATCCCTCA

AACATTACTCATTCTCCTTTGCCTTCGTATCCTCCTCCTTCAGAATTGTCTCCAGCCCCG

GCACCACCAAATGAAGGGAAGGGCCCTTCAAATATCACACATGCTCCTTCAACTTCACAT

CCCAATCCTTCAGGGTCGTCTCCAGTGATGAGTCCTACACCACATGAAGTGAAGGAACCC

CCAAATGTTACAGTCTCTCCTTTGCCTTCTCCAATGACAAGCCCTCCACCACATGAATTG

AAGGATCCATCAAATGTGACTCATTCTCCATTTCCTTCACCTCCCCATTCGTCAGAGCAT

TCGCCAGCTGTAAGCCCCGTGCCACATGGAGTGAAACGTCAATCGAGTGCAACCAATGCT

CCTAGGGCTTCACCTCTTCACTCTTCACTATCTTCACCATTGTTAAGCCCCATGCCTCAT

GAAGTGAAAAATACCTCGAATGTTACCCATGCTCTTTTGCCTTCACCTCCTCATCCGTCA

GAAGGAATTCAGACCCCTGCATTTGCTCCACTTGCTCCGCCTCCAACGTCATCACACCAT

GCAGGTTCAATGATATCTCCAGTTCCTTCACATATTCCTGCAGTATCTCCAAGCAATGAG

GGAAGGATACACGCTCCTTCTCCGCATATTCGAGTCTCTTCTAGTACTCCGGTCCAAGCC

CCTTCGCTTGTAGTACCGAAAAATAACCAAAGGAATCGTGCCCCTTCACTTTCTCCGATC

CAAGCTCCATTTCCATCAGCAAATAGAGAAGTAGCACATTCTCCTTCACCGTCATCAGTG

ATTCCTTCGCAGCATAACAAATGGCCCATTCATCCACCTGTTCTTTCGCCTGGGTTCTCA

AAGAAGCCAAGGGCACCACCACCAAAGCCTGTTTGGGTATTTCCACCCCCACCTCCTAAT

TTGGATTGTAAGTCATTATCCTGCCAAGAGCCTCGTACATATCCTTCTCCAGGATCTGTC

TGCACGTGTGTCATGCCAATTAACGTTGGCCTCCGTTTAAGTGTCTCACTGTACATGTTC

TTTCCGTTGATCTCGGAGCTTGCCCAAGAAGTTGCGTCTGGAATTTTTGTGAAACAAAGT

CAGGTGCGCATCATGGGAGCCAATTCTACTACCGAGGATGCTGAGAAGACTTCTGTGTTA

ATTGATCTAGTGCCATTTGGTCGCTCTTTTGACATGAATACGGCGTTATTAATTTTTGAG

AGGTTCTGGCATAAGCAGGTTCTCATAAATGCCATGTTTTTTGGGGACTACGATGTATTG

TACGTTCGCTATCCAGGCCTCCCTCCATCTCCACCAGTGGCACCTGGAGATGTAAATGTT

GATGGTGGGTTAGAGAACGGTGAAAATGCAAGGAAAATGAAACCCCTTGGAGTTGACGTG

GGAAAGAGAAAAGAAAAACTCAGTGGCGGTATAATAGCTATTGTAGTACTGGCGTGTGTG

ATAGCATTAGTTATCAGTATTGGAGCTGCATGGCTTTTATTATTAAAATGTAGGAATCGT

TCTAGTCAACCGCCACCAACACCAACACAACCACCCTCAGGGACTGGACCATCAAATCTG

GGAAGCGGGCCTAGTTCAACATCAGCTTCTCTCACCTCTAGCATTGCTGCTGGATCGGCA

AAAACATTCAGTCTAGCTGAAATGGAAAGAGCTACAGATAGATTCCACGAATCAAAAGTT

ATAGGGGAAGGTGGTTTCGGGCGTGTGTACCAGGGTACTCTTGAAGATGGGACCATGGTA

GCGGTAAAGGTCCTCAAGAGACACGATCACCAGGGTGGACGGGAATTCTTGGCCGAGATA

GAGATGCTTAGCCGCTTGCATCACAGGAATTTGGTCAAGTTGATAGGAATATGCACAGAG

GAGAATGCTCGCTGCCTTGTTTATGAGCTAATTTCAAATGGCAGTGTGGAATCTCATCTC

CATGGACTGGACAAGGAAACTTCTCCCCTTGATTGGAATGCACGCATGAAGATTGCGCTT

GGTGCAGCTCGAGGTCTGGCCTATCTTCATGAAGACTCGAGCCCTCGTGTCATACATCGA

GATTTCAAGTCCAGCAACATCTTGCTAGAGCATGATTTTACACCGAAAGTCTCTGATTTT

GGGCTGGCCAGAACAGCTCTGGATGAGGGAAATGAACATATCTCTACGCGTGTTATGGGA

ACTTTTGGTTATGTGGCCCCTGAATACGCAATGACGGGTCATCTTCTCGTCAAGAGCGAT

GTTTACAGCTATGGGGTTGTCCTTCTCGAGCTTCTCACAGGGAGGAAGCCGGTGGACATG

TCACAACCACCCGGACAAGAAAACTTAGTCAGCTGGGCCAGTCCTCTCCTCACAAGTATG

GATGGACTAGAGAAAATGATAGATCCAGCTCTCGGTGCAAGCATTTCATTAGACAGCGTG

GCGAAGGTGGCGGCAATCGCGTCAATGTGTGTTCAACCTGAAGTCTCTCATCGCCCATTT

ATGGGCGAGGTAGTACAGGCTCTGAAACTAGTGTGCAACGAGAGCGAAGAGCATAGAAGA

TCGTGCAGCCAAGGAGAGTTGTCAGCTAAAGACTCGGACATCAGAGTAAGTATGGGTCTA

GAAGACGAGACAGTGCTATCGGAATCCAACATATTTTGTACATCGGAAACGTTTGCCAGA

GATGATGAATCCAGCTCTTTCCGAAGATATTCTAACTCCGGTCCAGTGACAATGGACAGA

AGCCAGAAGTTTTGGGAGAAGGTGAGAGGGTTCTCATCAGGGTTTGCTGGCAAGCCAAGG

TCAGGTGTGGATGGTGGAGAACATTGGCCTTAG

>TRINITY_DN1009_c0_g1|m.141 TRINITY_DN1009_c0_g1|g.141 ORF TRINITY_DN1009_c0_g1|g.141 TRINITY_DN1009_c0_g1|m.141 type:complete len:167 (+) TRINITY_DN1009_c0_g1:67-567(+)

ATGGCTTCTCTCCTCCGCATCGCCCCTCTAGTACTCCTCCTACTAATAATCCCTTCTTCA

CCGACCCATGCTCTGATCTCGCCTCAAAAAGAGCTTCTAAAATCAGCTCAAAACCCCGAG

TTCTTCGACTGGTTGAAGACGATAAGAAGAAAAATCCACCAGAATCCCGAGCTCTCCTTC

GAAGAATTCGATACCAGCGAGCTCATCCGATCCGAGCTCGATGCGCTCGGCGTTAACTAC

ACCTGGCCGGTCGCCAAAACTGGGGTCGTCGCCCAAATTGGGTCCGGAGATGGACCCACG

TTCGCCCTCAGGGCGGATATGGATGCCCTCCCTCTTCAGGTAATTTCCTCGAACACGTCT

CTGCTGTTGTTTCAACTCTTTCCGATTTCAGCCATGTTGATCGATGATCACAACGTGCTT

CTCAAAATGTTCATCAGTAACCAATGCGTTATAACTAAGGCTCTAAAGAGCTTGTATATG

TTAGCATTCCGTTATGAATGA

>TRINITY_DN1009_c0_g1|m.142 TRINITY_DN1009_c0_g1|g.142 ORF TRINITY_DN1009_c0_g1|g.142 TRINITY_DN1009_c0_g1|m.142 type:complete len:136 (+) TRINITY_DN1009_c0_g1:2224-2631(+)

ATGCATGCTTGTGGTCATGATGCTCATGTGACAATGCTACTTGGTGCAGCCAAATTGCTT

CAAGATCGAATGGATAAGTTGAAGGGAACTGTCAAGCTTGTTTTCCAACCAGCAGAGGAG

GGTTATGCTGGTGCTTATCATGTGCTACGAGAAGGTGTTTTGGATGATGTCGAGGGCATC

TTTGGAATGCATGTTAGCCCTTCGCTTCCAACTGGCACCATTGGATCTAGATCTGGTCCA

TTTTTCGCAGCATCTAGTCGATTTTCAGCGACAATTATAGGAAGGGGTGGGCATGCAGCG

TACCCGCACAATACTAATGATCCAATTATTGCTGCATCTTTTGCTATTTTAGCCCTTCAG

CAGCTTGTATCTCGGGAATCAGACCCTTTGCAAAGCAGAGTGAGTTAG

>TRINITY_DN1009_c0_g1|m.144 TRINITY_DN1009_c0_g1|g.144 ORF TRINITY_DN1009_c0_g1|g.144 TRINITY_DN1009_c0_g1|m.144 type:complete len:113 (+) TRINITY_DN1009_c0_g1:4017-4355(+)

ATGGGGAAGGACCGCATTGCTTATCCTGCAACCATCAATGACAAAGGGATGTACAACCAT

GCAAAAACAGTCGGGCAGGGCCTCCTCGGGGAAGCCAATGTCCTGGAGACCCCGATGCTG

ATGGCAGCAGAGGACTTCAGTTTCTACGGAGAGACGATGCCAAGTGCTCTCTTCTTCATT

GGCATCAACAGTGATTCTCAAAAGAATGCCTACCCTTTGCATTCCCCGAACTTCTTTCTG

GACGAGGAGGCACTCCCCATAGGAGCCGCCTTCCATGCTGCGGTTGCAATGGCTTATTTA

AACAGTCCTCTCCCGGCAGGTTTAACTGTTGAGAATTAA

>TRINITY_DN100_c0_g1|m.146 TRINITY_DN100_c0_g1|g.146 ORF TRINITY_DN100_c0_g1|g.146 TRINITY_DN100_c0_g1|m.146 type:3prime_partial len:370 (-) TRINITY_DN100_c0_g1:1-1107(-)

ATGGAGTTAGTTTCACAAGTATCCCTACTTGTGACAATGCTAGCGTTCATCTTGTCTTCT

TCGGCTGCATCTGCTCCTAATTCTATAGGCTTCCGAGTGGATCTCACCCACGTCGATGCC

GCAGGAAGCTACACCCAGTCCGAGCTTCTTAAACGAGCGGTGGAACGTAGCAAGAGTCGT

GCTGCAATGTTAGCAAGCGGGTTCGGCCCAGTCTCGGTTTATCCTAGCCAAGGTGAATAC

ATATTGGACCTGGCCATTGGAACCCCGCCTCTCACCTTCACGGGCATCCTCGACACTGGA

AGCGACCTCATCTGGACCCAATGCAAGACCTGCATGAAATGCATACCGCAGCCATCACCA

CTTTTCGACCCATTCAGCTCCCCCTCCTTCTCCACCCTTCCTTGCACTCACAAGCTCTGC

CTCGCCCTGGCGCTCGATGAACGCTCTGCATGCGACCCCAACTGCTTGTATCATTATAAT

TACGGTGATAACACGGATACAATAGGATTTATGGGAACGGAAGCTTTTACATTCGGCACC

ACCAAAAAAGTCCGCATTCCGAACATCGTTTTCGGTTGTGGCCTGTACAACTCGGGCGAT

ATCCAGAACTCATCCGGGATCATCGGATTCGGGCGAGGGCCGATTTCCCTAATCTCTCAG

CTGGGAATAGCGACCTTCTCCTACTGCTTCACTGACAACTACAGAGATGACACAACCAAA

AGCCCTCTCTTCTTCGGCTCGTTAGCAAGCTTAACGGGTCCGAATGTCCGAACCATGCCC

CTAGTCCAAGTTAACCCGGCACTCTCAACGTATTACTATCTCTCATTAAAAGGGATAACA

GTTGGGAACACAATGTTACCGATCCCACAGGAAGCGTTTCAGGTGAAGAAGGACGGGAGC

GGGGGGATAATAATCGACTCAGGCACATCCCACCTCATGCTGGATACGCCGGCATACAAG

CCGTTTGTGGATGCTTTTAGGTCTCAGATCAAGCTGCATCCAGCGAATGTTACTGGTCTC

ATGCCATTTGATCTCTGCTACAAAGTAAGGAGACCTCCGGCAAGGGGGATTCCAAAGCTA

GTATTCCACTTTGAAAGTGCCGATTGG

>TRINITY_DN10101_c0_g2|m.147 TRINITY_DN10101_c0_g2|g.147 ORF TRINITY_DN10101_c0_g2|g.147 TRINITY_DN10101_c0_g2|m.147 type:complete len:198 (+) TRINITY_DN10101_c0_g2:270-863(+)

ATGAGTTGCAGCGGCTGCAGAGTTCTCAGAAAGGGTTGCAGCGACGTATGCGTCCTGCGG

CCGTCGATCTGCTGGATCGACGGCTCCGAAGCTCAAGGCCATGCCACCGTCTTCGTGGCC

AAGTTCTTCGGCCGAGCCGGCCTCCTTTCCTCCCTCTCCTCCGTTCCCGAATCCCATCGT

CCCGCTTTGTTTCGATCTTTGCTTTACGAAGCCTGCGGCCGCACGGTCAGTCCGATCACC

GGAGCAGCGGGGCTGCTGTGGAGCGGCAACTGGGACGCGTGCGAGGCAGCAGTGGAGACT

GTTTTGCGCGGCAAAATCCCTCGGCCGGTGGTGGCGCAGCTCGATTGCGAATCAGATGTG

CTGTACAAGCTGGAGACGACGCATCCCACAATAAAGAGGAGGAAGACATCGTGTGACGAT

CTTGATCTATGCTTGAGATCGTCGGCCAGACCTGATGGCAGGGCGGTGCCGATGAGACAA

CGGGCGGCGACACCGTCCGACGAGTCCGGGATGACGAGTGCAGAGAGTGGCCGTGAACAT

CATCATCAGTTTGCTAATAGGGAACAGCCTCAGCTCTTAGATTTATTTGTTTGA

>TRINITY_DN101029_c0_g1|m.149 TRINITY_DN101029_c0_g1|g.149 ORF TRINITY_DN101029_c0_g1|g.149 TRINITY_DN101029_c0_g1|m.149 type:complete len:171 (+) TRINITY_DN101029_c0_g1:112-624(+)

ATGACGGTGGATTTGGAGATAAACCCTAGAATTTTGAGGGGGGTTATAGTCTTGATGGGG

ATCGGCATTGGCGCGTACATTTTGGGGCCGCCTCTCTGTTGGCACATAGCTGAAGCCCTA

GCTCGCAATGCGGCGGATTGCTCTCCTTGCTCTTGCGATTGCTCGTCTCAGCTTCTTCTA

TCGATCAGGGAAGATTGTGGAAAACGTGATCCTGATATCACCGAGGATGTGGAGAAGAAC

TTCACTGACCTTCTCTCTGAGGAAGTGAATCTCAGAGAAGCAGAGGCGATAGATGCTCAG

AGACGAGCAGATATAATTCTACTTGACGCCAAAAAGTTAGCGTCACAGTACCAAAAGGAG

GCTGACAAGTGCAGCTCGGGGATGGACACCTGTGAGGAAGCGAGGGAAAGAGCCGAAGCT

GCAGTTACACAACAAAGGAGACAAACTTCAGCGTGGGAGATGAGGGCAAGACAGCATGGA

TGGAAGCCAAAGAGCAGTAAATCTCAATCTTGA

>TRINITY_DN10103_c0_g1|m.152 TRINITY_DN10103_c0_g1|g.152 ORF TRINITY_DN10103_c0_g1|g.152 TRINITY_DN10103_c0_g1|m.152 type:3prime_partial len:204 (-) TRINITY_DN10103_c0_g1:1-609(-)

ATGCGGGCGTCGGCGCGGGCGACACGCAGGGTGGCGTACACGCTGTTGGCCTTCATCTCC

TCCGTGATGACCGCCGTGGTCGACTTGACCGTCGTGCGGGCGATCGGGAAGAGCGCCCCC

GTGTGCTGGACGGCGTTCTTGGTCTCCTCCGGAGCCGAGTCGCCGTTCTTGGGCTTGTTG

ACGCTCTTGCGCGGGAAGACGACGAGCTTGGCCTTGTACGCCTTGAGGCGGTGCACGTTG

GCCTGGAGCGACTCGACCGACTTGTTGGTGCGACGGAAGTCGACGGCGATGCCGATCGAC

AGCGCCTGCTTGCGGCTGATGCCGGCCTCCTTCAGCTCCTCGAGCGTGAAGCCCTTGCCC

GCGCGCACCTTCGAGGCGTACTTGACGGTGGGGCAGTGGACGGCAGGACGCAGCAGACCA

GCGGCCGGGCGCGGGGCGATGGCAGCAGCCTTAGCCTTGCGCCGCAGACGACGGGTCTTC

TTCTTGGCAGCCTGGTCGAACCAGGTCTTGATGCGGCGCTGCCAGTCCTTGTGGAAGTGG

CCGTTGGGGATGACGTTGTTGTGCTTGACCATTTTCGCTCACTTCGGGGGAAGTTACGAC

GAAACCTGC

>TRINITY_DN10103_c0_g1|m.151 TRINITY_DN10103_c0_g1|g.151 ORF TRINITY_DN10103_c0_g1|g.151 TRINITY_DN10103_c0_g1|m.151 type:complete len:207 (+) TRINITY_DN10103_c0_g1:38-658(+)

ATGGTCAAGCACAACAACGTCATCCCCAACGGCCACTTCCACAAGGACTGGCAGCGCCGC

ATCAAGACCTGGTTCGACCAGGCTGCCAAGAAGAAGACCCGTCGTCTGCGGCGCAAGGCT

AAGGCTGCTGCCATCGCCCCGCGCCCGGCCGCTGGTCTGCTGCGTCCTGCCGTCCACTGC

CCCACCGTCAAGTACGCCTCGAAGGTGCGCGCGGGCAAGGGCTTCACGCTCGAGGAGCTG

AAGGAGGCCGGCATCAGCCGCAAGCAGGCGCTGTCGATCGGCATCGCCGTCGACTTCCGT

CGCACCAACAAGTCGGTCGAGTCGCTCCAGGCCAACGTGCACCGCCTCAAGGCGTACAAG

GCCAAGCTCGTCGTCTTCCCGCGCAAGAGCGTCAACAAGCCCAAGAACGGCGACTCGGCT

CCGGAGGAGACCAAGAACGCCGTCCAGCACACGGGGGCGCTCTTCCCGATCGCCCGCACG

ACGGTCAAGTCGACCACGGCGGTCATCACGGAGGAGATGAAGGCCAACAGCGTGTACGCC

ACCCTGCGTGTCGCCCGCGCCGACGCCCGCATCGCCGGCAAGCAGAAGAAGCGCCTTGCC

GACAAGGCTGCCGAGAAGTAA

>TRINITY_DN10108_c0_g1|m.153 TRINITY_DN10108_c0_g1|g.153 ORF TRINITY_DN10108_c0_g1|g.153 TRINITY_DN10108_c0_g1|m.153 type:internal len:126 (+) TRINITY_DN10108_c0_g1:3-377(+)

AATAGGGAGACCCTGTGGCTTTCAGTTTTTCTCCTTGTTATGTGTGCTGCTGTGGCAACT

GGTATGGGACTATGGCTGCATCATCATGCACACGAGCTTGACACTCTACCTTACTACAGG

AGGAGATACTTCACAAAGGGTCGTGATAATGGAATGCAGTACAAGTATTATGGTATTTGG

TTGGAGATACTGTTTTCATTTTTGAGCTCCGTGATAATTTTTCAAATAATGATTCCTATA

TCACTTTACATTACAATGGAGCTAGTCCGGTTGGGCCAATCTTACTTCATGATTGGTGAT

TCGGAGATGTATGATAGTAGTTCAGACTCAAGGTTCCAGTGCAGATCCTTGAATATCAAT

GAAGATTTGGGTCAA

>TRINITY_DN1010_c0_g1|m.154 TRINITY_DN1010_c0_g1|g.154 ORF TRINITY_DN1010_c0_g1|g.154 TRINITY_DN1010_c0_g1|m.154 type:complete len:610 (+) TRINITY_DN1010_c0_g1:250-2079(+)

ATGGCGAGGCCTCCACAGGACGCGATCGACACTTTCGTCAGCATCACCGGAGCCTCTGAG

GCCGTCGCGGTCCAGAAGCTCGAGGAACATGGAGGGGACCTTAATGCAGCTGTTAATGCA

CACTTCACCGAGGACAGGACCAATGTGCACACCGGCTCCCTTCCTGGTCCTGAAAATGAT

TTTATGGATACAGACGATCTGATAGACGTGGATGCTCCTGTTCCTGCGCACTCTTTACTG

TCAGCTGCCCAAAATTTGAACCCATTTTCTCTCATCAACTCAAATTTTGGTCCAAGATTT

TTTGATGGAGGTACTATTGAGTTTCCCAGCCGCACACCACATGTTTCACATCCGAGGGAG

GTAAGGGAGATTCCAATTGGATTCAAAGATGGGAATAGTCAACCTGGTTCTTCTATTCAT

GGACCTACCGGTGAGGAAGTTACTGGAAGCATACCAACACATGATCTAGATGTTCGTGGA

AATGTGATTATTGATGTTGACGATGATGACAATATTTTTCCAAGTGCTCCTCATCCTGAG

ACCAGCGAGTTTGGTGAAATTTCCTTGGGAAGACATCCTGGACCAAATTTTATCCCATCC

TCTAACATGTCTGACTATGGAAATGATATTGAAGAGGAAATGATCAAAGCTGCAATTGAG

GCTTCCAAGCGAGAGGCTGAGGCATCTGCAAGCCAGCAGTTTGATGTTTCTAATGGTTCA

GCTGGTCCCAGACGTGACGAGGCATCACCTGCTTTGGAGGAAACGGATCTTGTACATGCT

GTTTCCTTGTCCCTAAAGACTGCAGAGGAAGAAAAAATACTGCGAGAACTGGGGTTGCAG

GTTGGGGAGCACTCTTCTGACCCATTGGGTGTAGAAATGGAGGATGTGGGAAGAGTGCCT

ACTGCAAATGGAAGGCAAGTATTTGTTCCATCTCAGGCCGGAACTTCAGGTAAATTGGTT

CTAGAAGAAGGCAACACATCTATTCAAGAGGAAGCTAAAGATGTAGATGAACAACCATTG

GTGAGGAACCGTTCCAGGCACTTTGCTTCTGATAACGTAGAGTCGGCAAATGTTATTCAA

AGTCCTCCATTAAGTCCTCATGGGAATGATCGTGGTAGTCGTCAACCTAATGGAGATGCC

TTTCATGAGTGGGGTGGCATATCTTCTGAAGAGCATGACGAGGCTGTAATGCTTGAAGCT

GCCATGTTCGGTGGGATTCCTGAAGAAGGTGCATATAATTTCACATACCCACATCCTCGT

GTCCTACATCCTGATAACAGCTCAAATTTCTATCCTCGGGTACCTCGACCTCCATCACCC

TCCCTAGCTGCACAACGTTTGCTGAGGGAGCAACAGGATGATGAGTATCTTGCGGCATTG

CAAGCTGATAGGGAAAAGGAGTTGAAGGCTGTAGAGGAGGCTGAATGTCGTCGCATAGAA

GAAGCTGCTGCCAGACAAGCTGCTCTCGAGGAAGAAAAACATAGAGAGGAAGAAACTCGC

AGAAAATTGACTGAGCAAGAGGAGTTCGTGAGAATACTTGCTGCAAAACAAGCATCCCTT

CCGCATGAACCTTCTTCGGATGATGATAGTGCTGTGACCCTCCTTTTCCGCATGCCTGAT

GGAAGCCGGTTTGAACGCCGTTTTTTGAAGTCTGACAAGCTTGAGTCCCTGTTCAATTAC

ATAGATGTTGGAAGGGCTGTTATGCCTGGAACCTACAGATTGGTGAGGACATACCCTCGA

CGAGTATTTACTGATGGGGAAAGCACATTGTCTTTGAGTGAATTGGGTTTGAATAACAAG

CAAGAGGCATTGTTCTTAGAGTTGATATAG

>TRINITY_DN101104_c0_g1|m.158 TRINITY_DN101104_c0_g1|g.158 ORF TRINITY_DN101104_c0_g1|g.158 TRINITY_DN101104_c0_g1|m.158 type:internal len:279 (+) TRINITY_DN101104_c0_g1:2-835(+)

GAATTCATCGATGACATGACCGCCAACGCCGACGCGGTACAAGAGAAGGTTCTGGCTGAG

ATCCTTAGCAGGAACGCCGATACTGAGTACCTGAAAAGATACAAGCTCGACGGGGCGACC

GACCGAGAGACCTTCAAGGCTAGAATTCCTCTGAGCACTTATGAGACTCTTCAGCCTGAG

ATTCAGAGGATTGCCAACGGCGACCGCTCTGCAATTCTCTCCGCTCATCCCATCTCTGAG

TTCTTGACCAGTTCAGGGACGTCAGCCGGAGAAAGAAAGCTGATGCCGACAATCCAAGAA

GAGCTGGGTCGAAGACAGCTCCTGTATAGTCTTCTTATGCCAATTATGAGCCTTTATGTC

CCTGGACTGGACAAAGGCAAGGCCCTCAACTTCCTGTTTGTGAAATCAGAATCAAAAACT

CCCGGCGGCCTCCCGGCACGCCCAGTTCTGACCAGCTACTACAAGTCCGACCACTTCCGA

AGCCGCCCTTACGATCCCTACAACGTCCAGACCAGCCCCAATGCCGCCGTCCTTTGCGCC

GACTCCTTCCAGTCCATGTACATCCAGATGCTCTGCGGCCTCTACGACCGCCTCCAAGTC

CTTCACGTCGGCGCCGTGTTTGCATCCGGCCTCCTTCGCGCCATCCGCTTCCTCCAGCTC

CACTGGCAGAAGCTCGCCAACGACATCGCCACCGGTACACTCACAGCAGACGTCACCGAC

CCATCGGTCCGCGGCTCCGTCACTGACATCCTCACCCCCAATCCCGAGCTCGCACAGTTC

ATCACCGCCGAATGCTCCAAGAACGAATGGGCCGGAATCATCACCAGAATCTGG

>TRINITY_DN10111_c0_g1|m.161 TRINITY_DN10111_c0_g1|g.161 ORF TRINITY_DN10111_c0_g1|g.161 TRINITY_DN10111_c0_g1|m.161 type:complete len:592 (-) TRINITY_DN10111_c0_g1:442-2217(-)

ATGTTGGCAAAGTTCTCAACTTTTCGAAGCCAGAGAACCCACAAGTCTCAGAAGGAAAAG

CAACAGAACTATGAGAGATTTAACTCACACCAGCTTCTTACTACTACAGACACAGACATT

CCAAAGCCGGATGTATCTTACTTATCAAAATCAGTGCTAGCTGATAAAACAGCTTACGAG

GACTCTTATGCTGAATCACCAAGTAGAAATTCTGATGCCTCATTTCCAACAACACCCGAG

TCAAATGAAAATTCAAGTTCAGGTTCATTTAAAACATTTTCTGTTTATGACGGTAAAGGA

AGCACAACGCCTTCTTCAACTATATATGAGGAAATTGCTATTGGTCCATCTGTATTGCTG

CCATACGACGATGCTGAAGAACAGTTCTTAGAAGATTATGAGAGCTACTTTTTACACCTT

TCAGTTGATAATGTGCGCAAGGATCAATACCCTGATCTTGAGCTTCAAAGAATCGTTTAC

CTAGACTATGCAACCTGCCCACTTTACTCCAGATTCCAGGTAAAGCAACATATGAAGTTC

CTGCTTGAAGAGAGTGACTCAGAGTTCGGGTCTGATTTTCCTATCGACAAACAGTCTGCA

GTACATAACCCCTATATTGACAGTACCTACCAACACATATTGAATCTTCTCAATACAACA

CGGGATGATTATTCCATAATTTTTGCTCCTGGACTATCGTCATGTTATCGCCTATTTGGC

GAGATGTATGACTTGCAAAAGGGAAGTCTTCTTCTTGCAAGCAATGACCACCATAAGTCT

GTTCAGCATTTAGTAGATGTGGCAACACGATCCAATGTGAAAGTAGGAAGCATACCGCTG

AAGAGCAAAGATTGGCGTATCCATGGGGACGATATGTACAGATTACTAAGAAAACAAGGA

AGGAGTGGCAATGGGTGTGGACTGTTGGTCTACCCTGCACAGTCCTTCTTAACTGGGATA

TGTCATTCGCTGAATTGGATTCCGACTGCACAGCAAAATGGATGGAAAGTCCTTTTAGAT

GTTTCCAGTTGCCTCCCTATGGTTAACGTTGACTTGTCTCTATATCAACCTGAGTTTGTT

GTTGGTTCCTTGCATCACATGATTGGATATCCTTCAGATGTTGGTTTTTTACTAGTCAGA

AGTAGTTCACATTCAATTTGTGCTCAGAAAACATTGAATGAATTAAAATTAACTGATCGC

CCTGGCAATGGAAATACAGTTCATGTCATAACAGATGGCAATAGGGTGAATATCCACACA

TTTGCAGCTCTAAGATTTGGCCTTGATCATTTAGAGGACATCGGCATCATGGCAATTCAG

AGGCGGGTGCAGAGCCTGACCGCGTGGCTGGTCAAGACTCTGAATTCGTTGAAGCACAAA

TTGGATAGTAAGCCTTTGTTGCAGTTGTATGGAACTCTAGACCCGAAGCAAAGGGGCTCT

ATATTGGCATTCAATGTCGTAGATTCCACAGGAAATATATTTGCTCCTAGGCTTGTTAGG

CAACTGGCAGAGAGAAGCAACATTTTCTTAGATACTGGAAGTTTATGTAACATCAACATG

CTTCATCTCGTGCAATCAAAATCACATAAACAGGTGGATAGTTCAAGTGCTTCTTCATCA

CAACGTGATATTCAAGTCCTTCGGCTATCACTAGGCCCTGTTACGACCTTTGATGATGCC

TACAGGTTAGTGCAGTTCTTAGCCCGCTTTAGGGATGAAGACTACATGTCTTCTGAAGCT

GCTGGATATGTCCAAGAGCTAGAAAATGTTGTTTGA

>TRINITY_DN10112_c0_g1|m.162 TRINITY_DN10112_c0_g1|g.162 ORF TRINITY_DN10112_c0_g1|g.162 TRINITY_DN10112_c0_g1|m.162 type:complete len:448 (+) TRINITY_DN10112_c0_g1:156-1499(+)

ATGCAGAAGTGGAGCAGGAAGAAGTCCCAGCTCTCCTTCTTCCTCTCTCTCTTCACCCTC

TTCATGCTCTCATTCCATCTCTCCACCGACACCAACACCACAACAGCAACAATCCAAGTG

GGCCACGATGGAGTGTCGAGGTCTCAGCCTTCGTTCCCTACTTACGTCACCGTCAACGTC

TCCTCTCGTCACCCCATCAGCAACCGGGTCTTGAATGCTTCAGATTGGTCTAAGGCGTGT

ACTTCAGCTGAGAGCTACAATGGCAGGTCGAGTTCGGTGTTGGAGCACAGAATGGAGAGT

GCAAAGAGGAATCAACAATGTGATATGTTCTCAGGGAGATGGGTGTATGATAATATATCG

TATCCGCTGTACGATGAGGCAAGCTGCCCTTATATGTCAGATCAGCTAGCTTGCACGAAG

CACGGCCGGCCAGATACGGATTATCAGAAATTGCGGTGGCAACCTCACGGTTGTGACTTG

AAGAGGTGGAATGGAACAGAAATGTGGGAAAAGCTAAGAGGTAAACGGCTAATGTTTGTT

GGTGATTCTCTAAACAGAGGGCAATGGATATCCATGGTGTGCCTCTTACAATCACTAATT

CCTGCTGGAAAGAGGTCCATGTCACCCAATGCCCCTTTGACCATTTTCAAGGCAGAGGAA

TATAATGCCTCAGTTGAGTTTTACTGGGCACCCCTCATTGTTGAATCAAATTCTGATGAT

CCAGTGAACCACAGATTAGACCATAGGATGATCCGTCCAAACTCACTTCTCAAGCATGCA

TCTCAATGGGAGAAAGCAGACATACTGGTTTTTAATTCTTACTTATGGTGGAGAAGCGGG

CCAAAAATAAAACTATTATGGGATGTTGACGATGCAACGCATTGTGAAGATGCAGACGGT

TTTGATGCCATGAAGTTAGCTTTAGAAACATGGGCCGATTGGGTTTCTTCCAGGGTGAAT

CCTTTGACGCAGCGAGTCTTCTTTGTCACCATGTCACCTACCCATCTCTGGAGCCGAGAG

TGGAATCCAGGAAGCGAGGGGAACTGCTTCCAGGAAAAGGCTCCAATAATCGACAATGAA

GGTTACTGGGGGACTGGATCAGACCTCGACACGATGAGGATGGTGGAGAACATATTGGGT

GGTTTGGGATCAAAGGTTTCAGTTATTAGCATCACTCAGTTGTCAGAGTATCGAAAAGAT

GGCCATCCTTCAATATATCGAAAATTCTGGGAAACATTCACCCAAGAGCAACTTTCAAAT

CCCGCAAGCTATTCTGATTGTATACACTGGTGCTTGCCTGGAGTCCCTGATGTCTGGAAT

GAGTTGCTCTTCAATTTTTTGTGA

>TRINITY_DN10113_c0_g1|m.163 TRINITY_DN10113_c0_g1|g.163 ORF TRINITY_DN10113_c0_g1|g.163 TRINITY_DN10113_c0_g1|m.163 type:complete len:308 (-) TRINITY_DN10113_c0_g1:248-1171(-)

ATGGCCAGCAAGCTCCACACCCTCATCGGCGCCTTAAAAGACAAGGCCACCCTCTCCACC

GCCACGGCCTCCACCGACATCGCCGTCCTCCGCGCCACCCCCCACCATCCCTCCTCCTCC

CCTCCCTCCCCCCGCCACCTCGCCGCCCTCCTCGACTTCGGCCGCTCCTCCCGCCTCTCG

TCCTCCGCCCTCCTCCACTCCCTCTCCGCCCGCCTCGCCTCCGCCCGCGACCCCTCCGTC

GCCCTAAAGTCCCTCCTCTGCCTCCACCACCTCCTCACCCGAGGCTCCTTCATCCTCGTC

GGCACCCTCGCCTCCTCCCTCCGCCCCTCCTCCGGCCGCCGCAGCCCCCTCAACCTCTCC

TCCTTCCGCGACCCTTCGTCCCTCTCCCTCTCCTCCTGGGTGCGGTGGTACGCCGCCCTC

ATCGAGCTTCTAGTATCAACGACACCGTTCAGGAGCGGAAGCGACGACGAGCTTGCCGAC

CCCGACAGGCTGACGTCGCTCCTGAACGCGGACCTGATCGCGGAGATCGATTGCATGGTC

AGGGTGGCCGAGGAGATGGGCAGGCGGCCGGACACGGCGGCGGTCGAGGAGAACAAGCTT

GTGATGGAGGTGGTGAGGGTGGTGGCGGAGGAGAGGGCGGGGATAGAGCGGGGGATATTG

GCTAGGGTTCAAGAAGTGAAGGAGAGGGTTGATGAGCTAGGGTTTGCTGATGTTGTTGAG

TTGGCCTGCTTGTTCAAGAGGTTGGAGGAGTGCAGGCTGAAGTTGAGTGGCGAGGTGCGA

GAAGTTAGAGAGAGAGTGGAGAGGAAAGAGGAGAGGGAGAGAGCGAGGGTGAGGAGAGGG

AAAGTTAGCGAGTCGGCTCGGCTTGTGGACCGAGTTGGGGCTGGGCCAGTGATCTTTGGC

TCTAGTAGGTGGCTAAATGGTTGA

>TRINITY_DN10115_c0_g1|m.166 TRINITY_DN10115_c0_g1|g.166 ORF TRINITY_DN10115_c0_g1|g.166 TRINITY_DN10115_c0_g1|m.166 type:internal len:106 (-) TRINITY_DN10115_c0_g1:1-315(-)

CGCCCCAATCCAATCACTCTGGAAAAAATGAAGAGACAAGAATGGAGCCTCGACGACTTC

GAGATCGGAAAGTTGATCGGCGAGGGAAAATTCGGAAAAGTCTATCTCGCCAGAGAAAAG

CAGAGCGGATACGTAGTGGCACTGAAGGTAATTTTCAAGGAGAAGATCGAGAAGTACCGA

TTCCACGCTCATCTCCGCCGCGAGATTGAGATCCAGCACAGCCTCAACCACCCCAATGTC

CTTCGCCTTTTCGCTTGGTTTCACGACGAATCGAGAATCTTCTTGGTTCTTGAGTACGCT

GCGCGTGGAGAGCTG

>TRINITY_DN10115_c0_g1|m.165 TRINITY_DN10115_c0_g1|g.165 ORF TRINITY_DN10115_c0_g1|g.165 TRINITY_DN10115_c0_g1|m.165 type:internal len:106 (+) TRINITY_DN10115_c0_g1:1-315(+)

CAGCTCTCCACGCGCAGCGTACTCAAGAACCAAGAAGATTCTCGATTCGTCGTGAAACCA

AGCGAAAAGGCGAAGGACATTGGGGTGGTTGAGGCTGTGCTGGATCTCAATCTCGCGGCG

GAGATGAGCGTGGAATCGGTACTTCTCGATCTTCTCCTTGAAAATTACCTTCAGTGCCAC

TACGTATCCGCTCTGCTTTTCTCTGGCGAGATAGACTTTTCCGAATTTTCCCTCGCCGAT

CAACTTTCCGATCTCGAAGTCGTCGAGGCTCCATTCTTGTCTCTTCATTTTTTCCAGAGT

GATTGGATTGGGGCG

>TRINITY_DN10116_c0_g1|m.167 TRINITY_DN10116_c0_g1|g.167 ORF TRINITY_DN10116_c0_g1|g.167 TRINITY_DN10116_c0_g1|m.167 type:complete len:149 (-) TRINITY_DN10116_c0_g1:181-627(-)

ATGGGAGGAGGTAACGAGCGCTACTCCGGCCCATCGCAACCTCCATGGTCAAGACCTCCT

CCAAGGCAAGGACCATCATATCAAGCTTCATTCTTTGGAGGACATCCTCCTCCAGGTCAA

CACCCTGGAGGAAATAGTGAGCATAATCCATGGTGGGGCTATACAGGGGGAGGATACTAC

AATCCTTATTTCAGCGGCATGCCTCAAGATTTCCAACAAGGTGGACGGCAATGTTCTGGT

AACACCTCCAGCTACGTGCCTCGACCACAGAGGGTTCCATACCAGGGAACCAATGGTTAT

CAAGGTGGTAATGGCCCTGATCCTGGGATCCAAGTTTACGATAACATAGTTACCGGTGAC

CCAAGGACGGTGATGATTGGTGAAGGCAATCGTGCAGGGGGACAAGTAAGGGGGCGGATT

AGTGGAAACCGCATCTTCCGTTCTTGA

>TRINITY_DN10117_c0_g1|m.168 TRINITY_DN10117_c0_g1|g.168 ORF TRINITY_DN10117_c0_g1|g.168 TRINITY_DN10117_c0_g1|m.168 type:internal len:116 (+) TRINITY_DN10117_c0_g1:2-346(+)

TTCGATGCAAAGCGCTTGATCGGTAGGCGTTTCAGTGACCCATCAGTGCAGAGTGACATG

AAGCTATGGCCCTTCAAGGTGATCCCAGGCCCGGGCGACAAGCCCATGATTGTCGTCCAG

TACAAGGGCGAGGAGAAGCAGTTCTCAGCAGAGGAGATCTCCTCAATGGTCCTTATCAAG

ATGAGGGAGATTGCCGAGGCCTACCTCGGGTCGACCATCAAGAATGCAGTGGTCACTGTT

CCAGCATACTTCAATGACTCCCAGCGTCAGGCCACCAAGGATGCCGGGGTCATTGCCGGG

CTGAATGTGATGAGGATTATCAACGAGCCCACTGCTGCTGCTATT

>TRINITY_DN10120_c0_g2|m.170 TRINITY_DN10120_c0_g2|g.170 ORF TRINITY_DN10120_c0_g2|g.170 TRINITY_DN10120_c0_g2|m.170 type:5prime_partial len:291 (+) TRINITY_DN10120_c0_g2:1-873(+)

CGAGCCCGCCTCAACCTCACAGCCGCCACCATGAAACCGGCACAGGGAGTCAGCCTAGGT

CAATTGCCTAGCTATGTGCGGATATGCGTGCGACAGGCACACTCTGGCCGGTCTCGGCGC

CCCACGACGGGCTTTGTAGGTCCTGAGGGCCATGGGGAGCACATCTGGGTCTTTGGACAC

CGGAGGACAGACCAGATCATCTACAGCTTTGACGAGAAGCTCGATGGCTTTCACGACCTC

AAGCAACTCCCCTTCAACGGCAAAAAGACAAAACCCGCCAAGCTACGCAAGGATTACTGG

TCGCCCTTCGCCCGCATCTCCTTCCCCGCCGGCCAGGGCCCCGCCGGCCGCTCAGTCTTC

CAGAAGCTGCGTGAGCTTAAGCACCTGCACGAGGTGGCGTGGGACAACGAGTTCCGATAC

AAGCGCCCCGAGGAGTTCACGGCCGCCGACCGCAAGCGGATCGCCGAGGAGGAGAAGAAG

GGCAACCCGGGATACCGCCCCATTCGCAGCAAGCAGGAGCGCGGTATTGCCCTGAACGCT

CAGAAGCCGAACTCTATCGCCGACATGGCTGCCGTCCTGGGCGGTCTCGGACGGGGCAAC

AAGATTGTGACCAACGAGGCCGCCGAGGGCGAGGAGCAGAAGCTCTTGGATGTGACGGTG

AGCTGGGCCAACGACCAGGATAGAGAGTACGCCGAGAAGTGGTCTGGCAATGTTACGCAC

GAACTGTTCGAGAACCCCACATACTTTACGGAAAAGACGGTGGAAGCGGCAGCGGAAGCA

GAGGAGCCTCCCAAGGAAGAAAAGAAAGAGGCAACACCGGCTGGGGATGCTCCGGTTCAA

GATGCGGATGTGGTGGCAACCAAGGCACAATAG

>TRINITY_DN10120_c0_g2|m.175 TRINITY_DN10120_c0_g2|g.175 ORF TRINITY_DN10120_c0_g2|g.175 TRINITY_DN10120_c0_g2|m.175 type:3prime_partial len:105 (+) TRINITY_DN10120_c0_g2:2001-2312(+)

ATGCACGTGGGTGCGTCGCGGCGAAGATGGGGCGGGGCGCCATTTTGGATGGAAGAACCA

GGAAGAAAAGGGAGAAATAGCAGACAAGTGTTGTCGTCTCAACATCAAGCTGGAGGCTGC

AGCAAGAGGGGTGAGAAGAAGGGGAAAGCTGCCAAAGCTCTGAAGCGGCAGCTCAAGCAA

TATATGGCAGATGGACTCAACCAGGAGATGCTACAGAGTTTGCTCCTCCAGCTTCCGGAC

CATCGCCCGATATTTAACGTTCGAGCGATGGTCCGTAGCCCGCTCCACCAGGGGGCCCAG

ATCTTCCCACGC

>TRINITY_DN10120_c0_g2|m.169 TRINITY_DN10120_c0_g2|g.169 ORF TRINITY_DN10120_c0_g2|g.169 TRINITY_DN10120_c0_g2|m.169 type:complete len:303 (-) TRINITY_DN10120_c0_g2:1135-2043(-)

ATGGCGCCCCGCCCCATCTTCGCCGCGACGCACCCACGTGCATGCTCTACAGCCTTCGAA

CGGGTCTTCATGACTCGGCGCGACATCATGGAGAGTGTTCACGAACCATTTGGGGATGCC

TTCTACTACGGCCCCGAGTTTCTGAGCGATCGCTTCAGAGACGACGCCGACCAGCGCGAG

CGCAGTGGCTTCGCCGACAAGACGTACAAGGACATCTTTGACCAAGTCATGGATGCCGGA

AAAGAGGGAAAGAGAATCTTCATCAAGGATATGGCCTACTACCTCATGACCCCGGATGGC

AAGCCTACCAAGGTCGCGCCGTCCCTGGGCGAGGAGGAGCCCGGCAACCCAACCGTGGTG

CCGCTAGAGATCCTGAAGCAATTCCAGTTCACATTCCTCATCCGCCACCCCCGCAGGTCC

ATCCCCTCGTACTATCGCTGCACTGTGCCGCCTCTGGACGAGGTCACTGGCTTCTACAAC

TTCATGCCAAATGAGGCTGGCTACGAGGAGCTGGTGCGTCTCTTTGACTATCTGCTCAAG

GAGGGCGTCGTCGACAAGGACAACCTTGTCGTCGTCGACGCCGATGACCTGCTGGACAAT

CCTGAGAAGACGATCCGTCTCTACTGCGAGAAGACGGGCATTGACTTCAAGCCCGAGATG

CTCGAGTGGAACGACCAGGACAGCAAGTTTGCCAGCAACGCCTTTGAGAAGTGGAATGGC

TTCCACAACGATGCCATCAAGAGCTCGGCTCTCAGGCCACGCACTCATGCCCAGACATCC

ACCACCGAATCGGAAAATAAGGAGTGGACCGCCAAGTATGGAGCCGAGGCGCAAAAGGTG

ATCCGCAAGACGGTTGAAGACAACGTCGCGTACTACGAGTACCTGAAGCAGTATGCTCTG

CCTGTTTAA

>TRINITY_DN10121_c0_g1|m.176 TRINITY_DN10121_c0_g1|g.176 ORF TRINITY_DN10121_c0_g1|g.176 TRINITY_DN10121_c0_g1|m.176 type:5prime_partial len:386 (+) TRINITY_DN10121_c0_g1:3-1160(+)

GACAACACCGTACAACAAACTTCTCAATTAAAGGAAAACAGAGAGAGTAGAGAGATAGTC

ACAGTGAGAGAGAGGTGGAGAGCGGCGGCAGCAGCAATGGTGTCGATCTCGCTCTACAGA

GGCAACCTCCACCGCGTCTCCGACGCCCCTCGGCAATATCCGATGCCCCCGCGTGCCATA

ACCCTCGCCCAGTTTCGGATCCTCACCCGCAGGCGCGACGAAGCCCTCGCTCGCCTCGCC

GCCGCCAACAGAAACCCTAACCCTAGCTCTGGTAACAAGAAGGATGAGGAAGAGGAAGAG

GAAGCGAAGGTCAAAGAAGAGAAGAAAGATGAGGGAGTAGAAGAAGGAGACGTGAAGAAC

GCTGACGTTTCGGATCCGCAACCGAGCAAAGCTGGAGAAGAGGCGAAAGCAGCTGAGGAT

TCTAAACCGATCGATGCCGATGCGAAGGGGAAGGGGAAGAGTTCGGATGCTGATGCGGTT

AAGCGATCGGAGATCGGGACGACTGAGGCGAAGCCGGAGGTAAGCAATAGCTTGGACACT

GAGAAAGAGAAACGGAGAATAGAAGTTGAGAAGAAGTTGGAAGTTCTGAAGCAGAAAAAG

CACCATCTTGTGCAAGTGCTGAAACAGATTTTAAATGTTGAGGAGGAAATTAAAAGACGA

AATGCCCAATCTGCTGCCTTGCGCTCTTCAATTCCTCTGCAAGCTGAAACTGCAGTTGAC

ATGGGCTCTGCTACTAGGCATGTTCCAAAACTTAGTGTGGAGGTAAACTTCGGTGGGGAT

TTAGGTGCAGAATCAGATGCTACTGCTAACCACAGTGCTCATAGACATCAAATGCATCAT

GTGCATGGTACATCCCCATCTGCTGCATCTCTCACAAGGCCTTCCCAGCAGAATACGTTC

CTTTCCACCCCAAGGTCAAGTTTGGTGACTTCTGGACATGGCCAAACATCATCTAATGTG

TTTGTTGGCAATACTGCAGCAGCAACATCTAATCCATCCCTCGTTGCTCCAACTGGACAC

CAGGGCCATCATCATTCTGCAAGCCTGCCTCCAGTGTCTGTTCCTGGTTCCCATTTCATG

GCATCCTCTTCGTCCCCAGCAGCATCCGGCAGTGCTTCTGCAGCCTTTAGGGACTCTCGT

CTGACTAGTTCTTCCTGA

>TRINITY_DN101225_c0_g1|m.180 TRINITY_DN101225_c0_g1|g.180 ORF TRINITY_DN101225_c0_g1|g.180 TRINITY_DN101225_c0_g1|m.180 type:complete len:549 (+) TRINITY_DN101225_c0_g1:165-1811(+)

ATGGCCCCCGCCAATACCCTCCCCGCCTGGTCGGACCTCCAGGCTCACCGCGATAGCGTT

GGCAAGTCGTTTGTCCTCAAGGAGGCCTTTGCCTCGGACCCCGATCGATTCGACCGCTTC

ACCCGAACCTTCAAGGCTGAGGGCAGTAACGATATCCTCTTCGACTTTAGCAAGAACTTC

CTTACCGATGAGACCCTCGACCTCCTCGTCAAGCTTGCCGAGCAGGCCGGCGTCGAGAAG

AAGCGCGATGCCATGTTTGCTGGCGAAAAGATCAACTTTACCGAGGACCGTGCTGTCTAC

CACACTGCTCTGCGAAACGTTGGCGGCTGGGAGATGAAGGTCGATGGCGTCGACGTTACT

GCTGGTGTCAACGAGGTTTTGGACCACATGAAGGAGTTCTCTGAGCAGGTCCGCAGCGGC

GAGTGGAAGGGATACACCGGAAAGAAGCTCACCACCATTATCAACGTCGGTATTGGTGGT

TCTGATCTCGGCCCCGTCATGGTCACCGAGGCTCTTAAGCACTACGGTGCTGATGACATG

ACCCTTCACTTTGTCTCCAACATTGATGGCACCCACATGGCTGAGGCTCTCAAGGCTTCT

GACCCCGAGACCACTCTCTTCCTCATTGCCTCCAAGACCTTTACCACTGCTGAGACCACC

ACCAATGCCAACACCGCCAAGACATGGTTCCTCGAGAAGACCAACGGCGAGGGCGAGATT

GCCAAGCACTTTGTTGCTCTGTCCACCAACGAGTCCGAAGTGACCAAGTTTGGCATTGAC

AGCAAGAACATGTTTGGCTTTGAGAGCTGGGTCGGTGGCCGATACTCTGTCTGGAGTGCC

ATTGGTCTTAGCGTTGCTCTGTACATTGGCTTTGACAACTTCCACAAGTTCCTCAGCGGT

GCTCATGCCATGGACAAGCACTTCCGCGAGACTCCTCTCAAGGAGAACATTCCCGTTCTC

GGTGGTCTTCTGAGTGTGTGGTACTCTGACTTTTTCCAGGCCCAGACTCACCTGGTTGCT

CCCTTCGATCAGTACCTTCACAGATTCCCTGCCTACCTCCAGCAGCTTTCCATGGAGTCT

AACGGCAAGACCATCACCTCTGATGGATCTTCCGCCAAGTACACCACTGGCCCCATCCTC

TTTGGCGAGCCTTGCACCAACGCTCAGCACTCCTTCTTCCAGCTTGTCCACCAGGGTACC

AAGCTGATCCCCACCGACTTCATTCTGGCTGCCAAGTCTCACAACCCCATCACCGGCAAC

CTCCACCAGAAGATGCTGGCCTCCAACTACTTTGCTCAGGCCGAGGCTCTCATGGTCGGC

AAGACTGACGAGCAGGTGCGAGCTGAGGGTGCTCCCGAGGCTCTTGTTCCTCACAAGCGA

TTCCTTGGTAACCGACCTACCACTTCGATCCTGGTTGGCGGCACCATCGGCCCTGCTGAG

CTGGGTGCTCTGATTGTCTACTACGAGCACCTGACCTTCACTGAGGGAGCGGTCTGGGAC

GTCAACAGCTTCGACCAGTGGGGTGTCGAGCTGGGCAAGGTGTTGGCCAAGAAGATTCTC

AAGGAGCTCGATGAGGAGGGCAACGGTGAGGGTCACGATGCTTCGACGGGCGGCCTCATT

GGTGCGTTCAAGAAGTATGCCAACTAA

>TRINITY_DN101225_c0_g1|m.179 TRINITY_DN101225_c0_g1|g.179 ORF TRINITY_DN101225_c0_g1|g.179 TRINITY_DN101225_c0_g1|m.179 type:complete len:598 (-) TRINITY_DN101225_c0_g1:147-1940(-)

ATGTTCCATGTCCTACGCCGCAATGCATCGAGACTCATCTTGCAACACGGCCGTATCGGT

TCATCATGCTATCTTTTCAATGTTTCATTATCCACAGTGTTCCATTTGTCCTCCCATTAC

CATTTCCTTTTAGTTGGCATACTTCTTGAACGCACCAATGAGGCCGCCCGTCGAAGCATC

GTGACCCTCACCGTTGCCCTCCTCATCGAGCTCCTTGAGAATCTTCTTGGCCAACACCTT

GCCCAGCTCGACACCCCACTGGTCGAAGCTGTTGACGTCCCAGACCGCTCCCTCAGTGAA

GGTCAGGTGCTCGTAGTAGACAATCAGAGCACCCAGCTCAGCAGGGCCGATGGTGCCGCC

AACCAGGATCGAAGTGGTAGGTCGGTTACCAAGGAATCGCTTGTGAGGAACAAGAGCCTC

GGGAGCACCCTCAGCTCGCACCTGCTCGTCAGTCTTGCCGACCATGAGAGCCTCGGCCTG

AGCAAAGTAGTTGGAGGCCAGCATCTTCTGGTGGAGGTTGCCGGTGATGGGGTTGTGAGA

CTTGGCAGCCAGAATGAAGTCGGTGGGGATCAGCTTGGTACCCTGGTGGACAAGCTGGAA

GAAGGAGTGCTGAGCGTTGGTGCAAGGCTCGCCAAAGAGGATGGGGCCAGTGGTGTACTT

GGCGGAAGATCCATCAGAGGTGATGGTCTTGCCGTTAGACTCCATGGAAAGCTGCTGGAG

GTAGGCAGGGAATCTGTGAAGGTACTGATCGAAGGGAGCAACCAGGTGAGTCTGGGCCTG

GAAAAAGTCAGAGTACCACACACTCAGAAGACCACCGAGAACGGGAATGTTCTCCTTGAG

AGGAGTCTCGCGGAAGTGCTTGTCCATGGCATGAGCACCGCTGAGGAACTTGTGGAAGTT

GTCAAAGCCAATGTACAGAGCAACGCTAAGACCAATGGCACTCCAGACAGAGTATCGGCC

ACCGACCCAGCTCTCAAAGCCAAACATGTTCTTGCTGTCAATGCCAAACTTGGTCACTTC

GGACTCGTTGGTGGACAGAGCAACAAAGTGCTTGGCAATCTCGCCCTCGCCGTTGGTCTT

CTCGAGGAACCATGTCTTGGCGGTGTTGGCATTGGTGGTGGTCTCAGCAGTGGTAAAGGT

CTTGGAGGCAATGAGGAAGAGAGTGGTCTCGGGGTCAGAAGCCTTGAGAGCCTCAGCCAT

GTGGGTGCCATCAATGTTGGAGACAAAGTGAAGGGTCATGTCATCAGCACCGTAGTGCTT

AAGAGCCTCGGTGACCATGACGGGGCCGAGATCAGAACCACCAATACCGACGTTGATAAT

GGTGGTGAGCTTCTTTCCGGTGTATCCCTTCCACTCGCCGCTGCGGACCTGCTCAGAGAA

CTCCTTCATGTGGTCCAAAACCTCGTTGACACCAGCAGTAACGTCGACGCCATCGACCTT

CATCTCCCAGCCGCCAACGTTTCGCAGAGCAGTGTGGTAGACAGCACGGTCCTCGGTAAA

GTTGATCTTTTCGCCAGCAAACATGGCATCGCGCTTCTTCTCGACGCCGGCCTGCTCGGC

AAGCTTGACGAGGAGGTCGAGGGTCTCATCGGTAAGGAAGTTCTTGCTAAAGTCGAAGAG

GATATCGTTACTGCCCTCAGCCTTGAAGGTTCGGGTGAAGCGGTCGAATCGATCGGGGTC

CGAGGCAAAGGCCTCCTTGAGGACAAACGACTTGCCAACGCTATCGCGGTGAGCCTGGAG

GTCCGACCAGGCGGGGAGGGTATTGGCGGGGGCCATTTTCGAGGTGGGGGGTGA

>TRINITY_DN10122_c0_g1|m.183 TRINITY_DN10122_c0_g1|g.183 ORF TRINITY_DN10122_c0_g1|g.183 TRINITY_DN10122_c0_g1|m.183 type:5prime_partial len:133 (-) TRINITY_DN10122_c0_g1:160-558(-)

TTCGGCATCTCCCGTTGCGCAACTTTCGCCAACAGGCTGTACTTCTTTAACGCAACGCAT

TCGCAGGATCCGTCGCTGGATCCATTGTACGCATCGTTCATGAAGATTAACTGCCCCAAC

AGTGCCGACGCAGGCGCTCGGACGATGAACATGGACATGTTCACACCGAACCGACTGGAC

AATGTGTACTACGCGAACTTGATGCATCACCGGGGGTTGTTTACCTCGGATCAGACGCTG

TACGAGAGTCCGTCGACACGGCGGATTGTGGAGGAGAATGCGAGGAGGGCGAGGAGTTGG

AAGAACAAGTTCGGCAGGGCGATGGTGAAGATGGGGGCGATTGAGGTGGTCACTGGGAGG

CAGGGGGAGATCAGGAGGCAATGCAATGTTGTCAACTGA

>TRINITY_DN10123_c0_g1|m.184 TRINITY_DN10123_c0_g1|g.184 ORF TRINITY_DN10123_c0_g1|g.184 TRINITY_DN10123_c0_g1|m.184 type:complete len:596 (-) TRINITY_DN10123_c0_g1:686-2473(-)

ATGTGGTTTCTTCACTCACTTTTTGTACTCATTCTTCTCTGGACATTGGTTTATGGTCAG

AACTTCACCGTTCCCAGTGCTTGGCGAAAACCAACATCCTCTATTGACCAAGATGCGAGA

TACAATATCCTAAGCGGCGCCATAGACTCTCTTATTCCTTCATTTAACGAGTCAAGTGGT

CAGATTGATGCGCTGGAGTACACTCAGAACGCGAATCTCTTATGTGCGATTGCGCAGAAG

GACTGGATCACGAGCAGTAATGAGCATCAGTCAGACGTTGTCCGTCATATTGACACTGTA

TTCAACTTGAATCCGAATATTTCCTCGACTTTGAACACAGACCCGATAAATTGGGGACTA

GCAGCGGCATATGGGTACCGGGCGTATCAGGACACAGGCCTGCTGAACCTGGCTGTGTCA

ATATGGGAAGAAATCAACAAGTACATGATTTATCCGGAAGACGCCGTGAGGGGGCGACAT

GCGACGAAGAATGTGACCATCCCAGAGACATGTAACGGAGCTAGCATTGCCGGGGGTGTC

TTCTATATCCGCCATATGACTGAAAATACCGATGTAAACGGCGCCACTGTCGGAGGCTTC

CTAGCGCTTTCTGCACACCTCTGGGAAGGAACGAATGAACCTCGATACGCCAACGCTGCC

TTGCTTTCCTCACAGTTCATACAGTCACAGCTCTATAATGGAACAATCATCCTCGACACC

ATCACGGTCGCCAACTGTTACAGAAACCCTACATTGTCGGTCACGTACAACTCCGGATTT

ACCATTGAAGGTCTGAGCATCTTTTCGAACCTAACAAGCAATGCAACGTGGACGGGATTC

TTGCATGATCTGGTTTCGACAACTGCGGTCTTCCCTCACTGGACTGAATCGGATGGAATC

ATCATTGAGGGTGCCTCCGCCGCTGAAGATGCATATACGAACGGTTTCGGCCAGGCCTTG

AAGGGAATTTATGTTAGGGGTCTTTATGAAGCCTGGTCTCGGGCTAAACCTAATGGTCCC

TTTGCAAACTATGTTCAAAGCTTCCTCACAGTACAGTTCAATGCTTTGCAGGATCTCGCA

AGGACCCCAAACACGAACATCTATTCCCCAAGATGGCAGGGCCCGAATGTGAATCAAACT

CTTCCATGGGGTCAACTCGCCGCACTGGACGTTTTAAACGCAGCTGCTGGCATGGCCACT

ACTCCCAAAAGTGCGGGGACCAGTACGACCCAGATCACACAACCCTCAATGACTGCCGTC

AGCGACTCGAAGCCCAAGAGTACCAATCATACCCCTGTGATCGCTGGTGTAACGGTGGGC

GTCGGAGGCTTTCTGTTACTCATATCCCTAGCGATCTTCTTCTTGTTTCGTCGACATCGC

AAGCAGAAAGAAGATCGCGAACGCGCTATGTTGGAGGTGCCTACTCCATTCGGCATGGAT

CAATACACCCAGTACCCACCATACGAGACATATCAACCTAACATGTCTCAGATCCCACTG

TCGGCATCAGTTGATACTCCTTCGAGCATTGGAAAAGGAGGTATGACTGTCCTCTCGTAC

GCACCTGAGGGGTCAACAGCTAGACCAAGCATCTCCAACTCTATTGCGAATGGTTCGGGT

ACTGCACGAGCAGGGCCAAGCACATCACCCTTAGTCCCAGGGGTGCAGACGGATGAGACG

CTGGATTTGGCTGCGGTACCAGTTCTTCTGGAACAACTAAACCAGATCATGGCTAAACTA

CCTCCAGGAGGTGTGGATGAAGAGCACCCTCCTCGTTACGAGGGCTGA

>TRINITY_DN10123_c0_g1|m.185 TRINITY_DN10123_c0_g1|g.185 ORF TRINITY_DN10123_c0_g1|g.185 TRINITY_DN10123_c0_g1|m.185 type:5prime_partial len:144 (+) TRINITY_DN10123_c0_g1:2-433(+)

CGCCGGGCCCATTCCTCGCTATCCTCAAGACACCCGACACTTTTCAAGATGACCAAGAAG

AGAAGAAATAATGGCCGCAACAAGAAGGGTCGTGGCCACGTTAACTTTGTCCGCTGCTCC

AACTGCTCACGCTGCGTGGGCAAGGACAAGGCCATTAAGCGCTTCACAGTGAGGAACATG

GTCGAGAGCGCGGCTGTCCGTGATATCAGCGAGGCTAGTGTCTACCCTGAATATGTCATC

CCCAAGCTCTACATTAAAATTGCGTATTGTGTGTCGTGCGCTATCCACTCGCACGTTGTC

CGTGTACGCTCGCGTGAGGGTCGCCGCAACCGCGCACCTCCCCCTCGCATCCGGTGGAAG

GATGGCAAGAAGGTCAACCCCGCCGTCGCAGCTGCTGAGGATGCCAAGGCGGCTGGTGGT

GCTAAGGCATAA

>TRINITY_DN10125_c0_g2|m.189 TRINITY_DN10125_c0_g2|g.189 ORF TRINITY_DN10125_c0_g2|g.189 TRINITY_DN10125_c0_g2|m.189 type:complete len:223 (-) TRINITY_DN10125_c0_g2:102-770(-)

ATGTCTGCAGAACTCGACAAGTCTCTCGACCAGATCATGGCGACGCGCCCCAAAAACAAC

CGCAGGGGATCTCGTCGTGCTAGCGCTCCTGCATCGACCACTGCTCGCCAGCGCTATGCC

ACTGATGTTCCCGCCTCGAAGCGCGGGGCGGCTCCGGCTTCCACTGCTGGTACTCCCGCC

CAGGCCAATGCGCAAAAGATTATGGTTTCTGGACTGCCGCCTGATGTAAACGAGGAGCAA

ATCAAGGAGCTCTTCACTTCGACTGTTGGCCCTCTCAGCTCTGTTCAACTCAACTTTGAC

GCCCAGGCCCGCTCCAAGGGCACTGCGACTGTTATCTTCCAGAAGCGGGGCGACGCCAAC

AAGGCCTTTGCGCAATACAACAACCGACTCATCGATGGGAAACGCCCGATGAAGATTGAA

ATCGTTATGGATCCCACCAAGGTGCCTCCCCCATCTCTCGTCTCGCGTGTCGCTCCCGCC

CCCGAGGCGAAGCAAACGCCCAAGGCTGCTGCTAGCGGCGCCGAGAAGGCAAAGTCTGGG

AAGCCTAACGCACGTGGTGGACGTGGCCGTCGCCGCAAGGGTGGAAACGATCGCCCGGCG

AAGAGTGCCGCTGATTTGGATGCCGAGATGGAGGTCTACAAGGCGGCCCCGGAAGAGGCG

GCTGCGTGA

>TRINITY_DN101266_c0_g1|m.190 TRINITY_DN101266_c0_g1|g.190 ORF TRINITY_DN101266_c0_g1|g.190 TRINITY_DN101266_c0_g1|m.190 type:5prime_partial len:134 (+) TRINITY_DN101266_c0_g1:1-402(+)

GTTCTGGAGGTTGTTAGTGGAAGACCTATTGCGGATACATCACTTGATGAAGAAAAGATT

TACCTGCTTGAATGGGCATGGCATCTACACGAGCATAGTCGTGACCTAGAGTTAGTAGAT

CAGAAACTGGTATCATTTGATGAGAAGGAGGCCGCTCGACTTATTGGTGTATCTCTTCTC

TGCACCCAGGTAACACCGATGCCACGACCTCACATGTCACGAGTGGTGGCTACGCTGACA

GGAGACGTCGAGGTGGCTGAAGTCACAACAAGGCCTGGTTACTTAATGGAATGGCAATTG

AAGGGTCTGAGCAGCAATTTCATAAGTACCGACTTATCGAAAGCCTCAACCTCAGGCTCA

ACATATACCCAAGATCCTGGGACGTCAGAGCAAGTACGGTAG

>TRINITY_DN10126_c0_g1|m.191 TRINITY_DN10126_c0_g1|g.191 ORF TRINITY_DN10126_c0_g1|g.191 TRINITY_DN10126_c0_g1|m.191 type:complete len:282 (+) TRINITY_DN10126_c0_g1:89-934(+)

ATGCCCCTCGCCTCCGCCTCCCTCTCCTTCTTCTCCTCTTCCCTATTTTCTCCCCAGCAT

TCCCCAAAATCCCCTCTGTGTCCCCGACCAATCCGCTCCTCCTCAAAACCCCAATCGATC

CGCTCCGAGCTCACCGCCACCCTCCCTGTCCTCTCCTTCACCGGCAACCAGACCTCCACC

GCCGTCCTCACCCTCAAGTCTGCCCCTCCCTCCACCTCCCGCGCCGTCGTCCACCGCGGC

CTCATCGCCGACCAGCAGAACGCCCGCCGCGGCACCGCCTCCACCCTCACCCGAGCCGAG

GTCCGGGGCGGCGGCAAGAAACCTTACCAACAAAAGAAAACCGGCCGCGCCCGCCGCGGG

TCCCAGCGGACCCCGCTCCGCCCCGGTGGCGGGGTCATCTTCGGCCCCAAACCCAGAGAC

TGGTCCATCAAGATCAACCGCAAGGAGAAGCGCCTCTCCATCTCCACCGCCATCGCCAGC

GCCGCCAGGTCCGCCGACGCGTTCGTGGTTGAGGAGTTCGAGGAGGAGTTCGAGTCCGGG

CCCAAGACCAAGGAGTTCATCGCAGCGCTGAAGAGGTGGGGGCTGGATCCCAAGGACAAG

GCGACTTTTCTCGTGACGGAGCTCTCGGATAACGTGGTACTCAGCTGCAGGAACATTGGG

ACCATTAAGATGCTCACCCCGAGGACGCTGAATTTGTTTGATATTCTTGATTCGGAGAAG

CTCGTGCTGACCACGTCGGCCGTGGATTATTTGAATGCCATGTACGGGGAGGGTGGCGAT

GGCGATGGCGATGACGAGGAGGGGATTGAAGGGGATGAAGAAGCCGAGGGTGATGAAAGC

TCTTAG

>TRINITY_DN10127_c0_g1|m.195 TRINITY_DN10127_c0_g1|g.195 ORF TRINITY_DN10127_c0_g1|g.195 TRINITY_DN10127_c0_g1|m.195 type:complete len:509 (-) TRINITY_DN10127_c0_g1:540-2066(-)

ATGTTGTCCTCAAATTCTGTCACATCTCCACCCCTCTCTCTCTTCTCTCCATCCCAACCT

CAAAATCTCAACTCCAAACCATCTCCTCTCTCTAACTCCGCATCATCAACTCCTCGCTAC

AATTCCCTCATCAGAACCCACGTCAACTCCCACCAGCCGACCCAAGCCCTCTCCCTCTTC

CGCCGCCTACTCCTCTCCGACCCCCCTCTCCTCCCCGACAATTTCACCTTCCCTCTCGTC

CTCAAGGCCTGCGCGCACCTCTCCGCCCCCAGACAAACCCAGCAGATCCACTCCCTCATC

ATCAAATCCCCCTCCATCCCCAACCACGACGTCTTCATCCTAACCTCTGTCGTCAACGCG

TACGCCGAATGCGGCTGCATCGGCGATGCCCGTCTCGTGTTCGACGGAATGCCCCAACGA

AATATAGTTACGTGGAACTCTATGATGGCCGGGCTTTTAAAATGCGGGGACGTCAGCTCT

GCTGTCGAGTGTTTCGACCAAATGCCAGAGAGGAATATCGTCTCGTGGAATTCGCTGATT

GGGGGATATGTGAAAGCGAACATGCCGCATGAGGCTGTTGTGCTGTTCCTTGAGCTGCGA

GTTTCGGGTCTGAGACCGGACGAGTCGACTATGGTGAGCTTGGTATCGGCGATTTCTGAT

CTGGGTCTGCTTGATTTGGGACGAAGAGCGCACGGGTACATAGCACGGAATGAGTTCTCG

CTTGACGGTGCTCTTGGTGTCGTGCTGATCACTATGTATACTAGATGCGGGAGTATTGAC

GCCGCTTATCAAGTGTTTCTAACCATTCCGGTGAAGAATGTTGGGCATTGGAGTTCTATG

ATTGCAGGGTTCGCTAACCATGGCCTCACCGAGGATTCTCTTCGAGTTTTCTCCGAGATG

CTACGGTCAGGCATCAGGCCGAACCACATTACATTTATCAGCGTTCTGAATGCTTGCAGG

CACGCAGGCTTGGCAGAAGAAGGCCTGAAGTATTTCAATCTCATGAAATGCTTTGACATG

GAACCTCGGATCGAGCATTACGGGTGTTTGATCGACTTACTCGGGCGTTATGGGCTTGTG

GAAGAAGCAATGCAGATAGCTTGCAACTTGCCGATGGATCCGGGGTTCGTGGTATGGGGC

ACGCTGCTAGCTGCTTGTACAAATCACAGGAATGTTGAGATCGCAGAGATTGTAGCAAAG

AAGTTGATCGAACTCAGACCTGAACACGGGAGTGCTTATGTGCTGCTCTCTAATCTTTAT

GCGAAGGTCGGGAGATGTGAGGATTTCAAAAGGATGAGGAAGATGATGGAGGAGGGAAGG

GTGACAAAAGTTGCAGGATTCAGTTGGATTGAAGTGGATGGCAGAGTTCATGAATTTGTC

GCCGGAGATCAGTTTCATGCGAAGGCTGAAGAGATTTACGGCGTTTTGGAAGAGATGAAG

TGGAATTTGAGATGCGAAGGGCATGAATCAGTTGCATATGGAGTTTCTAGTAGTAGTGTA

GAAAATATTGAGGAAGAAGAGACTTGA

>TRINITY_DN10128_c1_g1|m.198 TRINITY_DN10128_c1_g1|g.198 ORF TRINITY_DN10128_c1_g1|g.198 TRINITY_DN10128_c1_g1|m.198 type:complete len:259 (-) TRINITY_DN10128_c1_g1:299-1075(-)

ATGGATGGAAATTTTGGCCCGACTGAGCAAATACTCTGGCCGGTTTCGGTCTTTTCCGGC

ATCGTCATGTGCAAGATTGTATATGAAATCACTGGCCGGATCAGCTTCATGTACTTTAAG

GGATACAACAAGCTAAGCAAATCACAGAAAATCGAGTGGAATAACCGGGGGTTTTCTACC

TTCCATGCAATTGTCGCTGCTGCAATATCTTTATACTTGCTCGTTCTATCAGATACTTTC

AAAGAGGGCAATTCTGATGATTTATTAGTAAATAGAAAATCAGTACTATCTGATTCCATG

TTTGGGATTTCGCTGGGTTATTTTCTCGCAGACTTGGGGATGATCCTATGGCATTTTCCA

GCATTAGGGGGCAAGGAGTATATCTTTCATCATGCAGTCTCTATGTACTCGTTTTTGTTG

GCTCTGATAAGTGGCAAAGGACATTTCTATATACTCATGGTTTTGTTTACTGAATTTACA

ACCCCCTTTGTAAACTTAAGATGGTATCTGGATATTTCTGGTCAAAAGAGTTCTATGCTT

TATATTTACAACGGTGTGGCTCTGTTTCTTGGTTGGCTGGTTGCAAGAATACTTTTCTTC

ATCTACTTCTTCATCCACATGTATCTTCACTTTGATCAGGTGAAAACAATATTTCCATTG

GGTTTCTACAGTATGCTGTTGGTTCCTTCCCTGCTCTCAGTAATGAATGTGTTGTGGTTC

CGGAAAATCTTGAGGGGGATGATCAAAACTCTTTCTAGAAAGAGACATGAGCAATGA

>TRINITY_DN10128_c1_g1|m.199 TRINITY_DN10128_c1_g1|g.199 ORF TRINITY_DN10128_c1_g1|g.199 TRINITY_DN10128_c1_g1|m.199 type:3prime_partial len:140 (+) TRINITY_DN10128_c1_g1:777-1193(+)

ATGGAATCAGATAGTACTGATTTTCTATTTACTAATAAATCATCAGAATTGCCCTCTTTG

AAAGTATCTGATAGAACGAGCAAGTATAAAGATATTGCAGCAGCGACAATTGCATGGAAG

GTAGAAAACCCCCGGTTATTCCACTCGATTTTCTGTGATTTGCTTAGCTTGTTGTATCCC

TTAAAGTACATGAAGCTGATCCGGCCAGTGATTTCATATACAATCTTGCACATGACGATG

CCGGAAAAGACCGAAACCGGCCAGAGTATTTGCTCAGTCGGGCCAAAATTTCCATCCATC

ACTTCTTCCTTCCAAATTGCTATAGACCAACTAATTAAACTTGCAAGAGAAGAAAAAGAC

CCGAAAACGATGCGCCTTCAGTTGTCCAAATTTAACAGGGATTTTAGAAGAGAGAGA

>TRINITY_DN10129_c0_g1|m.200 TRINITY_DN10129_c0_g1|g.200 ORF TRINITY_DN10129_c0_g1|g.200 TRINITY_DN10129_c0_g1|m.200 type:5prime_partial len:433 (+) TRINITY_DN10129_c0_g1:2-1300(+)

TTCCGGCCGACAGCCCTCGCAATCCAGCGTGCTGGAGAGGATCCAAATCCCTCTTCCGCT

ATTTTGCTCTTTCCTATTTTTTTTGCCCCCTTGGTCGGTGGAATGCTGAAGGGGATGAAG

AGATTGCGATTCGAAGCCTCGCTTCCCGCCCTAATTTCAGGCGGAACGAAGAAGATCTCG

GGATTCCCGCCCACTGCCCCCTTCGCCAGTGGCGGACAGCGGCTCAACACGAGGAACAGT

TATATCATCTCATCTTTTCAGAGAATATCTACTTCAGTACAATGGATGAATCTGCATACT

GCTGTAGATTCACAGCAGTCATTGGAGTTTCCAAGATTTGCAAATATTGTCATCGAAGAC

TTGAAAACTTCGCTGCCAAGTACTACAGTAACTGATGATGACTTCGACGAACTGGGTCCT

TCGGTTTCAGGAGATAGTGATTCAGGCTCTGTTTTGACAAAGAAAGAGTCCAAGGCCCCC

AAAACAACTCAGGAGCAAAGTTCCCCCATCAAACTGCATGGTGCCAAGTTCCCTACTAAG

CAGGACAAGTGTCATCTAGGTGGGCACTTCAATTCGGAAGGCCAAAGAGGCCCATTAAAA

AGCAAGACCAAGGCTGGGAAACAGAAATCTTTGTCTAACAATAATAAAAAACAAACCTCA

TCCCTGTTCATTTCAATTGATAACATACCCCCAAATATCAGTCCCCATGAACTGAGGGAA

GCAATTTCAGTTTATGGAGAGATTCTGAGTTCGTCTATGAGAGCAGGAGCAGATGGACTG

GCCAGCTGCCATGTCAAATTTATGAACTTGGAAGGAAAGACAAGGGCACTTGCAGCTAGA

TGGATTACCATTAAGAATTCCCAGCTTCCAATCTGTGGGCTTCTCGATAGACATGATTAC

ATCTCGATTAGGATAGCTAACATTAGCATGGATACTACTGAGGTGGCTATACACTCAATA

TGCAAATCATTTGGAGAAGTGTTTGGTCTTGCAAGGACTAAAGAGGGTGCAGCTGATGTG

ATGTTCAGAGTTGAGGATGTTGAAGTTTCCCAGAGCATAGTGAAGCATTTGAGTGATGTT

GTAATTGATGACCGCCAATGGTCAGCTAAGCTGCTTACGAAGCTCAACGACACCCCCACA

ACCAACGCTGCAGACTCTCAGCGGCAAGTTGGTTCGCAAATTACCAACCACTTGGAGAGA

TTTCGGAATCAACTCAATATGCAGAGGATTTATTTGGAGGATTTAGAGAACCTGCACCAT

GCAGTGTTACACCTCAGAAATAGCCCTCCTAATCTGTAG

>TRINITY_DN1012_c0_g1|m.202 TRINITY_DN1012_c0_g1|g.202 ORF TRINITY_DN1012_c0_g1|g.202 TRINITY_DN1012_c0_g1|m.202 type:complete len:958 (+) TRINITY_DN1012_c0_g1:296-3169(+)

ATGGGAAGAATGATTAATGTGTTTGAGTTGAGTGCAGGCATGGGTGGAACCAAGTTGCTC

ACTGATAAAGCTCAGAGAGATGGTTCTCCATGTCGTATAAATCGTAATGATGTTGCTAAG

AAACCTGCGGATTCTGGGGTAGCTCATGCAGAGGACAAACAAATGACCAGTGAGCTGAGG

AAGAGTTCTTCAGCCAAGAAATCGGGGGGAACACCAATGAAGATGCTCATAGCTCAGGAG

ATGTCTAAACAAGCCGAGTCTAAGAGGAAGCAAACCAATGTTGTGGCAAGGTTAATGGGT

CTTGATGAAGCACTGCCTGCTCGTCAGTCAGCTCCAAATGGTGAAGGACGCTCTGACAAG

AGTGGTCGTTCTTCACGTGTTACTTTAGCTGGAGCACGTCAGAACTCCCGGCGACTGGAA

GATGGCTATTTTGAGAGGCCATTGCCTCACGAAGTTCGCTCTTGTGCCCGTGGGAAGGAA

TATAGAGATATCTATGAGGTGTGGCAGCAACCACCAAGAACCGGTCATTTCAAGGATCAG

CCTCTTCAGAAGGGAAGGTATGTTGAGACTCCAAATGACAGAAGGATGGCTCTTGTTCGT

CAGAAATTCATGGAAGCAAAGAGGCTGGCCACAGATGAAAAGCTCCTACAGTCGAAAGAA

TTCCATGATGCTCTTGAAGTTTTAAGCTCAAATAGGGATTTGTTCCTGAAATTTCTGGAG

GAACCAAATTGCCTTTTCTCAAAGCATTTGAAAGAACTTCATTCCACTCCTGCGGCACCT

CAAACAAAGAGAATTACTTTACTGAAGCCGTCAAGAACAGTAGAGACGAAAGGTGGTACA

CAGGTGAAGAAGCAGCAATATCCAGTGACTACCGAAGGTGGATGGCACCTGGACAAACCG

CAACGAAGCTCTGGCACGACTCAACCAACCAGGATAGTGGTCTTAAAACCTAGTCCTGGA

AAGCGCCATGACACAAAACCTATGGCTATCCCAATTATATCTTCACCAGAACTAACAGAG

AAGGATGATTTTGGCCAAGTTCTGGAAACTACTGAATCCATAGGGTCTGGAGAAGTGGCC

AAGGATATCGCTCAGCAGATGAGGGAGAGTGTTAGTAGTAGTCACCGGAGAGATGAATCT

TTATTATCTTCAGTCCTATCGAATGGCTATGTTGGAGATGAGAGCTCATTTAATAGGTCA

GAAAATGATTACAGAGGAGAAGAGGATGGAGGTTTCAGTGATTCAGAGATTCTTTCTCCG

ACTTCACGATATTCTTGGGACTTTTCCAACAGATATGGCAGTCCATACTCATTCTCATCC

TTTAGTCGGGCATCCTGTTCTCCTGAGCCATCCGTGATCAGGGAAGCCAAGAAACGGCTT

TCAGAGAGGTGGTCCATGGTAGCTTCTAATGGAAACAGTCGAGAGCAAATGGAACTACGA

AGGAGCTCTAGCACCTTGGGTGAGATGCTTGCTCTTCCTGAGGTAAAGAAAGAAGAAGTG

AAGGTCAGGGGCCTCACTGTTTCGAGCAGTAGATCATATGGAGGTGAAGAAGACTTGAGG

GTGTCGAGTGCCAGCTTGTCTACTTCTCAAACAAAGGGTGGAGATGGTGTCGAGAGATCC

CCAGGGAATTTATCGAGGTCAAGGTCTCTTCCAGTATCTTCTGCTTTCGAGGACAATGTG

TCGAATTCAGAAGATTCCAACTCACAAGTCAAGAAGCCCATTGTGGCCAAGGACGCCGCA

AAGTCTAAGAGTGGGAGATCATCATTCAAGGGGAAAGTTTCAAGTTTGTTCTTCTCTAAG

AATAAGAAGGGTGGTAGAAATAAATCCACTTCTTCTCCCTTGGTGGGATCTGAAAATGTT

GGGAAGAGTCGCAGTGATGAGCCCGTTGAGAAGGAAAATGATGATAATAATAATCAGCCT

GAAAGTTGTCCGCCAATTAACTCCGGAGAAGAATCTATCAAGGAGTCTTCTCCTGCTACG

GTGAAGGGAGCATCAAAACAGGGAGCTCTCTCTTTCAGGGATGTTCTTTCTCTGGAGAAA

CCGAATGCATCTGAAAGTTTAAACGATAATCAGAACAACCCTTGTTCGTCTAATAAAATT

GGTGAGAACCAGAACCAACCCAGTCCCACCTCAGTTCTAGATGCACCATTTGAAAATGAT

ACAAGCAACAGCACACCTAGTTCAGCTGAAAGTTTCAATGCTGTACATTCACTGGCACCA

ATCGAGTCAGTTGCCTGGACTTTGTCGTGGGATGACACCAACCTGGAAATATCATCACCT

GACACGCCAAACCTTCCCGTAAATTTGCTCAAGGCCAATGCAGAGGATCAGCAACGGCAT

ATTTTTGTCCAGAGAATACTCTCAGCTGCTGGGTTAGACAATGGGAAGTTGAACGTGGCG

TTCAGATGGCATTCAGTTCATAGCCCGTTGGACCCGATTTTGCTTGACAAGTTTTTGAAC

CGGAAAGAGGAAGATGCTAAGAGCAGGGAAAGGAGATCAAACCAGAGGCTCATTTTTGAT

TGTGTCAATTATGTTCTCTTAGAAATCAGCAGGACTGCCTACTTGGGTGCATATCCATGG

GCCAGAGCATGTAGTGTGAGTCGAAAGAAAGCTCTTGCTGGTGCTTTTATAGAAGATGAA

GTGCAGGAGCTCGTAAGTGACTGGTTTACCGGTGAAGGGAAGTTGGAACCATGCATGATC

GACAATGGTGGTCTGGTGGTGGACAGACTATCGAGACGGGAGGTAGGAGGAAACGGATGG

GCTGAATCGATGCAATCGGAGATCGATGAAATCAGCAAAGAGGTTGGTGGCAAGGTATTG

GATGAGTTAGTTTGGGAGGCTCTGGCAGACCTAACTGTTGGGTGTTTGTCATGA

>TRINITY_DN10131_c0_g1|m.204 TRINITY_DN10131_c0_g1|g.204 ORF TRINITY_DN10131_c0_g1|g.204 TRINITY_DN10131_c0_g1|m.204 type:complete len:161 (+) TRINITY_DN10131_c0_g1:84-566(+)

ATGTCGCAAACCCAACAACAGTGCTCCATGGACATCGACGGCGTGCACGCAGACGCATCG

CCTGATCCGCCATCCTCGAAAGACCCGGCCGGCGACTACCAAAGGAGCACGCTCCTTGTG

GTCCTCACCCTGATCGCCACCCTATCATTCCAGCTCGGCGTGAACCCTCCGGGGGGTTTC

TGGCAGGACGATGCAAGCGGGCACGTCGCAGGGAACGCCATCATGAAGGACAAGAACCCG

AAGAGGTACCGGGCCTTCTCCATCTTCACCTGGCTCTGCTTCTGGGAGTCCATGGTGCTC

ATCTTGGCGTTGATGAATGGAGTGTCGCTAAAGTCCCGCTGGATCAGGGGGCTCTTCATA

TTCTGCTACTCCTGTCTGGCGTTCCTCTTTCTTGCCTCGCAAACAGATGTGCACCTGCCG

TTGGCCTTTGCTGTGTGGTTCAGTGTCATGTTGTGGGCCGCATGGGTCATGTGGAAAAAC

TAG

>TRINITY_DN10132_c0_g1|m.205 TRINITY_DN10132_c0_g1|g.205 ORF TRINITY_DN10132_c0_g1|g.205 TRINITY_DN10132_c0_g1|m.205 type:complete len:125 (+) TRINITY_DN10132_c0_g1:975-1349(+)

ATGGAGGTCTCATCTTCTTCTTGTTTCCAAGGAGAAGACGCTTCATGCATTATCGACTTC

GCTAAGCATAGCTACCCGCGGCCTCAACTCTTGTTTCCGAAGCAAGATTGCCGCGAGGAA

GTTGTTACCCCTGCCCTGACCCCGGGAAGCTCGTCGTCGGACTGTTTCGAGTTCCCGGCT

CTCGGGACGCTGGACGGATCAGATCAGGTGGTTGAAGAATTTACAGGAGCGGTATCGGAG

AATAGTGACATAACATCAGGATTTGAACAGTCTTTTTGTGAGAGTTTGGGGTGGGATTTC

ATGATGGAAGAGTCGTTTGATTTTCGTGAGATGCTTAATTTTGAACATGATGTGTTGTTT

CACAACAACATATAA

>TRINITY_DN10133_c0_g2|m.207 TRINITY_DN10133_c0_g2|g.207 ORF TRINITY_DN10133_c0_g2|g.207 TRINITY_DN10133_c0_g2|m.207 type:complete len:422 (+) TRINITY_DN10133_c0_g2:79-1344(+)

ATGGGGTTGGGGGAGCCGGAAGCTTCGATCGACAAGGGATCGGCGATCGCCTGCTCGATC

TGCCTCGAGATCGTGGCCGGCGGTGGGGATCGGTCCACGGCGAGGCTCCAATGCGGCCAC

GAGTTCCATCTCGATTGCATTGGATCAGCTTTTAACGTTAAAGGAGTGATGCAATGTCCA

AATTGTCGAAAAGTTGAGAAAGGCAGTTGGCTTTATGCCAATGGTTCCCGTTCACAGCCA

GAGCTTGGCTTTGATGATTGGACTCATGATGAAGATCTTTACGAGCTCAGTTACTCAGAA

ATGACTTTTGGAGTTCATTGGTGTCCATTCAGTAGATTAGCCCGGGCACCTTCATCATTT

GAGGAAGGGGAATCCTCTCCAGCATTTGCTTTTCATGATCTTATAGGGTATCATACCATG

TTCACTGGGAACACAGCTACTTCGTTGGCAGCTGATCCAGGGTCATACTTGGCATACCTC

CAACCACTCCAACCTTCAGCTTCCTCAAACCCTCATATTTCTTCTGAAAGTCCTATTGAT

CATTGGAGTCGTACCACTGGAACTCCAGAGGTTCAGACTCATGGCTGGGAACACCATCCT

GCACCATATCCCCTGCCTAGCAGTCGTTCTAGTGGTTCCTATCAAGAAGGGTTTTCTTCA

ACAACTGTTCCGGCATTGAGGGTTGATTCCGATGGTCTAGCCAGGGCTGGATCTTTGTTC

CATCCCCTTATTCTTGGCCATGGATCTGGTTCTAGACTTGCGGGTTCATTTGTGTCTTCA

CTGATTCCACCATATCTCACCAACTCTCGACCTCATGGCCATGTTCGTGATCACTACCAA

CATCGCAATCTCCCAGGACTACATGGTACAATCTTCCCAGGCACGCAGCAGCGATCAGTA

TCTACAAACCGGCTGCCTCCAGCTCCGGTAGGACCAACTCCTCCTTCGATACCAGATCAG

ACAACCTATTACCTGTTCCCACCCACAGCTTCATCCAGCCGAAGCCACCAAGATGCTGAA

AACCCAGGAGATCATTTCTACTCAACGTGGGAAAGAGAACGTCTCGTGCCCTTCCCGTTA

TTGCCTGTTCCAATCGATAGGGAGTCTGGTTGGTGGAGGCCTTTTCCGCATTCTAGTAGT

TCGTCAGATTCCAGCAGCCGGATGGGTTACTGGCACCGGAACGGCTCTGAGAGGCCTTTG

TCTCAAGGACGAACAGAGGCCTCTTATCGGCCGCCCCATGTAGGGAGAACGAGGCCTTAT

ATTTGA

>TRINITY_DN10136_c0_g2|m.208 TRINITY_DN10136_c0_g2|g.208 ORF TRINITY_DN10136_c0_g2|g.208 TRINITY_DN10136_c0_g2|m.208 type:internal len:168 (+) TRINITY_DN10136_c0_g2:3-503(+)

TTTTTTTCTAGGTTTTCTTCAATCTCCTACGCTCGATCCCAAATAAAAGGTAAAAATTCT

CTCTCTCAGTCCAAAATTAGGGCCCCGATCTCTAATTACCCTCATCAATTTCACCAAAAA

TCTCATCTTTTTACTCAGATCAAGATGTTGGAGCAGCTTCTGATCTTCACGAGAGGCGGT

TTGATCCTGTGGACCTGCAACGAGCTGAGCAACGCCCTTAAAGGATCTCCGATCGAAGCC

CTAATCAAATCTTGCCTCCTCGAGGAGCGATCCGCCGAGACCTCCTTCAACTACGACGCC

GGCAGCGGCGGCGGCGGTGCATACACCCTGAAGTGGACGTTCCACAACGATCTCGGTCTC

GTTTTCGTCGCGGTGTACCAGCGGATCTTGCATCTTTTGTATGTCGACGATCTTTTGACG

GCGGTGAAGCGGGAGTTTGAGCAGATCTACGATCCGAAGCGGACGGGGTACAATGAGTTT

GATCAGATCTTCAGGCAGCTG

>TRINITY_DN10137_c0_g1|m.209 TRINITY_DN10137_c0_g1|g.209 ORF TRINITY_DN10137_c0_g1|g.209 TRINITY_DN10137_c0_g1|m.209 type:5prime_partial len:366 (+) TRINITY_DN10137_c0_g1:1-1098(+)

CAACTTAAATCAGTTGCTGCCTTGTTCATCTCACTCTCCGTCACCAACATGAAGTTCTTG

ATCCCTTCTTGCTGCTACTGCGCTCGCACCGCCGACGATATTTGGCCCTCGGCCTCCAAG

GTCGCCGACATCTTCACCGAACAAGATCAAGATGAGACCAACAAGGATTCAATCCGGAGG

TTCAGCTCCTCAGAAATTCACTATACCATCACGAGGAGTTCCAACGTCATTGCCGAGGGA

GGTTTCAGCACAGTCTACCGTGCACGCCTCCCTGGTTCCTCCTCGCCTCTGGCGGCTGCC

GCCTTCAAAGTCCACGACCAGTCCAGCGAGCGCCTCTACCGTATGTTCTGCCGAGAACGC

GACGTCCTTCTCAAAGTCAGCCACCCCAACATTGTCCGCCTTCTCGGTTACAGCGATGAC

GGGGAGGCTGCAGGAGTCTTAGTGTTCGAACACGTTCCCAACGGAACAGTGCACGACGTG

ATTCATTCTAGCAGTAAATTCGTGCTGTCCTGGCCTCAGCGCATGTCCATTGCGTATCAG

CTCGCACTGGCCTTGGACTACCTGCACGAAGGCTGCGACATCCAAATCATACACGGCGAT

ATCAAGGCGTCGAATGTCCTTCTCGACAGCAACCTGAGCCCGAAACTATGCGATTTCGGG

TTCGCTCGGATGGGCTTCTCGGCCACGGTCACTCGCTCTGCTAGCCCGATGATGGGATCT

CCTGGCTACGTCGACCCTTGCTATCTGAGAACGGGTTTAGTTTCGAAGAAGAGCGACGTG

TACAGCTTCGGGGTACTGATTTTGGAACTCATCACCGGAACGGAGGCATTCTCTGAAGAG

AAATCGCGGATGCTGACGACGGTGATGCGGCCTCGACTGCAGGAGGCATCGAAAGCGAGA

GAACTGTTGGATCACAGGATCAGCGGAGAGTATGATGAAGCTGAGGTTGTAGCTATGGCC

AAGATCGCAGCACAATGCATCGGAAGTAACCCGAGCCTTCGGCCTTCGATGGGAGAAGTA

TTGAAGATCATGAGAGAGAGCGTCCCCTCGGCCGTCGCTGCCGCGGACGTGATATCCAGC

GGCAAGAAAAAGAATTAA

>TRINITY_DN10138_c0_g1|m.211 TRINITY_DN10138_c0_g1|g.211 ORF TRINITY_DN10138_c0_g1|g.211 TRINITY_DN10138_c0_g1|m.211 type:5prime_partial len:525 (-) TRINITY_DN10138_c0_g1:342-1916(-)

CTAATACCTTACCCTCTCAAATTCTCAACTCTTTCTTGTGGACAATGCGCAATGGCCCTC

TCTATACTCATCTCCCTCACTTTACTCTCCATCGCCATTTACTTGCTCTTCTTCATCTTC

AATAAAGGCAAGAAGCATAATCCCAATCCAAATGCCATCAAAATATATCCCATCGTCGGC

GTCCTCCCGCAGTTCCTCGCGAACCGCCACCGACTACACGACTGGTTGGCCGACGTCCTC

GCGGCCTCCCCGACCAACACAATCGTTTACCGCCGCCCTGGCAACATCCGCGGCATCACC

ACCGCCAACCCCGCCAACGTCGAACACATCCTCAAGTCCAACTCTGACAACTTCCCCAAA

GGCTCCTGGTTCCGCTCCGTGCTTCTAGATTTCCTCGGCAACGGCATCTTCAACTCCGAC

GGGCATCAATGGAAGGTCCAGCGAAAGACGGCCAGCTTCGAGTTCAACACCCGGTCCCTC

CGCAATTTCGTCGTTGGAAACGTTCAGCAGGAGACTTCTACTAGGCTTCTTCCCTTGCTA

AAGAGAGCGGCTGATCGTGAGCAGGTTATCGACATACAGGATGTGCTCGAGCGATATGCG

TTCGATAACGTTTGCAAGGTTGCGTTCAATGAAGACCCCGGCTGCCTAGCTGATGCCGAG

CTCCCCGGATTCGCTGACGCTTTCAGGGATGCTGCTGAGTTCAGCGCCGGAAGGTTCCGG

TACGCAATTCCAGGATTCTGGAAGATCAAGAAATTTTTTGGCATCGGTTCCGAAAGGAGG

CTGAAGGAAGCCATCGCCACTGTCCACGAATTTGCCACCCAGATCATCCGGTCACGGATG

AAGGAGATGCAGACCGCTTCCAAGCCTCAGGCCGACGATCTGCTGTCGCGGTTCATCGCG

CTCGATGAGTATTCCGAGGAGTTTCTGAGAGACATCGTGATCAGTTTCATACTGGCCGGC

CGGGAGACCACATCCTCGGCTCTCACCTGGTTCTTCTGGCTCTTGTCGTCCAGGCCTGTT

GTGCAACAGAAGATACTTGAAGAGATTGCGTCGACTCGGGCCCGAAGTGAGAGCTCCAAT

AACGAGACATTCGAATTCGACGAGCTGAGAGAGATGCAATACTTGCATGCCGCGATAACA

GAGTCAATGAGGCTGTACCCTCCGGTGCCGACAAACCTGCTCCAGTGCGAAGAAGACGAT

GTGCTGCCGGATGGGACCGAAATCAAGAAGGGTTGGTTCATCTCATACCAATCGTATGCA

ATGGGAAGAATGGAGGCAGTTTGGGGGGAAAATTGTAAGGAGTATAAGCCAGAGAGGTGG

CTGGATGATGCAGGGATGTTCCGGCCAGAGAGCCCGTTCAAGTTCTTGGCGTTTCACGCG

GGGCCAAGGATGTGTTTGGGGAAGGAGATGGCCTACATTCAGATGAAGTCGATTGCGGCG

TGCGTGCTCGAGAGATTCGTGGTGGATGTCGTGGAGAAGGACAAGTACCCGGAGCAGATG

CAATCGCTGACATTGAGGATGAGAGGAGGGCTCGCCGTGCGTGTCAGGAATCGGCATGCG

GCCAACACCGATTGA

>TRINITY_DN10139_c0_g1|m.215 TRINITY_DN10139_c0_g1|g.215 ORF TRINITY_DN10139_c0_g1|g.215 TRINITY_DN10139_c0_g1|m.215 type:complete len:364 (+) TRINITY_DN10139_c0_g1:204-1295(+)

ATGGGAGCCGCCGCCGATGAGGCCGAGAAGGACGTCCTCCAGTCTCTCGTGTTCAAGAAG

TCTGAGAGCATCGATTCCCTCGTGATCGGCGGCTACGACTTCAGCCGCGGCCTCGACCTT

GCCGGGCTGCTCGACTCCATGGCCTCCACCGGCTTCCAGGCCACAAACTTGGGAGATGCC

ATGGATGTCGTCAATCAAATGCTCGCTTGGAGGCTTTCGCACGAGGTTCCCACTGAGGAG

TGCAGCAAGGATGAAAGAGATCCTGCTTTCAGAGAGTCGGTAAAGACCAAGATCTTCCTT

GGCTTCACGTCAAACCTCATCTCCTCTGGAATCCGAGAGACCATCCGGTTTCTTGTCCAG

AACAAACTGGTAGATGTATTGGTGACAACAGCTGGAGGTATAGAAGAAGACTTGATCAAA

TGCCTTGCACCCACATTCAAAGGTGACTTTGCCTTGCAAGGGACTCACCTGCGCTCTAAA

GGATGGAACAGGATTGGGAACCTATTGGTTCCCAATGGCAACTATTGTAAATTTGGGGAC

TGGATCATGCCCATTCTGGATCAAATGCTAAAAGAACAAAACTCACAGAAGGTACTATGG

ACGCCATCTAAGGTGATCGAGCGATTTGGAAAGGAGATTAACGATGAAAGTTCGTATTTG

TACTGGGCGGCAAAGAATGGTATTCCGGTCTACTGTCCAGCGATAACTGACGGGTCGATA

GGAGACATGTTCTACCTCCATGCCTTGGAAAAACCCGGTCTAGTTGTCGATATTGTACAA

GATGTGAAGGCAATGGACGAAGAAGCAGTTCACGCAAGTCCGCGGAAGACGGGCATCATA

ATCCTCGGAGGAGGTGTTCCGAAACACCACATCTGCAACGCCAACATGCTGCGGGATGGG

GCAGATTACGCGGTGTTCATCAACACGGCGCAGGAGTTTGACGGGAGCGACTCAGGAGCT

CGCCCGGATGAGGCTGTGTCATGGGGCAAGATCAAGGGTTCAGCAAAAACTGCAAAGGTT

CATTGTGATGCAACCATAGCATTCCCATTGTTAGTAGCCGGGACATTTGCCAAAAAAAAT

GGAAAAAACTAG

>TRINITY_DN1013_c0_g1|m.217 TRINITY_DN1013_c0_g1|g.217 ORF TRINITY_DN1013_c0_g1|g.217 TRINITY_DN1013_c0_g1|m.217 type:complete len:129 (+) TRINITY_DN1013_c0_g1:1389-1775(+)

ATGATAGCTGCAACAATACGGCACCGATGGCAACCTACACGACCGGCAAAGACTCCGTTG

CAATCAAGTCCGCTGGTGACCACTTCTACCTCTGTGGATTCCCCGGCCACTGCCTCATCG

GACAGAAGCTCAATATCACGGCTCTCGCCAAGGGTTCCTCTGCTGCCCCTTCCACGGCCC

CTGCTGGTGCCCCGTCCACGTCCCCGGCGCCTGGCGGGAGCGGTGGAAGTACCGGCGGCG

GTGCCGCCACTGCTGGCCCTTCGGAGGCCCCAACTTCGGGCGGTTCTGCAAGTCCAGCGG

CTGCCCCGAATGCGAAGAATTCAGCTGTGGCTTACAAGGGGGTGGACATGGGACTGTCTG

CTCTTGCAGTTGCTGCAGGGTTTATGA

>TRINITY_DN1013_c0_g1|m.216 TRINITY_DN1013_c0_g1|g.216 ORF TRINITY_DN1013_c0_g1|g.216 TRINITY_DN1013_c0_g1|m.216 type:complete len:141 (+) TRINITY_DN1013_c0_g1:1367-1789(+)

ATGGAGGTGAATAAGGCTGATTATGATAGCTGCAACAATACGGCACCGATGGCAACCTAC

ACGACCGGCAAAGACTCCGTTGCAATCAAGTCCGCTGGTGACCACTTCTACCTCTGTGGA

TTCCCCGGCCACTGCCTCATCGGACAGAAGCTCAATATCACGGCTCTCGCCAAGGGTTCC

TCTGCTGCCCCTTCCACGGCCCCTGCTGGTGCCCCGTCCACGTCCCCGGCGCCTGGCGGG

AGCGGTGGAAGTACCGGCGGCGGTGCCGCCACTGCTGGCCCTTCGGAGGCCCCAACTTCG

GGCGGTTCTGCAAGTCCAGCGGCTGCCCCGAATGCGAAGAATTCAGCTGTGGCTTACAAG

GGGGTGGACATGGGACTGTCTGCTCTTGCAGTTGCTGCAGGGTTTATGATTATGGCTTAT

TAA

>TRINITY_DN1013_c1_g1|m.219 TRINITY_DN1013_c1_g1|g.219 ORF TRINITY_DN1013_c1_g1|g.219 TRINITY_DN1013_c1_g1|m.219 type:complete len:390 (-) TRINITY_DN1013_c1_g1:181-1350(-)

ATGGTTCATCAGCCGGTTGGCCCGCTCCTCTGTGCCCATGTCCACGCACAGCATGCCCCC

GAACCCGTACCCTGGGTTGGCCATCAACTTGAGCAGCTCATGGTTCGGATGGTCCTCCAG

GCCTGGATAGATCACCTTCAGGCCCATCTTCTTCATGCGGGTGGCGAATGTGAGCGCGCG

GTGGCAGTGCTCCTTCATGCGGAGGGAGAGGTGGGGGAGGCGCTCCGAGATCTCGAAGGC

CACCTTGGCGTTCATGGTCGGGCCAAGGAGCATCAAAGCGCCCTGCTGGAGGTCCATCAT

CGAGTTCACCAGGCTTGCCGGTCCGCAGATCGCACCTGCGATGACGTCAGCGCCACCGCT

GATGTACTTGGAAACACTATGCACCACCACATCCGCCCCCAGCCTCGCCGGCGACACCAC

CATGGGCGTAAACGTATTGTCCACCACCAGCATGACCCCCTTTTCTTTCGCTATCCTGCT

CAGCGCCGGGATATCCGCCACCGCCAGCGTCGGGTTCGACATGGTCTCGGCGTACAAAAC

CCTCGTCTCGCCCTCTTTAATCGCCCTCCTCACGGCGTCCAGATCCCCGGCGTCGACGAA

GCTCGTCGTGATCCCGCAGGCCCGCGGCAGGAATCGCGCCAGCAGTGCGTGGGTCCCGCC

GTAGAGGCACCGGGAGGCGACGACGTGGCCGCCGGCGGGGCAGAGCTGGAGGAGGACGGA

GGAGATGGCGGACATGCCGCTGGCGGTGCAGTAGGCGGCCTCGGTGCCCTCCATGGCGGC

CATCTGGCGGCCGAGGTTGAGGACCGTCGGGTTGAAGTGGCGGCTGTACACGAAGAAGTC

GCTCTCCGGGCCCAGCTCGCCGGAGAACATCCTCCTCATCGTGTCCGGCTCCATCACCGT

GAAGGTGGCGGAGGCCTCGATGGACATGTTCACTCCGCCGTGCTCGCCGAACTCGTGCCG

GGCGCTCGCCAGCGCCTGGGCCGGGTCGGCCACGGGCTTCTTCTTCGCCGCGGCAGCCGA

CGACATCCAGTGGTCGTCGTTGTTGTCTTCCATGTCTTTGGAGGAGCGTTTCTTGGGGTT

GGAGTTGGTGAAGGGGAGGGAGGGCATGGTTTCAGCCATGCTCATGGACGGATGATTTAT

TTTCCTATCTCTCTCTTTGTATTTGTTTAG

>TRINITY_DN1013_c1_g1|m.218 TRINITY_DN1013_c1_g1|g.218 ORF TRINITY_DN1013_c1_g1|g.218 TRINITY_DN1013_c1_g1|m.218 type:complete len:457 (+) TRINITY_DN1013_c1_g1:226-1596(+)

ATGAGCATGGCTGAAACCATGCCCTCCCTCCCCTTCACCAACTCCAACCCCAAGAAACGC

TCCTCCAAAGACATGGAAGACAACAACGACGACCACTGGATGTCGTCGGCTGCCGCGGCG

AAGAAGAAGCCCGTGGCCGACCCGGCCCAGGCGCTGGCGAGCGCCCGGCACGAGTTCGGC

GAGCACGGCGGAGTGAACATGTCCATCGAGGCCTCCGCCACCTTCACGGTGATGGAGCCG

GACACGATGAGGAGGATGTTCTCCGGCGAGCTGGGCCCGGAGAGCGACTTCTTCGTGTAC

AGCCGCCACTTCAACCCGACGGTCCTCAACCTCGGCCGCCAGATGGCCGCCATGGAGGGC

ACCGAGGCCGCCTACTGCACCGCCAGCGGCATGTCCGCCATCTCCTCCGTCCTCCTCCAG

CTCTGCCCCGCCGGCGGCCACGTCGTCGCCTCCCGGTGCCTCTACGGCGGGACCCACGCA

CTGCTGGCGCGATTCCTGCCGCGGGCCTGCGGGATCACGACGAGCTTCGTCGACGCCGGG

GATCTGGACGCCGTGAGGAGGGCGATTAAAGAGGGCGAGACGAGGGTTTTGTACGCCGAG

ACCATGTCGAACCCGACGCTGGCGGTGGCGGATATCCCGGCGCTGAGCAGGATAGCGAAA

GAAAAGGGGGTCATGCTGGTGGTGGACAATACGTTTACGCCCATGGTGGTGTCGCCGGCG

AGGCTGGGGGCGGATGTGGTGGTGCATAGTGTTTCCAAGTACATCAGCGGTGGCGCTGAC

GTCATCGCAGGTGCGATCTGCGGACCGGCAAGCCTGGTGAACTCGATGATGGACCTCCAG

CAGGGCGCTTTGATGCTCCTTGGCCCGACCATGAACGCCAAGGTGGCCTTCGAGATCTCG

GAGCGCCTCCCCCACCTCTCCCTCCGCATGAAGGAGCACTGCCACCGCGCGCTCACATTC

GCCACCCGCATGAAGAAGATGGGCCTGAAGGTGATCTATCCAGGCCTGGAGGACCATCCG

AACCATGAGCTGCTCAAGTTGATGGCCAACCCAGGGTACGGGTTCGGGGGCATGCTGTGC

GTGGACATGGGCACAGAGGAGCGGGCCAACCGGCTGATGAACCATCTGCAGAACTCCACC

CAGTTCGGGCTCATGGCCGTGTCGCTGGGGTACTACGAGACGCTCATGTCGTGCTCCGGG

AGCAGCACCAGCAGCGAGATGACCGTGCAGGACAAGGAGCTGGCCGGGATCTCACCCGGG

CTGGTGAGGATGTCAGTCGGGTACAGCGGGACGGTGGAGCAGAGATGGGCACAGTTTGAG

AAGGCCATTTCTTGGATGCAGGATGCAAACCCTCACTATGGCAAGAACTGA

>TRINITY_DN10140_c0_g2|m.222 TRINITY_DN10140_c0_g2|g.222 ORF TRINITY_DN10140_c0_g2|g.222 TRINITY_DN10140_c0_g2|m.222 type:internal len:129 (+) TRINITY_DN10140_c0_g2:2-385(+)

CGCCTCGTGGAGTCCTTGTCGCAGTTCCCCTCGGGCGCGGGAAAGATCCCCCGCCTCAAC

GCCGTCGTCCTCGGCGAGTCGATCGCCTCCGAGGAGGACGACCTCGTCTTTCCCAGCGAC

GATTTTTGCAAGCAGGCCCTCGTCTCCACCCCAAAACAATATCTGGAGATGTACAAGAAG

TCAATAGAGGATCCCGCGGGGTTCTGGTCCGAGATTGCATCGCAATTCTACTGGAAAGAG

AAATGGAGTCCCGAACTGTATACTGAAAACCTTGATGTGACAAAGGGGCCTATTAAGATC

GAGTGGTTCAAAGGAGCAAGCACAAACATAAGCTACAATGCTTTGGACCGAAACATTGAA

GCGGGGAATGGTGACAAGATCGCG

>TRINITY_DN10141_c0_g1|m.223 TRINITY_DN10141_c0_g1|g.223 ORF TRINITY_DN10141_c0_g1|g.223 TRINITY_DN10141_c0_g1|m.223 type:5prime_partial len:507 (-) TRINITY_DN10141_c0_g1:474-1994(-)

CCGGTTCGGGCCGTCTGGGTTAAAATAAGTCCCCACCCATCTTTCTCCACTAAAACCCTA

GCTCATCCCAAAACCCCTCTCTCTTACTCTCCATCGACCACCGCCAGCCTCCGATCGAGC

AAGATGCCTAGAACGAGCGCCGTTGAGAGCCCCGGTTGCCCGCCGCTGAGAGCCCTAACC

ACCGACATCCTCGGCCTCATAAAGGTTGTCGAGGCTCGAGGGAAGGCCGGGCCGCTGAAG

GTCGTCGAGACGTGGGGCCCGCCCGACGCCTCGCGGGGAATTCTCGCGGCGTCCTACGCA

GACGACGAGAACCGACCGCTTATAGCTGTTGCACGGAAAAATAATATGATTGAAATTGTT

AATCCTTTGAATGGACATCCTCTTACATCAAACAAAGTTAGTGAATGTAGTACTGTAGGG

TGTGCTGTTGAAGATGATCCAATTGTTGGATTGCATCTCCTAAAGACAAAAACTGAGTTA

TCCTCCAGGTCAAACAAGCTCCTTTTGTGTACGGAAAAAGGAAAAGCAAGCCTTAGGTCT

GTTTCCATGGCTGATGCATCAGCGGCCTCTACAACTACTGTTTCTCAAAGTACATGGGAT

GTATGCGCTGCTGGTAAAGTGACATGTTCTGCAATGACTGCAAATGAGAAATATGCCTTA

TTTGGAGGGAAGGGAATCGAAGTGAATGTGTGGGATCTTGACAGTTGTAGTAAAACCTGG

ACTGCAAAACCTCCTCCATCTAACAGTCTTGGTATATTCTCTCCAACATGGTTCACTGCT

GCAACTTTTCTGAGCAGAGAGGATCATCATAAAATTGCAACTGGTACGAACAATCATCAG

GTCCGCCTTTATGATCTTTCTGCGCAAAGAAGACCTGTTATATCAGTCAATTTCAGAGAG

TCAGCAATTAAGGCAGTCACTGCTGATCTAGATGGTCATACAGTCTACATAGGGACTGGT

TCTGGAGATCTCGCTTCCTTTGACATGCGAACAGGGAAATTACTAGGATGCTTTGTAGGT

AAGTGTTGCGGAAGCATTAGATCCATAGCCAGGCACCCAGAGCTACCTATACTGGCTTCA

TGTGGTCTTGATAGCTATTTGCGTATTTGGGATACGAAGACCAGACAACTTCTTTCTGCA

GTCTTCCTCAAGCAGCATCTCACAAATGTAGTGATAGATTCACATTTCTCCTATGAAGGC

AATTCTTCTGATCAACCAAATGATTTGCATGCGGTTGAACAGACCAAAGCTGATCTTAGT

GATAATGATGATGACGATGAATCATCACTCTTCCAGAAAAGCAAGAAATCAGAGAAAGGG

GAGAAAAACAAGTCAAAGAGCAAGACAAAGAAAACCAATAAAGAAGTACCTGAAGACGAT

CAGATAACGGAAACAGGCCTTCTTAGCCTTAGTGAAGATGAAACCGATAAACTGCCAGCT

TTGAAACGAAGAAAACCATATTCAGAAGGTAAAGGGCGAAGTAAAAAGATAAAGAAGAAT

AAGAACAAGGGAGCCACATGA

>TRINITY_DN10142_c0_g1|m.225 TRINITY_DN10142_c0_g1|g.225 ORF TRINITY_DN10142_c0_g1|g.225 TRINITY_DN10142_c0_g1|m.225 type:complete len:165 (-) TRINITY_DN10142_c0_g1:434-928(-)

ATGGCTCCTCTTGTGTTGCTTTTGGTGGGTATTCTCCCATTGTTCTCTCCTGCATATGGT

TATGGGCGTGGAAGCAGAGGAGGAGGATGGACCAATGCGCATGCCACCTTCTATGGAGGG

GGTGATGCCTCTGGAACAATGGGTGGGGCTTGTGGGTATGGGAACCTGTACAGCCAAGGG

TATGGAACAAACACAGCAGCTCTGAGCACAGCCCTGTTCAACAATGGGCTCAGCTGCGGG

TCCTGCTATGAGATCGTGTGTGTGGGTGATCGCAAGTGGTGCCTGCCGGGCGCCATCGTG

GTCACTGCCACCAACTTCTGCCCGCCGAACAACGCTCTCCCCAACAATGCTGGGGGGTGG

TGCAACCCTCCTCTTCACCACTTTGATCTCTCTCAGCCTGTCTTCCAGCACATTGCTCAG

TACAGGGCTGGGATTGTTCCTGTTGCCTACAGAAGGTACGTTGGTATTTCATCAGTTTTA

GACTTGGTTGGTTAG

>TRINITY_DN10145_c0_g1|m.227 TRINITY_DN10145_c0_g1|g.227 ORF TRINITY_DN10145_c0_g1|g.227 TRINITY_DN10145_c0_g1|m.227 type:complete len:641 (+) TRINITY_DN10145_c0_g1:102-2024(+)

ATGCTGCTGCGTGGCTTCGCGCACGCCTCGTGCTTTGCTCGCGCGCACGACTCTCCCCCC

TCCTTCGCTCCCTCAGCTCAAATCTCGTCAATCGAAGACAGGAAGACCTCAGTCTCCTTC

TCGTATCCCAAGACGACGATGACGACGACTCGATCAACGTCGTGTTCGTCATCGGGCCCT

TCGTCATTAGCTTGCCGAGCTTCAGCCCATAGCCCGAACCATCGAGAGGATGGGTTGTCG

ATCCATGGAGTGTGTGAGACTGTTGTCGGCATTCTGGGAGGTGGACAGTTGGGTCGAATG

TTATGTCAAGCTGCAAGTCAGATGGGAATCAAAGTGATCACCTTGGATCCCCTTGAGAAC

TGTCCGGCTAGTGGTGTCTCCCATCAGCATATCGTTGGAAATTTCGATGACGGTGCTGCG

GTCCGTGAGTTTGCGAAGAGATGTGGGGTGCTGACTGTTGAGATTGAACATGTTGACGCC

GATGCATTGGAGAAACTTGAGCAACAAGATGTAGATTGCGAACCGAAAGCTTCTACGATA

CGGATTATCCAAGACAAATATATTCAAAAAGTTCATTTTTCAAAGCATGGAATTCCACTT

CCTGATTTTATGCAGATAAATAATTCAGAAAGTGCTGAGAAAGCTGGTGACTTGTTTAGT

TACCCTTTGATGATCAAAAGTAAGAAGTTAGCATATGATGGGCGAGGGAATGCTGTTGCT

TACAGCAAAGAGGAGCTTCCTTCTGCTGTTAATGCATTGGGAGGCTATGATCGTGGGCTA

TATGTTGAGAGATGGACTGAATTTGTCAAGGAGCTGGCAGTCATTGTAGCAAGGGGTAGA

GATGGTTCAATTTTATGCTATCCAGTTGTGGAAACTATTCACAGGAATAACATATGTCAC

ATAGTTGAAGCTCCTGCTGATGTGCCTGAAAACATAAAGAAGAGCGCTGTTGACGTTGCA

CGAAAAGCTGTTAGCTCCTTAGAGGGGGCTGGTGTTTTTGCCGTAGAATTATTTTTGACA

AAGGATGGAAAGGTTTTACTAAATGAAGTTGCTCCTAGACCGCACAATAGTGGCCATCAC

ACAATCGAGTCTTGCTACACCTCTCAGTATGAACAACATTTGAGAGCTGTGATTGGTCTT

CCACTTGGTGATGCATCAATGAAGACACCAGCTGCAATCATGTACAACATACTGGGGGAA

GATGAGGGTGAACCTGGTTTCTATTTAGCTCACCAGCTGATAAGAAGGGCATTATATATT

CCTGGGGCTGCTGTTCATTGGTATGACAAACCAGAAATGCGGAAGCAACGGAAGATGGGA

CATATCACAATTGTAGGGCCTTCAATGAACAAAGTTAAAGAACGCCTGAATTTATTGTTA

GAGACAGAAAATGTTGGAGGTCACACTGCAGTTACGTCACACGTTGCTATTATAATGGGA

TCCGATTCGGATCTCCCAATCATGAAGGACGCTGCAGAAATTTTGAAGACTTTTGATGTG

CCATTCGAGTTAACAATTGTTTCGGCGCATCGAACACCTGAGAGGATGTACTCTTTTGCA

TTATCTGCTAGAGAAAGAGGAATTCGAACCATCATTGCTGGTGCTGGTGGTGCAGCACAT

TTGCCAGGCATGGTAGCTTCGTTGACTCCATTACCTGTTGTAGGGGTCCCGGTAAAAACC

TCGTCACTTGGTGGAGTTGACTCCCTCTACTCCATTGTGCAGATGCCAAAAGGCGTCCCA

GTTGCGACAGTTGCAATCAATAATGCGGCCAATGCTGGTTTACTAGCAGTCAAGATTTTG

GCAACAACGGATTCTGATCTGTCGGACAGGTTGATCAAATACCAAGAGGACATGAAGGAC

ACTGTTTTGACTAAAGCGGAGAAGCTTGAGGCTGAAGGCTGGGAACGATATTTAAATCCT

TGA

>TRINITY_DN10147_c0_g1|m.229 TRINITY_DN10147_c0_g1|g.229 ORF TRINITY_DN10147_c0_g1|g.229 TRINITY_DN10147_c0_g1|m.229 type:complete len:336 (+) TRINITY_DN10147_c0_g1:157-1164(+)

ATGGCGGTTCTGGTCACCGCTCCGATGAGGCTCAGGGTTAAGGCTAGGGTTTCGGGGAGC

GAGTTCCCGCCATTTCTCCCCAAGGAGGTGGAGAAGATCAGGGACCCGCCGGCGCGGGAG

CTCGCCAAGAGGATCCAGCGACTCCCCGTCAACGTAGGCTTCTTGAAAACCTCTATTGCA

AGTAGCTGTGTGAAGCCATTGAGACATAAGGGAACGGAACCAGTCGTTCTACTTCATGGC

TTTGACAGTTCTTGTTTAGAATGGAGATATACATATCCGCTGCTTGAGGATGCTGGACTG

GAGACATGGGCACTGGATATCCTTGGATGGGGTTTCTCAGATTTAGGAATTTCTCTTCCA

TGTAATGTTGCTGCCAAACGTGAACACCTCTACCAGTTTTGGAGGTCATATATTGAAAGG

CCCATGGTATTAGTTGGACCAAGCCTTGGTGCTGCAGTTGCAATTGATTTTGCAGCTAAC

CATCCAGAAGCAGTGGAGAAATTGGTTCTCATTGATGCGAGTGTGTATGCAGAAGGCACA

GGAAATATGAAAAAATTACCAAAAGCAGTAGCTTATGCTGGAGCAGCGATATTGAAGAGT

TTCCCGCTGCGGCTTTATGCGAATTCCTTGACCTTCAGCAACATTTCATTTTTCACCTCC

CTTGACTGGACAAATGTGGGTCGGCTACACTGCTTATTCCCTTGGTGGGCGGATGCAACT

GTTGACTTTATGATTAGTGGGGGCTATAACGTTGGCCATCAGATAAAGCAGATTAAACAA

AAAACACTTATCATATGGGGTGAGAACGACCAAATTATCAGTAACTCACAAGCGCAGAGA

TTAGACAATGAGCTCCCAAATTCAGTTTTACATCAGATACCAGAATGCGGTCATCTTCCT

CATGTAGAGAAGCCACAGTTTGTAGCCGAGTCGATCCTGGAGTTCCTTCGAGGAGATCAC

AGCTTAAAAACCACATATGCATATTCTCAAAGTATTGCGAACGTGTAA

>TRINITY_DN10148_c0_g1|m.230 TRINITY_DN10148_c0_g1|g.230 ORF TRINITY_DN10148_c0_g1|g.230 TRINITY_DN10148_c0_g1|m.230 type:complete len:544 (-) TRINITY_DN10148_c0_g1:185-1816(-)

ATGGCACTATCAAATCTCTCATCCTTCTGCCTCTTCTCTCTCTCCACAACCCTTGATCCC

TCTACCACCAGCAGCGGCACCATCAGAAACCATAAACCATTGCCTAGTATCATCTTGAGA

AACAACAACAACAATAACAGATCAACAAGAAGAAGAAGGTTGGGAGAGTGCAGAGCTGCA

ATAGTCGAAGATACCGATAAAGAAGTACTGCTTCCTCCAGAAACAGGAGAAGAAGAGAAG

AAGCAAGAAGCTGCGGAGTATGACTGGAGAGAAGAGTGGTACCCTCTCTACCTCACGGCC

GAAGTCCCCGACGACGCCCCTCTCGGCCTCACCGTCTACCACAAGCAGATCGTGCTCTAC

CGCGACGGCCAAGGCGTCCTCCGCTGCTACGAGGATCGCTGCCCCCACAGGCTAGCCAAA

CTCTCGGAAGGCCAGCTGATTGATGGCCGGCTGGAGTGCCTGTACCATGGATGGCAATTC

GAAGGCGACGGCAAATGCGTCAAGATACCTCAGCTACCAGAAGGAGCAAAGATACCTCGG

GCAGCTTGCCTGAAGACATACGAGGTGCGAGACTCCCAGGGAGTAGTATGGATCTGGATG

TCGGAAAAGAATGCACCAGACAACGATAAGCTGCCATATTTTGAGCACTATGCTCGTCCG

GGATTCCAGGATGTGTCGACGATCCACGAGCTCCCCTACGACCACTCGATATTGCTGGAG

AACCTCATGGACCCTGCACACGTTCCCATCTCCCATGACAGAACGGATTGGACTGCCAAG

CGTGAGGATGCGCAGCCGCTTGTCATGACAGTCACCGAGAGAACCAACAGAGGGTTTGCT

GGACAATGGGGGAGGTCAAGGAACTTGAATCTCATGAATAATCTGAGGTTTGAAGCGCCA

TGTGTCTTGCAAAACAATGTGGAATTTGTTGATAAGAAGGGAGTGAAGCAGTACTTCTCG

GCTTTGTTTCTGTGTCGACCAGCCGGGCAGGGGAAGTCCATGCTGATCGTTAGGTTCGGA

GCGAGCACGAGGAGTTTTCTTGCCAACTTGCTGCCCAAATGGTACTTCCACCAGAATGCT

GGTAAGGTTTTCGAGCAGGATATGGGATTCTTGTCATCGCAGAACGAGGTGCTGATGAAG

GAGAAGGTTCCGACAAAGCAGTTGTATCTCAATTTGAAGTCTTCGGACACTTGGGTTGCC

GAGTACAGGAAGTGGATGGACAAAGTTGGGCATGGCATGCCTTATTATTTCGGGCATAAT

ACAATTTCGCTGCCCAAAGTGCCTGCAGTGGTTGAGCAGGCTCCTGCAGGGCTGGCAGCA

GGGATTTCGGCTTCTTTTCCGGCGAAAGGGGGTTTCGGAACTCACCACGCTCCGAATCCA

GTGAACCGATACTTCAGACACGTAATTCACTGCAAGGGATGTAGGAAAGTGGTTCAGGCA

TTTCAAACATGGAAGAACATACTGCTGTGCCTGTCTCTTGCTTCTGCTGCAACGGCAATT

CTTGCTTCGACAAGGCAGTGGAAGGTTCTTTTCGTGGTATCTGCAGCATTTTTAGCGGCC

ACATCATATGCTTGTTCGAGTGCAGTTTCGCTCATCACTACAAACTTCATTAGAACACAC

AGAAGATTGTAA

>TRINITY_DN10149_c0_g1|m.232 TRINITY_DN10149_c0_g1|g.232 ORF TRINITY_DN10149_c0_g1|g.232 TRINITY_DN10149_c0_g1|m.232 type:complete len:512 (+) TRINITY_DN10149_c0_g1:175-1710(+)

ATGCAGCCCCAAGGATCTAACCGTGCGTCGCTGCTTGCCAACCTGCGCACTGGCGGTGTG

CGCTCTGCGTCTGGTGTCCCGCACACTGCTGGCCCCACTGGGTCTTTTCAGATTCCCCGA

TTTGCGTCGCAGGCCGGATACAACAATGCGATCTACGAAGACGACGAAGGCATCTCTGCC

CAGATGCAGCAGATGAACTTTAACAATGCCGCCATGGGTCCGCGCTCCATGATCAACGGC

GCTCCGATGACGTCCGGGCTGGCCGGCAACTTTCAGCAGCAGCAGCAACAGCAGCTCTTG

ATGATGCAGATGCTCCAGCAGCAGGGAATGGGCATGGGTCTGAGCGCCGACCAACAAGCT

CTGCAGCTGCAGCTCCAAAACGAGATCTTGATCCTTCAGGCAAAGCAGCAGCAGCAACAG

CAGCAGTTCCAGGCCCAGATCCTCGCTCAGGCGCAGGCTCAACAGGCTCAGCTGCAGGCC

CAGATCCAGGCTGCCCAAGCCGCCCAGCAGGCGCAGTCGGGTCTCTCCACTCGTTTGAAT

CCCGTCATCACTCGTCGCACTTCGGCTGCCAATACCGCTGTCCCGTCGTCTGCCGGCCCG

CTCACCACGTCGTTTAATCTCGGAAATGTCCCTCGCCGCACCATGTCCGTGGACCAGGAT

ATTCCTCAGCCCCCCATGACTGCCGGCCTTGGAGGTCGCTTTGGCGCACGTGCGACTCAG

CCTCCGGGACTCAACCCCAACGCGGCCGCATTTTCGTTTGGTGCTCGCGTTGCGTCCCCA

GTCAAGGAACATGCCGAGAGCGGAAGCCCGCCAAGCAACAATCCTCTGGGCAACTATGCT

GCCACGACCGTCATTAGCGGTGGCACGGCCCTTGGTGGAGCCGCCCCAGCACCCAACCGG

GCCCAGGCCGACATGTCTTGGCGTCGTCCGTCGGTTGCTGCCTCGCCCGAACCCCAGCGC

CAGGCTACCAACAGCCCCCCGTTCCGCGATCAGTCTCCGGTTTCTGCTGGCTCCCGTGAC

AACAGCCGAGCTCGCCCGCAGCCTCTGCGTTTCAATTCCGCTCTGTCCACGGCCGTGGAA

GCTCCGCAAAGCACCCCGAGCTCTCCCGAGCCTGATGTGGTTACCACTGGCACCGACGGG

GTCCAGACGATCCTGGTTGACAATGGACACAGCACCGGAAGCCGCAGCCCTTCTTCCAAT

TCGCCCACGACCCCTGTGGGTGGAGAGCCCCTGCCGGCTTCGCGAGAGGAAGCTTCGAAG

CGTCTGTACGAGGGTCTCGGTGTGGGTCGCCCCATCCAGACTCAATTGGCTTCGGCCATG

GGATCCAGTGCCTCGCTCACGAGCATGAGCGGAACGTCGACCCCGGCCCGTGGCGTTTCT

CAGGCTGTGCGTCAGCCTCGCGGCCCCCCGACCGAAGTAAGCGAGCTTGGCCCCCGCAAT

TTTGCCTCTCGCGTGCGTTCCAAGGCTATCGGTGGACTCGAAGTGATGATGATGGCCCGC

AACCGTCGCCAGGGACTCGATGTCGAGGCCTTCTGA

>TRINITY_DN10150_c0_g1|m.238 TRINITY_DN10150_c0_g1|g.238 ORF TRINITY_DN10150_c0_g1|g.238 TRINITY_DN10150_c0_g1|m.238 type:complete len:1133 (+) TRINITY_DN10150_c0_g1:78-3476(+)

ATGGTCTCCTCTTCGTTTCCCGCGGGATCCGAGGTCGAGGTTCGCTCCAACGAGGAAGGA

TTCAAGGGCGCCTGGTTCGAGGCGACGGTCCTCAAGCCCCTCCCAAAGTACCGCAGGTAC

ACCGTTTCGTACGCCTCCCTCGTCTCCGACGAGCTCACCGGCAAGCCCCTCGAAGACACC

GTCTACTACTCCTTCGTGCGGCCGCGGCGGCCGCCGCGACCGGCCGCCGCCGCCGGATTT

GAATTGCATCAGCTCGTCGAGGCGTATCATAACGATGGGTGGTGGAAGGGAGTGGTGAGC

GAAGTGCCCAAGGCGAGGAGTGGGCTTTATAGGGTTTGCTTCCCGAGCTCGAGAGAGGAG

TTTGAGTTTAAGGGCGAGGAGCTGAGGGAGCAGCTGGATTGGGCTAAGGGCAAATGGGTT

GCTGTTAAGGATCTAGAGGTAGATGAAGATGATATTATGTTTGATGTGGGAAGTCGAGTA

GAAGTAAGTCGGGACAAAGAGAACTATGGAGCAGCTTGGTATGTTGGGACTGTCTTGAAG

TTTATTGTGAAGAACAACTTCTTGGTGGAGTACGAGAGTCTAAGAGTAGATAATGATGGT

AGCAGTGGATTGCTCCTAAAGGAGATTGTTGATTTGCAGTACATTAGGCCCTCCCCCCCT

CCTGCATCGGAGTTTGATAATTTCATCGATATCTTAGAAGAAGTCGAGGCATTTCATAAG

GGTGGGTGGGTAGCTGGGGTGGTCTCGGAGCTTCGTCGTGGGTCAAGGTACATTATCAAG

TGCAAGCACCAGGACAACGAGATTGAGCTTGACCGAATGGCAATAAGGCCTTGCTTTGAG

TGGATAAATGAGCACTGGGTTCGCATTTCTCGGGAGAAGAGTAGCAACACAATGTCTGCA

ATGGGAACAAGAACATATAGTCGCAGACGGAAACCCCGTGCTATTAAACTATCCAGTTTA

CCTATTCCAGGACCAAGTTTTAGTGGTAATGATGATGAAGTTGCTAGGAGATCTGAAGGT

TCCAGTATGAAGGCTAAGAAGTATGAGGATGCCAAAGTTGAAGGCAGGGTTGGGACAACT

CAACCTTGCAAAAAGTTGAAAACAGGAAAATCGCTAGAAGAGCACCTGAAGTTGGTAGAT

GAAACGAATTCAGTTGGTCGCAGACGTAAACGGACATCCCGCACTTGCAAACGGTCTACT

CTATCTATTGCAGCCCTTTCTTCCCGCAATAGTAACAGTGACGTTGATGACAGATCTGTT

ACTGACAAATTGATGGATAAAAAGGTCGAGGAGTCCAGTATAGAAGAAAGGATCAACTCT

TCTCAATCTCACGAGAAGTCAGAAAAAGGAAAATCTCTGGAGGAACACTTGCAGCTGTTA

GATTGCACATTGATGAATTGTGGTACTAAAAGCCCCAGAATGTTACGGAAGAGATCCTCT

GTTGGGACGATGCTAGAAGGCAGCGGAGCATTGGCTACTGGACAAACTTGTTCGAAGAGT

CCTCGTCGGAGAGTAGGAACAAAATCAGCAGAACATGAAAAAGTTATATCTAAAGTGAAT

GGTAATGACTCAGTGGACAATGTTGAAGGATCTTCTAGTTGCTTGGGTGATGTACCTGAA

AATCAGTCAAAGGTTGCTGATTTTGGCCGTTCAACAGCTGGAGGTGAGCCTTCTGATTCT

CTTCCAGTTGCATGCTGGGGAAACCATAAGGTTGATTATGGTGTGACAGAAAATACGAAG

ACACGGAGAAAGAAGCTAGTCATATGGTCATCACGTCACAAAAGAAAGACTCTTGATGTT

GATGATGTGAAACAGCAAGTACAGGAGGAGACGGAGCAGCATGTACAGGAGGAGACACAG

CAACGTAAGAGAGGCAGACCTCCAAAGAAGAAAATTAGTAACTTGAATGTAGGAACAGAA

TCTCAACAGTTGGAAGAAAGGAAAGTAGTGGGAGGTGAAGATAGGACGGAGTTTGTGGAA

CCATCCAACCAAACACCGACTGTTCATGAATCAGACAAAGTTCAATCCCTATGCCATGAC

AAAGTAATGGAGAGCAAGAATTCTCTGTCGACTGTCAATATGGATGGTATGTCTGAATTA

CCTAATATTTGTGTAGGACACAGACCTGCTAATGATGTTACCGGTATTTGTTTGTACAAT

ATGCAAGCAGATATCATGGACAATTCTCCTGAATCACCCCAAATAGATTCTTCCATTCTA

GTTACCAAATCGCTTCAGTACTTGACTATCCAGCAATCGCCAACATTGAAATCTTTCAAT

GGTAGACTTGTTGAGACTGAAGCTGAAGAACGAGCAGAGGTCTTTCCTTCTGAAGAAGAT

ATTCATGATCATGACAAGCTTATTTTAAATGGATTAAAGAATACGGTTGAGATGGAGAGG

TCACCTGTTGATCCTACACCTGATGGGCTGTCACTTCTCAAGGAATATAATGCTTGTGAT

CAGAATGAGTTAACAACTAGTCGGTCATTTTCTAGAAAGGCCTATGCTGAGATTGAGGAC

AGGGCACCCATTGATCACAGCCCTAACGGGTCATCACTTTCAAGGGAAGAGTATGTTCCT

GATGGCAAAGAGTCTACGCCAAGTAGGTCACTGTCAGAGAAGACTGTTGTCAAGATGGAA

ATGATGCCAAATGGTCTCAAATCTAATGGGTCGTCACCTTCCAAGGAAGATGTTTATGAT

CATGATGAGCTTACTCCCAGTGGATTGCCATCTGAAAGGGCTATAGTTGAGGCAATGGGG

AGGACTGAGATTTATCCCATGCCTAGCATGTCATTATCTAGCGAGGTAGCATTGCCTTTT

ATGAAGGAGTCTTCAATGTGGGAGATTGTTGACTCAATGGAAGTTTTTCGCACAATGCCA

CAACAGCCACATTTCCAGCCCTTGGAGCAGTATGAGGTAGAATTTCGTGAAGGAATGGCT

ATTGGTTTGATGGTGACCTTTGCCAATTTAGTTTCCAGTATTCAGAAGCTGCAAATTTCT

GATTCTCAAGATATTTTTGAAGAAAAATTAAAGGCCCTTGTGCCCTTGGAGTTACATGGT

TTCAATATTCAATTTGTCCGATCTCGCCTTGAGAAATTACTAGAGATACGAAGTAATGTT

GGTCAATCTGAGGGCAATAAAGGGTCTTTGAAAGAGAGAATCCTGGAGACAGTGGACGAC

AAAGAACAAATTGATGCACATATTGCCGCACATGATAAAATTATCCTCGAACTGGAAGAA

AATCTCTATCGCTACAAAGAGAGAAGGACATCCATGATTTTGGCAAGGAACAGAAAAGAT

TCTGAAATTGCTCGATTGAAAATGGATTTGCAAGCAACTGAAGAAGCCTATTTTTCTGCA

GAAGAACACTTTAAAGCTGTTGCAAATGCTCCATGGTAA

>TRINITY_DN10153_c0_g1|m.240 TRINITY_DN10153_c0_g1|g.240 ORF TRINITY_DN10153_c0_g1|g.240 TRINITY_DN10153_c0_g1|m.240 type:internal len:109 (+) TRINITY_DN10153_c0_g1:3-326(+)

CGGAATGTGAGGCGGTTGCATACTATACTTACCTCGAGAGATTCCATGAACAATGTCCCT

AAGAATCTGGAGGCCCGTCGCCGTATTGCTTTCTTCAGTAACTCTTTGTTCATGAACATG

CCTCGGGCTCCCCAAGTTGAAAAGATGCTGGCCTTCAGTGTTCTCACTCCATACTATAAT

GAAGAAGTTTTGTACAGCAAGGAACAACTCCGAACAGAGAATGAAGACGGCATCTCAATC

ATATTCTATCTTCAAAAGATTTATGATGATGAATGGGTGAACTTTCTGGAACGGATGAAG

GAAAATGGATTTGCTGATGAAGAA

>TRINITY_DN10155_c0_g1|m.241 TRINITY_DN10155_c0_g1|g.241 ORF TRINITY_DN10155_c0_g1|g.241 TRINITY_DN10155_c0_g1|m.241 type:5prime_partial len:402 (+) TRINITY_DN10155_c0_g1:2-1207(+)

GATACCCATGCGACACCACCACCCCCATGGCTTGGTGGATATAGAAATGAATCATTTGAG

TATGGTCCGAATAATTACAACAGTAGCCATATTGGTCTTGATGGGGAGGCACCTGAGGAA

TTCTCTATGCGACTTCTGTGTCCCACTGGAAGAATTGGTGGGGTGATTGGCAAGTCTGGT

GCCACTGTCAGACAGTTAGAGCATGAAACACAGGCTAGAATTCAAGTCGAGGACACTTCT

CCAGAGGCTGAAGAACAAATCATTAACGTGTCCTCTAGGGAGGGTCCTTGGGATCAAATT

TCCCCAACTATTGAGGCTATTCTTCAGCTTCAAGTCAGAACGTGTGCATCCTCTGAAGAA

GGTACCATCACCACAAGGCTTCTTGTTCCATCAAGTAAAGTTGGCTGCCTCCTTGGACAA

GGCGGTACCGTAATCTCAGAAATGAGAAGGCGTACTAGAGCAGACATTAGAGTTTTCTCT

AAGGAGGACAAGCCGAATTATACATCTGCTAATGAAGAGCTCGTGCAGATATCTGGAAAT

CCTGATGTGGCTAGGGAGGCTCTCTTGGAAATTGCATCAAGGCTTAGAACGAGGACTTTC

CGAAGTGGGAATGCCACTGTAAATCCTGCACCTATGGTCCCAGACCGTGGATTTGCTCCA

CCTTCAGAGAGGATGTCTGGTAGAGGTCTGCCGTCATCTCGCATGTTTGGAGCTGCTGGA

AATCCGCCGTCTGTATATCCTGCCCAACGATTTGCTCCAGCTGAAAACATCTCCAGTAGA

GGACCGCCATCATCCCGCATGTTTAGAGCGGGTAACACTGGTAACTATGAGTATCCAGAG

TATCCAAAGGGACCTGGACCTGTATATGATTCACAGGCTTATCCTCCTGGTCCACCAGCA

GCTGCTTCTGGGTACCCAAGCGTGACCAGCACAATGGAGATCAAGATTCCAAACAGTGCT

GTGCCTTCAGTTCTTGGCGTGGGCGGTAGTAATCTTTCTGAAATTTATCAGATTTCTGGA

GCTAGAGTGAAATTACAGGATCCTATACCTGGTTCTTCAGAATGCGTGGTCGGGCTACAG

GGGTCTTCTGAGCAGTTGAAGGCGGCGCAGAGCCTCCTTCAGGCCTTTGTAGCATCTGGC

GGTCAGAACATACACCAACCTATGGCACAACCTCCAATGCAACCTCCCCTGTACCGCCGA

TACTGA

>TRINITY_DN10155_c0_g2|m.243 TRINITY_DN10155_c0_g2|g.243 ORF TRINITY_DN10155_c0_g2|g.243 TRINITY_DN10155_c0_g2|m.243 type:complete len:682 (+) TRINITY_DN10155_c0_g2:126-2171(+)

ATGGACGATGATGCTTGCGGGAAATCTAGATCGGGTAGTTTCAAGAAAAGATATCATAAT

CAGTTGGATAATGGGAAACGGAAGAGACACAATCTTTATCCTAGCCATGATTATAGTTCT

GGTACCCCTAAAGTTATTGAGACGGTTTACCGCATCCTTTGCCCTGTCAAAAAGATTGGT

AGTGTTCTTGGCAAAGGGGGTGGAATTGTTAATGCTCTCAGAGGTGAAACCCATGCAAAG

ATAAGGGTCGCTGATGCCATACCTGGGGCGGAGGAGAGAGTGATTATCATTTTTAATTAC

CCATCAAACCTATCAGGAAAAAGTGACGGTGATTGGGGTCCTGAGGTTGATGACTTTGCT

GAGAATGAATCTGATGACATGCGTCCTCATTGTCCTGCTCAGAATGCTCTTATGAAGGTC

CATGAAAGGATTGCTGCCGACGAGTATGCAGGTGGTGGAGTGGTGCGTGAAAAGACTGAC

CCTGATGTTGGTGCCACTGCTCGCATATTGGTTCCAAGCAATCAAGTCGGGTGCCTTCTT

GGTAAGGGTGGAACCGTAATAAAGAAACTTCGAAGTGATTATACTGGTGCAAATATCCGT

GTTTTGCCTTCTGAACATCTTCCACCTTGTGCCATGAGCACTGATGAATTGGTGCAGATA

TCCGGAACACCAACTGTTGTGAAGAAGGCTCTATATGATATTTCTACTAGGTTGCATGAA

CATTCTCGTAAAGAAAACCCTCAGATTGATGATCTTATATATGCCAGTACCCAAGGATTA

TTTCAGCCTGGTGCTTCCATGGCTCCGCCACCTCATGGAAATCCTATGTGGTCGCAGCAT

GATACCCATGCGACACCACCACCCCCATGGCTTGGTGGATATAGAAACGAATCATCTGCG

TATGGGCCGAGTAATTACAATAGTAGCCATATTGGTCATGATGGGGAGGCACCCGAGGAA

TTCTCTATGCGACTTTTGTGTCCTGCTGGAAGAATTGGTTGGGTGATTGGCAAGTCGGGT

GCCAATGTCAGACAGTTAGAGCGTGAAACACAAGCAAGAATTCAAGTTGAGGACACTTCT

CCAGAGGCTGAAGAACGAATCATTAATGTCTCCTCTAGGGAGGGTCCTTGGGATCAAATT

TCCCCAACTATTGAGGCTATTCTTCAGCTTCAAGTCAGAACATGTGCATCCTCCGAAGAA

GGTACAATCACCACAAGGCTTCTTGTTCCATCAAGTAAAGTCGGCTGCCTTCTTGGACAA

GGCGGCAGCATAATCTCAGAAATGAGAAGGCGCACTAGAGCAGACATTAGAGTTGACTCT

AAGGAGGAGGACAAGCCAAAGTATACATCTGCTAATGAAGAGCTCGTGCAGATATCTGGA

AATCCTGATGTGGCTAGGGAGGCTCTCTTGGAAATTGCATCAAGGCTTAGAATGAGGACT

TTCCGAAATGGGAATTCCGCTGGAAATCCTGCACTTATGGTCCCAGACCATGGATTTGCT

CCACCTTCAGAAAGGATGTCTGGTAGAGGTCTGCCACCATCTCGCATATTTGGAGTTGCT

GGAAATCTGCCTTCCGTATATCCTGCGCTCGGGTTCGCTCCAGCTGAAAACATCTCTAGT

GCTGGCCCGCCATCTTCCAGCATGTTTAGAGTGGGTAACACTGTCAGCTACGAGTATCCA

GAGTATCCGAAGGGACCTAGACCTGTATATGATTCACAGGCTTACCCTCCTGGTCCACCA

CCAGCTGCTTCTGGGTACCCAAACATCCGTACAATGGAGATCAAGATTCTAAACAGTGCT

GTGCCTTCTGTTCTTGGTGTGGGCAGCAGTAATATTTCTGATATTCGTCAGATTTCCGGA

GCTAGGGTGAAATTACAGGATCCTATACCTGGTTCCTCAGAATGTGTTGTCGAGCTACAG

GGGTCTTCTGAGCAGTTGAAGGCAGCGCAGAGCCTCCTTCAGGCCTTCGTAGCATCCGGT

GGGCAGAACGCACGCCAACCGATGGCACATCCTCCAATGCAACCTCGCCTGTACCGCCAA

TACTGA

>TRINITY_DN10155_c0_g2|m.246 TRINITY_DN10155_c0_g2|g.246 ORF TRINITY_DN10155_c0_g2|g.246 TRINITY_DN10155_c0_g2|m.246 type:complete len:104 (+) TRINITY_DN10155_c0_g2:820-1131(+)

ATGATATTTCTACTAGGTTGCATGAACATTCTCGTAAAGAAAACCCTCAGATTGATGATC

TTATATATGCCAGTACCCAAGGATTATTTCAGCCTGGTGCTTCCATGGCTCCGCCACCTC

ATGGAAATCCTATGTGGTCGCAGCATGATACCCATGCGACACCACCACCCCCATGGCTTG

GTGGATATAGAAACGAATCATCTGCGTATGGGCCGAGTAATTACAATAGTAGCCATATTG

GTCATGATGGGGAGGCACCCGAGGAATTCTCTATGCGACTTTTGTGTCCTGCTGGAAGAA

TTGGTTGGGTGA

>TRINITY_DN101574_c0_g1|m.248 TRINITY_DN101574_c0_g1|g.248 ORF TRINITY_DN101574_c0_g1|g.248 TRINITY_DN101574_c0_g1|m.248 type:complete len:249 (+) TRINITY_DN101574_c0_g1:102-848(+)

ATGACTCCTAAGCTCTTAAGAGAGCTCAAAGAAGCAAACATCTCATGCACATCTCCATCC

TCGGCCGCCACCATCAACCAACAGTCCATGCTAATGTCCTCCTCCGGCGCCATCGGCGGC

CGAGCCATCGACCGCTGCTCGCCGCACCTCCGCGACCCGCTCCGCGCAAAATCCCTCAAC

TCTGTCCTCTACCAGCCCCCTACCAATAAGCTCAAACCCTTCAACTCGAAGGGCAAGAAA

ATTGCTGCCGAGCAACTCATCAGCCCCGCGAGCTCCCAACGTTATCTGCTGAGCGACAGC

AACTCGGATGAGGTCTTCGATTCAGACGCCGGTTCCTTTGTTGCTGTAAATGAACCGTCG

AGGTTTCAGGCTCCGAAGATCGAAGAACAGACTGTCGTCTGGCCGTCGATGGAGACGGCG

AAGATTGAGACTGTGAAGGTTGAAGAAGCAAATGATGCAAAGGCTTCTTCTTCCTCCTCC

TCGTCTGCTGCTAAGCCACAAGAGCAACAGGTTGTGGTATTGAAGGTGTCCTTGCACTGC

AAGGGGTGTGAAGGGAAAGTCAGGAAACACATTTCTAAGATGGAGGGTAAGAATTCTAGA

GTAATTTTTTTTAAAACTTTAAAAAAGTTCCTTTGTAAAAGAAAATTTACCATGAACATT

CAGTTTTTGTGGTATTTTTTTTCCAAAAATATACTGTACTTATTTTATTTTTTTAAAAAC

CCCCATGTGAAAGACCATGCAGTTTAA

>TRINITY_DN10157_c0_g1|m.249 TRINITY_DN10157_c0_g1|g.249 ORF TRINITY_DN10157_c0_g1|g.249 TRINITY_DN10157_c0_g1|m.249 type:complete len:250 (-) TRINITY_DN10157_c0_g1:373-1122(-)

ATGAGTGACAGCAGCCGCAGCCCTTCAAGGGATCGCAAGATTCGTAGAGAACGAGAGTCA

TACCGTGATGCTCCATACAGGAGGGATGAGCGTCGGAATTCAAGGAGACCTGATTTGTGC

AAGAACTGCAAGCGGCCAGGACATTATGCTAGAGAATGCCCCAATGCGGCAGTCTGCAAC

AACTGTGGACTTCCTGGGCATATTGCAGCTGAATGTACCACAAAGGCCCTTTGCTGGAAC

TGCAAGGAGCCTGGCCACTTGGCCAGCAAGTGCCCCAATGAGGGCATCTGTCATACCTGT

GGCAAGGCTGGCCACCTTGCAAGGGACTGCGCAACTCCCCAGCTTCCTCCCGGTGACATG

AGACTCTGCAACAACTGCTACAAGCAGGGGCACATCGCTGTTGATTGTACCAATGAGAAG

GCCTGCAACAACTGTAGGAAGACTGGGCACTTGGCTCGTGACTGCCCGAATGACCCTGTT

TGCAACCTGTGCAACATCTCTGGTCATGTAGCCAGGCAGTGCCCCAAGGGTGATGTGCTG

GGAGAAAGGGGTGGCAGCGGCTATTACCGTGGAGGTGGAGGTGGATACAGGGATGTGGTC

TGCCGCACCTGTCACCAGATGGGCCACATGAGTAGGGACTGCATGGGCCCGCTGATGATC

TGCCATAACTGTGGTGGTCGCGGTCATCTGGCTTATGAGTGCCCATCTGGGAGGTTCATG

GACCGTGTGCATGGGCATCGGAGGTACTGA

>TRINITY_DN10159_c0_g1|m.254 TRINITY_DN10159_c0_g1|g.254 ORF TRINITY_DN10159_c0_g1|g.254 TRINITY_DN10159_c0_g1|m.254 type:5prime_partial len:104 (-) TRINITY_DN10159_c0_g1:1559-1870(-)

CTCCTTCGTCCCCCTTCTCACCTTCACCAACAAAACCATAACCATAATTCTAATTTCTCT

CCTTCATTCCTTCCTCTCTCTCCTTCCTCTGATCCCCCTCCTCCTCCACCTTTGTCATGC

CCGAGGGCACCGACCCTCTCTTTCGCCGCCGCCAGTCTCTACTGCCCTACTTTCTCCTTC

TTATCCGACTGTCGCCGCTCTGACTCCTTCGTCACTGCCGCTCCTTTGGTTGGTCTCCAC

TGCCTAACGGTATACTGGTCTAATCCCAACTGGATTGTTTATAGGCCCAATGAAAGCCTA

ACAAGCCCGTAA

>TRINITY_DN10159_c0_g1|m.252 TRINITY_DN10159_c0_g1|g.252 ORF TRINITY_DN10159_c0_g1|g.252 TRINITY_DN10159_c0_g1|m.252 type:complete len:439 (-) TRINITY_DN10159_c0_g1:174-1490(-)

ATGAAGTTCCGGGCCTTGCTCCTCCTTCCTTTTTCCTACCACCTCCGAAAAAGTCGGTCT

CATCAACCCTTTCCCCTTCTCCCTCTCCAAAACCCTAACTCCTCCCTTTACTCCTCGATG

GCGCCGGAGCCGCCGCAAGAAGGCCCCGACGAGCTCCTCTGCTCCTCCCTCACCGCCGCC

TGCTCTCTCTCCGATAAGTCCCTTCCCGATTCCAGCCCTCTCTCCATCCCTCGCCCTCTG

ACGCCCGATTCCCGCGTCGAGAGAGCGTGGGCCCACTGGAACAGCCTCGGAGCGCCGAAG

CTCGTCGTCGCTCCGATGGTCGACAACTCCGAGCTCCCCTTCAGGATGCTCTGCCGCAAG

TACGGCGCCCAGGCGGCGTACACGCCCATGCTGCACTCGAGAATCTTCTCGGAGAACGAC

AAGTATCGAAGCATGGAGTTCACCACTTGCACGGAGGACCGGCCTCTGTTCGTTCAGTTT

TGTGCGAATGATCCTGATACCTTGTTGGATGCAGCTAAAGTAGTTGAGCCTTACTGCGAT

TACGTCGATATAAATCTGGGATGTCCACAACGAATTGCCCGTCGGGGAAATTATGGTGCT

TTTCTCATGGATAACCTTCCTCTCATAAGGTCACTTGTCGAGAAGCTGGCTGTCAGTCTC

CAAGTTCCAGTGTCATGTAAGATCCGGATTTTCCCAAATCTACAAGACACTTTATCTTAT

GCTAAGATGCTTGAAGAGGCTGGCTGCTCCCTTTTAGCTGTCCATGGTCGTACAAGAGAA

GAAAAGGATGGGAAGAAATTTCGAGCTGACTGGGATGCCATCAGAGCAGTAAAGAATGCT

GTTAAAATTCCAGTCCTTGCTAATGGAAACATACGTCACATGGATGATGTTCAGAACTGT

TTGGAGTGCACTGGCGCTGATGGCGTGCTTTCAGCTGAGACTCTTCTTGAGAATCCTGCA

CTTTTTGCTGGATTTCGGACAGCAGAATGGAGGGAAAGTGTTGGTGAAGATCAAAGCAGT

AATCTAAATGGTGTGGGATTAGACCAGGCAGATCTTGTGGAAGAATATTTGAACCTGTGT

GAGCAATATCCAGTGCCCTGGAGAATGATCCGTTCTCATGTGCACAAGATGTTGGGGAAT

TGGTTTAGGCTCCATCCAGAAGTGAGAGAGGAGTTGAATCGTCAATCGAAGCTCACCTTT

GAGTTTTTGCATGATATGGTGGGCAGGCTTCGGAATCTTGGGGGCAGTGTTCCGCTTTAT

GCCAGCCCAACTAATACTAGTAAAATTACAGAAAACGGATTTACTGCCTTCAATTGA

>TRINITY_DN101615_c0_g1|m.255 TRINITY_DN101615_c0_g1|g.255 ORF TRINITY_DN101615_c0_g1|g.255 TRINITY_DN101615_c0_g1|m.255 type:5prime_partial len:782 (+) TRINITY_DN101615_c0_g1:3-2348(+)

CCACTCACCACCCCAATTTCCCAAATCAGGAAGGAGAAGAAGAAGAAGAAGATGAAGTTC

CAATTGGAGGACATCACCGTGTACTTCCCGTACGACCACATCTACCCTGAGCAGTACGCG

TACATGCTGGACCTGAAGCGCGCCCTCGACGCCCGCGGCCACTGCCTCCTCGAGATGCCC

ACCGGCACCGGCAAGACCGCCGCCCTCCTCTCCCTCATCACCTCCTACTCCCTCGCCCGC

CCCTCCGACCCCACCAAGCTCCTCTACTGCACCCGCACCGTCCACGAGATGGAGAAAACC

CTAGCCGAGCTCCGCCTCCTCCACCAAACCCTAGCCTCCTCCGCCTCCGCCCCCCACCGC

GCCCTCGCCCTCGGCCTCTCCTCCCGCAAGAACCTCTGCGTCAACCCCGCCGCCCTCTCC

GCCTCCGCCTCCCGCGACTCCGTCGACTCCGCCTGCCGCCGCCTCACCGCCTCCTGGGTC

CGCTCCGCCGCCGCCGAGCGCCCCCCCGGAGAGGTCCCCCTCTGCTCCTTCTTCGAGAAC

TACGCCGCGCTGTCCGACTCCGGCGGGCCGGACTCCGTCATGCTGCCGCCGGGGGTGTAC

ACGCTGCAGGACCTCAGGCGGTACGGGAAGGAGAAGGGGATGTGCCCGTACTTCCTCGCG

AGGCAGATGATCAAGTATGCGAATGTGGTGGTGTACAGCTACCAGTACCTGTTGGATCCC

AAGGTGGCCGGGATCATTTCCAAGGAGATGCAGAGGGAGTGCGTGGTGGTGTTTGACGAG

GCCCATAATATCGATAACGTGTGTATTGAGGCGCTGAGTGTGAGCGTCAGGAAGCAGACG

CTGGATGGGGCCACAAGGAATTTGAATAAGATGGCGCAGGAGATTGATAGGTTCAAGGCC

ACAGATGCAGGCAGGTTGCGTTCTGAATACAACCGCCTCGTCGAGGGCTTAGCACAGAGA

GGAAACTTGCCCCTGACCGATGCTTGGCTTGCGAATCCTGCCCTACCTGATGATATTTTG

AAGGAGGCCGTGCCTGGAAACATCCGAAGAGCAGAGCATTTTCTATCAGTTTTACGAAGA

TTGGTACAGTACTTAAAAGGGCGTCTTCAGACTGAGAATGTCGAGACAGAAGGACCTGTT

GCTTTTGTTGCTTCAGTAAATTCTCAGGCTGGAATCGATCAGAAAATGCTGAAGTTCTGC

TATGACCGACTGCACTCGCTTATGCTTACACTTGAAATAACTGACACAGATGAATTTTTA

CATATTCAAACTGTCTGTGACTTTGCAACGCTAGTTGGGACATACACTCGTGGATTTTCG

ATCATAATCGAACCGTTTGACGAAAGGATGCCTCATATACCAGACCCTGTATTGCAGCTC

AGCTGTCATGATGCCTCTCTTGCCATAAAACCTGTTTTCGACAGATTCCAGTCTGTTATA

ATTACGTCAGGAACTCTAAGCCCAATCGATCTGTATCCTCGACTGTTAAATTTCAATCCT

GTCATCAGTCGGAGTTTTACAATGTCTTTGACAAGGGATTGTATATGTCCCATGGTTCTT

ACTCGTGGAAGTGATCAGCTACCTGTAAGTACCAAATTCGATATGCGGAGTGATCCTGGT

GTTGTAAGGAACTATGGACGGCTCTTGTTGGAAATGGTTGCTGCTGTTCCAGATGGCATT

GTCTGCTTCTTTGTCAGTTACTCGTATATGGATGGCATTGTCAATAGTTGGAACGAATTG

GGCATTTTGCAGGATGTGATGCAGCATAAGTTAGTATTCATTGAAACTCAAGATGTAGTA

GAGACCACCTTGGCACTTGATAATTATCGTCGAGCGTGCGATTGCGGCAGAGGAGCAGTC

TTTTTTTCTGTTGCGAGGGGAAAAGTAGCCGAAGGTATTGATTTTGATAGGCACTATGGG

CGACTTGTTATCATGTTTGGGATTCCTTTCCAGTACACTCTTAGCAGGATTTTGCTCGCC

AGATTAGAGTACTTAAGAGAAACTTTCCAAATAAAAGAGGGGGACTTTCTGACTTTTGAT

GCATTGAGGCAAGCTGCACAATGTGTTGGTAGAGTAATTCGCTCAAAAGCAGACTATGGA

ATGATGATATTTGCGGATAAGAGATATAGCAGACATGACAAACGTTCTAAATTGCCTGGA

TGGATATTGTCACATTTACGTGATGCGCATCTCAACCTGAGCACAGACATGGCATTGCAT

ATAGCACGTGAGTTCCTGCGAAGGATGGCTCAACCATACGATAAGACTGGCGGCGGTAGC

AAGAAGACACTACTATCGCAAGAAGACTTGGAGAAGATGGGGCAGAGCGCTATGGAGATG

TTTTGA

>TRINITY_DN101615_c0_g1|m.257 TRINITY_DN101615_c0_g1|g.257 ORF TRINITY_DN101615_c0_g1|g.257 TRINITY_DN101615_c0_g1|m.257 type:complete len:120 (-) TRINITY_DN101615_c0_g1:2295-2654(-)

ATGCCAGCAACATCTTGCGTTACTCATGTTCTTAACATTAAAAAGAAAAGGAAAAAAAAA

AAAAAAAAAAAAGGAAAAAGGAACAGGAACAATGATACAAAACTTTCTGATATTTCTATC

ATCCAAACATACAGTTGCATAGTATCCATGGAACCCCCACCCGCTCTCTTCATGGTGGTG

TATAAAACAACTGAAGCATTCGCCTCGTCCTCATATTACCATCTCTTGAAACAGTGCCAA

CTTGATAAGAGAAGTACATGCTCTGCCGAACCAGCACTAAAGATAATGGCAAATCACTTG

AAGTTTTCAAAACATCTCCATAGCGCTCTGCCCCATCTTCTCCAAGTCTTCTTGCGATAG

>TRINITY_DN10161_c0_g1|m.259 TRINITY_DN10161_c0_g1|g.259 ORF TRINITY_DN10161_c0_g1|g.259 TRINITY_DN10161_c0_g1|m.259 type:complete len:262 (-) TRINITY_DN10161_c0_g1:307-1092(-)

ATGGCTCCTACTACTGTCGGAAGTAAAACAGCAAATGATGCCAACGGCATTGCGTGTTCT

TATTCTGCTGCAAGTGCCCCGACATCAGGAGAGAGTACTCCAACTGAAGCAGATGACATT

CGAAAAGCGACAACATCTGGACCTCCCTCGCTCACTCCCACTTTAAGGGAGAGTGCATCG

ACTGGGGATAATTTGAGGGTGTCAGGAACGATAGATGGAAAGTTTGAGCATGGCTATTTT

GCTACTGTCAAGATTGGCACTCAGATTCTCCACGGGGTTCTCTATCATGTTTCCCCGGTG

GCCGAGGCTTACTCCACTCTACATCAAGCCAGCCGCCCACCACCACCCGGGCCCACTCCT

GCTACCGTGCTATCTCCGTCTAATGCTGTTACTCCATACATCCCTCAAACTCGATCAGTC

CGTAGGAGGAGGAAATACAAAAAACGTGCCCCAGACCACCCGAAACCAAACAGGAGCTCG

TACAATTTTTTCTTTGCTGAGAAGCACAGGAAGCTCAAGACTGAGTGCCCTAACCAGGAA

AGGGAGTACAGCAAGATGATTGGAGTAGCTTGGAATCAGCTCACTGGAGATGAGCGGAGG

GTATATGATGACTACGGGAAGAGAGACAAGGATAGGTACATAAGAGAGTTGCAGGAATAT

AAGGAGAGGATGAGGCTTGTGCAGCCGAGGGGACTCTTGAAGGTTAACGGCGTCGTGAGA

GGGACTGAAGTTGCAAGGGAAGTCCCAAACTCAAAGGAGGCTGAAATGGCAGAGGCAGCA

TCGTAA

>TRINITY_DN10163_c0_g1|m.262 TRINITY_DN10163_c0_g1|g.262 ORF TRINITY_DN10163_c0_g1|g.262 TRINITY_DN10163_c0_g1|m.262 type:internal len:120 (-) TRINITY_DN10163_c0_g1:2-358(-)

GTCGATGGAGATGAGGCCGGCGCTGCAGCCCATCCCCGAGAGGTTGAAGCTGCGGATGTT

GCTGCGGAGCTTGTACTTGTTGATGATCATCGCCGACAGCGACGGGGTCGGAGAGAAGAG

ACTGCAGTTGACGATCAGGATGTCGATGTCCTTCGCCTTGAGCCCTGTTTTACGGAACAG

ATCGTCGATAGCGGAGAAGATCACCAGCTGGGCCTCCACCCGGGCCGCCTCCATGTTGGG

CCGAGGCGGCAGGTAATGATTAGATGGGGGCAAACAGGTCTCCTCGCCGAGGCCGGACCG

TTCCAGGATCCGCATTTGGAACTCGACGGACTTCTCGTTGAAGAAGGGCAAGAGCTT

>TRINITY_DN10163_c0_g1|m.263 TRINITY_DN10163_c0_g1|g.263 ORF TRINITY_DN10163_c0_g1|g.263 TRINITY_DN10163_c0_g1|m.263 type:internal len:120 (+) TRINITY_DN10163_c0_g1:2-358(+)

AAGCTCTTGCCCTTCTTCAACGAGAAGTCCGTCGAGTTCCAAATGCGGATCCTGGAACGG

TCCGGCCTCGGCGAGGAGACCTGTTTGCCCCCATCTAATCATTACCTGCCGCCTCGGCCC

AACATGGAGGCGGCCCGGGTGGAGGCCCAGCTGGTGATCTTCTCCGCTATCGACGATCTG

TTCCGTAAAACAGGGCTCAAGGCGAAGGACATCGACATCCTGATCGTCAACTGCAGTCTC

TTCTCTCCGACCCCGTCGCTGTCGGCGATGATCATCAACAAGTACAAGCTCCGCAGCAAC

ATCCGCAGCTTCAACCTCTCGGGGATGGGCTGCAGCGCCGGCCTCATCTCCATCGAC

>TRINITY_DN101677_c0_g2|m.266 TRINITY_DN101677_c0_g2|g.266 ORF TRINITY_DN101677_c0_g2|g.266 TRINITY_DN101677_c0_g2|m.266 type:5prime_partial len:123 (-) TRINITY_DN101677_c0_g2:1480-1848(-)

CCGAAACCGCCCAGATCGCAACACCATTGCACCCAACATCATCACTCTTTTTTAATCAAA

CGCCACCTTACAAACACCGCCAAGATGAGCTTCCTCGGAATGGGTCGTCCTCAGCCTACC

TCGGAGCAAAAGATTGCTGCCGTGGAGAGCGAGATGCGCATGATGGCCGACACCTACAAC

CGTCTCCAGAACTCGTGCCAGAAGAAGTGCATCCCCAACGACTACCGCGAGGGCGAGCTC

AACAAGGGCGAGTCCGTCTGCCTCGACCGCTGCACCGCAAAGTTCCTCGACACGTCGATG

AAGGTCAGCGAGATTATGCAGCAGCAGGGCCAGGCCCTCGGCGGCCAGCAGGGCGGCGGC

ATGTTTTAA

>TRINITY_DN101677_c0_g2|m.264 TRINITY_DN101677_c0_g2|g.264 ORF TRINITY_DN101677_c0_g2|g.264 TRINITY_DN101677_c0_g2|m.264 type:5prime_partial len:329 (+) TRINITY_DN101677_c0_g2:1-987(+)

GCCATGCTTTCCACCACAGCACCCATGCCAGGGGTAGGCAGGGCATTCACTTGGAAGAAC

AAACCAGAAGAGTACCTGGCTGCGATGAGGCGGGGTGAGACGGATGTGATTCCAGGTCTG

CCCCAAACTTCGCACTTTGCGTCTACTTCGACTGTCGATTTCAAACCTGCCTCGGCTCCA

GCTTGTGAGACTCAGAAGCGTGAGCAGGAGGGTGAGCTGATGGACATTGGTGCCGAGGTG

CGCAAGGATGTACTCTCCAAAGCGCCCTCGGCTGTTGGATCTCTGGTTGATGAAGCAGAC

ACTCAGGGACCGTTTCTGGAAGCAGAACGCAAGAATAGGCCCGCGCGCGTGGGACTCTCA

CGACGGCATAGCATCCAGCTGCTCCAGTGCGGATCAGAGAGGATCCTCAATGAGAACCGA

TGGCCACGCCGAATGTCGTTTAGCGATGCCGAAGAAGCCGTGCTAGGGTGGGATGATATC

ATTGATATCACGGATACCACGGGCGACTTTGCGAATCTGGAAGCATTCAATGACCTTGCT

CAGTTCTTCAGCCGGCAGATGGATGAGATCAAGGAGGGCATCGAGCCGTGGGTGACAGAG

AAGCTCAAGGCGGTTGAGCTCCTCGACGATCGCTACGCAAAGGACAAGGATGAGCTCCAC

GCTCTCTACTACCAGTTGAATGAGGCATGTCAAAGGATGCGTATCAGTTCTCACGAACTC

CTTGCCGAGGAGCGATCCCACCTGACGGAGGGCTTGAAGGAGGTCGAGGTGCTTGTAGCG

CGTCTCGACTATGAGATCAATGCTCTCCTATCAAAGGTGAATGACGTCGAAGATGGTATT

GTCACATTTGAGAGGCAGGTGGAGGACATGGAGAAGAAAGCCGAGGACCTCAAGATCCAG

CTCGAAACGGAAGGATGGTTACATTGGCTCTTCAGGACGATGACAGGCATTGGAACTGGC

CCGGATATCACACGGTCTACGCCATAA

>TRINITY_DN101677_c0_g2|m.265 TRINITY_DN101677_c0_g2|g.265 ORF TRINITY_DN101677_c0_g2|g.265 TRINITY_DN101677_c0_g2|m.265 type:5prime_partial len:135 (-) TRINITY_DN101677_c0_g2:1445-1849(-)

GCCGAAACCGCCCAGATCGCAACACCATTGCACCCAACATCATCACTCTTTTTTAATCAA

ACGCCACCTTACAAACACCGCCAAGATGAGCTTCCTCGGAATGGGTCGTCCTCAGCCTAC

CTCGGAGCAAAAGATTGCTGCCGTGGAGAGCGAGATGCGCATGATGGCCGACACCTACAA

CCGTCTCCAGAACTCGTGCCAGAAGAAGTGCATCCCCAACGACTACCGCGAGGGCGAGCT

CAACAAGGGCGAGTCCGTCTGCCTCGACCGCTGCACCGCAAAGTTCCTCGACACGTCGAT

GAAGGTCAGCGAGATTATGCAGCAGCAGGGCCAGGCCCTCGGCGGCCAGCAGGGCGGCGG

CATGTTTTAAGAGGCATTAGATATGGGGGGACACAACACGAATAG

>TRINITY_DN10167_c0_g1|m.267 TRINITY_DN10167_c0_g1|g.267 ORF TRINITY_DN10167_c0_g1|g.267 TRINITY_DN10167_c0_g1|m.267 type:complete len:610 (-) TRINITY_DN10167_c0_g1:75-1904(-)

ATGAGCAACGCTGTCAACCTCGACACCGACGTCATTGCCCAGTCCGGGCTCGTCGACCCC

GAGACCAAGGGCATCCCTCAGATCCCCGCAAAGGGATCCATGGACAAGGGCGCCCGTCAC

GACACTCGCTCGACCAGCGATGAGTCGTACGACGACGCTCCCACCGAGCACGACCTGCAG

ACCCTGCGCCGTGTCTCTGGAAAGATCAAGTGGAGCATGTACACCATCGCCTTCGTCGAG

CTGTGCGAACGTTTCTCCTACTACGGTTCTTCCGTCCTCTACACCAACTTCGTCAACAAG

CCTCTCCCCGATGGCTCCAACACTGGTGCTCCCACCGACCCCTCGGCCTCTAACGCCCAG

GCTGGTGCTCTCGGTATGGGACCCCAGGCCGCCCAGGGTATCTCCCTCTTCAACCAGTTC

TTCGCCTATCTGATGCCTCTTGTTGGTGCCTGGATTGCTGATGCCCGCATGGGTCGTTTC

TGGACCATCCACCTTGCCATCGGTATCTCCACCATTGCCCACGTTATCCTCGTTGCTGCC

TCTGCTCCCAATGTCATCATCCACGCCGACTCCTCCTTCGCCGCCTTCATCATCGGTCTC

CTGTGCCTGTGTGTCGGTACCGGTTTCTTCAAGGCCAACGTCTCCCCCTTGCTTGCTGAG

CAGAACGAGGATACCCGCATGCGCGTTGAGACCCTCGCCTCTGGTGAGCGCGTCATCATC

GACCCTGCCGTCACCAACACTCGAGTCTTCCTCTACTTCTACTTCTGTATCAACATGGGC

TCCCTGGCCGGTCAGATCGGCATGGTCTACGTTGAGAAGTACGTTGGTTTCTGGCTCGCC

TTCCTCATCCCCACCGCCATGTTCCTCATCGCCCCCTTCGTCCTCTGGATCAACAAGAAG

AACTACAAGCTCTCTCCCCCCACCGGCTCTGTCCTGTCCAAGTTCCTGCGCATGTTCCGA

TATGTCCAGAAGCGCTCCCAGTTCTTCAAGCCCCAGTGGGAGTTGGCCAAGCCTTCCAAC

GTCCCCATCGACGAGCGCCCCGTCTGGATGACCTACGATGACGCCTGGGTTGATGAAGTC

CGCCGAGGTCTCATGGCCTGCAAGGTCTTCCTCTTCCTCCCCGTCTTCTTCCTCGCCTAC

AACCAGATGACTGGTAACCTGACCACCCAGGCCTCCACCATGGAGCTCCACGGTACCCCC

AACGACGTTATCCAGAACCTCAACCCCATCTCCATTGTCATCATGGTGCCCCTCATCGAT

CACCTCCTGTACCCTGGTCTCCGCAAGATCGGCGTCGCCTTCACCCCCATCAAGCGTATG

ACTTTCGGTTTCGGTATTGCTGCCCTTTCCATGGTTGCTTCCGCTGTTATGCAGCACTAC

ATCTACAAGATGAGCCCCTGCGGTGACCACGCCAACGACACCGACTGCTCCGGTCCCGCC

CCCATCAACGTCTGGGCTCAGTGCCTTCCCTACATCCTCATCGGTCTCGCTGAGATTCTG

ACCAACGTCACCTCCTACGAGTACGCTTACTCCAAGGCTCCCGAGAACATGAAGTCCCTC

GTCATGAGTGTCAACTTGTTCATGAGCGCCATCTCCGCCGCCATCGGCCAGGCCTTCACC

CCTCTCTCCATCGACCCTCTCCTGGTCTGGAACTACACCGTCGTCGCTGTCATCTCTGGT

GTCGGAGGTGTTGCTTTCTGGTTCTGCTTCAAGCACCTCGACTCCGAGGAAGACAAGTGG

AACATGCTTAAGAAGTCCGAGTACCAGGGTACCAACCAGCCCACTGCTCTGGAGAACAAG

CTTGCTCACGAGGGTACCGATGCGGCTTAG

>TRINITY_DN101680_c0_g1|m.270 TRINITY_DN101680_c0_g1|g.270 ORF TRINITY_DN101680_c0_g1|g.270 TRINITY_DN101680_c0_g1|m.270 type:internal len:159 (-) TRINITY_DN101680_c0_g1:1-474(-)

TATGTCGAGCCCAGGATGAATCTTGATGGGTTGATGGAATCGCTGTCGAGGTTCACACAG

TTTACGATTCAACAGGTGCACATGTCCAGCAAGCTATGTTTATCTGAAGATGATGCCGGC

AATAACGGAAGGATGTGGGGCATCGAAAAATTGGTGTACAAAGACATAAAGCGAGGGATC

AGGCGTGATCGAAAGTACATGATCCAGCCTCGCAACGCCTTTTCCGTTGGCGTCCACCTG

TCTCAGAACTTTGCGGGCAAGACCACCCACAAGACCGAGGGCAGGATCATGTACTACCAC

TACCACGGCACCATTGCCAACCGGCAAGAGCCCTGCAGAGAGTTCGTGAATGCAACTGAG

CTGACATACGAAGGCACGCCATACTATCGGGACGAGACTCTCCGGCGACTTGCCGGTTTG

ATCAAGGGGTTCGAGCTGAAGACCATTGGAAGCCGGCTTGTGACAACAAGGCAA

>TRINITY_DN101689_c0_g1|m.272 TRINITY_DN101689_c0_g1|g.272 ORF TRINITY_DN101689_c0_g1|g.272 TRINITY_DN101689_c0_g1|m.272 type:5prime_partial len:351 (+) TRINITY_DN101689_c0_g1:1-1053(+)

AAAAAAATGCGACTCTTCGGTCTGAGTTCCGCTCCTCCGCTTTCTTTTCCCCTTTCTCCA

TCTCGTAAAACAACTACTCTCCAGTGGAAGACGAAGAAGAAGATGGCGGCGGCCGCGGAG

GGTTGCTCGATCGCCGAGATGCCTCCGACTCCGGCGTTGACCGTTGACGGAATTCGAACC

CTAGCTGAGTCCGGTCGCTTCAAGGCATGGTTCCTAGATCAGTTTGGAGTTCTTCATGAT

GGGAAGAAGCCATATCCTGGTGCCATTTCGACATTAGAAAATCTAGCAAATCATGGGGCA

AAGATGGTAATCATAAGCAATTCATCCAGGCGAGCATCCGTAACCTTGGAGAAGCTGAAG

GGCCTCGGGTTTGATTCTTCTCTTTTTCTAGGGGTCATCACAAGTGGAGAATTAACACAT

CAATACCTTCAAAGAAGGGAAGATCCATGGTTCGCCCAACTAGGAAGATCTTGTATTCAC

TTGACATGGAGTGATCGGGGTGCCATATCTCTTGATGGTCTAGGCTTGCAAGTTGTGAGC

AGTGTGGATCATGCAGATTTTATCCTAGCTCATGGTACTGAAGCACTGGGCCTTCCTTCT

GGAGATGCACTCCCCAAGAATCTCGAAGATCTTGAACAAATTTTAGAACTATGTGCCAAA

CGAAAAATCCCTATGGTTGTTGCAAATCCAGATTTTGTTACTGTAGAAGCTAGAGCTCTG

TGTGTTATGCCAGGTACCCTGGCAGCTAAATATGAAAAGCTTGGAGGTGAAGTAAAATGG

ATGGGAAAACCTGATAAGGTTATCTATAAATCAGCAATGGCCATGGCCGGTGTAGATCCT

TGTGATTGTATTACTGTAGGGGACTCTCTCCACCATGACATCAAGGGTGCAAATTTTGCT

GGAATTGACTCAGCATTTGTCACTGGAGGAATTCATGCTACTGAACTTGGAATCAGCACC

TTTGGGGAAGTTGCTGACTTCGCTGCAGTCCAAGCTTTGGCGAGTAAATATGGGGCTCAT

CCATCTTTTGTTTTGCCGGCTTTTACGTGGTAG

>TRINITY_DN10169_c0_g1|m.274 TRINITY_DN10169_c0_g1|g.274 ORF TRINITY_DN10169_c0_g1|g.274 TRINITY_DN10169_c0_g1|m.274 type:complete len:150 (-) TRINITY_DN10169_c0_g1:2-451(-)

ATGCCCTCCTGGTCGGCCCCGCCCACCCCGACCCCGGGGGAGATCTTCACCTTCACGTGG

GCCCCGCCGGCCGAGATCTTCCGTCCGTCGGAGTCCTTGGCGACCACGGTGAAGGTGGAG

GGACCGCCCGCGGTGCCGCCTGCGATGCCGGCGCCGGCGGCGAGGCACTTGGAGGGGTCG

ACGGGGCCCACGGGGCGGGCGGACCCGCGGTCGTCGTCGCTGCTGCTGTCAGAGTCATCA

GCGGGGGCGGAGGCGTTGTTGTTGTTGTCGGCGGACTTGGAGTTCTCTAGGGCTTTGAAG

GTGGCCTCGAAGGCGGCCTTGGCGGCGGCGGTCTTCTCGGCCTCGCTCTTGATCCTCGCT

TCCTCCGCTTGCTTCATCCATATGGGCTTCGCCGCTGCCGCCGCACGATCCGACATCGAT

CCGATCGCTAGGGATTGCAAAACAGAATAA

>TRINITY_DN10169_c0_g1|m.273 TRINITY_DN10169_c0_g1|g.273 ORF TRINITY_DN10169_c0_g1|g.273 TRINITY_DN10169_c0_g1|m.273 type:internal len:170 (+) TRINITY_DN10169_c0_g1:3-509(+)

TATTCTGTTTTGCAATCCCTAGCGATCGGATCGATGTCGGATCGTGCGGCGGCAGCGGCG

AAGCCCATATGGATGAAGCAAGCGGAGGAAGCGAGGATCAAGAGCGAGGCCGAGAAGACC

GCCGCCGCCAAGGCCGCCTTCGAGGCCACCTTCAAAGCCCTAGAGAACTCCAAGTCCGCC

GACAACAACAACAACGCCTCCGCCCCCGCTGATGACTCTGACAGCAGCAGCGACGACGAC

CGCGGGTCCGCCCGCCCCGTGGGCCCCGTCGACCCCTCCAAGTGCCTCGCCGCCGGCGCC

GGCATCGCAGGCGGCACCGCGGGCGGTCCCTCCACCTTCACCGTGGTCGCCAAGGACTCC

GACGGACGGAAGATCTCGGCCGGCGGGGCCCACGTGAAGGTGAAGATCTCCCCCGGGGTC

GGGGTGGGCGGGGCCGACCAGGAGGGCATCGTGAAGGACCAGAACGACGGGACCTACGGC

GTGACCTACGCGGTGCCAAAGAGGGGG

>TRINITY_DN1016_c0_g1|m.277 TRINITY_DN1016_c0_g1|g.277 ORF TRINITY_DN1016_c0_g1|g.277 TRINITY_DN1016_c0_g1|m.277 type:5prime_partial len:606 (+) TRINITY_DN1016_c0_g1:1-1818(+)

CCAAAACTTCACTCACAACAACCCTCAAAGTCGACGACGAGCAAAAAAAAAAAGACGACC

CGGTCTTTTTTACGCCTTCCTCTTCTTTCGACGATATCTCCTCGAGCCGACCACATGAGT

GACAACGCGGGTGCCAAGCCGGGCCCTGAGCCTGGCGACCGAGTCGCCATCGGTATTACC

TTTGGCAACTCCAACAGTTCTATTGCCTTTACTGTTGACGACAAGGCTGAGGTTATTGCC

AACGAGGACGGCGACCGACAGATTCCTACCGTCCTCTCCTACGTTGATGGCGACGAGTAC

TATGGTGGCCAGGCCAAGAACTTCCTCGTCCGCAACCCCGACAACACCATCGCCAACTTC

CGAGACTTCCTTGGTCAAGAGTTCAAGGCCATCGACCCTACCCACTCCCACGCTTCTGCC

CACCCCCAGGATGCTGCCGGCTCTGTCGTTTTCACTGTCAAGGACAAGGACAACGAGGAG

ACTTCCACCGTCTCCATCTCTGAGGCCGCTACCCGATACCTGCGACGCCTCGTCGGCTCT

GCCTCCGACTACCTGGGCAAGAAGGTCACCTCTGCCGTCATCACCGTTCCCACCAACTTC

TCTGAGAAGCAGCGAGAGGCCCTGATCCAGGCCGCCAACAATGCCGGCATCGAGGTTCTC

CAGCTCATCTCCGACCCCGTTGCCGCCGTCCTGGCCTACGATGCTCGCCCCGAGGCTACC

GTCGAGGACAAGATCATTGTCGTTGCCGACCTTGGTGGAACCCGCTCTGATGTTGCCGTT

GTCGCCTCGCGCGGCGGCATGTACACCATCCTGGCCACTGCCCACGACTACGATTACGCC

GGTGTCCACCTGGACCAGGCTCTCATGGACCACTTCGCCAAGGAGTTCCAGAAGAAGCAC

AACGTCGACCCCCGAGAGAACGCCCGCAGTCTCGCCAAGCTCCGACTCGAGTCTGAGGCT

ACCAAGCGAGCCCTGAGCCTGGGCAGCAACGCTCAGTTCAGCGTGGAGAGTCTGGCGGAT

GGCTACGACTTCTCTGTCACCATCAACAGGATACGATACGAGATGGTTGGCCGCAAGGTG

TTTGAGGGCTTCAACCGCCTCATCGAGGGTGTTGTCAAGAAGGCTGGCCTCGACGTCCTC

GATATCGACGAGGTCATCCTGTCTGGTGGCACTTCTCACACTCCCCGTATCGCCAACAAC

CTCCGCGGCATCTTCCCCGAGACCACCAACATCCTGGCCCCCGCTACCTCCGCCACCGCT

ATCAACCCCTCCGAGCTCCAGGCTCGCGGTGCTGCTCTCCAGGCCTCGCTCATCCAGGAG

TACGAGGCTGCCGACATTGAGCAGTCCACTCACCCCGCCGTTACCACCGTCAAGCACATC

TCCAACGCCATTGGTGTCATCACCGTCGGTGCCGATGGTGAGGACGTCTTCACCCCTATC

ATGCCCGCCGAGACTGCTGCCCCTGCTCGACGCGTCGTCCGCATCCCCGCTCCCAAGGAG

GGTGGTGATGTGATCATCAAGGTCGTTGAGGGTGGCACCCACATCAAGGTGACCAAGCCT

GAGCCCAAGACCAAGGCCGAGGAGGATGGCGAGAAGGACTCTGACGACGACTCCGACTTT

GACTCGGATGAGGAGGAGGAGAAGCGCGAGAAGGTGTGGAAGATCGGCAACCTCCTCGCT

GAGGCCGCCCTCAAGGGCGTCAAGGCCGACGGCAAGGTCGAGGTCACGATTAACGTCCTG

GCCGACCTCGGCGTCACCGTGACCATCCGCGAGGTTGGCGGCAAGGGCGGTGTGCGAGGA

AACATCCAGGGCCTGTAA

>TRINITY_DN1016_c0_g1|m.278 TRINITY_DN1016_c0_g1|g.278 ORF TRINITY_DN1016_c0_g1|g.278 TRINITY_DN1016_c0_g1|m.278 type:complete len:414 (-) TRINITY_DN1016_c0_g1:2178-3419(-)

ATGTCCTCTTTAATTGGCAAGGCTCGAAAGCCTAAATTTGAGCTTCATCTCAAGATCTAC

GACCTCAACAATGTCCCTCTCGTCTCGGGCCAGTCCTTCATTAAATGGAACCTCGCCCAC

TCCATGAACGCCGAGCACCGCGGCCGCACCGCAAAGTGCCCCATCGCCAACCACAGGGTC

GACTACAGCTTCGTCACTCTCGTGCCCTCTATCCGCATCTCCATAGACCGTAACAACAAC

CTCGCCGAGTGTCCCATCGAGTTCGAGGTGATCCAGGAGTTTGGCATCTCCGAAAAGATG

ACCCTTGGCGTCGTCCGCCTCAACCTCAGCGAGTATGTCGAGGAGAGTGAAGCTTTTGGC

AAGGATGTCGCGTCGCCTGGTCGCATGCGCAGCGGCAGTATTGGTGTCAGCCCAACTAGG

AGCGCGACAGGGCGTCCCCGCCGCGATTCAGATGTTGTCGAAGATGGCATCGTGAGGAGG

TATCTCATGCAGGACAGCAAGGTCAACAGTACCTTGAAGATCGGCATTCTTATGGTCCAA

GTCGATGGCGAGCGCAGCTACGTTGCTCCTCCTCTCAAGACGGCCCCCGTGTTTGGCGGT

ATCGGCGGCATCATGGGCGAGTCAGTAGAGGACGACGTCGGACCTGTCCCCAGCATCTCA

AAGTCACGAGATACCGCAGAGCTCCATGATCTCTACCGCAGCGTCCTCGCCGCCTCATGG

TGCCGTCAGCCTTCGGAGCTGTCGGCTGAGGAAGTCATCGAGGACATATTCAGCGGCGGT

AATGGCTGGAAGACCAAACCCCAAAACGCCTCACCAGACACAGATGGTGATGATGATTAT

GACGACGATTTGGACCACCGCGATACTCTTCGTCCTCGTGATGCCCGTCGTGTAACTCAC

CTCAACGTACATCATCACGGCACATCTCCCACCACGGCCTCCTCACCAACACACGCTCAG

CCTAATCACGGTCCTGTCGGCTCCTCCCATCGGCGTTCGTCCAGCAACTCGAGTGATAGG

AGCTTCTCGACTGTCACCGTGACGCCCGCCAACCGGCGCAAGGGGGTGCGCATCCATGAG

CTCGACCACTCTCGCAGCCTCGCTAGCATGGCCTCCACCATGTCGCTCAGCAGCGATGTC

CTCCGCGAAGTGGGATTTAAAGGGTCCCGTGAGGTGAGGGAGGATGACGTTCGAAATGAC

CTCGTCGCCTGGAGGATGCCGGGCGACCCGCAAGTCATGTAG

>TRINITY_DN1016_c0_g1|m.284 TRINITY_DN1016_c0_g1|g.284 ORF TRINITY_DN1016_c0_g1|g.284 TRINITY_DN1016_c0_g1|m.284 type:complete len:107 (+) TRINITY_DN1016_c0_g1:2134-2454(+)

ATGTATCCCAGTGCCCATCCATCCAAACTCCCAATACCCGTCGTCTACATGACTTGCGGG

TCGCCCGGCATCCTCCAGGCGACGAGGTCATTTCGAACGTCATCCTCCCTCACCTCACGG

GACCCTTTAAATCCCACTTCGCGGAGGACATCGCTGCTGAGCGACATGGTGGAGGCCATG

CTAGCGAGGCTGCGAGAGTGGTCGAGCTCATGGATGCGCACCCCCTTGCGCCGGTTGGCG

GGCGTCACGGTGACAGTCGAGAAGCTCCTATCACTCGAGTTGCTGGACGAACGCCGATGG

GAGGAGCCGACAGGACCGTGA

>TRINITY_DN10170_c0_g1|m.285 TRINITY_DN10170_c0_g1|g.285 ORF TRINITY_DN10170_c0_g1|g.285 TRINITY_DN10170_c0_g1|m.285 type:5prime_partial len:589 (+) TRINITY_DN10170_c0_g1:1-1767(+)

TTGCAAGCAAACTTGGAACTATTTTACAAATCCTTGTATGCAAATTATTCTCTCTTCCTT

TCAATTATTCAACATGAGATAGACATAAATTTGTCCATGTCCAGCCTATTTATGTCCATC

CCCAAAAGTTTATACTCAAAAACGCAAGAAGAGGAAGCAGAGCTTAAGCATCCAACTTCA

ATGGCTTCCATCATCTCAGATGACCCCAAAGCCCCTCTCCTCCCCCTCCAAGGCCCTCTT

CAGGCCTCCAACAGCCCTCTAAGGTCCCTCCTCACCGCCAAGAACCTCGCCATCGTATCG

GGCCCTCTCTCTTGCTTCCTCATCTTCTTCTTCGTCGAGCTCGACAACCATCCCCTGAGC

CGCAACATGCTCGCAGTTCTCGCTTGGGTCTTCGTATGGTGGCTCGCCGAGGCCGTGCCC

ATGGCAGTCACCTCAATGGCCCCCCTCTTCCTCTTCCCTGCCTTTGGCATCTCCACGGCC

GATGACGTCTCCCGCTGCTACATGAACGACATCATCTCCCTCGTCCTCGGTAGCTTCATA

CTCGCACTGGCCGTCGAGCACTACAACATCCACCGCCGCCTCGCGCTGAATATAACATCT

TTATTCTGTGGGGATCCTCTGAACCCCCCGCTGTTACTGTTAGGAATCTGCGGAACGACT

GCATTCGTGAGCATGTGGATGCACAACACGCCGACCGCGGTGATGATGATACCAGTGGCT

ACCGGAATACTGCAGAGGTTCCCGGACGGGGAGGCCGTGCACCCGGACGTGACTAAGTTC

TGCAAAGGGGTGATCCTTGGGGTCATCTACTCTACGGCCGTGGGAGGGATGAGCACGCTG

ACGGGGACCGGTGTCAATCTGATCTTGGTGGGAATGTGGGCGAGTTACTTCCCTCAGGAG

GAACCTATCAGTTTCAGCTCGTGGTTTCTCTTCGGATTCCCGTTGGCTCTTGTGACTTTC

TTTACTCTCTGGGGGATCCTCTGCCTTCTCTACTGCTCCAAGGGATCGGGGAAGGCGCTC

TCTGCTTATTTGGACAGAGCCCATTTGAAAAGAGAGCTCGATTTGCTAGGGCCAATGCCT

TTTGCTGAGAAGATGGTTTTATTTGTATTCGGGATGCTAATTGTTCTGTGGATGACGAGA

AACATAACAAATGATATCCCTGGATGGGGAGCTCTGTTTGATGATCGTGTGGGAGATGGA

ACTGTTAGTATAATGATGGCCACATTGCTATTCATAATCCCAAACGGAAAGAACCCAGGT

GAGAAACTGATGGACTGGAACAAATGCAAGAAGCTCCACTGGAACATCATCCTCCTACTC

GGCGCAGGCTACGCCATTGCCGATGGTGTCCACAAGAGCGGCCTGGCCGACGTGCTCTCC

CAGGGCCTCGACTTCCTCAAGACCGCCCCGTACCTGGCAATTGCCCCCCTTGTCTGCCTG

GTGAGCAGCACGATGACCGAATTCATCACCTCCAACAACACCACCACCACCCTCGTCATC

CCCCTCCTGATCGAGATGGCGAAGACAATGAATGTGCACCCTCTGCTACTGATGGTCCCG

GGCGCCATCGGGGCGCAGTTTGCCTTCCTGCTGCCGACGAGCACGCCTTCCAACATCGTC

GGGTTCTCAACCGGGCATATCGATATCAAGGACATGATCAAGACTGGACTGCCTCTCAAG

GTTGCAGGCATTGTTGTGCTTTCAATCTTGATGCCCACATTAGGTGCATTTGTGTTTGGG

ACAAGCAATCATGTACACATTGATTAG

>TRINITY_DN10172_c0_g1|m.290 TRINITY_DN10172_c0_g1|g.290 ORF TRINITY_DN10172_c0_g1|g.290 TRINITY_DN10172_c0_g1|m.290 type:complete len:107 (-) TRINITY_DN10172_c0_g1:593-913(-)

ATGATGCCTCCACTGGGGTCGGCTCTGAGCGAATATAGGGTTGCTGATAATACAGGTCTG

TATTGGGCATCGACAGCATTGAAGTATTGGGTTGCCGAGGCTGTGAGTAGCCAAGCCCAG

AGCCTGATGACGACGGGGGAGAATATTGTGGTAATGAGCAGTCGCTGGGATTTGGACTCT

GCTGCGCCGAGTGCTGTGCGTCGCGATGATACGTTGGTACTAGTGTTGGATCATAGGAAA

GAGTCGGCGGTGGCGACGGGGACATCCGGTACAATTGTGGTTGATGAACTGCGAGGGGCT

CGCTTGGCTGCTCGGACTTGA

>TRINITY_DN10172_c0_g1|m.289 TRINITY_DN10172_c0_g1|g.289 ORF TRINITY_DN10172_c0_g1|g.289 TRINITY_DN10172_c0_g1|m.289 type:complete len:390 (+) TRINITY_DN10172_c0_g1:127-1296(+)

ATGTCTACCGATTCCCTGCACGACTCTTCAGTACACGCCCCCCCTCTCCAGTTCAAGGGA

GAGATTATGGACTTTGACCTCGGAATCGAAGTCGACCACCGCAAGAGGCGACGCAACCGG

ACAACTCAGTCCTGTCTCAACTGCCACACTTCAAAACGCAAGTGTGACAGGAAGAGACCG

TGCCAACGTTGTATCCAACTTGGCCTCACTGGTCTCTGCGTATACGAAGTCGACGATCCC

GCTTTAAGGGATGACCCAAACATAGATGAGACTACCCGTCTCCGGAACAGGATCGCAGAG

CTAGAAAGTCTAGTACGGGAACTTCGCGGGAAGCCGCATCCGCGGTGGGCGGAGCCCAAC

TACTGCGAAGGAGATGCCTCCGAGAAGTGGCACTCGCGATCAACAAGAAGGTCGACAATG

CAGTTCAGACAGCGAGCGGCCACACTAAGTGGTGCAACGCCTATGGTCAAGTCCGAGCAG

CCAAGCGAGCCCCTCGCAGTTCATCAACCACAATTGTACCGGATGTCCCCGTCGCCACCG

CCGACTCTTTCCTATGATCCAACACTAGTACCAACGTATCATCGCGACGCACAGCACTCG

GCGCAGCAGAGTCCAAATCCCAGCGACTGCTCATTACCACAATATTCTCCCCCGTCGTCA

TCAGGCTCTGGGCTTGGCTACTCACAGCCTCGGCAACCCAATACTTCAATGCTGTCGATG

CCCAATACAGACCTGTATTATCAGCAACCCTATATTCGCTCAGAGCCGACCCCAGTGGAG

GCATCATGTACCTGCCTGACGAACCCAGCAGCAGGTCACCCTCTGATAGCGCTGAATCAT

CAGCTCCAGAACACTCTCGAGCTTCTTAGGCATCTTCCAGAGCATGCTACAACGCGAAAC

CATTGCCTCATACTGAAGCGCATTAATCAATTACATGATCTCATGCAAGGTGGCGCCGGA

GAGACCCCTCCACCGCCCTTTGAAACTCTTCCGACTCCCGAAAGCGAGCTGATGTCACCT

GTCTCGACATCAAGTCACGCTTCGAGTCTAAATGGTGGCATGCACCAAGATTGGTCAAGC

ATGAGCGCAACGCCCTCGAGCCAATATGACTCGTACTTCCCCGCCGTGGCGGAAAGTAGC

ATGTACCACAAGCCATACCACCTACACTGA

>TRINITY_DN10177_c0_g1|m.291 TRINITY_DN10177_c0_g1|g.291 ORF TRINITY_DN10177_c0_g1|g.291 TRINITY_DN10177_c0_g1|m.291 type:internal len:329 (-) TRINITY_DN10177_c0_g1:1-984(-)

AACTCTCTCCCTCTCTCTCTCTCTACATATAACTCAAACAAAATCACCTGGCGGCGGTTT

GCCGGTTCCGACGCCGATGGTGAGATCTCCCGCCAACAGCACCACCACTTCCCGCAAAAA

ATGAGCGGCCAAGATCGCACCTTTCTGCTCATCCCCCTCCTCCTCGCCTTGATCCCCATC

TCCCTCTCACCTCTCCAACCCAAAGACCCCTCAAACCGAACCCTAACCCTAGCCCCATCC

AGAAACTACATCGTCAGATTCCTCGACTACAAGATTGCCGGCGATCACCGCTCGTACCTC

GAGGAGAGTCTCCGATCGGTCACGAACTGGCGATGGATCGAGAGAAAGAACCCCGCCGTG

GCCTTCCCCACGGATTTTGGGGTTGTGGAGGTTGCGGATTCGAATCGGGCGGATGTGATC

GGTGAGATTGAGAGGTTGGAGCGAGTGAAGGACGTGTATGCTGATTCGAGCTATTCGAGG

ACCTTGTTTGTGGATGATGGTAGTGAGAAAGATGGGAGCTTTTTCGACGAGAAGAAGCGG

CCGGGCAAGATCTTTACGTCCATGTCGTTTGAGGAGGGAGAAGGGATCTATAGTCCGATG

ATTAATACTACGTTCAGCTGGAAAAGAAAGATTTTGATGCAGAGATCTCAGGTTACATCA

CTTTTTGGGGCTGATAGACTTTGGAAGAAAGGATTCACTGGTACAAAAGTTAAAATGGCA

ATCTTTGATACTGGGATTCGAGCTAACCACCCCCATTTTCGGAATATCAAGGAGAGGACT

AACTGGACAAATGAGGATACACTAAATGACAATCTTGGGCACGGGACTTTTGTAGCTGGA

GTTATTGCTGGTGAAGATGAAGAGTGCTTGGGGTTTTCTCCAGACACTGAGATATATGCT

TTTCGAGTATTTACTGATGCTCAGTCTCACTGCAGGTATCCTACACGTCATGGTTTCTGG

ACGCATTTAATTATGCTATTGCAA

>TRINITY_DN10179_c0_g1|m.292 TRINITY_DN10179_c0_g1|g.292 ORF TRINITY_DN10179_c0_g1|g.292 TRINITY_DN10179_c0_g1|m.292 type:complete len:790 (+) TRINITY_DN10179_c0_g1:226-2595(+)

ATGAGCGCCCCACCCAAGAGGAGAACTGCAGGAGAGAATGCGGAGAGTGGAATTGACCCT

GGTCTGACCATCTTGATCAGCAATGGGGAGGATCTGGGCCCCATCGTCAGGCATGCTTTT

GAGGCGGGGAAACCTGAGGCCCTCTTGCATCAGCTGAGAAACATTGTGAAAAGGAAGGAA

ATTGAGATCGAAGAGCTGTGTAAGCTCCACTATGAGGACTTCATTATTGCTGTCGATGAG

CTTCGCGGTGTGTTGGTGGATGCTGAGGAGCTGAAAAGCATGCTGTCAGGGGAGAATTTC

AGGCTGCAAGAAGTGGCTAGTTCTCTTTTGTCGAAGCTTGAGGAACTTCTCGAGTTGTAC

TCTATCAAGAAGAATGTTGCGGAGGCCATACAAACGTTGAAAGTTTGTGTTCAGGTAACG

AGCCTATGCCAGACATGCAACCACCACATCTCAGAGTCCCGGTTTCACCCTGCTCTCAAG

ACGCTGAATCTTATTGAGAAAGATTACCTGCACAATATTCCTGTTAGGGCCCTCAGAAGG

GTGATTGAGAAGCAAATACCAGCAATTAAGTTCCACATTGAGAAAAAAGTCACGAGTGAG

TTCAATGATTGGCTTGTTGCAGTAAGGAGCAAAGCTAGGGAAATTGGACAGATGTCTATA

GGACAAGCTTCCTCAGCTCGCCAAAGAGATGAGGAGATGCGCGCCCGCCAAAGAGAAGCT

GAGGAGCAGAGCCGTTCCAAGGTTGGGGAATGTGTGTACACTTTGGATGTCGAGCAGATT

GATGAAGATTCTGGTTTTGAGTTTGATCTTACACCGGTGTATCGGGCTCATCATATACAC

ACCATCCTTGGTATTGAGGAGCGATTCCGGGATTACTACTACAAGAACCGCCTCATGCAG

TTGAACTTGGACCTACAAATTTCCACGACACAACCCTTTTTGGAATCTCATCAGCCATTC

TTTGCCCAGATAGCTGGATTCTTCATAGTGGAGGACCGGGTTTTAAGAACGGCAGGTGGA

TTGTTATCTGAGAGTCAGGTGGAGACAATATGGGATACAGCTGCTGCTAAGATGACGTCC

ATTCTAGAGAACCAGTTCTCTAGAATGGATACTGCTAGCCATCTCCTCTTGATCAAAGAC

TTTGTTACTCTTCTGGGTGCTACTCTTAGGCGGTGTGGTTACCGAGTGACTCCCTTGCTT

GAGGTTCTAGATAATAGTAGAGATAAATACCATGAACTCCTCCTTGACGAATGCCGAAAG

CAGATGGCAGATATTCTTTCCAATGACACATTTGAGCAGATGGTGATACGGAAGGAGTAC

GAGTACAACATGAATGTCTTGTCCTTTCATCTACAGTCGTCTGACATAGTGCCGGTGTTT

CCATATGTAGCTCCATTCTCTTCTTCGGTTCCTGATGCATACCGGATCATACGCTCATTT

ATCGATGACTCAGTCAGCTATTTGTCGTATGGAGGTCACATAAATTTCTATGATGTTGTA

AAGAAGTATTTGGACAAGCTCTTGATCGAGGTGCTGAATCAGTCTCTTTTAAACTTGATT

CACGGTGGTAGCTTGGATGTTTCGCAAGCAATGCAAATTGCAGCCAACATAGCTGTTTTT

GAGCGTGCTTGCGATCTTTTCCTTTGGCAAGCTGCTCAACGTTGCGGTGTTCCTGTACGC

TTAATTGAAAGGCCTCATGCTGGTTTGACTGCTAAAGCTGTCTTAAAGGCCTCACAGAAT

GCTGCCGACACTGCTCTAGTAAACCTGGTAAGCAATAAGTTGGATGAATTTATGGCTCTC

ATAAACAATATAAATTGGATAGCTGATGAAGCCCCACAAAGTGGAAATGAATATATCAAT

GAAGTTGTTATCTATCTTGAGACCCTACTGTCTTCTGCTCAGCAGATTTTACCTTTGGAT

GCCTTGTATAAGCTTATGATTGGTGCACTGGATCATATTTGTGATTCTATCGGGACAGCT

TTTCTTAGCGACAACGTGAAGAGATTCACCGTCAGTGCTGTTATTGGCATTGACAGTGAC

TTGAAGAAGTTGGAATCTTTTGCTGATGAGAGCTTCCACAGTACAGGCTTGAGTGATTTA

AAGAAAGATAAAAGTTTTAAGGATTGCTTGGTAGAAGCACGGCAGTTGGTCAACCTTCTA

ATGAGCAACCAGCCAGAGAATTTCATGAATCCTGTGATAAGGCAGAGAAATTATGGGGCA

TTGGACTACAAGAAAGTTGCCAGCATTTGTGAGAAATTCAAGGATTCACCGGATAGACTG

TTTGGGAGTCTTTCGAACCGTAATGCAAAGACAAATGCTCGGAAGAAGTCAATGGACATG

TTGAAAAAAAGGCTAAAGGATTTCAATTGA

>TRINITY_DN1017_c0_g1|m.293 TRINITY_DN1017_c0_g1|g.293 ORF TRINITY_DN1017_c0_g1|g.293 TRINITY_DN1017_c0_g1|m.293 type:complete len:773 (-) TRINITY_DN1017_c0_g1:429-2747(-)

ATGTCCGCTCTCCAAGTCTCTCTCCTCTGCCGCCCCTCCCCTTCCCTCTTCTCTCTCCCC

AAACCCTCGCCTCCGATCAGAGCCACCCGCCACCTTTCGTCGGCAGCCCTCCGCTCCCAC

CGTCGCCTCGCCCTCTTCCGCCCCCGCGTGGCACTCTCTCAGGGGGAGAGCTCCGACAAA

GACCCGACCTTGGACCCTCAGGCCCCTCGAAGCCCCGACGTGGATGCCGAGATCTTGAAG

AACGAGGAGGCTGCGGTGGCGGCGGGAAATGGAGGTGGGGAGGAGTCGGCGGTGGCGGTG

GAGAGGAAGGAGAGCAGGTTGCCGCTGCTGGCGTTCTTGATTGGGGTGTGGGAGAGCACG

AGGCGAGGGTTTGATCAGGTGGCGATGTCGGAGTGGTTGAGCTGGTGGCCGTTCTGGCGG

CAGGAGAAGAGGCTGGAGAAGTTGTTTGCGGAGGCCGATGCTCACCCTAAGGATGCGGCC

AAGCAGAGCGCGCTGCTCGCCGAGCTCAACAAGCACAGTCCTGAATCCGTTATAAGACGC

TTCGAAGAAAGAAGCCATGCAGTAGATAGTAAAGGGGTTGCAGAATATCTTCGAGCTCTA

GTTGTCACAAATGCTCTTGCAGAATATCTTCCAGATGAGCGTTTGGGAAAGCCTTCCAGT

CTTCCAACACTGCTGCAAGAGTTGAAGCAACGTGCATCCGGAAGTGATGAAGAGGCATTC

TTAAATCCTGGAATATCTGAGAAACAGCCATTACATGTGGTTATGGTTGATCCCAAAGCA

TCAAATAGATCAACTCGTTTTGCTCAAGAACTTATCTCAACCATCTTGTTTACCATTGTC

GTGGGCCTAATGTGGGTTATGGGTGCATCGGCGCTTCAGAAGTATGTTGGCAGCTTAGGT

GGGATCGGTACATCTGGTGTTGGTTCAAGTGCTACATATGCTCCTAAAGATTTGAACAAA

GAAATCATGCCAGAGAAGAATGTGAAAACCTTCAAAGATGTTAAAGGTTGCGATGATGCA

AAGCAAGAACTTGAGGAAGTAGTGGAGTACCTAAAAAACCCAGGGAAATTCACACGTCTT

GGCGGCAAGTTGCCAAAGGGGATCCTTTTAACTGGTGCACCGGGGACCGGGAAGACATTA

CTTGCAAAGGCTATTGCAGGCGAAGCAGGAGTGCCCTTTTTCTATCGTGCAGGTTCTGAA

TTTGAGGAAATGTTTGTTGGAGTCGGTGCTCGGCGTGTCAGGTCCTTATTTCAGGCTGCC

AAGAAGAAGGCACCTTGCATCATTTTTATTGATGAAATTGATGCTGTGGGCTCAACTAGA

AAACAATGGGAAGGTCATACTAAGAAAACATTACATCAGCTACTTGTGGAAATGGATGGC

TTTGAGCAGAATGAGGGCATAATACTGATGGCTGCAACAAACTTGCCAGATATTCTTGAT

CCAGCATTGACAAGACCTGGTAGATTCGACAGACATATTGTTGTTCCAAGTCCTGATGTG

CGGGGAAGACAAGAGATTCTGGAGCTCTATTTGCAGGACAAACCATTGGCTGATGATGTA

GATGTCAAAGCAATTGCTCGTGGTACTCCGGGCTTTAATGGTGCAGATCTGGCAAACCTT

GTCAATATTGCTGCCATTAAGGCAGCAGTAGATGGTGTTGAAAAATTAAATGCTGCTCAG

TTGGAGTTTGCAAAGGACAGGATAATTATGGGTACAGAGAGAAAAACTATGTTCATATCT

GGAGAATCGAAGAAGCTCACAGCTTACCATGAGAGTGGACATGCTATTGTTGCACTAAAC

ACTGATGGCGCACACCCTATTCATAAAGCAACGATAATGCCGCGGGGGTCTGCTTTAGGG

ATGGTTACACAGCTTCCTTCTCACGACGAAACATCAATAAGCAAGAAACAATTATTAGCA

CGTCTTGATGTTTGTATGGGGGGAAGAGTTGCCGAGGAACTTATCTTCGGTGAAGACTCT

GTCACAACCGGTGCAAGTAATGATCTCCACACTGCTACAGAGCTTGCCCAGTACATGGTG

TCAACTTGTGGCATGAGTGATGCAATAGGTCCTGTGTATGTCAAAGAGCGGCCAGGATCT

GAGATGCAATCACGAATCGATGCTGAAGTGGTAAAACTCCTTAAAGAAGCTTATGAACGA

GTCAAACAGCTACTAAAAAAGCATGAAAGTTCGTTGCATACTCTAGCAAACGCACTGCTT

GAATATGAAACCCTCGGTGCTGATGATATTAAACAAATCCTAAATCCTTACGGAGGAGTC

CAGCTATCTGAACAACAAGAGGAGCTTGCAATGATTTGA

>TRINITY_DN10180_c0_g1|m.295 TRINITY_DN10180_c0_g1|g.295 ORF TRINITY_DN10180_c0_g1|g.295 TRINITY_DN10180_c0_g1|m.295 type:complete len:444 (+) TRINITY_DN10180_c0_g1:85-1416(+)

ATGACCGACTCCTACGTTTCTTCTGCCAACGGGGACTCTATCACTGGCAACGGCATCAAC

AAGATGAACGGCTCAAGTCCCTCCTTACCTGCCCAGTCCGAGCTCGGTGTCTCCTCTATC

ATCCGCAAAAAGCTTATGGGCTACGTCGGTTTTGCCAACCTACCAAACCAGGTCCATAGG

AAGAGTGTGCGGAAAGGCTTCCAGTTCACTGCTATGGTCGTTGGTGAATCCGGCCTGGGT

AAATCCACGCTCATTAACACTCTTTTCAATACTCGTCTATACCCTCCCAAGGTTCCTGTT

CCCCCCAGTCAAGAGCGTCCGCAGACTGTCGCGATTGAAAGTATCAGCGCCGACATCGAA

GAGAATGGTGTCCGTCTCTGCTTAACCGTCGTTGACACGCCAGGGTTTGGTGATTTTGTC

AACAATGACGATAGCTGGAAGCCCATTGTTGAGAACATTGAGTCCCGCTTCGACTCATAT

CTCGAACAAGAAAATCGCGTCAACAGGCAGAAAATCGTGGACAACCGTGTGCATGCATGC

CTCTACTTCATTCAGCCTACTGGTCACTCTTTGAAGCCTATCGACATCGAGTTTATGCGT

CAACTCCATACTAAAGTCAACTTGATTCCGATCATCGCCAAGGCCGACACTCTGACTGAT

GAGGAGGTTGCGGACTTCAAGGCTCGCATTCTGGCCGACATTGCGTATCACAACATCCAC

ATTTTCCAAGCTCCTACCTACGAGAACGAGGATGAGGAGGCTCTCGCTGAGGCCGAGGAA

ATCGCCAGCAAGATTCCCTTTGCAGTCGTTGGCTCAGATACCATTGTTAAGACCCCTGAT

GGCCGGGAAGTCCGCGGTCGTGTCTATCCATGGGGCGTTGTCGAGGTTGACAATGAGGAT

CACTGCGATTTCGTCAAGCTCCGACAAATGCTCGTTAGGACCTATATGGAGGAGCTCCGC

GAGTACACTAATGATGTGTTATACGAGAACTGGCGTACAGAGAAGCTCATTAGCATGGGC

GTAGTTCAAGATCAGAGTGTCTTCAAGGAAATCAACCCTGCGAGCCGGATGCAGGAGGAG

AGGGTCATGCACGAGGCGAAGCTGGCCAAGATGGAGGCTGAGATGAAGATGGTCTTCCAA

CAGAAGGTACAGGAGAAGGAGGCGAAGCTCAAGCAATCAGAAGAGGAGCTCTACGCTCGT

CACAGGGAGATGAAGGAGGCTCTCGAGAAGCAACGCCTGGACCTCGAAGACAAGAAGCGC

CGTATCGAGAGCGGCAGGCCACTCACGCCAGAGAAGACCAGCACAAGAAAGAAGGGTTTC

CTTCGCGCATAA

>TRINITY_DN10182_c0_g1|m.297 TRINITY_DN10182_c0_g1|g.297 ORF TRINITY_DN10182_c0_g1|g.297 TRINITY_DN10182_c0_g1|m.297 type:internal len:196 (-) TRINITY_DN10182_c0_g1:3-587(-)

AAGAGGGCTCCATTTGACCTCTTTGACACAAAGAAGAAGCCAAACAACATCAAGCTCTAT

GTCCGCCGTGTATTCATCATGGACAACTGTGATGAGCTAATTCCCGAGTACTTAAGCTTC

GTCAAGGGTATTGTGGACTCTGAGGACCTTCCCCTTAACATCTCCAGAGAGACGCTGCAA

CAAAACAAGATCCTGAAGGTCATTCGTAAGAACCTTGTGAAGAAGTGTGTTGAGCTTTTC

TTCGAGATTGCTGAGAACAAGGAGGACTACAATAAGTTCTATGAGGCATTCTCCAAAAAC

CTCAAGCTTGGCATCCACGAGGACTCCCAGAACAGGCCCAAGCTGGCTGATCTCCTGAGG

TACCACTCCACCAAGAGTGGTGATGAGTTGACCAGCCTGAAGGACTATGTGACAAGGATG

AAGGAGGGCCAGAGTGACATCTATTACATCACTGGTGAGAGCAAGAAGGCCGTGGAGAAC

TCTCCATTCCTTGAGAAGCTGAAGAAGAAGGGCTATGAGGTCCTCTACATGGTTGATGCC

ATTGATGAGTATGTCATCGGCCAGCTGAAGGAATTTGAGGGGAAG

>TRINITY_DN101859_c0_g1|m.299 TRINITY_DN101859_c0_g1|g.299 ORF TRINITY_DN101859_c0_g1|g.299 TRINITY_DN101859_c0_g1|m.299 type:complete len:123 (+) TRINITY_DN101859_c0_g1:86-454(+)

ATGATGGCATCCTCAGCTTCAATCCTGAGCTCAGCTGGAGCTCGATTCTCCTACAGCCCG

TCGTCGTTCGATGCCGTGCAGAGGCAAAACCCGAGCTCCGCTCAAATGCCATCATCATCA

AGTCGAAGATCATCATCGTTTGCAATGAAAGCTGAAGGAGAAGGAGGCAGCAGGAGCAGC

ATCAACCCCGACATCCGCAAGAGCGAGGCGAAAGTCGTTGATAACGTCCTCGTCTCCGAC

CTCGAGAAGCCCCTCACCGCCTATTGCCGCTGCTGGAGGTCCGGGACGTTCCCGCTGTGC

GATGGGAGCCATGTGAAGCACAACAAGGCCACCGGCGACAACGTGGGACCCCTCCTTCTC

AAGAAATAA

>TRINITY_DN101865_c0_g1|m.301 TRINITY_DN101865_c0_g1|g.301 ORF TRINITY_DN101865_c0_g1|g.301 TRINITY_DN101865_c0_g1|m.301 type:complete len:136 (-) TRINITY_DN101865_c0_g1:167-574(-)

ATGACTTTCTCCCGCTTCGTGTCGATCGGCCGCGTGGCGCTCATCAACTACGGCCCGGAC

GCCGGCAAGATCGCGACCATCATCGACGTCGTGGACGAGAACAAGGCGCTGATCGACGGC

CCGTTCAGCGTCACGGGCGTGAACCGCCAGGTGATCCCGTTCAAGCGCCTCGCGCTGACG

GACCTGACGATCCAGATCCCGCGCCAGCCGCGTGAGGCCACGCTCAAGAAGGCCCTGGCC

AAGGACAACACGGTCGAGAAGTGGGGCAAGACCACCTGGGCCAAGAAGATCGCCAACAAG

CAGACGCGCGCCCAGCTGTCGGACTTTGACCGCTTCAAGGCCATGATCGCCCGCAAGCAG

AAGGCCAGCCTCGTCAAGAAGACCATCGCGGCCGCCCGCAAGAACTAG

>TRINITY_DN10186_c0_g1|m.303 TRINITY_DN10186_c0_g1|g.303 ORF TRINITY_DN10186_c0_g1|g.303 TRINITY_DN10186_c0_g1|m.303 type:complete len:275 (+) TRINITY_DN10186_c0_g1:72-896(+)

ATGTTGGTGAATTGTACTACTGGTAACCTGATGAGGGACTGTGACTCGTGCCGGTCGGCG

CCGGGCTCGGTATACTGCCATGCAGATGCGGCGGTGCTCTGCGCCGTCTGCGACGCTAAC

ATCCACTCGGCCAACCTCATCTCCCACCGCCACCACCGGGTGCCGGTCCTCAGCATCCCT

CAGGAGACTGAGTACAATGATGAGGAGGGCAACCACAACAACTATGCAGCTCTGTTTGGT

GAGGAGGTGGATGAGTACTTGGACCTTGTGGAGTTCAACAACTCATGCTCAGAGGAGAAG

CCTGCAGGCTTCAACTTGAACTACGAGTGTGTGAAGAGTGAGGTGAGTGAAGGTGTTGTG

CCTGTTCAGTCTGTGGTGACCAGTGAGGAGCAGGAACAGGAGAAGCAGCACAAGGATTAT

GATGAGTTTGGATACTCTGCTGCAATCAGACATCAAGCTGTTTCCATGTCATCCATGGAG

GCAAACAGTGTCGTGCCAGACACGGCAGTGGTGACAGACATCCCGAGCCCTCACATCCGA

CCTTCCAAAGGAACGATAGAGCTTTTCTCCGGCCCCCCAATTCATGTCCCGACACAATTC

TCCCCCATGGACAGGGAAGCAAGAGTACTCCGATACAGGGAGAAGAAGAAGACTCGAAAG

TTTGTGAAGACAATAAGGTATGCATCTAGGAAGGCCTACGCCGAAGCCAGGCCAAGAATC

AAAGGGCGGTTTGCTAAGAGATCGGAAGTAGAGATCGAGGTGGATCAGATGTTCTCCAAC

TATGTTGTCACCGATAATGGCTATGGCATTGTCCCCTCATTCTAG

>TRINITY_DN10186_c0_g2|m.304 TRINITY_DN10186_c0_g2|g.304 ORF TRINITY_DN10186_c0_g2|g.304 TRINITY_DN10186_c0_g2|m.304 type:complete len:270 (+) TRINITY_DN10186_c0_g2:724-1533(+)

ATGTTGGTGAACTGTACTAGCAGTAACCTGCTGATGAGGGACAGTGACTCGTGCCGGCCG

GCGCCGGGCTCGGTGTACCGCCATGCGGATGCGGCGGCCGCCTGCGACGCCAACATCTAC

ACGGCCAACCTCGTCTCCCACCGCCACCACCGGGTGCCGATCCTAGGCATCCCTCAGGAG

ACTGAATACAATGATGAGGAGGGGAATGAGAGCAGCAACTATGCAGCTCTGTTTGGTGAG

GTGGTGGATGAGTACCTGGACCTTGTGGAGTTCAACAACTCATGCACTGAGGAGAAGCCT

GCAGGCTTCAACCTGAACCATGAGTGTGTGAAGAGTGAGGTGAGTGAAGGGGTTGTGCCT

GTTCGTCCTGTAGAGACACCCGAGCAGGAGCAGGAGAAGGATTATGATGAGTTTGGATAC

ACTGCTGCAATCAGACAACAAGTTGTTTCCTTGTCATCCTTAGAGGCAAACAGCTTCGTG

CCAGACACGCCAGCGGTGACAGATATCCCGAACCATCACATCCGACCTTCCAAGGGAACG

ATAGAGCTCTTCTCTGGTCCTCCAATTCAAGTCCCAGCCCAATTCTCCCCCATGGACAGG

GAAGCTAGGGTGCTCAGATACAAGGAGAAGAAGAAAACTCGAAAGTTTGAGAAGACGATT

AGGTATGCATCTAGGAAGGCCTACGCTGAAGCCAGGCCGAGAATCAAAGGGCGGTTTGCT

AAGAGATCGGAAGTAGAGCTCGAAGTGGATCAGATGTTTTCCAACTATGTCGTCTCTGAA

GATGGCTATGGCATTGTCCCCTCATTCTAG

>TRINITY_DN10188_c0_g1|m.305 TRINITY_DN10188_c0_g1|g.305 ORF TRINITY_DN10188_c0_g1|g.305 TRINITY_DN10188_c0_g1|m.305 type:complete len:1490 (+) TRINITY_DN10188_c0_g1:117-4586(+)

ATGACGACCAAAAAAGCCTCGTCATCGACGAAGAAAGTACAGCAACTCCCGCACACGCCG

GGCATCGTCGGCAGCATCATCGAGAAGGGCTTCTCCTCCTCCTCCTCCGGCGCCGCCCCC

AAACCTCCTCTCCCCACCGTCCTCCCCTTTCCCGTCGCCCGCCACCGCTCCCAAGGCGGC

CCGCAGTGGGGGTCTTTGGCGATTGTGGAGCCGAGACACGAGGACGATGAAATGGAGGAA

GAAAACGATGCGGATTACGAATCGGCGTCGTCGTTTGCGAACCCTATCGAGAGGAAGAAG

AAGAAGGGTTTGGATTTCGGCAGGTGGAAAGAAGTTGTGAATAGTGACGAGAGGAAACCC

CAGTTGAGGAAAAAGGAAGCAAAAATTGCCGCTACGAAGAAAGTAGTACCTGTACCTGGA

GTTGAGAAGGAGCCTGAAATCGAAGGAGAGAGTAGAAATTTGGCGGCTGCTTGTACTTCT

AGTACTAATGGAGTTGTTCGTAGCGAAAGAGAACAGGATATGTTGGTGTCTGATGGGGAG

CAGCCGACGTTGATTGATGAGATCGAAGCCGAGAATATTGCTCGGTTGAGCGAGATGTCC

ACGGATGAGATTGCAATGGCGCAGGCAGAGATCACGGAGAAGATGGACCCTGAATTGCTT

GAAATGCTGAAGAAGCGAGGGCGGAATAAGTTGGGGAAAAAGAAGGGCACAGGGGAGCAG

ATTGAAGGTAGTAAGAGGTCGAAAACTGGGGCTTCGATTGGGGACTGGACACCGTCGGGT

GAAGTTAGCAATAAGTCATGGAAAGCTTGGAGTGAGAGAGTTGAGAAGGTTAGGGAGTTG

AGGTTTGCATTGGACGGGAGTGTAGTGGATGTTGGTTCCGACCAGTTAAACTGTAATAAG

CTTGATTCCAATCAATATAATGGTGAGACTGTGGTAGAACGCGATTTCTTGCGAACTGAG

GGGGATCCGGCTGCTTTGGGTTACACCATCAAAGAAGCAGTTGCACTTATTCGGAGCATG

GTACCTGGCCAGCGGGTGCTTGCCTTGCAGCTTCTTGTTTCTATCTTTAATAAGGCTATA

TACAACATGCAAGAGAAGGATGGCGGAGATAGTATGAGAAAAATTAATTCTATTGATAAA

CTTATTGATTGGCAGGCTATTTGGGCTTTTGCCCTTGGCCCTGAGCCTCAGATGGCTTTG

TCCCTAAGGATTGCCTTGGACGATAACCATGATTCCGTGGTCTTGGCTTGTGCCAAGGCT

ATACAATGTATATTGAGTTGTGATATAAATGAGAAATTCTTTAACATCACAGAGAAAGTG

CCTGCTCTTCTGAAGTATCTTTGCACTGCTCCTGTATTTCGAAGTAAACCGGAAATTGAT

GGTGGTTATCTTCATGGTGGATTCTGGAAGTACAATACAAAACCTTCAAACATACTCCCT

TTTAATGATGCAACGGTAGACGATGAAAGCGAAGGAGAACGTACTATCCAAGATGATGTT

ATTGTGGCTGGCCAAGATATTGCTGCTGGCTTCATTAGGATGGGAATACTGCCAAGAATT

TGCTACCTTTTGGAGATGGACCCCCTCACGGCATTAGAAGAATGTCTGCTTTCGATACTT

ATAGCTTTAGCAAGACACTCGCCTACAAGTGCTAGTGCAATTATGAGATGCCCAAGGCTT

ATTCAAACTACTGTTAAGATGTGTACCAGGCATGAAACAGCAGAAAGTCCTGTCCAGATA

AAAGCAATTATCCTTCTAAAGGTATTGTCTCAATCTGACAAGCAAACCTGCTTAGATTTG

GTGAAACGTGGAGTTTTCCAGCAGGCTATGTGGCAGTGGTATAGACATGCGTTAACCCTT

GAGCGTTGGGTAGAAACTGGAAGGGACCATTGCAAACTCACTTCCAGCTTGATGGTTGAG

CAGTTACGCCTGTGGAGAGTGTGTATTCGTTATGGTTTTTGTATAGAATACTTCTCAGAT

TTTTTCCCTGCTATGTGTTTGTGGCTGTCTCTTCCTACATTTGAAAAACTAATTGAGAAC

AATGTGCTGGCTGAATTTGCTTCCGTCACAAGGGAAGCATACCTTGTCCTGGAGGCTCTA

GCTCAAAGGCTACCAATTCTTCATTCAGTGGATCAACTAAATAAGCAATCCATGGATACT

TCTGTTGATACAGTAGAAGTTTGGTCTTGGAGTCATGTGATTCCAATGATTGACTTAGCT

ATAAGCTGGCTAGCATTGAAAAATATCCCATTTGTATCTTCTCTTGTGGGTGGCCACAAG

AGAAGTGCAGATGCCCTTGATTCGTCTGCAACCTGCATGATCTGGGTGATCTCAGCAGTG

CTGCACATGCTTGTTAGTGTACTTCACAGAATAGCCCCAGTCAGTATCGGTGGAATGAAT

AATAGCTGTACTCATGTGCCTTGGCTTCCTGAGTTTGTTCCCAAAGTCGGTCTGGAGATT

GTCAGAAATGGATTTCTGAACTTCGTTGGTATAGGGGATGCAAAGCATGTAGAGTTTCCT

ACTGATGGTGCCTCTTTAGCGAATGTTCTTTGTTGTTTGAGAAAACAAAGTGAGGTTGGT

GCCTCATTATCTTCAGTAAGCTGCCTTCACGGATTAGTAAGGCTTGCCTCTTCAATTGAT

CGTTCTGTCCAGGGAGCTAGAAATGTATGCTATACTCAGCCTCCTGAGGTTTATAGTTCA

GGAACAGCAGAGAAGATACTAGAGGAAGGCCTATGTAACTGGGGTAAAAATGATTTGATG

GGGATTCTAACTATTTTCCTGACCGCGGTTTCTGCAGAATGGCACTTAGTGCAATCTATT

GAAGTATTTGGCAGAGGAGGACCTGCTCCTGGAATTGGATTTGGCTGGGGATCTGCTAAT

GGAGGATTTTGGTCCACAAATGTTTTACTAGCACAAATGGATGCACAGTTAATCTTAGAA

TTGTTTGAATTTTTACCAGTGATGCTTGAGGAAGAACTTGCATTTGTTGAAGGTTTGAAC

TCTGAAAGATCTGATGTTGCGACGACCCTTGCATCAGGAAGAGTTATTTCTGTTCTTGCA

GTATGTTTGGTTGCAGGACCAAGAGATAGAGGTAGCTTAGAGAAGGCTTTAGATATACTA

CTCCAGGCTCCTGTTCTCAAGTATCTGAATTTCTGTATATATTTGTTTCTTCATGGTAAT

AAGGGGTGCAAATCACTTAAGTGGCAGTACAAAGAAGGGGACTATAAATTCTTCAGTGAG

GTTTTAAAATCACATTTTAGAGAAAGGTGGATAAGTGTAAAAAAGAAGGCATCTGGTAAA

GAGGATAGAGGCGGTGACAGCCATGAGATATCCAGAAAAAGTAATGTGCTGGAAACAATT

CACGAAGACCAAGAGACATCAGAAGTGCCCGTCAAATATCCAGACTGCAGTAATTGGATA

ACTGAGTGGGCACGCCAGAGACTGCCTCTTCCTGACCATTGGTTTCTTAGTGCAGTTTGT

AGCATTGGTGACATGAAAAGTACTAGTACTTGTTCATCTACCGATCTTTGTGATGTGGCA

AAGAGCGGGCTATTCCTTCTTTTAGGTCTTGAATCAATGTCCTATCTTCTGAGTTCTGAT

CTTCAGCCCTCTCCAATTTCTGGTGTGTCTTTGGTTTGGAAGTTGCATGCCCTTTCTATG

GCATTGCGTGCCAATATGGATGTGCTTCTTGATGAAAAATGTAGTGATATCTTTGAAACT

TTACAAGAATTGTATGGCCAACATCTTGAGAAGTTAAGATGCAGAGACACCAGACAACAG

CCAAACAAGAATGGGGAATATCTAGTGTCTTATTTTAAGTTGCCCGAGGCTCAGGAGAGT

GGTAGCGTAGAGTTATTGAACTTCCAAACACAAGTTCATGGAAGCTACACTACGTTTGTT

GAGGAACTCATAGAGCAGTTTGGAGCTATATCTTATGGTGATATAAGATTTGGCAGGCAG

GTAGCGCTTTATTTGCACCAATCTGTTGAACGACCAGTCAGACTTGCTGCGTGGAATGCA

TTATCTAATGCCCATCTTCTCGAACTCTTGCCACCATTAGAGAAGTGTTTTGCCAAACCT

GAAGGATACCTGATGCCTGTTGAGGAGGATGAAGGGATTTTGGAGGCATACGTAAAATCA

TGGAGTTCGGGAGCTCTTGATAGAGCAGGCACTCGAGAATCAATCGGTTTCACGTTGGCT

CTGCATCACATTTCTTGTTTTATTTTCAAACCTAATGCTTCTGAGAAATCATCACTGCAA

TGTAAGCTGGCAAGATCTCTTCTTCGCAGCTATGGTCAGAAACCACGTCAAGAGGGAATG

CTATTGAACTTTCTTCGATATAAATTACCATCATCACAAGACCCGCTGTATAAAACCGAG

ACTGGAAGAAGGCTCGATCTGTTGAAGGAAGCTTGTGAAGGGAGCTCTTCTCTTCTTGCT

GTAGTGGACAAGCTGAAGCCTGCTGTATGA

>TRINITY_DN1018_c0_g1|m.306 TRINITY_DN1018_c0_g1|g.306 ORF TRINITY_DN1018_c0_g1|g.306 TRINITY_DN1018_c0_g1|m.306 type:complete len:207 (-) TRINITY_DN1018_c0_g1:416-1036(-)

ATGCCTTACACATCTCCCACCATGCCCTACTCTTCCCCCAAATCCTCCCTCTACTCCGCC

CCAATCTTGAGTTCCCCGGCAACCTTGACCAAGAATCTGCGCGCCGCTGCCATCCCGGAC

TCGGTGGCCCGCCACCACGACCACGCGGTGGGGCCCAACCAGTGCTGCTCCGCCGTCGTC

CAGACCATCTCCGCCCCCGTCCCCACCGTCTGGTCCCTCGTCCGCCGCTTTGACAACCCC

CAGGCCTACAAGCACTTCGTCAAGTCCTGCCACGTGGTCGACGGCAACGGCGCCGCCGTC

GGCTCCCTCCGCGAGGTCCGCGTCATCTCCGGCATCCCCGCCGTCACCAGCACCGAGAGG

CTCGAGATCCTGGACGACGAGAGCCACGTCCTCAGCTTCCGCGTCGTCGGCGGCGAGCAC

CGCCTGACCAACTACCGATCCGTCACCACGCTCCACCCCTGCGGGCTCCACGGCACCACC

GTCGTCGAGAGCTACGTCGCCGACGTCCCCGCCGGTAACACGCCCGAGGACACGTGCACG

TTCGTCGACACCATCGTGCGCTGCAACCTCCAGTCGCTGGCGCATACCGTGGAGAAGAAG

CTCCGCGGGAGGGCAAATTAG

>TRINITY_DN10191_c0_g1|m.309 TRINITY_DN10191_c0_g1|g.309 ORF TRINITY_DN10191_c0_g1|g.309 TRINITY_DN10191_c0_g1|m.309 type:complete len:374 (-) TRINITY_DN10191_c0_g1:99-1220(-)

ATGGTAGACAACCTTGACGTTCTCAAGGCGCTTAAGATCCGCCGGGAAAGAGGGAACATC

CTCGCCATTTTCGTCCTTGTAAGCGATCGCAATCTTGATCTTTTCGAAGCCGTCCAGGAT

GTCGAGCTTTGTGAGGTTCAGTGCCGTGTAGTGGTTGATAGCTGTGCTGTACTTGACGAC

AACAAGGTCGAGCCATCCGCATCGGCGTCTCCGGCCGGTGGTAGTTCCCCATTCACGGCC

GACCTCCTGAAGCTTTACGCCGACCTCCTCAGTCAGGTCCTCAGTGGGGAAAGGGCCGGA

TCCGACTCTGGTTGTGTATGCCTTGACAACACCAATGACTTCTTCAATCTTGGTTGGGTT

AAGGGCAAGTCCTGCAATGCAGCCTCCCAAACAGGTAGAGCTGCTGGTCACGTACGGGAA

AGTGCCCCAGTCGAGATCGAGCATGAGCGCGTTCGCTCCTTCGATGAGGATGCTGGTGTT

GCTCTCCTGCGCCGAACTCATGAAAGCCACAGCGTCGACGGCATGCTTGGCAAGCTTGGG

GCGATACTCCTTGAATCGGGCGATCTCCTCCTCGACGTCATACTTGAGAAGGTCACCGTA

CCGCTTGGCGTAGCCAGCGGCCAGCCTTCGGAGCTTGGATTCGAAAAGCTCCTCGTCAAA

AACCTCGGACAGTCGGATACCGCTTCGAGCAGCCTTGCTGCTATAGGAAGGCCCAATGCC

CTTTCCTGTTGTTCCGACCTTGCGCTCTCCAAGTTCGACCTCTTCGAGTCCGTCAACAGC

GGCGTGAAGGTCGAAATCGATGTGTACTCGGTCCGAAACAAGGATACGGTCGTAAACGTT

GGGAAGACCCTTGGCATCCAACTCCTCGAGCTCCTGGAAGAAGGAGGGGACGTGGAAGAC

GACACTGGTGCCGATGAAGTTCATGCATTTGGGGTTGATGAGGCCCGAAGGAAGCAGGTG

GAAGCTATAAGACACGCCATTGGCGACGATCGAGTGTCCAGCATTGTGACCTCCAGCAGC

GCGCGCGCAAAGCTGGGCCTCCGGGGCCAGGATATCGGTAAGTTTTCCTTTGCCTTCATC

GCCCCATTGGGAGCCCAGAATGAGTTTGATACCCATGATTGA

>TRINITY_DN10191_c0_g1|m.308 TRINITY_DN10191_c0_g1|g.308 ORF TRINITY_DN10191_c0_g1|g.308 TRINITY_DN10191_c0_g1|m.308 type:complete len:426 (+) TRINITY_DN10191_c0_g1:105-1382(+)

ATGGGTATCAAACTCATTCTGGGCTCCCAATGGGGCGATGAAGGCAAAGGAAAACTTACC

GATATCCTGGCCCCGGAGGCCCAGCTTTGCGCGCGCGCTGCTGGAGGTCACAATGCTGGA

CACTCGATCGTCGCCAATGGCGTGTCTTATAGCTTCCACCTGCTTCCTTCGGGCCTCATC

AACCCCAAATGCATGAACTTCATCGGCACCAGTGTCGTCTTCCACGTCCCCTCCTTCTTC

CAGGAGCTCGAGGAGTTGGATGCCAAGGGTCTTCCCAACGTTTACGACCGTATCCTTGTT

TCGGACCGAGTACACATCGATTTCGACCTTCACGCCGCTGTTGACGGACTCGAAGAGGTC

GAACTTGGAGAGCGCAAGGTCGGAACAACAGGAAAGGGCATTGGGCCTTCCTATAGCAGC

AAGGCTGCTCGAAGCGGTATCCGACTGTCCGAGGTTTTTGACGAGGAGCTTTTCGAATCC

AAGCTCCGAAGGCTGGCCGCTGGCTACGCCAAGCGGTACGGTGACCTTCTCAAGTATGAC

GTCGAGGAGGAGATCGCCCGATTCAAGGAGTATCGCCCCAAGCTTGCCAAGCATGCCGTC

GACGCTGTGGCTTTCATGAGTTCGGCGCAGGAGAGCAACACCAGCATCCTCATCGAAGGA

GCGAACGCGCTCATGCTCGATCTCGACTGGGGCACTTTCCCGTACGTGACCAGCAGCTCT

ACCTGTTTGGGAGGCTGCATTGCAGGACTTGCCCTTAACCCAACCAAGATTGAAGAAGTC

ATTGGTGTTGTCAAGGCATACACAACCAGAGTCGGATCCGGCCCTTTCCCCACTGAGGAC

CTGACTGAGGAGGTCGGCGTAAAGCTTCAGGAGGTCGGCCGTGAATGGGGAACTACCACC

GGCCGGAGACGCCGATGCGGATGGCTCGACCTTGTTGTCGTCAAGTACAGCACAGCTATC

AACCACTACACGGCACTGAACCTCACAAAGCTCGACATCCTGGACGGCTTCGAAAAGATC

AAGATTGCGATCGCTTACAAGGACGAAAATGGCGAGGATGTTCCCTCTTTCCCGGCGGAT

CTTAAGCGCCTTGAGAACGTCAAGGTTGTCTACCATGAGATGGATGGCTGGATGAAGCCT

ACCACCGACGCCAAGAACTACTACGACTTGCCTAAGCAAGCGCGAGAGTACATTGAATTC

ATTGAGAAGTTTGTCGGTGTCAAGGTCAAGTGGATTGGAACCGGTCCCAGCCGTGACTCG

ATGATCAAGCGGTCTTAG

>TRINITY_DN10196_c0_g1|m.312 TRINITY_DN10196_c0_g1|g.312 ORF TRINITY_DN10196_c0_g1|g.312 TRINITY_DN10196_c0_g1|m.312 type:5prime_partial len:349 (+) TRINITY_DN10196_c0_g1:2-1048(+)

TCGACATCAACAAAGAAAATGGCTTCAGCATGGCACTCCATTGTGTTACTGTTCCTGATT

GTTGCTGCAGGGTTATCTGATGCTGCAGATGTTTACAACATTCTCGAACTTGACATGGAG

ACAAGGCATGAGCAGTGGATGGCCGAGTACGGGCGGGTATATAAAGACGAGGCTGAGAAA

GCACGAAGATTTGAGATCTTCGAGAAGAACGTCAGGTATATCGAAGATTTCAACAATGCC

GGAGAGTACAATTACACCCTAGGCGTCAACGAGTTTGCTGACCTCACCACTGAAGAGTTC

ATGTCTACTCACACCGGTGCAGCGGACGCCGGCCCGCCGGAAGAGTTTGAGTTACCGGAG

TCTTTCGAGCTTGAGACCGAAGACCCTCGGCTGGTCCCTTCTAGCTTTGATTGGAGGGAC

GAGGGTGCGGTTACGGAGGTCAAGAATCAAGGGACTTGCTCGTCGTGCTGGGCATTTGCA

TCAGTTTCAACAGTGGAAAGCCTGACCAAAATAAAGAAAGGTGTACTCTACTCTTTATCC

GAACAACAGGTTATGGATTGCGAGAGAATGAACATAGGATGCGAACCCGGTTATTCGGAC

GATGCCTACAAGTACATGAGGGGAGGAATCACAACTGAAGCTAACTATCCATACAAAGGA

GTACGAAACAATTGCGACTCGAATAAAGCAAAAAACACAGTGACAAAAATTACAGGTTTC

ATATTAGTACGACCGTATGATAGCGAGTCTGCACTCAAGGCCGCGGTGGCACGGCAGCCG

GTTTCGGTAGTTGTTCAAATTACTGGGAACTGGCAATTCTTTAGATCCGGTCTCTACAGC

GGACCCTGTGGCAGTGGTCCTACGTTCGGGCATGCCGTTGCTGCAGTCGGTTACGGAGAG

ATGGGCACAGACAAGTACTGGATTCTGAAAAACTCTTGGGGAAATGGGTGGTCTGAAAAT

GGATATATTAAGCTGAAGAGAGATGTCGGTATAAAAGGTGGATTATGCGGAATTGCGACA

TATGCAAGTTACCCGTACATGCTGTGA

>TRINITY_DN10196_c0_g2|m.313 TRINITY_DN10196_c0_g2|g.313 ORF TRINITY_DN10196_c0_g2|g.313 TRINITY_DN10196_c0_g2|m.313 type:5prime_partial len:175 (+) TRINITY_DN10196_c0_g2:1-525(+)

GTACTCTACTCTTTATCCGAACAGCAGGTTGTGGATTGCGACATAGGGAACGGAGGATGC

AAACCCGGCTATAGGGACAGAGCCTACATGTACATGACGAAGGGAATCACAACTGAAGCT

AACTATCCATACAAAGCAGTTCAAAACAATTGCGACGCTACTAAAGCGCAAAACAATGTC

ACACAAATTACAGGTTACCTCAACGTACGACCGTTTAATAGCGAGTCTGCACTCAAGGAT

GCGGTGGCGCAGCAGCCGGTTTCGGTAGGTCTTCAAATTACTGGAAACTGGCAGTTCTTT

AGATCTGGTCTCTTCAGCGGACCCTGTGGAAGTGGTCCTAGGTTCGGGCATGCCGTTGTT

GCAGTTGGTTACGGAGAGAATGGCTCGGACAAATACTGGATAATCAAAAACTCTTGGGGA

AAAAAGTGGGCTGAAAATGGATATATTAAGCTGGAGAGGGATGTCGCTACAGCAGGAGGA

TTATGTGGAATTGCGAGAACTCCACGTTACCCGACCATGGTGTGA

>TRINITY_DN101973_c0_g1|m.316 TRINITY_DN101973_c0_g1|g.316 ORF TRINITY_DN101973_c0_g1|g.316 TRINITY_DN101973_c0_g1|m.316 type:internal len:101 (+) TRINITY_DN101973_c0_g1:2-301(+)

CGAGTACCGCCGCTCGACCACGAAGTCCGGCTCGCTGGCAGTGGACGACGACCCCGAGAC

GGACGCGCGCTGCGACGCGAGCCGCGGGTTGTTGACGTTGGTCGGCAGTCGCGAGTTGTA

GTGGAACAGGTACACGTTGACGATGTAGTAGGTGACGCCCTCGCGCACGATCGCGTCGCG

GATCTCGACCCGCTCGATGTTGGACAGGAACGAGAGCGCGCACGTGGCCGTCGACCAGCG

CAGCGGCTCCAGCAGCACCGGCGAGAGCGGCGGCGCGCGCGGCTTGCCTTCTTCTCCCTG

>TRINITY_DN10197_c0_g2|m.320 TRINITY_DN10197_c0_g2|g.320 ORF TRINITY_DN10197_c0_g2|g.320 TRINITY_DN10197_c0_g2|m.320 type:complete len:197 (+) TRINITY_DN10197_c0_g2:138-728(+)

ATGTTGTTCTCCAAGCAAGTTGTCGTGCTCGCTTCGCTCCTTATTGGCACTCTTGCTAGT

GCCGCTGCCATTCCTATGCCAATGGCGAACGAGATTGCCAAGATGAAGCTGAGGGACGTC

AAGATGATCTCCGCGTACCAAAAACGCCAGGACATTGTTCAGATTAGCGATAGCGCGCCA

GTCAAAGCACCTGAGGGCGTCGTGCAAAGCTATCCTCGCCAGGTGGGAGATGTTGTTAGC

ATTGGAGATAGTACCCCAGTCAAGGCCCCGACCGGCGTTGTGGAGAACTATCCCCGACAG

GCCGGTGATGTTGTCACTATTGGGGATAGCAACCCAGTCAAGGCCCCAGCGGGTGTGGTA

GAGAACTATCCTCGACAGGCAGGTGATGTTGTTACCATTGGAGATAGCAACCCGGTCAAG

GCTCCGGCCGGCGTAGTAGAGAATTACCCTCGCCAAGCAGGCGATGTTGTTACCATTGGC

GACAGCACACCAGTGAAAGCTCCTGCCGGAGTCGTTGAGAGTTACCCCCGCCGGGTCCGC

AAGGAACTTCCTCGTATGAGGCGCACCCGTAGCTCCAAGCGACATGCATAG

>TRINITY_DN10198_c0_g1|m.321 TRINITY_DN10198_c0_g1|g.321 ORF TRINITY_DN10198_c0_g1|g.321 TRINITY_DN10198_c0_g1|m.321 type:5prime_partial len:320 (-) TRINITY_DN10198_c0_g1:390-1349(-)

AAATATGATGAGGAATTTATATCGAGTCCTCGAGGAATCAAGCAATTTACATGCAGATGG

CTTCCATCTAATCAAGAACCAAAGGCCATAATCTTCTTATGTCACGGGTATGCAATGGAA

TGCAGCATATCTATGAGAGATACTGCGATTCGGCTAGCTCTAGCAGGGTATGCAGTCTAC

GGAATCGACTACGAAGGCCATGGAAAATCATCTGGTCTGCTTGGATATGTTCCGAGCTTT

GATGGTCTTGTTGATGACTGCAATCACTATTTTACAAGCATATGTGAGAAGCAAGAGAAC

AAGATAAAGAAGAGATTTCTTTTGGGGGAGTCCATGGGAGGAGCTGTTGCTCTTCTTCTT

CATAGGAAGCAACCAACCTATTGGAGTGGGGCTGTGTTGGTTGCCCCCATGTGCAAGATT

GCTGAGGAGTTGAAACCTCATCCCATAGTTACCAGCCTCTTGCAGAAAATGTGTCATTTC

ATACCAACATGGAAAATAACCCCTACTCAAGATATCATTGATGTTGCATTCAAAAGCCCT

GAAAAAAGAAAAGAGATTAGGAATAATCCTTATTGTTACAAGGGGAGGCCAAGGTTGAAG

ACTGCCTATGAGCTTCTCATGGTGAGCTTAGACATTGAAAAGAATCTTCACCAGGTGTCG

ATGCCATTTATAATTGTGCACGGTGGAGATGACATTGTGACCGATCCCTCAGTCAGCAAA

CTGCTGTACGAATCGGCATCCAGCACGGACAAGACGTTCAAGTTGTATCCTGGGATGTGG

CACGCGCTCACCTCTGCAGAGCCCCTCGAGAGCATCGACCATGTATTCTCAGACATATTT

GCTTGGTTAGAAGAAAGAATCGTCAGTCCGGAGGCAAAGCTGGAGATAGAACGGAAGGCC

AAACACGAAATGGACACAAGTGTGATACATGACAAATCAGCAGGGCTGGTTCTAGGGTAA

>TRINITY_DN1019_c0_g1|m.322 TRINITY_DN1019_c0_g1|g.322 ORF TRINITY_DN1019_c0_g1|g.322 TRINITY_DN1019_c0_g1|m.322 type:complete len:536 (+) TRINITY_DN1019_c0_g1:352-1959(+)

ATGGGGGACGTCGGCGGCGCAACTCCACCACCATCATCGAACGGTGGTGACCAGATCCAC

CACCCGAAAAGAGAAAGAGAAAGAGAAGAGGAGCCAAAAAGCCCCAGCCGGCGGTTACCG

GACTTCTTGCAGAGCGTGAATCTGAAGCACGTCAAGCTCGGGTACCACTACCTCATCACC

CATCTCCTCACCCTCCTCCTCATCCCTTTAATGGTGGTGATCGTAATCGATGCTGGCCGA

ACCGATCCCGATGACGTCAGGCAGCTGTGGGTCCACCTCCAGTACAACCTCGTCAGCGTC

ATAATCTGCTCCGCCGTCCTCGTCTTCGCCGTGACTCTCTACGTCATGACCCGCCCCCGC

CCCGTCTACCTCGTCGACTTCGCCTGCTACCGCCCCCCGGACCGCCTCCAGGTCCGCTTC

CGCCAGTTCATCGACCACTCCCGACTCTGCGGCGAGTTCAACGACTCCGCCCTCGACTTC

CAGCGCAAGATCCTCGAGCGCTCCGGCCTCGGCGAGGATACCTACGTCCCCGACGCCATG

CACTTCTTGCCCCCTCGGCCATCTATGCAGGCGGCGAGGGAAGAGGCCGAGCAGGTCATG

TTCGGCTCCCTCGACATGCTCTTCAACAACACCTCCGTCAAGCCCAAGGACGTCGGGATC

CTCGTCGTCAATTGCAGCCTCTTCAATCCCACCCCCTCGCTCTCGGCCATGATTGTCAAT

CGTTACAAGCTGAGGGGGAACATCAGGAGCTTCAATTTGGGAGGGATGGGATGCAGCGCC

GGAGTGATCGCAGTCGATCTCGCCAGGGATCTGCTTCAGGTGCATAGGGCCACGTACGCC

GTGGTGGTCAGCACTGAGAACATTACCCAGAATTGGTACTTTGGGAATAAGAAATCGATG

CTGATACCCAACTGTCTGTTCCGTGTCGGTGGTTCTGCCGTGCTGCTGTCCAATAAGTCG

GCGGACCGCCGCAGGGCCAAGTATCGATTGGTCCATGTGGTTCGCACTCATCGCGGGGCT

GACGAGAGGTCATTCCGGTGCGTTTATCAGGAGCAGGACGATAACGGAAAGGTCGGGGTG

TCCCTGTCCAAGGACCTGATGGCGATTGCCGGCGGGTCGCTGAAGACTAACATCACTACT

CTCGGCCCACTCGTGCTTCCCGTGAGCGAGCAGCTCCTCTTCTTTGCAACGCTTGTGGCC

AAGAAGCTCTTCAATGCCAAGGTCAAGCCTTACATCCCTGACTTCAAGCTAGCGTTTGAC

CACTTCTGCATCCATGCTGGAGGGAGGGCCGTGATCGATGAGCTGGAAAAGAACCTCCAC

CTGCTGCCTGTACATGTGGAGGCGTCTCGGATGACCCTCCACCGGTTTGGTAACACCTCG

TCGAGCTCCATCTGGTACGAGCTGGCGTATATCGAAGCTAAGGGGAGGATGAGGAAGGGG

CATCGGATCTGGCAGATTGCGTTTGGGAGCGGGTTCAAATGCAACAGTGCCGTGTGGGAA

GCGCTTCGAAATGTGAAGCCGTCGCCTGACGGACCGTGGGAGGATTGCATCGATAGGTAC

CCGGTCGAAATTGTTGATGGCATTCCTGCATCGCAGCCGCAGCAATAA

>TRINITY_DN1019_c0_g2|m.325 TRINITY_DN1019_c0_g2|g.325 ORF TRINITY_DN1019_c0_g2|g.325 TRINITY_DN1019_c0_g2|m.325 type:5prime_partial len:552 (+) TRINITY_DN1019_c0_g2:2-1657(+)

CCCTCCTCACTCCCTCCTACTTCTTCTTACTCCTCCCCCATTTATATCCCATCTTCATCC

CTCTCCCTCCCCTCATCCTCCTCCCAAAAACCATTCACTAGCGTCGTCTCGTTCGTCATG

TCGTCGGAATCCGAAGCCACCGCCGGCAGCGGTCCACCTCGAACCCTCCTCCCGAACCTC

CTCCTCTCCGTCCGCCTCAAGTACGTCAAGCTCGGATACCACTACCTTATCTCCAACGCC

CTCTATCTCCTCCTCGTGCCGCTCCTTGCCGCGGTCACGTTGCAGCTCACTCGTCTCACC

GCCGACGACCTCTACTATTTGTGGGACCACCTCCGCCTCAACCTTGTCACAGTCGTCCTC

AGCTCCACCGTCGTCGTCTTTTTAACGACCTTCTACTTCATGACGCGGCCGCGGCCAGTG

TACCTCCTCGACTTCTCGTGCTACAAGCCCGAGGACGCCCGTAAATGCCCGAAAGAGCTC

TTCGTGGAGCGGTCCGTGCAGGCGGGCACGTTCACTGAGGAAAACCTGCTGTTCCAGAGG

AAAATTCTAGAGCGGTCCGGGCTCGGGCAGTCCACGTACTTCCCCGAGGCCGTGCTCAGG

GTGCCGCCCAACCCTTGCATGGCCGAGGCGAGGAAGGAGGCGGAGATGGTGATGTTCGGG

GCCATCGACGATCTGCTGGCGAAGACAGGAGTGAAGCCGAAGGATATCGGCATCCTGATC

GCCAACTGCAGCTTGTTCAACCCAACGCCGTCGCTGTCCGCGATGGTAGTGAACCATTAC

AAGCTGAGAGGGAACATCATCAGCTACAATCTCGGAGGCATGGGATGCAGTGCGGGATTG

ATATCCATTGATCTTGCCAAGCAACTGCTTCAGGTTCATCGCAACTCGTATGCCTTGGTG

GTGAGCATGGAGAACATCACCCTCAACTGGTACGTCGGCAACAACCGCTCAATGCTGGTC

TCCAACTGCCTCTTCCGCATGGGCGGCGCCGCCATCCTCCTCACCAGCCGCCGCTCCGAG

CGCCGCCGCTCCAAATACCAGCTCATGCACACCGTCCGCACGCACAAGGGCGCGGACGAC

CGCTCATTCCGCTGCGTGTTCCAAGAGGAGGACCAAGCCGGCATGGTGGGCGTCTCCCTC

TCCAAGGACCTCATGGCCGTGGCCGGCGAGGCCCTCAAGTCCAACATCACCACCCTCGGC

CCCCTCGTCCTCCCGATGTCCGAACAGTTGCTCTTTTTCCTCACGCTGGTTGGCCGAAAG

ATCTTCAAAATGAAGATCAAGCCGTACATCCCCGACTTCAAGCTGGCGTTCGAGCACTTC

TGCATCCACGCCGGGGGCCGGGCAGTGCTGGACGAGCTCGAGAAGAACCTTGAGCTCAGC

GATTGGCACATGGAGCCGTCGAGGATGACTCTGTATCGGTTCGGGAACACGTCGAGCAGC

TCGCTGTGGTACGAGCTGGCGTACACGGAGGCGAAGGGGCGGATGAGGAAGGGGGACCGG

GCGTGGCAGATTGCGTTCGGGTCCGGGTTCAAGTGCAACAGCGCGGTGTGGCGGGCGCTG

AAGACCATCGTTCCGGCCAAGGAGAAGAATCCGTGGACGGATGAGATCCACGAGTTCCCC

GTTCATGTGCCGAGAGTGGCTACCATTGCTGCGTGA

>TRINITY_DN1019_c0_g2|m.328 TRINITY_DN1019_c0_g2|g.328 ORF TRINITY_DN1019_c0_g2|g.328 TRINITY_DN1019_c0_g2|m.328 type:5prime_partial len:128 (+) TRINITY_DN1019_c0_g2:3-386(+)

CCTCCTCACTCCCTCCTACTTCTTCTTACTCCTCCCCCATTTATATCCCATCTTCATCCC

TCTCCCTCCCCTCATCCTCCTCCCAAAAACCATTCACTAGCGTCGTCTCGTTCGTCATGT

CGTCGGAATCCGAAGCCACCGCCGGCAGCGGTCCACCTCGAACCCTCCTCCCGAACCTCC

TCCTCTCCGTCCGCCTCAAGTACGTCAAGCTCGGATACCACTACCTTATCTCCAACGCCC

TCTATCTCCTCCTCGTGCCGCTCCTTGCCGCGGTCACGTTGCAGCTCACTCGTCTCACCG

CCGACGACCTCTACTATTTGTGGGACCACCTCCGCCTCAACCTTGTCACAGTCGTCCTCA

GCTCCACCGTCGTCGTCTTTTTAA

>TRINITY_DN1019_c0_g3|m.330 TRINITY_DN1019_c0_g3|g.330 ORF TRINITY_DN1019_c0_g3|g.330 TRINITY_DN1019_c0_g3|m.330 type:internal len:528 (+) TRINITY_DN1019_c0_g3:2-1582(+)

AAAAAAAAAAAAAAAAAAAAAATCTCCCCTTCCTCCTTCAACATCAGATTGAAGAAGAGG

AGAGGGGCTAAAAAAAAAAAAAAAAAAAATCTTTCCTCGGCCGCCGGATTCAATCATTCT

ATCTTTCAGATTTGGATCTACGGGGGAAAAAAAATGGCGGAGGAGCGGAGACCGGCGACA

TCTAACAATCTGCCCGATTTCAAGCAATCGGTGAAGCTCAAGTACGTGAAGCTAGGGTAC

CATCACGTGATCACCCACGCAATGTACCTCCTCCTCCTCCCTCTCCTCGCCGTCATCGCC

GCCCAGCTCTCCACCTTCTCCGGCCAGGACCTCCTCGACCTGTGGGACCACCTCCGCTTC

AACCTCATCTCCGCCGTCGTCTGCTCCGCCCTCCTCGTCTTCCTCTCCACCGTCTACTTC

CTCACCCGCCCCCGCCCCGTCTACCTCGTCGACTTCTCCTGCTACAAGCCCCACGACGCA

CGCAAGTGCACGCGGCCAATCTTCATGGAGAAGTCCACTCTCACGGGTTCCTTCACTCAA

GAAAATTTAGATTTCCAGCGGAAGATCCTCGAACGGTCCGGTCTCGGCGAGGACACTTAC

CTCCCCGAGGCCGTCACCAACGTCCCGCCGAATCCGTCCATGGCGGAGGCCAGAAAAGAG

GCTGAGATTGTCATGTTCGGGGCTTTGGATGAGCTCTTCGCCAAGACTAAGCTCAAGCCG

AAGGACGTTGGGATCTTGGTTGTGAACTGTAGCCTGTTCAATCCGACGCCGTCGCTGTCC

GCGATGATTGTGAATCATTACAAGCTGAGGGGGAATATTGTGAGTTATAACCTTGGAGGA

ATGGGATGCAGCGCTGGCTTGATCTCTATTGATCTCGCCAAGAATTTGCTGCAGGTTTAT

CCAAATTCGTATGCGTTGGTTGTTAGCATGGAGAACATTACTCTGAATTGGTACTTTGGG

AATAACCGGTCTATGCTCGTGTCGAACTGCTTGTTCCGGATGGGCGGAGCGGCGATCCTC

CTGTCGAACAAGAGGTCCGACGGGCGGCGGTCCAAGTATCAGTTGGTTCACACGGTTCGG

ACGCACAAGGGTGCGGACGACAAGTGCTTCAGCTGCGTGACTCAGGAAGAGGACGCCGAT

GGTAAGATCGGCGTTGCTCTATCGAAAGATCTCATGGGGGTCGCAGGTGATGCTTTGAAG

ACTAATATCACGACACTGGGGCCTCTTGTGTTGCCAATGTCCGAACAGTTGCTCTTCTTC

GCTACGTTGGTAGGGAGGAAGCTGTTCAAGATGAAGATTAAGCCTTACATTCCCGATTTC

AAGCTGGCGTTCGAGCATTTCTGCATTCATGCTGGCGGGAGGGCCGTGCTGGACGAGCTG

GAGAAGAACTTGAAGCTTTCGGACTGGCATATGGAGCCGTCGAGGATGACTCTGTACAGG

TTTGGGAACACGTCGAGCAGCTCGTTGTGGTATGAATTGGCGTACATGGAGGCGAAAGGG

AGGGTGAAGAAGAGGGACAGGATATGGCAGATTGCGTTCGGGTCAGGGTTCAAGTGCAAC

AGCGCGGTGTGGCGGGCGCTG

>TRINITY_DN101_c0_g1|m.331 TRINITY_DN101_c0_g1|g.331 ORF TRINITY_DN101_c0_g1|g.331 TRINITY_DN101_c0_g1|m.331 type:complete len:383 (-) TRINITY_DN101_c0_g1:359-1507(-)

ATGGCTACGGTGAATCCGTTCGATCTTCTCGGGGATGACGATAACGACGATCCGACGCAG

TTGCTTGCTGCTCAGGAGAAGAAGGTCGTTGCCAAGAAGCCTGCTCCGGCAGCGGCCGGC

GGCGCTCCGGCCGCGGCTGCGAAGATGCCGTCGAAGCCCCTGCCGCCGTCTCAAGCTGTG

AGGGAGGCAAAGAGCAACACTCCAGCACCGCGCGGAGGTGGTTCAGGTCGAGGTGGGCCA

GGACGTGGAAGGGGTGGCAGCAGTGATGGCCGTGATTTTGGGAATGGAAATGCCAATGGG

TATGCAGGAGTCTATGGTGGGGTGTGTGGAGGTGAAGAAGGAGATAACCATAGGCCGTTG

GACAAGGAGCGTAGGCCCTATGATGGACCATCGGACAGGGAGCGTGGGCCGTACAATGGA

CCCCGCCAATCTTTTCGAGGTGGACGTCGGGATGGGTATGGGAATGAGGAGGCTGGTGGT

GATTCGGATCGGCCTCGGAGGGCCTATGAGCGTCGGAGTGGAACCGGCCGTGGATATGAG

GTCAAACGAGATGGAGCAGGGCGTGGGAACTGGGGAACTGCAGCTGATGATGCTCTCACA

CAGGAAACTGAGGATAATGTGAATGCTGACGAGAAGATTGCAAGTCCAAAGAAACAGCCA

GAGCAGGAAGATGCACCATCAACAGAGGTGGGTAAGGAAGGACAAGCTAATGGCGCTGAA

GTCAAGGAAGAGGATAAGGAGATGACTTTGGAGGAGTATGAGAAAATAAGGGAAGAGAAG

CGGAAAGCCTTGCTTGCAATGAAGGCTGAAGAAAGAAAAGTTGAGCTAGACAATGAACTA

AAGGCTATGCAGCTGCTCTCCATGAAGAAGGAAAATGATCCTATCTTTATCAAGTTGGGT

TCTGACAAGGAGAAAAAGAAAGAAAACGCCGATAGGGATGAAAAGGCCAAGAAGGCTCTG

AGCATCAATGAGTTTTTAAAGCCAGCTGAAGGTGAAAGGTATTACAGCCCCAGTGGCCGT

GGCCGTGGCAGGGGCCGAGGAGGAGATCGTGGGTCGTTCCGCGGTGGTTATGGTGGGGGC

GCTAATACCCTTGCATATGCACCGTCAATTGAAGACCCCGGTCAGTTTCCCACTCTTGGT

GGGAAATAG

>TRINITY_DN10208_c0_g1|m.335 TRINITY_DN10208_c0_g1|g.335 ORF TRINITY_DN10208_c0_g1|g.335 TRINITY_DN10208_c0_g1|m.335 type:5prime_partial len:352 (+) TRINITY_DN10208_c0_g1:1-1056(+)

ACAGTGCGTATTATAGTCAGAAAAATGAGAAAAGAAAAGAAAAGAAAAGAAAAATCTGTA

GTGAATCTCCCCAACTCCGCACATATGACCGAACATCCTGGAGATAAACCCATCGTCATC

TTCCTCCGCCCCTCGTTCTCCCCCATCAGCGACATCCTCTCCCCTCGCTTCCACCTCATC

AACTCCTGGGACTCCTCTCTCCCCACCTACCAGTTCCTCTCCCAGCCCCACGCCGCCTAC

GCCCGCGCCCTGGTCGTCTCCGGCGGCACCCCCGCCGCCGCCGACACCCTCCGCCTCCTC

CCCAACCTCGAGTTCCTCATCACCACCAGCGCCGGCTTCAACCACATCGACCTCGCCGAG

TGCCGCCGCAGGGGGATAAGGGTCGCCAATGCCGGCGGAGTCTTCTCGGTCGATACCGCC

GATTACGCGGTGGGGCTGCTGATTGATGTCCTGAGGAAGGTGTCGGCCGGGGATCGGTAC

GTGAGGAGGGGCATGTGGCCGGTTAAAGACATGTTTATGCTGGGCAACAAGGTTGGTGGC

AAGCGAGTAGGTATGGTTGGACTGGGGAACATTGGCTCCGAAATCGCAAAGAGGCTGGAG

GCCTTTGGCTGTATCATCTCCTATAATTCAAGACAACGAAAACCATCCCTCTCGTACCCT

TACTTTTCCAATGCTCGCGATCTTGCATCTGAGAGTGACATTCTAGTTGTATGCTGTGCC

CTGAACAACGAAACGCACCATATGATCAACAAAGATGTTCTGCTTGCCTTAGGAAAGGAG

GGGGTTGTGATTAACGTAGCGCGCGGGGCCCTGATCGACGAGAAGGAACTGGTCCAGTGC

TTGATGCGGGGAGAGATCGGAGGTGCTGGTCTCGACGTGTTTGAGGATGAACCTAATGTG

CCTGGAGAGCTCTTCAACATGGATAATGTCGTGTTGTCGTCTCACAAGGCTGTCTTCACC

AAGGAAGCCATTGATGATCTGGGAGAGCTAATCGCAGCCAATTTAGACGCCTTTTTTGCG

AATAAGCCTTTGCTTACTCCTGTCCCATTGCAGTGA

>TRINITY_DN10208_c0_g1|m.336 TRINITY_DN10208_c0_g1|g.336 ORF TRINITY_DN10208_c0_g1|g.336 TRINITY_DN10208_c0_g1|m.336 type:complete len:106 (-) TRINITY_DN10208_c0_g1:1952-2269(-)

ATGAGGGAGCAACGGCGTCATCTAAGTCGACCAACACATCGGCATGGCGGAGCAGAGGTC

GTGTCTACATCGAGTGAGGTGGAAGACTCGAGCATCGCGTCGAGGGGCTGCTCGATCGCA

TCTCGACTCAAGAAGTCGATCATGTCTAGCCACGAGGGAACGACGGCATCGGCTAGGACA

ACCAATGCGTCCGCGGACAGAGCGACGATCGCGCCTCGACGAAGAGGCCATGGCGTTCAG

CTGATGACGCGTTCGCATCTGCAAGGGAAGAGATTGTGGAATGGCATCGGGATTTGGGTG

AGAAGGCATGGGGGTTAG

>TRINITY_DN1020_c0_g1|m.341 TRINITY_DN1020_c0_g1|g.341 ORF TRINITY_DN1020_c0_g1|g.341 TRINITY_DN1020_c0_g1|m.341 type:complete len:105 (-) TRINITY_DN1020_c0_g1:714-1028(-)

ATGATGGAATCGACCTCTGGCACCGACCTAGAGACAGTGCTACAATCCCCTCCAGAAGTA

GCGATTTTAATCCACTTTTCCGTGAAGGAACTGATTTTTGGGTTCGGCCTAGAGATGGTA

CAAACTTCCCCAGAAGCAGTAATGATATGCATCCACTTTTCCGCGATGGAATTGACCCTT

GGCATCGGCCTAGAGAGAGTACAACCGTCCCCCGAAGTAGCGATGCAGATCCGGCTTTCC

CTGATCGAGCTGAACTTTGGCATAGGTTCAATGTGTATGGCCTTTGTGCCACTGCTGACA

TCGGAAGGGGGGTAG

>TRINITY_DN1020_c0_g1|m.337 TRINITY_DN1020_c0_g1|g.337 ORF TRINITY_DN1020_c0_g1|g.337 TRINITY_DN1020_c0_g1|m.337 type:complete len:734 (-) TRINITY_DN1020_c0_g1:430-2631(-)

ATGGCGGATGAGGGAAATGAGAGCACGAGCGGAAGAATGGATCTGAATCTTTATTTGGGT

TTTCGTCGATCTCCTCGGCCTCGAAGTTCAGACCTTGGATCTGATCTTGCCCTCAGCTCG

CTCCCATTGTCCACTTCATCCGCAACTGAAGAAAGCCGTGCCCCGGTGGAGATGACTAGT

GCTTTAGAACCCACTAATTCTCATGCCATGTACTCTCCTTCTCATGCTTCATTCATCCAT

GAGTCCAGCATTCCACCTGTCGACCCTGTTGTTCCCGGCGATGGGGATTCCAATCAAGAA

TACACCCCTTACTCTCCTTCTTATGAGCCATACGCTCCTAGTCCTGTAATGTTACCGGAA

TCCGAAGAGCTTGACATTTCAGACTCTCCGCCATCACCTGCCCATCCCTTGCCACCTCTT

CAAGAGACTGTAGAGCCACCCTTGTCTCCGCCAGGCCACCATGAACCTCATGCTGCTTAT

CCAGCTTCTTACACCCCTTCCTACCTTCCTCGGTCTCCATCTTACATTCCTTTAGACCCC

CAAGAAGCAAGTGATGACCCGTTGCCAGTGTCTCCGCTGCGGACGGTGTCTCCACTTATA

AGACCGTATATCCCCCCTTTCCTGCAGGATGTCTCCATGTATGATCCTCCGATGCCGTCT

TATGTTCCGAGGTCACCGTCTTACTCTCCTCCTTTGCCCACATACTCTCCTCAGATAGCT

GAGCCATTGGGTAGGGATGATGAGTCATCTTCGAGGCGTGATTTGCTTGAGTCTCCTGAG

TATCGTTTGAGGAGATTGGTTGAGTCAAGTCACCGAGCTCGACTTAGAAGGTTCCGATCG

TCCCTTCCGTACCATGGTGGCAGTGAAAGGTCCAATTTTAGCCGGTCTGCTTCGCCTGTT

CCTGAGCAACAGCAGCCGATACATGACATGATCAGTTCTCAGCGATCCTCAGAAACTAAT

GGGAAGCATAAAGTTCCTGCAGAAGGTATTACGGCTGAGAGTTCGGAAGAGGAGAAGGAA

GAAAAGAGCCAAAATGCGGCTAATTTTGAGTGCAATGTCTGTTTGGAGATGGCCGAAGAA

CCTGTTGTTACTTCATGTGGTCATTTGTTTTGCTGGTCATGTCTCTATCAGTGGTTACAT

ATCCATAGCGAGCATAAGGAATGTCCAGTATGCAAAGGAGAGGTTACTGAAGCTAATATA

ACACCTATTTATGGTAGAGGGAGCTCGGAAGTTAGTGATGAGAAAAAGCATGTCGAGAGT

GGGGAATCCAATGTAAAGATTCCCCCGAGGCCTCGTGGGAATAGGTTTGAGAGCTTAAGG

CAGCAGTTCAGGCCCATCTCCAGAAGATTTGGGGAAGGTATCACCACCTCTTGGAGGCGG

CTCGTGAATCAGCAAATGCGCAACAGGAATAGGTTTGAAGGGCATGGAGATCTTGCTATT

GGTGCTGGAATGCTCAATGAGCATCATGTTTTGACTAGGCTGAGAGCAAGGAGACTCCGG

CGGGAAGAACTGAATACTGATTTTGGGCCTGAAGACGAAGATAATAATTTGCCTGGTGAC

AGTACAGCAGTCCCTCAGAGTAGTATCACGAATCCATTTCTCCATGATGGAATCGACCTC

TGGCACCGACCTAGAGACAGTGCTACAATCCCCTCCAGAAGTAGCGATTTTAATCCACTT

TTCCGTGAAGGAACTGATTTTTGGGTTCGGCCTAGAGATGGTACAAACTTCCCCAGAAGC

AGTAATGATATGCATCCACTTTTCCGCGATGGAATTGACCCTTGGCATCGGCCTAGAGAG

AGTACAACCGTCCCCCGAAGTAGCGATGCAGATCCGGCTTTCCCTGATCGAGCTGAACTT

TGGCATAGGTTCAATGTGTATGGCCTTTGTGCCACTGCTGACATCGGAAGGGGGGTAGGA

AATGGTAATGGATATGGAGCCTCAACATCTTCTGCGAATCCTCCAAGTCCTGAGCAGCCA

ATTTCAAGGGCGCATGTAGCTTCGGGTTCTGCTGCTGATCGGGTCTCTGCTTCAAGCACA

ATTGCAGTGATACAGGGAGACGTTGGTCTTCCAGAAGGTTCAGCAGAACCAAATAGTGCT

GGATCATCCAGGCCTTACAGGAGGAGAGCAAGAAGCAGTGCTTCTGGCTCGTTAGATGTG

GATGGAAGCATGCATGCACGTAAGAGGACAAGTTTGAATTAA

>TRINITY_DN1020_c0_g1|m.338 TRINITY_DN1020_c0_g1|g.338 ORF TRINITY_DN1020_c0_g1|g.338 TRINITY_DN1020_c0_g1|m.338 type:complete len:184 (+) TRINITY_DN1020_c0_g1:766-1317(+)

ATGCCAAAGTTCAGCTCGATCAGGGAAAGCCGGATCTGCATCGCTACTTCGGGGGACGGT

TGTACTCTCTCTAGGCCGATGCCAAGGGTCAATTCCATCGCGGAAAAGTGGATGCATATC

ATTACTGCTTCTGGGGAAGTTTGTACCATCTCTAGGCCGAACCCAAAAATCAGTTCCTTC

ACGGAAAAGTGGATTAAAATCGCTACTTCTGGAGGGGATTGTAGCACTGTCTCTAGGTCG

GTGCCAGAGGTCGATTCCATCATGGAGAAATGGATTCGTGATACTACTCTGAGGGACTGC

TGTACTGTCACCAGGCAAATTATTATCTTCGTCTTCAGGCCCAAAATCAGTATTCAGTTC

TTCCCGCCGGAGTCTCCTTGCTCTCAGCCTAGTCAAAACATGATGCTCATTGAGCATTCC

AGCACCAATAGCAAGATCTCCATGCCCTTCAAACCTATTCCTGTTGCGCATTTGCTGATT

CACGAGCCGCCTCCAAGAGGTGGTGATACCTTCCCCAAATCTTCTGGAGATGGGCCTGAA

CTGCTGCCTTAA

>TRINITY_DN10210_c0_g1|m.342 TRINITY_DN10210_c0_g1|g.342 ORF TRINITY_DN10210_c0_g1|g.342 TRINITY_DN10210_c0_g1|m.342 type:complete len:130 (-) TRINITY_DN10210_c0_g1:622-1011(-)

ATGGCCTCCGCTGCCGCATCGAAAAAGTTGCTGCTCGCCGATGACGTCCCGTGGCGAGCT

TCTCCTTCTGGCACGAAGCCCCTTCCCAAAATCCACCAGAGCCCTATCCTCAAGCTCCCC

CAAAACCCTAGCTCCAGCTACGCCCTCGCCGTGATGAAGCATCCGGATCCAATTGGGGAG

GGGTTTGCGATGGAGGCGAGACTGGAGGCGGCGGGACCGGAGTGCATCGTGCCGGGTCAG

GCCACTCCTGTGAAGTTGCTTGGATTGAAGGTTTGGCCTATTGAAGTTGACTTAAAGTTT

CTGGAACCAGTTGGACGGCAACTTAAGGACATTGGGAAGTTCATGGATTCAGCTGTAAAG

CTCATGGATGCAGCATTTCAAGATCGTTAG

>TRINITY_DN10213_c0_g2|m.343 TRINITY_DN10213_c0_g2|g.343 ORF TRINITY_DN10213_c0_g2|g.343 TRINITY_DN10213_c0_g2|m.343 type:complete len:249 (+) TRINITY_DN10213_c0_g2:49-795(+)

ATGGCCTCCGCTTCCTTCAGCACCCTAGCGAACGCTCGGCCGGCGGTGTCCGCTCCAGCG

ACGACCCGCCGCTGCGCCGTCGCGAGCTTCCGGAAATGCCCTCGGTGGGCCTTGGATATC

TCGTCGGCGTCCTTCTCCCCTCTCTTCTCCTCCTCGCCCGCCCCTTCTCGGATCCAGACC

CGACCCGCCAGCTCCATCCGATCCGCATTGCAGGAGCCTGCACTGCAGTCTAAAGTCACC

CAAAAAGTTTACTTTGACATAAGCATCGGAAACCCAGTGGGGAGTCTTGTTGGTAGGATC

GTGATCGGTCTTTTTGGGGATGATGTGCCCCAAACAGCCGAAAACTTTCGAGCACTTTGT

ACAGGAGAGAAAGGCTTTGGGTACAAGGGATCTGCATTCCACCGTGTCATCAAAGATTTC

ATGATCCAAGGGGGAGACTTTGATAAGGGAAATGGAACCGGAGGTAAAAGCATATATGGG

CGTACATTTAAGGATGAGAATTTCAAGTTGGTGCATGTGGGACCTGGAGTTCTTAGCATG

GCAAATGCAGGACCTAATACCAATGGAAGTCAATTCTTTATTTGCACTGTCAAGACCCCA

TGGTTAGACCAGAGGCACGTCGTGTTTGGGCAGGTCCTGGAGGGAATGGATGTGGTCAAA

TTGATTGAATCTCAAGAGACGGACAGAGGGGACCGCCCTAAGAAGAAGGTGGTCATCAGC

GATTGCGGCGAGCTTCCACTGGTCTGA

>TRINITY_DN10213_c0_g2|m.344 TRINITY_DN10213_c0_g2|g.344 ORF TRINITY_DN10213_c0_g2|g.344 TRINITY_DN10213_c0_g2|m.344 type:complete len:164 (-) TRINITY_DN10213_c0_g2:73-564(-)

ATGCACCAACTTGAAATTCTCATCCTTAAATGTACGCCCATATATGCTTTTACCTCCGGT

TCCATTTCCCTTATCAAAGTCTCCCCCTTGGATCATGAAATCTTTGATGACACGGTGGAA

TGCAGATCCCTTGTACCCAAAGCCTTTCTCTCCTGTACAAAGTGCTCGAAAGTTTTCGGC

TGTTTGGGGCACATCATCCCCAAAAAGACCGATCACGATCCTACCAACAAGACTCCCCAC

TGGGTTTCCGATGCTTATGTCAAAGTAAACTTTTTGGGTGACTTTAGACTGCAGTGCAGG

CTCCTGCAATGCGGATCGGATGGAGCTGGCGGGTCGGGTCTGGATCCGAGAAGGGGCGGG

CGAGGAGGAGAAGAGAGGGGAGAAGGACGCCGACGAGATATCCAAGGCCCACCGAGGGCA

TTTCCGGAAGCTCGCGACGGCGCAGCGGCGGGTCGTCGCTGGAGCGGACACCGCCGGCCG

AGCGTTCGCTAG

>TRINITY_DN102146_c0_g1|m.346 TRINITY_DN102146_c0_g1|g.346 ORF TRINITY_DN102146_c0_g1|g.346 TRINITY_DN102146_c0_g1|m.346 type:5prime_partial len:435 (-) TRINITY_DN102146_c0_g1:2887-4191(-)

AAACACCATTGCTTTCTGCCATCAATCGAGACAATGCTCGCGAGACCGTCCACAGCCCGT

CTTCCCTCCCTCGTCTGCCGCACGTGGGCTCGCAGCCTCTCAGGCGTCGCTCCCACTCGG

ACGCCGACGACGACGCTGGCCCCACGTCGACTGGACCTGAACCCCACCAAGTCCATCAAC

CCCTCCATCCGTCTCAAACTCTTCCCTTCGAGAGCTGCATCTACACCATCATATTTTGTA

TTTACTAGACAATTGAACACCATGGCCCCCGTTAAAGAGTTTTCCCTCCTCTGCCTGGAG

AACCCTCTCCTCGATATCCAGGCTGTCGGTGATCAGGCCCTGCTCGACAAGTACGGCCTC

AAGGCCAACGATGCCATCCTCGCCGAGGAGAAGCACATCCCTCTCTACGAGGACCTCCTC

AACAACTACGATGCCAAGCTCATCGCCGGCGGTGCTGCCCAGAACAGCGCCCGAGGTGCC

CAGTACATCCTCCCTCCCAACAGCGTCGTCTACATCGGCGGTGCCGGCGACGACAAGTAC

TCTGCCATCCTCCACGACGCCGTCAAGGCCGCTGGCCTCCGCGTCGAGTACCGCGTCGAC

CCCAAGGAGAAGACTGGCCGCTGCGGTGCTATCATCACTGGCCACAACCGAAGCCTGTGC

ACCGACCTCGGTGCTGCCAACCACTACGACCTGGATCACCTGAAGCAGCCCGAGATCTGG

AAGCTCGTCGAGAACGCCGAGGTGTACTACGTCGGTGGCTTCCACTTCACTGTCTGCCCC

CCCGCCATCATGGAGCTTGCCAAGCAGGCTGCTGAGCACAACAAGATCTTTGTCCTCTCC

CTCTCCGCCCCCTTCATTCCTCAGTTCTTCAAGGAGGTTGTTGATGCCAGCGCCCCCTAC

TGGGACTACATCATCGGTAACGAGACCGAGGCCGCTGCTTACGCCGAGTCCCACGGCCTG

CCCAGCAAGGAGCCCAAGGACGTCGTCAAGCACCTTGCCAACCTCCCCAAGGAGAACACC

AAGCGGAAGCGAGTTGCCATTGTCACTCAGGGCACTGACCCTACCCTTGTTGCCATCCAG

GGTGAGGAGGGTATCAAGGAGTTCCCCGTCCACGCCATCGAGACCGAGAAGATCAACGAC

ACCAACGGTGCCGGTGACGCCTTTGCCGGTGGTCTCCTTGCCGGTATCCTCGAGGGCAAG

CCCCTTGAGACCAGCATCGACCTCGGCCAGTGGCTCGCCCGTCTCAGCATCCAGGAGCTT

GGACCTTCATACCCCTTCCCCAAGCAGACCTACCAGGGTGCTTAA

>TRINITY_DN102146_c0_g1|m.345 TRINITY_DN102146_c0_g1|g.345 ORF TRINITY_DN102146_c0_g1|g.345 TRINITY_DN102146_c0_g1|m.345 type:5prime_partial len:738 (+) TRINITY_DN102146_c0_g1:3-2216(+)

CGTTACTACGTTCAGTCCGGTCTGAGAAAGTCGAGCTACAGAACCGCATTGGCCTACTAC

AGCCTTGCCTACGACCTCAAGTCCGATTCGGGCTTCCCCTTCCACCAGATGGGCATTATC

AGCCTGGAGGAGGGCAAGGACCTCGACGTCGTCTACTACTTTTACCGGAGCATCGCGACC

GCCGACCCTCACCCCAATGCGAGACAGAACCTCGAGTCCAAGTTCAAGACCATTTTGCAG

CCGGATAAGAACCCGTCCAAGAAGCAAACACGTGGGCCGAGCGATGCCTTTGTCACCTGG

TTCACCAAGCTCCACGCCTCGTACTATCGGGATGAAGTCATGTCACACAGCTCCGAGCTC

GAGAGAGAGGTCCTGCACCGTCTTGACATGGCCTCTAAGAGCGCTAGCCACACCCAGGTC

CTGTTCAAGATGGCATTGATCAACATGTCGAGCTACTATGTTGCCACGCAAAAGTTTACC

GAGTCTCAAACCCCTGCGACATCGCGCTTTTGCCAGCATACTTTGCGCCTGAACTCTCAG

TTTATCCTGGCCTTTTGTGCTGCACTGGAATCTGAGCTCACCGAAATTGTTAGTCGCGAA

AGTCACGAATCCGAGGACCCGGCTACCGCAAAGTCATCTCCTACTATCACTTCGCTCCTC

CCACTTCTCCGCATCTATGGCATGTGGCTCGCCGCTCGCCGCCACGAGATCTTTGCTGCT

GGTGAAGCGCTGGGCGCAGTTCTGCCCGATATGGTCAAAGCGCTCTCCAAGGTCTTCTCT

CTCTTGTGCAACGACACATACACCCAGGAGAACCTGGCTTCGTGCCCTTACCTGCTGGCC

GAGGATGTCGACACCCAAGGATTGCTTCCCCTTTCCCAGGAACATGTTCCTCTGGCTTGC

CGATCTTACTTTAACGAGAATGGCACCCTGAAACCACGACTGCCGTCTCAAGAGAACCGA

CTTGATCCCTTCCAAGAGATGCTGGCCAGAATTCTGGACATCCTTCGCTGCGCCTATTTC

CTGGCTGAAGACGACTCTTGCCCCCTCACATATCGTGTCTCTGAACGGGGTCTGGTCTTT

GAATATCAAGACGTCCCTGTTGTGAGCAACGCCCCCGCTATCAACAATGTCCCCGTTATG

AACAATGCTCCCGAGCCGATGTCAATTGTCAATTCCCCTGTGCGCGAACTCAAGGTCAAC

ACCAAGAACCCCGACCGGCAGCGCACTGCATCTGATAATCGACAGCCTGACAATCGACAA

ACCGAAAACCGACCTCCAGCAGACGATGGCGCGAGAAAGGTATCCCTCACATCTCCCCAA

CCCACGGTTCAACCCCAGCCTCAACCCCAGGCTCAGCCTCGAGCACCTTCCCCCGCCCAG

AGCGAAAGCACCTTTATCCTCGGCATGCTCACCCCTTTCCTGAAGCCCCCGATCTCGGAT

GCTACCCAGTCATCCCACCGGTCTAGCGATGAGACTTCCTATGGAATGCATAGTGCAACT

GCAAATGAGGTCTTTGGGATGATCCAGCAGCCTGAGCCCAGCCCAACCGGTTCGATCACC

TCTGGCAAGCTGAAGCCCCTCCCATGGGATTGGGTATACACGCCCACCCCTCATAAGGCG

ACTGGCCCCTCCGCCATTGCTTGCAAAGATGTCTTCGACGCCCCTGTCAGCCCCAACTTT

TCGTCCCGAGACCTGCCCCGAGCGACTGTCAGTACTCTGGAGGATCCCTTTGCGAGCACT

TCGCCTCAGCAACTCCCGACGAACCTGACCCCTCGGGTGGCAAGCGGTATGATGGGAAGC

CCTCGCCGCGTTTCAGCTGCAGATGAAGCCGCTCACCGCAACAGCCTGCTTCAATCCTTT

GTGAGCACCAGCGCTCCCCGAACGTCTACGTTCAGCCAGTGGGGTCAGAACTCGTCCCGC

ATTCCCAAGGAGACTGTCCCCTCGTCCTACGGCCACCACCAGGCCTTCAACGGCTATCCC

ATGTCCTCCGCGTCTGCCTTCTCCCACCCCAGCAGTCTGTACCAGGGAACCCCAGCTAAC

GGAGCTGTCTATGGCATGCCGTCTGGAGGTTACATGGATATGAGCCAGTATAATCGCACT

CAGACTCAGGAGTCGATGACTGTTCCGTCCGCCGCCCGGCGTTTCCAGATGGATGAGACG

GCTCTGAACTACGACGCCGCTATTCTTCAGGCTGCTTTTCGCGACAACAAGTAG

>TRINITY_DN10214_c0_g1|m.350 TRINITY_DN10214_c0_g1|g.350 ORF TRINITY_DN10214_c0_g1|g.350 TRINITY_DN10214_c0_g1|m.350 type:complete len:280 (+) TRINITY_DN10214_c0_g1:326-1165(+)

ATGTATAAGGAGCGAGGGATCGGATCGAAGTCCGAGATCGGGCCGATCGATCGGAAGCGG

ATCAACGACGCACTCGACAGGCATCTGGAGCGGTCGTCGCCGTCGACGTCGAGGGGGCTG

AAGGACAAGTATCGCGTCTCGTTGCCGTCGACCTCTTCGGGCAAGCACCCAGATCAGCTC

CCTGGATCGATGCCCGAGAACAAGTGCTCCAATGAAGAATCCGACACAGATAGTGAAGAG

TCGGATGTTAGTGGTTCAGAAGGAGAAGACACATCTTGGATTTCATGGTTCTGTAATCTA

AGAGGGAATGAGTTCTTCTGTGAAGTTGATGATGAGTACATACAAGATGATTTTAACCTT

TGTGGCCTGAGCAGTCAACTTCCTTACTACGATTATGCCCTTGATCTGATTTTGGATGTT

GAGTCTTCTCATGGTGATATGTTAACCGAAGAACAAAATGAACTGGTTGAATCGGCAGCA

GAGATGCTGTATGGTCTTATTCATGTGCGCTACATTTTAACTAGTAAAGGGATGGCCGCT

ATGCTAGAAAAATTCAAGAATTATGATTTTGGAAGGTGCCCTAGAGTTTACTGCTGCGGT

CAGCCCTGCCTTCCTGTTGGTCAATCAGACATTCCTCGATCAAGTACTGTGAAAATCTAC

TGCCCTAAATGTGAAGATATATATTATCCCAGATCCAAGTACCAAGGCAACATTGATGGA

GCTTACTTTGGCACAACATTTCCTCATCTGTTTCTGATGACATACGGCCACCTGAAGCCA

CAGAAGGCATCTCAGCGATATGTTCCAAGAGTGTTTGGCTTTAAAATCCACAAGCCATGA

>TRINITY_DN10214_c0_g1|m.351 TRINITY_DN10214_c0_g1|g.351 ORF TRINITY_DN10214_c0_g1|g.351 TRINITY_DN10214_c0_g1|m.351 type:5prime_partial len:111 (+) TRINITY_DN10214_c0_g1:1-333(+)

AAAATATCATCGGAGCAGGGACCCCTGTTACCTCGAACATCCGCCCAAATTACAACTCTA

CCCTTCCTCCACCTCTCTCTCTTCCCCCCCTTTTCATTGTCCTCGTGCTCTCCCAAGCTC

CTCAAGAGCCCGACGAGGGTTCGCTCGCTCTCTCTTCCCCCTCTCGGAAAAAACCCTAAC

CCTAACCCCAACCCTAACCCCAACCCTAACCCTAACACTTATTTTTATTTCCACCGAGTG

CGTATCGAATCTGAACACATTTCTTTGTTTGTCAAATCGGCATGTTGCGGCGAGTTGATT

GAGCTAGGTATCGGAGGAGGGAGAGATGTATAA

>TRINITY_DN10215_c0_g1|m.352 TRINITY_DN10215_c0_g1|g.352 ORF TRINITY_DN10215_c0_g1|g.352 TRINITY_DN10215_c0_g1|m.352 type:5prime_partial len:335 (+) TRINITY_DN10215_c0_g1:1-1005(+)

CCCAAACCAAATTTACCATCTTTCTTCGATTCTCCTCTCGATTCGATTACAGAAAGCGAG

AGAGCGATGGAATCAGCATCGATGCTGAAGAGATACGAGAAATTAGGGCTTGGCGATGCC

CTAGCTCGCCGTTACGATTACCATTGGGCCTGCCACGAACTCAGCCTCATCCTGAGGATC

GCATATCCCAAGCTCCCCAAGAATCTACAAGCCCTCGTCTTTAGCGATGTTCTCTCCGCC

TTTCGCCTTCTCCCCGAGGTGCAGACCTGCCGTGGAATTTCATCGGCGAACCTCCTTGTC

CAAGCCGCAGAAGCCTCACTGCCTAAGCAGAAAAGGTCATTGGCTGTTTCAGAATTCAAG

CATGCTGTTGTTGCACATAAGAGGCGTGGTAAAGCTCATCAAGATCAAGGTTCAATGCTA

CTCCCACAAGACGTTCTTGTTCATGTATTCAGTTTTCTAGACTTGAGGTCTTTAGTAAAC

GTCAGTTCAGTATGCTGGGCATGGTGTTCAGCTGCTGCTGATAATACATTGTGGCAGTTA

CAGTATTCCCTTATGTTTGGCGATTATGATAACAAAACCAATGAGCAGTCGCAGAAACTG

TCTGGGAAGAAGAAAGTAGTTTTGCACCCACCTGTACAAGATGTAGATGCAATGTCCAAC

ATTGACTGGAGAGACGCTTTTAAAAGAAAATATATAGGTAATTTGGCTTGGAGATCTGCA

ACACATAGGGCTATATGTGGCCATTGTAAATCAATTATTTGGCTCAGCCATATGACAAGT

GCCAGGTCACACCATTGCCAAAAAGTTATGAATAAGAGGTTTATAGTAAATCCTGTTTCA

GCACACAAGGTTGTGGACTACCTCCTTGGGGAGACAGACTTCACAGCATCGTCATCTGAC

AGCGATGACAGTGATTCTGATGGTCCATCGCTCATCCAGCGAATGCATAAGCTATGGGTC

TATCCAAAACTAGCAAGCAGCGCAGAGAAGCCTTCTGAAATTTGA

>TRINITY_DN10218_c0_g1|m.353 TRINITY_DN10218_c0_g1|g.353 ORF TRINITY_DN10218_c0_g1|g.353 TRINITY_DN10218_c0_g1|m.353 type:complete len:231 (-) TRINITY_DN10218_c0_g1:260-952(-)

ATGCCTATGGAATCAGAGGAAGCATTTGATGATGACTTCATGGTAAACAGACCCCCACTG

GGGCCAATAGATCCTGGGAGAGCCCGATTTCCATGTTGCATTGTGTGGACTCCTCTTCCA

GTTATCACCTGGTTTATACCTTTCATTGGCCACATTGGCATTTGCAGAGAGGATGGGGTG

ATCTTGGACTTTGCGGGGCCAAACTTTGTATGTGTCGACAATTTCACATTTGGTGTGGTG

GCCCGTTTCGTACAATTAAACAGAGAAAAGTGCTGCTTATATCCTACAATGGCCGAGGTT

GCATCTCGTGACAGCAATAAACAGAGAGGTTCGAACGAAATTATCTCCTGGGATGACGCG

CTTAAGAAGAGCACACAAGAGTATCAGCACAGAAACTATAGCTTGCTCACCTGCAACTGC

CATTCCTTTGTAGCTAACAACCTAAACAGAATGGTTTACGGTGGGCACAACAAATGGAAT

GTGGTGAACCTAGCAGTGTTGATGTTCTTCAAAGGTACTTGGGTGAGCAAAGCATCAGCA

GCACGATCTTTGTTACCTTTTGTGGTGGTGTGCTGCCTAGGGCTTCTGTTTGGGGGTTCG

AACTTCCTGATGGTCGTGGGTGTTTTTGCTCTCCTTCTTGTGGGTTGGTTTATCATCGGT

ACTTACTGCCTCAAGAATCTTATCCAGTTGTAG

>TRINITY_DN10219_c0_g2|m.354 TRINITY_DN10219_c0_g2|g.354 ORF TRINITY_DN10219_c0_g2|g.354 TRINITY_DN10219_c0_g2|m.354 type:complete len:493 (+) TRINITY_DN10219_c0_g2:351-1829(+)

ATGGCTTCTTCTTCTTTCTTCTCTCTTCTGCTGCTCCTTGTTCTGAGCTTTTCTTCATCT

ATTTCTTCGGCTCCGGATGGCGCTCTCGTCACCGTCGTCCCCGGCTTCGACGGGACTTTC

CCTTCCAAGCACTACGCTGGGTATGTGACCATTGATGAGAAGCATGGAAGGAATCTGTTC

TACTACTTTGTGGTTTCCGAAGGGAACCCATCTAACGACCCCGTCGTTCTTTGGCTCAAC

GGCGGCCCTGGTTGCTCTAGCTTAGACGGTTTCGTTTACGAGCATGGCCCATTCAATTTT

GAGGCTGGAGAGGCGCCTGGCACGTTGCCCAAACTGCATCTCAATCCCTACAGTTGGTCC

AAGGTGTCTAATATTATCTATTTGGACTCACCTGCTGGGGTTGGACTCTCGTACTCGAAT

GACACATCGGATTATGATACAGGAGATCTTAAGACGGCTTCGGATACACACACTTTTCTT

TTGAAGTGGTTTGATTTGTATCCTGAGTTTCTTAAGAACCCTTTTTATATTGCCGGTGAA

TCATATGCTGGGATTTATGTTCCGACTCTTTCAGCCCAAGTTGCAAATGGAATAAAAGCC

GGTGTAAAGCCTGAACTGAACTTCAAGGGATATATGGTAGGAAATGGATGCACGGATGAC

AAATTCGATGGTGATGCTCTCGTTCCATTTGCGCATGGAATGGGTCTTATATCCAATGAT

CTGTTTCAGGAGGTTAATGTTGCCTGTAAAGGAAGTTACTGGAATCCCCTAAATCAAAAC

TGCCAAGACAAGCTAGACAAAGTGGACTGGGAACTTAACAACATAAATGTCTATGACATC

CTTGAGCCGTGCTACCATAGTCCTGGAATCAGATACCCCTTTGCTGATAACAGCAGGGTC

CCTGTTAGTTTCAGAATGCTTGGGGAGACTGACAAGCCACTTCCTGTGAGGAAAAGAATG

TTTGGCCGCTCTTGGCCTTTCAAAGCTCCAGTCAAAGCTGGACGCGTTCCAACATGGCCG

GAGCTGGGAAGCAATTCAGTTCCGTGCACAAATGACGAAGTTGCAACTGCATGGTTGAAA

AATGAAGCTGTGAGAACCGCAATTCATGCACAGCCGGCAAATGTGGTTGAATGGATGCTA

TGCACTGATAATATAAATTTTCATCATGATGCTGGAAGTATGATCAAGTATCATGAGAAT

CTTACTGCGCAAGGGTATCGTGCACTAATATTCAGTGGAGATCATGACATGTGTGTGCCT

TTTACCGGGAGCGAAGCATGGACTAGATCATTGGGATATAAAACTGTAGATTCATGGAGG

CCATGGTTTTTTAATGACCAAGTTGCAGGATACACCCAAGGCTACGAGCATGATTTTACC

TTTCTTACAATAAAGGGATCAGGCCATACCGTTCCAGAGTACAAACCACAAGAGGCATTG

GCATTCTATAGCCGTTGGTTGGCAGGCAGGAAAATCTGA

>TRINITY_DN1021_c0_g1|m.356 TRINITY_DN1021_c0_g1|g.356 ORF TRINITY_DN1021_c0_g1|g.356 TRINITY_DN1021_c0_g1|m.356 type:complete len:355 (-) TRINITY_DN1021_c0_g1:1017-2081(-)

ATGGTGAAGGATGAAGTGGTGGTGGAGGAGGAGGAGGTTGTGGAGATTGTGAGCCCAAGG

CCAATGAAAGGCTTGCATGAGATTGGGCCACCTCCATTCTTGACCAAGACATTTGAGATG

GTGGAGGATCCAGACACTGATTCAGTGGTCTCTTGGAACCACGCAAGGAACAGCTTCATA

GTTTGGGACTCTCACAAGTTTGCAGGCACTCTGCTCCGTAAATACTTCAAGCACAGCAAC

TTCTCTAGCTTCATTCGCCAGCTCAACACCTATGGTTTCCGAAAGGTTGATCCAGATCGA

TGGGAGTTTGCCAACGAGAGATTTCTAGGTGGTCAGAAGCATCTTTTGAAGTACATCAAG

AGGCGGCGACACATCAACCAGTCCTTACAACAACAGCAGCAACAGCAACTAGGAGTCCAT

ATCCAACCTGGACAGATAAGCGAGGCATGTATTGAACTAGAACAGTTTGGACTCGAAACT

GAAGCAGAAAGGCTTAAGAGTGACCACAACATTCTGGTCCTGGAGATTGCCAAGCTCAGG

CAGCAGCAGCAGACCTCTCGTGCCCAGGTCATCGCGATGGAGCAGATGATACAAGGCACA

GAGAGGAAGCACAATCAGATGATGGTCTTCTTGGCCAAAGCTCTCAGGAATCCAGGCCTC

GTCCAACAGCTGTCGCTTCGGAGCGAGGAGAAGCAGCAGTTGTTGGAGAATACAGGCAAG

AAGAGGAGATTGCCTCTAAACGCAGGCTTTGAGAATCTGCAAGCAAGTGAAGAAATTGGA

GTTGAATTGGAGATAAAAAACATGATTTCAGAAATGGTTAATGGCAGGGCAAGCAGCTCA

AGCAATCACCTGACGGTAGAGAAGCTGATGATGGAATCATGCAATGAGAATGTCGATGGC

ATTAGTGAAGTAATATGGGAGGAGTTACTGACTGAAGGTCTAATACCACCAGGAAATGGG

ACTGAAGAAGGTGACGACAAGGAGGTTGGTGTCGATGTGGAGGAATTGGTCGGAGATGCA

ATTGGATGGGGTGAGTATGTGCAAAATTTGGTGGATCAGATGTGA

>TRINITY_DN10220_c0_g1|m.358 TRINITY_DN10220_c0_g1|g.358 ORF TRINITY_DN10220_c0_g1|g.358 TRINITY_DN10220_c0_g1|m.358 type:complete len:396 (+) TRINITY_DN10220_c0_g1:210-1397(+)

ATGAGCAACGGGCCCCACCACAGCGACTCCGACGATGGAGAAGACGAAGAAGAGGTTTTC

TTGGACGTCGATGACGTCATCCAAGAGATCCCAATCGACGAAGAAGATCTCCCCGATCGC

GGCGATGACTACGAGATTGATTCAGAAGCTGATATTGAAGAAGCTGATGACTCGGTGTTC

GTGTTTCGAGGCCATACAGATGAAGTCTATACAGTCGCATGCAGCCCAATAGATGAGTCT

CTGGTGGCTACGGGCGGCAAAGACGATGGAGGATTTTTGTGGAGGATTGGGTCAGAAAAC

CCATTATTAGAGCTCAAAGGTCATAGAGACACAGTTTCTAGTTTAGCATTCAGCTCTGAT

GGACAGCTACTTGCATCTGGAAGTTTTGACGGGCTTATTCAAGTATGGGATGTGCTGTCA

GGTAGAGCCATGTGTTCGCTTGAAGGTGCAGGGGGTATAGAGTGGCTCAAATGGCATCCT

AGAGGACACCTGATATTAGCAGGATCAGAGGATGCTAGTGTGTGGTTGTGGAATGCTGAC

AAAGGCATCTTGCTCAACACGTTCTCTGGTCACGGTAGTAGTGTCACCTGTGGTGCATTC

ACACCTGATGGTAGAATTATCTGTACTGGTTCCGACGATGCATCGTTGAGGATATGGAAC

CCAAAAAATGGGGAGAGTATTCATGCTGTTAGAGGTCATCCATATCACACAGATGGACTG

ACATGCTTGTCCATAACCTTTGATTCTGCAATTGCTATTACTGGTTCAAAAGATGGTTCC

GTCCACATGGTGAACCTAGCGACTGGGAAAGTGGTTAGTTCTTTAGTTGCTCACTCAAGC

TCTGTCGAGTGCATTGAGCTTTCAACAAGTGGCCCTTGGTGTGCAACTGGAAGTATGGAT

CAGAAGCTTATAATTTGGGATCTTCAGCATTCTTCTGCACGCTGCAACTGTGATCATGAT

GAAGGCGTAACGTGCCTTTTATGGCTGGGCGCATCAAGATATGTGGCTACGGGCTGTGTG

GACGGGAAGGTTCGAGTGTGGGACAGCCTTTCTGGCAGTTGCTTGCGAACATTCAGCGGC

CATTCTGACGTCGTTCAATCTCTGGCTATGTCGAAGGATGGCAATTCTCTTGTTTCTGTC

TCCACTGATGGAACGGCCTGCGTATTTGGGATCGCAGAGTTTCGGTAA

>TRINITY_DN102221_c0_g1|m.359 TRINITY_DN102221_c0_g1|g.359 ORF TRINITY_DN102221_c0_g1|g.359 TRINITY_DN102221_c0_g1|m.359 type:internal len:108 (-) TRINITY_DN102221_c0_g1:2-322(-)

GACACTGCTCCTGAAATATCAGAAAAAGAAGAATCTCTGAAACACCCTTCAGCGACTGAA

GAGCATGACAGGACTTCTTTAGAAGTATCATCTGTACCTACAGCGGAAGAAAGTGACCCT

CTGGAGAAAGAAAGCATCGACTCTGAAATCAATGACAAAGATATTAGTGATCTTGACATG

GCTTTGAAGTCTAATGTACCTGTAGAACTTGATGAAAGCAAAGCTGAAGCTGAATCTCCT

TCTCAAATGGATACTTGTTTGCCCACTGCTGAAAATATGAATGTGGAGGATTCTCCTCTC

CTATCTCAGCAGAATGAGGTT

>TRINITY_DN10224_c0_g1|m.360 TRINITY_DN10224_c0_g1|g.360 ORF TRINITY_DN10224_c0_g1|g.360 TRINITY_DN10224_c0_g1|m.360 type:complete len:243 (-) TRINITY_DN10224_c0_g1:257-985(-)

ATGGCGACGACAATGATTAGTCCAAAGCTCCCACTTCAGAGGAATCACTCTCCTTTTAGT

CCTCCTCGAGTAACAATTTCAAGGCATGCGAAATGCAGTTTTGGAGTTAGTGGAATTACC

GGTGGTATCTCCCTTCGTGATGAATCAGAAGGTGCATTTAGAAGGTTATCATTTTCTTCT

GGAAGGGGCTTAAGTCATGTTGCTTGGCCCATATCTGCAGTTGGCTCAGGTTTAGAAGCA

TCAATCGCTGATAAGAAGAAGAATGACTTATCATTGGACAATGTTAAGATAGTCATCGAG

TCACGTGATGTTGATAAAATTAATGTGAGAGTGGACTTGACTGGGGAACAAACCCAAAAG

GCATTTGATGATGTACTAATTAACCTGGCTCGTACTGCACCACCTATGCCAGGATTTCGT

AGGATGAAGGGTGGGAAAACATCCAATGTCCCAAAGAGCTTTCTGTTACAGATGTTGGGG

AGAGACCGTGTTACTAAATTCCTCATTCAAGAAGTTGTCAGCACAACCATTGGGGACTTC

GTGAAGAAGGAAAATTTTACAGTTAAAACCCAGTTCCAGACAACGCAGACTGCTGAAGAG

CTTGAATCGGCGTTTACTCCAGGCGGCGAGTTTGGATTCAACGCTACTATGGAGATTGTA

CAACCTGATTCTGGAACAACTACAAGCAACTCCGAACCTGATTCTGATTCCTCTGAGCCA

GAGACATAA

>TRINITY_DN102296_c0_g1|m.362 TRINITY_DN102296_c0_g1|g.362 ORF TRINITY_DN102296_c0_g1|g.362 TRINITY_DN102296_c0_g1|m.362 type:complete len:183 (+) TRINITY_DN102296_c0_g1:45-593(+)

ATGAACTTTCCCGGGACCGCTCCTCCGACTGCTGCTACCGCCGCTGCTGCGGGCGCGATG

CCCACGCCCGGCGGACCTCAAGATCCCAACGTCAAAGCTGTCCAAGCTGCGATGGAGTCT

TGCTATGGAAAGTCGGTCATGTCCGGAGTGATGGGCTTTGGCATGGGTGGTGTGTTTGGA

ATGTTTATGGCTTCTATGTCCTACGATACACCCTACCACACGGCCGCCCCCGGCGCCCCG

CAGACCACAATCAGCTCCCTCCCGCTAAAGGAGCAGCTCAGGATCGGCTTCAAGGATATG

GGAACGCGGTCGTGGTCCATGGCCAAGAACTTTGGCAAGGTCGGAGCCCTGTACTCGGGC

ATCGAGTGTGGCGTCGAGGGTCTCCGCGCAAAGAACGACCTCACCAACAGCGTCGCCGCC

GGATGTCTGACGGGCGGCGTCCTGGCCAAGAACGCTGGCCCTCAGGCTGCGGCCGGTGGG

TGCTTGGCGTTTGCGGCGTTCAGTGCGGCTATTGATGCTTGGATGAGGTCGCCCGCCAAG

GACGAGTAA

>TRINITY_DN1022_c0_g2|m.364 TRINITY_DN1022_c0_g2|g.364 ORF TRINITY_DN1022_c0_g2|g.364 TRINITY_DN1022_c0_g2|m.364 type:complete len:134 (-) TRINITY_DN1022_c0_g2:51-452(-)

ATGGATGAACAGGATTTTGTTGATGCAGACAAACTGATATTGAGGGGGTTGCAGTTTTTT

GGATTTCATGGGGTAAAAATAGAGGAAAAGAAGCTAGGTCAGAAGTTCTTGGTTGATGTG

GATGCCTGGTTAGACTTACGTGTTGCTGGTCAAACTGATAGCATACATGATACTGTCAGC

TATACCGACATCTACAGGATTGCGAAAGAAGTTGTGGAGGGTCCATCCCAGAATCTCTTG

GAGTCAGTGGCACACCTAATTGCAGACACCACACTACTTAAATTTCCTCAAATCTCTGCT

GTTCGAGTGAAGGTCGTTAAGCCTCACGTAGCCGTTCCTGGCCCTCTCGAATCTTTAGGC

GTTGAAATAACGAGACACAGAAGGAATGACATGGGTAGCTGA

>TRINITY_DN1022_c1_g1|m.365 TRINITY_DN1022_c1_g1|g.365 ORF TRINITY_DN1022_c1_g1|g.365 TRINITY_DN1022_c1_g1|m.365 type:complete len:154 (-) TRINITY_DN1022_c1_g1:481-942(-)

ATGACGATGCATCAGCTGCAAAATCCTCGTCGTCCTTGTAGCATTGCCCTAGCACTATGT

CTGTTCTCAGTCTTCGTTGCTGAATCGAACTCCCAGAATGCCCAGCCATCTGAGCATGGA

CTCATGTATCAGAAGAATGCTGGGGAGGCATCCCCTGAAATGATTGCTTTCTTTGGCCGT

CCGAAGGTGGAGCTCCCAGAGGCAAAGAATGTATCTGATCCTGTCTGGAGAGCAGCAACA

AAGGTTGTTCCGGGAGATCCAGCTAACAGTGGTGGACATGCGAGACCCGCCCTCTTGATT

ACTGGGATTGTGTTTGCTGTTCTGGGGGTTGGCCTGCTTGCTACAGCGGTGGTGGCTTAT

ATGCTCCATGCCCGCAGGTCCAAAGCATGGTCAAGTTCCTGTTCTTTGGCAGGCTTGACT

CATCGGAGAAATGACCCAGTAGTCCAGTTGGGAGCTGTTTGA

>TRINITY_DN10231_c0_g1|m.367 TRINITY_DN10231_c0_g1|g.367 ORF TRINITY_DN10231_c0_g1|g.367 TRINITY_DN10231_c0_g1|m.367 type:complete len:527 (-) TRINITY_DN10231_c0_g1:2225-3805(-)

ATGGGGTTTGAAGATGAGGGGATTGGAATTATTGGTAAAGGGAAAGAGAAGGCTGGTATC

GGATTCGGGTTTCGGAGTCTGATGAGGAGGAAGCAGGTGGATTCGGATCGGGTTCGGGCT

GCAGACACCGGCCACCACAAGCTCGCCAAGGAGTTGTCTATCCTTCAGCTCATTGCTATA

GGTGTTGGCTCGACAATAGGCGCCGGGGTCTATGTTCTTGTTGGAACTGTTGCTAGAGAA

CATTCAGGACCTGCTCTGACCATTTCCTTTTTAATAGCTGGAATAGCTGCTGCACTTTCA

GCATTCTGTTATGCCGAGCTTGCCAGTCGTTGCCCTTCAGCCGGGAGTGCTTATCATTAT

TCGTATATCTGCGTTGGAGAAGGTGTTGCTTGGTTGATTGGTTGGGCCCTAATTTTGGAA

TACACAATTGGTGGGTCAGCTGTTGCACGTGGCATAACCCCCAACTTGGCCTTGGCTTTT

GGAGGACCAGACAGCTTGCCTTTCTTTCTAGCACGTGCTCAAATTCCAGGGCTTGATATT

GTTGTTGATCCATGTGCTGCAATTCTAGTCTTTATAGTTACAGGCCTGCTATGTGTGGGA

ATAAAGGAGAGTTCATTCGTACAAGCTGTTGTCACCGTTTTAAATGTTTGTGTCATGTTA

TTTGTCGTTATAGCCGGTGGATATATTGGCTTTAAGACTGGATGGGTGGGATATAGTGTT

TCAAGCGGATACTTTCCTTATGGGATTAATGGAATGCTTGCTGGATCAGCAACGGTTTTC

TTTGCATACATAGGTTTTGATTCAGTTGCCAGCACAGCTGAGGAGGTGAAAAATCCTCAG

CGTGATTTGCCATGGGGTATAGCGTCATCTTTGTCTATTTGCTGTTTCCTCTATATGATG

GTTTCTGTTGTCGTGGTTGGCCTGGTACCATACTTCGCTATGGATCCTGATACCCCAATT

TCATCTGCATTTGCTAAACATGGGATGCAATGGGCAATGTACATTGTCACAGCTGGTGCT

GTTCTTGCTCTGTGCTCAACTTTGATGGGTTCACTTCTGCCACAGCCTAGAATATTAATG

GCAATGTCTAGAGATGGATTGTTGCCACCTTTTTTCGCGGATGTGAACAAACGCACTCAA

GTTCCTGTGAAGAGCACAATTTTGACCGGAATCTGTGCAGCTGCCCTAGCTTTTTTCATG

GATGTTTCACAGTTGGCAGGAATGGTTAGCGTTGGTACACTCCTTGCATTCACCATCGTT

GCAATTTCAATCTTGATCCTTCGATATGTCCCACCAAATGAGGTGCCTCTGCCATCATCA

CTCCAAGAGTCGATTGATTCAGTATCATTTCGTTACAGTATTCAGGAACAAGATGGGGGA

AGTTCAAAAAATCTTGATGGAAAAATCAATAAAGATGGTCTGGAGGCATCATTTGAATAT

CCTCTTATTGTCAAGGAAGATGAATTAGACAAAATGAATGAACAAAAACGGCGAAAGCGT

GCAGCATGGAGCATAGCATTTGTGTGCATTGGAGTTCTCATTCTCACTTCTTCAGCATCA

GTTACATCCTTGCCTGTGTAA

>TRINITY_DN10231_c0_g1|m.369 TRINITY_DN10231_c0_g1|g.369 ORF TRINITY_DN10231_c0_g1|g.369 TRINITY_DN10231_c0_g1|m.369 type:complete len:100 (-) TRINITY_DN10231_c0_g1:1411-1710(-)

ATGTCTGTTTCTTATTTGTATTTCAGTTTGGCATGGTATTCAGTATGCGGTTTTGGTGGC

TTTATCCTTCTGGGAGGTCTGGTTGTACTTTCCTGGATTGAGCAAGATGATGGTCGGCAC

AGTTTTGGACACGCTGGAGGTACCCTTAGTCGTTTATTCCATCATAACTCCTTGTTCTTA

TTGGTAGAGCTTGCAAAATTGATTACTTCACAAGTCACATTAATAACAATGTGGGTTCTG

TTTCTTCTCCAGGATTTATTTGCCCCTTTGTCCCCCTTCTGCCAATTTGTTGCATCCTAA

>TRINITY_DN10231_c0_g2|m.370 TRINITY_DN10231_c0_g2|g.370 ORF TRINITY_DN10231_c0_g2|g.370 TRINITY_DN10231_c0_g2|m.370 type:complete len:637 (-) TRINITY_DN10231_c0_g2:271-2181(-)

ATGGGATTTGAGGAGGGGAGCAGTAGCAAGGTGAGAGGATGGGGGGTTCAGAGCTTGACG

AGGAGAAAACAGGTGGATTCTGATCGAGCGAATTCGGAGAGAGGACATCATCAGCTTGCA

AAGGAATTGTCTTTTCTTCAGCTTATTGCTATTGGTGTGGGTTCAACCATAGGTGCTGGA

GTCTATATTCTTGTAGGAACTGTTGCAAGAGAGCATACTGGTCCAGCTCTGACTATTTCC

TTTCTGATAGCTGGAATAGCCGCAGCTCTTTCAGCATTCTGCTATGCAGAGCTTGCAAGT

CGTTGCCCCTCTGCAGGGAGTGCCTATCATTATTCATACATTTGCCTTGGAGAAGGTGTT

GCTTGGGTAATTGGTTGGGCTCTGATACTAGAATATACCCTTGGTGGTTCAGCTGTTGCG

CGAGGCATTTCTCCGAACTTGGCCTTATTCTTTGGGGGACCAGATAGTTTGCCTCCTTTT

CTAGCCCGTGTTGAAATTCCAGGTTTAGATGTTGTAGTTGACCCATGTGCTGCAATTCTT

GTGTTGATTGTCACTGCGCTATTATGCGTGGGAATAAAAGAGAGTTCATTTGTACAATCC

ATCACTACAACTGCCAATGTCATTGTCATGCTATTTGTCATCATAGCTGGTGGATATCTT

GGTTTTCAAACTGGTTGGGTTGGATATTCTGTTTCTAGTGGATACTTCCCCTATGGCATG

AATGGTGTGCTTGCTGGATCAGCAACTGTTTTCTTTGCATACATAGGTTTTGATTCAGTT

GCAAGCACTGCAGAGGAGGTAAAGAACCCGCAGCGTGATTTGCCATGGGGTATTGGATTG

TCTTTGTCTATATGTTGCTTGTTGTACATGATGGTTTCCGTTGTTATTGTTGGCCTGGTA

CCCTACTTTACAATGGATCCAGACACCCCGATTTCATCTGCATTTGCCAAAAATGGGATG

CAGTGGGCAGTGTATGTAGTAACCACCGGAGCTGTTCTTGCTCTTTGTTCGACCTTGATG

GGCTCAATTCTCCCACAGCCTCGAATACTGTTGGCAATGTCTCGAGATGGATTGTTGCCG

CCTTTCTTCTCAAAAGTTAACAGCCGTACTCAAGTACCAGTTAATAGCACTGTCTTAACT

GGGATAGGTGCAGCCATCCTAGCATTCTTCATGGATGTTTCAGAATTGGCAGGGATGGTC

AGTGTTGGCACACTTCTTGCATTCACAATGGTTGCAATTTCTATTTTGATACTAAGATAT

GTTCCTCCGAGTGAAGTTCCTTTGCCATCAGCATTTCATGAATCCATTAACTCAGTGTCC

TTGCGGTATAGTTCTCAAGAAATAGATGTGGGAAGTGAGAAAGCTCTTGTTGCAGAAAAC

AAGGAAAATAGCCAACATGCTCTTGATTTGGAACCATCATCTGAGTATCCTCTTATTGTG

AAGGAAGATGTTCAAGATAAAACAAATGAGCAAGAAAGGAGAAAGAAAGCAGCATGGAGC

ATAGTATCTGTGTGTGTTGGAGTTCTCATCCTCACTTCTTCAGCTTCAGCCACATTTTTG

TCATCACCTGTACAGTACTTTGCTTGCATTGTTGGTGGCTTGCTTCTCCTGATTGGTCTA

TTTGTACTGTCCTGGATCAACCAAGACGAAGCCAGGCACAGTTTCGGACATACTGGAGGA

TTTATTTGCCCCTTTGTTCCGCTTCTGCCTGTTTGCTGCATCCTCATTAACGCATACTTG

CTCATAAATCTTGGTGCAAGTACTTGGATGAAGGTTTCAGTATGGCTAGTGATCGGGGTA

TTTGTGTATATTTTCTATGGTCGAACCCACAGCTCATTGACGGACGTGATCTATGTGCCC

TCAGCCCATGTAGATGAGATTTATAGAACTTCGTTGCATAGTTTAGCCTAA

>TRINITY_DN10235_c0_g1|m.372 TRINITY_DN10235_c0_g1|g.372 ORF TRINITY_DN10235_c0_g1|g.372 TRINITY_DN10235_c0_g1|m.372 type:complete len:608 (+) TRINITY_DN10235_c0_g1:137-1960(+)

ATGGCCAGCAGCAGAGAGCTCTATAGGGACCCGAACGAGCCCGTTGAGGTCCGGGTCCGA

GATCTGCTTGGCCGCATGACCCTCGCGGAGAAGGCGGCCCAGATGACCCAGATCGACCGG

AGCGTCACCTCCCCCGCGGCCCTCACCAGGCTCTCCATCGGCAGCGTCCTCAGCGGCGGC

GGCAGCGCCCCTTGGGAGCGGGCCTCCGCCGAGGAGTGGGCCCACATGGTGGACCAGATG

CAGCGGTGGGCCCTCTCCTCCCGCCTCGGGATCCCCATCATCTACGGCTCCGACGCCGTC

CACGGCCACAACAACCTCTACGGCGCCACCATTTTCCCCCACAACGTCGGCCTCGGCGCT

ACAAGAGATGGGGAGTTAGCTCGTAGGATCGGAGTGGCGACGGCGCTGGAAGTGAGGGCC

AGCGGAATTCAATACACATTTGCCCCTTGCGTTGCGGTGTGTAGGGACCCGAGATGGGGA

AGATGCTACGAGAGCTATAGCGAGGACCCGGAAATAGTAAGGATGATGACGCTGATTATT

TCTGGCTTGCAAGGAACCCCGCCTCAAGGTCACCCTGCAGGATACCCTTTCCTTGCTGGA

AGTAAGAATGTGATTGCCTGTGCCAAGCATTTTGTTGGAGATGGAGGTACTGACAAAGGG

CTGAATGAGGGAAATACTATCTGTAGTTATGAGGAGTTGGAAAGGATTCATATGAGACCT

TATCTTGACTGCCTTGCTCAGGGTGTTTGTACTGTTATGGCTTCCTATTCCAGCTGGAAC

GGGAGACCATTGCATTCCGACCGTTATCTCATAACAGATATCCTGAAGAACAAGTTGGGT

TTTAAGGGTTTTGTAATATCAGATTGGGAAGGAATAGATAGGCTATCTAAGCCACATGGA

TCAAACTATCGTTATTGCATTTCTGCATCAATAAATGCTGGAATTGACATGATAATGGTG

CCTCACAGGTATGACAATCTCCTAGAAGATTTGATTTATCTGGTGGAATCAGGGGAGATA

CCCATATCCAGGATTGACGATGCTGTAGAGCGTATCTTAAGGGTGAAATTTATTGCTGGA

CTGTTTGAAAAGCCTTTTTCAGATCGATCTTTATTGAGTGTAGTTGGTTGCAAGGAGCAC

CGGGACCTAGCACGTGAAGCGGTTCGGAAGTCTTTGGTTTTGTTAAAAAATGGGAAGGAT

CCTACAAAAGCATTTCTACCTCTAGATAAAAATGCTAAAAGAATTCTTGTTGCTGGAAAA

CATGCCGATGATATTGGATATCAATGTGGGGGATGGACTATAATGTGGTATGGAGGCAGC

GGAAGAATAACGATTGGCACTTCTATTTTGGAGGGCGTTAAGGAGGCTGTAGGAGAAAGT

ACAGAAGTGGTATATGAGGAACGTCCATCAGCATCAACCATTGCAAACCAGGACTTCTCA

TTTGCAGTAGTCGTCGTGGGAGAGACTGCCTATGCAGAATATTTGGGAGACAGATCTCAA

CTCGATATCCCATTCAATGGTTCCGAGTTGATTAGTCTGGTAGCTAGCAAAATTCCTACA

CTAGTCATTACGATATCAGGAAGGCCTCTGGTTCTGGAACCAGAGATACTGGAGAAAACA

GAAGCCTTGGTTGCTGCTTGGCTACCAGGCAGCGAGGGAGGTGGGATCGCTGATGTCCTA

TTCGGAGATTATGACTTTGAGGGTCTGCTCCCAGTGACTTGGTTCAGATCAACTTATCAG

TTGCCACTGAATGCAGGACACCCATCCTATGACCCTCTATTCCCCCTTGGTTTCGGATTG

AAAATGGGTCTGAGCAGCAAATAA

>TRINITY_DN10235_c1_g1|m.374 TRINITY_DN10235_c1_g1|g.374 ORF TRINITY_DN10235_c1_g1|g.374 TRINITY_DN10235_c1_g1|m.374 type:5prime_partial len:531 (+) TRINITY_DN10235_c1_g1:2-1594(+)

TCCGAGCCTCTATTCCCATACTCGAGCACTATATTCTCTCTTGACTCTCTTGATCCGATC

GCAGCAACATCAGCAGCAGCGATGTCGATCGCCATCCCTCACCGGCAGCTGTTCATCGAC

GGCGAGTGGCGGCCGCCCGCCCTCGGCAAGCGGATCCCAGTCGTCAACCCCGCCACCGAG

GCCACCATCGGCGATATACCGGCGGCGACCGAGGACGACGTCAATCTGGCGGTGACGGCG

GCGAAGAGGGCGCTGAAGAGGAACGGGGGGAAGGACTGGGCTCGCGCTCCGGGTGCCGTC

CGTGCCAAATACCTTCGAGCAATCGCAGCCAAAATAACAGAAAGAAAGTCTGAGCTGGCT

AAACTTGAGACACTGGATAGTGGGAAGCCTCTGGATGAAGCAGCATGGGACATAGATGAT

GTTGCTGGCTGCTTTGAGTACTATGCAGATCTTGCAGAGGCCTTAGATAAAAAGCAAAGG

ACTCCAGTTTCTCTTCCCATGGAAACATTTAAGTCCTATGTCCTTCGAGAACCCATTGGA

GTAGTTGGATTGATTACTCCCTGGAACTATCCTTTACTAATGGCTACATGGAAAGTTGCT

CCTGCCTTGGCTGCTGGATGTGCAGCTGTACTAAAGCCTTCGGAACTTTCATCAGTGACT

TGCTTAGAACTTGCTGAAGTGTGTAAGGAAGTAGGACTACCTCCTGGTGTTTTGAATATA

CTAACAGGACTAGGCCCGGAAGCAGGTGGGCCTATAACATCCCATCCTGATGTTGACAAG

ATTGCTTTCACTGGAAGTACAGAAACTGGGAGAAGGATTATGTCTGCTGCTGCACAATTG

GTGAAGCCGGTTTCATTGGAACTTGGTGGTAAAAGTCCCATTCTTGTATTTGAAGATGTA

GACATTGAGAAAGCTGTTGAGTGGACTGTTTTTGGGTGCTTTTGGACAAATGGGCAAATA

TGCAGTGCGACTTCTCGTCTTCTTGTACATGAGAGCATTGCAAAAGAATTCTTGGAGAGA

CTTGTTGCTTGGGCTAAAAACATCAAGGTGTCGGATCCTCTGGAGGAAGGTTGCAGGCTT

GGTCCTGTTGTCAGTGCTGGGCAGTATGAGAAGATAAAGAAATTCATTTCAACAGCAAAG

GCTGAAGGTGCAACTATATTGTATGGAGGTGGACGCCCGAAGCATCTAGAGAAAGGGTAC

TTCATTGAACCAACAATCGTAACAGATGTTGATACCTCCATGCAAATTTGGAGGGAGGAG

GTTTTCGGGCCAGTTCTCTGTGTTAAAGTATTTAAGTCTGAAGATGAAGCTATAGAACTT

GCAAATGATACACAGTATGGATTGGGAGGTGCTGTGATTTCAAATGATCCGGAGCGCTGC

AAGCGTATAACTGAGGCAGTTCAAGCTGGAATCATGTGGGTAAACTGCTCACAGCCGTGC

TTTTGCCAAGCCCCCTGGGGAGGAAACAAGCGTAGTGGTTTCGGACGAGAACTTGGAGAA

TGGGGGCTTGACAACTATCTGAGTGTAAAGCAGGTGACTGAATATATATCGAATGAGCCA

TGGGGATGGTATTGCTCTCCTTCCAAGCTGTGA

>TRINITY_DN10236_c0_g1|m.377 TRINITY_DN10236_c0_g1|g.377 ORF TRINITY_DN10236_c0_g1|g.377 TRINITY_DN10236_c0_g1|m.377 type:complete len:557 (-) TRINITY_DN10236_c0_g1:323-1993(-)

ATGGACAATTCCCTCTCAAAGCCCATTCTACTGTTCCTACTGATAATTCTTCTCACACAT

TTATCTAACTATGCAACTGAAGCCAATGCAAGTGCCATTACTCAGCCAAACCACCACCGC

TACCATGGGTTCATGGCTGAAGTGATAGGAAGAGAAGAACTGAGAAGCAGGGGAAGAAGC

AGATGCAGAGGCAGAGGAAGGAGAGGAGGAAGATCACCTTCCTGTGATCCTTTATTCCGA

TACCTCTTCGGACGCTGTGGCAGATGGCCTTTTTCAACCTATCCTTCCCCTGACAACCCG

TTTATCCCCACTCGCCCTCCCCATTCCCCTCCCCCCTTCCCACTCCCGCCAATACCTCCC

ACTGCTCCTCCTCTGGTTCCCTCGCCGCCACCCATTGTCCAATTGTCACCACCACCTGTG

CAATTCCTGCCACCACTTGTCCCCTCACCTCCACCTCCCATGACATTGTCTCCGCCCCTT

CCACCGCCTCTCATCCCATCTCCTCCACCACCAATCCCTCCATCTCCACCACCACCTCTT

GTACCATCGCCACCCCCTCCGTCACCATCACCTCCACCACCTCTTGTGCCTTCACCACTC

CCACCATCACCCTCACCACCACCGCCTCTTGTGCCTTCACCACGTCCTCCATCACCGCCA

CCGCCCCTTGTGCTTTCCCCTCCACCAACACTATCCCCACCTGTATTTCCACCACCTCTC

GTCCCTTCACCGCCGCCAGAAATAAGTACACCGCCACCTTTCTTTCCCATATTCCCTCCG

CCTCTCGTTCCATCTCCTCCACCGAGAACAGATTTCTTTTCTCCTCCGATCATACCTCTA

CCAATTGACAATTCACCACCACCCATAGATCACTTCTTTCCTCCGTTCTTCTTTCCACCA

CCGTCTTTTCCAGATCAACCATTGGTGTCAATGCCACCAAGCACTCCTGCTGACTCCCCA

CCATCGTTTCTTCCTCCGCCCACTAACTTCACACCTCCAGCTGGTGATCAGCCAGAGCTC

CCACTGCCACCAACATTGGTTCCGGTTGATAATCCACCACCAGAGAGCATGGACCAACCA

TCATTTCCACTGCCACCTGTAGAACCAGTTGATAGCAGTCCTTCACTCCCTCCGCCTTTG

TTATTTCCGCCACCCATTGACTTCACACCTCCAGCAGATGGTGATCAGCCACTGCTCCCT

TTGCCGCCACCTTTGGCTTCATCTAATAACCCTCCACCAGAGAGCACTGACCAACCATCG

TTTCCACTACCACCTCTAGTGCTAGTTGACAGCAGTCCTCCTTCACTTCTTCCACCATCA

TTTCTTCCACCTCCAGTTGACTTCACACCTGTAGATGGTAATCAGCCAGTTCTCACATTG

CCACCATTGGTTCCGTCAGATAATCCTCCAACAGAGAGCACAGACCAACCATCACTTCCA

CTGCCACCTCTAGTGCCAATCGACAGCAGTCCTCCATCACTCCCGTTGCCACCTCAGTTT

CCAGTTGATAGCACACCTCCGTTGTTGCTGCCTCCACCAATGGTGCCTATTGTCGACCAT

TTGCCATTGCCACTTCCAGTAGAACCAGTTCAACCAGAAATGCCTCCTTTGCTTCCTTTT

GATGCTCCTCCATCCTTTCTTCCTCCTCCAACCACCCCCATCAGAAATTGA

>TRINITY_DN10236_c0_g1|m.378 TRINITY_DN10236_c0_g1|g.378 ORF TRINITY_DN10236_c0_g1|g.378 TRINITY_DN10236_c0_g1|m.378 type:complete len:196 (+) TRINITY_DN10236_c0_g1:1128-1715(+)

ATGGGTGGTGGTGAATTGTCAATTGGTAGAGGTATGATCGGAGGAGAAAAGAAATCTGTT

CTCGGTGGAGGAGATGGAACGAGAGGCGGAGGGAATATGGGAAAGAAAGGTGGCGGTGTA

CTTATTTCTGGCGGCGGTGAAGGGACGAGAGGTGGTGGAAATACAGGTGGGGATAGTGTT

GGTGGAGGGGAAAGCACAAGGGGCGGTGGCGGTGATGGAGGACGTGGTGAAGGCACAAGA

GGCGGTGGTGGTGAGGGTGATGGTGGGAGTGGTGAAGGCACAAGAGGTGGTGGAGGTGAT

GGTGACGGAGGGGGTGGCGATGGTACAAGAGGTGGTGGTGGAGATGGAGGGATTGGTGGT

GGAGGAGATGGGATGAGAGGCGGTGGAAGGGGCGGAGACAATGTCATGGGAGGTGGAGGT

GAGGGGACAAGTGGTGGCAGGAATTGCACAGGTGGTGGTGACAATTGGACAATGGGTGGC

GGCGAGGGAACCAGAGGAGGAGCAGTGGGAGGTATTGGCGGGAGTGGGAAGGGGGGAGGG

GAATGGGGAGGGCGAGTGGGGATAAACGGGTTGTCAGGGGAAGGATAG

>TRINITY_DN102380_c0_g1|m.380 TRINITY_DN102380_c0_g1|g.380 ORF TRINITY_DN102380_c0_g1|g.380 TRINITY_DN102380_c0_g1|m.380 type:complete len:285 (+) TRINITY_DN102380_c0_g1:136-990(+)

ATGGAGGCCATCAAGAAGAAGATGCAGGCGATGAAGCTCGAGAAGGACAATGCCATCGAC

CGAGCTGAAATCGCCGAGCAGAAGGCCCGAGATGCCAACCTCAGAGCTGAGAAGACTGAG

GAAGAAGTGCGCGCCCTGCAGAAGAAGATTCAGCAAATTGAGAACGAGTTGGACCAAGTC

CAGGAGCAATTCACTCAGGCCAACACCAAGTTGGAAGAGAAAGAAAAAGCCCTGCAAACC

GCCGAAGGCGATGTTGCTGCTCTCAATCGTCGTATCCAACTCATTGAAGAGGATCTCGAG

CGATCTGAAGAACGTCTCAAGGTTGCCACTGCCAAGCTCGAGGAGGCTTCCCAAGCTGCC

GATGAATCCGAACGAATGCGCAAGATGCTTGAGCACCGAAGCATCACCGATGAGGAGCGC

ATGGATGGCCTCGAGAACCAGCTCAAGGAGGCTCGTATGATGGCTGAGGATGCTGACCGA

AAGTACGATGAGGTCGCCCGTAAATTGGCCATGGTTGAGGCTGATCTGGAGCGTGCTGAG

GAGCGTGCCGAGACCGGCGAATCCAAAATCGTCGAGCTCGAGGAGGAGTTGCGTGTGGTT

GGCAACAACTTGAAATCCCTGGAAGTCAGCGAAGAGAAGGCCCAACAACGTGAGGAAGCC

TACGAACAACAGATCCGAATCTCCACCGCCAAGCTCAAGGAGGCTGAGGCTCGCGCTGAA

TTCGCTGAACGTTCGGTCCAAAAGCTGCAAAAGGAAGTCGACCGACTGGAAGACGAGTTG

GTTCACGAAAAGGAAAAATACAAGTCAATCTCGGACGAGTTGGACCAGACCTTTGCCGAA

CTCACTGGTTACTAA

>TRINITY_DN10238_c0_g1|m.383 TRINITY_DN10238_c0_g1|g.383 ORF TRINITY_DN10238_c0_g1|g.383 TRINITY_DN10238_c0_g1|m.383 type:complete len:512 (-) TRINITY_DN10238_c0_g1:280-1815(-)

ATGGTCGCAGCCAACCCACTTTTTGCTGGGGGAGGATACTACAAAAAGTTCAAGGCGACA

ATCATCAATGACATATGGCCGGAAGTCCTCTTCTTCACACTCGTTGGAGCCATGGTGACA

CTGGTGTCGGAGCTGACCGACAGGAAATTGTCTCTCTCCAACCAGATGCTCACTGTACTT

GGTACAGTTCTCGGTTTGGTTATATCGTTCCGAACGTCGACCGCATACGAACGCTTCTCT

GAGGGTAGAAGGCTTTGGACTTCCATCGCTATTGGCTCTAGGAACCTTGCTCAGATTATT

TGGATCCACGTTCCAAACGATCGTGTGGACAAGGCCACAGGAGAGAAGAAGCCGATACTT

CCGATCATTATTGAGAAGAAGAGTATGATTAACCTGGTACAAGCGTACTCGGTTTCTGTG

AAGCATCTTCTCCGTGGCGAAGGTGGTGTGTACTATGCTGACCTGTACCCACTCATCTCG

TTCTTGCCCCGTTTCACCAAGCACGTCGACCATGCCGAGGAGCACGACATGCTCCCTCTC

TGGAAAGCGTCTGCTATGGATCACGAGGAGCACCGGAAGCACGTTCGCGAGAAGACTTTC

GCTACTCTGTCCAACAGCAGTATTCACCGAACTCACTCTGACCCCGGCCCCGACCCCAAG

GAGGCTATGAGCGAGAAGGACGAATCTTGGATGAACTCTATCCGTCGCAGCAAGAAGAAG

AAATTTGACCCTGAGAGTGCTCTCCCCGTCCTGCCTAGCGAGCGGCCACTTCGTCCTGCA

AGGAATCCGCCGGAAGCCACACTATACGACTACTTCCCCTTCTTGCGTATCTTCAAGTCC

ATTTTGAAGCCATGCATCCGTCGTTTCAGGGCTCCTGACCAGAGGAGCCACTCACGCACC

ATCACTGGCAGGAAGATCAGACCTGAGGCCGCGGATTCGAACGTTCCAATGGAAATCACC

CTCTTCCTGACCTCCTACTTCGCATCACTCATGAGGCAGGGATTACTTACACCAGCGTCG

GCAACTGCCATGAACAACGCTATTACCTCCCTTCAAGATAATGTAAATAACTTGGAGAGG

ATTAAGACTACGCCTTTGCCATTCGCCTATCAAGCTCACTTGAGGATGTCGCTTTGGTTA

TATCTGTTCTTCCTTCCGTTCCAAATCTGGAACGCTTTCCACTGGTACACGATTCCTGCA

ACTACCTTTGCTTCATTCCTCCTTCTGGGTTTCTTGGAGATCGGTCAGGAGATTGAGAAC

CCCTTCAACTATGATTTGAATGATCTGGATCTTGATCACTTCTGCCTTGCTATCCAACGC

GAGCTGCACGAGGTCACTGCTCACACCTGCCCTGAGCCCGATGCTTACATCTTTACAGAG

TGGAACCAGCCGTTTGCGCCCGCTGACCGTCGCAATGCCAAGGAGATCATTGATGACGTC

AACCATGATTACCACGGCGCCGAGACGGGCATGCACAGCATACGCTCGACTCTGCTCAAG

AGTTGGCGTGATGTCGATGAGTTGACTCGTCACTAG

>TRINITY_DN10239_c0_g1|m.385 TRINITY_DN10239_c0_g1|g.385 ORF TRINITY_DN10239_c0_g1|g.385 TRINITY_DN10239_c0_g1|m.385 type:5prime_partial len:193 (-) TRINITY_DN10239_c0_g1:169-747(-)

GAGACTGGTATCCTCAAGCCTGGTATGGTTGTCACCTTTGGACCCACTGGGCTGACAACT

GAAGTTAAGTCTGTTGAGATGCACCATGAAGCTCTGCAGGAGGCTCTCCCCGGAGACAAT

GTTGGCTTCAACGTCAAGAATGTTGCCGTGAAGGATCTCAAGCGTGGTTTTGTTGCCTCC

AACTCCAAGGATGATCCTGCAAAGGAGGCTGCCAACTTCACTTCTCAGGTTATCATCATG

AACCATCCTGGTCAGATCGGCAATGGATATGCTCCAGTGCTTGACTGCCACACCTGCCAC

ATTGCGGTGAAGTTCTCTGAGATCCTCACCAAGATCGACAGACGATCTGGCAAGGAGCTT

GAGAAGGAGCCCAAGTTCTTGAAGAACGGTGATGCTGGTTTCGTTAAGATGATTCCCACC

AAGCCCATGGTCGTTGAGACTTTCTCTGAGTACCCACCACTCGGACGTTTTGCTGTGAGG

GACATGAGGCAGACTGTTGCTGTCGGAGTTATCAAGAGTGTGGAGAAGAAGGATCCCACT

GGTGCCAAGATCACCAAGGCCGCTGCCAAGAAGAAGTGA

>TRINITY_DN10239_c0_g3|m.386 TRINITY_DN10239_c0_g3|g.386 ORF TRINITY_DN10239_c0_g3|g.386 TRINITY_DN10239_c0_g3|m.386 type:internal len:103 (-) TRINITY_DN10239_c0_g3:2-307(-)

GTTGCCTCGAACTCCAAGGATGATCCTGCTAAGGAGGCAGCCAACTTCACCTCCCAGGTC

ATCATCATGAACCACCCTGGTCAGATTGGAAACGGCTATGCTCCTGTCCTAGATTGCCAC

ACTTGCCACATTGCAGTCAAGTTCTCTGAGATTCTGACCAAGATTGACAGGCGATCTGGC

AAGGAGCTTGAGAAGGAGCCCAAGTTCCTGAAGAACGGAGATGCTGGATTTGTTAAGATG

ATTCCGACCAAGCCCATGGTTGTGGAGACTTTCTCTGAGTACCCGCCATTGGGTAGGTTT

GCTGTC

>TRINITY_DN1023_c0_g1|m.387 TRINITY_DN1023_c0_g1|g.387 ORF TRINITY_DN1023_c0_g1|g.387 TRINITY_DN1023_c0_g1|m.387 type:complete len:132 (+) TRINITY_DN1023_c0_g1:286-681(+)

ATGTCTTGGCAAACTTACGTGGATGAGCACCTAATGTGCGACATCGAGGGCCATTTCCTC

ACCTCCGCCGCCATCACCGGCCACGACGGCAGCGTCTGGGCCCAGAGCGCCTCTTTCCCT

CAGTTTAAGCCTGAGGAGATCAATGGTATTATGAATGATTTTGCTGAACCAGGGTCTCTT

GCTCCTACTGGCTTATTTCTTGGGTCAACAAAGTATATGGTAATCCAAGGAGAGCCTGGA

GCTGTTATTCGTGGGAAGAAGGGATCGGGTGGAATCACTATTAAGAAGACAGGTCAGGCT

CTGATCATTGGCATCTATGATGAGCCCATGACACCAGGGCAGTGCAACATGGTTGTGGAG

AGGCTCGGGGACTACCTCATCGATCAGGGCATGTAG

>TRINITY_DN1023_c0_g2|m.388 TRINITY_DN1023_c0_g2|g.388 ORF TRINITY_DN1023_c0_g2|g.388 TRINITY_DN1023_c0_g2|m.388 type:complete len:132 (+) TRINITY_DN1023_c0_g2:169-564(+)

ATGTCGTGGCAAGCGTACGTCGACGATCACCTGATGTGCGAGATCGACGGCCAGCACCTC

ACCGCCGCCGCCATCATCGGCCACGACGGCAGCGTCTGGGCTCAGAGCGAGAACTTCCCC

CAATTGAAGCCTGAAGAAGTTACTGGAATCATAAAGGACTTTGAGGAACCCGGATCTCTT

GCACCCACTGGTTTATACCTTGGCGGTACAAAGTATATGGTTATCCAAGGTGAACCAGGG

GCTGTTATTCGAGGAAAGAAGGGTTCTGGCGGTGTTACTATCAAGAAAACAAATCTGGCT

TTGATCGTTGGTATTTATGATGAGCCAATGACTCCTGGACAGTGCAACATGGTTGTTGAG

AGGCTCGGTGATTACCTCATTGAACAGAACTTTTAA

>TRINITY_DN1023_c1_g1|m.389 TRINITY_DN1023_c1_g1|g.389 ORF TRINITY_DN1023_c1_g1|g.389 TRINITY_DN1023_c1_g1|m.389 type:complete len:277 (+) TRINITY_DN1023_c1_g1:482-1312(+)

ATGGAAGCAGAGGCGAGCTCGAGAGATCTGCTGCAGCGGTTCGCCGGAAGCACGACCCTC

TCCGACCGTCCTCCGCCGGCTCCGGCGGCGGCCAGGGCCGTGGACGACGCTGACCTCAGC

CTCGGCCTCTCATTGGGCGGTTGCTTCAGCTCCAACCCGAGAGAGAACACCAAGCTCATC

CGATCCTCCTCCATCTCGTCGTTCCCTTTTCTTGACGCTATCAAAGATGAGAACTTTACG

GTGCCGCCGGCGCCGCTGCACCGCACGTGCTCACTGCCCGTGCCGGGGGCAGACGAGGAC

TACAGGAAGAGGAAGGAGCTGCAAACGTTGAAGAGGTTGGAGGCCAAGAGGAAGAGATCG

GAGAAGAGGAATAGTTTGAAGAGCGACGATGGCGGAGGATGCACGTGGCAAGCGGGCAGC

CGGGCAGGTGCTGCCCGGCTGCCGCCATTATTGCAGGGATCGATTGGCTCGGCGGGGAGC

AGCTCGTCCATTGGTGGATCATTGCAAGAGACCAAAAGCTCCTCAATGGCCAACCACACC

AACCACCGATCATCTGCAGACGATAACACATTGAAGAAGATTGAAAGGAAAGGCAGCGGG

ATGAGCACAATGGAGGAGATGCCATGCGTGTCGACAAGAGGGAACGGCCCCAACGGGAAG

AGGATCGAAGGGTTCCTGTACAAGTACCAAAAAGGGGAAGAGGTGAGGATAGTATGCGTC

TGCCATGGCAGCTTCCTCACTCCAGCCGAGTTCGTCAGTCACGCTGGCGGGGGCGATGTC

GATCATCCTCTCAGGCACATTGTTGTCAGCCCCCCATCTCCCTTCTTGTAA

>TRINITY_DN1023_c1_g1|m.390 TRINITY_DN1023_c1_g1|g.390 ORF TRINITY_DN1023_c1_g1|g.390 TRINITY_DN1023_c1_g1|m.390 type:complete len:139 (-) TRINITY_DN1023_c1_g1:1031-1447(-)

ATGCTGATACCCACAGATTCGAGAGAGATGCTCAATAGCCTATACAAGCAACATAAGCAA

TGTGGAAATTACTTGATGAAAAGAATTTTAAACGACTCTCCGAATTCTTCATGGATATAT

TTCAAATGCCATGATTTACAAGAAGGGAGATGGGGGGCTGACAACAATGTGCCTGAGAGG

ATGATCGACATCGCCCCCGCCAGCGTGACTGACGAACTCGGCTGGAGTGAGGAAGCTGCC

ATGGCAGACGCATACTATCCTCACCTCTTCCCCTTTTTGGTACTTGTACAGGAACCCTTC

GATCCTCTTCCCGTTGGGGCCGTTCCCTCTTGTCGACACGCATGGCATCTCCTCCATTGT

GCTCATCCCGCTGCCTTTCCTTTCAATCTTCTTCAATGTGTTATCGTCTGCAGATGA

>TRINITY_DN10240_c0_g1|m.392 TRINITY_DN10240_c0_g1|g.392 ORF TRINITY_DN10240_c0_g1|g.392 TRINITY_DN10240_c0_g1|m.392 type:complete len:451 (+) TRINITY_DN10240_c0_g1:171-1523(+)

ATGAGGTCTCACGGTGCAAAACTCGCCCTTTTTCTCGCCCGGCGGTCTCTCCAATCCAAC

CTGTTCCGCCAATCGCAGTTCAAATCTTTGGAGCAGTCATCGCGGTGGATATGCAGCTCG

TCGTCTAGGGTTTTCTCGGACTCGAATTTGGATAAAGGAAAATGGAAGAGTGGGATTCAA

AGTGGTGTGTTTTATACTAATCTATATCCAAGGAGGCATTTTCATGGAACAAGGCCGGCG

CTAGCGAGAGATTACTATGATGTACTTGGAGTGAGCAAGAACGCAAGTGCCTCGGATATT

AAGAAAGCTTATTACGCGCTTGCAAAGAAGCTCCATCCTGATACAAATAAAGATGATGCT

GAGGCTGAAAGAAAATTCCAAGAAGTTTCTCGTGCTTATGAGGTTCTAAAGGATGAGGAA

AAGAGATCTCTTTATGACCAGGTTGGTCCTGATGCATTCGAACAGGCAGCTTCAGGAGGT

GGCCCTAGTGGGGGGCCATTTACTGGTGGTGGATTCAATCCATTTGAGGACATGTTTAAT

GGGTTTAGTGGGAATGGGAGCGATTTCTTCTCAAGCATGTTTACGAACAGGAATTTTGGC

GGACAAGATATCAAGGTAGCACTGGAAATATCATTTATGGAAGCAGTTCAAGGGTGCACA

AAAACTCTGACATTTGATTCTGCTGTACCTTGCGAAGCTTGTGGTGGCACTGGAGCCCCT

CCTGGGACTAGACCTGAAACATGTAAAAATTGTAGAGGCTCAGGAATGACCTACATGCAA

CAAGGTCCCTTTAGATTGCAGATATCCTGTACACACTGTAATGGAGAAGGAAAAATTACC

AAGAGTAAGTGCAAGTCATGCAGGGGTCAGAAAATTGTGAGAGGAACAAAATCAGTAAGA

CTAGATGTTATGCCTGGAGTTGACAATGATGACACCATAAAAGTATATAGAAATGGTGGA

GCAGATCCTGAAGGTGGCCAACCTGGTGATCTTTATGTTACTATAAAGGTTCGAGAGGAT

CCTGTATTTAGGAGAGAGCAGGCGAATATACATGTTGATGCCATCCTAAATGTCTCTCAG

GCAATCTTGGGCGGCACCATACAAGTCCCAACACTGACAGGGGATGTCGTCCTTAAGATA

AGACCGGGTACTCAACCAGGTCAGAAAGTTCTCTTGAAGGGGAAAGGAATAAGGTTGCGG

AACTCCTCAGTGTACGGCGACCAATTTGTTCATTTTAATGTGTCCATTCCTGCTAACTTG

ACCCAGAAGCAGCGCATGTTGATAGAGGAGTTTCAAAAAGAAGAGCAGGGGGAATACAAT

AAGGGCGCCAAAGCAGCAGGAGCTTCAGGATAG

>TRINITY_DN102414_c0_g1|m.393 TRINITY_DN102414_c0_g1|g.393 ORF TRINITY_DN102414_c0_g1|g.393 TRINITY_DN102414_c0_g1|m.393 type:complete len:433 (+) TRINITY_DN102414_c0_g1:94-1392(+)

ATGTTGCCCGTAATGAACCGGTTCGGACGCATCGCGCTAGCCGTGGTTGCAGTCCTGTTT

GTCTTCGTCCTGCTGCTGTCTTACCAGGCGCCCGACAGCTTCAGGGCCAGTCTACCGAGC

ACAGATTCGCTATGGTCGAAAGGAGGGAGCACCACCGAGGCCTCGGCGACACCAACACCT

ACACCCATTGAGGAAGAGGAAGAGGAGGAGGTCGACCCTAATAGGGACATTTCGTACGAC

TTGACCCGGCCGCCAACCGCCGGCTGCGAGGACCTTGTCAACGACCTTCAGCAGCGAATC

ATCCAGACCTACCAGAAGCGATTCAAGGGTATTCGCTACGCCAACATCTGGGGATACCTC

GAGACCGAGAACAAGGGAGATGCCGCCATTTGGTCCGCCCAACAGATCCTGCTCAGCATC

CTCGGCATTGAGACCATGGAAGCTTGCCGCTTCATGCACCAGGGATGTGACATGGAGAAG

TTCCGCAAGGGACTCGAGGAGCACCGCCCGCACTCGGCCATCATCATGGCTGGCGGCGGC

AACTTTAACGATTACTACTGGGAGGACCAACCGTCGCGCATGAAGATGATCTCAACCTTT

ACCAACGTCTCCATCCGCGCCTTTCCCCAGAGCATCTTTATGAACAACCCCGAGCGGATC

AACCTTACCCACGTCGCCTTCAAGAAGCACCACGACCTTCAGCTTGCGGCCCGTGACAAG

CCCAGCTACGATTGGCTTCTCGACAACTTTGGCCAGACCGATGGCATCGACAACGATCTC

ATTCCGGATATCGCCTTTATGTGGGGGAACCGATCAGACTTCCGAGTCAACACGCCCAAG

ACCCACGACATCCTCATTCTTGCACGAAAGGACGCCGAGATCTCTGCGGGCGACTCAGCC

ATGATCCCCTTTGGTGAAGGCCGCATCGACCTAGGCGGCGCCGTGGGCAACGTTACGTAC

CGCAAGGTGGACTGGAAGTTCACCGAGACGCCCGACATTGACAACAAGGACAACCGAGAG

CGCGGCAAGAACCAGCGAGCGTGGGCCAAGTCGATGGCCGGCTTCGACCTCCTCGGATCG

GCTCGCTTCGTCATCACGGATCGTCTACACGGCCACATCCTGTCGACCGTCATCGGCGTG

CCTCATGTGCTGATGGACAGCAAGCTGGGCAAGAACCTCAACTTCCACAACACGTGGACG

CGCGACTGCAAATGCACGCGCATCACCAAGAGCATCCACTCGGCGTTTGATGTGGCGCGC

ATGTTTTTCGAGCAAGAGCTGAACCAACCAAGTTCATGA

>TRINITY_DN10242_c0_g1|m.396 TRINITY_DN10242_c0_g1|g.396 ORF TRINITY_DN10242_c0_g1|g.396 TRINITY_DN10242_c0_g1|m.396 type:5prime_partial len:120 (+) TRINITY_DN10242_c0_g1:1-360(+)

AAAAAAGGAAAAAAAAAACTTTTTAATTTGTATAGGTCTCCGTCTGTTTCTGCTGCTGCT

TCTTCTCACTTCTCACTGCTCACTGCTGCTCATGGCTTTTTTTACTACTACTTCTTCTCA

TCGTCTTATCCTTCTCTTCTTTCTTGGGGTTTTGATACAGCATTTCTTTCTCTTTGGCCT

CTCCTATGCAACTGCAACCAATGTCTACATTGTATACATGGGGCAGAAAAAACCCCAAGC

TCATCACGACCTCCATCACGGAATCCTCTCCACCGCCCTCGGCAGCGAGGAAGCTGCGGT

TTCATCCATCCTGTACAGCTACAGCCGCGGGTTCTCGGGGTTTGCTGCTGTGCTCACTGA

>TRINITY_DN10242_c0_g1|m.395 TRINITY_DN10242_c0_g1|g.395 ORF TRINITY_DN10242_c0_g1|g.395 TRINITY_DN10242_c0_g1|m.395 type:5prime_partial len:806 (+) TRINITY_DN10242_c0_g1:2-2419(+)

AAAAAGGAAAAAAAAAACTTTTTAATTTGTATAGGTCTCCGTCTGTTTCTGCTGCTGCTT

CTTCTCACTTCTCACTGCTCACTGCTGCTCATGGCTTTTTTTACTACTACTTCTTCTCAT

CGTCTTATCCTTCTCTTCTTTCTTGGGGTTTTGATACAGCATTTCTTTCTCTTTGGCCTC

TCCTATGCAACTGCAACCAATGTCTACATTGTATACATGGGGCAGAAAAAACCCCAAGCT

CATCACGACCTCCATCACGGAATCCTCTCCACCGCCCTCGGCAGCGAGGAAGCTGCGGTT

TCATCCATCCTGTACAGCTACAGCCGCGGGTTCTCGGGGTTTGCTGCTGTGCTCACTGAA

TCCCAAGCTAATCACATTGCTGATTTCCCTGGAGTGGTTCGCGTCATTCCTAATAGGATT

CTTAGCCTGCACACCACCAGGAGTTGGGATTTCTTGCAGTTGAAGCTGAATCCTCGGCCG

TCGTCCGATGGGCATGGGCTCCTCTCGAGGAGCAGGTGGGGTGATGGGTCTATTATTGGT

GTTTTAGATACTGGAATCTGGCCTGAGTCTGCGAGTTTTGACGATCGTGGAATGGGGGAA

GTTCCGGCGAGATGGAAAGGAATATGCCAGGAAGGAGAGCAGTTTCATGCTTCCAAATGT

AACAGAAAAATAATTGGTGCACGCTGGTATGTCAAAGGCTACGAAGCTGAATTCGGAAGG

TTGAATACAAGTGAGATGACCGAGTTTCTGTCTGCTCGTGATGCAGCAGGGCATGGCACG

CATACATCATCTACTGCTGCTGGTGCTTTTGTAGGCGATGCAAGCTTTATGGGGCTTGCT

CATGGCACAGCAAGAGGAGGTGCTTCTAGTGCTAGGTTAGCAGTCTACAAAGTTTGTTGG

GCTACTGGCGGCTGCAGTTCTGCTGACATACTAGCTGCCTTTGATGATGCTATACATGAT

GGAGTAGATGTGCTTTCAGTGTCCCTGGGCTCACCACCTCCACTTGCTACTTATGTTGAT

GATGTTTTATCTATTGGATCCTTTCATGCGGTGGATAGAGGAATTACCGTAGTATGTTCT

GCAGGGAATTCTGGCCCTTACTCGCAAACAGTTATAAATACTGCCCCTTGGGTTGTTTCT

GTGGCAGCCAGCACTATTGATCGGACGTTCCTTACAGCAGTTACGCTTGGGAACAACCGA

ACCCTTGTGGGTCAATCGCTGTATGTTGGGAAGCATGTAGACAAATTCTATAAAATAGTT

TACGCTGAAGACATTGCTTCAAGTACTGCAGAGGCAGGTGATGCTAGAAGTTGTGATGAA

GGTTCTCTAAATGCTACTTTAGCAAGAGGAAATGTAATTCTCTGTTTCCAAACCCGAGGA

CAGAGGTTACCTGTAGTTGCTGCAAGAACAGTAAGTAGTGCTCGTGGTGCAGGTCTCATC

TTTGCTCAGTTCTTGACAAAGGATATTGCTCCAGTTTTCACGCTCCCCTGCATCCAGTTG

GACTTTGAAACTGGAACTTCTATTCTGACTTACATGGAGACCGCAAGAAGCCCAGTTATT

AAGTTCAGCCATACGAAGACAGTCATAGGGAGCGTGCTTTCCCCTGAAGTGGCTTACTTC

TCGTCACGGGGACCAAGCTCATTGTCTCCTTATGTTCTGAAGCCTGATATTGCTGCACCT

GGAGTGAACATATTAGCCTCATGGTCCCCAGCATCCTCGCTTTCAGCTGGCATGCCTCCA

CTTAACTTCAAGATTGAATCAGGAACCTCCATGTCCTGCCCTCATGTTTCAGCCATTGTT

GCGCTCATCAAGTCCATCCATCCTAATTGGAGTCCTGCCGCAATAAAATCTGCGCTGATC

ACAACAGCCTCCATAAGAGATGGATACAGTATGAGTTTAGTAGCTGAGGGAGCTCCCTAC

AAGCAAGCTGATCCGTTCGACTATGGAGGTGGGCACGTTAACCCAAATAGAGCGATAGAC

GCTGGTCTCATCTATGACATGAGCATGCCACAGTACACAGACTTTCTTTGTTCCATGGGA

TACAACAATTCTGCAGTGAGCTCGATGGCCGAACATCTCACTTTCTGCCACAGTAGTGCA

CCTGAATCACAGAAGGACCTCAATCTCCCATCCATCTCTATTCCCGAGCTAAGAAAGAAG

TTGAGTGTCGTAAGAACAGTAACAAACGTTGGTTTTGTTCCGTCAGTGTACACTGCTCGG

GTAGAAGCACCTCCTGGGGTGACCGTGAAGGTCAAGCCATTGGTCTTGTCATTCAATTCA

ACTGTCACGAGTTTGTCGTTCAAGGTGATATTCTCTTCGCGGTTTAGGGTGGAGGGAAGA

TACTTATTTGGTAGTTTGTCATGGGAAGATGGTGTTCATTCAGTGAGGATACCCATAGCT

GTCCGTCCTATGCATTGA

>TRINITY_DN10244_c0_g1|m.397 TRINITY_DN10244_c0_g1|g.397 ORF TRINITY_DN10244_c0_g1|g.397 TRINITY_DN10244_c0_g1|m.397 type:complete len:196 (-) TRINITY_DN10244_c0_g1:248-835(-)

ATGCAGTCCATTAAGTGCGTCGTCGTAGGAGATGGTGCTGTAGGCAAGACATGTCTGTTG

ATCTCATATACTACGAATGCTTTCCCAGGAGAATACATTCCTACCGTTTTCGACAACTAT

TCGGCGAATGTGATGGTAGACGGCAAGACTATCTCCCTTGGCTTGTGGGATACCGCCGGT

CAAGAGGATTACGACCGGCTTCGGCCCCTTTCATATCCCCAGACAGATGTATTCTTGATT

TGCTTCTCCCTCGTCAGCCCACCAAGTTATGAGAACGTAAGGACGAAGTGGTACCCTGAG

ATCTCTCACCATGCGCCATCAACCTCGATTGTCCTGGTTGGAACCAAGCTGGATCTGCGC

GAAGACCCTGCGACAATCGAGAAGCTGAGGGACCGCCGTATGGCTCCCATTCAATACTCA

CAAGGTGTCGCTATGGCGAAGGACATCGGCGCTGTCAAGTACTTGGAATGTTCTGCACTC

ACGCAAAAAGGGTTGAAGACCGTATTTGACGAAGCTATCCGCGCCGTCCTAAACCCCCCG

CCGCCCACCAAGAAGTCCAATGGGGGCAAGAAGTGCATCATTGCGTGA

>TRINITY_DN10245_c0_g1|m.398 TRINITY_DN10245_c0_g1|g.398 ORF TRINITY_DN10245_c0_g1|g.398 TRINITY_DN10245_c0_g1|m.398 type:complete len:226 (+) TRINITY_DN10245_c0_g1:199-876(+)

ATGGCGAGGGAGAAGATACAGATAAGGAAGATCGACAACGCGACGGCGAGGCAGGTGACG

TTCTCGAAGAGGAGGAGGGGGCTCTTCAAGAAGGCCGAGGAGCTGTCGATACTCTGCGAT

GCTGAGGTCGGGCTCATCATCTTCTCGGCTACTGGCAAGCTCTTTGAGTTCTCAAGCTCA

AGCATGAAGCAGATAATTGAGAAGCACAACATGCATTTGAAGAAAGCACTGAGACAAGAC

CAACCATCCCTTGACTTGAACCTAGAGAATGGCAACTATATCAGATTGAGCAAACAAGTG

GCCGAGACAAGCCAGCAACTAAGAAAGATGAAAGGTGAAGACATCCATGGATTAACTATA

GAGGAGCTGCAGAGTCTAGAGAAAACCCTTGAAGCTGGATTAAGCCGTGTTCTAGGAAGG

AAGAGTGAACAAATCATGGCACAGATCAGTGGGCTTCAACAGAAGGGATTGCAACTGATG

GAAGAGAATACGCGGTTGAGACAGCAAGTGGTAGACATATCAAGAGCAGGGAAACAGGTG

GTGACCGATTCAGAAAATGGCTTTGGCGAGGATGGTCAGTCCTCAGAATCAGTCACTAAC

GCCTCGCAGTCTGGTGCACCGCAAGACTATGATGACAGCTCTGACACTTCTCTCAAGTTA

GGTCTGCCATGGAAGTGA

>TRINITY_DN10248_c1_g1|m.399 TRINITY_DN10248_c1_g1|g.399 ORF TRINITY_DN10248_c1_g1|g.399 TRINITY_DN10248_c1_g1|m.399 type:complete len:432 (-) TRINITY_DN10248_c1_g1:273-1568(-)

ATGAAGTTGGTCGAGCAATCAAGAATCTGTCCACCCCCAGGCTCGGTCCCAGACTCCTCT

CTCCATCTCTCTCTGTTCGACGTTGTCTGGCTCGATTTCTCCGCAGTCAAGCGATGCTTC

TTCTATCGATTCCCTCACTCCACCTCTCACTTCATCAACTCCCACCTCCCCAACCTCAAG

CGCTCCCTCTCTCTCGCTCTCCAACACTTCTACCCCTTAGCCGGTAGAATCCGCCTCTCA

CCCGGCTCTGACGACCAGTTCGAGATACACTACGCCGACGGCGACTCCATCCCCTTGGCA

GTTGCCGAATGCTATGACGATCACTTCGCCGAGCTTGTCGGTAGCAGGCCTCACGATTAC

ACCAGACTCGAGCCTCTGCTCCCCGATTCGGACTCATTGCTAGCCATCCAGCTGACCCTC

TTCCCCAACATCGGTATCACCGTCGGCTTCGCGATACACCACGTGGCGACGGACGGGTTC

AGCTACACGCATTTCATCAAACACTGGGCTGCAGTTAGCAGGTCGGAGCATTTCTCGAAC

ATGCAGCAACCGTCCTTCGATCGGGCCGGCCTTGCCGGCCTGCACGATTACTACCTTCTT

GCTGTCAAGATGAGGAGAGAGCTGAAGCCTGATCAGGATCCGGCCGAAACGTCGTCTCGG

GGCGAAGCCGATTCGGTCTGCGGCATTTTCACCCTCGGCCCGGACCAAATCGAGAGGCTG

AAGCTGTCGGTCCGGGCCAGGGCCGATAACATGGCTAATCGCTCCACATTTGTGGTGTCG

TCCGCGTACCTGTGGACTTGCCTCGTCAAGCTGCGGCGGTACGCGGACGATCAAATGGTC

TACTTCCCAATTGCGGCCGACTGGAGACAGCGGCTGCAGCCGCCGCTCCCGGCGATGTAC

TTTGGCAACTGCGTGTTCTTCTCATTCGTGGAGATGAAAGCAGGGGATATTGTCAAGGAC

GACGGAGTTGCGGCAGCGAGCGTGGCGATTGGAAGAACTATTGAAGGGTTTAAGGATGGG

ATTGTTAAGGTTATAGAGGAGGGGAGGGAGATGAGAGCTAGAGCCTTTGCGAGCCGGTCG

CTGTTTACGATCGGCGGTTCGCCGAGGTTCGGAGTTTACAATGCCGATTTCGGATGGGGA

CGGCCTGTAAAGGTCGACGTGTGCATGAAGAATGGAGGAGTGTTTATGGCGGAGAGCAGG

GAGAAGGCCGGAGGTGTTGAGATTGGTATAGTGCTCCCAAGGCATGAGATGGAAGAATTT

GGTTCTATTTTTCGGGAGGGCCTTGAATTTATCTAA

>TRINITY_DN1024_c0_g1|m.402 TRINITY_DN1024_c0_g1|g.402 ORF TRINITY_DN1024_c0_g1|g.402 TRINITY_DN1024_c0_g1|m.402 type:5prime_partial len:123 (+) TRINITY_DN1024_c0_g1:2-370(+)

TCTCTTTCTCAAAACTCTCTCTCTCTCAAATCTCTCTCTCTGCTCAGCTCTCTCTCAACT

CTCTCAAATCTCTACCTCTCTCTCTCTCTCAAATCCCCGCGCTCAACGTCGACCCCAACA

CCGTCCCCCTCCCCCGACGGCGATGGCGAACGACGTCCCCTAGGGTTTCCGACGGCGGCG

GTCGGGTCTGCGGTTGCTGCTCAAAGAAGGGGAGGTGGTTCGGTCGAGCAGGAGGAGTCG

GGAAGAGGGGGGTTTGGACAGGCGGAGGAGGAAGCGACCGAGGTTGGAGAGGTCAAGGAA

GGGGGTTCAGGCGCTAGGGTTAGAGTTTCGGGTCGGGGCCGCTGGATGACTGGAGTTTTG

AGGAGATAG

>TRINITY_DN1024_c1_g1|m.404 TRINITY_DN1024_c1_g1|g.404 ORF TRINITY_DN1024_c1_g1|g.404 TRINITY_DN1024_c1_g1|m.404 type:5prime_partial len:512 (-) TRINITY_DN1024_c1_g1:2145-3680(-)

TTCCCTGCTCCCCCTCCTCCGCCCCAAAACACTAACTCCAACCATAACCCTAACGTCCTC

CCCCATTCTCCGCCGCCGTACCACCAGCAGCAGCCGCAGCCCTACCCTCCCCATCACTCC

GGCTACGGCCGAAACCCTAACCCTAATCCCAATTTCAGCAACCCCCAATCCCAGCGAAGC

TACCACCAGCAGCTGCCGCCTCCGCCGATCCACCGATCTCATCACCACCAGCAGCAGTAC

CCGTACTCGGGTCCTCCGCCTCCAGCTCCGCCTCCTCCGTCGTCCTATGCTCCGCCTCCG

CCTCATAACCCTCAGCCCCCGATTTATTACTCTTCCTCGGCTGCTGCGGCGGGTCCTTCG

TCATCGCAGTACAGTTCCAGCTCCCTCCCTCTGCCGCCGCCTCCCCCAGCTATGCCCCCG

CCACCACCGTCTTCCCCTCCGCCACCTCCACCTCTGTCGGGCCCACCACCGCCCCAGCCG

CCTGTTAACCACCGGAAAGATGCGGTATTGCTGTCAAAACAGGCACCACCTAAGCTGCCA

CAGCCGCAGCAGAAGAGAGCAGAGACGGAGGAGGAGCGGAGGGCCAGGAAGAAGAGGGAG

TACGATAAGCAGCGGGCAGAGGAGAGGAGGCAGCACATGCTGAAACAGTCGCAGGCGACC

GTGCTGCAGAGGACCAAGGAGGCCATGGGGTTGTCGCACGGTCATCACCATCATCAGGGG

TCGATGTCAGGATCGAAGATGATGGAGAAGAGGACCAACCTGTTTTTGAGTGGGGACAGA

GTCGAGAATAGGCTCAAGAAGCCTACCACTTTCATCTGCAAGTTCAAGTTTAGAAACCAA

TTGCCAGATCCATCAGCACAACCAAAGCTGTTACCAGTATTAAAAGATAAAGATCGTTAT

ACCAGGTATCGTATAACATCGTTGGAAAAAAATTACAAACCAAAGCTTTTCGTTGAGCCT

GATCTCGGGATACCTCTTGACCTACTCGATCTCAGCATATACAAAGCACCAGAAGTTCCA

CCACCCCTTAATCCAGAAGACGAGGAGCTATTGCGTGATACTGAGGTAGCAACCCCTATC

AAACATGAAGGTATCAGAATAAAAGAGAGGCCAACAGATATAGGTGTTTCGTGGTTGGTT

AAGACGCAATATATCTCACCACTTAGCATGGATTCGGCTAAACTGTCCTTAACAGAAAAG

CAAGCAAAAGAAATGCGGGAAACGAGGGAAGGGCGGAATGTTTCCTTGGAAAACCTTAAC

AATAGAGAGAAGCAAATACAAGCCATTGAGGAATCTTTCAAGGCTGCCAAGTTACCTCTG

GTCCACCAGACCGATCCGTCGTTGAAGCCTTTGGAAGTCATCCCCCTGTTACCTGATTTT

GATCGGTATGATGATAGGTTTGTCATGGTAGGCTTCGACAGTGATCCTACAGCTGATTCT

GAAATGTACAACAAGCTAGACAGATCTATTCGGGATGAGCATGAATCACAGGTGAAGAAT

GTTCTCTTTTGGTCAATAGGTCTATATAAATTTTAA

>TRINITY_DN1024_c1_g1|m.406 TRINITY_DN1024_c1_g1|g.406 ORF TRINITY_DN1024_c1_g1|g.406 TRINITY_DN1024_c1_g1|m.406 type:complete len:171 (-) TRINITY_DN1024_c1_g1:1179-1691(-)

ATGAAAAGTTTTGTAGTTAATGGTTCAGATCTTACAAACCCAGAAAAGTTTTTGGCGTAC

ATGGTTCCTGCACCAGATGAGCTAAACAAGGACATCTATGATGAAAGCGAAGATGTATCT

TATTCTTGGGTCAGAGAGTATCACTGGGATGTAAGGGGTGACGATGTTGACGACCCCACA

ACATACCTTGTTAATTTTGGAGATGAAGATGCCCGATATTTGCCTTTACCCACAAAGCTT

GTATTAAAAAAGAAGAAGGCGAAAGAAGGAAGGTCCAGGGATGAAGTTGAACATTATCCT

GTGCCTTCACGGGTGACTGTGAGGAAGAGGGCAACTGTTGCTGTTGGAGAGCAGAAGGAA

TTTGTGGGCACATCATCTAATCCTGACAAAGGAGACATCACAGATTTAAAACGGGGTAGA

TCTTTGAGGGAGTATGAATCTGGACCACGGGATAAAATCCCTCGAATGGACAGCATAGAT

CAATTCAGTGGAGAGGAAGACATGTCAGATTGA

>TRINITY_DN1024_c2_g1|m.410 TRINITY_DN1024_c2_g1|g.410 ORF TRINITY_DN1024_c2_g1|g.410 TRINITY_DN1024_c2_g1|m.410 type:complete len:872 (+) TRINITY_DN1024_c2_g1:599-3214(+)

ATGTCGACCGCCGACCACTTCAACGGCATGAACGACCCCCAGTTGAAGCCCCAGATGCTA

AGATCTCTCATCCGAGATCACCTCCCCGACGAGAAGCGCCCGTTCCCAAGCTCGCCGGAG

CTCGCATTCGTCCTCTCCCTGGTCAAGACCCACGGGCTCTTGTCGGAGCGAGACCCGAAT

ACGGATCCGAACCCGAAGGTCATGGACGCTTGGAGATCCGCCGTCGACGATTGGGTTGAC

CGCTTGATATCTCTCATTTCAAGCAACATGCCAGATAAGTGTTGGGCGGGTATTTGTTTG

CTGGGCGTGACTTGTGAAAGCTGCACGTCTCAACGGTTCTTAGCGTCGTACTCGATTTGG

TTCCAGAAGCTTCTGTCTAATATACAGCCCCCATCTAGCACCCATTTTGTTAGAGTGGCT

TCCTGTGCTTCCTTGGCAGATCTTTTAACAAGGCTGGCCAACTATCCGAATGTAAAGAAA

GATGCAACATCACTTGTCGGGAAACTTATCCAGCCCATACTCAATTTATTAGATGAGGAT

GGTGCGGAATCTGTATGGGAAGGAACTATTGATTTGTTATGTACCGCCATAACCTTGTTT

CCGTCTTCCATTCACCGTCATTATGACAATGTTGAAGCTGTTCTTGTATCTAAAATAATG

TTAGCAAAAAGGAACCGAGATCTGTCTAAGAAATTTGCCTGTTGTTTATCATTGCTGCCA

AAGGTGAAAGGTGATGAAGATAGCTGGTCCCTATTGATGCACAAGATTCTAATATCAATA

CACTTCCTTCTAACTGACGCCTTTCAAGGACTAGAAGAAGAAGCTAAAAGTTCTGAGATT

ATGAGACTATTAGCTCCGCCAGGAAAAGATCCTCCTCCCCCTTTAGGAGGCCAATTGATG

TTGGAAGCTTCTAAACAAGCAACAAAAATGCTTCCTGGATTGCTCATACCTCAAGTATCT

ACTCTTATGTGTTGTTGCTGTATGATGCTTTCTACACCCTATCCAGTTCAGGTGACCATT

CCGATCCACAATTTGCTAGTTCTGGTTGGGAGAGTGCTTCGTATTGATGGTTCTTTGCAT

GGCTCGTTATCACCTTTCACCACTGCCATGCATCAAGAACTTCTTTGTGCAGAGCTTCCA

GCCTTGCATTTGGACAGCTTGGATCTTTTGGTTGCCATCATTAAAGGAGTGCGGAGCCAA

CTGTTGCCCCATGGTGCCGGTATCGCTCAACTCCTAACAGAGTATTTCAGGAGAGCAGCA

TTGCCTTCAATACGGACAAAGGTTTATTTTATCATGCAAACATTGCTGATCTCTATGGGT

GTCGGTATGGCTCTATACCTTGCCCAAGAAGTAATCAACAATGCCTCCGCTGACCTTAAT

GATAGTCCTGGGAATGGGGCATTATCTTCTACTATGTACTTATCAAAATCAATAACTGAG

GCATCACAGCATAGTTCCCACAGAAAAAGGAAACATGCTTCTGGAACACCTATGGAGCAG

CCAAATGTGGTTAATGTTGGAGCTGAGGCACTCGACAGAAAAATGCTATCTCCTCTTTCT

GTTAAGATAGGTGCACTAAAAGCACTGGAAGCCCTTTTGACTGTGGGTGGTTCTTTGAGA

TCAGAGTGTTGGCGGTCCAATGTAGATTCCCTCCTTATAAATGTTGCAATGAATGCATGT

GATGTGGGGTGGGCTAGCGAACAAAAGTATCCCAATCTAAGAGACGAAACACCGGTCTCT

CGGGCAGATTTCCAGCTGGCAGCATTAGAAGCATTGTTGGCATCGCTTCTTTCCCCTGCA

CATGTTCGGCCACCATATTTGTCTCAAGGCCTTGAACTTTTCCGCAGAGGGAAGCAAGAA

ACTGGAACAAAGCTGGCTGCGTTCTGTGCTCATGCCCTTCTATCATTAGAGATTCTCATA

CATCCACGGGTGCTTCCACTTGTGGATGCTCCTATCGCCAAAAGCTCAGCACTTGATGAG

CGACTCAACCACAATCATGAAAACCTGAACATGTCCTCATTTTCAAGGGGTAACCTAGGA

AATATCAATGACCTATATGACGATGACGATGATCTGTATAATAGTTGGCTTGGTGACGAA

CCTACAATGGAGGATAACAATGCTACTACACATATGGAGAACCAAAAGCAGTCTTTGAGG

GGATCTATGGAAGAGGGCGTAGTAGAAAAGAATTCAGTAGATGAACAGGTAAGAGGTGAA

GTGGCTGCAGAAGCGAGCCAGGTGATGATGCACGCTGCTGACGTGGAGATGGCATCCACA

GATAGAGATGCTAATATTAACACTCACCAAGAATCAGCATCCAGTTACTTGCCTGAAGCC

GATAAGGAAGATTTCAGATTTACAAGCGGATCATCCCACAACAACAATTTTGGCGTATCA

ACAAACCTTATTTCTGGCAGTTTGTCCGGCGACAAGATAGAACCATGCAACGGTACTGTG

GTCTCAAGCAACAACTCAAATCTTAACAAAGACATTGGCAGTATTTCTGAAAGCCTATTG

TTCAAGGACCGGGACAGAACAAATTATGATTCTGATTCGATATCTGTCGACTCATTGCCG

GATATAGTTGATGGAGACCCAGATTCTGATGATTAA

>TRINITY_DN1024_c3_g1|m.412 TRINITY_DN1024_c3_g1|g.412 ORF TRINITY_DN1024_c3_g1|g.412 TRINITY_DN1024_c3_g1|m.412 type:complete len:143 (+) TRINITY_DN1024_c3_g1:223-651(+)

ATGTCACATCCAACGATGGGGACGACGCCTTCTTCGCCCAGGAACGCAAAGCGGTCCAAG

GCGGCGGCAGTTCAGAAGAATGAGGTGCCGGCGCCCGCGGAGTCGTCGCCGAATCGTGGT

GTCAACAACGGCGGCGCCGACGTCGATTCGTCGAAGGAGAAGAATGAGGTGCCAGCGTCG

ATTCGTCGATTCGTCGACGTCGATCCTACTGGAGAGTCGACGCCGGGGAAGGCGCAGCCA

CAGCTTGATATGAGAATCCCATGGGGCAAGCTCCTTTCTCAGTCTTCTGAGGTCAAGGGA

AAGGCGGTCATGCTCGATAAGATTATCAACTATGTTCAATCGTTGCAACAACATGTTGAG

CTTCGAAAAGACGTGGTTTTGGGGCTGAACCTGAGAAAAAGAAGGAAATCAAGGAGCAAT

GGCTGCTAA

>TRINITY_DN1024_c4_g1|m.414 TRINITY_DN1024_c4_g1|g.414 ORF TRINITY_DN1024_c4_g1|g.414 TRINITY_DN1024_c4_g1|m.414 type:5prime_partial len:168 (-) TRINITY_DN1024_c4_g1:339-842(-)

ATTAGCGAAAGAATGAAGTATCTTCAAGACCTTGTGCCTGGTTGTAGTAAAGTCACAGGA

AAGGCAGTCATGTTGGATGAGATTATCAACTACGTTCAATCATTGCAACGGCAGGTCGAG

TTTCTGTCTATGAAGCTTGCCACCGTGAATCCAAGTATTGACCTAAACATAGAAGAGCTT

CTGTCAAAAGATATGCTACAGGCCCGAGTTGGTCCATCTCCGATGGGTTTCTCGCCTGAT

CTTATTCATCCTCAGTTACTTCCACCTCAACAGGGTTTGGTTCAAACTAGGATTCCTGGC

ATGGTAGATCATGCAGAAGCACTCAGAAGGGCCATAAATGCTCAGTTTACAGCTATGAAT

GGTCTCAAGGAACCGACAACTCAGTTGATGCCGAATCCATGGGAAGCTGAGTTTCAAAAT

ATGGTTCACATGAACTTTGGCACCGATCCTCCTCCCAACAACCAGGAGTTTAATGACAAA

CCTAATGATGGCTTTCCACTATAA

>TRINITY_DN10255_c0_g1|m.415 TRINITY_DN10255_c0_g1|g.415 ORF TRINITY_DN10255_c0_g1|g.415 TRINITY_DN10255_c0_g1|m.415 type:complete len:484 (-) TRINITY_DN10255_c0_g1:440-1891(-)

ATGGAAACAACCCACCAGCAGCCGGCGGTGGAGGCCGAGGTGCAGCAGCCCCCAGCCTTC

CGCCTCTTCTCTACGAAAAACCCTAAAAAATCACCGTCCAAAACCCTAACCCTAGACCCA

AACCCTAGCCCTAGGACCTCCATCACCGACCACGGCGACTCCGACCCCGCCTCCACACTA

TCCTTCTCCAACCTCGGCCTCTCCCCATGGGCCGTCTCCACATGCGCCGCCCTTGGCATC

CACTGCCCCACCGCAGTCCAGCGCCGCTGTGTCCCCCACGTTCTCGCCGGCAAGGACGTC

CTCGCCATCGCCCGCACCGGCTCCGGCAAGACCGCTGCTTTCGCCCTGCCCATCCTCTGC

CGCCTCGCCCAGGACCCCTACGGGGTCTTCGCCCTTGTCGTTACTCCGTCGAGGGAGCTT

GCTGTTCAGTTGATCGAGCAGTTTAGGGCGTTTGGGGCGAGCCTGAATGTGAGGTGTTGT

TTGGTTGTTGGGGGGATGGGGATGACGGCGCAGGCGAAAGCGCTGGCGGAGCGGCCGCAT

GTGGTGGTGGCCACGCCGGGGAGGATTAGGGCTCTGATGGAGCAGGATCCGGATATTGCC

GCGGTGTTTGAGAAGACAAAGTTCCTGGTTCTGGATGAGGCGGACAGGGTTTTGGATGTT

GGTTTCGAGGAGGAACTAAGCGTGATTTTTAAATGCTTGCCTAAAAAGAGGCAAACTCTT

CTATTTTCTGCAACCAAGACAAGTACTTTGCAATCATTGCATGAGATTTCTCCAAACAAT

ACTTACTTTTATGAGGAATATGTAGGGCTGAGCACAGTTGAGTCTCTTAAACAACAATAC

ATTTTTATACCCAAAAATGTGAAGGAAGTCTATCTTTATCACATTTTGTCAAGAATGGCG

GATGATGGTATACGTTCAGCCATCGTTTTTATAGCTACATGCAAGAATTGTCATCTTCTG

AGTTTGTTATTGGAAGAGCTTGATCAGAAAGTAGTTGCTTTGCACTCTGACATTTCTCAG

TCTGAAAGGCTTTCGGCATTAAGTAGATTTAAATCTGGCCAGGTTCCTGTTCTGCTTGCA

ACTGATTTAGCCAGCCGAGGTTTGGATATTCCAACAGTGGATCTTGTCATCAACTATGAC

ATTCCAAGGGATCCACCAGACTATATTCATCGTGTGGGGCGTACTGCAAGAGCTGGCAGA

GGGGGGTTATCAATCAGCTTTATTACACAGAATGATATAGACATTATTCATGCGATTGAA

GCCGAGACTAAAAAGAGATTGGATGCATATGAATGTGATGAGAAGGAAGTGCTTGAAGAT

ATTACTAAGGTGTTCAAGGCAAGGCGGGTTGCTAGAATGAAGATGAGTGAGGATGGATAT

GATGAAAAAGCAGCAGCTCGGAAAAAACAGAAAAAGCGCAGAAGAGAAGGTGCTGAAGAA

ACAGGAATATAA

>TRINITY_DN10256_c0_g1|m.421 TRINITY_DN10256_c0_g1|g.421 ORF TRINITY_DN10256_c0_g1|g.421 TRINITY_DN10256_c0_g1|m.421 type:5prime_partial len:100 (-) TRINITY_DN10256_c0_g1:3851-4150(-)

CTCTCCAATGTCCGTTTGCTGCCGCTCGGGCAGTCGGCCTTTCTTCTTCGTCTCCAAATC

TTTTCTTCATCATCATCAAAAAAATATCTCCTTTCTCAATATCTCTCTTCGCCCTTATTG

CTCCGTAATGTCTTACCGCCCAAATTTCCAGGGCGGCCGGAGAGGCGGCAGGGGCGGCGG

AGGCCGCGGCGGCGGAGGAAGAGGTGGGGGAGGCGGACGTGGAGGCGGCGGTGGAGGAGG

GCGAGGCGAGCAGCGGTGGTGGGACCCGCAGTGGAGAGCGGAGCGGCTCGGCCAGATTAA

>TRINITY_DN10256_c0_g1|m.420 TRINITY_DN10256_c0_g1|g.420 ORF TRINITY_DN10256_c0_g1|g.420 TRINITY_DN10256_c0_g1|m.420 type:complete len:119 (+) TRINITY_DN10256_c0_g1:550-906(+)

ATGCTGAGCCAGCCCATTCTGTGCTTGATACAACAGGAGGAAATGTTACAGCCGGTCACT

CTAAGGCCAGAACCAACCCCAACAACCACCACCACCAACACAGTCAAGAAAGACTGGTCT

TATTCTCTAACGAACGATGAAGACGCAGATGAAGATCTCCTCATTGTGGCACTTTTAGAA

GACGACATCACACCAGAGGAAGTCGAACGTCGTTTCGGACGCACTGGCCCCTTTTTGCTC

ATTTGCCTGTTTTTCATTTTATCTTTATTTTGCCTTAGCAGGAAAAGAGAGGAAAGAACG

AAAAAAAAAAAAAAAAAAAAAAACATAGGACTAAATACAGTTCATACAGAGGTGTGA

>TRINITY_DN10256_c0_g1|m.418 TRINITY_DN10256_c0_g1|g.418 ORF TRINITY_DN10256_c0_g1|g.418 TRINITY_DN10256_c0_g1|m.418 type:5prime_partial len:1035 (-) TRINITY_DN10256_c0_g1:1045-4149(-)

TCTCCAATGTCCGTTTGCTGCCGCTCGGGCAGTCGGCCTTTCTTCTTCGTCTCCAAATCT

TTTCTTCATCATCATCAAAAAAATATCTCCTTTCTCAATATCTCTCTTCGCCCTTATTGC

TCCGTAATGTCTTACCGCCCAAATTTCCAGGGCGGCCGGAGAGGCGGCAGGGGCGGCGGA

GGCCGCGGCGGCGGAGGAAGAGGTGGGGGAGGCGGACGTGGAGGCGGCGGTGGAGGAGGG

CGAGGCGAGCAGCGGTGGTGGGACCCGCAGTGGAGAGCGGAGCGGCTCGGCCAGATTAAT

GGACCGGTGGAGAAGCTGGATGAAAGTGAATGGCAGACTAAGCTTCAACAACTGAAGGTA

AGTAACCAGAAAGAGCTGATCATCAAACGAAATTTTGGGCGTGAGGGGCAGAATATTCTT

GCCGACATGGCTCATCAGCAAGACCTTCATTTCCATGCATACAACAAAGGAAAAGCTCTT

GTTTTTAGCAAAGTTCCGTTGCCAGATTATCGGGCTGATCTTGATGAACGACATGGATCA

GCGCAGAAAGAGATTAAGATGTCAACAGAAACGGAGAGAAGAGTTGAAAACCTTCTGGCG

AGATCTGTAGACACATCAAGCACTAATGGCTTGAGTAGCACATCAAGTCACACGAGTAGG

CAGTCATTGCCTCACATGGTTACATCAAACAGCGAATCAACAACACAGGTGGATGCTGGT

AAGGAAAAGATCAGTCTTGATCTTAGAGGTCTGCAAAACTTCAAAAAGTCCACCCCAGGT

GCAAGAGCAATGCAGTCTTTCAGGGAAAAACTACCAGCATTTAAGGTTAAAGAGGAGTTC

CTGAAAGCCGTGGCAAGCAATCAGGTATTGGTCGTTTCGGGAGAAACTGGGTGTGGCAAA

ACTACCCAGCTACCCCAATTTATACTTGAGGAGGAAATATCAAATCTTCGTGGAGCAGAT

TGTAGCATAATATGCACCCAACCACGTCGCATATCTGCTATATCTGTTGCAGCAAGAATT

GCTTCAGAAAGGGGTGAGAATCTTGGAGAGACTGTTGGCTACCAAATTCGTTTGGAAGCC

AAACGTTCAGCACAAACCAGGCTTCTTTTCTGTACAACCGGGGTTTTACTTCGGAGGCTG

GTTCAAGAACCTGATCTGCGAGGTGTTACTCATTTACTAGTGGATGAAATTCACGAAAGA

GGCATGAATGAGGATTTCCTCATCATAATTTTGCGTGACCTTCTTCCTCGATGTCCAGAC

CTTCGCCTAATTTTAATGAGTGCCACCATAAATGCTGACCTATTTTCTAAGTACTTTGAA

AATGCGCCAGTCATCCATATCCCGGGGATGACATTTCCTGTAGATGAGATGTTTCTCGAG

GATATTCTGGAGAAAACTCATTACAAGATCATATCAGAGGCTGACAATTTCCAAGGGAAC

TCAAGAAGAAGAAGACAGACATTTTATAAAAGTGACCCATTGACTGAAATGTTTGAGAGT

GTTGATATCAACAGTCAATTTGGAAGCTACAGCATTTCTACAAGGCAATCTCTTGAGGCT

TGGTCTGGTGCACAGTTGGATTTGGGTCTTGTGGAAGCTACGATTGAGCATATCTGTTGG

AATGAAGGGGATGGGGCAATTCTCGTCTTCCTTACTGGTTGGGATGAGATATCTAAGCTG

CTCGAAAAAATTAAAGCAAACACTCTTCTTGGAAATTCAAGCAAGTTTCTTGTTCTTCCA

TTGCATGGTTCTATGCCAACTGTGAATCAGCGTGAAATATTTGACAGACCACCTAGTACC

ATGAGAAAAATTATTCTAGCAACGAATATTGCGGAAAGCAGTATCACCATAGATGATGTT

GTGTACGTCATAGATTGTGGAAAGGCAAAGGAAACAAGTTACGATGCTTTGAATAAGCTG

GCTTGTCTATTACCATCATGGATATCAAAGGCTTCAGCGCACCAGAGGCGAGGACGTGCA

GGCCGTGTTCGGCCCGGAGTGTGTTATAGGCTATACCCGAAACTTGTTCATGAAGCAATG

CCCCAATATCAGTTACCTGAAATTCTCAGAACTCCCTTGCAAGAACTTTGCCTTAATATC

AAGAGCCTACAGTTAGGTGCAGTTGCCACATTTTTATCAAAGGCGCTTCAGCCTCCTGAT

CCGCTTGCTGTTCAGAATGCAATCGAACTTCTCAAAACAATGGGGGCTTTAGATGAAATG

GAAGAACTCACTCCTCTTGGCCGTCATCTTTGCACACTGCCTTTGGATCCCAACATTGGA

AAGATGCTACTTATGGGATCAATATTCCAGTGCCTAGATCCGGCTCTGACAATAGCTGCT

GCGCTTGCACACCGCGACCCATTTGTTCTTCCCTTAAACAGGAAAGAGGAAGCTGATGAT

GCAAAAAGATCATTCGCTGGAGATTCTTGCAGTGATCACATTGCTCTTCTGAAAGCTTTT

GATACATGGAAAGATGCAAAACGTAGTGGAAGAGATCGTGCTTTTTGTTGGCAAAATTTC

TTATCACCCATAACACTACAGATGATGGATGACATGCGAGGCCAGTTTCTTGATCTTTTA

TCAGACATAGGTTTTGTTGACAAAGCTAAAGGAGTGAAGGAGTATAACCACTACGGACGT

GATCTGGAAATGATCTGTGCTATTCTATGTGCTGGGCTTTACCCTAACGTTATCCAGTGC

AAAAGAAGAGGGAAGAGGACAGCATTTTACAGCAAGGATGTCGGAAAAGTGGACATCCAT

CCGTCTTCTGTCAATGCAGGGGTTCACATGTTCCCTCTTCCTTACATGGTATACAGTGAG

AAGGTGAAAACAACGAGCATCTATATCCGGGATTCGACTAATATATCAGAGTATGCGTTG

CTTCTCTTCGGAGGTAGCCTCAAACCAAGCAAAAGTGGAGAGGGTATCACAATGCTAGGA

GGGTACCTTCATTTCTCTGCACCAAAGAATGTGATACAACTGATTCAGAAGTTGAGAGGG

GAACTTGACAGACTTCTTCAAAGAAAAATTGAGGAACCACGTCTCGATGTCTACTCAGAA

GGAACGGGCGTTGTTACTGCAGCAGTCGAGCTGTTGCACAGCTAA

>TRINITY_DN102573_c0_g1|m.422 TRINITY_DN102573_c0_g1|g.422 ORF TRINITY_DN102573_c0_g1|g.422 TRINITY_DN102573_c0_g1|m.422 type:complete len:1259 (+) TRINITY_DN102573_c0_g1:162-3938(+)

ATGGAGAGCATAAAAGAGGAGAAGTTTCGATTTTGCATTGACAGAGGAGGCACTTTCACT

GACATCTACGCTGAGGTCCCCGGGCGATCGGACTGTTGCGTGATGAAACTTCTGTCAGTG

GATCCGTTGAACTACGACGACGCGCCGATTGAAGGGATTAGGAGGATTTTGGAAGAGTAT

ACGGGTGAGAAGATCCCGAGATCGACCAAGATTCCGACGGATAAGATAGAGTGGATTCGG

ATGGGAACCACCGTTGCTACAAATGCTCTTCTTGAGCGGAAGGGGGAGAGGATCGCTCTT

TGTGTGACTCGGGGGTTTAGAGACTTGCTTCAGATTGGAAACCAAGCACGCCCGAATATC

TTTGACCTTAAAGTCTCGAAGCCCTCGAATCTATACGAGGAGGTTATAGAGGCGGACGAA

AGGATTGAACTTGTTCTTGACAGAGAGAAGGAGTCTTCTTCTTTGGTTGAAGGGATATCG

GGAGAGCCTATCAGGGTGGCAAAACCGCTCAACAAAGAAACTCTGAAGCCTCTATTGAAA

GGTTTGCTCGATAAGGGAATTAGCTGCTTGGCGGTTGTGTTGATGCATTCTTACACTTAT

CCTCACCACGAGATCTTGATCGAGGAGTTGGCTTTGAGCATGGGTTTCAGGCATGTTTCT

CTATCTTCGGCTTTGACTCCCATGGTTCGTGCAGTGCCACGTGGGTTGACAGCTAGTGTG

GATGCTTACTTGACACCGGTCATCAAGGAATATTTATCGGGTTTCATGTCTAGGTTTGAT

GGTGAGGATGACAAAGTGAATGTGCTATTTATGCAGTCGGATGGAGGTCTAGCACCAGAG

AAGAGATTTTCTGGTCATAAAGCGGTTCTATCAGGCCCGGCAGGAGGAGTTGTTGGCTAC

TCTCAAACCCTATTCGGGCTTGAAACATCCAAACCCTTGATTGGGTTTGACATGGGCGGC

ACTTCAACTGATGTTAGTCGTTATGGTGGAAGCTATGAGCAAGTTCTTGAGACCCAGATT

GCAGGGGCGATAATTCAAGCACCTCAGCTTGATATTAACACTGTGGCTGCTGGTGGTGGA

TCCAAGCTCAAATTCCAATTTGGGTCATTCAGAGTAGGACCTGAATCTGTCGGGGCACAC

CCAGGTCCAGTTTGTTACAGGAAAGGAGGGGATTTAGCAGTCACGGATGCTAATTTGATT

CTTGGAACTGTTATTCCTGACTACTTCCCTTCTATTTTTGGTCCTAATGAGGATCAGCCT

TTAGACATTGAAGCTACCCGAAAGACATTTGAAAAACTTTCCCTCGAGATCAATTCATAT

AGAAAAAGTCAGGATCCATCAGCAAAGGAGATGACGGTTGAGGAGATTGCTCTTGGGTTT

ATAAATGTAGCGAATGAAACTATGTGTCGGCCTATAAGGCAGCTGACAGAGATGAAGGGG

CATGAGACACGAAACCATGCTCTTGCTTGCTTTGGAGGTGCAGGTCCCCAGCATGCATGT

GCAATTGCAAGGTCATTGGGTATGTCAGAGGTCCTTATTCATAGATTTTGCGGGATATTA

AGTGCTTATGGTATGGGGCTGGCTGATGTTGTTGAAGAGACGCAGGAGCCATATTCTGCC

GTTTATGATGAGAATTCTGTACCCGAGGCCTTTCGAAGAGAGGCTCTTCTCTCGCAGCAA

GTAAAACAAAAGCTAAGAGAGCAGGGTTTTAAAGATGAAAGTATTGTGACCGAATCCTAT

CTAAACTTGAGATATGAAGGCACAGATACAGCTATCATGGTGAAAAGGCAAATCGAAGGG

GAGGGGAATGACTATGCTGCTGAATTTGTGAAGCTATTCCAGCAAGAATATGGTTTCAAA

CTACAAAAAAGGAAAATCCTTATCTGTGATGTGAGAGTCCGTGGTGTAGGAATCACCAAC

ATCTTGAAACCTCAGGAACTTGAACCAATTTCAGGAAATCCCAAACCTGAAAACAATTAC

AAGATTTATTTCAAAGATGGGTGGCATGATACGCCTTTGTTCAAGCTCGGGAAATTGGGA

TACGGACACATCTTGCAAGGCCCTGCGATCATCATGAATGGTAATAGCACTGTAATTGTA

GAACCTGCCTGTAGAGCTGTTATTACCAAGTATGGTAACATAAGAATCGAGATTGATTCA

GCTCCAACCACTGTAAAAGTAGCAGAGAAGGTTGCAGATGTTGTGCAGCTTTCTATCTTC

AATCACAGATTTATGGGCATTGCTGAGCAGATGGGTCGGACTCTTCAGAGGACATCCATT

TCAACTAATATAAAGGAACGTCTGGATTTCTCATGTGCTCTCTTTGGTCCGGATGGAGGG

CTCGTTGCAAATGCTCCTCATGTTCCTGTTCATCTTGGGGCCATGTCCAGCACGGTTTGC

TGGCAACTCAAGTACTGGGGTGACAATCTCAGAGAAGGAGATGTTCTTGTAACTAATCAT

CCTTGTGCTGGTGGAAGCCATCTCCCTGATATAACTGTCATCACACCAGTCTTCGATAAT

GGAAAGCTAGTCTTCTTTGTAGCGAGCCGAGGACACCATGCTGAGATCGGTGGTATAACT

CCCGGTAGCATGCCACCCTTCTCAAAGGCCATATGGGAAGAAGGGGCAGCCATCAAAGCA

TTCAAGCTCGTAGAGAAAGGTGTTTTTAATGAAGAAGGGATCATCAAACTGCTGCAACAT

CCGTGCTCCGATGAAATTTCTGCTCCTAGAGTTCCAGGAACCCGCAGGATTCAAGACAAT

TTATCTGACCTCCGAGCCCAAGTTGCTGCGAACCAGAGAGGAATATCACTGATCAAAGAA

CTAATCCAACAATATGGTCTGGAAACTGTCCAATCGTACATGACATATGTCCAGTCCAAT

GCAGAAGAAGCTGTGAGGGAAATGCTTAAAGCAGTTGCTGCTAGAGTTGAACAGGAGAAC

GGTTCTACAGTCATTGAAGAAGAAGACTACATGGATGATGGAACCGTAATCCATCTGAAA

CTCTCCATCGATTCAAAGATGGGTGAGGCTAGTTTCGACTTTGAAGGGACCAGCTCAGAG

GTTTATGGCAATTGGAATGCACCCGAAGCGGTAACAGCTGCAGCAGTGATCTACTGCCTC

CGTTGCTTGGTGGATGTAGATATTCCTCTGAATCAAGGGTGTCTTGCGCCCGTCAAAATC

CATATTCCCCAAGGTTCTTTCCTTTCTCCTAGCGATAAGGCTGCTGTCGTGGGAGGCAAT

GTACTCACATCTCAGAGAGTGACTGATGTAGTCCTTACTGCATTCCAGGCCTGTGCTTGC

TCTCAGGGATGCATGAACAATCTTACATTTGGTGACAATACCTTCGGCTACTATGAGACT

ATTGGTGGAGGCAGTGGAGCTGGACCAAGCTGGGACGGGACAAGTGGAGTTCAGTGCCAC

ATGACGAACACCAGGATGACCGACCCAGAGATATTTGAGCAGCGTTACCCAGTTAAGCTA

CACAGGTTTGGCCTTCGGGAGAACAGTGGGGGCAATGGTCATCATAGAGGGGGCGATGGT

CTTGTGAGGGAGATAGAGTTCAGGCGCCCAGTAGTGGTGAGCATACTTTCAGAGAGGAGA

GTGCATGCGCCAAGAGGATTGAAGGGCGGAGAGAATGGGGCTCGTGGCGCCAACCACCTG

ATCCGCAAGGACAAGAGAAGGGTGTATCTTGGAGGAAAGAACACAGTTGAGGTAGATGAA

GGGGAGATACTTCAGATTCTGACTCCAGGTGGTGGTGGATGGGGATCTCCTCGCTAA

>TRINITY_DN10257_c0_g1|m.423 TRINITY_DN10257_c0_g1|g.423 ORF TRINITY_DN10257_c0_g1|g.423 TRINITY_DN10257_c0_g1|m.423 type:complete len:190 (+) TRINITY_DN10257_c0_g1:57-626(+)

ATGTTCATTATCAACTGGTTCTGGGACGTTCTTGCGCAGCTCGGTCTGTTGCATAAGAAC

GCAAAGATTCTGTTCCTTGGTTTGGACAATGCTGGCAAGACAACCCTCTTGCACATGCTC

AAGAATGACCGGCTCGCCACGTTGCAGCCAACCCTCCACCCGACATCGGAGGAACTTGCT

ATTGGCAATGTGAAGTTCACAACATACGATTTGGGTGGACATCAGCAAGCCCGCCGTCTC

TGGCGTGATTACTTCCCCGAGGTCGACGGTATTGTCTTCCTCGTGGACAGCATGGATGTT

GAGCGTTTCCCCGAGTCCAAGGCAGAGTTGGACTCCCTCCTGTCTATTGAGGAACTCGCC

AAGGTGCCTTTCCTCATTTTGGGTAACAAGATTGATGCACCTGGTGCGGTGAGCGAGGAG

GAGCTCAGGCATCATCTTGGTCTCTATCAGACCACTGGCAAGGGCAAGGTTCCCCTCAAT

GACATCCGCCCCATTGAGATCTTCATGTGCTCAGTCGTGCAGAGGCAAGGGTATGGCGAG

GGTTTCAGGTGGCTCTCACAATACATTTAA

>TRINITY_DN10258_c0_g1|m.425 TRINITY_DN10258_c0_g1|g.425 ORF TRINITY_DN10258_c0_g1|g.425 TRINITY_DN10258_c0_g1|m.425 type:complete len:139 (+) TRINITY_DN10258_c0_g1:98-514(+)

ATGTCGAAATCGATCGCTCACAACCTCACGGCGAGCCACGACACGCTCCGGTTCGGATTG

GATGGGGTCAAGGGCGAGATCGTCGGCGTTTATCCCCTCCAATCAAGTCGAGAGATCAAT

CAGAGGAGCATGATGGAGAAGAAGAGGATGATTCTCGATCTGACCTATGGGACGGCTACT

AATTTGAGGATGGATCTCGATCGCCAGATCCTTTGCAGGTTTCAGAGACCTCCAGGAGCA

ATACCATCTTCCATGATAGGTTTGGAAGCTCTTACTGGTGACCTGGATGATTTCGGGGTT

GAGGATTACCTCAATGTGCCCCAAGACTCTGAAACTTTCCGACCAGTTGATATGCATCAC

GGTATGGAGGTTCGACTAGGCCTCTCCAAAGGGCCTGCTTGCCCAAGCTTTATTTGA

>TRINITY_DN102595_c0_g2|m.426 TRINITY_DN102595_c0_g2|g.426 ORF TRINITY_DN102595_c0_g2|g.426 TRINITY_DN102595_c0_g2|m.426 type:5prime_partial len:284 (-) TRINITY_DN102595_c0_g2:50-901(-)

TGCATTCTTAAAATCGCAAAAATGTCTGAACGAGGCGGATTTAGTGGAGGATTTGGTTCC

GGCGAACGAGGAGGCCGTGGCGGCGCTCGAGGTGGGCCTGGTGGCGGAGAGCGTGGCGGT

CGTGGCCGAGGACGTGGCGGTAGAGGTCGCGGTCGTGGTGGACGTGGAAAAGATGGCGAG

AAAGACTGGACACCGGTTACCAAGCTTGGTCGTTTGGTCAAGGAACGCAAGATCACATCT

ATCGAGGAAATTTATCGTCATTCTCTCCCAATCAAGGAGCATGAAATCATTGATCTTCTT

CTTACCGGACTTAAAGACGAGGTGCTTAAGATCATGCCTGTTCAAAAGCAAACTCGTGCC

GGTCAACGCACTCGTTTCAAGGCCTTTGTGGCCATCGGTGATCACAACGGACACGTGGGC

TTGGGTGTAAAATGTTCGAAGGAAGTTGCGACCGCTATTCGAGGAGCAATTGTTGCCGCA

AAACTCGCCATCATTCCTGTTCGACGAGGCTATTGGGGGAATAACATCGGAAAGCCACAT

ACTGTTCCCTGCAAGGTGACTGGAAAGTGCGCCTCTGTCCTCGTGCGCCTGATCCCGGCT

CCTCGAGGCACGGGCATTGTCGGCGCTCCTGTTCCAAAGAAGCTTCTTCAAATGGCTGGA

ATCGACGATTGCTATACGACTGCCGTGGGACAAACCGCCACATTAGGCAACTTTGCCAAG

GCCACCTACTACGCTATTCAGCGAACCTATTCATATTTGACACCTGATTTGTGGGAAGAG

AATCCCCTCGAAAAGGGTCCTTACCAAATTTACCACGAATTCTTGGCCGCCGCCGGAAAT

AAGCGTAATTAA

>TRINITY_DN1025_c0_g1|m.429 TRINITY_DN1025_c0_g1|g.429 ORF TRINITY_DN1025_c0_g1|g.429 TRINITY_DN1025_c0_g1|m.429 type:complete len:1017 (-) TRINITY_DN1025_c0_g1:278-3328(-)

ATGGATCTCACTATTCTCACCCTCACTTCCTCCTCCTCCACCTCCACCTCCCACCTCCTC

TTCTCCCCCTTCCGTCCCAACCCCCTTCGCCTCAAACCCCCGCCCCTCTCCCGCCCTCGC

CGCTGCCGCGTATCCTTCCCCCGCAGCTCCGCCGCCTCGACCCCCTCGATTTCGACGGAA

AATACTAACAACAAGGTCAAGGTCGACCCCTCGATCTTCCTCCCGAAGAAGGAGGTCGCC

GGCCCCAAATCGGCGTTTGGGGCCTTGCCGCAGCCGGCGAGGGTGGCGAGCTCTGTGATT

ATTGCTGCTGCGGCTGCGGCCGCGGGGTTTGGGCTGGGGTTCAGGTTCGGGAAGAGTAGG

GTTGCGGGGATCGGAGGGGCCGCGGTGGCTGGGGTGGCCGGCGGCGCGGCGGTGCTGGCG

CTGAATTCAAGCGTGCCGGAGGTGGCGGCGGCGGATTTGCATAATCTGGTTGCGGGGTTT

GATGATCCGGCGAAGCTGAAGAAGGAGGATGTCGATGGGGTTGTGCAAAAATATGGTGTG

AGCAAGCAAGACGAGACTTTCCGAGCAGAGCTCTGTGATCTCTATAGCCGGTTTGTAAAT

TCAGTGATTCCACCTGGAGCTGAAAATCTGAGAGGCAACGAGGTTGAGATGATCATCAAA

TTCAAAGCAGCTCTTGGAATTGATGACCCTGATGCTGCTTCCATTCATATGGAGATTGGG

AGGCGTATTTTCAGGCTAAGGATGGAGACCGGAGATCATGAGGCTGGTGTAGAGCAGCGC

CGAGCATTTCAAAAGCTCATCTATGTTTCAGCGCTTGTGTTCGGAGAAGCATCATCATTC

CTTTTGCCATGGAAGCGTATTTTCAATGTCACTGATTCTCAGGTTGATATTGCGATTCGA

GACAATGCCCAGAGATTGTATGCTTTGAAGTTGAGCTCAGTCAACAGAGGTATTGAAGAG

AAGCAACTCATTGATCTTAGAGAAGCACAACTTCTGTACAAACTTTCTGACGAGATTGCT

GCCGATATGTTTAGGGGACACACAAGAAAACTAGTTGAAGAAAATATCTCAAAGGCGCTT

GACATCGTGAAGTCTCGGGCAAAAGCAATGACGGGAACAATGCAAGTTGTTGAAGAACTC

GAGACAGTTTTGCAGTTCAATGATCTGCTTACACATTTAAGTAAACATCCTGAAGCTGGT

CGATTTCCCCCGGGGGTTGGGCCTGTTTCTTTGTTAGGCGGAGAATATGACAGCGATAGA

AAGATGGACGATTTGAAACTTCTCTATAGGGCATATGTAACAGAATGTTTTCCAAATGGT

CGTCTTGAAGAAACAAAGCTTGTAGCTTTGAACCATTTGAAGAACATATTCTGTTTAGGA

AAACGAGAGGCAGAGGCAATAATGCTAGATATTGCCTCTAAGGTCTATCGTAGACGGCTC

TCACAATCTTTCACTGGTGGTGATCTGGAAGCAGCTCCTAGCAAAGCAGCCTTTCTTCAA

AATCTTTGTGATGAATTACATTTTGATCCACAAAAGGCTAGTGAGATTCATGAAGAGATT

TATAGGCAAAAACTTCAACAGTCAGTTTCTAAAGGTGAGCTAAGTGAAGAGGATGTTGCT

GCTCTATTGCACATACGAGTTCTTCTCTGTATTCCTCAGGAAACAGTTGATGCGGCGCAT

GCCGACATTTGTGGCCGCCTGTTTGAGAAGGTAGTGAAGGATGCAATCGCCTCAGGTGTT

GAAGGGTATGATGCTGACGTAAGAGCCTCCGTAAGGAAGGCTTCTCAAGGTTTACGACTG

ACCAAAGAAGCTGCAATGGCCATCGCTAGCAAGGCGGTTCGGAAGGTATTTATGAATTAC

ATTCAACGATCGAAATCAGCTGGCAGCCGTACTGAAGCTGCAAAAGAGCTGAAGAAAATG

ATTGCATTTAACACGCTGGTCATCACTGAACTGATATCTGACATTAAAGGTGAGTCAACA

ACAGAACCTGTGGAACCTGTTAAGGATGAACCTAAACAGATGGAAGACGAAGATGAGTGG

GAGACCCTGCAAACGCTTAGAAAAACAAGACCAAATAGAGAACTGGAGGCTAAGTTAGGA

AAGCAAGGTCAGACTGAAATAACTCTCAAGGACGACCTTCCAGAGAGGGACAGGGTCGAC

CTTTACAGAACCTACTTGCTTTTCTGTCTTCAGGGGGAGGTCACTGTAGTTCCTTTTGGG

GCACAAATTACCACAAAGAAGGACAGTTCAGAATATTTGCTGTTGAACCAACTTGGAGGA

ATACTTGGTTTGACTGGCAAAGAGATCATAGAGATCCATAGGAATCTGGCCGAACAGGCT

TTCATGAAACAGGCTGAGGTGATTCTAGCAGATGGTCAGCTAACCAAAGCTAGAATTGAC

CAGCTCAATGACGTGCAGAAGCAGGTTGGCTTGCCTGCAGAATATGCACAGAAGGTTATC

AAGAACATAACAACAACTAAGATGGCAGCAGCAATTGAAACAGCTGTAAGTCAAGGAAGG

ATTGGTATCCAACAGGTCCGGGAGTTGAAAGAAGCTAGTATAGATTTAGACAGCATGATT

TCAGAACGTTTGCGTGAGAACTTGTTCAAGAAAACAGTAGAGGAGATTTTCTCATCTGGT

ACCGGCGTGTTTGATGAAGAGGAGGTTTATGAAAAAATACCTGCAGATCTTAACATAGAT

GCTCTGAAAGCCAAAGGAGTTGTTCAAGAGCTCGCCAAGAGTAGACTATCTAACTCATTG

GTTCAAGCTGTGGCACTTCTTCGGCAAAGGAATCGGGATGGAGTGGTTTCATCACTTAAT

GACATGCTAGCCTGTGACATGGCTGTCCCTGCGCAGCCCTTATCTTGGTCTTCACCAGAA

GAGCTTGCCGACCTGTACTTGATATACTTGAAGAGCATTCCTAAACCAGAAAAGCTAACC

CGGCTCCAGTACCTGCTTGGCATTAGTGATTCTACTGCTGCGGTACTTAGAGACACAGCA

GAACGAGGAGCTTTGCCGCTTGAAAATGAGGAGGAAGAGTTTGTATTTTAG

>TRINITY_DN10260_c0_g2|m.430 TRINITY_DN10260_c0_g2|g.430 ORF TRINITY_DN10260_c0_g2|g.430 TRINITY_DN10260_c0_g2|m.430 type:5prime_partial len:551 (-) TRINITY_DN10260_c0_g2:326-1978(-)

AAGAGTCACAACCTCCCCGTCCGATACCAATTCTCCCTTGACACCCATCCAATCCCCCCC

TCCTCACGAATTGGCCTCTATTACCCGAGGAAGATGTTGCCCGCCGGTGTTCGACGAGCT

GCCCGTCTCGCTAACGTCGCCCGCGTCGGCACGAGGGCTGCTCCGATCCAGAACCGATGC

CTCACGAACCAACGAGCCCTCTCGACGAGCCGAGCGCTCCTTACCGCTTCGCCTGGCCGA

GATAGGACCCGAGAGATTGTTGCGCAGACAGTGAGCAGCATTGGCAGCAAGCGTGAGGGC

CAGCAGTACCTCAAGCTCTTCACCTCAGTCTCGTCCCAAAAGTTCGCCGTCATCAAGGTC

GGTGGTGCTATCCTTACCGAATATCTCGACGAGCTCTGCCGCAGCTTGCTCTTTCTCTAC

GAGCTCGGCCTATACCCTGTTATTGTCCATGGTGCTGGCCCGCAGCTCAACCGCCTGCTG

GAGGAGGCCGGTGTCGAGCCCCAATTCGAGGAGGGCATCCGGGTCACCGACGCCAAGACT

CTGGGTGTTGCTCGCAAGCTCTTCCTTGAGGAGAACCTAAAGCTCATTGACCGCCTTGAT

GAGCTTGGTGTTGCTACCCGATCCATCAGCGGTGCCTTCATGGCCGACTACCTCGACAAG

GAGAAGTGGCAGTATGTGGGAAAGATCACCAAGGTCAACAAGGAGGCCATTGAGAAGTCT

ATTGAGGCCGGTTACATCCCCGTCCTGACCTCCATGGCCGAGTCCGAGGATGGACGTCTG

CTCAACGTCAACGCCGATGTCGCTGCCGCCGAGCTTGCCCGTGCCCTGGAGCCCTTGAAG

GTCGTGTATCTGTCTGAGAAGGGAGGACTGTTTGATGGTGATGGCGAGAAGATCTCGGCC

ATCAACTTGGATGCCGAGTTTGACCACCTCATGTCCCAGCCCTGGTGCCGATATGGAACA

CGACTCAAGATCAAGGAGATCAAGGAGCTTCTCGACACCCTTCCTCGCAGCTCCTCAGTT

GCCATCATCCACCCCAGTGATCTGCAGAAGGAGCTGTTCACCGACTCCGGTGCCGGTACC

CTGATCCGCCGCGGTGACAAGGTTCAGAAGGTCGAGTCTGTCAGTGAGATCAGCGATCTT

GCCAAGTTCAAGCAGACCTTGGTCCGTGACCGGGAGGGTCTTGATGCCGAGGCTACTGTT

GACCGATTTGTGGACCTCCTTGGTGAGAAGAAGTTCAGCGCCTACTATGATGATGGCATG

CAGTGCTTGGCTGTTGTCCTTCCTGCCAGCGAGGAGCGACCTGTCGCCACTCTGGCTACT

CTCAACATCACCAAGTCTGGTTGGTTGAGCAACATTGCTGAGAACGTGTTTGCCGCCATC

AAGAAGGACCACCCTAGCCTGGCCTGGACTGTCAGCGAGGAGGACGAGAACCTGACCTGG

TTCTTCGAGAAGGCCGACGGAAGCTTCAACAAGAACGGAAGTGTTCTGTTCTACTACGGA

TGTGATCTCCGCTCCGATGCGCTCGTCCCCGTCTACGACGACTTTGTCGCCCACGGCCGA

GCCATGCTTGGCGACACCAACCTGGAGTCCCAGCTCCGCAGCGCCGCCCAGACTGCTAGC

AAGGCTCTCAACGCCTCACAGAGCCGAACCTAA

>TRINITY_DN10261_c0_g1|m.431 TRINITY_DN10261_c0_g1|g.431 ORF TRINITY_DN10261_c0_g1|g.431 TRINITY_DN10261_c0_g1|m.431 type:complete len:609 (+) TRINITY_DN10261_c0_g1:74-1900(+)

ATGGTTGTGGTCTCTGAATCGTTCACCAAGCTCTCCTCTTCTTCTCCTCGATTCTGCAAC

TCTTCTTCAATCAGCAGGAGGATTTTTTCGGATGTTGCTGGGGATATAACCGTTTATGTG

GATGGCCAATCTTTTCTATTGCACAAGTTTCCTTTGGTTTCCCGTTGTGGGAAAATTCGC

AAAATAGTAGTGGAGTCCAGGGATCCAGACCTTTCCAGATTGGAACTTCTAAATGTACCT

GGGGGTTCCCAGACATTCGAACTCGCTGCCAAATTCTGTTATGGCACAAACTTTGAAATC

ACCACTGCTAATGTGGCTCATCTCAGATGCATTGCTGAATATCTAGAAATGACAGATGAC

TACAGGGAAGTAAACCTCATTTCTAGAACAGAAACCTACTTGAATGAAGTGGTGGTGCAA

AGCCTCGAGAAGTCTCTGGAAGTCCTCTGTGCTTGTGAGGGTCTGCTTCCCATCGCAGAG

GATGTTGACATTACAAATAGATGCATAGATGCAATTGCCATTAATGCCAGCAAAGAACAG

TTGGTCTCTGGCTTGGCTCGCTTAGAATGCAATGGGAGCTCCGGAAAGTTGAAGATGGAT

TGCCAGGATTGGTGGGTTGAAGACCTTTCGGTACTAAGGATAGATTTCTATCAGCAGGTG

ATTGCTGCAATGAGGAGAACAGGAGTAAGATCAGATAGCATCACCACATCACTTATACAC

TATGCTCATACCTCGCTTAAGGGGATTGAGAAACGGCAGGCTTGGGATGCTGGAGTAACT

TTTGGCGAAGAGCAGAGAACTTTAGTGGAATCGCTTGTAGGTCTCATAGCAACCGAGAAG

ATCACGTCTGTTCCCCTGTCCTTCTTGTTTGGTATGCTAAGGATGGCAATTGAGGTGGAT

GCTTCTCTCAGCTGTAAGCTTGAGCTTGAAAGGCGGATTGGTTTTCAATTGGAAATGGCA

TCACTTGATGATCTTCTCATTCCTTCCTTGCAGTCAAGTGAGTCAGTGTTTGACATTGAT

ACTGTTCACCGGATATTGGTGAATTTCTTACAGAGGATTGAAGAGGAAGACTCTGACGAA

TCATCACAATGTGGATATGAATCTGATGGTCTCAAGTCACCTAGTCACAGTTCAATTCTG

AAAGTAGGGAGGCTAATTGATGGTTACCTTGCAGAAATTGCACCAGATCCTTGTCTGAAG

CTGCAAAGGTTCATGGCCATTATCGAGCTGTTGCCAGATTATGCACGAGTAATTGAGGAT

GGTCTTTACCGAGCCATTGATATATATTTAAAGGCACATCCATCACTGACGGAGTCCGAG

TGCAAGAGACTGTGCAAGCTCATTGACTGCCAGAAGCTCTCCCAAGAAGCTGCCAACCAC

GCAGCACAGAACGACCGTCTCCCTGTCCAGATGGTCGTCCGTGTTCTCTACTTTGAGCAG

CTTCACCTCAAGTCTGCCATCTCCAGCACCTCCGCTGACGGCTCCTTCTCTCACAGAATG

ATCAGTGGCAGTGCAGTCCCAAGTGCAGCCGTCTCCCCCAGAGACAACTATGCTTCTTTA

AGGCGAGAGAACAGGGAGCTCAAGCTCGAGATATCCAGAATGAGAGTTAGGCTCAGTGAG

CTGGAGAAGGAACAATCATTCATGAAGCAAGGCATGAGAGATGGCAAGCAAATAGAGCAT

GGGAAAGCATTCTTCTCTTCTCTTTCTAGGGGGATTGGCCGTATCGGGATCTTTGGACCT

GCTTCTGCTAAGCAGCACAAGTCATCGACGAGGAAGTCACAAGGTTCGGATGCAAAGAGT

AACCGGAGGAAGCAGAGACGATCGTGA

>TRINITY_DN10262_c0_g1|m.432 TRINITY_DN10262_c0_g1|g.432 ORF TRINITY_DN10262_c0_g1|g.432 TRINITY_DN10262_c0_g1|m.432 type:complete len:348 (-) TRINITY_DN10262_c0_g1:342-1385(-)

ATGGGTCGAAAGCCAGGCGATCCGAGCGCCGCCGGCGGGCGCAGATCGCTGCCGGTCCTC

CTCCTCGTCGTGTTCGTCTCCTGCTGCTTCGCCTACATGGCCTTCTCCTTCTCTCTCCGC

CGCTCAAATTCTATCGCTGAGACCCAGATCAAAGGAGTTGGGTCGTTGATCGAAGAGGCG

GAGGCCGACGAGGCGGAGGAGGAGTGCTGTAGAGGGGTGGAGAACTTGGAGCTGTGGGGG

TCGGCGGTGAAATGGGGAACGGATCACAAGTTCAATTCGTCGGTGGGTTGCTGCAAAGCT

TGCAAGGTCATGTGCGGGGGTCACGATGGGCCTTGCCTCTGCGATTCTTGGGTCTTTTGC

GGGGATAGGGAGCGGTGCGGGGAGAAATTTGGGGAGTGCTGGTTAAAGAAACAAAAGGAT

GTCCTGTTTCCTGCGTTGCAAGAGTCTGGGGAAAAGGTTATCTGGACTTCTGGGCTTATT

TTTGGAAAAGGAGAGGGCATTGTTGGCCTCGAAACTGAATATGGAACTCTTCATATCAAA

CTTATGCCTGATTGTGCTCCACATTCTGTGTCATATATTGTTGAGCTATTGGGATCACGG

CACTGTGCTGGTTGCCAATTTTATCGTGCTGAAGGTCGAGGAAATTTGTGGGATTCCAGT

GGGAACCACATAACAGATGCTTCTTTGGGGCCTCCTTATGCCTTGATTCAAGGAACACTC

GAAGCTGATGGAGTTCCATTCAAGAAGATACCTACAGAAGCATGTCTCAACATAAGAAGA

GGATCAGTTGCATGGATCGGCTCTGGTCCCGAATTCTTCATTAGTTTAGCAAATCACAAT

GAATGGAGAAAGGCATACACCGTCTTTGGTTCTGTTCTGCCTGAAGATATGAAGATTGCG

GAGAAAATTGCAGGCCTTCCTACAAAATCTGATGTCTGGAATAACTTTAACGTGTCGGTG

TTGGAAAGTCCTGTTTATTTGAAGGTCAGACGTATCCAGGGAGATCTTAGCAAGATTTCA

AACTATGCTACAAATGTGGCATAG

>TRINITY_DN102636_c0_g1|m.433 TRINITY_DN102636_c0_g1|g.433 ORF TRINITY_DN102636_c0_g1|g.433 TRINITY_DN102636_c0_g1|m.433 type:5prime_partial len:896 (+) TRINITY_DN102636_c0_g1:1-2688(+)

TTTAAAACACCAACAAAAATGGGCGGACGTCAGGCTCACGGCCGATCACTCCTGGCCAAT

CCCAAGGCCAAAAATACCGGAAACAACAAAAAGTCCAAGGCGCGCTCGCAAAAGAACGCC

CTCAACGCCTTTGGCATCGCTCAAGAAAACTTTGCGCCCCGGCAGAAGCTGACCCCCCGC

GTGAGGCAGCTCGACGTCGACATCGAGAGCGAGAGGAAGCACGGCCGAGACGAAGACGAC

GAGGAGGACGAGGAAGAGGAGGAGGATGAGCCCCAGAGGAAAAAGGTCAAGAGGCCCAGT

CGACCCGCCGACGGTGGCGCCGAGGACGGAAGCGATAGCGAGGGTAATGAGTGGCGGCTA

GGCGGTCTGAGAGAAGACGATGAGGACTCTGAGATTGAGAGTGACGAGGCGTTTGGTGAT

AGCGATGAGGAAAAGTTCCAGGGATACACATTCCGCGGGAGCAAGTCGACACATCAAGAG

GATGGCGATTCGGAAGATGATTCCCAGGACGACGAGGGTCAGACACTTGGCGAGGATGCC

ATTGACCTGGCAACAGCGCTCGACCAATTTGAAGAGGATTCGGACGAGGAGCCTGAGGGC

AAGGACCAATCTGGTTCTTCAGAATCCGATGACGATGCTTCAGACGAGGAGGAGGATGAT

GATGATGACGAGGACGACGACAGCGATGATGACGATCAAGAAGCCAACCCGGAGAAGCTA

GAAGCTCTTCAGGGGTTGATCAAGGGTTACGGTGGTCAGAAGGATGATGAGGAGGACAAG

CCCAAATCAAAAACCAAGATCAGCCTAAGCGACCTGGGACTCTCGGGTATCAGCGACGCC

AACGTCAAGAAGTCGATGAAGCTCATAAACAAGGAGGAGAAGGAGAAGCGTCCCGGTGCT

GCCAAGAAACTGGATGTCCCCCTGGCGAAGCGACAACAGGACCGCCTGGACCGGAGCGCC

GCCTACGAAAAGACAAACGAGACACTGGACCGGTGGAACGACACAGTCAAGCAGAACCGA

AGAGCAGAGCACTTGATGTTCCCTCTACCGCAGAACTCGGCCACTGCTGGTTTGGATACA

ACTGAAATTCGACCCCTGACCACTTCGAACCCCAGCAACGAGCTCGAATCCGCCATCATG

TCCATCATGGAGCAGAGCGGTCTGACCATGGACAAGCCCCAGAAACCTAAGGAGAAGGAG

TACGACGAGGAGGGTAACGAGCTCACGCGCAAGGAGGTGCTGGCCCGGAAGCGCATGGAG

CGTGAGCTCAACTCGCGCGAGGCTAAGAGGGCTAAGCGCATCAAGAAGATCAAGAGCAAG

GCCTACCACCGCGTACACAGGAAGCAGCGCGAGCGCGACGAGATGGCTACCAAGGAGGCC

ATGGAGGAGGCTGGCGAGATTGACTCCGATGAAGAGCGCCTGGCTCAGGATCGCCGACGT

GCGCTTGAACGTGTCGGACAGCGGCACAAGGAGAGCAAGTGGGCCAAGATCGGCTCCAAG

ACGAAGCGAGCCGTGTGGGACGACGACTTCCGAGCTGGTCTGACAGAGATGGCCCGCAAG

GACGAGGAGCTCCGGCGGAGGAAGGAGGGCCGCGCCGGTGGCTCCGATGAGTCTTCATCA

TCTGATAGCGACTCTGATAACGGAGATGCTTCGCTGCGGCGACAGCTAGCAGCTCTTGAG

GAGGAGGAAGATGTGCCGCAGAAGGGTCTCATGAGCATGAAGTTTATGCAAAAAGCCGAG

GCTGCCAAGAAGGAGGCCAACGACGCTCTCATCCGCCAGATCCAGCGAGAGCTTGATGGT

GAGGAGTTTGATGGCGACGGCGATGAGGAAATGGAGGAGGTGGGCCGTCGACAGTACGGC

GCCGCCAAAGGTCAGGCATTCAAGCCAGCTTTCGAGAAGCCAACTCGGGTGGCCAAGAAG

CAAAAGTCAACAGAAGAGGACGACGACGATGATGATGTGGTGATAACTACCAACGGAGCT

GGCAGCAACCCTGCCATCCCCAACATCACATCCCTCGACTCCTCTCGAACCGTCTCATCA

ACCGCAGGCGCCTGGTCCCGCGGTGAAACCCGCCGCAAGAAGAAGGGTCAGTCGGCAGCC

AACGTCGGCGACCTCGACCTCTCATCCAACGTCATCGTCGCCTCCTCCCGCAAGACAAAG

AGCAAGTCCAAGCCCGACGAGGAAGCTTCAGATGCTGAATCCGACACTGACCAGCATCTC

CCTCTCGCGATCCGCGACCAGGAGATGGTGGCGCGCGCCTTTGCGGGCGAGGACGTTGTC

GGCGAGTTTGAGCGCGAAAAGGCAGACGTTGCCGAAGAGGACGATGACAAGGTCGTTGAT

AATACTCTCCCTGGTTGGGGCTCATGGGTCGGTGACGGCGTGAGCGAAAAGGAGAAGAAG

CGCCATCAGGGCCGGTTCCTGACAAAGGTCGAGGGTATTAAGAAGAAGGACCGCAAGGAT

GCCAAGCTGGACAAGGTGATGATCAACGAGAAGCGGATCAAAAAGAACGACCGCTACCTC

GCCTCACAACTCCCTCACCCCTTCGAGTCCCGCCAGCAGTACGAGCGCTCCCTGCGTCTG

CCCGTCGGTCCCGAATGGATGACAAAGGAGACGTTCCAGGAGAGCACCAAGCCTCGTGTG

CTCATGAAGCAGGGTATTATTGCGCCCATGTCCAAGCCCACCATCTGA

>TRINITY_DN102638_c0_g1|m.437 TRINITY_DN102638_c0_g1|g.437 ORF TRINITY_DN102638_c0_g1|g.437 TRINITY_DN102638_c0_g1|m.437 type:5prime_partial len:247 (-) TRINITY_DN102638_c0_g1:135-875(-)

GGCCATGGCTCCTCCCAAACCAACCTCAAGCTTTATCTCTTCGCTTTCTCCCTCTCTACC

AATTCTCAACTCTCTCTTTCTCTCCATCCCATGGCGACGTCAATATCGCCATCCCTCTCT

CTTCCTCGCCTCTTCCAACCGACCAAAAGAACCAATCTCTCCTCCTCATTCCTCCCTCCC

TTCGCTATTCTCACCCCTTCTCCCCTCAAAAACCACTCCCTCCTCCTCCGCCCCCCGCGG

CCTCTCGCCGTCAGGGCCATGGCTCCTCCCAAACCTGGGGGCAAAGCGAAGAAAGTGGTG

GGCATTGTGAAGCTTGCGCTGGAGGCCGGGAAGGCCACTCCGGCCCCGCCGGTGGGGCCC

GCATTGGGTGCGAAGGGTGTGAATATTATGGCCTTCTGCAAGGAGTACAATGCCAAGACT

GCCGAGAAGGATGGATATGTTATTCCTGTTGAAATTACTGTCTTTGATGATAAGAGCTTC

ACTTTTATTTTGAAGACTCCGCCGGCTTCGGTTCTGCTGCTGAAGGCTGCAGGAGCGGAG

AAAGGCTCGAAATCACCGCAAACGGAGAAGGTAGGAAAGATAACAATCGAACAGCTACGC

GCGATAGCCCAGGAAAAGCTACCAGATTTGAACTGTACCACCATCGAGTCGGCCATGAGA

GTTGTCGCAGGCACTGCAGCAAACATGGGCATAGACATTGACCCTCCAGTGCTTGAACCT

AAAAGGAAAGTAGTGTTGTAG

>TRINITY_DN10263_c1_g1|m.441 TRINITY_DN10263_c1_g1|g.441 ORF TRINITY_DN10263_c1_g1|g.441 TRINITY_DN10263_c1_g1|m.441 type:5prime_partial len:581 (-) TRINITY_DN10263_c1_g1:114-1856(-)

TTCGACATCACCACACACCTCGACACCGCAACCCACTTCACCATGTCTGAGGAGTCCAAG

CCGGTCGAGCCGGTGGTCGACGAGACCAGCGCCGAGCTCAAGGGCAAGGCCATCGCCGAA

GCCGAAGCTGAAGCCGAATCTGGCTCCGAGTCGGAGCACGACGAACATGACGAGCATGCC

GATCCCAACGCCGCCGAGGGCTCCAGCGACAAGAAAAAGAAGAAGAAGAAGAAGTCGAAG

CGCACCAAGGTCAAGGAGGCCCTAACCGGCCAAAAATCTGGCCCTACCGATTCCGACTTC

CACAAGGCCCTCGACGGCCTCACTCCCCAGCAGATCAAGGAGTTCCTCGCCCTCAACCCG

GCCCTGGCCAAGGAAGTGTCCAAGGCCTCGAGCAGCGAGAACCCCTCTGCCGATGAGGCC

GCCGCCATGCTGCGCAAGATGAACCTCCAGGACATCATGACTGGACTTGCCGCCGGCGGA

AAGAATGCCAAGGACATGGGCTCTTACAAGTTTTGGCAGACGCAGCCTGTGCCCAAGTTT

GGTGAGGACTCGACTCTGAAGGAGGGTCCCCTGCGCATCCAAAAGGTCGAGGAGGTGGAC

AAGGAGCCTGCGCCTCTGGTCGCCGGTTTCGAGTGGGTGACGATGGACCTGACTGATGAC

GAGGAGATCAAGGAGGTGTATGAGCTCCTCAACAAGCACTATGTTGAGGACGACGAGGCC

ATGTTCCGCTTCAACTACTCTCCCTCTATCCTTCGATGGGCCATGATGCCTCCTGGCTGG

AAGAAGGAGTATCACGTTGGCGTTCGCGCCACCCAGTCGCGCAAACTCGTCGCCTTCATC

TCAGCCATCCCCGTCAGGCTGCGCGTTCGCGAAAACTTCATCACCTGCTCCGAGGTCAAC

TTCCTCGCCATTCACAAGAAGCTCCGAGGCAAGCGCCTCACTCCCGTCCTCATCAAGGAA

ATTACCCGCCGCAGCAACCTTAACGAGATCTGGCAGGGTCTCTACACTGCTGGTGTCGTC

CTCCCCAAGCCCGTCAGCACATGCCGGTACTTCCACCGCGCCCTTAACTGGCAGAAGCTG

TACGAGTGCGGCTTCAGCCCTCTTCCCCCTAACAGCAAGCCTCAGTACCAGGTTCGCAAG

TATCACCTACCCGAGAGCACAAGCACCAAGGGTCTGCGCCCGATGGAGGAGAAGGATCTC

GAAGCCGTCATGGATCTGTACAAGCGATACACATCTCGTTTCGATATGACACCCGAGTTC

ACTCGTGAGGAGGCCTATCACTGGTTTGTCCCTAAGATTGAGCCCAGCGGAGAGCAGGTT

GTGTGGACATACGTTGTTGTGGATAACGACAACAAGATTACCGACTTCTTTTCCTTCTTC

TGCATTGAATCCTCTGCTATTGGTAACACCAAGCACAGCGTTATCAAGGTTGCCTACATG

TTCTACTATGGAACCGAGGTGGCCCTGCAAGAGACTTTTGACAAGGCCGCGCTTAAGAAG

CGCCTCAACGAGCTCGCGCACGACGCTCTCATCCTCGCCAAGCAGTACAAGTTTGACGTC

TTCAATGCCCTCACCCTCATGGACAACGCCCTCTTCCTGGAGCAGCAAAAGTTTGGTGCA

GGTGATGGCCAGCTTCACTACTACCTGTTCAATTATCGCCTCAACCCCATCAGCGGTGGC

GTCGATCGCAAGAACCACCTTGATGAGGAGAACCTCAGTGGCATCGGTTTGGTGATGCCT

TGA

>TRINITY_DN10264_c0_g1|m.443 TRINITY_DN10264_c0_g1|g.443 ORF TRINITY_DN10264_c0_g1|g.443 TRINITY_DN10264_c0_g1|m.443 type:complete len:515 (-) TRINITY_DN10264_c0_g1:1517-3061(-)

ATGAGTCCTCCTACAGATACCTTCTCCCGCGAGGAGGTCCGCTCGCACACGACAGAGGAC

TCTCTCTGGTGCATCATCGATAGCAAGGTCTATGACCTCACCGACTTCGTCGACGCCCAC

CCCGGCGGCGAGACCGTCCTCCGCCAGGTCGCCGGCCAGGATGCCACCGCCGCCTTCTAC

AACCTGCACCGACACGAGGTCCTGACAAGGAACGAGCGCCTCGTCGTCGGCACCATCGAG

GGCGAGAAGCCCCAGGTCATCACCCCGCAGCCCGGCGACCTCAGCAAGGTGCCCTATGCT

GAGCCCCTGTGGCTCGCGCCGCCGTTCCGCTCGCCCTACTACAGCGAGAGTCATAAGCGC

CTGCAGCGCAAGCTGAGAGAGTTTGTCGATAGCGAGCTGTACAAGGAGGCGCAGGAGTGC

GAGGCGACAGGGCGGTACGTTAGCCAAGCGATGATTGATCGCATGAGCGAGTTGGGAATC

TTGCACATGCGACTCGGTCCCGGCAAGCACCTCCACGGTGTTGAGCTCATGGGCGGCGCA

GTCAAGGGCGAGGAGTTTGACTACTTCCACGACTTGATTGTGGGACAAGAGCTGGCCAGG

GCCATGGCTAGAGGCTTCGCCGATGGCAACATGGCTGGTATGACCATTGGCTTGACGGCA

GTCCTTCAGTTTGCGCACGATGAGGCGTGGAAGAACAAGATCGCCCAAGAGGTCTTTAGC

GGCAAGAAGAAGCTGTGCCTTGCAATCACCGAGGCGTTCGCCGGTTCAGACGTTGCTGGA

CTACGAACCACTGCCGAAAAGACCCCTGATGGAAAGCACTATATTGTCAACGGAACAAAG

AAGTGGATCACCAACGGCTGTTGGGCAGATTACTTCGTGACGGGTGTTCGAACCGACAAG

GGCTTGAGCGTCGTCCTCATCGAGCGCGGAGAGGGCGTCGAGACCAAGGCCATCAAGACA

TCCTACTCTCCCGCCGCTGGCACGGCCTACGTGACATTCGAAAATGTCAAGGTCCCAGTA

GAGAACCTGCTTGGTCAGGAGAACAAGGGCATCCACGTCATCCTTAGCAACTTCAACCAC

GAGCGATGGATGATGGTTTGCGGCAGTCTTCGCATGTCGCGATCTATCGTCGAGGAGTGT

CTCAAGTGGTCGAACCAGCGCCAGGTTTTTGGCAAGCAGCTCATTGACCAGCCCGTCATC

CGCCAAAAGCTCGCCAAGATGATTGCGTTGGTCGAGGCCAATCAGTCCTGGCTCGAAACC

ATCACCTACCAAATGTGCCACATGCCCTACAAGCAGCAGGCACAGCACCTCGCAGGCCCA

ATTGGTCTGCTCAAGATGAGCGCGACTCGCGCTGCCCACGAGATTGCCGACGAGGCCGTG

CAAATTTGGGGCGGCCGAGGCCTCACACAAACCGGCATGGGCAAGTTCATCGAGATGTTC

CACCGAACATACAAGTTCGACGCCATCCTCGGAGGCGCGGAAGAGGTGTTGGGCGATCTG

GGTGTAAGACAGGCCATGCGAAACTTCCCCAAGGCCATGTTGTAA

>TRINITY_DN10264_c0_g1|m.444 TRINITY_DN10264_c0_g1|g.444 ORF TRINITY_DN10264_c0_g1|g.444 TRINITY_DN10264_c0_g1|m.444 type:5prime_partial len:220 (+) TRINITY_DN10264_c0_g1:3-662(+)

GACATTCTCTCTTCTCCCGCACCGCACGCACATCACCACGAATCTTTTCAATATCCTCCA

TCCCCCTTTCTTGCGCATCCCTCGCAAGATACTCACAGAAAACCTCCTCACACTACATTA

CTCAACTCATCAAAAATGGATTACCAGCAGCCTCGTGGTGCCTGCTACTCTTGCGGCTCT

ACCGGCCACCAGGCCCGTGACTGCCCTACCAAGGGACCTGCTAAGTGCTACAACTGCGGT

GGCGAGGGCCACATGAGCCGCGACTGCTCTGAGCCCATGAAGGACAACAAGTCCTGCTAC

AAGTGCGGCCAGCCCGGCCACATCTCCCGCGATTGCCCCATGAGCGGTGGCAGCGGACAG

GCCACTGAGTGCTACAAGTGTGGTGAGCTCGGCCACATTGCCCGCAACTGCACCAAGGCC

TCCTTCGGCAACTCATATGGTGGCGGCGGTGGCTTCGGCGGTGGTGCCGGCAAGACCTGC

TACTCTTGCGGTGGTTACGGCCACATGTCTCGTGAGTGCGTCAATGGCATGAAGTGCTAC

AACTGTGGCGAGTCTGGCCACTACTCCCGCGACTGCCCCAAGGAGTCCGCTGGTGGCGAG

AAGATCTGCTACAAGTGCCAGCAGTCTGGTCACGTCCAGGCTCAGTGCCCCAACAACTAA

>TRINITY_DN102664_c0_g1|m.447 TRINITY_DN102664_c0_g1|g.447 ORF TRINITY_DN102664_c0_g1|g.447 TRINITY_DN102664_c0_g1|m.447 type:complete len:406 (-) TRINITY_DN102664_c0_g1:268-1485(-)

ATGGAGCCTACAAATGGTGAAGTAATACGAAATGGAAGAGAAATCCTTTTCCAGGGTTTT

AACTGGGAATCTCATAAAATTGATTGGTGGAGGAATTTAGAGGAGAAAGTTATAGATCTT

GCTAAGTCTGGATTTACATCAGTTTGGTTGCCTCCAGCAACTCAGTCCTTCTCTTCAGAA

GGTTATCTACCACAAAACCTCGATTCTCTCGACTCTTCTTATGGTTCCCAAAACCAATTA

GAAACTTTGATTCAGAAGATGTGTAATAACAAAATTAGAGCAATGGCTGATATAGTCATT

AACCACCGAGTTGGGACTACTCAAGGGCACAGAGGAACGTATAATCGCTATGACGGAATC

CCATTTCCATGGGATGAACATGCTGTGACTTCTTGTTCTGGTGGACTGGGAAATCAAAGC

ACTGGTGCTAACTTTGATGGAGTTCCTAACATAGATCATACCCAAGCCTTTGTACGGGAA

GACATTATGAAGTGGCTAAGATGGCTTCGGAGAAGTGTTGGATTCCAGGACTTCCGATTT

GATTTTGCAAAAGGCTATGATGCTAAGTTTGTGAAAGAATATGTGGAGTCGTCAAAGCCT

GTATTCTCTGTGGGGGAATATTGGGATAACTGCAACTATAACCCCTCCGATTATCGCTTG

GACTACAATCAAGATAGCCATAGACAAAGGATTATAAATTGGATAGACAACACCGGAGGT

CTTTGTGCTGCATTTGATTTCACCACGAAAGGTGTTCTTCAGGAATCAGTAAAGGGAGAG

CTGTGGCGCTTGCGTGATTGTCAGGGAAAGCCACCTGGTGTAATGGGATGGTGGCCTTCG

AGAGCAGTAACATTCATTGAAAACCATGACACTGGGTCTACTCAGGCACATTGGCCTTTC

CCATCTGGTCATGTTATGGAGGGATATGCTTATATACTCACCCATCCTGGAATACCAACA

GTGTTCTATGACCATTTCTATGACTGCGGCGACACCATGCACGACCAAATTGTGAAGCTG

ATGAATATACGGAAATGTCAAGATATCCACAGCCGTTCATCAGTCAGAATTTTGGAAGCG

CAACCAAATCTCTATGCGGCAATCATTGATGACAAAGTGTGCATGAAGCTTGGAGACAGC

TCGTGGTGCCCGAATGATGGTGAATGGATACTGGCTACTAGTGGCCATAACTATGCAGTC

TGGCAAGTTAATGTTTAG

>TRINITY_DN10267_c0_g1|m.449 TRINITY_DN10267_c0_g1|g.449 ORF TRINITY_DN10267_c0_g1|g.449 TRINITY_DN10267_c0_g1|m.449 type:complete len:382 (+) TRINITY_DN10267_c0_g1:1735-2880(+)

ATGGTTAGGCACCACAAAGGTGTTGGGGTTGGCTTCGAGCATTGCGATGCGATAGTTGGC

GATGCCGAGCAGCAATTTGGACTTCTGAGCGAAGTTCCGAAGGATTCTGATGCTGCCGTG

CTCGCTCCTCACGAGGTGCATGAAGCTCTCCTGCTGGAAGACGTAGGGATTTCTTTCAGT

CCTCTCTTGTTCCTCCTCTTCTTCGCGTTGGGGGTTGATGTTGTCGCGCTCTCCGCCTCG

GCCTTGTTGGCGTTGCTGCTCCTCTTTGTACTCCCTCTGGCACTCCTGCTCGCACCTTTT

CTGCTGCTGCTGTCCTTCTTGTTGCCGGCAGTCCCGTCTGCACTCCTCGAGCCTCTCTTG

AGGGTCACGGCGGGTCTCGTCGCGCCCTTGCTCTTGTTGTTCTTGGTCGTATCGCTGCTG

GCATCGATGTTGGCACTGCAATTGTTCTTGCTGCTGGCCTTGGTGTCTCTCACGGCACTC

CTGCCTGCACTCCTCCAGTTTGCGCTTGGGGTCTCGTCGGCCCGAGTCACGTCCCTGCTG

GCGGCCGCGCTCCTCTTCGTACTTCTGTCGGCATTGTTGCTCGCAATCCCATCGTCGTGG

CTCGCCTTCACGTCCTTGGCGGCGGCACTGATCTTTGCATTGTTCGAATCTCCTCTCGGC

CTCCTCTTGACGTCCCTCGTCGCGTCCTCCTTGGCGTTTCTGCTCCTCTTCGTACTCTTG

TTGGCACTGACGCTCGCACTGCCATTGCTGCTGTTGGCCATGACGGCGTTGGCGGCACTT

CTGCTTGCACTGTTCAAGCCTTTTCTCCGGGTCCTGTCGGCTCTCGTCGCGTCCGTGTTC

TCTGCACTTCTCCTCTTCGTACTCCTGTTGGCACCGTTGCAAGCACTGCGAGCGTTGCCC

ACAGTGCGTGCAGCAGCTCACCTTACACTGCTCGAGACGTTGCTCGGGATCTTGACGATG

AGAGTTGAGGCTCTCATCATCATCATCACGTCCTCCTCGCTCCTTCTCTTGTTGTTGCTT

CTCCTCGCAGCGGCTCTCGCAACGCCTGCGCAAGTCCTGGTCGTAGTGGGGCTGCTGGCG

GCATTCTTGCTTGCACTGCTCGAGCTCGCGGCGCTCCTCTTCTTCTCTGCGAGAGGAGAG

AGCTAA

>TRINITY_DN10267_c0_g1|m.448 TRINITY_DN10267_c0_g1|g.448 ORF TRINITY_DN10267_c0_g1|g.448 TRINITY_DN10267_c0_g1|m.448 type:complete len:492 (-) TRINITY_DN10267_c0_g1:1468-2943(-)

ATGGCTACCAAAACATCCAAAGCTCTGTTCATAGTCCTCTCCCTCGTCCTTGTTTCCTCA

GCCTTAGCTCTCTCCTCTCGCAGAGAAGAAGAGGAGCGCCGCGAGCTCGAGCAGTGCAAG

CAAGAATGCCGCCAGCAGCCCCACTACGACCAGGACTTGCGCAGGCGTTGCGAGAGCCGC

TGCGAGGAGAAGCAACAACAAGAGAAGGAGCGAGGAGGACGTGATGATGATGATGAGAGC

CTCAACTCTCATCGTCAAGATCCCGAGCAACGTCTCGAGCAGTGTAAGGTGAGCTGCTGC

ACGCACTGTGGGCAACGCTCGCAGTGCTTGCAACGGTGCCAACAGGAGTACGAAGAGGAG

AAGTGCAGAGAACACGGACGCGACGAGAGCCGACAGGACCCGGAGAAAAGGCTTGAACAG

TGCAAGCAGAAGTGCCGCCAACGCCGTCATGGCCAACAGCAGCAATGGCAGTGCGAGCGT

CAGTGCCAACAAGAGTACGAAGAGGAGCAGAAACGCCAAGGAGGACGCGACGAGGGACGT

CAAGAGGAGGCCGAGAGGAGATTCGAACAATGCAAAGATCAGTGCCGCCGCCAAGGACGT

GAAGGCGAGCCACGACGATGGGATTGCGAGCAACAATGCCGACAGAAGTACGAAGAGGAG

CGCGGCCGCCAGCAGGGACGTGACTCGGGCCGACGAGACCCCAAGCGCAAACTGGAGGAG

TGCAGGCAGGAGTGCCGTGAGAGACACCAAGGCCAGCAGCAAGAACAATTGCAGTGCCAA

CATCGATGCCAGCAGCGATACGACCAAGAACAACAAGAGCAAGGGCGCGACGAGACCCGC

CGTGACCCTCAAGAGAGGCTCGAGGAGTGCAGACGGGACTGCCGGCAACAAGAAGGACAG

CAGCAGCAGAAAAGGTGCGAGCAGGAGTGCCAGAGGGAGTACAAAGAGGAGCAGCAACGC

CAACAAGGCCGAGGCGGAGAGCGCGACAACATCAACCCCCAACGCGAAGAAGAGGAGGAA

CAAGAGAGGACTGAAAGAAATCCCTACGTCTTCCAGCAGGAGAGCTTCATGCACCTCGTG

AGGAGCGAGCACGGCAGCATCAGAATCCTTCGGAACTTCGCTCAGAAGTCCAAATTGCTG

CTCGGCATCGCCAACTATCGCATCGCAATGCTCGAAGCCAACCCCAACACCTTTGTGGTG

CCTAACCATTTCGATGCAGACACCCTCTTCTATGTCGTGAGAGGTGAGGGAACAATCACT

CTCATCAAAGGAAACCAAGATCGCGAGTCGTACAACATAAGGCAAGGAGACATCTTGAAG

GTCTCGGCCGGTACCATTGTCTACTTGATCAACAGAAGCAACAACCAGAAGCTCAACATC

GCCAAGATCCTTAACCCCGTCTCCATTCCAGGCCAAGTCGAGGTACTAATTAAATTATTA

ATCAAGGCCATATATATATATATCCCATTATTTTAG

>TRINITY_DN10267_c0_g1|m.450 TRINITY_DN10267_c0_g1|g.450 ORF TRINITY_DN10267_c0_g1|g.450 TRINITY_DN10267_c0_g1|m.450 type:complete len:241 (-) TRINITY_DN10267_c0_g1:315-1037(-)

ATGAGCCGCCACTCCTCATCGGAACACGGCGGACACTGGCCTTTCGGCGGGGAATCAAGG

GGGCCGTACAGCCTCCTGAACAAGCGCCCGACCCACTCCAACCAGCACGGCCGCCTCTAC

GAGGTCGACTGCAACGACTACCAGCCCCTCCGTGCCCTCAACATCCAAGTCTCCTGGGCA

AACATCACCGGTGGGTCGATGGTGGCCCCCTTCTTCAACTCGAAGGCGACCAAGATCGCG

ATCGTGACCGAGGGCCGCGGATACTTCGAGATGGTATGCCCCCACTTGAGCCAGCAACAG

GGAGGCCAAGGGCAACGGGGTGAGCAAGAGCAAGAAGAAGGGCAGCAGCAACAAGGGCGA

CGGTACGAGAAGGTCCGGTCCCAGGTGTCGAAGAACACAGTGTTCATCGTGCCGCCAGGG

CACCCGGTGGCCATTGTGGCTGACAGGAACCAGAACCTGGAGGTATTGTGCTTCGAAGTG

AATGCCGAGAACAATAGGAGGGCGTTTGTTGCAGGGAGGAACAATGTGGTGAGCAAGATG

GAGAGGGAGGCCAAGGAGCTGGCGTTCGGCGTGCCAAGCAAGCTCGTGGACGAGGTGTTC

AGCGGACGGCAGCAAGAGGAGATGTTTGTTCGAGGTCCGCAGTCCTCGCAGCAGGAGAAG

GGGGAGGAAGAGGGAAGGCGCCAACCTCTCAACTCCATCCTGGAGTTTGCTGCAGGGTTT

TGA

>TRINITY_DN10269_c0_g1|m.455 TRINITY_DN10269_c0_g1|g.455 ORF TRINITY_DN10269_c0_g1|g.455 TRINITY_DN10269_c0_g1|m.455 type:complete len:509 (-) TRINITY_DN10269_c0_g1:524-2050(-)

ATGGCTCAAAATCTCCGCGCTCTCCTGCTGCTGCTCCTCACCTCCTTCCTCGTCTCCTCG

CACCGAGCCAACTCTCTCGACACCACCACCACCACCACCACCACCAACATCTGCATCGTC

GGCAGCGGAATCGCCGGCTCCTCTCTCGCGCATTTCATCAAACAATACACCTGCAACAAC

AGCAACCCCGACCCCTCCTCATGCATCGACGACATCCGAATCTTCGAGCGCAACGGCGTC

GCCGGCGGCCGCATGGCCACCGTTACCATCGGTGGCGACACTTTCGAGGCCGGCGGTTCC

ATCCTCCACCCCAAGAATCTCCACGCCTTGCGATTCGCGTCCATGCTCAATCTCTCCCAC

AAGCCTAAGTCTGAATCCGATTCCGATTCCTGGTTCGGGATTTGGGATGGGTCTCGGTTC

GTCTTCAAGACCCTGCAGCCTCCGGTCAGATCGAGCCCCTTCTTTTATAGGAAGATTTAC

TCGCTGCTGAATGATTTTCTGCTCTTTTGGCGATATGGGTTTTCTCTAATTCGGATGAAT

ACATTCGTTGAGAAAATGGTGGATCGCTTCTCGCTTTACTACAAGGAATTCGAGTCCCGG

CCTGTTTTTGAGACGGTGGATGGGATGCTCAAATGGTCGGGGTTGTATGAGCTCACCCGC

CGGACGCTGGAGGAGGAGCTGAAGGATGCGGGATTGTCTTCGCTCCTGATATCAGAGCTT

GTTACTGTAATAACAAGAATTAACTATGGACAGAGTGTCAAAATCAGTGGACTAGCTGGG

GCGGTCTCTTTAGCTGGTTCTGATTCAAAACTCTGGTCAGTCAATGGGGGAAATTGGCAG

CTTGCCGCTGGGCTAATTAATTATTCCAACGCCACATTGCATCTCCATGAAGGGATAACT

TCTATTGCCTATGCAGGAGACCACTATGTGCTTAACTCTACTGAGGGAAACACTTACAAC

TGCAAAGTTACTGTAATTGCTACACCTCTAGATGAGCTGAGCATCAGATTTACCCCTTCA

TTTTCTATTCCTAATAGGAGGTTACATCATACATTCACAACATTCGTGAGGGGCCTCTTA

AACCTTGAATATTTCGGCTTCAATCATGTATCGGAAATTCCTGACCTTATTGGTACTCTT

GAAGTCCCCAATGTTCCATTCTCCAGTATTTCTATTCTAAAGAAATATGGTGAAGAAGAG

ATGACTTACAAGATGTTCTCCCGCGGACCAGCGGATGATAGCTTGCTGGATCAACTTTTC

AGCGTTAGGAAGGAAACAATACGCATAGATTGGCCAGCATATCCTCATTTTGTAGCCCCA

GAAGTGTTTGCACCAATCCTTCTTGACGGAATGCATTTGTACTACATAAATTCATTTGAG

AATGCAGCAAGTACTATAGAGACTAGTGCGGTTGCAGCTGAGAATGTTGCGCGGCTAATC

ATCTCAAGACTTTCCAAAACGAGTTCTAGTTCTCCAACCATAAAGATCCTAACTTCGGAT

GAAGAGATATTGCATCTAGAATTATGA

>TRINITY_DN1026_c0_g1|m.458 TRINITY_DN1026_c0_g1|g.458 ORF TRINITY_DN1026_c0_g1|g.458 TRINITY_DN1026_c0_g1|m.458 type:complete len:211 (-) TRINITY_DN1026_c0_g1:433-1065(-)

ATGGGAGTCCAAGCCATGCTCATCCCTCTCCTCCTCCTCCTCCTCCTCTCCTCCACCCAC

GCTGCCGACTTCTGCGTCGGCGACGTAACCGGACCCGCCACCCCCGCCGGCTTCACCTGC

AAGAAGGAGTCCACCGTCACCGCCGACGACTTCGTCTTCACCGGCCTCGGCGTGGCCGGG

AACACCTCCAACATCATCAAGGCCTCCGTCACGCCGGCGTTCGGGGCCCAGTTCCCGGCC

ACCAACGGCCTCGGCATCTCCCTCGCCCGGCTTGACCTCGCCCCCAGCGGTGTCGTTCCC

TTTCACACCCACCCAGCGGCCTCCGAGCTACTGGTGGTGACTCAGGGCATTATTACCGCT

GGGTTTGTGTCGTCGGCTAATGCGGTGTACGTCAAGACCCTCCGTAAAGGGGATATCATG

ATTTTTCCACAGGGCTTGCTACATTTTCAGATAAACGGTGGAGGTGTGACTGCGGTTGCC

TTTGCAAGCTTCAGCAGCTCAAACCCAGGCCTGCAGATAACAGACTTTGCATTATTTGCT

AACGACCTCAGCACCAAGTTGGTAGCCGCCACCACTTTCCTCGACGTAGAACAGATAAAG

AAGCTCAAGGGGGTGCTCGGCGGAACCGGGTAG

>TRINITY_DN1026_c0_g1|m.459 TRINITY_DN1026_c0_g1|g.459 ORF TRINITY_DN1026_c0_g1|g.459 TRINITY_DN1026_c0_g1|m.459 type:5prime_partial len:102 (-) TRINITY_DN1026_c0_g1:791-1096(-)

GTTCAATCCAAATTAAGAGATAAAGAGAGCCATGGGAGTCCAAGCCATGCTCATCCCTCT

CCTCCTCCTCCTCCTCCTCTCCTCCACCCACGCTGCCGACTTCTGCGTCGGCGACGTAAC

CGGACCCGCCACCCCCGCCGGCTTCACCTGCAAGAAGGAGTCCACCGTCACCGCCGACGA

CTTCGTCTTCACCGGCCTCGGCGTGGCCGGGAACACCTCCAACATCATCAAGGCCTCCGT

CACGCCGGCGTTCGGGGCCCAGTTCCCGGCCACCAACGGCCTCGGCATCTCCCTCGCCCG

GCTTGA

>TRINITY_DN1026_c2_g1|m.460 TRINITY_DN1026_c2_g1|g.460 ORF TRINITY_DN1026_c2_g1|g.460 TRINITY_DN1026_c2_g1|m.460 type:complete len:313 (+) TRINITY_DN1026_c2_g1:322-1260(+)

ATGGCTCTCCAAGCAGAGAAGCGCCTCATCCTTTTTATCTTCTCAGTGTTTTGTGAGTCC

ACTTTCATTCTCCATGCTTCCCCTTATACCTTCACCCTGCTTGCCCTCACCTTCGTCTTC

CCCCTCTCCTTCACTATCCTTTCCCACACCCTTGTGACCCACCCGCCTTTCCTCCACCTC

CAACTCTTCACCTTTTCCCAGTTTTTCCTTTTTATTCTCCGCCAATTCGCCTACCTTGTT

GTTCTTTTTGTTCTCTCCCTCAAAATCTCTGCCGCACTCTTCTCCATATCCTCCTCCTAC

ACTTCAAAACCCTTCACCTTCTTGTCCACCTTCACTGCCATGCCTGTGATTTTTCCCCGC

CTCGTCAAAGCCTTCATTTGTGTCTCTGGCATCATGCTTTTGTACTACACCACCTTTTTC

CTGTCCACTTTCGCTCTTCACATGCTAATAGCACCTAGCCTGTATTCCCCGCTCTTTGCC

TTTACCCTTGTCTTTGCCACCATTCTCTTCAGCTTGATCTATAGCTCGATCTCTTTATTG

TGGCACCTTGCAAGTGTCATGTCGATCCTGGAATCTGCATACAGTCTCCCTATGAGGAGA

AAAAGCCATGAATTCCTTAGTTGGATGATGGAAATGGCATATGATTTTGCTTTTCTGCTT

TTTGTGGTCTGTGTAGCTATTGTGGAGGCTTTTAAAGTGTTGGTCTTCAAGGTGCCTGAG

GAGAAAGCAGGCTTAGAGATTGGGTCGGGGCCGAAGGCTTTGATTGGTGGAGTTCTTGTG

GGGTTCCCGATAATGGCAAATCTTCTTGGTTTACTGATTCAGAGCATATATTGTTATGCT

TTCAAGACTTATTATCATGAGGAGAGAAAATGGAGTGCGCTGTGTGATTACCTGGATGAA

TATCTAGAGGAAAATATGCCACCAAAGAGTCCAGTCTGA

>TRINITY_DN10272_c0_g1|m.461 TRINITY_DN10272_c0_g1|g.461 ORF TRINITY_DN10272_c0_g1|g.461 TRINITY_DN10272_c0_g1|m.461 type:complete len:391 (+) TRINITY_DN10272_c0_g1:76-1248(+)

ATGGGTCGCCGAAAGAGCGGGAACAAAAGCCTAGCTTCCTGCAGCAGCAGCCAGAGGAAC

GAATCGAGCAGGAACATCACCGAATTAGATACGAGGATGAGCGCGGTTGGGGAAGAAGAG

GAGGTGGTCATGGCGGAAAGGACCGAAAGCTCTAATCTTGAGGAACTCGAAACCCTAGCT

TTGGTCGGCAGCGATCAGACCAGCGCCGATTACTACTTCGATTCATACTCACATTTCGGG

ATTCACGAAGAAATGTTGAAAGATATTGTCAGGACAAAAACTTATCAAAATGTTATCTAC

CAGAATAACTTTCTCATCAAGGACAAAGTGGTTCTTGATGTGGGTTCAGGGACAGGAATC

CTCTCTCTTTTCTGTGCGAAAGCAGGGGCTAAGCATGTGTATTCAGTTGAGTGCTCTCGC

ATGGCTGACATGGCAAAAGAGATTGTTGAAGCCAATGGGTTTTCTGATGTCATAACAGTT

CTGAAGGGAAAGGTAGAAGAGATTGTACTGCCAGTTGTACATGTAGATGTTATCATCTCT

GAGTGGATGGGTTATTTTCTATTATTCGAAAATATGTTGAACACAGTTCTTTATGCACGT

GACAAATGGCTTGTTGATGGTGGAATTGTGCTACCGGACAAGGCTTCTCTGTACTTGACA

GCAATCGAGGATGCTGAGTACAAGGAAGATAAAATTGAATTCTGGAATAATGTGTATGGC

TTCGACATGAGCTGCATCAAGAAGCTGTCTATGATGGAACCTCTTGTTGACACAGTGGAC

CAAAATCAAATAGTCACAAAAGGCCAATTGCTCAAGACTATGGACATTTCCAAAATGGCT

CCAGGGGATGCTTCTTTCACTGCACCATTCAAACTTGTGGCTGAACGCAATGATTATATA

CATGCTCTTGTGGCTTATTTCGATGTGTCATTTACCAAATGTCACAAATTGATGGGATTT

TCTACAGGACCTAAATCCAGGGCAACTCATTGGAAGCAGACAGTTCTTTACCTTGAAGAT

GTGCTTACCATTTGTGAGGGTGAAGCAGTAGTCGGCACAATGACTGTTGAGCCCAACAAC

AAGAATCCCCGGGATGTTGACATCACTCTGAAGTACACCTTGAATGGACAGCGCTGCCAG

GTCTCTAGAACTCAGCATTACAAAATGCGGTGA

>TRINITY_DN10272_c0_g1|m.462 TRINITY_DN10272_c0_g1|g.462 ORF TRINITY_DN10272_c0_g1|g.462 TRINITY_DN10272_c0_g1|m.462 type:complete len:103 (-) TRINITY_DN10272_c0_g1:1003-1311(-)

ATGGGAAAAGTTGCCATTGCTTCTATTACAAGCGGAAAGAAGAGAAAAAGAGAAGAATGT

CGATCACCGCATTTTGTAATGCTGAGTTCTAGAGACCTGGCAGCGCTGTCCATTCAAGGT

GTACTTCAGAGTGATGTCAACATCCCGGGGATTCTTGTTGTTGGGCTCAACAGTCATTGT

GCCGACTACTGCTTCACCCTCACAAATGGTAAGCACATCTTCAAGGTAAAGAACTGTCTG

CTTCCAATGAGTTGCCCTGGATTTAGGTCCTGTAGAAAATCCCATCAATTTGTGACATTT

GGTAAATGA

>TRINITY_DN10275_c0_g1|m.463 TRINITY_DN10275_c0_g1|g.463 ORF TRINITY_DN10275_c0_g1|g.463 TRINITY_DN10275_c0_g1|m.463 type:complete len:114 (+) TRINITY_DN10275_c0_g1:48-389(+)

ATGAGCTACAACGGTGTCGGGCTCTCCACTCCGAGGGGATCGGGTTCGAGCGGGTACGTG

CAATCCAACAGGTTTCAGGCGAAGCCCAAACCCGCATCAGCCGTCGCTAGCGGCGGCGCC

GCCTGGTCGAAGGCGGTGGAGATAAAGAAACCGATGGCGAAGGGGATCACGGAGCACGAG

AGGAAGCGGAGGATCGAATCGGAGATTCTTGCTCTTTCCGAAACCCTAGCTGGCCAGGGG

TACACCGTGGCCGAGATCGCCGAGAAAGTTCGTGAAGCTAGGAGGGTTCTTGAGGCCGAG

AGCGATGCTGTTGATCATCAATCTGATGTTAAGAGATTTTGA

>TRINITY_DN10276_c0_g1|m.464 TRINITY_DN10276_c0_g1|g.464 ORF TRINITY_DN10276_c0_g1|g.464 TRINITY_DN10276_c0_g1|m.464 type:complete len:520 (-) TRINITY_DN10276_c0_g1:268-1827(-)

ATGGGTCGGAGGGCGGCGGAGCTGCACCGGCGGCCGACGAGGAGGAGGTTGTCGGAGAGG

ATCTGGTGGATACTGCTCTTCTTCTTCCTCGCGCTGCTGCTCTTGTTTATCGTGCACCAT

CAGCACCAAGATCCTTCTCTAATGCCCAACCAGGAGATTAGTACAGAGTTTCTTACTCAT

GAATTAAATTTCACGCAAGAATTATTGAGCACTACTTCTGTTGCACGGCAATTGATTGAC

CAGATCTCACTAGCAAAGGCTTATATTATCATCGCAAAAGAGCATGGCAACCACAATCTC

GCTTGGGAGTTTAGCTCTCTTGTCAGGAACTGCCAAGGACTGCTCTCTGAGGCTGCAGAA

ACAGCAAAACCTGTCGCCATTGAAGACGCTCTACCCATAATTAGCCGTATATCAAAGCTA

ATCTACAAAGCCCAAGGCTTGCATTACGACATCAGCACGACAATTACTACACTGAAGAAA

CATGTTGAGGCTCTTGAAGAGCGTGCAGTTGCAGCAGTGGCACAGAGTGCAGAATTTGGT

CGGTTGGCTACCGAAGCTATGCCCAAAGACCTACATTGCATGACTGTTAGGCTGACGGAA

GAGTGGTTTGAAGATACCTCACTTGAAAAGCTGGCAGAGGAGCAGAAAAACTCTCCCCGT

CTTGTGGACAACAATCTCTACCATTTCTGTGTTTTTTCAGATAATGTGATGTCGACTTCT

GTTCTCGTTAACTCCACAGTCTCAAATGCCGAACACCCTCAGCAGCTTGTCTTCCATATT

ATCACTGATGCAGTCAGTTACAGAGCCATGGCGGCCTGGTTTTTTAAGAACTACTTCAAG

GGTTGCACAATACATGTCAGGAAGATTGAAGACCTGACTTGGTTAGATGCTTCTTACTCT

CCATTGGTGAAGCAGTTATCGGAATCAAGAGAAGCAAAATTTAAGAGCCCAAAACCTGTG

ATTTTGCTGGATCACTTGCGCTTCTATCTACCGGAAATGCTCCCACTTTTAGAGAAGGTA

GTGTTTCTTGAGGATGATGTTGTGGTACAAAAGGATATAACCCCGCTATTTTCTTTAGAC

ATGCATGGTAATGTGATCGGAGCTGTGGAGACTTGTCTCGAGTCATCCCACCGGCTCTAC

AACTATCTTAACTTCTCTAATCCACTGGTAAGTTCAGGATTTGATCCCCAAGCTTGTGCT

TGGGCATTTGGAATAAATGTATTCGATCTGATAGCATGGAAGAAGGTCAATGCTACTGTA

AAGTATCACTACTGGCAGGAGAGGAATGCTGACTGGATGCTTTGGAAGGCTGGGACACTC

TCGGCCGGTCTTCTTACGTTTTATGGGCTGGTAGAGCCACTCGACAGGAGATGGCACGCG

ATGGGGTTAGGATATGAGTTGGATATCGACGAGAGGTTGATAGAGAGTGCAGCGGCAGTG

CACTTCACTGGGGACATGAAGCCGTGGCAGAGGCTTGGTACAAGCAGGTACAAGCATCTG

TGGCGACAGTATGTCAGTTTTACACACCCGTATATTCAAGATTGTTTCATGAGAGCATAA

>TRINITY_DN10279_c0_g1|m.465 TRINITY_DN10279_c0_g1|g.465 ORF TRINITY_DN10279_c0_g1|g.465 TRINITY_DN10279_c0_g1|m.465 type:complete len:389 (+) TRINITY_DN10279_c0_g1:155-1321(+)

ATGGAGGTTGTGGTGCCTGCAGTCAGCGACTTCCACTTCGACAGCAATGCCTCCTCCCCT

TACGTCAGCGCTCCGTCGAGCCCAAAGCGTTTCGGCCGCAGCGGCAGCTCCGATCCCTTC

GACTACTACTACCACTACGCCAGCGCTCCGACCAGCCCGTCTCGCGTCGCCTCCATCCTT

GCATCCTCTTCAGTTCCTTTCGACTGGGAGGAGACGCCCGGCACTCCCAGGCCGCCCAAA

TCGGATGATGCCGATGATTTCGCCTTCGATTTCAGCGGGCAGCTCGAGAAGGGCGTGCCG

CTGCCGGATCTCACCACGGCAGATGAGCTGTTTGAGGAAGGGATGATTCGGCCGCTGAAG

CTGAAGCTGCCGCCGAGGCTGTACAATTTGAGACTGGATGATAATATGACAAGTGTCGCC

CCCTCTCCACGATCGCCAAAATCTCCGAACAGGGCATTATGGTCGCCTCGCGCCAGAGGT

AAAGTCGGCAACGATGATCCCTTCACCGCTGCGATGGTGGAAGCTACAAGGGAGAGAGGG

AGGGAGAAGCGTCCCGCGTCTTCCGTGCACGCGACCTCGATCTCTTCCAGCCGTAGCAGG

AAGGGATCGAGGTCCCTCTCTCCCATGAGGAGCGGAGCTGGAGGATTCTTCACGAAATCC

TCCATCTCCAGCTCCTCTCCAAAAGCCTCCACTTTCGCCGCCGCCGCCACGGTCGCCTCT

GAGTGTCTCAAAACCACCAGCGGTGGCAGCAGGAAATGGAGGCTGAGGGACCTGCTCTTG

TTTCGCAGCGCGTCGGAAGGACGAGTCACCGGAAACAAGAGCAAGGACCCTCTTCGTAAG

TACACCATTATGCCGCCGTCGTCTTCAAACTCCCTGTTTTCGCCTCGGAAGGGTGAGGAT

TCGAGAAATTCGAGCTTCCGGTCGACTGACAGCGGCGGGTCGTCGATCAGGAGGTCGACC

GACGGGTCGATAAGGAGAGCGGGGAGCGGGGTTTCGGCTCATGAGATGCACTACATGTCT

AACCGAGCAGCGTCCGAGGAGAAGAAGAAGAAGACGGCTCTGCCGTTCAATCGGCAGGGG

CTGTTCGGATGCCTTCGGTTCAATCCGGCAGTCCACAGCATTGCCAAGGGTTTTCACAAT

AGCTACTCATTTGGCCGTCGGCAATGA

>TRINITY_DN1027_c0_g1|m.469 TRINITY_DN1027_c0_g1|g.469 ORF TRINITY_DN1027_c0_g1|g.469 TRINITY_DN1027_c0_g1|m.469 type:complete len:408 (-) TRINITY_DN1027_c0_g1:361-1584(-)

ATGGCAACAACTGCTGCTGCGGCTGTCAGCTCCGGCATCTCCGGCCTCCTCCGCTACCGC

CAAAAACAGCAGCCCAAGCACTCGGCCTTCGCCTTCGCCGGGCCCAACGGGCCCGTTGGC

GATGGCGGCAACCTCGTCTGGGGCCGCCAGCTCCGGCCCAACATCCTGCTTCCCTCTCCG

TCCAAATTGACGGAGAGAAGGGAGCTGCTGGTGCGGCCGAGATCGGCTGCGGCCTCGCCT

GCCGAAGGCGATTCCGCCGGTGAAGCGCAGGCGGCTCCGCTTAGTTTTGCGGCGAAGTAC

CCAGCGCTTGTCACCGGTTTTTTCTTCTTCATGTGGTACTTCTTGAACGTGATATTCAAC

ATCATCAACAAGAAGATCTACAATTACTTTCCTTACCCCTACTTCGTTTCGGTGATACAT

CTCGGCGTGGGTGTGGCGTACTGCCTGGCGAGCTGGGCTGTTGGCCTCCCCAAGCGCGCT

CCCATCGATTCCAATCTGCTGAAGCTGCTCATCCCAGTTGCGGTATGCCATGCTCTTGGT

CATGTTACTAGCAATGTGTCATTTGCCGCGGTTGCAGTCTCGTTCACCCACACGATCAAA

GCACTGGAACCTTTCTTCAATGCCGCGGCTTCCCAATTCATCCTTGGGCAACAGATACCT

TGGACGCTATGGCTATCTCTGGCACCCGTCGTGATTGGTGTCTCAATGGCATCTCTCACA

GAGTTATCATTTAACTGGCTTGGCTTTATTAGTGCCATGATTTCCAACATCTCATTCACT

TACCGGAGCATCTACTCTAAGAAAGCCATGGTTGACATGGACAGCACCAACTTGTATGCA

TATATTTCAATCATTGCGTTGATTGTTTGCATACCACCTGCCATAATTTTTGAAGGACCT

CAGTTAATGCAGCATGGATTTAAAGATGCAATTGCCAAAGTAGGAATGACAAAATTCGTC

ACAGATCTCTTCTGGGTCGGACTATTTTATCACCTATACAATCAGTTGGCAACAAACACC

TTGGAGAGGGTTGCACCCCTAACGCACGCAGTTGGCAATGTGCTGAAACGAGTCTTTGTG

ATCGGATTTTCTATAATTATTTTCGGTAACAAGATCTCAACACAAACAGCCATTGGTACC

AGCATTGCGATTGCAGGGGTTGCCATCTACTCGTACATCAAAGCCCAGATAGAAGAGGAG

AAAAGGAAAGCTAAAACTGCGTAA

>TRINITY_DN1027_c0_g1|m.471 TRINITY_DN1027_c0_g1|g.471 ORF TRINITY_DN1027_c0_g1|g.471 TRINITY_DN1027_c0_g1|m.471 type:5prime_partial len:123 (-) TRINITY_DN1027_c0_g1:1395-1763(-)

AACAAACTGCCATTTCTTTTGCCAGCAAATAGTATATCCCCACAAGCCCAAGATAACATT

TCCCTCCCCCTCCCTCTCTCTCAATCCATCCACCTCCACTCAAACCAGCTATACCTTACC

ACACACCTCATAATATCCATTCCCCTCGTTCCCTCCTTATCTTCAAAAACATACCAAAAA

TGGCAACAACTGCTGCTGCGGCTGTCAGCTCCGGCATCTCCGGCCTCCTCCGCTACCGCC

AAAAACAGCAGCCCAAGCACTCGGCCTTCGCCTTCGCCGGGCCCAACGGGCCCGTTGGCG

ATGGCGGCAACCTCGTCTGGGGCCGCCAGCTCCGGCCCAACATCCTGCTTCCCTCTCCGT

CCAAATTGA

>TRINITY_DN10280_c0_g1|m.473 TRINITY_DN10280_c0_g1|g.473 ORF TRINITY_DN10280_c0_g1|g.473 TRINITY_DN10280_c0_g1|m.473 type:complete len:263 (+) TRINITY_DN10280_c0_g1:124-912(+)

ATGGTCTCCGGTCGTCTCCTCCTCCTCCTTTTCGTCCTCTCTCTTCTCTCTTCGGCGATC

TCTCAAGATCTCCCATTTCGCGGTTGCTATTGGACTGAATCTTGCCAAAGCAAATGGTTT

GGAGGATGCAGTACTGGTCACATCATAACTGATCAGTCAGATGACTGCAATGGTCTTTGT

GGAGAATCAAGTTATCCACCATGCCTTCCATTCCATACACATTTTCACTGCTGCAAGCCA

GAAAGCCCAAAGGTAACAGACAGATGCACCAGATGCAAGAATAAGTTGGACTTTGGTGAG

GAGTACATTTGTTGCACTGATTGCTCGGACCCTTACGTAATAGACAAGAATACTAAGTTT

GGTTATTGCAAGACTGGCTCAGAGCTGGCCGTACAACTGAAGCCTCAAGAAACTTTTAAG

TGGGTTGCTGGACCATGGATGAAGTGTTCCTCCCCTTGTGATGGTGGAATTCGTTACCGA

GATGTGGGTTGTTTTGGAAGCATGGAAGATACATCTATTAAACATTATCCAGTCGATGAT

AGCAAGTGTCCTGCAGAGGAAATGCCTGCTAGACAGGAGCACTGTAATGTGAAAAGCTGT

ACTGATCTCAGCATCAGTGATGTGAATGAAAGCAAACAGAGTGGAATGTCTGGTTGGTTG

ATAACATTGCTTGTTCTTCTCGGGCTAGTAGCAGTTGGAGGTCTTGGATTTGCTGGTTAC

ATATTTTATAAGCGGCGAACATCAGCCCCTACGGGATTTGTCTATATCATGCTGGAAGGA

TACTCTTGA

>TRINITY_DN102810_c0_g1|m.474 TRINITY_DN102810_c0_g1|g.474 ORF TRINITY_DN102810_c0_g1|g.474 TRINITY_DN102810_c0_g1|m.474 type:internal len:117 (+) TRINITY_DN102810_c0_g1:3-350(+)

AGATGGTTTCGAAGAAATAACCGATGCTTTGGGCCTTCTCCTTTGGGAGACTGTTCTGGA

GCCGGCCGATCTAAGGCCAACCCATCCTCTACTCCCTCTTCCTTAGTTGATCATGCTCGA

GCGGCCGTTGCATCAACTTCTACTCTACCAGCTCTTTCTCAGCTCACTGGCCCTATTGAT

TCTGATAAGCCACTGCCTCTGACTAGTCTGATTGCCTTTAAGGATGATGTTATGGATGAT

CTTAATGTTCCCATTATTTCTTTTGCTCGTGGCAAAGGGAAGTTGTTAGCCGGTGGGAAG

ATTAAGTCTTCCAAGAAGAAGAAGGGTCGCCCTTACTCTCGGAAAGTC

>TRINITY_DN10284_c0_g1|m.475 TRINITY_DN10284_c0_g1|g.475 ORF TRINITY_DN10284_c0_g1|g.475 TRINITY_DN10284_c0_g1|m.475 type:complete len:504 (-) TRINITY_DN10284_c0_g1:877-2388(-)

ATGGCGGTTGCTGGACTTTACCGGCGAATTCTCCCGTCGCCGCCGGCAATCGAGTTCGCC

TCATCAGAGGGAAAGCAACTGTTCTCTGATGCCCTCCAAAGTGGAACCATGGAAGGATTT

TTCAAATTGATTTCCTACTTTCAAACACAATCCGAGCCTGCTTACTGTGGGTTGGCTAGT

CTGTCTATGGTCTTAAATGCCCTTGCTGTTGATCCAGGAAGAAAGTGGAAAGGTCCTTGG

AGATGGTTTGATGAGTCCATGCTAGATTGTTGTGAACCTCTGGAAAAAGTCAAAGCCGAA

GGAATCACATTTGGGAAAGTTGCATGCTTAGCTCATTGTGCTGGAGCTAATGTTGAAGCT

TTTCGTACAAGCCAAAGCACCCTTGATGATTTCCGTAAACATGTCATCTTATGTACTTCC

TCTGAAGATTGTCATGTCATCACTTCATACCATAGAAAATTCTTCACCCAGACTGGAACT

GGTCATTTTTCGCCAATTGGTGGTTATCATGCTGGACGTGACATGGTACTCATTTTGGAT

GTTGCGCGTTTTAAATATCCTCCTCACTGGGTTCCTCTTCCACTACTTTGGGAAGCGATG

GAGTCAATAGATGTATCAACTGGACATCCTAGGGGGTTTATGCTTATTTCAAGACTTCCG

AGAGCACCATCTCTCCTTTATACCTTGAGCTGCAGGCATGAGAGCTGGGTGAGTACATCA

AAGTTTTTGATGGATGATGTCCCTATTCTCTTGAAGTCAGAGGGCCTAAACAGTGTTCCA

GATGTTCTTTCCCTTATTTTCAAATCTCTACTTGCTAATGCTGGAGATTTCATCAAATGG

GTTGTCGAAGTTAAACAACGAGAAGAGGATGATACACTCTTGAGCAAAGAGGAGAAAGGA

AGACTTGCTGTTAAGGAAGCTGTACTGCAACAAGTTCGTGAAACTGAGCTATTTAAATAT

GTGGCAGACTGGCTATCTTCTATGAGATCATGTTGCCCAAATGCAGCTTCTTTAAACGAT

AAAGATTCATTAACTGAGATTGCAGCTAGTGTAGGTTGCCAAGGGGCTATGTTGTTAACA

GGTGGTCTTGGAGAGAGCAAAGGATTCTGCTGTAGAGCAACATGTGTCAAAAGTCTGAAA

GCCAACGGAGATGAGCCAATAACTGTCGTTTCAGGAACAGCGGTGTCAGGTGGCAGTGAG

CAAGGGGTTGATATGTTGATACCAGTGTCGCCAGCAAAATCAAGCTGTTGCAGTTTGGGC

CCGAGTAGTTGTGTCTTGGTTCACCCGGCAAGTGATGATGTTTTAACAGTTTTGCTATTG

GCATTACCTGATAATACCTGGGTAGGTATTAAAGATGAGAAGCTGTTGTCCGAAATTCTT

GGTCTAGTTTCTGTAGAAACACTTCCAAATGTTCTTCAGGAGGAGGCCTTGCATTTGCGC

AGGCAGCTCCATTTCCTTAGATGTAAGGATAAAGAAGTATGTGATGATCTTGAGTTGTCT

TCAGACCCATGA

>TRINITY_DN10285_c0_g2|m.476 TRINITY_DN10285_c0_g2|g.476 ORF TRINITY_DN10285_c0_g2|g.476 TRINITY_DN10285_c0_g2|m.476 type:5prime_partial len:100 (-) TRINITY_DN10285_c0_g2:106-405(-)

TTGTTTTACAACCGACCTTCAAATAACCTCTCATCTCCAATTTTCCTCTGTCACCGCCGT

CGCCTTCTCTCCGCCACCAACACCGTCGCCGCCGCCTCCTCTCTCCACCACCGTCGCCGC

CGCCTCCTCTCTCCACCACCGTCGCCGCCTCCTCCTCTCTACGCCACCGCCGGTGCTCCC

TATTACTTTAAAAACTGCGATTGTATTTCTCCCCTTGAAACCCTAACCCTAACTCCTTCC

TTCGATCCGATCATTTCAACATCTCTGAGCTCCAACTCCAAGATTACTAGGGCAATTTAG

>TRINITY_DN10286_c0_g1|m.477 TRINITY_DN10286_c0_g1|g.477 ORF TRINITY_DN10286_c0_g1|g.477 TRINITY_DN10286_c0_g1|m.477 type:complete len:366 (-) TRINITY_DN10286_c0_g1:685-1782(-)

ATGGAGGAAGAGAAAGCGGCAGCGTACTACGACGAGCTCACGCGCAAGGGCGAGGGCGCT

GCCAAATTCAAGCAAGGCCTCGGCTTCTCCTCTTCCTCCTCCGCCGCCGCCTCCGACGCA

TTTACCAAAAAGCCCTCGCCTTTTTCCCTCTCCAGCTTCGTTCGCGCCTCTGGCAACAAA

GGCGGAGATTCTTCGGAAAAAATCTCAGCTTTCTCTTCTTCTTCTTCTTCGCTCTCCAAC

TTCGTGAGAGCGTCTAGCCCTGAAAGGGACGATCGAGACGATAGAGCTCGCGTTCTTGGT

TGCATCCAGGATAAGCTCGGCAGCCGGCGGCGGTCGCCGGAGGGTGATAGGGAGAGGAGG

AGAGAGAGGCGCGATGAGAGAGAAAGAGACGATGCGAGATATTCTTCGAAAAGGAGTAGG

GAATCTAGGGATAGTGATAGAGAGAGGAGGCGGCGGCGGAGTCGGAGCAGGAGCTATTCG

CCGCCTAGGGTTTCGCATAGCAGGGATCGGGATCGAGATAGAGATAGAGAGAGGGATCGC

AAAAGGGACAAGGATCGGAGTTCAAAGGAGAGCAAGAGCGATAGAGAAGTAGATTATTCG

CAACTAATTGAAGGCTACTCCAAAATGACACCAGCTGAAAGGGTTAAGGCAAAGATGAAG

CTTCAGCTCTCACAAACTGCTGCGAAGGACACAACTATGGGTATGAGTTCTGGGTGGGAA

CGATTTGATTTCAACAAGGATGCCCCTCTTGATGATGACGACGAGGAAACCGAAGTGGCT

GAAGATGACATCTCGGTTGTCAAAAACATAGGGAGAAGCTTTCGATTTTCTGCTGTGGAG

GCAAGGCGTGAGGAAGAACTCAAGGCTGCCCATGACCAGGCAATGTTTGGAGCAGCATCA

GCAGCAGCATCTTCACCAGTCAACCTTTCACATGAAACCTTTGGATCAGCTGAGGAGAGT

GAGAACAACACAGCAGTCGGTAATAATGGAAATGATCTCACCACCACCCCGTTCTTAAGC

GACAAGGTGCTTTCTATGCAACAAGGTTCTTGGCGAGACAGAGCGCGGAGGTTTCAAAAT

GAATCAGAAAAGAGTTGA

>TRINITY_DN10289_c0_g1|m.478 TRINITY_DN10289_c0_g1|g.478 ORF TRINITY_DN10289_c0_g1|g.478 TRINITY_DN10289_c0_g1|m.478 type:complete len:412 (+) TRINITY_DN10289_c0_g1:201-1436(+)

ATGAATGCACTAGCTGCAACCAGTAGGAATTTCAAGCAGGCAGCTAAGCTGTTGGGATTG

GACTCTAAGCTGGAAAATTGCTTGCTCATCCCTTTTAGAGAGATCAAGGTTGAGTGTACG

ATTCCCAAAGATGATGGCACTTTGGCATCGTTTGTTGGATTTAGGGTGCAACATGACAAT

GCTAGAGGCCCTATGAAGGGTGGAATCAGATACCACCCTGAGGTTGACCCAGACGAGGTA

AATGCCTTAGCACAATTGATGACATGGAAGACAGCTGTAGCAAACATTCCATATGGAGGT

GCGAAAGGGGGGATAGGATGCAGTCCAGGGGATTTGAGCTCATCTGAGCTTGAGAGACTC

ACCCGAGTTTTTACACAAAAGATACATGATCTCATTGGCATTCACACTGATGTTCCGGCC

CCTGATATGGGAACCAATGCACAGACAATGGCTTGGATACTAGATGAGTACTCAAAATTC

CATGGCTACTCTCCTGCAGTTGTAACCGGAAAACCAGTTGATCTGGGTGGATCTCTGGGT

AGAGATGCGGCTACTGGAAGAGGAGTCCTATTTGCAACAGAAGCACTTCTGGCAGAATAT

GGAAAGAGCATTTCAGGCCAACGATTTGTTATACAGGGGTTTGGCAATGTTGGGTCTTGG

GCAGCTCAACTTATCAGTGAATCTGGTGGGAAAGTTGTTGCAGTCAGTGATGTCACAGGA

GCAATTAAGAACAGCAACGGTCTTGATGTAGAGAAATTACTTAAGCATTCTGTAGAAAAT

CGCGGTATCAAAGGTTTCAGTGGCGGTGATGCACTTGATCCCACCTCATTACTGACTGAA

GATTGTGATGTTCTCATTCCTGCTGCTCTCGGTGGTGTTATCAACAGGGAAAATGCAAAT

GATATTAGAGCCAAGTTCATCATTGAGGCAGCAAACCATCCAACAGACCCAGAGGCTGAT

GAGATTCTAGCAAAGAAAGGTGTTCTTATTCTGCCAGACATACTTGCAAATTCGGGCGGA

GTTACGGTCAGTTATTTTGAGTGGGTTCAGAACATCCAAGGGTTCATGTGGGATGAAGAG

AAGGTCAATTCCGAGCTGAAGACATACATGACCAAAGGCTTCAAAAACGTGAAGGAGATG

TGCAAGTCCCACAACTGCGATCTCCGCATGGGAGCCTTCACCCTTGGAGTCAACCGAGTT

GCACGTGCAACTATCCTCCGAGGTTGGGAAGCATAA

>TRINITY_DN10289_c1_g1|m.481 TRINITY_DN10289_c1_g1|g.481 ORF TRINITY_DN10289_c1_g1|g.481 TRINITY_DN10289_c1_g1|m.481 type:complete len:235 (-) TRINITY_DN10289_c1_g1:413-1117(-)

ATGGCAGCGGTGACCAGACGCGGCATCACATCCCTCCTCTCTCGAACGCTCCTCTCCTCT

CCCGCCCTCAAACCCCCTCGCTTCGCCGTCGCCGCCCTCAACCGGGTCGGGCTCGCCGCC

TCGGCCGGCCCTGCCCGGTTCAAGACGACCTCCGGTTCGGGCTACTCGCCGCTCAACGAC

TCGTCGCCGAACTGGAGCAATCGGCCGCCAAAGGACACGATCATGCTCGATGGCTGCGAC

TACGAGCATTGGCTGATCGTGATGGAGTTCCCTCAGGATCAGAAGATGTCGGAGGAGGAG

ATGGTCAACGCTTATGTCAAAACCCTAGCAAATGTCGTCGGAGATGAGGAGGAGGCAAAA

AAGAAGATTTACTCTGTGTGCACCACAACGTACACAGGATTTGGAGCACTGATATCAGAA

GAGCTTTCCTATAAAGTTAAAGGATTACCTGGAGTTCTGTGGGTGTTGCCTGATTCTTAT

CTAGATGTTCCCAACAAGGACTATGGAGGGGATCTTTTTGTAGACGGAAAGGTCATCCAC

AGACCACAGTTCCGATTCAGTGAAAGGCAGCAGACTAGGAATCGTCCTCGTCCTCGCTAT

GATAGGCGCAGAGAAACTATGCAAGTTGAAAGAAGGGAACCAATACGGAGGGGTTCGTGG

ACTCAAGAGAACCACGAAGTTCCTGGGGATAAGCCTGAGAACTGA

>TRINITY_DN1028_c0_g1|m.483 TRINITY_DN1028_c0_g1|g.483 ORF TRINITY_DN1028_c0_g1|g.483 TRINITY_DN1028_c0_g1|m.483 type:5prime_partial len:745 (+) TRINITY_DN1028_c0_g1:3-2237(+)

GTTGAAGAACACAATGGCAATTTAAGGAATGTAGTGGTGGAAAATGGATCAAATTCTCAC

CTTAGTTATGATAAAAAGAGTGGAAATAGATTCACGAGAAATGACAACGAAATCCAAGAA

CAAGCAGAGCATACACCAGGAGAATCAAGCACATTCGTTGTTGAAGAGCTTGATGTTGAA

AATGGTACTGTTGAAGCTAAAGAGTTCAATGGTCACAAGGATGTTCCTGTTGAAGAACAC

AATGGCAATTTAAGGAATGTAGTGGTGGAAAATGGATCAAATTCTCACCTTAGTTATGAT

AAAAAGAGTGGAAATAGATTCACGAGAAATGACAACGAAATCCAAGAACAAGCAGAGCAT

ACACCAGGAGAATCAAGCACATTCGTTGTTGAAGAACTTGATGTTGAAAGAGTTTTAAGG

GAACAAGAGACTTATGATTTGATCTGCCCTAAATGCCGCTCGTGTATTACCAAAAGGGTA

ATCTTACGCAAAAGAAAAAGGGCACCAAGTCAAAGTGGATTACCACCTCCAAAAGCCCAA

AATGTGTCTGGAGCTAATCCTCTTCCAACTGAACCGCAAGATCCCGATGTTACAGCAACA

TTCGGATATTTAGATACAGCAGTATTCAGATGTTTATCTTGCTTCAGCTTTTTCATCCCG

ACAGAGACTGGATGTAATATTCTCCGTGTTTTTTGGAAAAAATCCGCAAGTGATGATACA

CAAAGCTCAGAAAATAAACCTGCAAGGACAGGGAATTGGGTCACTTCTTGTTTCGGATCA

GATTCAAGCCTAAATGAAGATACGGAAGCTGGTTCATCAACTGGAGTATCAAATTCTATT

GGCGGAATAACGGGAAATCGGGCTCAAGGAGACGAGCATAAAAAACCAGCAATAACTGTA

TCAGAAGGTCCGTCGACGGGAGTATCAAATTCTAATGGAGCAATGGGAAATCAGGCTGAA

GGAGAGAAACAAAAAAATTCATCAACCGCTGTATCAGATGGTTCATCAACTGGGGTATCA

AATTCTAATGGCGGAATAACAGGAAATCAGGCTCTAGGAGACGAGCAAAATAAACCAGCA

TTAACTGTATCAGAAGATGTCTCATTGCTGCAAGAAACTAGAGATCCATCCATGGGATCA

AAGGAAAACTCTCCACCTATGTCCACACCAGGAATTGCAAATCATGGAGAAAACGGAACC

CCCATTGGAAAGAAGAATGTTGGTTCATCAACTGGAGTATCAATTTCTAATGGTGGAATA

ACAGGAAATCAGGCTCAAGGAGACAAGAAAAAAACCCCAGCAATAACTGTATCAGAAGGG

AACGCTACTGAGAAGCCTGGTTTCGTCGTTAAACCTCCATCTCTGGGAGGAATTTCTCAA

ATCAATGGAAATATTAACAATTCTGTAACACCAGTAATTGGTTTTCTTCCTCCAGGAAAT

AATATTGGTAGTTCTGTTGGCGTGGTTAAACCTACACGACCAGTAGAGAGTACCAACAGG

AATCCAAATATTGGTGGTTCTGAGATACCAGCTACTGGTGATGCTATATTAGATGTGGGT

GTGCAAGAAATAGAAGAATCTCCAAGAGACAATGACTGGGATGTGCTTAAAAGCATTGTG

TATGGAGGCTTGATCGAATCAATTACAAGTCTTGGTGTGGTATCATCTGCAGCAGGAACT

GGTTCTTCCACACTGAACATATGTGCCTTGGGGTTAGCAAATCTGATTGGCGGATTGTTT

GTGCTTGCTCATGATATTTCTGAAATGAGGAGCACACACGATGCTGGAGCCGCAAACCAA

AGGGATGAACAAGCGGGTCGTTACTGGGAGAAGCTTGGACAAAGAAAACATTTCCGGCGG

CATACATTCTTGGCTATACTATCATACATTATCTTCGGATTACTTCCTCCTGTTGTTTAT

GGCTTTGCGTTCAGAGTGAGTGACAACAAGGAATACAAGCTCATAGCTGTTGCCGCGACT

TCTATGCTGTGCGTTGCTCTGCTGGCAATCGGAAAGGCCCATGTCCGGCCACAGAAAGAT

TACATCGTATCTCTCTTCTACTACCTTGGAATCGCGGTGCCAGTCTCCGGCCTGTCCTAT

GTGGCCGGAGTGATGATCGATAGGCTCTTGGTGGAGCTCGGTTTGTTCAACCACAGTACT

GTTTCCCCTTCACCACCTTCTTCCATTGACCTCTTGAAATTTGGCTCCGGAGGTTCAGCG

TGGGCATCTATTTAA

>TRINITY_DN10291_c0_g1|m.486 TRINITY_DN10291_c0_g1|g.486 ORF TRINITY_DN10291_c0_g1|g.486 TRINITY_DN10291_c0_g1|m.486 type:5prime_partial len:118 (-) TRINITY_DN10291_c0_g1:2122-2475(-)

AGGGAGCCCCTTAATTCTCTTAGAGTGAAAGTATTAATGGCCCTCCGGTGGCTCCTCGGC

GTCGCGGCCACCCAGATAGTAGGCCAGTCGACGCCTGATCAGGGCGAGTGCTCAGACGAA

GCTGCGGAGAAGAAGAAGAAGGCGGTGGCGGAGGTGAAGAGAGCGAGCTTTGGGGACGGA

GAAGGGTTCCGGATGCCCTTGCATTACCCGCGTTACAAGAAGGAGGACTATGAGAAGATG

GAGGAGTGGAGGGTGGACATGTTGCTAATGCAATATGGATTGGCTTGTGAGGGATCACTT

GATGAGAAGAGGGCTTTTGCTATGGGCACCTTCCTCTGGCCTGACCAACTCTAA

>TRINITY_DN10291_c0_g1|m.485 TRINITY_DN10291_c0_g1|g.485 ORF TRINITY_DN10291_c0_g1|g.485 TRINITY_DN10291_c0_g1|m.485 type:complete len:196 (-) TRINITY_DN10291_c0_g1:176-763(-)

ATGGACTTTCTTATGTGCTGCAGTGCGAAGCCATGCTCACCGTCGGCACGACATCTTCAG

AAGAGAGAAGAAGTTCACCGTAGAGAGAAGATGCGTGAAAATCGACGAAAAATGGCAGAT

TATAATCTTGGTGAGTACGCGATGGAGGAGGACCGCTGGAGTACCAACGTACGGCCACTC

TTTTCCGATGAAGTTGCTGCGGAGGTCGAAAGTAGGAGTCGAGAATCATATTCCGACTAT

GATAGCACTGTGGACGTCGAAAAAGAGTTGCTACATGTAAAAGAAGTTGTCAGACGTCTT

AAAGCAGCATACAATGGAGTATTGATCGAGGACAACAATGAGGATGGCGATCAACAACTG

ATCGAGGGAGACGATGAAGATCTTGACGATAGGGGTGGAATAGCTTCGTCGTCTCAAGCA

TATGAGACGGCGGAGGAAGAATATGGCTCGAACGACAACTGCGAGCAGAGGACTGCGACG

ATGGATCATCTTCAAGTTGTACGGTCTTGTATTAGAACGACCTACGATGTTCGTGTCTTA

GCTTTTTCTTTCGGTTTGTTTTTTCTTGCTTTTTTGTTTAGCAGGTAG

>TRINITY_DN10292_c0_g1|m.487 TRINITY_DN10292_c0_g1|g.487 ORF TRINITY_DN10292_c0_g1|g.487 TRINITY_DN10292_c0_g1|m.487 type:complete len:149 (+) TRINITY_DN10292_c0_g1:118-564(+)

ATGGAGCAGTCCTTCATCATGATCAAGCCTGACGGCGTCCAGAGAGGCCTGATTGGAGAG

ATCATTAGCCGATTTGAGAAGAAGGGTTTCTACTTGAAAGCAATGAAGCTTATCACTGTG

GAGAAATGTTTAGCTGAGAAGCACTATGAGGACCTTTCCTCAAAGCCCTTCTTCCCTGCG

CTCGTTGACTACATCGTCTCTGGCCCAGTGGTTGCCATGGTTTGGGAGGGGAAGAATGTT

GTTTTGACTGGCCGCAAGGTTATTGGTGCCACCAACCCAGCAGAGTCTGCTCCTGGTACC

ATCCGTGGTGATTATGCCATTATGGTTGGCAGGAACATTATTCATGGAAGTGATTCAGTT

GAGAATGCAAGGAAGGAGATCGCACTGTGGTTCCCTGAGGGCATTTCTAACTGGCAGAGC

AACCTCCACCCCTGGATCTATGAGTAA

>TRINITY_DN10293_c0_g2|m.488 TRINITY_DN10293_c0_g2|g.488 ORF TRINITY_DN10293_c0_g2|g.488 TRINITY_DN10293_c0_g2|m.488 type:internal len:111 (+) TRINITY_DN10293_c0_g2:1-330(+)

CTAGCCCTAGCAGCCCAGCAACGCCGCTCCAGACTCCCGCAGCCGGCAGCCCCGCGTCCC

GCTCCAGACTCCCGCAGCCTCCGCGTCCCGCAGGCCCGCTTCAGCCTCCCGCTGTCCCAA

AGCCCCGGTCCCCAGGCCCAGGCTCCCCACTCGAGGCGGCCGTGGCCCGCCCTACTTCCT

CTTCTCAACCCTAGCGCCGCAAGCAGGCTCGCCGCAGCGCCGCTCACAGCCGAGGATCGC

CGCAGCGCTACTTTCTCCGGCGCATATTGTGTTGTTGCAGGTGAGTCAGCTGGCAGGCAG

GTTCCAATTGCTTTCCTTGAGAGGGTTAAG

>TRINITY_DN10297_c0_g1|m.492 TRINITY_DN10297_c0_g1|g.492 ORF TRINITY_DN10297_c0_g1|g.492 TRINITY_DN10297_c0_g1|m.492 type:internal len:123 (-) TRINITY_DN10297_c0_g1:1-366(-)

CAGTCCATCGACCGGCTGATCTCGCCGGCCACATCCGACGACGAGAAGACATCCCTCCTC

GCCGCCCTCGAGTCCCGCCTCGCCCAAGGATTCGACCGCGAATTCCAAGATCTGCTGCTG

AGATCCAACGCCTTCGCCGCCGTCGAGTCCCTGCTATCCCGTCCGGACTCGTCCAAGAGC

GTCCGGGAGCGATCCGCCGCCGCGATCCTCGCCTTCGTCCGCTTCAACAAGAACGTGTTC

GTCGGCGAGGTCCTGATGGGCCCCACCGTCAGATCCCTCGTCTCCATCGCCTCTGCCACC

TCCCTCCGCGTCCTCAATGGGCTGATCGCGTCCATCCGCAGCCCGCTGGTGGACCAGCTG

CACGCC

>TRINITY_DN10299_c0_g1|m.493 TRINITY_DN10299_c0_g1|g.493 ORF TRINITY_DN10299_c0_g1|g.493 TRINITY_DN10299_c0_g1|m.493 type:complete len:126 (-) TRINITY_DN10299_c0_g1:232-609(-)

ATGGCATCATCAGCAGTAAAACCTGGTGTGCTGGTTGCTTTACAGGAGCTAGAGCCTTCA

TCCCCATTCTTCAAGCATGGGACGTCTCTTCGTGTTATTGGGAAGCTACAAGCACATGGT

GTGGAAACTGACATTGCAATTATCATTGACGGGGGTGTTACCCTCAAGATCGACACCCAG

CACCTGACTGACCTTAGCCTTCGTGTAGGTTCTATTTATCAGTTCATCGGGGAGCTGCTA

ATTCAATCTGACAACGATGCAACCTTGCAAGCGCGAGTTGGCAGGAATGTGGATGGCATT

GATCTCAACCTATACCACCAATCTATGAAGCTTCGTCGACAATTTGAAGCTGATATTATG

AAATCAAGGGCAACATAA

>TRINITY_DN1029_c0_g2|m.495 TRINITY_DN1029_c0_g2|g.495 ORF TRINITY_DN1029_c0_g2|g.495 TRINITY_DN1029_c0_g2|m.495 type:complete len:599 (-) TRINITY_DN1029_c0_g2:318-2114(-)

ATGTCACTTTTGCTCTCGGCCTCAGCAGCCACCGCCACCACCAAAGCTGCTCACCTCTCC

CTTTATTCTCACAGGCAGTGCCTTCAGTGCCGCCAGTCGGATAGAGTTTTTTTGAACTCA

GTTCGATCACGGTTTGCTTCGAGCATTAGGGTTTCAATCAGGGCAATGGCAGAGACCGAG

ATTCTGATGCCAAAATCTCATATTTCAGCCGCTGGTTGCAAGCAAGCCCTTATATCATTG

TCAGACAAACGTGATCTGGCTTTCCTTGGAAATGGACTTCAAAAACTAGGGTATACTATT

GTTTCAACTGGAGGAACTGCATCTGCACTAGAAAATACTGGAGTATCGGTAACCAAAGTT

GAAGAAATTACTCATTTCCCTGAAATGCTTGATGGTAGAGTGAAAACTTTGCATCCTAGC

ATACATGGTGGTATTCTTGCTAGAAGAGATCAAGACCACCACATGGAAGCCTTAAATACA

CATGGCATTGGGACATTTGATATAGTGGTGGTCAACTTGTATCCCTTTTATGAAAAAGTG

TCTTCTGCTGATGGAATTACATTTGAGGATGGCATTGAGAATATTGATATTGGTGGCCCT

ACTTTAATCCGGGCTGCTGCTAAGAATCACAAGGATGTTCTGGTTGTGGTGGACCACAAT

GACTATGCTCCACTCCTGGAATTTTTGAAAAGAAATCAAGATAACCAGCAGTTCCGCAGA

GAGCTGGCATGGAAGGCTTTTCAGCATGTTGCTTCCTATGATTCTGCAGTTTCAGAGTGG

TTATGGAAGCAATCGGCTGGAGGTGATAAGTTTCCTCCTAGCTTTACGGTGCCTCTCTCA

AGGAAATGTACACTTCGATATGGTGAAAATCCTCATCAAAAAGCTGCATTTTATGATGAT

AAGAGTCTCTCTGTGGTGAATGCTGGCGGTATTGCCACTGCAATACAACACCATGGGAAG

GAAATGTCTTACAATAACTATTTAGATGCGGATGCTGCCTGGAACTGTGTGTCAGAATTT

AAAAACCCTACCTGTGTGATTGTGAAACACACAAATCCATGTGGTGTAGCATCGAGGCAA

GATATCCTTGAAGCATATAGATTGGCTGTGAAAGCTGATCCTGTAAGTGCCTTTGGTGGC

ATAGTTGCCTTTAACACAACAATAGATGAGGACCTTGCAAAAGAGATCCGGGAATTTAGG

AGCCCAACAGATGGTGAAACAAGAATGTTCTATGAGATAGTTGTCGCACCAAAATATACC

GAGAAAGGACTAGAGCTCCTCCGTGGAAAGTCAAAGACTTTGAGAATCCTTGAGGCGAAA

AAGAGTGAGAAGGGAATGCTCTCTCTAAGACAAGTTGGAGGCGGATGGTTAGCACAGGAC

TCGGATGATTTGACCCCAGAAGATGTCAATTTCAACGTGATGTCAGAAAAAATTCCAGAA

GAAGAAGAGCTTCATGATGCAGAGTTTGCATGGCTTTGTGTCAAACATGTCAAAAGTAAT

GCCATAGTAATAGCCAAGAATAACTGTATGTTAGGTATGGGGAGCGGACAGCCAAATAGG

GTTGAGAGCTTAAGAATAGCTTTCAGGAAAGCTGGAGAAGAAGCAAAAGGCGCTGCTTTG

GCTAGTGATGCATTTTTCCCATTTGCTTGGAATGATGCGGTCGAAGAAGCATGCCAGAAA

GGCATCGGAATTATCGCTGAACCCGGCGGTAGCATCAGAGACAAAGACGCCGTGGATTGC

TGCAACAAGTACGGCGTCTCTCTCGTGTTCACCAATGTGAGACACTTCAGGCACTGA

>TRINITY_DN102_c0_g1|m.497 TRINITY_DN102_c0_g1|g.497 ORF TRINITY_DN102_c0_g1|g.497 TRINITY_DN102_c0_g1|m.497 type:complete len:377 (-) TRINITY_DN102_c0_g1:1048-2178(-)

ATGTATCCTCATCTCTCAAGTAGTATTAATCTCCAACAAATTCAAACTAGTACTCGTACA

TCCACTCCATATTTTTCTTCGATAATGGCTTCTTCGTTTGCTCTCAACTCTCCCAACTCT

ACCATCAAAGAGCTTTGTAGCTCACGCACACTCTCCTCTCTTCCATCTACTCACACTCTG

AAAGATTCAGGAGACGGTGAAGTCGTGGAGGATGTAGATATTCCAGTCATCGATTTCTTT

AAGCTGACCGAAGGAACACCTGAGGAGCGGGCTCAAGTTGTTCAGGATCTTCGCAGGGCA

TGCGAAGAGTGGGGATTCTTTGTGCTTGTGAATCATGGCGTACCGGAAGAATTGATGAAT

TCTACGATGACGACACTCAAAGGGTTCTTCGATCTGCCAGCGGAAGAGAAGCGAGAGTAC

GCAGACACGCATCCGATGAGTCCTATTAGATATGGAACCAGCTTCAATACGAAAGTGGAG

GATGTCAAATATTGGAGGGACTATGTTAAGATTATAACACACCCGGAGTTTCACAGCCCT

GCAAAGCCCCCAGAGCTGAGAGAAGTATTGCAAGAGTATGCAGCGTCCACAAGAGAGCTC

GGAAGGAAACTGCTTGGCGGAATATGGGAGAGTCTAGGACTAGACGAGAACTACATGAAC

GGGGCTTTGAACCTCGACTCCCTGTTCCAAATTGTTGTTTGCAATCTCTATCCTGCATGC

CCGCAGCCGGAGCTGGCCATGGGGCTGCCCCCTCACTCCGACCACGGCCTCCTCACTGTC

CTCTACCAGAACGACGTCAATGGCCTTGAGTTGAAATATAACGGCAAATGGGTCCGTGTC

AAGCCCTCCCATAACTCTTACCTTATCAACACTGGAGATCATATGGAGATAGTTAGCAAC

GGCAAGTACAGGAGCATTCTGCACCGAGCAGTCGTGAATGGAAAGAACACGAGGATGTCG

ATTGTATCTGTTACAAGTCCGTCCCTGGACACGGTTGTTTCCCCGGCTCCCCAGCTCGTG

AGCAGCGAGAGCCCAGCAACGTTTCGTGGGATAAAATATGGAGAGTTCATGGAGTACCAA

CAGAGCAACAAACTGAAGGATAAATCTGTTCTGGATATGTTGCGACTTTGA

>TRINITY_DN102_c0_g2|m.499 TRINITY_DN102_c0_g2|g.499 ORF TRINITY_DN102_c0_g2|g.499 TRINITY_DN102_c0_g2|m.499 type:complete len:160 (+) TRINITY_DN102_c0_g2:276-755(+)

ATGGGTTGCTTGATTTCGATGAGTTTATTAGCTTGGTCCGGGGGGGCAATGGACTACTAC

AACATAGCCGAGATCAGGCGAATCTTCACCCGTTTCGACTCCAACAGCGACGGCAAGATC

TCCCCGACCGAGCTGCGCTTATTTATGTGGAGCGTTGGCGAGGAGCTGGTGCCGGAGGAG

GCGGAGGATGCGGTTCAGTCTACTGATTTGAACCGCGACGGGCTGTTGGATTTTGATGAG

TTTATTAGATTGGTTCGAGGAGAAGGTGGTAAAAGCGAACAGGATAGGGAATTGAGAGAG

GCATTCCAAATGTATTTGGCGGAGGGCGAGGGGTATATTACGGCAAAGAGCCTGAAGCGG

ATGCTTAGCTTGCTGGGGAACTCGAGGAGTTTCCAGGAGTGCAAGGATATGATATGCAGG

TTTGATGCGGATGGGGATGGTGTGATTAGTTTTGATGAGTTTAAGACTATGATGTGTTGA

>TRINITY_DN102_c0_g2|m.500 TRINITY_DN102_c0_g2|g.500 ORF TRINITY_DN102_c0_g2|g.500 TRINITY_DN102_c0_g2|m.500 type:complete len:141 (+) TRINITY_DN102_c0_g2:59-481(+)

ATGGGTTGCTTGATTTCGATGAGTTTATTAGCTTGGTCCGGGGGGGCAATGGACTACTAC

AACATAGCCGAGATCAGGCGAATCTTCACCCGTTTCGACTCAAACAGAGACGGCAAGCTC

TCCCCAACCGAGCTCCGGCTATTTATGTGGAGCGTCGGCGAGGAGCTGGTGCCGGAGGAG

GCAGAATATGCGGTTCAGTCTACTGACATGAACGGCGATGGGTTGCTTGATTTCGATGAG

TTTATTAGCTTGGTCCGGGGGGGCAATGGACTACTACAACATAGCCGAGATCAGGCGAAT

CTTCACCCGTTTCGACTCCAACAGCGACGGCAAGATCTCCCCGACCGAGCTGCGCTTATT

TATGTGGAGCGTTGGCGAGGAGCTGGTGCCGGAGGAGGCGGAGGATGCGGTTCAGTCTAC

TGA

>TRINITY_DN102_c0_g3|m.501 TRINITY_DN102_c0_g3|g.501 ORF TRINITY_DN102_c0_g3|g.501 TRINITY_DN102_c0_g3|m.501 type:complete len:146 (+) TRINITY_DN102_c0_g3:41-478(+)

ATGAGAAACATTCAGTGTTCCCACGGATTCGAGCGAGTAGTCTATCGCTTCGATGAGGAT

GGCGATGGTAAGATATCACCGGCCGAGCTGCGGTCCTGCATGAAAGCCATCGGCGAGGAG

CTGTCTTGGGAGGACGCGGACGCCTTGGTTAATTCTGCGGACTCGAATGGGGACGGGCTG

CTGGAGCCGGAGGAACTGTTGAAACTGTTGGATGAACAGGATGAAGGAGAAGAGGAGGAT

AAAGAACTGAGGGAAGCATTCGGGATGTACGAGATGGAGGGCGAGGGATGCATCACGGCC

AAGAGCCTGAGGCGGATGCTGAGCCGATTGGGAAACTCGAGAGATGTGGAGGAGTGCAAG

GCTATGATCTGTAGGTTTGATCTTGATGGAGATGGAGTGCTCAGCTTTGAAGAGTTCAGG

ACAATGATGATGCTTTGA

>TRINITY_DN103031_c0_g1|m.502 TRINITY_DN103031_c0_g1|g.502 ORF TRINITY_DN103031_c0_g1|g.502 TRINITY_DN103031_c0_g1|m.502 type:3prime_partial len:111 (+) TRINITY_DN103031_c0_g1:8-337(+)

ATGACACAGAAAACACTGAGTTTCAAATTATTCTATCAAGGAAACCTTTACCGCAGAACA

TTTTCTTCTTCAGTCACTTTTCAACACATTCGCAACGATATCACGAACACCATTACACAC

ACAGATCTCTTCTACTTACAATACTATGATAATGAAGGTGATTTAATTTCTCTCTCTTCC

GACATGGAGCTCGAAGAATTGGCCCAACTCTCTCAGGACTCTCCCATAAAAATTATGATT

AAAGAAGGCATCTTCCAACTTGAAGGCTCTTCTTCTTCACCCCAAATAAATTCGTTCACT

GTGTGTCATTCTCCCCTACCACCACCTCCG

>TRINITY_DN10303_c0_g1|m.503 TRINITY_DN10303_c0_g1|g.503 ORF TRINITY_DN10303_c0_g1|g.503 TRINITY_DN10303_c0_g1|m.503 type:5prime_partial len:327 (-) TRINITY_DN10303_c0_g1:236-1216(-)

GAATCAATCAACCAAAAGAAGTTAGAATTAAAAAAAAAAAAAAAAGGAAAACGAAAAAGA

AAAAAAATAAATCAAGATTTCTCTTTTCATCAACGAGAAGAGAGAGAGATGGTTCGTCTG

TACGCCGTCAAAGGCCGCAAGAAGCGAGCGAGAGTCGAAGAAGAACCGCAACAGCAAGAG

GCGGAAGCAGGAGACGAAGTGTTTGCAGAGGAGAACAAAGAAATCACTGCAGAGGGAGAG

CGCGGCGCGAACCCGGAAGAATCGACGGTGGAGGACGAGAGGAAGGCGGCGGAGACGATC

GATCGGATGCCGGGGATCCCGATCGCGGGAGGGATCCAGGAACCAGGGAAGAAGCCTGGG

GTCATCTTCGTGCTCGAGAGGGCGTGCCTTGAGGTCGCCAAGGTTGGAAAGACTTTCCAG

ATCTTGAACTCAGATGATCATGCCAACTTTCTCAAGAAGCAGAATCGTAATCCTGCTGAT

TACAGGCCTGATATCATACACCAGGCCGTCCTTGCTATACTGGATAGTCCCCTAACAAAG

GCAGGGAGGCTACAGGCTCTATATGTAAAAACAGAAAGGGGAGTGCTTTTTGAAATTAAA

CCACATGTTCGTATCCCACGAACATTCAAGCGATTTTGTGGTCTCATGTTACAGCTACTT

CAAAAATTGAGTATTACTGCCATGGGAAAACGTGAGAAGCTTCTCAATGTCATTAAAAAT

CCTGTAACCCGTCATTTGCCTGTTAATTCACGAAGGATAGGCCTTTCATATAGTTCACCG

AAAGTGGTTCAACTGCGAGATTATGTAGCTGCTGCCAGTGATGATGCCAACCTTGTATTT

GTGGTGGGTGCTATGGCACATGGAAAGATCAATAACGATTATACAGATGACTTCATATCA

ATTTCTAATTATCCGCTGAGTGCAGCTTGCTGCTTAGGCCGGATATGCAATGCTTTGGAA

CAGAAATGGGGCATACTGTAA

>TRINITY_DN10306_c0_g1|m.507 TRINITY_DN10306_c0_g1|g.507 ORF TRINITY_DN10306_c0_g1|g.507 TRINITY_DN10306_c0_g1|m.507 type:complete len:149 (+) TRINITY_DN10306_c0_g1:652-1098(+)

ATGATGGAGGATATTTGTAGTCATAAGGAGGATATTTGTAGTCATCAGGAGGGGTTGGTG

ATTTATAATAGTAAGGAGGTGGGGGATACTTGTAAGCATAAGGAGGATTTGAATAGTAAT

ATGGAGGAGGAGGTGATTTGTAACTGTAGGCAGGTGGTGGTGGTGACTTGTAGACATAAG

GTGGTGGTGGTGACTTGTAGACATAGGGAGGAGGCGGTGATTTATAATAATAAGGAGGTG

GTGAAGGATACTTGTAGGCATAAGGAGGATTTGCATAGTAGTAAGGAGGGGGAGGTGATT

TGTACTTATAGGGAGGTGGTGGTGGTGACTTGTAAACATAAGGAGGAGGTGGTGATTTGT

AATAATAATGAGGTGGTGGTAGAGGAGATATATAGCCATAAGGAGGCGAATAGTCGTAGG

GAGGGGAAGAATAGGGTGGTTGAGTAG

>TRINITY_DN10306_c0_g1|m.505 TRINITY_DN10306_c0_g1|g.505 ORF TRINITY_DN10306_c0_g1|g.505 TRINITY_DN10306_c0_g1|m.505 type:5prime_partial len:310 (-) TRINITY_DN10306_c0_g1:346-1275(-)

ATTACAAATCACCTCCTCCGCCTACTTATTATTACAAATCACCTCCTCCGCCTACTTATT

ATTACAAATCACCTCCTCCGCCTGTTTATTATTACAAATCACCTCCTCCGCCTACTTATG

ATTACAAATCGCCACCACCACCCTACAAGTCTCCACCATACTATTACAAGTCCCCATCTA

CTCAACCACCCTATTCTTCCCCTCCCTACGACTATTCGCCTCCTTATGGCTATATATCTC

CTCTACCACCACCTCATTATTATTACAAATCACCACCTCCTCCTTATGTTTACAAGTCAC

CACCACCACCTCCCTATAAGTACAAATCACCTCCCCCTCCTTACTACTATGCAAATCCTC

CTTATGCCTACAAGTATCCTTCACCACCTCCTTATTATTATAAATCACCGCCTCCTCCCT

ATGTCTACAAGTCACCACCACCACCTTATGTCTACAAGTCACCACCACCACCTGCCTACA

GTTACAAATCACCTCCTCCTCCATATTACTATTCAAATCCTCCTTATGCTTACAAGTATC

CCCCACCTCCTTACTATTATAAATCACCAACCCCTCCTGATGACTACAAATATCCTCCTT

ATGACTACAAATATCCTCCATCATCACCTCCTTACAAATAAATCACCATGTTCTCCTTAT

GTTTATAACTTATCACCGCCATTATCTCTGTCACCACCTCCACATGTTTGTTCCAGTCCT

CGTGTCTACAAGTATCCGTTATCGCCCCCTTCATTACCTCCCTACACACCATCTCTAAAC

ATCATCAACTCTCCCCCCTACCATTATCAAATCCTCCAGCTAAGTATCATCTACCACTAT

CTTCCTTACCTTCATCACTACTATAAATCACCTTTGCTCTCTTATGTTTACAAGTATCCT

CTACCACCACCTCCATCACCTTCTCATTAG

>TRINITY_DN10306_c0_g1|m.506 TRINITY_DN10306_c0_g1|g.506 ORF TRINITY_DN10306_c0_g1|g.506 TRINITY_DN10306_c0_g1|m.506 type:5prime_partial len:213 (-) TRINITY_DN10306_c0_g1:635-1273(-)

TACAAATCACCTCCTCCGCCTACTTATTATTACAAATCACCTCCTCCGCCTACTTATTAT

TACAAATCACCTCCTCCGCCTGTTTATTATTACAAATCACCTCCTCCGCCTACTTATGAT

TACAAATCGCCACCACCACCCTACAAGTCTCCACCATACTATTACAAGTCCCCATCTACT

CAACCACCCTATTCTTCCCCTCCCTACGACTATTCGCCTCCTTATGGCTATATATCTCCT

CTACCACCACCTCATTATTATTACAAATCACCACCTCCTCCTTATGTTTACAAGTCACCA

CCACCACCTCCCTATAAGTACAAATCACCTCCCCCTCCTTACTACTATGCAAATCCTCCT

TATGCCTACAAGTATCCTTCACCACCTCCTTATTATTATAAATCACCGCCTCCTCCCTAT

GTCTACAAGTCACCACCACCACCTTATGTCTACAAGTCACCACCACCACCTGCCTACAGT

TACAAATCACCTCCTCCTCCATATTACTATTCAAATCCTCCTTATGCTTACAAGTATCCC

CCACCTCCTTACTATTATAAATCACCAACCCCTCCTGATGACTACAAATATCCTCCTTAT

GACTACAAATATCCTCCATCATCACCTCCTTACAAATAA

>TRINITY_DN1030_c0_g1|m.511 TRINITY_DN1030_c0_g1|g.511 ORF TRINITY_DN1030_c0_g1|g.511 TRINITY_DN1030_c0_g1|m.511 type:3prime_partial len:103 (-) TRINITY_DN1030_c0_g1:2-307(-)

ATGGAGATGGAGGAGGTGGTGACTTATAGACATAGGGAGGTGGTGGAGAAGGTGATGGTG

GTGGAGGAGACTTGTAGACATAAGGAGGGGGCAGGAATGGAGGAGGAGGCGGTGACTTAT

ACATGTATGGGGGAGGTGGTGATGGAGAAGGAGGTGGAGGTGATTTGTAAACATAGGGAG

GTGGTGGTGACGGCGATGGAGGAGGTGGTGACTTATACACGTAGGGAGGTGGTGGAGATG

GTGATGGTGGTGGAGGAGACTTGTAAACATAAGGAAGGGGTGGGGACGGAGAAGGAGGAG

GTGGTG

>TRINITY_DN1030_c0_g1|m.510 TRINITY_DN1030_c0_g1|g.510 ORF TRINITY_DN1030_c0_g1|g.510 TRINITY_DN1030_c0_g1|m.510 type:complete len:119 (-) TRINITY_DN1030_c0_g1:391-747(-)

ATGTATGAAGCTCCAGAAAAGATTAAATTCGCATCAGAAAAAACTCCGATAACTGCCATG

AATCAGTCTAGTAGTAGGCGGGTGGAGGTGGAGACTTGTAGTAGTATGGAGGAGGGGGTG

AAGGAAATGGTGGTGGAGGGGACGTATAGTAGTAAGGAGGAGGTTGGGATGGAGATGGAG

GTGGAGGCGACTTGTAATGGTATGGAGGAGGTGGTGATGGTGATGGGGGAGGCGGCGACA

TGTAGATGTAGGGCGGAGGTGGCGAAGGGGAGGGTGGTGGAGGAGATTTGTAAACATAGG

GCGGGAGAGGTAAGGGGGAAGGAGGAGGTGGTGACTTGTAAATATATGGGGGGGTAG

>TRINITY_DN10312_c0_g1|m.513 TRINITY_DN10312_c0_g1|g.513 ORF TRINITY_DN10312_c0_g1|g.513 TRINITY_DN10312_c0_g1|m.513 type:3prime_partial len:126 (+) TRINITY_DN10312_c0_g1:102-476(+)

ATGGCACCTGTGATCGCCAACAACAAGGCCTTGGTCGCTGCCGCCGGGTTCGCTAAACCT

GTTGCGGGCTTGCCAAGCCGGGCCTTCGTCACTTTCCTTGCCGGCGACGGCGACTACGTC

AAGGGAGCTGTGGGCCTTGCTAAGGGCCTGCGAAAGACAGGCACGCCGTACCCGCTCGTC

GTGGCGGTGCTCCCGGACGTGCCGGTAGCTCACCGGAAGCTGCTCGAGTCGCAGGGTTGC

ATCGTCCGCGAGATCGTGCCCGTGCACCCGCCGGAGAACCAGACCCAGTTCGCCATGGCG

TATTACGTCATCAATTATTCCAAGCTCCGCATTTGGGAGTTTGTGGAGTATAGCAAGATG

ATATACTTGGACGCC

>TRINITY_DN10313_c0_g1|m.514 TRINITY_DN10313_c0_g1|g.514 ORF TRINITY_DN10313_c0_g1|g.514 TRINITY_DN10313_c0_g1|m.514 type:internal len:130 (-) TRINITY_DN10313_c0_g1:2-388(-)

CCCGTTTCCCAACTCCAAACCCTAGAAAAGAAAACATCCCCCAATAATCAAACCTCTCTC

TTAATTTATAAAAAAAAATTCGATCTCAATCAATCAATGACGAGCTACGGCACGATCCCG

ACATCGATGCCGGCGGCAGCGGCCTCCGACTCGGCCTCCCGGCTCGACTTCCTCTCCCGC

GCGAAGGAGCGCGGCCGATCCGTCCTCGCCTTGCGGCGGCCGTGGCGGGAGGTGGTCCAC

CGGCACGCCTTCTCCCTCCCGCCGTCCCTAGGCGACGCCTACCTCCGGATCCGCACCAGC

CTCTCCTACTTCGCCATGAACTACGCCATCGTCGCCCTCCTCGTCGTCTTCCTCAGCCTC

CTCTGGCACCCAGTCTCCCTCATCGTC

>TRINITY_DN103181_c0_g1|m.515 TRINITY_DN103181_c0_g1|g.515 ORF TRINITY_DN103181_c0_g1|g.515 TRINITY_DN103181_c0_g1|m.515 type:complete len:101 (-) TRINITY_DN103181_c0_g1:374-676(-)

ATGCCTAAGGCTGCTGCTACCAAGCGTGGAGGAGTTAAGAAGCGCGCGAAGAAGGACCCC

AACGCCCCCAAGCGTGGCCTTTCTGCCTACATGTTCTTCGCCAATGAGCAGCGCGAGAAC

GTGCGTGAGGAGAACCCCGGCATCTCCTTCGGCCAGGTCGGCAAGCTCCTTGGTGAGCGA

TGGAAGGCCCTGAACGAGAAGCAGCGCACGCCCTACGAGGCCAAGGCTGCCGCCGACAAG

AAGCGTTATGAGGATGAGAAGCAAGCCTACAACGCCGACCAGGAGGAGGATGAGTCCTCT

TAG

>TRINITY_DN103181_c0_g2|m.516 TRINITY_DN103181_c0_g2|g.516 ORF TRINITY_DN103181_c0_g2|g.516 TRINITY_DN103181_c0_g2|m.516 type:5prime_partial len:191 (-) TRINITY_DN103181_c0_g2:413-985(-)

CCCAGCATAACAGTCCGTCCAAGTGTGGTAGCTGGGCAGGCCGCGTTCCCTCCCCACGCA

CAATCAGCTCCCCATAGTAAAAAGGCTGCCTTGGCCTCCCCATACATTTTCCCTCCCTCT

CTCCTCTCCCATACAATCTTTCACCCAAAATCCTCCCTCATACCCGCGTCTCTCTATACC

TACTCGTCCACGCTCGACACGTCTTTGTGTTCACGACATCACCACCACTTTCTCGTTCCC

AAATTCATTTCCAAGAACATATCCAAGATGCCTAAGGCTGCTGCTGGCAAGCGTGGAAAG

GTTGAGAAGAAGCGAGGCAAGAAGGACCCCAATGCCCCCAAGCGTGGTCTCTCTGCCTAC

ATGTTCTTCGCCAACGAGCAGCGTGAGAACGTTCGTGAGGAGAACCCTGGCATCTCTTTC

GGCCAGGTCGGCAAGCTCCTCGGTGAGCGATGGAAGGCCCTGAACGAGAAGCAGCGTGCC

CCCTATGAGGCCAAGGCTGCTGCCGACAAGAAGCGATACGAAGATGAGAAGCAAGCTTAC

AACGCCGACCAGGAAGAGGAGGAGTCCTCCTAG

>TRINITY_DN10319_c0_g1|m.517 TRINITY_DN10319_c0_g1|g.517 ORF TRINITY_DN10319_c0_g1|g.517 TRINITY_DN10319_c0_g1|m.517 type:complete len:281 (-) TRINITY_DN10319_c0_g1:341-1183(-)

ATGGCGGAACATCTTGCTTCGATATTTGGCACAGAAAAGGACAGGGTCAACTGCCCCTTT

TACTTCAAGATTGGTGCTTGCCGCCACGGTGATCGATGCTCCCGCCTGCACAATCGCCCT

ACCATCTCCCCCACACTCCTACTGTCCAACATGTATCAGCGTCCAGACATGATCACTCCT

GGTGTTGATGCTCAAGGCCAGCCCATTGATCCACGGAAGATACAGGAACATTTTGAGGAC

TTCTATGAAGACATCTTTGAGGAACTCAGTAAGTTTGGTGAAATTGAGAGCCTGAACATC

TGCGATAACCTAGCAGATCACATGGTGGGGAATGTTTATGTTCAGTTTAAAGAGGAGGAT

CAAGCGGCTGCAGCTCTTCGGGCCCTTCAAGGTCGGTTTTACTCTGGACGTCCAATAATT

GTAGATTTTTCCCCAGTTACCGACTTCAGGGAAGCCACATGTCGTCAGTTTGATGAGAAT

AGTTGCAACCGAGGTGGCTATTGTAATTTCATGCATGTGAAGCAAATCGGGAGGGAGCTG

AGAAGGAAGCTATATGGGCACTACATGACGTCTCGAGGGAGCCGAAGCCGGAGCAGGAGT

CCTAGTCCATACTATAGAAGGAGCCACAGTGATTACGGGGACTATCGCGATCGTGGGGAT

CATCGGGACAGTGGCCGGAGGAGTAGTGATCGGAGAAGCAGTGATCGCCATGGGAAGTAT

GATAGTGATGGGGGAAGGCGGCGACATGGGAGTCTGAGGACTAGGAGCCCAATTAGGGAA

GGGAGCGAGGAAAGGAGGGCTAGAATTGAGCAGTGGAACAGAGAACGAGAGGAAAGGATT

TGA

>TRINITY_DN1031_c0_g1|m.518 TRINITY_DN1031_c0_g1|g.518 ORF TRINITY_DN1031_c0_g1|g.518 TRINITY_DN1031_c0_g1|m.518 type:complete len:108 (-) TRINITY_DN1031_c0_g1:864-1187(-)

ATGCCCAGGATGAAGGTGAAAAGTCTCCACACTGTTCGTGCTGAGACAGAGACGCCTGTT

AGTGCTGTTTCCATCAGAAGAATTCCAAGGGTTCCAGCAGATATGCCTCTGTATGATATA

CTGAACGAATTTCAAAAAGGAAGCAGCTATATGGCTGCTGTTGTAAAGGTTAAAGGAAAA

GGAAAGAATCCCCCACCTGCTGATGGTGGAAAATCTAAAGAAAAAAAGAATCTAGTGGAA

ACACCACTGCGACTCTTCTTTCTAAAGCTGACAAAAAATCAGACAATGTTGTTGTTGACA

TTGACAAACGACAAATTCAGGTAA

>TRINITY_DN1031_c1_g1|m.519 TRINITY_DN1031_c1_g1|g.519 ORF TRINITY_DN1031_c1_g1|g.519 TRINITY_DN1031_c1_g1|m.519 type:internal len:306 (+) TRINITY_DN1031_c1_g1:2-916(+)

AAAAAAAAAAAAAAAAAAAAAAAAAAAAAGAAGGAAAAAAAGATGCAGGCTTTGAGCGCC

GTTGCCGTGGCTCGGATGCTGATGATGAAGGGCAACGGAATGATCGTGGTGGAGGGCGAG

GACATCTCGCTGGGATCTCTGTCTTGGTACATCTACGCTGGGATCTCCTGCTTCCTCGTC

CTCTTCGCCGGTATAATGTCGGGATTGACCCTCGGATTGATGTCGCTGGGCCTCGTCGAG

CTCGAGATCCTCCAGCGCAGCGGAACGCCCACCGAGAAGAAGCAGGCCGCTCACATCCTT

CCTGTCGTTCAAAAGCAACATCAGCTTCTGGTGACATTATTATTATGTAACGCTGCTGCC

ATGGAGGCTCTTCCTATATTCCTTGACAAGATATTTCATCCTATTGTTGCTGTTATCTTA

TCCGTAACATTTGTTCTAGCTTTTGGAGAGGTAATTCCACAAGCCATATGTACAAGATAT

GGCTTGGCTGTGGGTTCTAATTTTGTTTGGCTTGTTCGTATTTTGATGGTCGTTTGCTAC

CCCATATCTTATCCTATTGGGAAGCTTTTAGATTTTGCACTTGGGCATAATGAATCTGCA

CTTTTTAGACGTGCTCAGCTAAAAGCTTTGGTCTCTATTCATGGCAAAGAGGCTGGTAAG

GGTGGGGAACTCACTCATGACGAGACAACAATTATAAGTGGCGCGCTAGATTTGACTGAA

AAGACTGCTGAGGAGGCCATGACACCTATTGAGTCAACGTTCTCACTAGACGTGAATTCG

AAACTGGACTGGGAAGCAATTGGCAAAATTCTTGCTCGGGGGCACAGCCGAGTCCCTGTT

TATTCAGGAAATCCTAAAAACATTATTGGCCTTCTGCTGGTGAAAAGTCTCCTCACTGTT

CGTGCTGAGACAGAG

>TRINITY_DN1031_c1_g1|m.520 TRINITY_DN1031_c1_g1|g.520 ORF TRINITY_DN1031_c1_g1|g.520 TRINITY_DN1031_c1_g1|m.520 type:5prime_partial len:117 (+) TRINITY_DN1031_c1_g1:1-351(+)

AAAAAAAAAAAAAAAAAAAAAAAAAAAAAAGAAGGAAAAAAAGATGCAGGCTTTGAGCGC

CGTTGCCGTGGCTCGGATGCTGATGATGAAGGGCAACGGAATGATCGTGGTGGAGGGCGA

GGACATCTCGCTGGGATCTCTGTCTTGGTACATCTACGCTGGGATCTCCTGCTTCCTCGT

CCTCTTCGCCGGTATAATGTCGGGATTGACCCTCGGATTGATGTCGCTGGGCCTCGTCGA

GCTCGAGATCCTCCAGCGCAGCGGAACGCCCACCGAGAAGAAGCAGGCCGCTCACATCCT

TCCTGTCGTTCAAAAGCAACATCAGCTTCTGGTGACATTATTATTATGTAA

>TRINITY_DN1031_c2_g1|m.523 TRINITY_DN1031_c2_g1|g.523 ORF TRINITY_DN1031_c2_g1|g.523 TRINITY_DN1031_c2_g1|m.523 type:5prime_partial len:670 (-) TRINITY_DN1031_c2_g1:272-2281(-)

GCAGAGTTCCTTTCAAAGGCTACAGGGACTGCTGTCGATTGGGTCCAGATGCCCGGGATG

AAGCCTGGTCCGGATTCGATTGGGATTGTTGCCATTTCACAAACTTGCAGAGGAGTGGCG

GCACGAGCCTGTGGTCTAGTGAGTTTAGAGCCTACAAAGATCGCAGAAATTCTCAAAGAT

CGTCCATCCTGGTTCCGTGATTGTCGGAGCCTCGAAATCTTCACCATGTTTCCAGCTGGA

AATGGAGGTACAATAGAAATGGTACATATGCAGATGTATGCTCCAACGACATTAGCGCCT

GCGCGCGACCTTTGGACTCTGAGGTATACAACGAATTTAGATGATGGCAGTCTTGTGGTT

TGTGAGAGATCTTTGAGCGGTTCTGGGGCTGGTCCTGGTTCAACCAGTGCTCATCAGTTT

GTAAGAGCTGAGATGCTTCCCAGTGGGTTTTTAATTCGGCCATGTGATGGCGGTGGTTCA

ATCGTCCACATTGTTGACCATTTGAATCTTGAGGCGTGGAGTGTGCCTGAAGTGTTGCGA

CCCCTTTACGAGTCATCCAGGGTAGTGGCTCAGAAGATGACCACTGCGGCACTCCGCCAC

ATAAGGCAAATAGCTCAAGAGATGAGTGGTGAAGTGGTGTATGCTTTAGGAAGGCAGCCT

GCTGTTCTACGTACCTTTAGTCAAAGATTGAGCAGAGGTTTTAATGATGCTATCAGTGGC

TTCGGTGATGATGGTTGGTCAATTATGGGATGTGATGGTGAAGATGTACTAATTGCTACC

AATTCATTGAAGAAAACTAATTCTCATGCTAATCCTGTGAATGCTGTCTCCCTGCCTGAT

GGGGTGATTTGTGCCAAGGCATCAATGCTACTTCAAAATGTGTCTCCTGCATTGCTGGTT

AAGTTTTTGAGGGAACACCGTTCGGAATGGGCTGATTACAACATGGATGCATACTCTGCT

TCCTCTTTGAAGGCGGCCTCAGCTTCTTTTCCTGGACTGAGGCCTACTAGGTTTTCTGGG

AGCCAAACCATCATGCATCTTGCTCACACGGTCGAGAATGAGGAGTTTCTTGAGGTGGTT

CGTCTTGACGGCCAGGCTCTTACTCAAGATGAAGCTGTTTTATCAAGGGATATTCATCTA

TTACAGCTTTGTAGTGGAATGGATGAAAATGCTGTTGGAGCGTGCTACCAGCTTGTTTTT

GCTCCAATTGATGAGCTTTTCCCGGACGATGCTCCTCTGCTGCCTTCCGGTTTCCGTGTT

ATTCCGCTGGATGCTAAATCAGATGGTTTGAATTCCAATCGCACACTAGATTTGACATCT

AGTCTTGAAGTAGGCTCTGCTGCAAACCGAGCTGGTGGAGAAACATCTCCGATTGATTAT

AGTTTGCGGTCAGTGTTGACAATTGCCTTTCAGTTCCCTTATGAACTCCATCTCCAGGAG

AGTGTTGCTGCAATGGCCAGACAGTATGTTCGCAACATTGTCTCCGCCGTGCAGAGGGTT

GCCATGGCCATAACTCAATCCCGACTTAGACTTGGTTCGGGAGATAAACTACTCCCTGGA

TCCCCTGAAGCCGTTACCCTTGCTCGCTGGATTTGCGATAGCTACAATTATCATTTGGGT

TTGGATTTGCTGAGATCCACCGAAGAATCAGGCGAATCCTTGTTAAAAATGGTGTGGGAT

CATCAGGATGCCATCTTATGTTGTTCCTTGAAGACGCAACCAGTCTTCACCTTTGCCAAC

CAAGCAGGGCTTGATATGTTGGAGACAACACTGATTGCCTTACAAGACATTACTCTGGAT

AAGATCTTCGATGAGCCTGGACGAAGGGCGCTGTGCCCCGACTTTGCCAAGTTGATGGAA

CAGGGTTATTCCTATTTGCCAGGGGGTGTTTGTTTGTCAGGAATGGGGCGTCATGTTTCG

TATGAGCAGACAGTAGCCTGGAAAGTCCTGGGGGAGGACAACAGCGTCCACTGCCTTGCC

TTCTGTTTCATGAACTGGTCGTTTGTTTAA

>TRINITY_DN10324_c0_g1|m.525 TRINITY_DN10324_c0_g1|g.525 ORF TRINITY_DN10324_c0_g1|g.525 TRINITY_DN10324_c0_g1|m.525 type:5prime_partial len:425 (-) TRINITY_DN10324_c0_g1:160-1434(-)

GTAACCCACCACGTACTGACTATGAAGTTCAGGTTCGCTGCGCTGCTCGGCGTCGTCTCT

GCTGGCGTCGCACTCGCCTCCATAACTCCCCAGCAAGTCCCCCTTCAAGCCCTCGAGGAT

GAGCTCACCGAGGCCTTCGTCGAGCAGTGGAAGAACATGGATGTCGTTGACGCGATGAAG

ATGGCTGGGTTTGGTGCTGGTCTCGATGAGCAGAGAGTTATCCAGATATTTGGGGAGGAT

ACCCCTAAGCGTGTCACGGAGGGCGACAAGCTTCGTCTTAAGAAGGCTGGCTATAGTTTC

ATGGATATCACAGACCACCCCAACCTTGGTCAGCGAAATGCAGCTAGGCTGCAATCTTCC

GCAGCCACTTTCGACATCTCGCAGCCCCTCAAGAATGCGAAACAGACCCAAGCTATGCTT

CGGGGGACATTTGATGACGCAATCAATTTCCTCAAAGCGGACATCGAGAAGCTCTCATCG

TTCTGGAATAGAAACTATAAGAGCGAGTGGGGGCACAAGAGCAGCAATTGGGTGTACGAA

CACGTCAAGGAGATCCTGGCAGAGTCCAAGGCCCATGCGACGATCAGGAAAGTACACCAT

GCCTTCCCCCAGAACTCTGTGATTGCCCGCTTGGAGGCACCCGATTCGACCGTCGAAGAC

AAGCAGGTCGTCATCCTCGGTGCGCATCAAGACTCCCTCAACTATCGCCTGCCCTTCTAC

CGCGCCCCTGGTGCAGACGATGATGGGTCTGGTTCGGTTACTATCATGCATGTGCTGCGC

TCACTCGTTAGGAGCGGTTTTGTACCCCCAGAGGACGTTGCAGTTGAGTTCCACTGGTAC

GCCGCCGAGGAGGGTGGACTGCTTGGTAGCCAGGACATTGCGGCGCAGTACGAGAAAGAT

GAGGTCAAGGTGAAGAGTATGCTCCAAATGGACATGACTGCGTTCGTCAAGGAGGGTACT

ACACCCATCATCGGCTTCTTCAAAGAATCCGTTGACAGGAATCTCACCACATACTGCACT

AAGCTTGTTGATGCGTACATTCCTCTGCCATGGAATCTTACTGGCTGCGGGCCTGTCTGC

GGTTCGGATCATATGAGTTGGACCAAAGCCGGTTACCCTGCTGCGTTCGCCACCGAGGGT

CTCTTTGAATATTTCCCTAGGGGCATCCACACGGAGTTTGACGATACGGACCAAGATGGC

CAGTACAGCTTTGAACATATGTTCCAGTTCGTCAAGCTCGGTATCGCATACATCACTGAG

ATTGTTGTTGTTTAG

>TRINITY_DN10325_c0_g1|m.529 TRINITY_DN10325_c0_g1|g.529 ORF TRINITY_DN10325_c0_g1|g.529 TRINITY_DN10325_c0_g1|m.529 type:complete len:442 (-) TRINITY_DN10325_c0_g1:372-1697(-)

ATGTTATTTAAATTTAATTTTACTTTTGATACCTTGTTCAGTTGCGGTTCGGTGATGAGT

TTCTCCTTCGTCCCCGAGTTCGATCTTGAAGACGACGATCCCGTCACCTCCGCTGCTCCG

GTTGACCATCACAACCGCAAGATCATTTCTCAGTCACACGAAGATGATGAGTCTCAGCTG

CTGGAGCTGCTATGGCAGCAGGGTCCTGTCGTCTGCCCGCTCGAGTCCGCCGCTACGCTG

CCTCCCGAGCGCAGACACGTGGGATCCACGGCGGAGGCCAGCCACCAGCTGCAGCAGCTG

TTCATGGAGGAGGACGAGATGGCCACGTGGCTACATCACTATCAGGAAGGCGGTGGCGAG

GGAGGAGCAGGAGGAGGAGGAAGTGATCTGTACAGCGAGATGATGGGGCCGTCGTCGAGA

GCGAGCTGCTGCAGTCAGGTTGCCGGGGTTCAGCAGGTGAAGCATCAGGAAGCTGCTGGT

GCTGCTGCGAGGGCGGCGGCGGCGGCGAGACAGGGGAGGTCGACCGTGGTGGCGGTGGAC

GCAAGCAAGGTGTTCGATGCTGGGTCTGTGAGAGCTTCCGTCGCCGAATGCAGCATGCAT

GTGAGCGACAGGAAGCGCAAGGGCGGCCCGAGGGCGGAAAAGGACAGCGAGAATGTCGAG

GTTGAATATGGTAACAAGAGAAAGGATGCCCGCCAGTCTGCACCCCCAAGAACTCGTACT

GCTGAGGTTCACAATCTATCTGAACGGAGACGTCGAGATAGGATCAATGAGAAGATGAAG

GCCTTGCAAGAACTCATACCCAGATGCAATAAGTCAGACAAAGCTTCGATGTTGGACAAT

GCAATAGAATACATGAAGTCACTTCAATTGCAAGTGCAGATGATGTCACCTGTGCAATAC

AACATGGGCCAGATGATGTTCCCAGATGCTCAAAGGTACCTGCAGACTATGAGATTCATG

AGCATGGGTATGGGAATGCCTTCAGGGGGCATGCAACGTCTGGAGATACCGTTTGGCTCA

ATCTTGCCTTACCCCCCTGTTGTTGGACCATCACCAGAAAATCTACAACCACGGCTAGTG

CCATCATTCCGTCCGGCAAATGCTGCTATCCCTGATCAAGTGAGAATGGAAGCATCCAAC

CAGCAAGACCCACTATCAAGTTCTATTGGCGTGATGGGCCCCAACATGGTGCAAATCCCA

AATCTTGCTGATCCTTATCACCATTATGCTCCCCTTCACCATCTTCAAGTACCATTTCAG

GGTCAAACATTGGGTGAACCTGGAACTAGCAAGGAAACTGATAATACTAGAAGGTCTACA

GGTTGA

>TRINITY_DN10326_c0_g1|m.531 TRINITY_DN10326_c0_g1|g.531 ORF TRINITY_DN10326_c0_g1|g.531 TRINITY_DN10326_c0_g1|m.531 type:complete len:137 (-) TRINITY_DN10326_c0_g1:402-812(-)

ATGCACCTTATCAATATACAGGTCAAAAGGTTAGTGAAGTGGAATCTAAAATACCTTTCT

GTATCATCTCCGCGGGTCCTCCCTATCGTTTTTGTATCCAACTTCAAACTTGGACAGAGC

TGCGAAACAAATGCATTCTTTTGTCAGGAATTGGCATCCGTGCTGGTTTGTGAGGATAGC

AACAAGGGGATGTCTCTAATCCGTGATCCATTGCCCCAAAAGGAAGCAAGGCTTAGACCC

ATGATTGCCATTGCTACGACCGAGCATGCAGCGGGAACTGCATGGCTACTTCCAAGGAAT

TGTGTCGCCAGTAGCCCAGCCAAGGTGGAGCTCTGCATTCCTGTGCATAATGAAATTGTC

CTGCAAACCGGCTCTTCTTGCCTCAAAGATGGCAATTTGGATAACCAATAA

>TRINITY_DN10326_c0_g1|m.530 TRINITY_DN10326_c0_g1|g.530 ORF TRINITY_DN10326_c0_g1|g.530 TRINITY_DN10326_c0_g1|m.530 type:5prime_partial len:223 (+) TRINITY_DN10326_c0_g1:1-669(+)

GTTGCGGGGGCACAACTCTCAAGTTATGCCAGTTATTTGAGTAAAGGAGACGTTGCATTG

AGTATCTTATTAACTAGCTCAACAACAATTTCTTCTGTGCTCGTAACCCCTCTTCTTACT

GGCCTTCTAATTGGTTCTGTTGTCCCAGTTGATGCAGTTGCAATGTCAAAGTCAATTTTG

CAGGTTGTGCTTTTTCCAGTAACCGTGGGCCTTGTTCTCAATACTTATGCCAAAGGTGTG

GTAAATTTCATTCAACCAATTATGCCCTTTGTTGCTATGATCTGCACTTCATTATGCATC

GGAAGCCCTCTTGCTATCAATCGGAGGCAGATCCTATCATCAGATGGTCTGTATTTACTT

CTTCCCATACTGACATTCCATCTTCTAGCCTTCACAATTGGTTATTGGTTATCCAAATTG

CCATCTTTGAGGCAAGAAGAGCCGGTTTGCAGGACAATTTCATTATGCACAGGAATGCAG

AGCTCCACCTTGGCTGGGCTACTGGCGACACAATTCCTTGGAAGTAGCCATGCAGTTCCC

GCTGCATGCTCGGTCGTAGCAATGGCAATCATGGGTCTAAGCCTTGCTTCCTTTTGGGGC

AATGGATCACGGATTAGAGACATCCCCTTGTTGCTATCCTCACAAACCAGCACGGATGCC

AATTCCTGA

>TRINITY_DN1032_c0_g1|m.533 TRINITY_DN1032_c0_g1|g.533 ORF TRINITY_DN1032_c0_g1|g.533 TRINITY_DN1032_c0_g1|m.533 type:complete len:818 (+) TRINITY_DN1032_c0_g1:261-2714(+)

ATGGATAATGAAGTTGCACCAGAGTTTATCTATGATAATGGCCTATATTATCCTGCACCT

GGCGTTGGCAACTATTATGGATATGTCTGCACAGGGATGGAACCACCTTGTGAATGGGAT

GACCACAATAAGTTTTTGAGCACAGATAGTCAAGATCTTCAGTTCCCTGGGTTGCAGACT

GAAAATTTGCCGTGTGTATATTACACACCTAGCTATGACTATGCACAGTCTCCTTACAAC

CCTTTCAATCCGTACATTCCTGGTGCTGTCATGGGAACCGATGGTCCATACATTGGAACA

CAACAGTGCTTCAACAGCTCTCCGTACCTTCAAACCGTTTCTCCACCTGGTTATGTCCCT

GTTATCAGTCAACCTGGTTCAGATCCTCTCCTGAATGGTTCACCTGATGCATTGATTTTC

AACAGTGGTGCTGTAAAATATGCACCCTTGTTATCATCTATGAGTGTAACCACCGCACCT

TTGAAAGTTTCTTTTGAGAACTTATCGCTAGAGCCTTCCCACTCGTCTTCCCATCAGATT

CAGTTACCTGGCAGATTGTCAGAGGGGGCAGGGGTTAATGAAGCACCAAGAAACAAGTTC

TCACCATATGGAGACATGACAAGTGGTGTTGCTCCTCAAATAGCCCAGGGAACAGAACAT

TTGCCGTTTGTGGGAGTTTCTTCTATCCGCAGTCCATTGAAAGTACCATTTCCAGCCAAC

AATGGCTTTACAAATCCTGTATCCAACATCCGTGGATGGGCTCCAGTCGATAGAATGAGG

CCACGAATCCAATATGGTGGGTTGCCAAACAGAAGCCTGGGTGAGCAAAATATGGGTCCT

AGAAACAACAAGCCTAAAACTCAGTTGACATCACCAATTACTCTAAAAACCTACACAACC

AAGGTCGGCACTGGTAATTTGGATGGAAACATAGTTGTTCATGCTGATCAATATAACAGG

GATGATTTTCCTGTTGATTATCCGGATGCCAAGTTCTTCGTCATCAAATCCTACAGTGAG

GATGACGTTCATAAGAGCATCAAATACAATGTATGGTCAAGCACTTCCAGTGGAAACAAG

AAACTGGACATTGCCTATGACGAGGCACAGAGAATCTCTGTTGGGAAACAAAGAAAATGT

CCAGTGTTTCTTTTCTTCTCTGTTAATGCTAGTGGTCATTTCTGTGGTGTTGCTGAGATG

ATTGGCCCTGTTGACTTCCACAAGGACATGGACTTCTGGCTGCAAGACAAATGGACTGGT

AGCTTCCCCGTCAAGTGGCATATAATAAAGGATGTTCCCAATTCAAGCTTTTGGCACATT

ATATTGGTGAACAATGAATACAGACCTGTGACTAGTAGCAGAGATACCCAAGAGGTTCCA

TACCTACCAGGCGTTAGTATGTTGACTATTTTTAAGAACTGCCCGGTGAATACATGGATA

CTTGATGACTTCATGTACTATGAAGAGCGTCAGCGGATGATGCAGGAAGAGAAATCCTTG

CTTCTGGGAAGAAAAACCCTTGAAACCTCTGTTTCTCCAGCGACATCTGTTCCCTCCATC

AAACCTATTGGTGCAATCAATCATCTTCCGAATGGGGATGAACAGCAACTGATTCGTGGT

GTCGTCCAGCCTCCTATTGCTAATGGAAAAGAAGCAAATGTTAAAGCCAGCCATTGTCTT

GAATCCGATGGAAGGAAGGCTATTCTCAACACTGCTGATCAAGGTCTGAAGGCAGACAAT

AAGCAGAAGGATGCTGAAGTCAGCCGTCTACTAAGTCGTGAAGGACATTTGAATGGTACG

GTGAACCAACCTTCAAAATCTGATGCTCATTTGTCAGGTGATGCCAATCCTGCGGCGAAA

GCAGATGCAAAACAAATAAAGCATAGATCAAAACAGTCTCTGGAAACTAAAAATGAAAGG

AAAGCTATTCCTAAGACTGCTGATCAAGATCCGAAGGCAGACAATAAGCAGAAGGATGCT

GAAGTCAGCTGTCTACCAAGTTGTGAAGGACAACTGAATGGTACTGTGAACCAACCTTCA

AAATCTGATACTCATTTGTCAGGTGATGCCAGTCCTGCGGCAGAACCAGAAGGAAAACAA

ATAAAGCATAGATCAAAACAGTCTCTGGAAACTAAAAATGATCTAGGCAAGAATCAAAGT

ATCCGTATTGCCTCAAGCAAATCAAGCCCTGACGATGTGCATGCTACTTTGAGACATTCT

GTCACAGATTCGAAAGATAAGAGAAATGTCAGGTCTGAGTCCAAGGTGGGTTCCAACAGA

CAGAATTCTTCTTCTGGAGCGATTGATTCCATCTCTGCTAGTGATAAGATCGACGAACCT

GATGTTCTCACTGTGGGCTCGATGCAATTTGAAATCACCAACAAGAACAGCTCTATTTCT

GGGACCCTAACCATAGGCTCAATCCCCATCGACACCAATGGATTGAAGTCTTAA

>TRINITY_DN1032_c0_g2|m.534 TRINITY_DN1032_c0_g2|g.534 ORF TRINITY_DN1032_c0_g2|g.534 TRINITY_DN1032_c0_g2|m.534 type:5prime_partial len:111 (+) TRINITY_DN1032_c0_g2:1-333(+)

CTGGAAACTAAAACTGATCAAGGAAAGAATCAAAGTATCCCTATTGCGACAAGCAATTCA

AGCCCTGATGATGTGCATGCTACTTCGAGACATTCTGCCACAGATTCGAAAGATAAAAGA

AATGTCAGGCCCGAGTCTAAGGGAGGTTCCAATAGACAGAATTCTTCTTCTGGGGCGGTC

GGTTCTCTCTCTGCCAGCAGCAATAAGATGGATGAACCTGATGTTCTCACTGTGGGTTCG

ATGCAAATCGAAATTACTAACACCAACAGCTCTATTTCTGGGACCCTAACCATAGGCTCA

ATCCCCATTGATACCAATGGGTTGAAGTCTTGA

>TRINITY_DN103301_c0_g1|m.535 TRINITY_DN103301_c0_g1|g.535 ORF TRINITY_DN103301_c0_g1|g.535 TRINITY_DN103301_c0_g1|m.535 type:complete len:277 (-) TRINITY_DN103301_c0_g1:476-1306(-)

ATGCTCAAGGTTAGCATAGCCGTCCTCCTCTTTGTTGGCGTTGCCACCGCCAACCCCCTG

CAAGGTTCCATCAAGGGTTATGGCCTTGTGTCTCCCATCTCCAAGCACCGATCTGCCGTG

GCCGGTCCAGTCCGACAGGCTCCCCCATCTTATGTCTCCCCACCAGTGTCCACCAAGGGA

GGTTCCAAGTTGGTTCTGCCCAACTACAAGCCCCGACCAGCCACCAAGGTCGGTCCAGTC

ACCGCCGCCGTGATCACCTCCCGAGCCCAGGAAGTGATCAACGTGCCAATCAGCGAGGAC

ATCGTCACTCCCCAGGTGGTGATCATCGAGCCCAATCTGACCCCAGTCACCATTGAGTTT

CGCTCTCGCTCGAGCCCAGTCAATATTCAACAAGTTCACATTCCCGGTCCACGCGGTCAA

ACTAAGACTACCCGCTCTCAAGAGCAGCCTGACCGAGTTATTCACGAAGTTGTCAAGCCA

GTTATCCAGGAAGTCCGAGAAATCATCCAACCCTTCCGAAAGATCACCCAGCAAATCCTC

CCCGTCCAAGAAGAAGTCTTGAGCGTGGTCTCCAAGGGCGAGCGAAAGCAGTCCATCGAA

CAGGTTGAGGTGCCAAAGGTTGAGATCCCAGAGGAGCCCGCCGCAGAGGTGCAACAGGAG

GAGCAGCAACAGTCCGAGTCCGCTCCAGCTGCCGCCCTTGCCGGTTCCGGAACTATTGTG

CAGGCTGGTCTGATCGGTGGTGCCCGAATCCTGCCCGCTGAAGACTCCGCCGCCCTGCTC

GGCTCGATCAACAACCGACCCAACAAGGGCTTCAAGACTGCCCGCCGATAA

>TRINITY_DN10330_c0_g1|m.536 TRINITY_DN10330_c0_g1|g.536 ORF TRINITY_DN10330_c0_g1|g.536 TRINITY_DN10330_c0_g1|m.536 type:5prime_partial len:845 (-) TRINITY_DN10330_c0_g1:239-2773(-)

CGCAGATCCCCCATAAACTGTGATAGTGGTTATGATATCACGATTGACCTTCCGGTAACC

GCTCATTCGCAATCGTCTATTTGGTCGGTTGGCCTGCACCGCGAGCGGTATACTAGTAGA

TTTGCAAATCAACCCCTCACAATGGCGGCGAATCCACCGAAGATCCCAATCCCCGGCAAG

CGAAACATCCTCATCACTAGCGCCTTGCCCTACGTCAACAACGTCCCCCACCTCGGCAAC

ATCATCGGATGTGTTTTGAGCGCGGATGTATTTGCGAGATATTGCCGGTTGAGGGGGTAC

AATGCAATTTACATGTGCGGGACGGATGAGTACGGGACGGCGACGGAGACGAAGGCGATG

GAGGAGAAGTGCTCCCCTAAAGAGATCTGTGATAAATACCATGCGATTCACAAGGAAGTT

TATGATTGGTTCGACATAAATTTTGATGAGTTTGGTCGCACATCCACTCCCCAGCAGACT

GAAGTTTGCCAAGCGATATTTCAAAAATTGTGGGAGAATGAATGGCTTACTGAAAAAACT

ATGGAGCAGCTTTACTGTGATACATGCAAACGCTTCTTAGCTGATCGACTAGTGGAGGGC

ATATGCCCACATTTGGACTGTAATTATGCGTCTGCGCGTGGTGACCAATGTGAAAATTGT

GGGAAGCTGTTAAATCCTACTGAGCTTAAGGATCCTAAATGCAAGGTATGTCGGACTACT

CCCCGCCTCCAAGAAACCAATCACTTGTTTCTGGAGCTCCCTTTGCTGAAGGATAAATTG

GAAGAGTACATCAACAAGATGTCAGTGGCTGGGTCTTGGAGCCAAAATGCTATTCAAACA

ACATATGCATGGTTGAAGGAAGGATTAAGGTCTCGTTGTATCACAAGGGATCTTAAATGG

GGTGTCCCTGTTCCTCATGAGAACTATAAGGATAAGGTATTTTATGTGTGGTTTGATGCT

CCAATTGGATATGTTTCTATCACATCATGCTACACACCTGAATGGGAGAAGTGGTGGAAG

AATCCTGAAAATGTCGAGTTATATCAATTCATGGGCAAGGATAACGTGCCATTTCACACG

GTCATGTTTCCTTCTACACTTCTTGGAACTGGTGAAAGCTGGACGTTAATGAAGACGATA

AGTGTGACAGAGTATTTAAACTATGAGTCAGGAAAATTCTCCAAAAGTAAAGGCATTGGA

GTTTTTGGTAATGATGCAAAGGATACAAAAATTCCTTCAGAAGTATGGCGATATTACTTG

TTAAGTAACAGGCCTGAAGTATCAGACACATTGTTCACATGGGCAGACTTGCAGGCTAAG

CTAAACAGCGAGTTGCTGAACAACTTGGGCAACTTTATTAATCGTGTTTTGAGCTTCATT

GCAAAAGCATCAGGATTGGGCTATGATTCCATCATTCCTGATGCCCATAATGCAGAATCA

CATCTGTTAACAAAGACTTTAGGTGAGACAGTTGGGAAATTGGTTGATCAATATCTTGAT

GCCATGGAGAAGGTTAAACTAAAACAAGGTTTAAAGACTGCTATGAGCATATCAAGCGAA

GGGAATGCATATTTGCAAGAGAGCCAATTTTGGAAGCTTTATAAAGAAGATCCATCTTCA

TGCTCTGTCGTTATAAAGACTTCAGCTGGACTTGTTTACCTCCTTGCGATTCTCTTAGAA

CCTTTCATGCCTTCTTTTACCGTTGAAGTTCTGAAGCAGCTTAATTTACCTCGTGAAACA

CTTCTGTCATTCTGTGATGGGGAAGGAGATACCAATAAGGCCAAAAGACCATGGGACTTT

CTATCCTCTGGTCATAAAATAGGGAAACCTGAGCCGCTGTTCAAAGAAATGAAAGATGAA

GAAGTAGAAATTTTCAGGCAGAAATTTGCTGGTAGTCAATCCGATAGATTTGAGAAAGCA

GAAGCTGAGGCAAAAAAGATTGCTGAACAGCTGAAGACCACAAAACTATCTGGCACAGAA

GCGAAAAAACAGAAGGCCAAGTCCGGCGGTGCTACAAAACCAAAGGCTACAGAGACAGAG

ATTTCTGTTTCTAGACTTGACATTCGCGTGGGTCTCATCAAGAAAATTCAGAAACATCCA

GATGCCGATTCCCTCTATGTTGAAGAGATTGATATAGGAGAAGGGTCCACACGGACAGTT

GTGAGTGGCCTTGTAAAATACATTCCTCTTGAAGAAATGCAGGATCGAAAAGTTTGTGTT

CTTTGTAATCTGAAGCCAGCGACCATGAGAGGTATTAAATCCCACGCAATGGTATTGGCT

GCATCCAATGATGACCACACCAAAGTCGAGCTAGTTGATCCGCCGCCGTCGGCCAAGGTG

GGGGAACCAGTTACCTTTCCCGGGTTTTCAGGTGAGCCAGACAGTGTCTTGAACGCCAAG

AGCAAGATTTGGGAGAAGGTGCAGGTAGATTTACACACAGATGCAGAGCTTGTTGCTCGC

TTTAAAGACGTCCCGTTCACAACATCTGCTGGAATTTGCACCGTGTCATCCATTGCTAAC

GGAGCCATTAGGTAG

>TRINITY_DN10330_c0_g1|m.537 TRINITY_DN10330_c0_g1|g.537 ORF TRINITY_DN10330_c0_g1|g.537 TRINITY_DN10330_c0_g1|m.537 type:complete len:196 (+) TRINITY_DN10330_c0_g1:72-659(+)

ATGCCAAATGGGGAAAAATCTTACATCAAAACTTCTTATTTAGGAAAACAATATCAATCT

ATAAAAATGAAAATTTTTAGACAAAACTTCTTGATGTTAAACAATGAGGTATCAGTCTAT

CAAAGAAAAACCTTATGGATGATCCGGCTGCAGCTACAATCAACAATCTACCTAATGGCT

CCGTTAGCAATGGATGACACGGTGCAAATTCCAGCAGATGTTGTGAACGGGACGTCTTTA

AAGCGAGCAACAAGCTCTGCATCTGTGTGTAAATCTACCTGCACCTTCTCCCAAATCTTG

CTCTTGGCGTTCAAGACACTGTCTGGCTCACCTGAAAACCCGGGAAAGGTAACTGGTTCC

CCCACCTTGGCCGACGGCGGCGGATCAACTAGCTCGACTTTGGTGTGGTCATCATTGGAT

GCAGCCAATACCATTGCGTGGGATTTAATACCTCTCATGGTCGCTGGCTTCAGATTACAA

AGAACACAAACTTTTCGATCCTGCATTTCTTCAAGAGGAATGTATTTTACAAGGCCACTC

ACAACTGTCCGTGTGGACCCTTCTCCTATATCAATCTCTTCAACATAG

>TRINITY_DN10330_c0_g2|m.539 TRINITY_DN10330_c0_g2|g.539 ORF TRINITY_DN10330_c0_g2|g.539 TRINITY_DN10330_c0_g2|m.539 type:5prime_partial len:686 (-) TRINITY_DN10330_c0_g2:237-2294(-)

GAACCTCTCTTTTCTCTATTCGAGTCGGTGCATCATTTAGCTGCGACTTCAAACATGGGT

TCCAAAGCCCCCATTCTCCCCGAAGAGGGCAAGCGCAACATCCTCATCACCAGCGCCTTG

CCCTACGTGAACAACATCCCCCACCTGGGCAACATCATCGGCAGTGTCCTGTCAGCCGAC

GTTTTTGCTCGCTACTGCAGAGCCCGCGGTCACAACACCATCTACATCTGCGGTTCCGAC

GAGTACGGTACCGCTACCGAGACCAAGGCCTTGGAGGAGGGCCTCTCCCCCTCCGACCTG

TGCGCCAAGTACCACGCCCTTCACAAGGGAGTCTACGACTGGTTCCGAATCGACTTTGAT

ATCTTCGGCCGAACACCGACCGAGCAACAGACCCAGATTGTCCAGCAGATCTTTTCTGAT

CTCTGGAAGAACGGCCTTATCGAGGAGCGCGAGACTACACAGCCATTCTGCGCCGACCCC

GCCCACTGCAAGTTCCTTGCTGATCGTTTCGTCGAGGGAGAGTGCAGCATCTGCCACGAC

CTTGGTGCTCGCGGAGACCAATGCGATGCCTGCGGTGGACTTTTGGACCCCTTCGAGCCC

GAACGTGAGCCCAGCAACGAGGACGACGACCATGTCGAGGCTAAGGCGACTGGCTGGCTG

ATTAACCCCCGATGCAAGGTCGACGGTACCGCCCCCGAGAAGCGGAGAACCAAGCACCTC

TACCTGCGCCTGGACGCGATTAAGGATCTGCTCGTCCCATGGTTCCACAAGAGCAGCAAG

GAGGGCGACTGGAGCGCAAACTGTATCTCCATCACCCAGGCATGGATCGACAAGGGTCTG

CTCCCCCGTGGCATCACCCGAGATCTCAAGTGGGGTGTTCCTATCCCCAAGGGTCTTGAT

GGACTCAGCGATGAGGAGTACGCCGAGAAGGTCTTCTATGTCTGGTTCGATGCCTGCATT

GGCTACGTCTCGATTACCAAGAACTTGACAGACCCCGGCAACCTGGACGGTACCAACTGG

GAGAAGTGGTGGAAGAACCCCGAGAACGTCAAGCTCTACCAGTTCATGGGCAAGGACAAT

GTGCCCTTCCACACCATCATCTTCCCTGCTTCTCAGCTTGGAACTGGAGAGAACTGGACC
[truncated: 27,365,656 more chars]
